
JOURNAL INFORMATION
==============================
NLM Title Abbreviation: JBMR Plus
Journal ID: JBMR Plus
NLM Journal ID: 101707013
Journal ID: jbmrplus

JBMR Plus

EISSN: 2473-4039
Publisher: Oxford University Press

ARTICLE INFORMATION
==============================
PMCID: PMC13403558
DOI: 10.1093/jbmrpl/ziag118
Article ID: ziag118
Article version: 1
Subjects: Abstracts, AcademicSubjects/MED00010, AcademicSubjects/MED00160, AcademicSubjects/MED00250

Abstracts of the 12th International Conference on Children's Bone Health

Publication date: 2026 Aug
Electronic publication date: 2026 Jul 27
Volume: 10
Issue: Suppl 2
First page: ii1
Last page: ii436
Copyright: © The Author(s) 2026. Published by Oxford University Press on behalf of the American Society for Bone and Mineral Research.
Copyright year: 2026
License: This is an Open Access article distributed under the terms of the Creative Commons Attribution License (https://creativecommons.org/licenses/by/4.0/), which permits unrestricted reuse, distribution, and reproduction in any medium, provided the original work is properly cited.
License URL: https://creativecommons.org/licenses/by/4.0/

Page count: 1

==============================

ICCBH 2026

12TH INTERNATIONAL CONFERENCE ON CHILDREN'S BONE HEALTH

27-30 JUNE 2026

MONTREAL | CANADA

www.theiscbh.org/iccbh2026

### Book of Abstracts

### Montreal, Canada

### Saturday 27 to Tuesday 30 June 2026

### Oral Communication Presentation Abstracts

### AHP Oral Communication Presentation Abstracts

### Printed Poster Abstracts

### Overview of the ICCBH 2026 Conference

From Saturday 27 to Tuesday 30 June 2026 a total of 528 delegates from over 45 countries attended the 12th International Conference on Children's Bone Health (ICCBH) in the beautiful city of Montreal, Canada, arranged by International Society for Children's Bone Health (ISCBH).

The meeting was held at Centre Mont-Royal, our selected conference venue in the heart of the city. The meeting was preceded by five pre-conference sessions, including the well-attended OPTIMIZE DMD and ISCBH 2026 Workshop and the ISCBH 4th Achondroplasia Workshop on Obesity both of which were held on Friday 26th June. On Saturday 27th June delegates could choose to attend either the XLH Workshop, a stimulating New Investigators Workshop and collaborative Specialised Nurse & Allied Professionals (SNAP) Workshop.

The meeting featured two very popular keynote lectures, eight Symposia covering hot topics in paediatric bone diseases, 12 Meet the Expert sessions, 34 Oral Communication presentations, 230 Posters, and 16 sponsors and exhibitors. We were also joined by many Patient Advisory groups at our new Patient Group forum which was held on Sunday.

There were five Industry Sponsored sessions throughout the meeting

The posters and exhibition hall were all located in the same space which enabled a great networking opportunity and exchange of ideas for all. In addition, dedicated "networking hives" were held towards the end of the meeting to facilitate collaborations around a variety of challenging topics.

Professor Leanne Ward (Ottawa, Canada) was the recipient of the prestigious Charles Slemenda Award for Research Excellence in the field of Pediatric Bone Disorders. Professor Ward provided an overview of her successful career and her award was presented by the ICCBH Programme Committee members, Erik Imel (Indianapolis, USA) and Outi Mäkitie (Helsinki, Finland)

Dr Noor Ul Ain (Islamabad, Pakistan) was the recipient of the ICCBH Maria Luisa Bianchi Award, introduced to recognise the work of female researchers and healthcare professionals towards the beginning of their careers, who have already made a significant contribution to the field of paediatric bone health, and who are based in low and middle income countries.

Feedback from delegates on the meeting was resoundingly positive, and the ISCBH is now actively planning its next meetings including the 4th ISCBH Bone School (25-28 May 2027, Annecy, France) and the 13th ICCBH which will be held in the Summer of 2028 in Lisbon, Portugal.

### ISCBH Executive Committee

Leanne Ward – President (Ottawa, Canada)
Adalbert Raimann – Treasurer (Vienna, Austria)
Ciara McDonnell – Secretary (Dublin, Ireland)
Frank Rauch – Past-President (Montreal, Canada)

### ISCBH Steering Committee

Alison Boyce (Bethesda, USA)
Moira Cheung (London, UK)
Antonella Forlino (Pavia, Italy)
Jeanne Franzone (Wilmington, USA)
Eric Imel (Indianapolis, USA)
Alex Ireland (Manchester, UK)
Outi Mäkitie (Helsinki, Finland)
Noriyuki Namba (Tottori, Japan)
Marie-Eve Robinson (Montreal, Canada)
Peter Simm (Melbourne, Australia)

BRIDGEBIO

*The ICCBH 2026 online abstracts are kindly supported by BridgeBio*

### ICCBH 2026 Programme Organising Committee

Erik Imel – POC Chair (Indianapolis, USA)
Janet Crane (Baltimore, USA)
Antonella Forlino (Pavia, Italy)
Corinna Grasemann (Bochum, Germany)
Alex Ireland (Manchester, UK)
Anges Linglart (Paris, France)
Cathleen Raggio (New York, USA)
Peter Simm (Melbourne, Australia)
Bettina Willie (Montreal, Canada)
EX-OFFICIO MEMBER: Jeanne Franzone (Delaware, USA) (New Investigator Chair, 2026)

### ICCBH 2026 Local Organising Committee

Marie-Eve Robinson – LOC Chair (Montreal, Canada)
Melissa Fiscaletti (Montreal, Canada)
Frank Rauch (Montreal, Canada)
Anne Marie Sbrocchi (Montreal, Canada)

### ICCBH 2026 Abstracts

Abstracts are published as submitted by the authors. All parties will make reasonable efforts to present educational subject matter in a scientific, balanced and unbiased way. However, participants must always use their own judgment and professional opinion when considering future application of this information, particularly as it may relate to patient diagnostic or treatment decisions.

Statements and descriptions are informational only and are not made or given as a warranty. The views, opinions and statements made at the conference are solely those of the presenters and may not reflect the views of the Organisers. Furthermore, participants should bear in mind that presenters may have vested interests in the concepts and products they discuss.

### Oral Communication Presentation Abstracts

### OC1.1

### Abnormal Bone Microarchitecture Phenotypes Identified in Adolescents with Unsupervised Machine Learning Methods Could Predict Suboptimal Peak Bone Mass in Early Adulthood: A Study of 1213 Adolescents

Kenneth Guangpu Yang, Eric Cheuk-Kin Kwan, Wayne Yuk-Wai Lee, Alec Lik-Hang Hung, Vivian Wing-Ying Hung, Jack Chun-Yiu Cheng, Tsz-Ping Lam, Adam Yiu-Chung Lau

The Chinese University of Hong Kong, Hong Kong, China

**Abstract**

**Objectives:**

Adolescent Idiopathic Scoliosis (AIS) has been shown to be associated with systemic low bone density and deranged bone qualities. Whether high-resolution peripheral quantitative computed tomography (HR-pQCT) in adolescence can predict impaired peak bone mass in early adulthood remains unclear. This study aimed to (a) apply unsupervised machine learning approach to cluster bone microarchitecture phenotypes from HR-pQCT data in AIS and non-AIS adolescents, and (b) evaluate peak bone mass in each phenotype after follow-up beyond 18 years old

**Methods:**

A total of 1,213 adolescents (aged 10–18 years; 602 AIS vs 611 non-AIS subjects; 963 females vs 250 males) who underwent HR-pQCT assessment of the non-dominant distal radius were included. Bone microarchitecture phenotypes were visualized using Uniform Manifold Approximation and Projection (UMAP) and clustered by a Fuzzy C-Means (FCM) model based on the HR-pQCT generated parameters. Of these, 190 AIS patients were reassessed 5–8 years beyond 18 years old.

**Results:**

Three phenotypes were identified among these subjects: Phenotype-1 (normal bone quality, n = 430), Phenotype-2 (low bone volume, n = 528), and Phenotype-3 (low cortical and trabecular density with impaired trabecular microarchitecture, n = 255). Patients with AIS had significantly lower total volumetric BMD (259.48±59.19 vs 281.40±69.17 mg/cm$^3$, P<0.001), cortical volumetric BMD, cortical bone area, cortical bone thickness, trabecular volumetric BMD, trabecular thickness, trabecular number, and significantly greater trabecular separation. Follow-up in early adulthood included 92 AIS girls in Phenotype-1 (mean age 19.3±0.8 years), 50 in Phenotype-2 (19.8±0.7 years), and 48 in Phenotype-3 (19.0±0.7 years). AIS patients with Phenotype-3 had significantly lower peak bone mass and impaired bone qualities in their early adulthood compared with the normal Phenotype-1 (Figure1).

**Conclusions:**

Unsupervised machine learning identified three distinct bone microarchitecture phenotypes in adolescents. These phenotypic features remained stable into early adulthood, with Phenotype-3 associated with suboptimal peak bone mass and impaired bone qualities. HR-pQCT-based bone microarchitecture phenotyping during adolescence may provide predictive insights into long-term bone health in AIS.

**Figure 1**

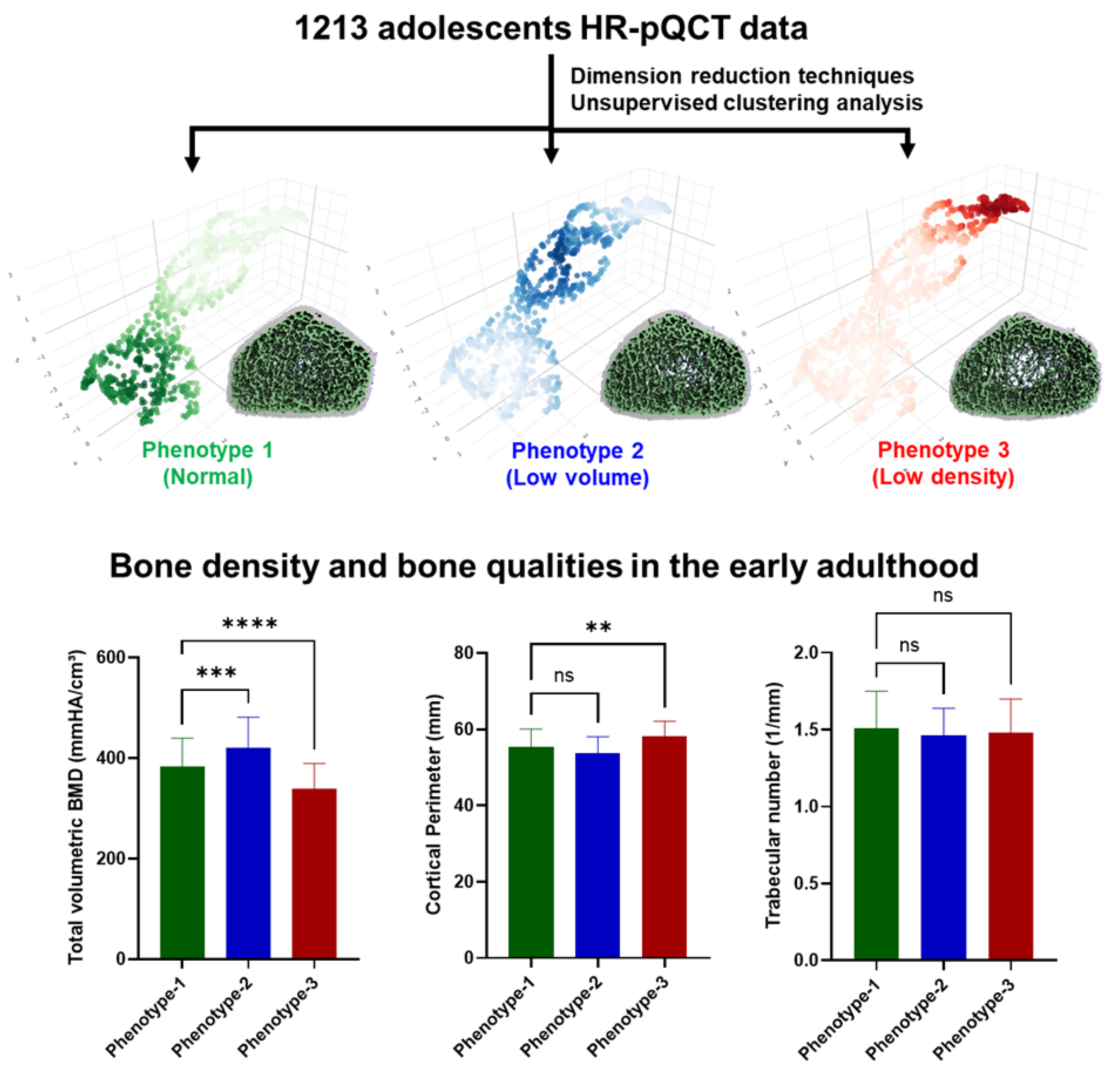

### Disclosure

None declared

### OC1.2

### Primary Osteoporosis in Cleidocranial Dysplasia: A Multicentre Paediatric Cohort Study

Ruggero Lanzafame[1], Matteo Cerutti[2], Thomas Zoller[1], Francesca Panza[1], Franco Antoniazzi[1], Angelo Pietrobelli[1,3], Ana Beleza-Meireles[4], Rhoda Akilapa[4], Ayse Pinar Ozturk[5], Rui Santos[6], Alessandra Cocca[5]

[1]University Hospital of Verona, Department of Surgical Science, Dentistry, Gynecology and Pediatrics, Verona, Italy. [2]University of Florence, Department of Health Sciences, Florence, Italy. [3]Pennington Biomedical Research Center, Baton Rouge, Louisiana, USA. [4]Guy's and St Thomas's NHS Trust, Clinical Genetics, London, UK. [5]Evelina London Children's Hospital, Paediatric Endocrine and Bone Service, London, UK. [6]Evelina London Children's Hospital, Pediatric Radiology Service, London, UK

### Abstract

**Objectives:** Cleidocranial dysplasia (CCD) is a rare skeletal dysplasia caused by haploinsufficiency of *RUNX2,* a regulator of osteoblast differentiation. We aimed to assess its role as an underrecognized cause of primary osteoporosis.

**Methods:** We conducted a multicentre observational study including 32 paediatric patients with CCD followed at Evelina London Children's Hospital (UK) and Verona University Hospital (Italy). Clinical manifestations, biochemical bone turnover markers, fracture history, and dual-energy X-ray absorptiometry (DXA) data were collected. Lumbar spine, total body less head (TBLH), and femoral bone mineral density (BMD) Z-scores were analysed using the first available DXA for each skeletal site. Reduced BMD was defined as Z-score ≤ −2; BMD of patients with height <-2 SDS was adjusted using BMAD.

**Results:** Short stature (height < −2 SDS) was observed in 12.5% (n=4/32). Delayed fontanelle closure and dental anomalies were present in 87.5% (n=28/32). ENT involvement (e.g. recurrent otitis, hearing impairment, chronic sinusitis) was reported in 37.5% (n=12/32).

A history of bone fractures was documented in 37.5% of patients (n=12/32), including vertebral fractures fulfilling ISCD criteria for paediatric osteoporosis in a subset (n=5). Bisphosphonate therapy was administered in 9.4% of patients (n=3/32), all presenting with vertebral fractures.

Bone turnover markers were available in a subgroup (n=8): alkaline phosphatase levels were below the age- and sex-specific mean in 62.5% (n=5/8); CTX in 57.1% (n=4/7); P1NP in 71.4% (n=5/7); osteocalcin in 100% (n=8/8), with values below the normal reference range in 37.5% (n=3/8).

Lumbar spine BMD Z-scores were available in 31 patients, with reduced BMD or BMAD in 41.9% (n=13/31). TBLH-BMD Z-scores were available in 24 patients, with values ≤ −2 in 45.8% (n=11/24). Femoral DXA was available in 7 patients, with reduced values in 3 cases (n=3/7). There was no statistically significant correlation between BMD Z-scores in each skeletal site and fractures: these parameters might not independently predict fracture risk.

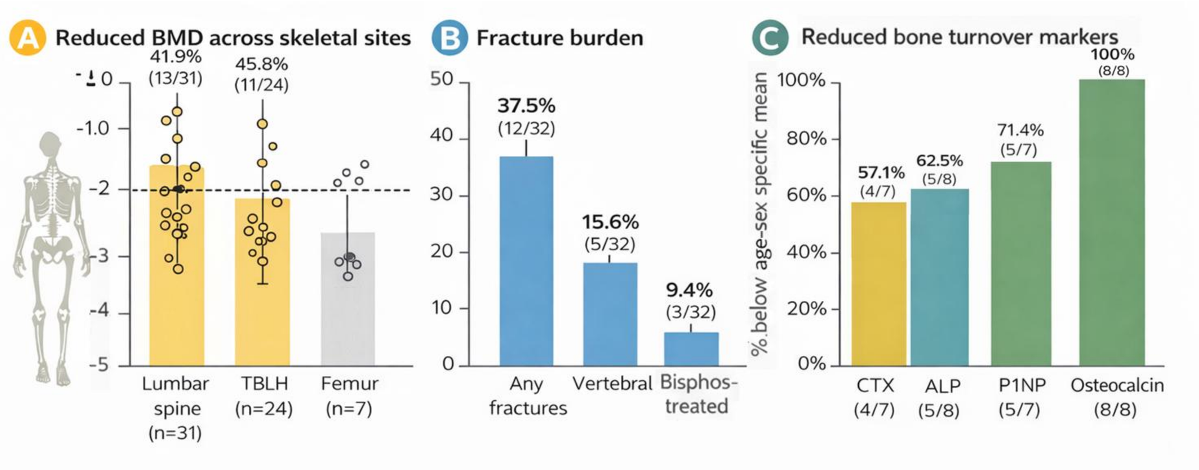

**Figure 1.** Bone mineral density, **fracture burden**, and bone turnover alterations in paediatric cleidocranial dysplasia.

**Conclusions:** This study represents one of the largest paediatric multicentre cohorts of CCD with integrated DXA, fracture, and bone turnover marker data. Our findings support routine DXA screening and structured bone health surveillance in patients with CCD, enabling early identification and prevention of osteoporosis-related complications.

### Disclosure

None declared

### OC1.3

### Trabecular Bone Score is Associated with Increased Vertebral Fracture Risk in Pediatric Patients with Glucocorticoid-Induced Osteoporosis

Funmbi Babalola[1,2], Kristin Clemens[2], Maya Scharke[3], Michael Miller[1], Scott Walker[3], Hayley Patrick[1], Ryley Yost[1], Keira Stein[1], Jinhu Ma[4], Leanne Ward[3]

[1]Children's Hospital, London Health Sciences Centre, London, Canada. [2]University of Western Ontario, London, Canada. [3]Children's Hospital of Eastern Ontario, Ottawa, Canada. [4]McMaster University, Hamilton, Canada

### Abstract

### Objectives:

Glucocorticoids (GCs), widely used to treat a variety of childhood conditions, are associated with increased vertebral fracture (VF) rates. In adults, DXA-based trabecular bone score (TBS) improved VF prediction compared to standard BMD by adding information about bone microarchitecture. We assessed the utility of TBS z-score (TBSz) alone and in combination with lumbar spine areal bone mineral density z-score (LSaBMDz) for predicting six-year cumulative VF incidence in GC-treated children ≥5 years enrolled in the Canadaian **ST**eroid-associated **O**steoporosis in the **P**ediatric **P**opulation (**STOPP**) Study.

### Methods:

TBS was calculated using Medimaps MCv19.4.0.2 (equivalent to TBS iNsight v4.0.2), with z-scores derived from available normative data. Lunar and Hologic DXA scans were analyzed separately. Logistic regression models assessed associations between baseline TBSz and LSaBMDz and six-year VF incidence; generalized mixed logistic regression models were fit to examine the influence of TBSz and LSaBMDz. Pearson correlation was used to assess associations between Spinal Deformity Index (SDI, sum of the Genant Grades from T4-L4) and TBSz.

### Results:

226 participants were included (46.5% male); mean ± SD age was 10 ± 3.6 years at baseline. 128 of participants (56.6%) had Lunar scans and 98 (43.4%) had Hologic scans. At baseline, 10% of children had at least one VF, increasing to 22% by study end.

Mean baseline TBSz was −0.60 ± 1.17 (Lunar) and −0.06 ± 0.86 (Hologic). Baseline TBSz did not significantly predict fractures over six-years in the Lunar group (p=0.223); however, in the Hologic group, a 1-unit increase in TBSz was associated with a 55% reduction in fracture risk over six years (OR 0.45; 95% CI 0.24–0.83). In regression models including TBSz and LSaBMDz, LSaBMDz significantly predicted fractures for Lunar (p=0.023), while TBSz was significant for Hologic (p=0.027). At baseline, TBSz was inversely correlated with SDI in both Lunar (*r*=-0.18, p=0.038) and Hologic (*r*=-0.27, p=0.008) groups.

### Conclusions:

TBS is a promising tool for pediatric fracture risk assessment. Lower baseline TBSz was strongly correlated with higher VF burden. On Hologic scans, higher baseline TBSz was associated with lower VF incidence, independent of LSaBMD. Additional studies to validate these findings in other disease groups with high fracture rates are warranted.

### Disclosure

None declared

### OC1.4

### Comparison of Defective Mineral Patterning in XLH and *Hyp* mouse Bone by 2D and 3D Electron Microscopy

Marc McKee[1], Catherine Chaussain[2,3,4], Agnes Linglart[5,6], Natalie Reznikov[1]

[1]McGill University, Montreal, Canada. [2]University Paris Cite, Paris, France. [3]University Sorbonne Paris Nord, Paris, France. [4]Hospital Bretonneau, Paris, France. [5]Bicetre Hospital, Paris, France. [6]University Paris-Saclay INSERM, Paris, France

### Abstract

**Objectives**:

Previously we incorporated first principles of atomic/nanoscale mineral initiation and growth to develop a symmetry-breaking notion for bone mineralization called the *Stenciling Principle*. The notion describes a default condition where all connective tissues (starting embryonically) are first prevented from mineralizing by inhibitors. Subsequently, this is followed by cell/tissue-specific expression of enzymes that degrade the inhibitors at skeletal sites as a 'biological stenciling' of mineralization. This double-negative 'inhibiting-the-inhibitors' sequence is initially enacted by small biomolecules such as pyrophosphate. Enzymatic release from inhibition (by TNAP degrading pyrophosphate) allows initiation of mineralization. This is followed by sustained regulation enacted by enzymatic degradation (by PHEX) of mineralization-inhibiting, calcium-binding phosphoproteins such as osteopontin. Mineralization initiated at the atomic/nanoscale then extends towards the microscale, further regulated by the fibrous collagen extracellular matrix, and is patterned as a 3D Voronoi-type tessellation/tiling. Here, we use 2D and tomographic 3D X-ray and electron microscopy techniques to describe and compare the defective nanoscale-to-microscale mineralization patterning in XLH adolescent human bone to *Hyp* mouse bone.

**Methods**:

To provide comparative nano/microscale information on mineralization patterning in both XLH and *Hyp* lamellar bone, focused ion beam scanning electron microscopy (FIB-SEM) and conventional 2D electron microscopy methods were used to compare the defective bone mineralization. **Results**: Here we describe in the locally oriented collagen of healthy lamellar bone volumetric simulations and direct observations of mineral growth estimating a growth rate of 1.67× preferentially along the long axis direction of collagen. This mineralization growth process generates prolate ellipsoidal mineral aggregates called *tesselles* that pack closely with one another into a microscale tessellated/tiling pattern. A clear parallelism is demonstrated between local collagen fibril texture and the preferred direction of microscale mineral growth, expansion and patterning. This crossfibrillar mineral tessellation pattern is identically defective in both adolescent XLH human bone and *Hyp* mouse bone, where decreased serum phosphate and elevated inhibitory osteopontin both contribute to delayed and incomplete mineral tessellation of bone rendering it soft (hypomineralized/osteomalacic) and deformed.

**Conclusion**.

Electron microscopy methods resolve a 3D crossfibrillar tessellation pattern in lamellar bone which is similarly defective in XLH bone and the commonly used mouse model for XLH.

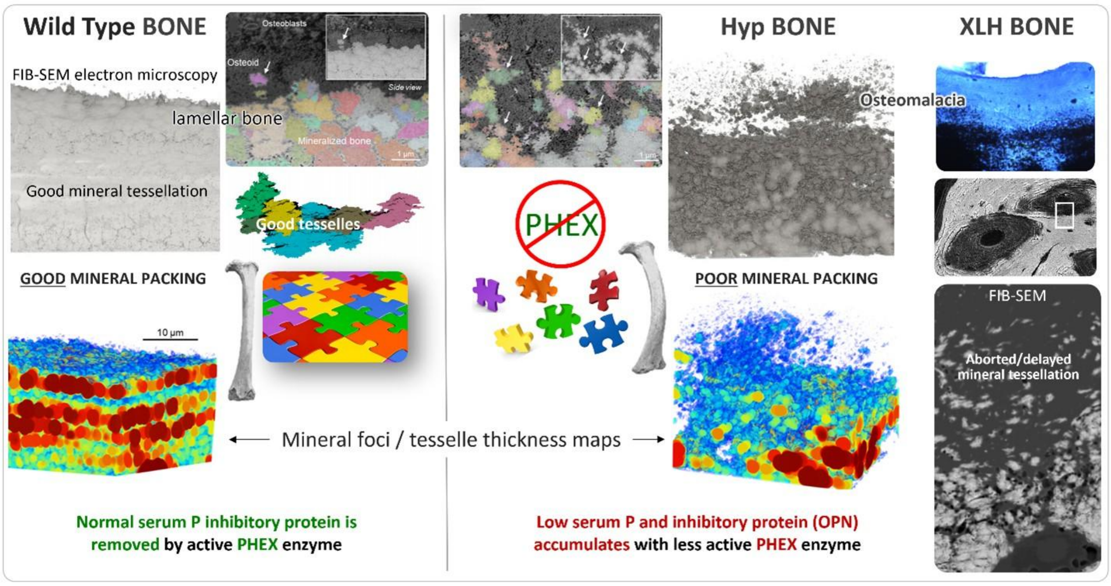

### Disclosure

Agnes Linglart (Kyowa Kirin) Marc McKee, Catherine Chaussain, Natalie Reznikov (no disclosures)

### OC1.5

### From Cilia to Spine: How POC5 Mutations Shape AIS Pathophysiology?

Pardis Behzadi[1], Amani Hassan[1,2], Hélène Mathieu[1], Florina Moldovan[1]

[1]CHU Sainte Justine, Universite de Montréal, Montreal, Canada. [2]Divisions of Plastic Surgery and Surgical and Interventional Sciences, Department of Surgery, McGill University, Montreal, Canada

### Abstract

**OBJECTIVES:**
Adolescent idiopathic scoliosis (AIS) is a three-dimensional spinal deformity affecting approximately 3% of adolescents. Although its etiology remains unclear, genetic evidence increasingly implicates dysfunction of primary cilia and centrosomes, particularly involving variants of the *POC5* centriolar protein. This study aimed to define the cellular, skeletal, and organismal consequences of *POC5* mutations and clarify their contribution to AIS pathogenesis.

**METHODS:**
We combined *in vitro* cellular analyses with *in vivo* vertebrate models. Subcellular localization, protein–protein interactions, ciliary morphology, and cell-cycle progression were examined in cells overexpressing wild-type *POC5* or the AIS-associated variant *POC5*A429V, as well as in osteoblasts derived from AIS patients. Immunoprecipitation coupled with mass spectrometry identified interaction partners, while immunolabeling with acetylated α-tubulin assessed ciliary integrity. Functional consequences were further evaluated in zebrafish loss-of-function models and CRISPR/Cas9-generated *Poc5* mutant mouse lines through histology, immunohistochemistry, and analyses of skeletal phenotype, osteogenic differentiation, proliferation, and ciliogenesis.

**RESULTS:**
Mutation of *POC5* altered centrosomal localization, induced ciliary retraction, and disrupted interactions with multiple ciliary and cytoskeletal proteins. Cells expressing *POC5*A429V showed impaired cell-cycle progression with accumulation in S-phase, reduced proliferation, and defective osteogenic differentiation. In zebrafish, retinal abnormalities were localized to the ganglion cell layer and cone photoreceptors. In mice, *Poc5* mutations caused defective ciliogenesis, impaired bone-related cellular functions, hepatic steatosis in ciliated tissues, and a moderate three-dimensional spinal deformity in a double-mutant line.

**CONCLUSIONS:**
*POC5* mutations produce convergent defects in centrosome function, primary cilia structure, cell-cycle regulation, and osteogenic activity. These findings support a model in which ciliopathy-related mechanisms contribute to AIS, with skeletal manifestations influenced by biomechanical context. *POC5*-dependent ciliary pathways therefore represent promising targets for mechanistic investigation into AIS pathogenesis.

Supported by Fondation Yves Cotrel- Institut de France

### Disclosure

None declared

### OC2.1

### Efficacy and Safety of Alkaline Phosphatase (ALP) Enzyme Replacement Therapy (ERT) Efzimfotase Alfa in Children with Hypophosphatasia (HPP): Results of MULBERRY and CHESTNUT as Part of a Three-trial Phase 3 Clinical Program

Jill H Simmons[1], Chunxiu Gong[2], Lothar Seefried[3], Jawad Hasan[4], Xiaoyu Jiang[5], Anna Petryk[6], Damien Simoneau[7], Hiroaki Wuebbenhorst[8], Derek Dunn[9], Aneal Khan[10], Kathryn M Dahir[1], Raja Padidela[11,12]

[1]Vanderbilt University Medical Center, Nashville, USA. [2]Beijing Children's Hospital, Beijing, China. [3]University of Wuerzburg, Wuerzburg, Germany. [4]Global Patient Safety, Alexion Rare Disease, AstraZeneca, Boston, USA. [5]Biostatistics, Alexion Rare Disease, AstraZeneca, Boston, USA. [6]Global Medical Affairs, Alexion Rare Disease, AstraZeneca, Boston, USA. [7]Global Medical Affairs, Alexion Rare Disease, AstraZeneca, Baar, Switzerland. [8]Clinical Development, Alexion Rare Disease, AstraZeneca, Barcelona, Spain. [9]Clinical Development, Alexion Rare Disease, AstraZeneca, Boston, USA. [10]M.A.G.I.C. (Metabolics and Genetics in Canada) Clinic, Alberta, Canada. [11]Royal Manchester Children's Hospital, Manchester, UK. [12]Faculty of Biology Medicine and Health, University of Manchester, Manchester, UK

### Abstract

**Objectives:** To evaluate the efficacy and safety of efzimfotase alfa (EFZ), an investigational ALP ERT for HPP.

**Methods:** MULBERRY was a placebo-controlled double-blind study in ERT-naive children aged 2–<12 years. CHESTNUT was an active-controlled open-label switch study from asfotase alfa in children aged 2–<12 years. EFZ dosing was subcutaneous every 2 weeks; efficacy endpoints were change from baseline to Week 25.

**Results:** MULBERRY (EFZ: 19 patients; placebo: 10) met its primary endpoint, achieving statistically significant, clinically meaningful improvement in bone health by Radiographic Global Impression of Change Scale (RGI-C) (median +1.67 [EFZ] versus 0.00 [placebo]; median-difference: 1.67; 95%CI: 0.66, 2.00; *P*=0.0003). Rickets Severity Score (RSS; key secondary endpoint) significantly improved with EFZ (median: –1.00 [EFZ] versus 0.00 [placebo]; median-difference: –1.00; 95%CI: –2.00, –0.33; *P*=0.0008). Functional and quality-of-life secondary endpoints significantly improved with EFZ: PODCI Global Function Score (median-difference: 9.00; 95%CI: 1.00, 22.00; nominal *P*=0.0234), BOT-2 Strength and Agility composite standard score (median-difference: 7.00; 95%CI: 0.00, 16.00; nominal *P*=0.0384), and EQ-5D-Y/EQ-5D-Y Proxy Version 1 Health Index score (median-difference: 0.14; 95%CI: 0.01, 0.24; nominal *P*=0.0196).

CHESTNUT (EFZ: 21; asfotase alfa: 22) met its primary endpoint of similar incidence of Treatment Emergent Adverse Events (90.5% and 86.4%, respectively) with a favorable safety profile, confirming the safety of switching from asfotase alfa to EFZ. Secondary endpoints showed maintained bone health with EFZ after switch using RGI-C (median: 0.00 [EFZ] versus 0.00 [asfotase alfa]; median-difference: 0.00) and using RSS (median: 0.00 [EFZ] versus –0.08 [asfotase alfa]; median-difference: 0.08; 95%CI: –0.17, 0.32).

Additionally, in the third Phase 3 study of this clinical program, HICKORY (placebo-controlled, double-blind study in patients ≥12 years), EFZ showed numerical improvement versus placebo for the primary endpoint (6-Minute Walk Test) without statistical significance. Among other secondary endpoints, Functional Assessment of Chronic Illness Therapy-Fatigue (FACIT-Fatigue) showed nominally significant improvements.

A lower percentage of patients experienced injection site reactions with EFZ versus registrational asfotase alfa studies.

**Conclusions:** EFZ demonstrated positive results in a broad HPP patient population with a favorable safety profile (**Table**).

**Table.** Summary of efzimfotase alfa's efficacy and safety

| **MULBERRY:** *Treatment-naive, 2–<12 y, EFZ vs pbo* |
|---|
| **Structure**<br>• Improved RGI-C (95%CI: 0.66, 2.00; *P*=0.0003)<br>• Improved RSS (95%CI: –2.00, –0.33; *P*=0.0008) |
| **Function**<br>• Improved PODCI Global Function Score (95%CI: 1.00, 22.00; nominal *P*=0.0234)<br>• Improved BOT-2[a] Strength and Agility composite standard score (0.00, 16.00; nominal *P*=0.0384)<br>• Improved EQ-5D-Y/EQ-5D-Y proxy health index score[a] (95%CI: 0.01, 0.24; nominal *P*=0.0196) |
| **Safety**<br>• Safe and well tolerated |
| **CHESTNUT:** *Switch to EFZ from asfotase alfa vs continued asfotase alfa, 2–<12 yr* |
| **Safety**<br>• Comparable TEAEs (90.5% [95%CI: 69.6%, 98.8%] vs 86.4% [95%CI: 65.1%, 97.1%]) |
| **Structure**<br>• Maintained RGI-C and RSS (median differences 0.00 [95%CI not calculated], 0.08 [95%CI: –0.17, 0.32]) |
| **Function**<br>• PODCI Global Function Score (least squares mean difference: 7.30; 95%CI: 0.66, 13.94) with greater numerical improvement in 5/6 domains from baseline vs asfotase alfa |

[a]Assessed in children ≥4 years old.
*P*-values for efzimfotase alfa (SC Q2W) vs placebo.

### Disclosure

JHS has received consulting fees from Alexion, AstraZeneca Rare Disease. CG has nothing to disclose. LS has received research funding, grant support, and honoraria for speaking, writing, and participating in educational events from Alexion, AstraZeneca Rare Disease. JH, XJ, AP, DS, HW, and DD are employees of Alexion, AstraZeneca Rare Disease and may hold stock or options in AstraZeneca. AK has received research funding from Alexion, AstraZeneca Rare Disease. KMD has received research funding and support for meetings and/or travel from Alexion, AstraZeneca Rare Disease. RP has received research funding/honoraria, consulting fees, and support for participation in advisory boards, speakers' bureau, and meetings/travel from Alexion, AstraZeneca Rare Disease.

### OC2.2

### A Randomized Controlled Trial of Oral Infigratinib in Children with Achondroplasia

Ravi Savarirayan[1], Julie Hoover-Fong[2], Melita Irving[3], Paul Arundel[4], Josep de Bergua[5], Philippe Campeau[6], Toby Candler[7], Benjamin Cocanougher[8], Valérie Cormier-Daire[9], Thomas Edouard[10], Svein Fredwall[11], Paul Harmatz[12], Daniel Hoernschemeyer[13], Henrik Irgens[14], Saumya Jamuar[15], Peter Kannu[16], Janet Legare[17], Antonio Leiva-Gea[18], Helen McDevitt[19], Roberta Onesimo[20], John Phillips III[21], Mariana del Pino[22], Marie-Eve Robinson[23], Massimiliano Rossi[24], Mars Skae[25], Leanne Ward[26], Klane White[27], Jane Schmidt[28], Ted Lystig[28], Ariana Salvatici[28], Yun Bai[28], Peter Butler[28], David van Veenhuyzen[28], Daniela Rogoff[28]

[1]Murdoch Children's Research Institute, Melbourne, Australia. [2]Johns Hopkins University, Baltimore, USA. [3]Guy's and St Thomas' NHS Foundation Trust, London, UK. [4]Sheffield Children's NHS Foundation Trust, Sheffield, UK. [5]MIKS Hospital, Vitoria-Gasteiz, Spain. [6]Centre Hospitalier Universitaire Sainte-Justine, Montreal, Canada. [7]University Hospitals Bristol and Weston NHS Foundation Trust, Bristol, UK. [8]Cincinnati Children's Hospital Medical Center, Cincinnati, USA. [9]Hôpital Necker–Enfants Malades, Paris, France. [10]Children's Hospital, Toulouse University Hospital, Toulouse, France. [11]Oslo University Hospital, Oslo, Norway. [12]UCSF Benioff Children's Hospital Oakland, Oakland, USA. [13]University of Missouri, Columbia, USA. [14]Haukeland University Hospital, Bergen, Norway. [15]KK Women's and Children's Hospital, Singapore, Singapore. [16]University of Alberta–Stollery Children's Hospital, Edmonton, Canada. [17]University of Wisconsin School of Medicine and Public Health, Madison, USA. [18]Hospital Universitario Virgen de la Victoria, Malaga, Spain. [19]NHS Greater Glasgow and Clyde, Glasgow, UK. [20]Fondazione Policlinico A Gemelli IRCCS, Rome, Italy. [21]Vanderbilt Health, Nashville, USA. [22]Hospital de Pediatría Garrahan, Buenos Aires, Argentina. [23]Children's Hospital of Eastern Ontario, University of Ottawa, Ottawa, Canada. [24]Hôpital Femme Mère Enfant, Hospices Civils de Lyon, Lyon, France. [25]Manchester University NHS Foundation Trust, Manchester, UK. [26]Children's Hospital at London Health Sciences Centre, London, Canada. [27]Children's Hospital of Colorado, Aurora, USA. [28]BridgeBio Pharma, San Francisco, USA

### Abstract

**Objectives:** Achondroplasia is a skeletal dysplasia condition characterized by disproportionate short stature and caused by pathogenic variants in *FGFR3*. Infigratinib, an orally bioavailable FGFR1–3 selective tyrosine kinase inhibitor, downregulates key pathways in the pathogenesis of achondroplasia.

**Methods:** In this phase 3, multicenter, double-blind, placebo-controlled trial (PROPEL 3, NCT06164951), children with achondroplasia were randomized to receive infigratinib at 0.25 mg/kg or placebo once daily for 52 weeks. The primary endpoint was change from baseline in annualized height velocity (AHV) at week 52. Key secondary endpoints were change from baseline in height z-score and change from baseline in upper-to-lower body segment ratio at week 52.

**Results:** A total of 114 participants underwent randomization in a 2:1 ratio to receive infigratinib or placebo. The difference between infigratinib and placebo in the least-squares mean change from baseline to week 52 was 1.74 cm/year ($p<0.0001$) for AHV, and 0.32 ($p<0.0001$) for height z-score. A trend toward a reduction in upper-to-lower body segment ratio was observed, which was most pronounced in the subgroup of children aged 3 to <8 years. Adverse events were comparable between infigratinib and placebo groups. There were no serious adverse events or discontinuations that were deemed treatment related.

**Conclusions:** In children with achondroplasia, treatment with once-daily oral infigratinib for 52 weeks led to significant increases in AHV and height z-score compared to placebo, and a trend toward improvement in upper-to-lower body segment ratio. Infigratinib was well-tolerated with no safety concerns identified.

### Disclosure

Ravi Savarirayan has received consulting fees and grants from BioMarin, has participated on advisory boards with Ascendis and BioMarin, and has received consulting fees from BridgeBio Pharma. Julie Hoover-

Fong has received grants or contracts for clinical trials from BioMarin, BridgeBio Pharma, and Pfizer, has received consulting fees from BioMarin, BridgeBio Pharma, Ascendis, Tyra and Novo Nordisk, has received honoraria from Medscape and BioMarin, and has participated on advisory boards with MCDS-Therapy Clinical Trial (unpaid), BioMarin, and BridgeBio Pharma. Melita Irving has received honoraria and travel-associated expenses from BridgeBio Pharma, BioMarin, Ascendis Pharma, and Tyra Biosciences. Paul Arundel's institution received research funding from BridgeBio Pharma and BioMarin for the conduct of studies in which he is serving and has served as Principal Investigator. Josep de Bergua declares no conflicts of interest. Philippe Campeau declares no conflicts of interest. Toby Candler declares no conflicts of interest. Benjamin Cocanougher declares no conflicts of interest. Valérie Cormier-Daire has received consultancy fees from BioMarin and BridgeBio Pharma. Thomas Edouard has received consultancy fees from BioMarin and Novo Nordisk. Svein Fredwall has participated in advisory boards and given presentations for BioMarin, Sanofi, Ascendis Pharma, BridgeBio Pharma, and Novo Nordisk, honoraria have been paid to his institution and he is a principal investigator in ongoing clinical trials for BridgeBio Pharma and Ascendis Pharma. Paul Harmatz has received research grants for clinical trials from Adrenas, Allievex, Amicus, Ascendis, Azafaros, BioMarin, BridgeBio Pharma, Denali, GC Biopharma, Idorsia, JCR, Orphazyme, Prevail, RegenXbio, Sangamo, Sanofi, and Takeda, has served as a speaker at a BioMarin-sponsored symposium, has served as a consultant for Capsida, Chiesi USA, Inc., Edigene, Grace Science Foundation, Inventiva Pharma, JCR, Novel Pharma, Orchard Therapeutics, Rallybio, Renoviron, Saliogen, Sanofi, and Sobi, Inc., and has participated in data and safety monitoring activities for Aeglea BioTherapeutics and Neurogene. Daniel Hoernschemeyer has received research support from Ascendis Pharma, BioMarin, and BridgeBio Pharma for the conduct of studies in which he served as a site Principal Investigator, is a royalty stockholder of OrthoPediatrics, and has served as a faculty speaker for Highridge Medical. Henrik Irgens declares no conflicts of interest. Saumya Jamuar declares no conflicts of interest. Peter Kannu declares no conflicts of interest. Janet Legare has served as a site Principal Investigator for Ascendis Pharma and BridgeBio Pharma, as a paid speaker for Ascendis Pharma, BioMarin, and BridgeBio Pharma, and as an advisory board member for BioMarin and Ascendis Pharma. Antonio Leiva-Gea declares no conflicts of interest. Helen McDevitt has served as a Principal Investigator for Amgen, Ascendis Pharma, BioMarin, and BridgeBio Pharma, and as a speaker for Kyowa Kirin. Roberta Onesimo has received funding as a principal investigator for clinical trials sponsored by Sanofi, BioMarin, and BridgeBio Pharma, has served as a speaker at a BioMarin-sponsored symposium, and has served as a member of a BioMarin and Ascendis Pharma advisory board. John Phillips III declares no conflicts of interest. Mariana del Pino declares no conflicts of interest. Marie-Eve Robinson has received research support from Ascendis Biopharma, Ipsen Biopharmaceuticals, and BridgeBio Pharma, and has served as a consultant for Ultragenyx, BioMarin, and Ipsen Biopharmaceuticals. Massimiliano Rossi has served as a Principal Investigator for clinical trials sponsored by BioMarin and BridgeBio Pharma, has received travel support from BioMarin and BridgeBio Pharma, has participated in advisory board activities for BioMarin, and his institution has received payment for speaking engagements from BioMarin. Mars Skae has served as a consultant for and received travel support from BridgeBio Pharma, Sanofi and Rhythm Pharmaceuticals, and received support for conference registration from Pfizer previously. Leanne Ward's institution received research support from Ascendis Pharma and BridgeBio Pharma for studies in which she served as a Principal Investigator, and consultancy fees from BioMarin and BridgeBio Pharma. Klane White has received research contracts from Ascendis Pharma, BridgeBio Pharma, Tyra Biosciences, and Ultragenyx, has received consulting fees and honoraria from BioMarin and BridgeBio Pharma, and serves as a medical board member for Little People of America, a scientific advisory board member for the National MPS Society, and a member of the board of directors for Camp Korey. Jane Schmidt, Ted Lystig, Ariana Salvatici, Yun Bai, Peter Butler, David van Veenhuyzen, and Daniela Rogoff are employees and shareholders of BridgeBio Pharma.

### OC2.3

### Growth- and Sex-Dependent Changes in Trabecular Bone, Bone Marrow Adipose Tissue, and the Lipid Microenvironment in Healthy C57BL/6 Mice

Alana Stahl[1,2,3], Michael Kuczynski[1,2], Fernanda Sousa Monteiro[4], Erica Baldesarra[5], Adriana Zardini Buzatto[4], Roman Krawetz[1,3,6], Danielle Whittier[1,2,7]

[1]McCaig Institute for Bone and Joint Health, Calgary, Canada. [2]Alberta Children's Hospital Research Institute, Calgary, Canada. [3]Department of Biomedical Engineering, University of Calgary, Calgary, Canada. [4]Department of Biological Sciences, University of Calgary, Calgary, Canada. [5]5David R. Cheriton School of Computer Science, Department of Mathematics, University of Waterloo, Waterloo, Canada. [6]Department of Biochemistry and Molecular Biology, University of Calgary, Calgary, Canada. [7]Department of Cell Biology and Anatomy, University of Calgary, Calgary, Canada

### Abstract

**OBJECTIVES:** Skeletal growth is accompanied by significant changes in the bone marrow microenvironment, particularly bone marrow adipose tissue (BMAT) expansion, yet whether these changes differ between sexes during this period remains poorly understood. We hypothesized that sex-specific differences in BMAT alongside bone microstructure would emerge during skeletal growth in healthy C57BL/6 mice.

**METHODS:** Twenty C57BL/6 mice were euthanized at 9 and 12 weeks of age (n=5 per sex per age), corresponding to a period of skeletal growth and maturation. BMAT was measured by OsO4 staining of the tibia, quantified as fat volume fraction within the marrow compartment (Fig.1A). Bone marrow lipids were extracted from femurs and analyzed by liquid chromatography-tandem mass spectrometry (LC-MS/MS), yielding 828 high-confidence lipid annotations. Trabecular bone parameters (BV/TV, Tb.N, Tb.Th, Tb.Sp, BMD) of the proximal tibial metaphysis were additionally assessed by micro computed tomography at 10 µm resolution. Statistical significance was defined as $p<0.05$.

**RESULTS:** Whole bone BMAT increased significantly in females but decreased in males between 9 and 12 weeks, while the distal region showed no significant changes in either sex (Fig.1B). Concurrently, females showed a greater decline in BV/TV, Tb.N, and BMD between 9 and 12 weeks, while Tb.Sp increased significantly with age in both sexes (Fig.1C). Lipidomics revealed broad lipid compositional changes accompanying skeletal growth, with 211 high-confidence lipids increasing between 9 and 12 weeks in both sexes, primarily phosphatidylethanolamine (n=48), sphingomyelin (n=25), phosphatidylcholine (n=23), and ceramide (n=20). No sex differences in the lipidome were detected at 9 weeks, while at 12 weeks 69 lipids differed, with triacylglycerols (n=7) and diacylglycerols (n=17) elevated in females, consistent with greater whole bone BMAT expansion.

**CONCLUSIONS:** The emergence of sex-specific differences in both whole bone BMAT and the bone marrow lipidome at 12 weeks suggests these changes are linked, driven by divergent sex-specific biology during later growth. Notably, females showed greater trabecular bone decline coinciding with BMAT expansion, consistent with their known inverse relationship. These findings help establish a normal developmental baseline, essential for understanding how disruptions during growth may contribute to long-term skeletal disease risk. Given that osteoporosis disproportionately affects women, understanding these sex-specific developmental patterns is particularly relevant.

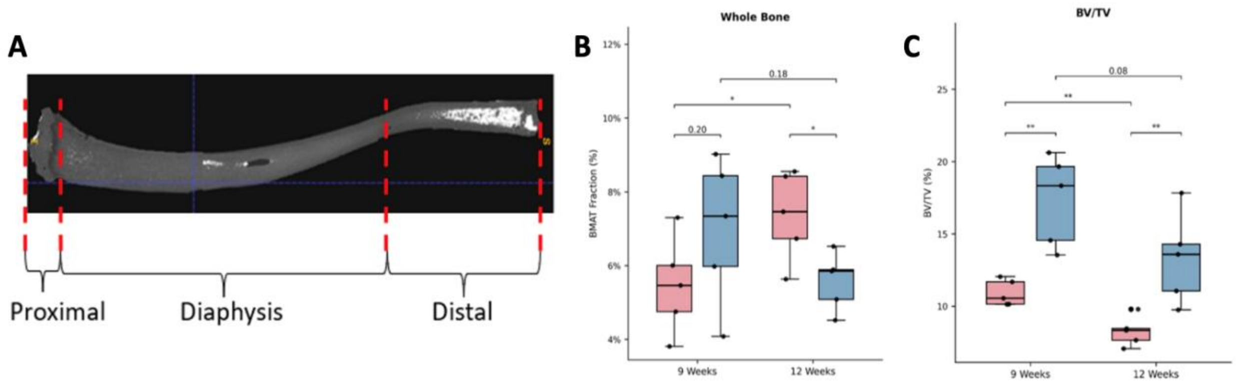

Figure 1. Growth- and sex-dependent changes in trabecular bone, bone marrow adipose tissue (BMAT), and the bone marrow lipidome in healthy C57BL/6 mice. (A) Representative µCT image of the tibia illustrating the three skeletal regions of interest: proximal metaphysis, diaphysis, and distal. (B) Whole bone BMAT fraction (%) at 9 and 12 weeks in female and male mice. (C) Trabecular bone volume fraction (BV/TV) at 9 and 12 weeks in female and male mice.

### Disclosure

None declared

### OC3.1

### Prenatal Treatment with Dabogratinib Increases Both Long Bones and Size of the Foramen Magnum and Prevents Premature Fusion of Synchondroses in a Mouse Model Mimicking Achondroplasia

Clara Lemoine[1], Jacqueline Starrett[2], Ronald Swanson[2], Laurence Legeai-Mallet[1]

[1]Université de Paris Cité, Imagine Institute, Genetics of development disorders INSERM UMR1163, Paris, France. [2]Tyra Biosciences, Carlsbad, California, USA

### Abstract

**Objectives:**

Achondroplasia (ACH) is a multi-system condition which results in disproportionate short stature, functional challenges and serious medical complications. Infants with ACH have an increased risk for death related to critical foramen magnum stenosis, which has been detected as early as the third trimester. An autosomal dominant mutation in Fibroblast Growth Factor Receptor 3 (FGFR3) causes ACH. FGFR3 gain-of-function mutations impair endochondral bone formation by disrupting both chondrogenesis and osteogenesis. Dabogratinib (formerly TYRA-300), a selective oral FGFR3 inhibitor, recently demonstrated improved growth when treated from 1 day old for 15 days in the $Fgfr3^{Y367C/+}$ mouse model which mimics the phenotype of ACH (Starrett et al., 2025). While these results represent promising therapeutic potential, the current study addresses whether additional improvement could result from initiating treatment before birth.

**Methods:**

Dabogratinib was administered daily at a 2.7 mg/kg dose in pregnant mice from embryonic day 14.5 through birth, which models the third trimester in mice, then administered to $Fgfr3^{Y367C/+}$ neonates at a 1.2 mg/kg dose from 1 day after birth for 15 days.

**Results:**

Dabogratinib treatment of $Fgfr3^{Y367C/+}$ mice resulted in a 37.4% increase in the length of the tibias and 26.3% increase in the femurs, compared with vehicle-treated $Fgfr3^{Y367C/+}$ mice. The increase in long bone size is greater when mice are treated in utero, confirming that dabogratinib crosses the placental barrier. Importantly, µCT analyses showed a significant effect of dabogratinib treatment on the area of the foramen magnum (+35.1%) and synchondroses. Analysis of the intraoccipital synchondrosis anterior revealed that while vehicle-treated mice had prematurely fused synchondroses, dabogratinib treatment resulted in open intraoccipital synchondroses (10/11 mice). We also observed reduced premature fusion of the spheno-occipital synchondroses.

**Conclusions:**

This study demonstrates that inhibiting FGFR3 prenatally can further increase bone growth in $Fgfr3^{Y367C/+}$ mice. Most importantly, prenatal plus postnatal treatment resulted in a greater increase in the area of the foramen magnum and opening of the synchondroses compared to postnatal treatment alone. Dabogratinib is currently being evaluated in a Phase 2 trial in children ages 3-10 with ACH, BEACH301 (NCT06842355). These results suggest that earlier treatment may positively impact foramen magnum stenosis in infants with ACH.

**Figure.** Increased foramen magnum area and reduced premature fusion (*) of intraoccipital synchondrosis anterior (IOSA) were observed with dabogratinib treatment.

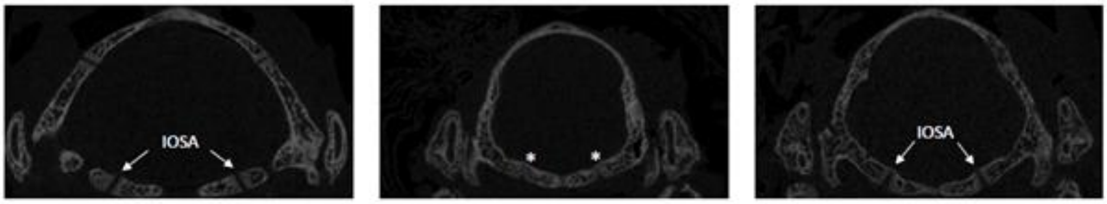

*Fgfr3*$^{+/+}$ + vehicle    *Fgfr3*$^{Y367C/+}$ + vehicle    *Fgfr3*$^{Y367C/+}$ + Dabogratinib

### Disclosure

None declared (Clara Lemoine and Laurence Legeai-Mallet). Jaqueline Starrett and Ronald Swanson are employed by Tyra Biosciences.

### OC3.2

### Continued Improvements in Lower Extremity Alignment in Navepegritide-Treated Children with Achondroplasia: Week 104 Results from the ApproaCH Trial

Leanne M Ward[1,2], Daniel G Hoernschemeyer[3], Janet Legare[4], Hanne B Hove[5], Carlos A Bacino[6], Philippe M Campeau[7], Paul Hofman[8], Josep Maria de Bergua Domingo[9], M Jennifer Abuzzahab[10], Stefan Jackowski[2], Kevin Smit[2], Andrew Tice[2], Sasha Carsen[2], Helle Vaever Hartvig[11], Laerke Clement Freiberg[11], Michael Makara[12], Michael S Ominsky[12], Aimee D Shu[12], Ravi Savarirayan[13,14,15], Ciara McDonnell[16,17]

[1]University of Ottawa, Ottawa, Ontario, Canada. [2]Children's Hospital of Eastern Ontario, Ottawa, ON, Canada. [3]University of Missouri Children's Hospital, Columbia, MO, USA. [4]University of Wisconsin School of Medicine and Public Health, Madison, WI, USA. [5]Copenhagen University Hospital Rigshospitalet, Copenhagen, Denmark. [6]Baylor College of Medicine, Houston, TX, USA. [7]CHU Sainte-Justine Research Center, Montreal, QC, Canada. [8]The Liggins Institute, University of Aukland, Auckland, New Zealand. [9]Unidad de Cirugía Artroscópica, Vithas Vitoria Hospital, Vitoria-Gasteiz, Spain. [10]Children's Minnesota, Minneapolis, MN, USA. [11]Ascendis Pharma A/S, Hellerup, Denmark. [12]Ascendis Pharma, Inc, Palo Alto, CA, USA. [13]Murdoch Children's Research Institute, Parkville, Australia. [14]Royal Children's Hospital, Parkville, Australia. [15]University of Melbourne, Parkville, Australia. [16]Children's Health Ireland at Temple Street, Dublin, Ireland. [17]University of Dublin, Trinity College, Dubline, Ireland

### Abstract

### Objectives:

In children with achondroplasia, disproportionate and reduced growth contribute to lower extremity malalignment, which is often associated with pain, altered mobility, and need for surgical intervention. Navepegritide, an investigational prodrug of C-type natriuretic peptide (CNP) administered once-weekly by subcutaneous injection, is designed to provide sustained release and continuous exposure of active CNP to counteract constitutively active fibroblast growth factor receptor 3 (FGFR3) signaling in achondroplasia. In the pivotal randomized, double-blind, placebo-controlled ApproaCH Trial, children treated with navepegritide for 52 weeks had superior annualized growth velocity (AGV) versus placebo (primary endpoint), and improved proportional growth and radiographic alignment in the lower extremities. Here we report radiographic outcomes at 104 weeks.

### Methods:

ApproaCH enrolled 84 children (aged 2-11 years) with achondroplasia. Participants were randomized 2:1 to receive navepegritide 100 mg/kg/week (n=57) or placebo (n=27) for 52 weeks, followed by 52 weeks of navepegritide treatment for all participants during the open-label extension (OLE). As an exploratory endpoint, lower extremity radiographs were assessed annually by blinded central readers using a pre-specified measurement protocol.

### Results:

Continued improvements in indices of lower extremity alignment were observed during the OLE, with decreased mean tibial-femoral angle (TFA) in children continuing navepegritide treatment, as well as those who switched from placebo to navepegritide at Week 52 (Table). The TFA improvement in the placebo-navepegritide group in the OLE was in line with navepegritide-related improvements observed at Week 52. Even greater correction was evident in children with preexisting genu varum (TFA ≥5°). In the subgroup of children with preexisting genu varum at trial baseline, mean TFA was reduced from 13.3° at baseline to 8.0° after 104 weeks of navepegritide treatment. During the OLE, fibula-to-tibia length ratio remained stable in the overall population, reflecting proportional growth of the lower leg.

**Conclusions:**

In the ApproaCH Trial, weekly administration of navepegritide continued to improve lower limb alignment in children with achondroplasia through Week 104, with greater effects in those with more pronounced genu varum. These findings suggest that through continuous systemic exposure to active CNP, navepegritide may address features of skeletal dysplasia that contribute to major health-related complications associated with achondroplasia.

**Table.**

| | Treatment group (DB period/OLE) | |
|---|---|---|
| | **navepegritide-navepegritide** | **placebo-navepegritide** |
| **Mean [SE] change in TFA (degrees), Week 0 to Week 52 (DB Period)** | | |
| All children | -1.33 [0.41] (n=54) | +0.21 [0.52] (n=26) |
| Children with TFA ≥5° at Week 0 (Baseline) | -2.07 [0.75] (n=26) | +0.51 [0.96] (n=13) |
| **Mean [SE] change in TFA (degrees), Week 52 to Week 104 (OLE)** | | |
| All children | -0.80 [0.34] (n=53) | -1.70 [0.67] (n=26) |
| Children with TFA ≥5° at Week 52 | -1.84 [0.66] (n=20) | -3.54 [0.70] (n=16) |

DB, double-blind; OLE, open label extension; SE, standard error; TFA, tibial-femoral angle
Data reported are observed mean [SE] absolute change in TFA among children with data available at both time points; negative numbers indicate improvement in TFA toward neutral alignment.

### Disclosure

LMW, DGH, JML, HBH, CAB, PMC, PLH, JMBD, MJA, RS and CM are Clinical Trial Investigators at Ascendis Pharma; HVH, LCF, MM, MO, and ADS are employees of Ascendis Pharma and hold stock in the company

### OC3.3

### Health-Related Quality of Life in Children with Achondroplasia: Findings from the Observational PROPEL Study

Marie-Eve Robinson[1], Julie Hoover-Fong[2], Josep de Bergua[3], Ravi Savarirayan[4], Saumya Jamuar[5], Thomas Edouard[6], Svein Fredwall[7], Marc Nicolino[8], Valérie Cormier-Daire[9], Mars Skae[10], Mariana del Pino[11], Roberta Onesimo[12], Katja Palm[13], Elizabeth Freas[14], Karla Mehl[14], Lixin Zhang[14], Melita Irving[15]

[1]University of Ottawa, Ottawa, Canada. [2]Johns Hopkins University, Baltimore, USA. [3]Vithas Hospital San Jose, Vitoria-Gasteiz, Spain. [4]Murdoch Children's Research Institute, Parkville, Australia. [5]KK Women's and Children's Hospital, Singapore, Singapore. [6]Children's Hospital, Toulouse University Hospital, Toulouse, France. [7]Oslo University Hospital, Oslo, Norway. [8]Hôpital Femme Mère Enfant, Hospices Civils de Lyon, Lyon, France. [9]Hôpital Necker–Enfants Malades, Paris, France. [10]Manchester University NHS Foundation Trust, Manchester, UK. [11]Hospital de Pediatría Garrahan, Buenos Aires, Argentina. [12]Fondazione Policlinico A Gemelli IRCCS, Rome, Italy. [13]Otto-von-Guericke-University, Magdeburg, Germany. [14]BridgeBio Pharma, San Francisco, USA. [15]Guy's and Saint Thomas' NHS Foundation Trust, London, UK

### Abstract

**Objectives:** Previous studies have found that achondroplasia (ACH), the most common skeletal dysplasia, is associated with reduced health-related quality of life (HRQoL) throughout the lifespan of affected individuals. HRQoL findings from children with ACH enrolled in the observational PROPEL study are presented.

**Methods:** PROPEL is a global, prospective, observational study that enrolled children aged 2.5 to <17 years at study entry with a confirmed diagnosis of ACH across Asia, Australia, Europe, North America, and South America. Participants were followed for a minimum of 6 months and a maximum of 2 years and were potentially eligible to screen for interventional studies with oral infigratinib after completing at least 6 months of observation. HRQoL was assessed using PedsQL, QoLISSY, Pain-NRS, and WeeFIM questionnaires completed by children, parents, or caregivers at baseline and every 6 months thereafter until completion of PROPEL. HRQoL outcomes were summarized using descriptive statistics.

**Results:** Among the 259 children enrolled, 135 (52.1%) were male and 124 (47.9%) were female, and the median (range) age was 6.5 years (2.4–14.6). Children and parents reported reduced HRQoL scores at baseline relative to published data from average-stature populations across instruments, and scores remained generally stable during the observational period (**Table**). Physical domains were scored lower than emotional and social domains by both children and parents. Parent-reported scores were lower than child-reported scores for PedsQL and QoLISSY, whereas children reported higher pain severity than parents on the Pain-NRS. WeeFIM scores were generally high for cognition and mobility, with lower scores observed in the self-care domain.

**Conclusions:** Children with ACH were found to have reduced HRQoL across multiple patient-reported instruments, with the physical domain most affected. Differences between child self-reports and parent proxy-reports were evident. These findings are directionally consistent with published literature and contribute to the expanding evidence base describing HRQoL in children with ACH. Interpretation of the longitudinal stability in HRQoL scores is limited by short observational follow-up and rollover into interventional studies, which may have constrained the ability to assess changes over time. Nevertheless, these results provide important contextual baseline data for interpreting future HRQoL outcomes in children treated with infigratinib.

**Table**

| Instrument, mean scores | Domain | Baseline | Month 6 | End of Study |
|---|---|---|---|---|

| | | | | |
|---|---|---|---|---|
| **PedsQL[a] (Child)** | **Total score** | **72.0** | **72.6** | **71.7** |
| | Physical functioning | 69.6 | 70.8 | 68.6 |
| | Emotional functioning | 72.5 | 72.4 | 73.4 |
| | Social functioning | 76.2 | 78.6 | 79.2 |
| | School functioning | 71.0 | 69.8 | 67.0 |
| **PedsQL[a] (Parent)** | **Total score** | **68.6** | **67.5** | **66.6** |
| | Physical functioning | 62.3 | 63.3 | 63.2 |
| | Emotional functioning | 71.0 | 67.7 | 67.9 |
| | Social functioning | 77.9 | 76.1 | 74.2 |
| | School functioning | 66.7 | 66.0 | 63.0 |
| **QoLISSY[a] (Child)** | **Total score** | **63.6** | **64.6** | **67.6** |
| | Physical | 57.8 | 57.1 | 61.4 |
| | Emotional | 69.4 | 70.7 | 73.2 |
| | Social | 63.8 | 65.9 | 68.2 |
| **QoLISSY[a] (Parent)** | **Total score** | **50.5** | **52.7** | **53.0** |
| | Physical | 39.0 | 42.3 | 41.8 |
| | Emotional | 61.7 | 61.5 | 63.3 |
| | Social | 50.8 | 54.4 | 54.2 |
| **Pain-NRS[b] (Child)** | N/A | **1.9** | **1.9** | **1.7** |
| **Pain-NRS[b] (Parent)** | N/A | **1.2** | **1.3** | **1.2** |
| **WeeFIM[a]** | **Total score** | **81.9** | **84.4** | **85.0** |
| | Cognition | 92.2 | 93.6 | 94.0 |
| | Mobility | 83.8 | 85.7 | 85.9 |
| | Self-care | 69.6 | 73.9 | 75.0 |

N/A, not applicable. [a]Scores can range from 0 to 100 (higher scores indicate better quality of life); [b]Scores can range from 0 (not pain at all) to 10 (worst pain possible).

### Disclosure

Marie-Eve Robinson has received research support from Ascendis Biopharma, Ipsen Biopharmaceuticals, and BridgeBio Pharma, and has served as a consultant for Ultragenyx, BioMarin, and Ipsen Biopharmaceuticals. Julie Hoover-Fong has received grants or contracts for clinical trials from BioMarin, BridgeBio Pharma and Pfizer, has received consulting fees from BioMarin, BridgeBio Pharma, Ascendis, Tyra and Novo Nordisk, has received honoraria from Medscape and BioMarin, and has participated on advisory boards with MCDS-Therapy Clinical Trial (unpaid), BioMarin, and BridgeBio Pharma. Josep de Bergua declares no conflicts of interest. Ravi Savarirayan has received consulting fees and grants from BioMarin, has participated on advisory boards with Ascendis and BioMarin, and has received consulting fees from BridgeBio Pharma. Saumya Jamuar declares no conflicts of interest. Thomas Edouard has received consultancy fees from BioMarin and Novo Nordisk. Svein Fredwall has participated in advisory boards and given presentations for BioMarin, Sanofi, Ascendis Pharma, BridgeBio Pharma, and Novo Nordisk, honoraria have been paid to his institution and he is a principal investigator in ongoing clinical trials for BridgeBio Pharma and Ascendis Pharma. Marc Nicolino declares no conflicts of interest. Valérie Cormier-Daire has received consultancy fees from BioMarin and BridgeBio Pharma. Mars Skae has served as a consultant for and received travel support from BridgeBio Pharma, Sanofi and Rhythm Pharmaceuticals, and received support for conference registration from Pfizer previously. Mariana del Pino declares no conflicts of interest. Roberta Onesimo has received funding as a principal investigator for clinical trials sponsored by Sanofi, BioMarin, and BridgeBio Pharma, has served as a speaker at a BioMarin-sponsored symposium, and has served as a member of a BioMarin and Ascendis Pharma advisory board. Katja Palm has received consulting payments from BioMarin and Pfizer, participated as a clinical trial manager for BioMarin, BridgeBio Pharma, and Ultragenyx, received speaker fees from BioMarin, received travel support from Novo Nordisk, Pfizer, and BioMarin, and served on advisory boards for Sanofi and BioMarin. Elizabeth Freas and Karla Mehl are employees and shareholders of BridgeBio Pharma. Lixin Zhang is an employee of BridgeBio Pharma. Melita Irving has received honoraria and travel-associated expenses from BridgeBio Pharma, BioMarin, Ascendis Pharma, and Tyra Biosciences.

### OC3.4

### Clinical Outcomes of 21 Paediatric Patients Treated with Denosumab and Zoledronate for Aneurysmal Bone Cyst (ABC)

Benjamin Jacobs, Kayleigh Hoey, Luqman Wali

Royal National Orthopaedic Hospital, London, UK

### Abstract

**Background:**

Aneurysmal Bone Cysts (ABCs) are benign but destructive tumours of osteoclast-like cells with high RANK receptor expression. Denosumab is a biologic drug which inhibits osteoclast maturation and has been used to treat ABCs which are not amenable to surgical intervention.

**Presenting Problem:**

Denosumab use in patients younger than 18 years has been restricted due to the risk of severe rebound hypercalcaemia.

**Clinical Management:**

We used Zoledronate (0.05mg/kg IV) to stabilise calcium metabolism prior to initiating Denosumab treatment, every 6 months during treatment, and once more following the final Denosumab dose. Denosumab was given in 60mg subcutaneous injections every month which was extended to two- or three-monthly intervals, depending on tumour response. Calcium metabolism was closely monitored biochemically, and tumour status was monitored clinically and radiologically.

We have now treated 21 paediatric patients (age at start of treatment 7.5 – 17.5 years, median 14.7 years). Diagnoses were confirmed by our multidisciplinary team in all cases, through clinical and radiological examination, and additionally by histology in 11 patients. All patients reported rapid symptom relief, usually from the first Zoledronate infusion, with further relief from the first Denosumab dose. All patients showed radiological response, including two patients in whom the ABC diagnosis was subsequently revised to sarcoma. All treatment has now been stopped in 11 patients (median duration off-treatment 4 years). The two sarcoma patients had further treatment with chemotherapy and surgery but the initial response to Denosumab was satisfactory. Three other patients had further treatment (sclerotherapy, radio-ablation and curettage respectively), all with good outcomes.

Adverse effects, in the form of pathological fracture and hypercalcaemia, occurred in the first four patients we treated. These have not occurred in the last 2 years, as we have optimised our treatment protocol by reducing the dose of Denosumab and ensuring regular Zoledronate infusions.

**Discussion:**

Denosumab combined with Zoledronate is effective in treating ABCs whilst mitigating rebound hypercalcaemia. Medical management is successful as a standalone treatment in most patients, and acts as a safe adjunct for other interventions, such as surgery or sclerotherapy. Long-term surveillance is required to detect tumour recurrence or adverse effects of this novel but promising treatment protocol.

### Disclosure

None declared

### OC3.5

### A Phase 2 Study of Denosumab for Prevention of Skeletal Disease Progression in Children with Fibrous Dysplasia

Vivian MacDonald, Kelly Milligan, Afua Asante-Otoo, Babak Saboury, Alison Boyce

National Institutes of Health, Bethesda, USA

### Abstract

OBJECTIVES:

Fibrous dysplasia (FD) is a mosaic disorder associated with fractures, pain, and deformities. Bone lesions appear in early childhood and expand during skeletal growth, before reaching final disease burden in adulthood. Growing evidence supports that the pro-osteoclastic factor RANKL plays a pivotal role in FD pathogenesis, and that the RANKL inhibitor denosumab can reduce lesional activity in adults. However, it is unknown if denosumab can prevent FD expansion in children. Additionally, pediatric denosumab trials have been limited by post-treatment rebound, resulting in life-threatening hypercalcemia in patients with high bone turnover disorders and open growth plates. This study evaluated the safety and efficacy of denosumab to prevent FD lesion progression in children.

METHODS:

15 children (age 5-14) were treated with denosumab for 48-weeks followed by a 28-week post-discontinuation monitoring period. Dosing ranged between 0.25-0.7 mg/kg monthly. The primary endpoint was change in FD progression over 48 weeks compared to untreated historical controls. All patients received oral calcium +/- calcitriol while on-treatment, and all received zoledronate at weeks 4 and 8 post-denosumab discontinuation.

RESULTS:

Denosumab treatment was associated with a marked decline in Skeletal Burden Score (median -7.54 units/year, IQR-9.36 to -0.76), in contrast to controls who showed a median increase of 2.12 units/year (IQR 0.81 to 2.94), p=<0.01. Bone turnover markers were elevated at baseline and declined post-treatment: CTX -74.8% (50.7-83.0%, p=<0.0001), P1NP -78.4% (64.9-85.8%, p=<0.0001), alkaline phosphatase -63.3% (58.3-76.5%, p=<0.0001). While on denosumab, 8 patients developed mild asymptomatic calcium disturbances (8 with hypocalcemia and 4 with hypercalcemia), which resolved with changes in oral supplements. At the end of the post-discontinuation monitoring period, bone turnover markers returned to pre-treatment baseline. Post-discontinuation, 4 patients developed mild asymptomatic hypercalcemia (10.5-11 mg/dL), which resolved with small interval doses of denosumab. All treatment-related adverse events were mild, and none resulted in treatment or trial withdrawal.

CONCLUSIONS:

Denosumab treatment in children with FD was well-tolerated and associated with improvement in Skeletal Burden Score compared to historical controls. Denosumab was safely discontinued without clinically significant disturbances in calcium. Results from this trial support that denosumab is a safe and effective treatment strategy to prevent FD lesion progression in children.

Figure 1.pdf

### Disclosure

This research was funded in part by the Intramural Research Programs of the NIDCR and Clinical Center, National Institutes of Health. Clinical trial NCT05419050 was conducted as an investigator sponsored study with support from Amgen Inc

### OC3.6

### Discordance Between Radiological and Biochemical Definitions of Nutritional Rickets Compared with Histologic Gold Standard at Post-Mortem

Suma Uday[1,2,3], Rui Santos[4], Marta Cohen[5], Martin Hewison[3], Carl Jenkinson[3], Fozia Shaheen[3], Tamas Marton[1], Wolfgang Högler[6,3]

[1]Birmingham Women's and Children's Hospital, Birmingham, UK. [2]Narayana Health City, Bengaluru, India. [3]University of Birmingham, Birmingham, UK. [4]Evelina Children's Hospital, London, UK. [5]Sheffield Children's Hospital, Sheffield, UK. [6]Johannes Kepler University, Linz, Austria

### Abstract

**Background/Objectives:**
Calcium deprivation evolves to nutritional rickets/osteomalacia. Therefore, detection of early biochemical and radiological signs is essential. Histologic confirmation of rickets is almost never available in clinical practice, and the extent to which biochemistry or radiology reflect true growth plate pathology remains unclear. Moreover, subtle radiologic findings such as ulna lucency (UL) are regarded as physiologic in infants, and their clinical significance remains uncertain. We aimed to evaluate concordance between imaging, biochemical markers, and histologic rickets in a paired post-mortem cohort.

**Methods:**
Thirty-one, predominantly Caucasian and formula-fed infants (median age 75 days; 19 male) with contemporaneous radiographs and rib growth plate histology were analyzed. Subjects with suspected bone pathology were excluded. Radiology and histology were independently rated by two reviewers. Histologic rickets was defined using six predefined structural components; cases were classified as positive if ≥2 components were present. Radiologic rickets severity score (RSS) and UL were recorded. 25-hydroxyvitamin-D (25OHD) (n=21/31) was analysed by LC–MS/MS at a DEQAS accredited laboratory. Radiographic abnormalities (RSS≥ 1 and/or UL), biochemical 25OHD deficiency (<30 nmol/L) and combined pathology were assessed as predictors of histologic disease. Sensitivity and specificity were calculated relative to histologic rickets using contingency tables, with 95% confidence intervals derived from exact binomial estimates.

**Results:**
Inter-rater reliability was high for histology (mean $\kappa = 0.79$) and substantial for radiologic features RSS/UL (88.9% agreement; $\kappa = 0.73$). Histologic rickets was identified in 17/31 subjects. Diagnostic performance varied across markers **(Table 1)**: RSS alone showed very low sensitivity (12%) but perfect specificity (100%), whereas UL demonstrated higher sensitivity (53%) with preserved specificity (93%). Combining RSS and UL did not alter performance. A combined radiologic and biochemical classifier improved sensitivity (68%) with modest reduction in specificity (86%). UL was significantly associated with histologic rickets (odds ratio 14.63; Fisher's exact p=0.009).

**Conclusions:**
Histologic rickets was far more prevalent than identified by radiologic or biochemical criteria. Subtle structural finding of ulna lucency may represent a meaningful disease marker rather than being a physiologic variant. Imaging and biochemical measures incompletely capture early skeletal pathology, but combining modalities improved diagnostic accuracy, supporting a multimodal approach to identifying early rickets.

**Table 1:** Diagnostic performance of radiology and biochemistry relative to gold standard histology in detecting rickets

| Marker | n | Sensitivity (95% CI) | Specificity (95% CI) |
|---|---|---|---|
| Rickets Severity Score (RSS) | 31 | 12% (1–36%) | 100% (77–100%) |
| Ulna lucency (UL) | 31 | 53% (28–77%) | 93% (66–100%) |
| Any radiologic abnormality (RSS + UL) | 31 | 53% (28–77%) | 93% (66–100%) |
| Radiologic + biochemical abnormality | 21 | 68% (43–87%) | 86% (57–98%) |

### Disclosure

None declared

### OC4.1

### Bone Marrow Adiposity in Iliac Bone Samples of Children with Osteogenesis Imperfecta Type I

Daniela Gabriel[1], Stéphane Blouin[1,2], Markus A Hartmann[1,2], Frank Rauch[3], Nadja Fratzl-Zelman[1,2]

[1]Ludwig Boltzmann Institute of Osteology at Hanusch Hospital of OEGK and AUVA Trauma Centre Meidling, 1st Med. Dept. Hanusch Hospital, Vienna, Austria. [2]Vienna Bone and Growth Center, Vienna, Austria. [3]Shriners Hospitals for Children, Montreal, Canada

### Abstract

Objective:

Bone marrow adipocytes (BMAs) are a unique fat cell type that expand after birth and with diseases. Like osteoblasts BMAs originate from mesenchymal stem cells and might critically influence bone homeostasis as their development is inversely related to bone formation. Osteogenesis Imperfecta (OI) is an inherited bone fragility disorder predominantly caused by defects in type I collagen resulting in compromised osteogenesis. Whether an imbalance between marrow adipogenesis and osteoblastogenesis contributes to the OI phenotype remains unclear. We therefore reexamined bone biopsies from children with OI type I and healthy controls, quantifying adipocytes as an additional histomorphometric parameter.

Material and Methods:

We evaluated adipocyte number and area in the bone marrow area along with histomorphometric bone formation indices on 3-5µm-thick Goldner-stained undecalcified histological sections from 10 children with OI (four quantitative, one qualitative, one splice mutation, mean age: 8.7 years, range: 2.6-14.8 years) and 10 healthy controls (mean age 8.5 years, range: 2.0-13.6 years, p=0.92). For adipocyte analysis, optical images (1.5 $mm^2$, n=3) were examined using ImageJ and the BioVoxxel Toolbox to detect adipocytes (minimal size: 520 $\mu m^2$), to calculate the adipocyte area relative to the bone marrow area, and to determine adipocyte density per $mm^2$.

Results:

Adipocytes were present in all OI samples and in 8 out of 10 control samples (none were observed in samples from a 2.1 and an 8.8-year-old child). The median adipocyte area relative to the bone marrow area was 6.7 times higher in OI than in controls but values were more variable (15.61%, range: 0.13% - 47.10%; versus 2.3%, range: 0.00% -24.54%, p=0.042). While adipocyte size measures (area, perimeter and diameter) were numerically greater in OI, high inter-individual variability meant these differences were not statistically significant. Across both groups, adipocyte area strongly correlated with adipocyte density (Spearman r=0.94, p<0.0001), but not with age or indices of bone formation (adipocyte area versus ObS/BS, Spearman r=0.40, p=0.07).

Conclusions:

Our bone biopsy study revealed a wide range of marrow adipocyte levels in healthy children, and even more variation in those with OI. The complex relationship between bone formation and marrow adipogenesis in both groups warrants further investigations.

### Disclosure

None declared

### OC4.2

### Craniofacial Effects of Zoledronic Acid in a Mouse Model of Severe Osteogenesis Imperfecta

Annika Lavoie[1], Juliana Marulanda[1,2], Frank Rauch[1,2]

[1]McGill University, Montreal, Canada. [2]Shriners Hospital for Children, Montreal, Canada

### Abstract

**Objectives:**

Moderate to severe osteogenesis imperfecta (OI) is associated with dysplasia of craniofacial bones, leading to malocclusion, temporomandibular joint dysfunction and pain. Intravenous bisphosphonates are the standard of care in children with moderate to severe OI, but the effect of this therapy on the craniofacial skeleton is unclear. This study therefore assessed the effects of zoledronic acid (ZA) on craniofacial bones in *Col1a1*$^{Jrt/+}$ mice, a model of severe dominant OI.

**Methods:**

OI mice and wild-type (WT) littermates, both male and female, received subcutaneous injections of ZA (50 µg/kg, 3x/week) or placebo from 4-10 weeks of age, an age range with rapid skeletal development (n=12-13 mice per group). After euthanasia, skulls were scanned using micro-computed tomography at an isotropic voxel size of 10 µm. Linear measurements were performed using 30 pre-established landmarks in SkyScan DataViewer. Microarchitecture was quantified in SkyScan CT Analyzer, Version 1.16.1.0. Group differences were assessed using a two-way ANOVA and Sidak's post hoc test for multiple comparisons in GraphPad Prism, Version 10.6.2.

**Results:**

OI mice had reduced transverse skull dimensions (bi-temporal distance: -6%, $p<0.0001$) and mandibular dimensions (effective mandibular length: -5%, $p<0.0001$). ZA treatment had no effect on these dimensions. The height/width ratio of mandibular condyles was significantly reduced in OI mice compared to WT littermates (-10%, $p=0.05$), resulting in a flattened condylar morphology (Figure 1C). This phenotype was rescued by ZA treatment (Figure 1D). OI mice receiving control injections had a high prevalence (42%, n=5/12) of unilateral morphological abnormalities of the mandibular condyle and surrounding temporal squamous bone, including enlargement and deformation (Figure 1E). These abnormalities were not observed in WT mice or in OI mice receiving ZA. OI mice had reduced BV/TV at the calvaria (-25%, $p=0.0001$), mandible (-41%, $p=0.0007$) and mandibular condyle (-17%, $p=0.04$). ZA treatment was associated with increased BV/TV of OI mice only at the mandibular condyle (+29%, $p=0.001$).

**Conclusions:**

ZA treatment had beneficial effects on the craniofacial skeleton of OI mice. However, improvements seemed to be limited to sites of endochondral ossification, such as the mandibular condyle, whereas intramembranous ossification sites appeared to be relatively resistant to ZA exposure.

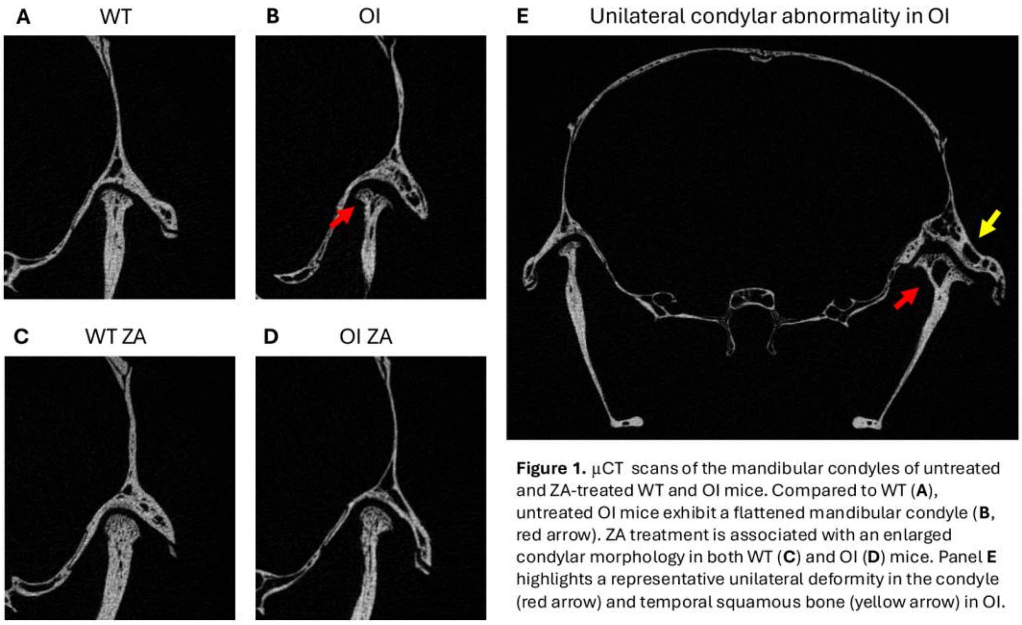

**Figure 1.** µCT scans of the mandibular condyles of untreated and ZA-treated WT and OI mice. Compared to WT (**A**), untreated OI mice exhibit a flattened mandibular condyle (**B**, red arrow). ZA treatment is associated with an enlarged condylar morphology in both WT (**C**) and OI (**D**) mice. Panel **E** highlights a representative unilateral deformity in the condyle (red arrow) and temporal squamous bone (yellow arrow) in OI.

### Disclosure

None declared

### OC4.3

### A CNP Analog Adjuvant Treatment Improves Bone Microarchitecture and Fracture Prevention in Growing Mice with Moderate-to-Severe Osteogenesis Imperfecta

Jonathan Chacko[1], Jack Mulcrone[1], Erin Carter[1], Nancy Pleshko[2], Cathleen Raggio[1]

[1]Hospital for Special Surgery, NYC, USA. [2]Temple University, Philadelphia, USA

**Abstract**

**Objectives:**

Osteogenesis imperfecta (OI) is a heterogenous type 1 collagenopathy characterized by fragile bones, decreased bone mass, and shorter stature. Currently, bisphosphonates are used to manage moderate-to-severe OI in children because they are known to reduce fracture incidence, yet they do not increase growth velocity. C-type natriuretic peptide (CNP) is produced in the growth plate and positively regulates linear bone growth; an unpublished study by Bober found that people with OI have a reduced level of CNP. This study investigates if administering the CNP analog as adjuvant treatment with a standard bisphosphonate (alendronate (ALN)) will reduce fracture incidence while increasing bone growth and improving bone mineral density (BMD) in growing *oim/oim* (OIM) mice.

**Methods:**

Two-week-old OIM female mice (N=42) were divided into three groups: mice receiving saline controls (N=8), CNP 20 ug/kg x 5 days/week alone (N=17) or CNP 20 ug/kg x 5 days/week + weekly ALN (N=17). Faxitron images were taken at 2, 8, and 14 weeks (sacrifice) to assess fracture incidence and measure femoral length and vertebral height. Microcomputed tomography was used to assess bone microstructural parameters.

**Results:**

At post-sacrifice, the CNP + ALN group had a 98.66% reduction of fracture incidence, the CNP alone group decreased 90.32%, and the control group decreased 77.78%. Femoral length was increased in both treatment groups (CNP alone: 6.31% increase, CNP + ALN: 6.23%) compared to controls. Vertebral height increased in both treatment groups (CNP alone :6.94%, CNP + ALN: 6.89%) relative to controls. Both treatment groups increased in trabecular TMD ($p \leq 0.0001$) as well as in cortical TMD ($p \leq 0.0001$) and BMD ($\leq 0.0001$) compared to controls. CNP + ALN increased cortical thickness ($p \leq 0.0001$) and trabecular BVF ($p \leq 0.0001$), trabecular number ($p \leq 0.0001$) and trabecular BMD ($p \leq 0.0001$) relative to controls.

**Conclusion:**

CNP analog adjuvant treatment greatly decreased fracture incidence at post-sacrifice, increased femoral length and vertebral height while providing added benefits for trabecular and cortical bone (not observed with bisphosphonates alone). These results indicate the potential of CNP + ALN treatment to improve femoral and vertebral length, reduce fracture incidence and increase bone thickness. Future biomechanics and FTIR spectroscopy analyses will help determine bone strength and quality.

**Figure 1. Vertebral Height**

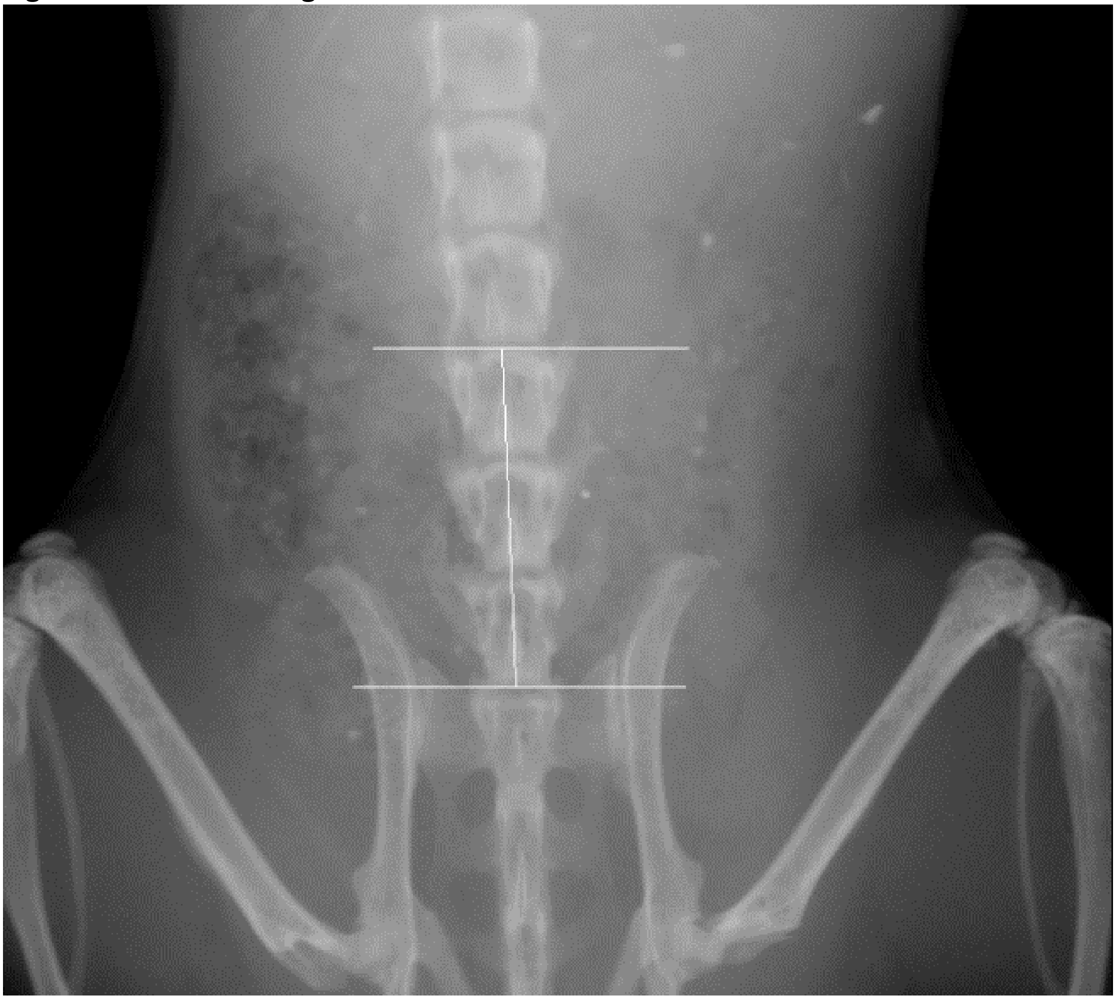

### Disclosure

No conflicts of interest

### OC4.4

### Cortical Bone Adaptive Response to Loading is Impaired in Male *Col1a1*$^{Jrt/+}$ Mouse Model of Osteogenesis Imperfecta

Abhimanyu Mertiya[1,2], Mayumi Umebayashi[1], Richard Ilianu[1], Karim Elsayed[1], Meet Shah[1,2], Michael Bruccoleri[1], Frank Rauch[1,3], Bettina Willie[1,2,4]

[1]Shriners Hospital for Children, Research Center, Montreal, Canada. [2]Faculty of Dental Medicine and Oral Health Sciences, McGill University, Montreal, Canada. [3]Department of Pediatrics, McGill University, Montreal, Canada. [4]4College of Veterinary Medicine, Utah State University, Logan, USA

### Abstract

### Objective:

Osteogenesis Imperfecta (OI) is a heritable bone fragility disorder, with most cases caused by mutations in genes encoding type I collagen. The cross-sectional area of long bones is persistently low in people with OI, and may be a key factor leading to increased long bone fracture rates. We hypothesize that this reduced bone circumference is the result of reduced periosteal apposition caused by an impaired response to mechanical loading. The **objective** of this study is to determine bone (re)modeling response to 2 weeks of *in-vivo* loading in male *Col1a1*$^{Jrt/+}$ OI mouse model.

### Methods:

Sixteen-week old male *Col1a1*$^{Jrt/+}$ OI mice and their wild type (WT) littermates (n=10 mice/genotype) underwent two weeks of *in-vivo* tibial loading to the left tibia, while right tibia served as a non-loaded control (Fig-1A). *In-vivo* microcomputed tomography analysis was performed at baseline (day 0) and day 15 to quantify changes in diaphyseal cortical and metaphyseal trabecular bone mass and microstructure. ANOVA, Tukey's post hoc, and paired t-tests were performed ($p<0.05$).

### Results:

Loading led to a 134% greater increase in cortical thickness of left limb compared to non-loaded right limb in WT mice (Fig-1B). We also observed a 118% greater decrease in marrow area of loaded limb compared to non-loaded limb in WT mice, although this was only trending towards significance ($p=0.06$, Fig-1C). Whereas no significant difference was measured in cortical thickness or marrow area between the loaded and non-loaded limbs in the *Col1a1*$^{Jrt/+}$ mice (Fig-1B & 1C). Loading did not significantly increase metaphyseal trabecular bone in WT mice, but showed a trend of increased bone deposition in trabecular bone volume (per unit total volume) in loaded limb as compared to non-loaded limb for WT mice (Fig-1D).

### Conclusion:

As hypothesized, male *Col1a1*$^{Jrt/+}$ OI mice did not increase cortical bone mass with loading, unlike WT mice. Trabecular bone was not altered in either genotype. Although previous studies with C57BL/6 mice have reported trabecular responses, this is the first study to examine FVB albino WT mice. Studies in female *Col1a1*$^{Jrt/+}$ mice are ongoing. These results indicate that the male *Col1a1*$^{Jrt/+}$ OI mouse model has a reduced cortical mechano-adaptive response.

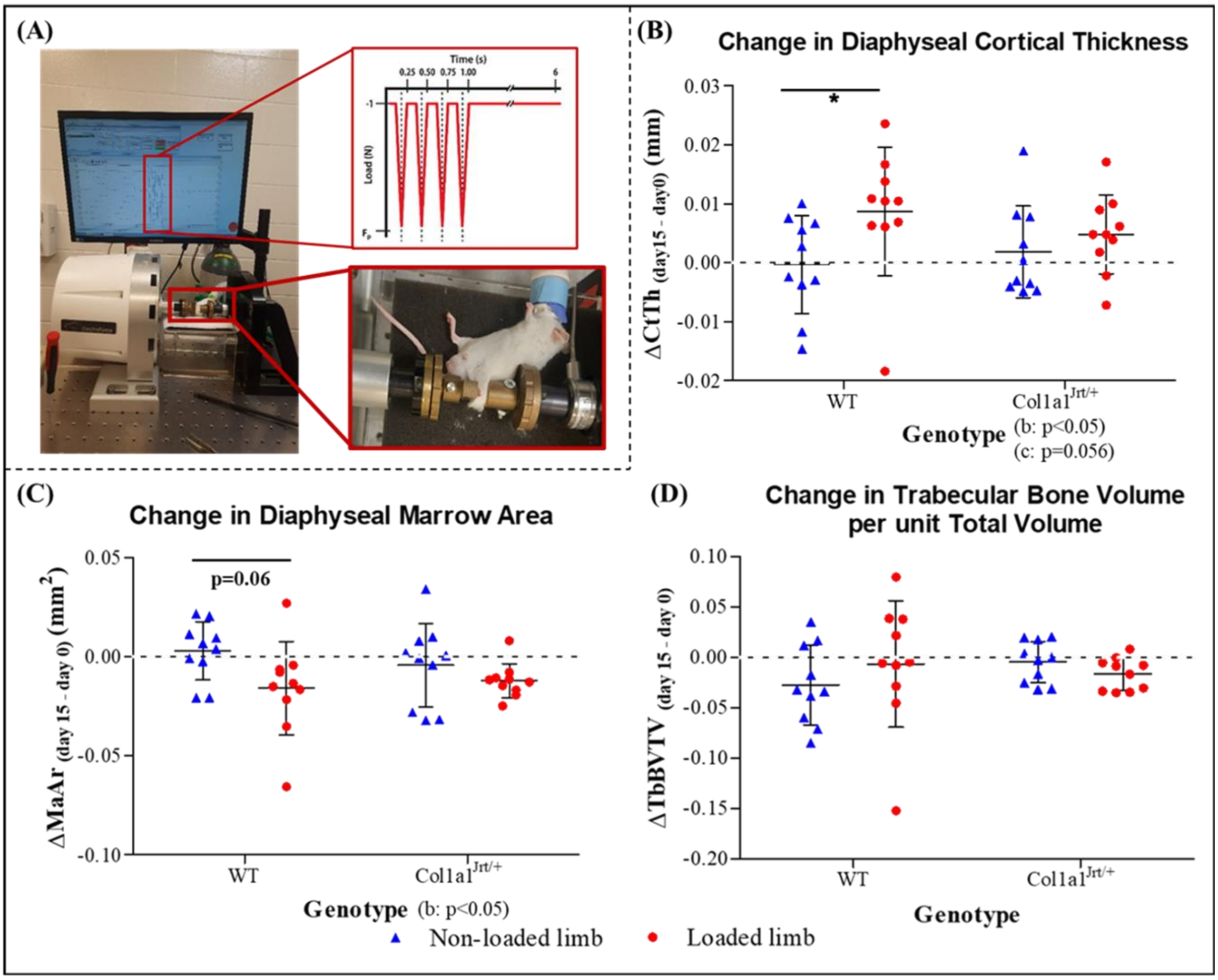

**Fig-1. Two-week *in-vivo* tibial loading experiment and results: (A) Experimental setup:** loading apparatus with mouse position shown in bottom right and loading waveform shown in upper right; **(B) change in cortical thickness (ΔCtTh $_{(day\ 15\text{-}day\ 0)}$); (C) change in marrow area (ΔMaAr $_{(day\ 15\text{-}day\ 0)}$); (D) change in trabecular bone volume per unit total volume (ΔTbBVTV $_{(day\ 15\text{-}day\ 0)}$);** Means ± SD; ANOVA (a=genotype, b=loading, c=interaction), tukey's post hoc test; paired t-test (* $p<0.05$).

### Disclosure

None declared

### OC4.5

### Vertebral and Non-Vertebral Fracture Rates in Children and Young People with Complex Osteogenesis Imperfecta in England

Paul Arundel[1], Toby Candler[2], Catherine DeVile[3], Vrinda Saraff[4], Alistair Calder[3], Nicola Crabtree[4], Amaka Offiah[5,1], Stephanie Borg[1], Manju Chandwani[2], Moira Cheung[6,3], Jacqui Clinch[2], Belinda Crowe[3], Ruchi Nadar[4]

[1]Sheffield Children's NHS Foundation Trust, Sheffield, UK. [2]University Hospitals Bristol and Weston NHS Foundation Trust, Bristol, UK. [3]Great Ormond Street Hospital for Children NHS Foundation Trust, London, UK. [4]Birmingham Women's and Children's Hospital NHS Foundation Trust, Birmingham, UK. [5]University of Sheffield, Sheffield, UK. [6]University College London, London, UK

### Abstract

**Objectives:**

Prospective fracture data are presented from a national cohort of children with osteogenesis imperfecta (OI).

**Methods:**

The Complex Childhood OI service, established 2011, is delivered across four centres, providing equitable access to multidisciplinary care for the children and young people (CYP) in England most severely affected by OI (eligibility criteria include but are not limited to multiple neonatal fractures, six or more vertebral fractures (VF), multiple limb deformities, or recessive inheritance). We estimate it sees about a quarter to a third of all CYP with OI in England. Consistent methods of prospective real-world data collection have been instituted alongside clinical practice in an iterative process. All non-VF reported here have been confirmed by radiology and/or orthopaedic opinion. Analysis of vertebral imaging (dual-energy X-ray absorptiometry and/or radiographs; typically, annual screening) was done by specialist paediatric skeletal radiologists.

**Results:**

Data are presented from the period 2016-2025. Mean (SD) number of individuals in the service per year is 328 (10). Distribution of OI types per year is: OI-I 31%; OI-IV 21%; OI-III 20%; OI-V 7%; OI-other 21%; with consistent distribution across years (SD 0-2%). Mean (SD) percentage on bisphosphonate treatment per year is 79% (4%). Mean (SD) number of non-VF per year was 211 (34). Mean (SD; CI) percentage of individuals with incident non-VF per year is 33% (4%; +/-2%). Mean (SD) number of incident VF per year (excluding COVID 19-affected years) was 161 (25). Mean (SD; CI) percentage of individuals with incident VF per year is 20% (2%; +/-3%). Mean (SD) percentage of individuals with prevalent VF per year is 62% (5%).

**Conclusions:**

Prospective longitudinal real-world data show consistent rates of non-VF and VF in a clearly defined population of CYP with OI. Missing data is a potential limitation given the clinical setting. However, we believe these data are accurate and unbiased given the prospective and consistent nature of data collection and that they relate to a geographically complete population (selected only for service eligibility criteria). Thus they provide estimates for rates of fracture in a bisphosphonate-treated population than can be used as comparative evidence in future studies.

### Disclosure

None declared

### OC4.6

### Bone Material Properties in Brtl/+ Mice Treated with a Bispecific Antibody Targeting Dickkopf-1 and Sclerostin

Nicolas Plays[1,2], Michal Kulasek[1], Catherine Julien[1], Joan Marini[3], Frank Rauch[4], Bettina Willie[1,2,5]

[1]Shriners Hospital for Children, Research Center, Montreal, Canada. [2]Faculty of Dental Medicine and Oral Health Sciences, McGill University, Montreal, Canada. [3]Section on Heritable Disorders of Bone and Extracellular Matrix, NICHD, NIH, Bethesda, USA. [4]Department of Pediatrics, McGill University, Montreal, Canada. [5]College of Veterinary Medicine, Utah State University, Logan, USA

### Abstract

**Objectives:**

Sclerostin neutralizing antibodies increased bone mass in mouse models and adults with osteogenesis imperfecta (OI). However, expression of both *Sost* and *Dkk-1* are elevated in mice after sclerostin antibody treatment, potentially dampening the therapeutic response[1]. We recently showed that a bispecific anti-sclerostin/DKK1 antibody treatment (rbsAb) leads to a significant bone mass gains in Brtl/+ mice[2]**.**In this work, we aimed to examine the effects of rbsAb on the bone porosity and material properties.

**Methods:**

Ten-week-old male and female Brtl/+ and WT mice (n=10 mice/genotype/sex/treatment) received twice weekly injections (40mg/kg) of rbsAb or PBS for 8 weeks. Collagen fiber orientation, lacunar properties and material properties were assessed at day 56. Tissue age was controlled for in the latter two analyses by segmenting the bone into 3 regions using fluorochrome labeling: intracortical (older bone), endosteal and periosteal (newly formed bone). ANOVA, Tukey and paired t-tests were performed.

**Results:**

Second harmonic generation imaging showed that the ordered collagen area normalized to total collagen area was 42% greater in rbsAb treated than vehicle WT female mice (Fig. 1A). A similar treatment-related trend was seen in Brtl/+ male (30%, p=0.07). Synchrotron tomography results showed that individual lacunar volume was 12.5-15.5% greater with rbsAb treatment for all genotypes in the periosteal region compared to vehicle and 14-21% lower in the endosteal region, except for male Brtl/+ (Fig. 1B). Lacunar number density was lower in the periosteal region of the rbsAb group compared to vehicle. Lacunar oblateness was increased in rbsAb groups across all regions. Nanoindentation in females revealed that the elastic modulus was 16% lower in rbsAb compared to the vehicle treated female Brtl/+ mice in the intracortical region, and 20% lower in the female WT mice in the endocortical region (Fig. 1C).

**Conclusions:**

The rbsAb treatment led to more ordered collagen, which is known to improve fracture resistance. Additionally, the rbsAb treatment influenced the density and shape of lacunae in both WT and Brtl/+ mice, which combined with the lower elastic modulus, likely influences the osteocytes' ability to sense mechanical stress.

**References**:

1) Holdsworth Bone, 2018; 2) Kulasesk et al ASBMR, 2025.

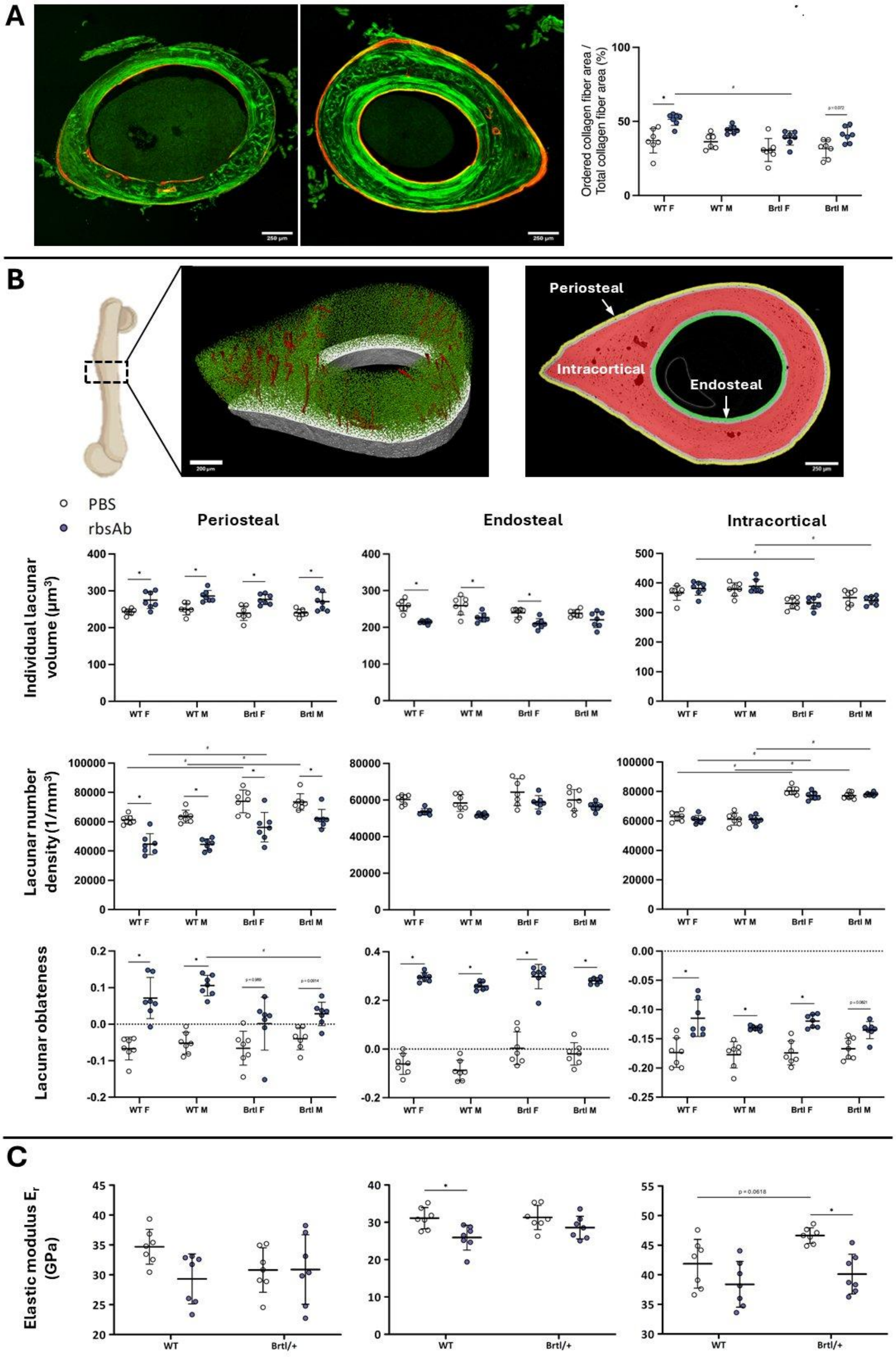

**Figure 1:** Measurements on mice femurs at mid-diaphysis. A) Second harmonic generation microscopy images of female WT PBS (left) and female Brtl/+ rbsAb (right), green is second harmonic signal generated with an 880 nm laser and imaged at 440 nm, red is calcein labelling, objective is 20x / NA = 0.8. B) Synchrotron tomography volume analyzed on the femurs at mid-diaphysis,(left; pixel size = 0.86 µm, pink beam with peak at 35 keV), lacunae and blood vessels (respectively green and red) are segmented out of the full volume. Slice showing the three regions of interest : periosteal, endosteal and intracortical (right). Lacunar oblateness describes the flatness/rod-like shape of a lacuna, from -1 (perfect rod, oblong) to 1 (perfectly flat, oblate). C) Elastic modulus (Er) aquired with nanoindentation using a Berkovich probe at 5000 µN peak load. Data for male groups is currently being analyzed.

### Disclosure

The Angitia Biopharmaceuticals company financed the present study. B.W. and F.R. act as consultants for Angitia Biopharmaceuticals.

### OC5.1

### Peak Bone Mass Development: Trabecular Bone Score and Areal Bone Mineral Density Trajectories in Mexican Children and Adolescents with TBS Version 4

Miguel Angel Guagnelli[1], Desiree Lopez-González[1], Anna Walstra[2], Madeleine Davies[2], Karen Hind[2,3], Didier Hans[2,4], Patricia Clark[1]

[1]Clinical Epidemiology Unit, Hospital Infantil de México Federico Gomez - Facultad de Medicina, Universidad Nacional Autónoma de México, Mexico City, Mexico. [2]Medimaps Group SA, Geneva, Switzerland. [3]Faculty of Health and Medicine, Lancaster University, Lancaster, UK. [4]Center of Bone Diseases, Rheumatology Unit, Bone and Joint Department, Lausanne University Hospital & University of Lausanne, Lausanne, Switzerland

### Abstract

OBJECTIVES: Achieving peak bone mass is critical for long-term skeletal health. Bone mineral accumulation and trabecular microarchitectural development may follow different biological patterns during growth. Trabecular bone score (TBS) is a DXA-derived metric reflecting trabecular bone microarchitecture and offers information that complements areal bone mineral density (BMD) measurements. This study examined age- and sex-specific patterns of lumbar spine TBS and BMD in a healthy pediatric population from Mexico City in relation to peak bone mass attainment.

METHODS: This cross-sectional analysis included lumbar spine DXA scans from 1,552 healthy individuals aged 5–18 years from Mexico City. All scans were performed using a GE Lunar iDXA system. TBS values were computed using TBS Osteo Advanced (version 4), with an adjustment for abdominal soft-tissue thickness. Both BMD and TBS measurements were stratified by sex and evaluated across the age range.

RESULTS: Data from 1,533 participants (781 females, 752 males) were analyzed. Both BMD and TBS were higher at older ages; however, distinct age-related patterns were observed. BMD showed a pronounced pubertal pattern, with the steepest age-associated differences at approximately 12–13 years in females and 13–14 years in males, followed by attenuation toward late adolescence. In contrast, TBS demonstrated smaller age-associated differences and greater variability across age groups. In females, higher TBS values were observed at younger ages relative to the age at which BMD values were highest, whereas in males, higher TBS values were observed at older ages relative to BMD. A moderate positive association between BMD and TBS was observed in females (Spearman rho = 0.651) and males (rho = 0.644).

CONCLUSIONS: TBS and areal BMD demonstrate age- and sex-specific trajectories reflecting distinct aspects of skeletal maturation contributing to peak bone mass. While BMD predominantly captures mineral content, TBS provides supplementary insight into microarchitectural integrity. Sex-specific differences in the timing of higher TBS values align with known patterns of pubertal maturation, emphasizing the necessity for age- and sex-specific normative data. Together, these findings support the combined use of TBS and BMD to improve characterization of bone health during the critical period of peak bone mass development.

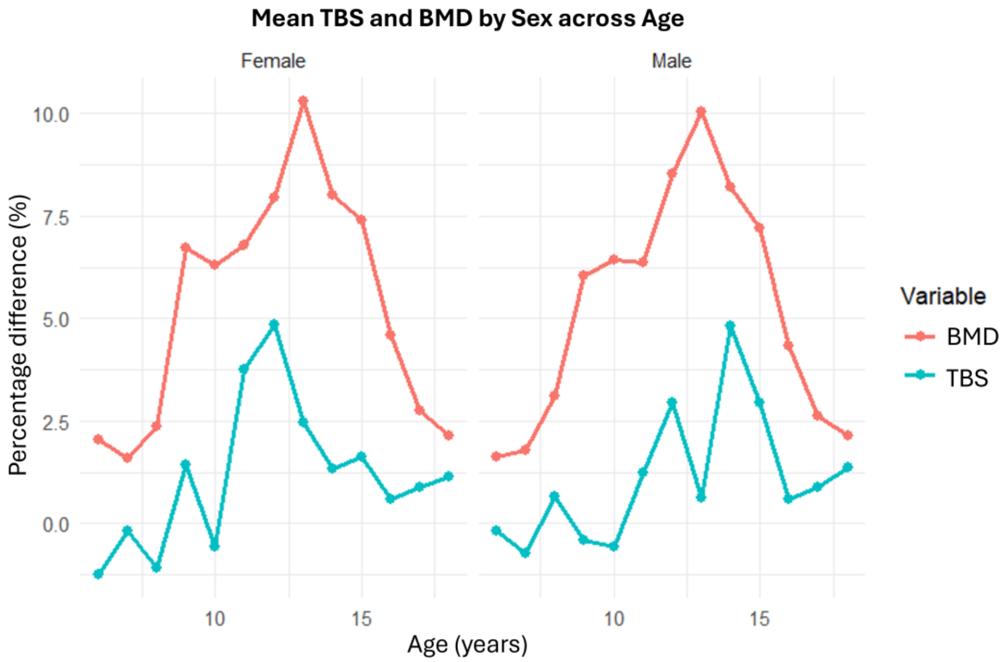

***Figure** 1 Sex-specific mean trabecular bone score (TBS; blue) and areal bone mineral density (BMD; red) across ages 5 to 18*

### Disclosure

MG, DLG and PC declare they have no conflicts of interest. AW, MD and KH are employees of Medimaps Group SA, developers of TBS iNsight™ software. DH is co-owner of the TBS patent, has corresponding shares, and is part-time CEO at Medimaps Group.

### OC5.2

### Bone Adaptation and Impaired Muscle Function in Overweight Rural Indian Children: Implications for Fracture Risk

Sharvani Patil[1,2], Rubina Mandlik[1,2], Dipali Ladkat[1], Nikhil Shah[1,3], Vivek Patwardhan[1], Anuradha Khadilkar[1,2]

[1]Hirabai Cowasji Jehangir Medical Research Institute, Jehangir Hospital, Pune, India. [2]2- School of Health Sciences Savitribai Phule Pune University, Pune, India. [3]Department of Pediatric Endocrinology, MRR Children's Hospital, Mumbai, India

**Abstract**

Background: Body composition, bone parameters, body composition and muscle function have been widely studied across pediatric body mass index (BMI) categories. Both under-nutrition and overweight are associated with impaired muscle function, altered bone health, and increased fracture risk, yet these outcomes have largely been examined in isolation. Integrated evidence on muscle–bone interactions and muscle composition, particularly intramuscular adipose tissue, across BMI categories remains limited, especially in nutritionally transitional populations. We aimed to examine muscle function, bone parameters, and intramuscular adipose fat (IMAT) to elucidate how muscle–bone–fat relationships differ across BMI categories in a nutritionally transitional setting.

Methods: This was a cross-sectional study among 422 rural Indian children aged 8-10 years. Anthropometry was assessed. BMI was categorized using ethnic specific Z-scores. Bone health, IMAT, and muscle function parameters were assessed using pQCT (XCT-2000 scanner, Stratec, Inc; Pforzheim, Germany) and Leonardo Mechanograph Ground Reaction Force Plate (Novotec Medical, Pforzheim, Germany), respectively; muscle function parameters were weight-adjusted. Fracture history was recorded.

Results: Among 422 children, 11.6% were underweight, 73.9% normal weight, and 14.5% overweight. Bone density geometry and strength metrics, including trabecular density, cortical bone mineral content, endosteal circumference, periosteal circumference, and the stress-strain index were markedly elevated in overweight children when contrasted with their normal-weight and underweight counterparts ($p < 0.05$; Table 1). Weight-adjusted muscle function parameters, including hand-grip strength, maximum force, and maximum power, differed significantly across BMI categories ($p < 0.05$), with progressively lower values observed in overweight children. This divergence suggests disproportionate gains in bone parameters relative to muscle function across BMI categories. Overweight children had a higher prevalence of fractures (9.9%) as compared to underweight (2%) and normal weight (6.7%)

Conclusion: Despite higher bone mass, estimated strength, and favourable geometry, overweight children demonstrated reduced muscle function and higher intramuscular fat, suggesting altered muscle–bone interactions. This apparent mismatch between bone adaptation and muscle performance may contribute to functional vulnerability during falls. The observed fracture prevalence in overweight children supports the need to consider muscle quality alongside bone measures when evaluating musculoskeletal health in children undergoing nutritional transition.

Table 1: Bone and muscle parameters by weight category in rural Indian children.

| Parameters | Normal Weight (N=312) | Underweight (N=49) | Overweight (N=61) |
|---|---|---|---|
| Trabecular density (mg/$cm_3$)[b] | 174.6±31.7 | 169.0±31.8 | 187.0±27.0 |
| Cortical bone mineral content(mg/$cm_3$)[b] | 3.6±0.8 | 3.5±0.6 | 4.1±0.9 |
| Endosteal circumference(mm)a | 26.2±3.8 | 24.7±2.6 | 27.3±3.6 |
| Periosteal circumference(mm)[ab] | 34.0±3.0 | 32.4±1.9 | 35.7±2.8 |
| Stress strain Index(mm3)[ab] | 121.8±31.3 | 110.3±21.2 | 142.5±34.2 |
| Intramuscular fat (%)[ab] | 26.3±8.1 | 19.3±6.6 | 42.5±8.3 |
| Hand grip strength (kg)[b] | 11.4±2.6 | 10.1±1.7 | 13.2±3.3 |
| Maximum force(N/kg)[b] | 2.7±0.29 | 2.8±0.2 | 2.6±0.2 |
| Maximum power(N/kg)[b] | 34.9±4.3 | 35.8±4.1 | 30.8±3.7 |
| Reported fractures(%) | 6.7 | 2 | 9.9 |

Footnote - All the mentioned parameters had significantly(*$p<0.05$)* different means between groups (*$p<0.05$).* a= significant difference between underweight and normal weight children. b= significant difference between overweight and normal weight children.

**Disclosure**

None declared

### OC5.3

### The Muscle-Bone Unit in Children and Adolescents: Insights from the Canadian Health Measures Survey

Oliver Li[1], Leanne M Ward[2], Lauren E Griffith[1], Laura N Anderson[1], Andy Kin On Wong[3], Jonathan D Adachi[1], Jinhui Ma[1], Frank Rauch[4]

[1]McMaster University, Hamilton, Canada. [2]Children's Hospital of Eastern Ontario, Ottawa, Canada. [3]University Health Network, Toronto, Canada. [4]Shriners Hospital for Children Canada, Montreal, Canada

### Abstract

OBJECTIVES:

Muscle and bone are closely related during growth and development. We sought to identify determinants of the muscle-bone relationship using peripheral quantitative computed tomography (pQCT) at the distal lower extremity and jumping mechanography among Canadian children and youth.

METHODS:

Data were obtained from the Canadian Health Measures Survey, a national, cross-sectional, population-based study. 3,225 participants (age 6 to 19.9 years) had valid pQCT measures available for analysis. We used calf muscle cross-sectional area (CSA; 66% site), a measure correlated with muscle strength, and bone mineral content (BMC) of the tibia shaft cross-section (38% site), a measure associated with bone strength. Maximum jumping power (Pmax) from the single two-legged jump was assessed as a potential mediator of the association between muscle CSA and BMC z-scores using path analysis.

RESULTS:

We found a strong positive association between muscle CSA z-score and tibia BMC z-score. A muscle CSA z-score change of 1.00 was associated with a BMC z-score change of 0.64 (95% CI: 0.62 to 0.67). Calf subcutaneous fat CSA z-score (66% site), muscle density z-score (a measure of intramuscular fat), and body mass index (BMI) z-score were determinants of the association between muscle CSA z-score and BMC z-score. Ethnicity (White vs. non-White), self-reported moderate-to-vigorous physical activity, and serum concentrations of total calcium, 25-hydroxy-vitamin D3, and parathyroid hormone did not reach the threshold of being classified as determinants of the muscle-bone relationship. Pmax z-score was a partial mediator of the association between muscle CSA z-score and BMC z-score, even after adjustment for z-scores of muscle density, fat CSA, or BMI.

CONCLUSIONS:

In conclusion, we found that the effect of muscle size on bone mass was in part mediated by muscle power. Both subcutaneous and intramuscular fat were determinants of the muscle-bone relationship and were related to lower bone mass.

### Disclosure

None declared

### OC5.4

### Suture Fusion Stage-Dependent Bone Structural, Material and Osteocyte Lacunar Changes in Non-Syndromic Craniosynostosis

Taylor deVet[1,2], Maximilian Rummler[3], Aren Bezdjian[4], Samantha McCluskey[4], Paul Zaslansky[5], Markus Hartmann[6,7], Stéphane Blouin[6,7], Nadja Fratzl-Zelman[6,7], Richard Weinkamer[3], Roy Dudley[4], Bettina Willie[1,2,8]

[1]McGill, Montreal, Canada. [2]Shriners Hospital for Children Canada, Montreal, Canada. [3]Max Planck Institute of Colloids and Interfaces, Potsdam, Germany. [4]McGill University, Montreal, Canada. [5]Charité-Universitätsmedizin, Berlin, Germany. [6]Ludwig Boltzmann Institute of Osteology at the Hanusch Hospital of OEGK and AUVA Trauma Centre Meidling, Vienna, Austria. [7]Vienna Bone and Growth Center, Vienna, Austria. [8]Utah State University, Logan, USA

### Abstract

Non-syndromic craniosynostosis (NSC) is characterized by premature fusion of cranial sutures in the absence of syndromic features, leading to abnormal skull growth and neurodevelopmental complications. Despite its clinical prevalence, etiology and mechanobiology underlying NSC remain poorly understood. This study used a multi-modal imaging approach to characterize structural, compositional, and cellular changes in cranial bone across different stages of sagittal suture fusion in NSC.

Cranial bone obtained during craniectomy from six infants (ages 3–8 months) was analyzed using micro-computed tomography, confocal laser scanning microscopy, second-harmonic generation microscopy, quantitative backscattered electron imaging, synchrotron radiation-based tomography, and histology. Sutures were classified as patent, fusing, or fused based on micro-computed tomography images. Microstructure, mineralization, collagen organization, and osteocyte lacunar properties were quantified adjacent to the suture (peri-suture bone) and 2 mm from the suture (peripheral bone), in both primary woven and secondary lamellar bone.

Trabecular bone structure was largely similar between peri-suture and peripheral regions, irrespective of fusion status. Consistent with advanced bone maturation, bone surrounding fused sutures exhibited lower heterogeneity in mineralization (CaWidth -10%) compared to patent sutures. Second-harmonic generation imaging revealed that patent sutures exhibited highly disorganized collagen fibers and as fusion progressed, fibrous tissue was replaced by primary woven bone and subsequently remodeled into organized lamellar bone. In accordance, histological analysis confirmed active osteogenesis with advancing fusion.

Osteocyte lacunar orientation was significantly altered adjacent to fused sutures compared to fusing and patent sutures within lamellar bone (+10%). Lacunar number density was consistently lower (-35%) and lacunar volume (23%) was higher in peri-suture bone compared to peripheral bone. These changes were more pronounced in lamellar than in woven bone consistent with ongoing bone remodeling.

Overall, NSC progression is associated with localized changes in bone mineralization, collagen organization, and osteocyte morphology. The alteration of osteocyte lacunae orientation dependent on suture fusion status is of particular interest as this may indicate a disrupted mechanosensitive adaptive ability of bone, resulting in accumulation of more disorganized bone as the suture fuses too quickly. These findings underscore the importance of local mechanobiological and cellular processes in driving suture fusion, providing new insights into NSC pathophysiology.

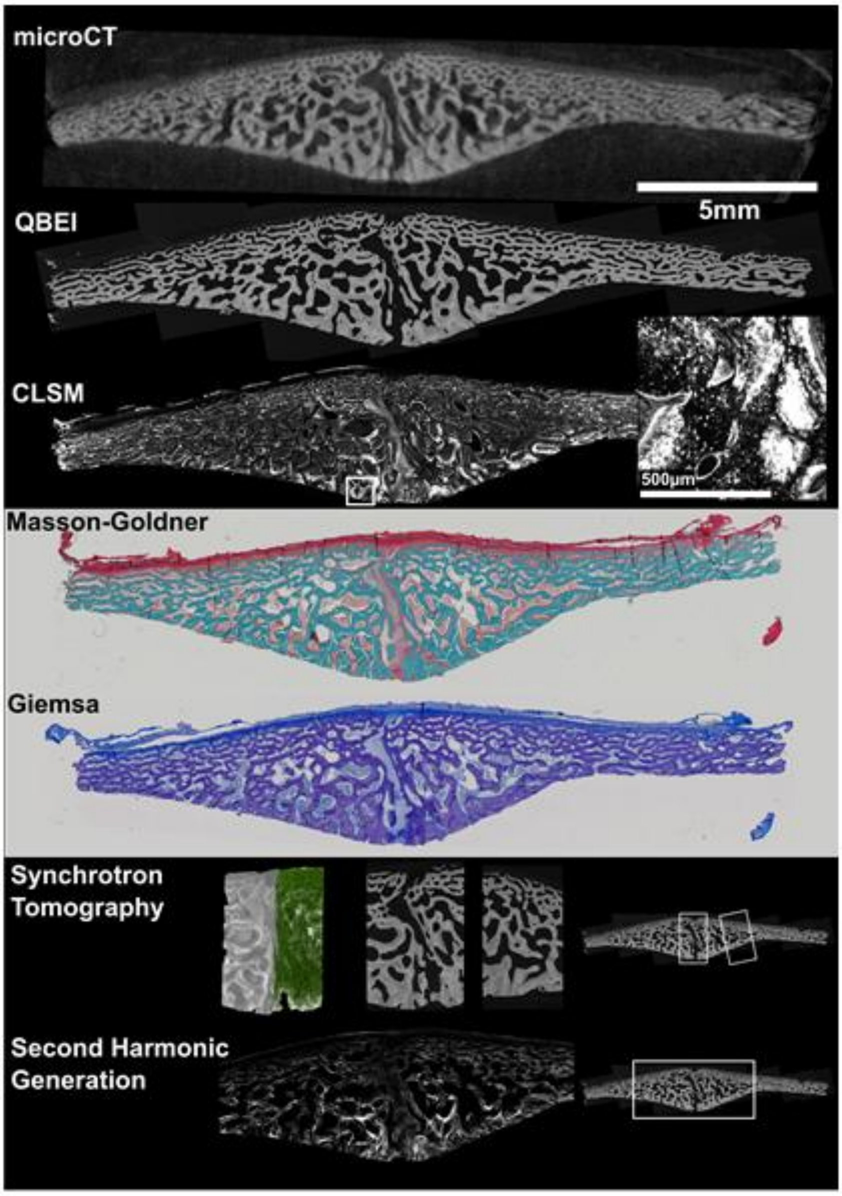

Figure 1: Overview of analysis techniques of craniosynostosis samples including microcomputer tomography (microCT), quantitative backscattered electron imaging (qBEI), confocal laser scanning microscopy (CLSM), histology on Masson-Goldner and Giemsa-stained samples, synchrotron tomography and second harmonic generation imaging.

### Disclosure

None declared

### OC5.5

### Laplace-Hamming Segmentation Changes Clinical Interpretation of HR-pQCT Microarchitecture and Strength Outcomes in Children with Osteogenesis Imperfecta and Healthy Controls

Seyedmahdi Hosseinitabatabaei[1,2], Minhao Zhou[3], Frank Rauch[1,4], Galateia Kazakia[3], Bettina Willie[1,2,5]

[1]Shriners Hospital for Children, Research Center, Montreal, Canada. [2]Department of Bioengineering, McGill University, Montreal, Canada. [3]Department of Radiology and Biomedical Imaging, University of California, San Francisco, San Francisco, USA. [4]Department of Pediatrics, McGill University, Montreal, Canada. [5]College of Veterinary Medicine, Utah State University, Logan, USA

### Abstract

**Objectives:**

High-resolution peripheral-quantitative CT (HR-pQCT) combined with micro-finite element (µFE) models is a powerful tool for assessing bone quality and fracture risk. A Laplace-Hamming (LH) segmentation method has improved microarchitectural accuracy in adults (Sadoughi et al. JBMR 2023). We compared HR-pQCT outcomes obtained using LH and standard segmentation methods in children with osteogenesis imperfecta (OI) and healthy controls (Hosseinitabatabaei et al. JBMR 2025).

**Methods:**

Radius and tibia scans from 20 children with OI and 20 age- and sex-matched controls were obtained using a second-generation HR-pQCT scanner. Microarchitectural and µFE outcomes were derived using both the default and LH segmentation. Absolute differences between segmentation methods were assessed using paired t-tests with Benjamini-Hochberg correction. Clinical impact was evaluated by comparing baseline OI vs control differences and 1-year changes using linear mixed-models.

**Results:**

All outcomes differed significantly between segmentation methods ($p<0.05$). Visual inspection revealed LH as a more accurate alternative for clinical HR-pQCT evaluation (Figure). Cortical porosity (Ct.Po) showed the largest discrepancy, with LH producing 700% (tibia) and 1250% (radius) higher values. Trabecular outcomes differed systematically: LH yielded 9% higher trabecular bone volume fraction (Tb.BV/TV), 15% higher number (Tb.N), and 9% lower thickness (Tb.Th). Cortical thickness (Ct.Th) decreased with LH, and estimated bone strength was 6–8% lower. Importantly, LH segmentation altered clinical interpretations. At tibia, only LH detected significant between-group differences in 1-year changes for Ct.Po and Tb.BV/TV (LH: Tb.BV/TV in controls increased form an average 25.3% to 26.9% while OI decreased from 17.7% to 17.2%; default: controls from 26.5% to 27.5%, OI from 14.9% to 14.6%). Baseline differences in Tb.N was no longer significant with LH. At radius, only LH detected significant group differences in 1-year changes for Tb.Th, Ct.Po, and Tb.BV/TV (LH: controls from an average 22.0% to 23.8%, OI from 16.6% to 16.4%; default: controls from 21.9% to 22.9%, OI from 15.3% to 15.0%), and detected baseline differences in Tb.Th and Ct.Th that were not observed using the default method.

**Conclusions:**

For these second-generation HR-pQCT images, LH segmentation led to different clinical interpretations for several outcomes. Tb.BV/TV 1-year changes nearly doubled in magnitude in controls using LH.

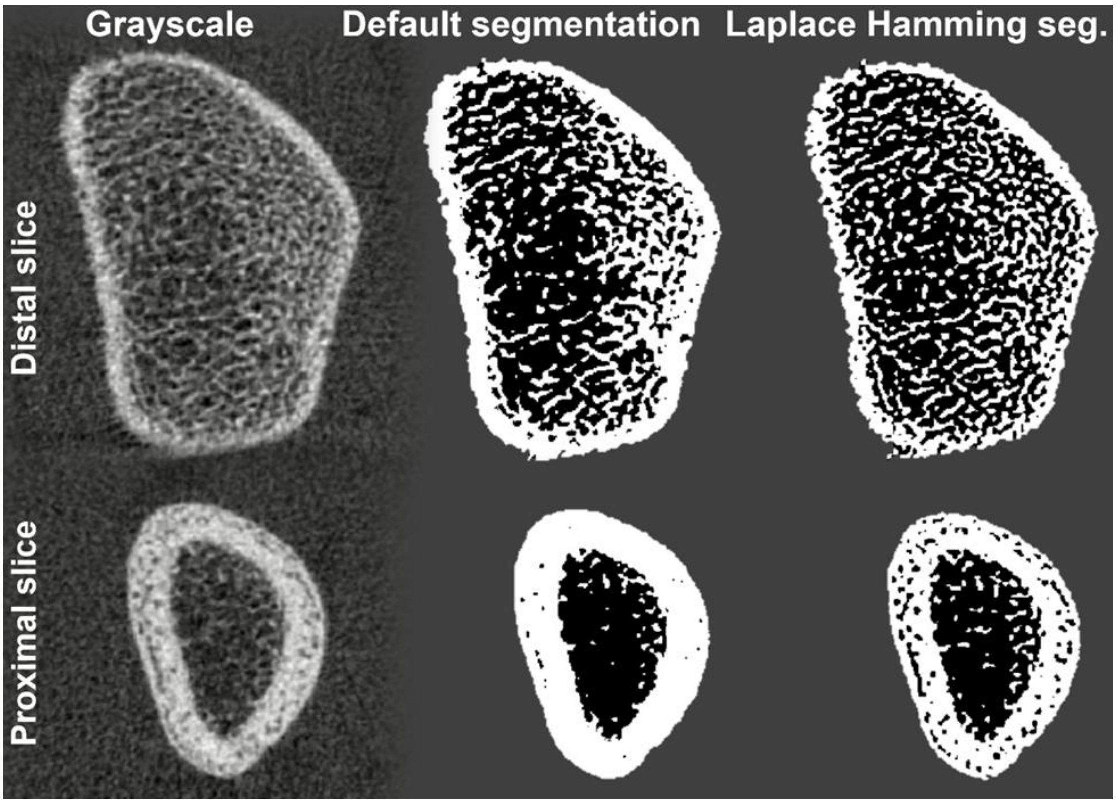

### Disclosure

This research was supported by Mereo Biopharma and Shriners Hospitals for Children. B.M.W. and F.R. have received institutional research support and materials and have consulted for Mereo BioPharma.

### OC5.6

### Validation of Automated Bone Age Assessment in X-Linked Hypophosphatemia Across Rickets Severity

Rodrigo Montero-Lopez[1,2], Rana Halloun[1,2], Damian Rauch[2], Maya Scharke[2], Lisa Dilworth[3], Joel Hetzer[3], Rui Santos[4,2], Tom Thacher[5], Kerry Siminoski[2], Stefan Jackowski[2], Nasrin Khan[2], Leanne M Ward[1,2]

[1]Department of Pediatrics, University of Ottawa, Division of Endocrinology, Children's Hospital of Eastern Ontario, Ottawa, Canada. [2]The Ottawa Pediatric Bone Health Research Group, Children's Hospital of Eastern Ontario Research Institute, Ottawa, Canada. [3]Ultragenyx Pharmaceutical Inc.,, Novato, CA, USA. [4]Evelina London Children's Hospital, Guy's and St Thomas' NHS Foundation Trust, London, UK. [5]Department of Family Medicine, Mayo Clinic, Rochester, MN, USA

### Abstract

**OBJECTIVE:** Bone age (BA) from a hand/wrist radiograph is widely used to characterize skeletal maturation. In X-linked hypophosphatemia (XLH), rickets-related abnormalities may alter ossification patterns and potentially compromise automated BA algorithms. BoneXpert™ has been validated in healthy children and growth disorders, but not in rachitic conditions such as XLH. We aimed to validate the accuracy of BoneXpert™ for BA assessment across rickets severities in XLH.

**METHODS:** Hand radiographs were obtained from the XLH Disease Monitoring Program (NCT03651505). Images were stratified by bilateral wrist Rickets Severity Score (RSS) into three groups: absent (0), low (0.5–1.5), and high (2–4), with 1:1 sex- and age-matching across groups. Two pediatric endocrinologists (expert readers), blinded to chronological age (CA), independently assigned BA using Greulich & Pyle Atlas references; discrepancies >1.5 years were resolved by consensus. Expert BA was the mean of the expert readers' scores (or consensus when required). Automated BA was generated using BoneXpert™ (v3.2.2). Agreement between expert BA and BoneXpert™ was evaluated using intraclass correlation coefficients (ICC) and Bland–Altman analysis. BoneXpert™ BA:CA delay was calculated as BoneXpert™ BA minus CA. Secondary analyses compared BoneXpert™ phalangeal- and carpal-based BA (paired t-test).

**RESULTS:** Ninety-nine hand radiographs were analyzed (CA mean±SD 5.78±3.0 years; 48% female) from 80 participants. Inter-reader concordance was excellent (ICC 0.975 [95% CI 0.963–0.983], p<0.0001). Expert BA showed excellent agreement with BoneXpert™ (ICC 0.984 [95% CI 0.976–0.989], p<0.0001; r=0.985 [95% CI 0.978–0.990]) with a small mean underestimation by BoneXpert™ versus expert BA (−0.17±0.52 years; p=0.002). Agreement remained excellent across RSS strata (ICC [95% CI]: high RSS 0.993 [0.986–0.997], low RSS 0.982 [0.963–0.991], RSS absent 0.919 [0.844–0.959]). Overall, the BA:CA delay was -0.57±0.91 years, with the greatest delays in the highest RSS group (-0.76±0.58 years). Within BoneXpert™, carpal BA was more delayed than phalangeal; estimates differed by a mean of 0.32±0.84 years (N=91; p<0.001).

**CONCLUSIONS:** In children with XLH, BoneXpert™ BA closely aligns with expert assessment and appears robust across the spectrum of rickets severity. These findings support BoneXpert™ as a practical, standardized tool for BA assessment in XLH clinical care and research.

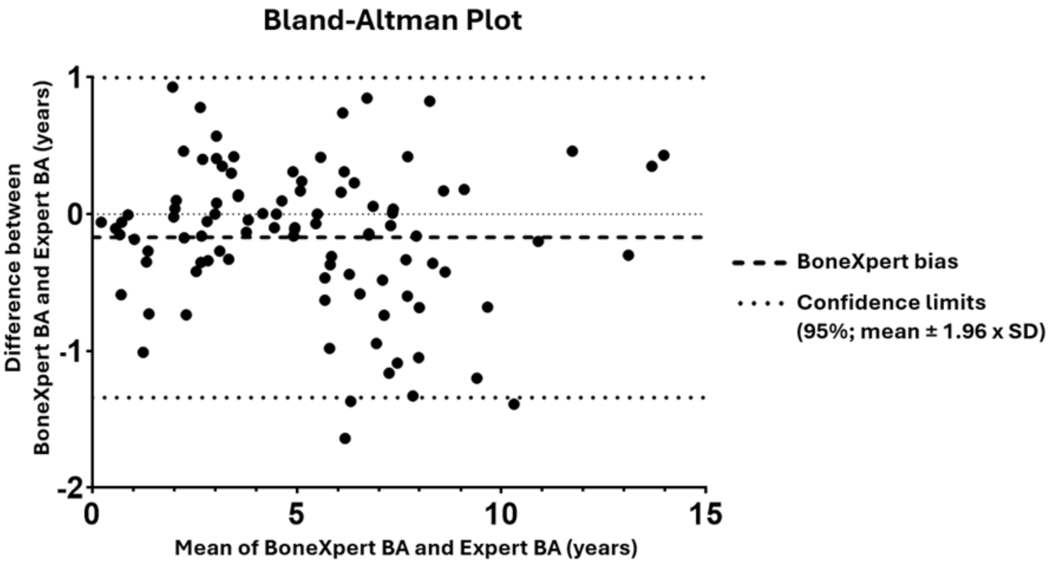

### Disclosure

LMW: Consultant to Ultragenyx and Kyowa Kirin, and participation in clinical trials with Ultragenyx (with funds to Dr. Ward's institution). LD, JH: Employee, Stockholder: Ultragenyx Pharmaceutical Inc. RS: Consultant to Kyowa Kirin and BridgeBio. TT: Consultant to Ultragenyx, Kyowa Kirin, and Acculine. No other disclosures

### P27

### From Panic to Preparedness: How Emergency Splint Kits Improve Fracture Care in Osteogenesis Imperfecta

Lori VandenBroek, Nadia Narducci, Argerie Tsimicalis

Shriners Hospital for Children - Canada, Montreal, Canada

### Abstract

**Background:**
Osteogenesis imperfecta (OI) is a genetic disorder characterized by increased bone fragility, low bone mass, and other connective tissue manifestations[1]. Individuals with severe OI may experience multiple fractures annually from birth through adulthood. These fractures range from minor microfractures to severe fractures necessitating surgical intervention. Proper fracture management is essential for pain control, optimal bone healing, reduction of complications, and restoration of function and mobility.

In 2019, Shriners Hospitals for Children–Canada (Shriners), in collaboration with one of our OI patients, introduced a fracture splint kit. This kit contains the tools, materials, and educational resources required to appropriately immobilize a fracture during an emergency[2]. Approximately 130 fracture kits have been distributed to OI patients at the Shriners, ensuring immediate access to supplies when needed. Families are educated upon receiving their fracture kits by the OI nursing care team.

**Objective:**
Since the implementation of the splint kits, we opted to evaluate the kits via a quality improvement initiative to understand the perceived usefulness, and impact on the autonomy and quality of life of the patients and their families.

**Methods:**
Over a four-month period patients with OI or their family members were invited to complete an anonymous questionnaire while attending a clinic visit at Shriners. Data were descriptively analyzed.

**Results:**
To date 20 patients with OI or their family members responded. Overall, they expressed a significant perceived usefulness of the kit, which has become an essential item for the immobilization of fractures. Patients and families' responses were overwhelmingly positive, highlighting increased confidence, reassurance, and improved fracture management at home. The families also reported a reduction in anxiety during fracture emergencies.

**Conclusion**

This initiative improved patient and family autonomy, confidence, and perceived quality of life while promoting safer early fracture management. The program shows strong potential for expansion to other osteogenesis imperfecta care settings.

Disclosure none declared

### P127

### Collaborative Practice-Based Creation of a Podcast Series on Virtual Reality for Pain and Anxiety in Children's Orthopaedic Healthcare

Jessica Ding[1], Alisha Michalovic[2], Emily K Drake[3], Argerie Tsimicalis[1,2]

[1]Shriners Hospitals for Children - Canada, Montreal, Canada. [2]McGill University, Montreal, Canada. [3]Mount Allison University, Sackville, Canada

### Abstract

**Objectives:**

Significant levels of pain and anxiety are experienced by children undergoing medical procedures, such as orthopaedic surgeries. Pain and anxiety may be lessened by distraction techniques such as virtual reality (VR). However, barriers have contributed to a significant lag in implementing VR into clinical practice. This practice-based study sought to collaboratively create a podcast series to promote knowledge dissemination and VR implementation into practice.

**Methods:**

A practice-based study, guided by the principles of andragogy and cognitive theory of multimedia learning (CTML), was conducted. Twelve experts in the field of VR participated in a one-time, audio-recorded interview and contributed to the creation of their respective episode, on topics related to VR evidence, use, integration, creation, troubleshooting and decision-making. Across the episodes, a thematic analysis was conducted to inform a summary episode to the podcast series. The process of creating the podcast series was documented.

**Results**:

Nine podcast episodes were created with 12 participants including a child life specialist, a clinical research coordinator, doctors, decision-makers, nurses and clinicians. Main themes identified included VR benefits and future use, clinician difficulties, immersion, interaction with users, non-universality of VR, adverse effects and practice implementation.

**Conclusions:**

Podcasts offer a novel way to disseminate knowledge but there has been limited understanding of their optimal structure. This paper delineates the systematic approach undertaken to produce a podcast series to disseminate science. The created podcast series will help disseminate knowledge on VR use in children's orthopaedic healthcare, consequently promoting its implementation into practice.

### Disclosure

None declared

### P128

### The Experience of Spinal Bracing at a UK-Based Paediatric Osteogenesis Imperfecta Centre

Emilie Hupin, Alice Walker, Zoe Bowman Bayles, Abigail Bolland, Mark Heathfield, Alex Chesover, Moira Cheung, Belinda Crowe, Catherine DeVile

Osteogenesis Imperfecta Service, Great Ormond Street Hospital for Children NHS Foundation Trust, London, UK

### Abstract

**BACKGROUND:**

Osteogenesis Imperfecta (OI) is a bone fragility disorder typically caused by a defect in the genes which produce type 1 collagen. Features include propensity to fracture and skeletal deformities. The development of scoliosis is common, with overall prevalence ranging from 39% - 80%. Evidence shows that in children, untreated spinal curves may progress, and timely intervention is key in preventing complications such as pain and reduced pulmonary function. Conservative, medical and surgical management options should be considered for best outcomes.

**PRESENTING PROBLEM:**

The use of spinal bracing for scoliosis management in OI appears to vary internationally. Previous literature suggests the risk of rib fractures and chest wall deformities is high, tolerance poor, and bracing is not effective in stabilising spinal curves.

Some of the research pre-dates bisphosphonate therapy and more recently spinal bracing is being considered across OI centres.

**CLINICAL MANAGEMENT:**

At a UK-based paediatric OI centre, spinal bracing is being trialled to support scoliosis management (N=15). Orthotic devices varied depending on clinical presentation, age, mobility, and the decision-making process involved the multidisciplinary team, orthotist and patient /family. Details regarding function, comfort and tolerance post-orthotic intervention were recorded. Interval radiology to evaluate progression of curves and spinal orthopaedic monitoring continued as normal.

Table 1.

**DISCUSSION:**

Our data suggest that using spinal bracing in OI is not without complications, with tolerance issues and discomfort frequently reported. In some cases, mobility was negatively impacted, and wear was interrupted by limb fractures or surgery. Orthosis modifications were often required. 6 patients achieved prolonged periods of wear (> 8 hours per day), and others continue to build up use gradually. Orthotic management was discontinued in 3 cases; one following skeletal maturity, one due to poor tolerance and one who proceeded to spinal surgery. To date, no serious adverse events such as fractures have occurred.

Further investigation is needed to determine the effect of bracing on spinal curve progression and chest wall shape over time. Bracing in paediatric OI requires careful MDT consideration and monitoring. We continue to explore spinal bracing as a safe and appropriate adjunct for scoliosis management.

Table 1

| *Sex* | *Age at initial bracing* | *OI type/ (Genetics)* | *Cobb angle* | *Orthotic device* | *Adverse effects* | *Wear > 8 hours* |
|---|---|---|---|---|---|---|

| | ***(yrs)*** | | | | | |
|---|---|---|---|---|---|---|
| M | 16 | Type 4 (*COL1A1*) | 24° | Rigid TLSO[1] | Discomfort, poor tolerance | No* |
| F | 12 | Type 1 (*COL1A1*) | 38° | Rigid TLSO | Discomfort, reduced balance | Yes (night) |
| M | 15 | Type 1 (*COL1A1*) | 27° | Rigid TLSO | Discomfort, self-conscious | No |
| F | 7 | Type 8 (*P3H1*) | 65° | Soft Boston brace | Negative impact on floor mobility | Yes |
| M | 12 | Type 3 (*COL1A1*) | 48° | Bivalve soft Boston brace | Overheating, poor fit in wheelchair | No |
| M | 10 | Type 3 (*COL1A2*) | 34° | Soft Boston brace | Discomfort, poor tolerance | No |
| F | 2 | Type 7 (*CRTAP*) | 57° | Semi-rigid TLSO | Negative impact on floor mobility | Yes |
| M | 15 | Type 3 (*COL1A1*) | 67° | Rigid TLSO | Nil reported | Yes (night) |
| M | 5 | Type 8 (P3H1) | 31° | DEFO[2] | Carer anxiety | No |
| F | 2 | Type 15 (*WNT1*) | 32° | DEFO | Unable to use during limb fractures | Yes |
| M | 10 | Type 7 (*CRTAP*) | 69° | Posterior Boston brace with abdominal binder | Reduced tolerance, skin redness | No |
| F | 8 | Type 4 (*COL1A2*) | 39° | Semi-rigid TLSO | Overheating | Yes |
| F | 8 | Type 11 Bruck (*FKBP10*) | 18° | Soft Boston brace | Reduced tolerance, poor fit in wheelchair | No |

| | | | | | | |
|---|---|---|---|---|---|---|
| F | 6 | Type 1 /HGPPS[3] (*COL1A1*/*ROBO3*) | 78° | Rigid TLSO | Poor tolerance – refusal to wear | No* |
| M | 9 | Type 8 (*P3H1*) | 45° | DEFO | Poor tolerance – risk of fracture when applying | No* |

*[1]TLSO – thoracolumbar support orthosis*

*[2]DEFO- dynamic elastomeric fabric orthosis (Lycra suit)*

*[3]Horizontal gaze palsy with progressive scoliosis*

**Bracing discontinued*

### Disclosure

None declared

### P133

### Investigating the Effects of Group Aquatic Therapy for Children with Osteogenesis Imperfecta

Mallory Gardner, Anthony Puglisi

Children's Hospital of Philadelphia, Philadelphia, USA

### Abstract

OBJECTIVES:

Aquatic physical therapy is routinely recommended for children with Osteogenesis Imperfecta (OI) as a safe modality for muscle strengthening, weight-bearing, and balance exercise. The buoyancy of the water protects from falls while the viscosity provides resistance for strengthening. Despite this recommendation, there is little evidence on the effects of aquatic physical therapy on muscle strength, muscle power, or function in children with OI. The objectives of this case series are to outline specific exercises appropriate for ambulatory children with OI and to review the effects on muscle strength, muscle power, and function after a six-week aquatic group therapy program.

METHODS:

The 50 minute weekly aquatic therapy group will focus on swimming, lower extremity strengthening activities, and balance exercise in floating, kneeling, and standing. Prior to the initiation of the program and within two weeks after the last session, leg strength will be assessed by handheld dynamometry, muscle power by broad jump distance and vertical jump height, and functional mobility with timed up and down stairs. The Water Orientation Test Alyn 2 will be used to assess function and swimming skills in the water. Baseline and post-intervention outcomes will be converted into and age and sex specific Z-scores using standard references and paired t-tests will be used to determine significance.

RESULTS:

To date, four patients with OI have agreed to participate in this pilot aquatics group (two females, two males, ages 8-11). Comparison of baseline and post 6-week intervention outcomes will be completed upon cessation of the program. Description of aquatic exercises will include walking activities, swim strokes, floatation exercises, balancing in varied positions, step ups, jumping, and lunging.

CONCLUSIONS:

We plan to supplement the evidence on aquatic physical therapy for ambulatory children with OI, by outlining specific exercises performed and the effects on muscle strength, muscle power, function, and swimming skills after six weeks in the group setting in the pool.

### Disclosure none declared

### P136

### Use of Standardised Motor Assessments to Evaluate Developmental Motor Skills in Children with Osteogenesis Imperfecta

Lisa Mills[1], Amy Pilkington[1], Hannah Bassett[1], Toby Candler[1,2]

[1]Bristol Royal Hospital for Children, Bristol, UK. [2]NIHR Biomedical Research Centre (Diet & Physical Activity Theme), University of Bristol, Bristol, UK

### Abstract

Developmental motor skills and praxis underpin performance of functional movement and participation in daily activities. In Osteogenesis Imperfecta (OI), skeletal fragility, hypermobility, pain, and reduced muscle strength may disrupt typical motor development. Clinical monitoring often focuses on motor milestones and mobility, but standardised motor assessments may offer a more detailed evaluation of functional abilities.

**Objectives**

- To evaluate the utility and compare outcomes of two widely used standardised motor assessments in children with mild–moderate OI.
- To explore the range of motor skill strengths and difficulties in this group

**Methods**

Twenty-three children with mild-moderate OI attending a UK specialist centre were invited to participate. Participants completed the Movement Assessment Battery for Children–3 (MABC3) and the Bruinincks–Oseretsky Test of Motor Proficiency–2 (BOT2) in two separate appointments. Pain and fatigue (0–10) were rated pre and post assessment. Children also rated task difficulty and enjoyment (1–6). Administration and scoring times were recorded. Outcomes were analysed using Bland–Altman plot, Spearman's Rank correlation, and Mann-Whitney U calculations.

**Results**

Twenty children completed both assessments; 12F:8M; median age ten years(range=6-14). Median total previous fractures six (range=0-14); six were receiving bisphosphonates and three had prior rodding surgery. Mean composite motor scores were within the average range (BOT2=41.56; MABC3=44.5), with good agreement and correlation between tests (R=0.734). Participants showed weaker performance across several subtests; fine motor precision, co-ordination, strength, and gross motor agility tasks. Mean differences between pre and post-assessment ratings of pain (BOT2=0.8; MABC3=0.6, p=0.182) and fatigue (BOT2=0.5; MABC3=0.85, p=0.399) ratings were low and similar across assessments. Mean enjoyment ratings were comparable (BOT2=1.65/6; MABC3=1.95/6, p=0.342). Scoring time was identical (38 minutes). MABC3 was rated easier (BOT2=2.85/6; MABC3=2.35/6, p=0.016). BOT2 administration was longer (56 vs 45 minutes, p=<0.001). but assessed a broader range and depth of skills.

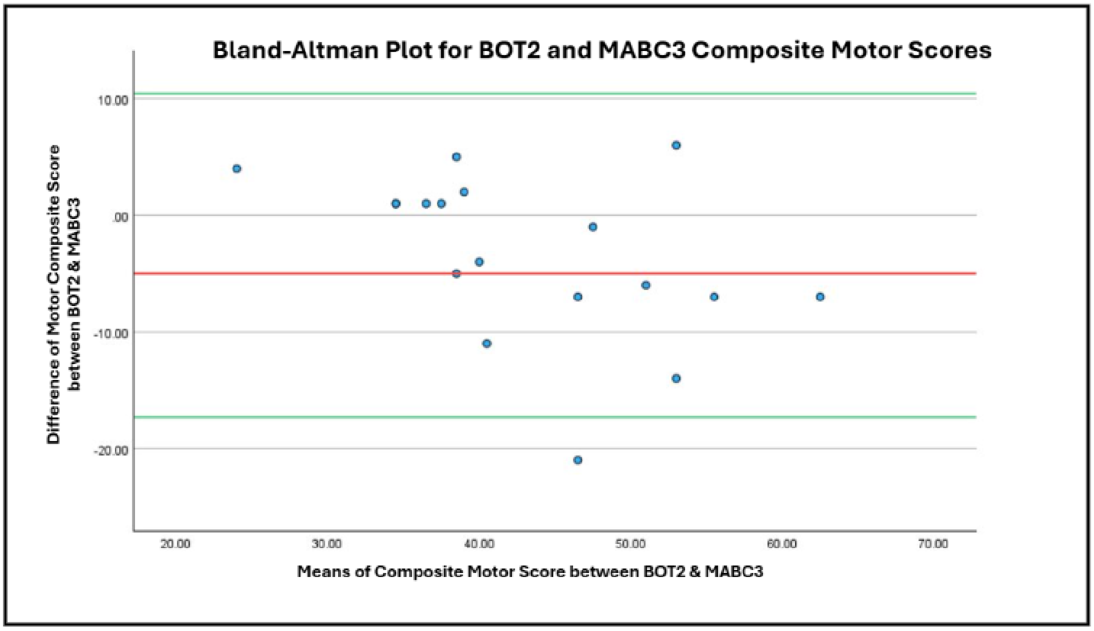

**Conclusions**

Standardised motor assessments are valuable in identifying specific motor difficulties, informing targeted therapy when administered by clinicians experienced with children with OI. Both assessments were comparable across a range of outcome and experience measures. Further research is warranted to evaluate applicability for children with more severe OI phenotype.

### Disclosure

None declared

### P137

### Navigating the Move to Adult Services: Consultation Insights from Skeletal Dysplasia Youth and Their Parents

Anita Freeman, Catherine Ali, Marianna Shiafkou, Emma Bewick, Emilie Hupin, Daniela Melamed, Emily Golding, Rachael Worthen, Moira Cheung

Great Ormond Street Hospital NHS Trust, London, UK

### Abstract

**Objectives:**

Young people with skeletal dysplasia (SKD) face complex physical, psychological, and social challenges as they transition from paediatric to adult healthcare. Despite established transition frameworks, limited research has explored condition-specific needs and priorities for this population.

**Methods:**

A service development consultation was conducted at Great Ormond Street Hospital for Children between July and November 2025. Parallel focus groups with young people (aged 15–20) and parents explored experiences of gaining independence, priorities for transition preparation, and perspectives on multidisciplinary care. Transcriptions and facilitator notes underwent thematic analysis, followed by multidisciplinary team (MDT) review and parent feedback.

**Results:**

Nine young people expressed an interested in joining the consultation, and a total of 4 young people and 5 parents were able to join the focus groups (Figure 1). The thematic analysis from the focus groups highlighted systemic, psychological and practical barriers to independence, including navigating visible difference, fluctuating support needs in education, challenges using public transport, and difficulties advocating in healthcare settings. Parents emphasised concerns about readiness for medical decision-making, inconsistent coordination across services, and the need for structured preparation. Young people prioritised understanding their condition, developing confidence in appointments, and accessing meaningful peer support. Across groups, early preparation, clear information-sharing, and holistic, well-coordinated care were identified as essential.

***Figure 1.*** Participant Demographics

| Sex | Age | Diagnosis | Young Person Group | Parent group |
|---|---|---|---|---|
| Female | 20 | Hajdu-Cheney Syndrome | Yes | Yes |
| Male | 17 | Pseudoachondroplasia | Yes | Yes |
| Female | 15 | Achondroplasia | Yes | Yes |
| Female | 16 | Chondrodysplasia | Yes | Yes |
| Male | 17 | Achondroplasia | No | Yes |

**Conclusions:**

Findings informed a set of targeted service developments, including independence-focused outpatient practices with pre-clinic preparation calls with the young person, annual peer workshops, joint OT–Physiotherapy sessions with patient led goal-based to enable independence (e.g. use of public transport), enhanced psychological support, and strengthened transfer-of-care processes. The consultation underscores the need for a developmentally attuned MDT support, with a SKD-informed, and family-inclusive transition pathway that promotes autonomy while recognising the unique social and emotional contexts faced by young people with SKD.

### Disclosure

None Declared

### P163

### Perceived Barriers to Physio- and Occupational Therapy Needs in Children with Bone Disorders: A Survey-Based Study as Part of the Navigating the Inter-disciplinary Care Experience (NICE) Bone Health Research Program

Stefan Jackowski[1], Jessica Buffam Buffam[1], Serdar Kilicarslan[1,2], Ayushi Regmi[1], Jinhui Ma[3], Mei Wang[1], Nasrin Khan[1], Fahd Alshammri[1,4], Sarah Bakhamis[5], Ulrich Montcho[1,4], Rana Halloun[1,4], Leanne Ward[1,4]

[1]Children's Hospital of Eastern Ontario, Ottawa, Canada. [2]Division of Neurology, Department of Pediatrics, University of Ottawa, Ottawa, Canada. [3]McMaster University, Hamilton, Canada. [4]Division of Endocrinology, Department of Pediatrics, University of Ottawa, Ottawa, Canada. [5]Section of Pediatric Endocrinology, Department of Pediatrics, King Faisal Specialist Hospital & Research Centre, Riyadh, Saudi Arabia

### Abstract

***Objective:*** Pediatric patients with rare bone diseases face bone fragility, chronic pain, and reduced mobility. Physiotherapy (PT), occupational therapy (OT), and mobility devices are often recommended, yet access may be limited by barriers. This study examines the barriers perceived by caregivers of patients with rare bone diseases in accessing rehabilitation services.

***Methods***: A prospective, cross-sectional observational survey study was conducted involving 105 children <19 years of age receiving care in a tertiary care pediatric rare bone disease clinic. Data were collected through patient and caregiver questionnaires and chart reviews. The questionnaires were designed to explore perceived barriers to accessing and participating in rehabilitation services (e.g. logistical, financial, and personal factors). Descriptive analyses summarized participant characteristics and reported barriers.

***Results:*** Overall, 49.5% of caregivers and patients (52/105) reported at least one barrier to accessing PT, OT, or mobility devices as part of rehabilitative care. Among those reporting at least one barrier, 15.4% (8/52) reported feeling overwhelmed when choosing a rehabilitation clinic, and 43.3% (17/52) indicated a need for more information about available rehabilitation services in their community. Among the 62.9% of patients (66/105) requiring PT, 18.2% (12/66) were unable to access services, with 71.2% (47/66) reporting at least one barrier. The most frequently reported barrier was limited appointment availability outside of work or school hours (29/66, 43.9%). OT was required by 27.6% of patients (29/105), of whom 72.4% (21/29) experienced at least one barrier. As for PT, limited appointment availability outside of work or school hours was the most common barrier to OT (11/29, 37.9%). Among patients requiring mobility devices (38.1%, 40/105), 60.0% (24/40) reported barriers, mostly related to family financial constraints (15/40, 37.5%).

***Conclusions:*** Patients with rare bone disorders and their caregivers experienced substantial barriers to accessing rehabilitation services across PT, OT, and mobility devices. Common barriers included limited appointment availability outside of work or school hours and financial constraints. Many families also reported feeling overwhelmed when selecting providers and needing more information. Flexible scheduling, financial support, and patient navigation services may help reduce these barriers and improve access to rehabilitative care.

### Disclosure

None declared

### P183

### Patient Reported Pain in Children with Osteogenesis Imperfecta

Kieran Murphy, Claire Hill, Chidambaram Sethuraman

Sheffield Childrens Hospital foundation trust, Sheffield, UK

### Abstract

**OBJECTIVES**

To understand the prevalence of pain in children with Osteogenesis Imperfecta (OI) under the care of the metabolic bone disease team at Sheffield Children's Hospital.

To gain greater understanding of patient reported pain in paediatric OI population

To understand if children and families are interested in pain education

**METHODS**

Retrospective analysis of the pain domain (Q15-19) on 362 completed OI Quality of Life (OIQOL) questionnaires completed by patients under the care of the metabolic bone disease team between September 2016 and October 2024.

A scoping pain questionnaire was handed out to patients with OI and their families during their clinic appointments or admissions for bisphosphonates between January and March 2025.

**RESULTS**

Analysis of the OIQOL identified 6.9% of patients reporting a high frequency of pain with a further 17.1% reporting moderate frequency of pain.

The self-reported pain questionnaire was completed by 23 patients age between 8-17

26% reported daily pain, 9% weekly pain. 43% monthly pain with the remaining 22% never reporting pain. There was no correlation between OI type and reported incidence of pain. Back 32% and legs 28% were the most common areas for pain.

8 participants (35%) wanted pain education with 2 patients (9%) being unsure and 13 (56%) were not interested in pain education. There was a strong correlation between participants who reporting daily or weekly pain and wanting pain education of 62.5%.

**CONCLUSIONS:**

The service evaluation highlighted the reported prevalence of pain on children under the metabolic bone disease team at Sheffield Children's Hospital with 24% of patients who completed the OI QOL reporting pain having a significant impact on quality of life. 35% of patients who completed the scoping pain questionnaire reported daily or weekly pain with 35% of completed questionnaires wanting pain education.

As a service we aim to offer a larger program of support on pain management and pain education to all patients with a diagnosis of OI for them to opt in to.

### Disclosure

Dr Claire Hill developed OI quality of life questionnaire

### Poster Abstracts

### P1

Abstract Withdrawn

### P2

### Longitudinal Changes in Serum PRO-C1 and PRO-C2 Reflect the Pubertal Growth Spurt in Healthy Children and Adolescents

Li Juel Mortensen[1,2], Yi He[3], Martin Blomberg-Jensen[4,5], Jørgen Holm Petersen[6], Stine Agergaard Holmboe[7,8], Anne-Christine Bay-Jensen[3], Anders Juul[7,9,5]

[1]Department of Growth and Reproduction, Copenhagen University Hospital-Rigshospitalet, Copenhagen, Denmark. [2]: International Centre for Research and Research Training in Endocrine Disruption of Male Reproduction and Child Health (EDMaRC), Copenhagen University Hospital - Rigshospitalet, Copenhagen, Denmark. [3]Nordic Bioscience, Department of Rheumatology, Nordic Bioscience, Biomarkers and Re-search, Herlev, Denmark. [4]Department of Endocrinology and Internal Medicine, Division of Translational Endocrinology, Copenhagen University Hospital-Herlev and Gentofte, Herlev, Denmark. [5]Department of Clinical Medicine, University of Copenhagen, Copenhagen, Denmark. [6]Section of Biostatistics, Institute of Public Health, Faculty of Health Sciences, University of Copenhagen, Copenhagen, Denmark. [7]Department of Growth and Reproduction, Copenhagen University Hospital-Rigshospitalet, Copenhagen, Copenhagen, Denmark. [8]International Centre for Research and Research Training in Endocrine Disruption of Male Reproduction and Child Health (EDMaRC), Copenhagen University Hospital - Rigshospitalet, Copenhagen, Denmark. [9]International Centre for Research and Research Training in Endocrine Disruption of Male Reproduction and Child Health (EDMaRC), Copenhagen University Hospital - Rigshospitalet, Copenhagen, Copenhagen, Denmark

### Abstract

**Introduction** Epiphyseal growth in children and adolescents is stimulated directly by growth hormone (GH) and indirectly via insulin-like growth factor 1 (IGF-I). Monitoring of growth in short children treated with GH is commonly done by serum IGF-I, which is not an ideal biomarker. Thus, other biomarkers reflecting endogenous and GH-induced growth are needed. Growth plate activity and collagen synthesis are reflected by increasing procollagens like PRO-C1 and PRO-C2. Therefore, we evaluated PRO-C1 and PRO-C2, from the longitudinal part of the Copenhagen Puberty Study, established reference intervals, and evaluated the association to linear growth.

**Methods**. We included 1700 serum samples from 108 females and 105 males (age 5-16 years), followed longitudinally between 2006-2014. We assessed height velocity, including peak height velocity (PHV), pubertal development by Tanner-staging, and serum concentrations of sex-steroids, IGF-I, and PRO-C1, and PRO-C2 by specific immunoassays. In addition, PRO-C1 and PRO-C2 were determined in short children born small for gestational age (SGA) before and after 12 months of high-dose GH treatment.

**Results** PRO-C1 and PRO-C2 increased significantly with increasing age and pubertal development (females: Tanner B1 through B3, $p < 0.0001$; males: G1 through G4, $p < 0.001$). Both procollanges increased to maximal levels at the time of PHV, with a subsequent rapid decline. By contrast, sex-steroids and IGF-I continued to increase after cessation of linear growth. IGF-I standard deviation score (SDS) was correlated with both PRO-C1 SDS (females $r^2 = 0.40$; males $r^2 = 0.34$, both $p < 0.0001$); and with PRO-C2 SDS (females $r^2 = 0.24$; males $r^2 = 0.32$, both $p < 0.0001$). In GH-treated prepubertal children, both markers increased significantly compared to baseline (PRO-C1: mean 0.70 SD vs. 3.78 SD, $p=1.33 \times 10^{-5}$; PRO-C2 SDS: mean -2.07 SD vs. mean 0.69 SD, $p=0.0055$).

**In conclusion,** we present longitudinal changes in serum levels of PRO-C1 and PRO-C2, as potential biomarkers of endogenous and GH-induced linear growth. Further studies are needed to evaluate the clinical usefulness of procollagens as biomarkers of GH-induced growth.

### Disclosure

PRO-C1 and PRO-C2 are measured using commercially avalable kits for Nordic Bioscience. ACBJ is an employee and shareholder of Nordic Bioscience. YH is an employee of Nordic Bioscience.

### P3

### Exploring the Relationship Between Disease Duration, Glycemic Control, and HR-pQCT-derived Bone Parameters in Adolescents with Type-1 Diabetes

Jada Kiss, Danielle Whittier, Steven Boyd, Richard Walker, Josephine Ho, Leigh Gabel

University of Calgary, Calgary, Canada

### Abstract

**Background:** Type-1 diabetes (T1D) is the most common childhood endocrine disorder. Sub-optimal bone accrual during adolescence increases the risk of fractures and osteoporosis, and individuals with T1D are at elevated risk, although the mechanism is unclear. T1D duration and poor glycemic control may impair bone density and microarchitecture; however, evidence remains conflicting.

**Objectives:** To examine the relationship between disease duration, glycemic control (HbA1c, percent of time spent within target blood sugar range; TIR), and bone outcomes from high-resolution peripheral quantitative computed tomography (HR-pQCT) in 10 to 12-year-old adolescents with T1D.

**Methods:** Adolescents (age 10-12) with T1D were recruited into the Calgary arm of the Canadian Bone Strength Development Study. Participants reported demographics, T1D duration, and TIR from their continuous glucose monitors over the past 90 days. HbA1c was obtained from medical records from the four most recent measurements. HR-pQCT (Xtreme CT II, 61[illegible]m resolution) scans were performed at the 4% radius, 30% radius, 6% tibia, and 30% tibia to examine cortical (distal/proximal sites) and trabecular (distal sites) volumetric bone mineral density (vBMD) and microarchitecture. Descriptive medians (interquartile range) were used, and Spearman's correlations examined the relationships between disease duration, TIR, and HbA1c with vBMD and microarchitecture.

**Results:** Seven females and three males (11.8 years (11.6-12.1) have been recruited to date. Median T1D duration was 5.2 (3.1-5.8) years, HbA1c was 7.7 (7.4-8.8), and 90-day average TIR was 57.0% (28.3-63.3%). Spearman's correlations revealed a significant positive correlation between T1D duration and cortical vBMD at the 30% tibia ($\rho=0.70$, $p=0.031$), though correlations between microarchitecture at this site were weak and non-significant (thickness: $\rho=0.26$; porosity: $\rho=-0.27$). There were no relationships detected between T1D duration, TIR, or HbA1c and cortical or trabecular vBMD or microarchitecture at any other scan site.

**Conclusions:** The lack of association between glycemic control and bone outcomes may be reassuring for individuals who are still optimizing their T1D management, though low power limits these findings. The positive association between T1D duration and proximal tibia cortical vBMD suggests T1D onset closer to rapid bone accrual periods may influence cortical mineralization. Future analyses from this ongoing study will confirm observations in a larger sample.

(350/350)

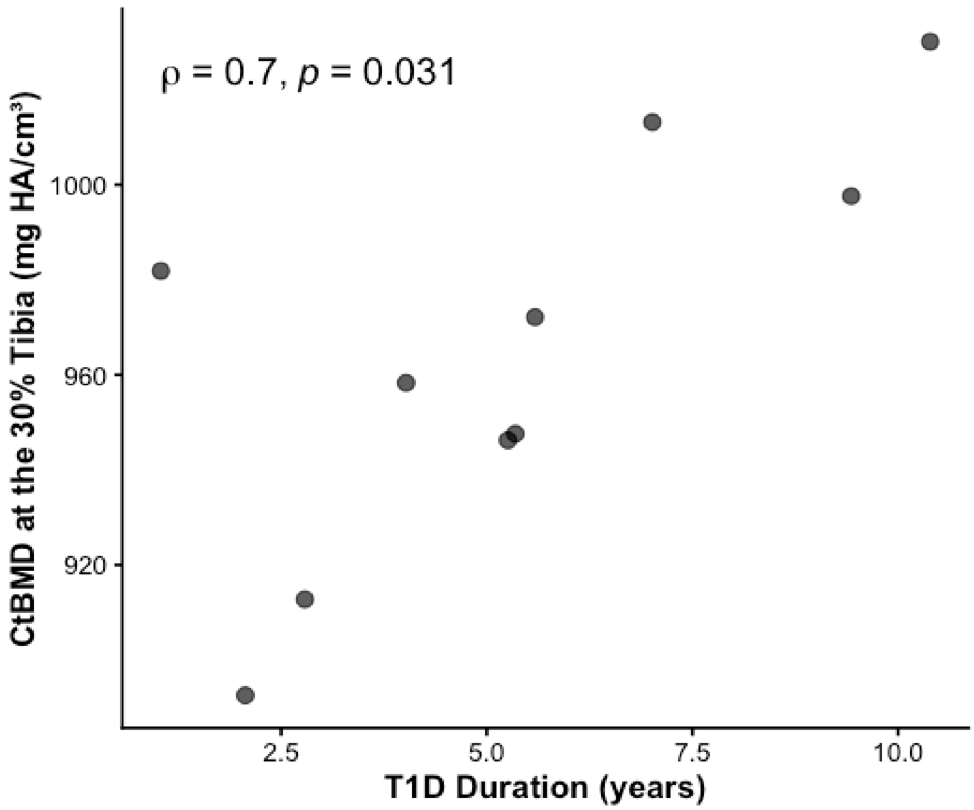

**Figure 1:** Scatterplot demonstrating the relationship between T1D disease duration and cortical vBMD at the 30% tibia.

**Disclosure**

None declared.

### P4

### Agreement Between Predicted and Longitudinally Observed Maturity Offsets in Children and Youth with Type 1 Diabetes

Zahra Ghafouri[1], James (J D) Johnston[2], Munier Nour[3], Saija Kontulainen[1]

[1]College of Kinesiology, University of Saskatchewan, Saskatoon, Canada. [2]Department of Mechanical Engineering, College of Engineering, University of Saskatchewan, Saskatoon, Canada. [3]College of Medicine, University of Saskatchewan, Saskatoon, Canada

### Abstract

### Objectives

Accurate assessment of biological maturation is fundamental in pediatric research and clinical care, particularly in chronic conditions that alter typical growth trajectories, such as Type 1 diabetes (T1D). Maturation influences physiological responses, metabolic control, and the effectiveness of intervention outcomes, including physical activity trials. However, clinical methods for assessing maturation are often viewed as sensitive, invasive or impractical in research or clinical settings. Sex-specific maturity offset predictions offer a practical, non-invasive approach for estimating somatic maturity using routinely collected clinical measures, yet their accuracy has not been evaluated in youth with T1D.

### Methods

Age and standing height data were extracted from the clinical records of 31 youth with T1D (17 males), aged 5–18 years, resulting in 283 longitudinal observations. Predicted maturity offset (MO) values derived from sex-specific Moore equations were compared with growth-based maturity offsets calculated from longitudinal height velocity curves generated using cubic spline models. Analyses were restricted to observations within ±4 years of age at peak height velocity (APHV). Agreement was evaluated using linear regression, Bland-Altman analyses, and subgroup comparisons across discrete maturity stages relative to APHV.

### Results

Predicted maturity offsets showed strong associations with growth-based estimates in females ($R^2 = 0.79$) and males ($R^2 = 0.82$) (Figure 1). In males, a proportional bias was evident, with systematic underestimation of maturity offset by the Moore equation (mean difference: -0.42 years; 95% *CI:* -0.57 to -0.27), particularly at higher height-for-age Z-scores. Subgroup analyses demonstrated closer agreement near APHV, with larger discrepancies (>0.5 years) observed further from APHV in both sexes, especially among males.

### Conclusions

Sex-specific Moore maturity offset predictions provide a practical approximation of maturational timing in youth with T1D using routinely collected clinical data. However, systematic underestimation observed in males may lead to misclassification of maturational status, with important implications for the interpretation of growth patterns, physical activity responses and other health-related outcomes. When precise maturity assessment is required in pediatric research, longitudinal, growth-based methods remain preferable.

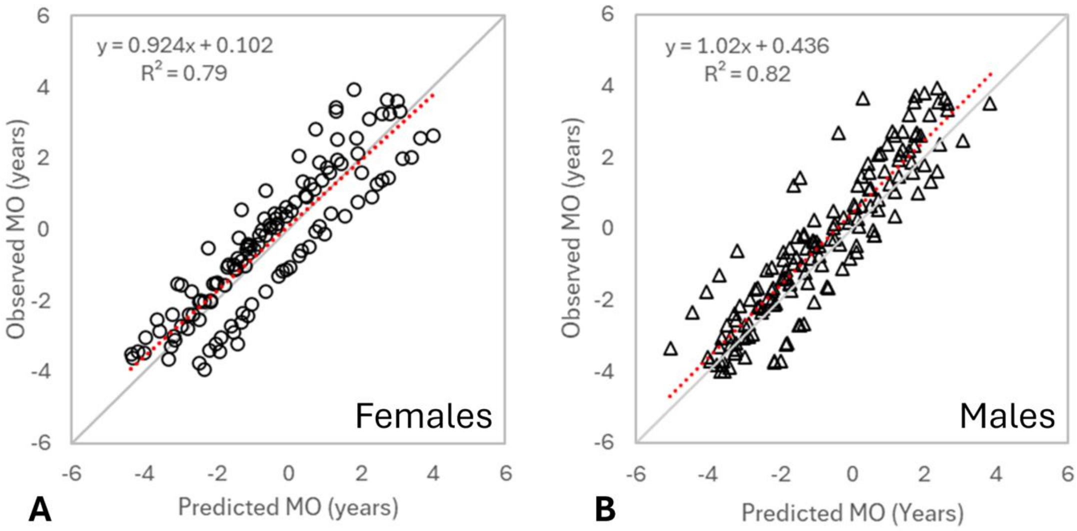

**Figure 1.** Scatter-plot, with linear regression fits (red dotted line) between predicted maturity offset (MO) and observed MO in female (A) and male (B) children and adolescents with T1D. Grey line pertains to the case where y = x.

### Disclosure

None declared.

### P5

### Trabecular Bone Score By Tanner Stage in Children from Mexico City: TBS Osteo Advanced for Pediatrics (Version 4)

Miguel Angel Guagnelli[1], Desiree Lopez-González[1], Anna Walstra[2], Madeleine Davies[2], Karen Hind[2,3], Didier Hans[2,4], Patricia Clark[1]

[1]Clinical Epidemiology Unit, Hospital Infantil de México Federico Gomez - Facultad de Medicina, Universidad Nacional Autónoma de México, Mexico City, Mexico. [2]Medimaps Group SA, Geneva, Switzerland. [3]Faculty of Health and Medicine, Lancaster University, Lancaster, UK. [4]Center of Bone Diseases, Rheumatology Unit, Bone and Joint Department, Lausanne University Hospital & University of Lausanne, Lausanne, Switzerland

### Abstract

OBJECTIVES*:*

Trabecular bone score (TBS), obtained through dual-energy X-ray absorptiometry (DXA), offers an indirect measure of trabecular bone microarchitecture that may provide additional information to bone mineral density during growth. Despite the close relationship between skeletal maturation and puberty, there remains limited data on expected pediatric TBS, related to specific pubertal stages. This study sought to establish sex-specific TBS reference curves for a Mexican pediatric population, and to examine how pubertal development, defined by Tanner stage, affects TBS values in males and females.

METHODS*:*

This cross-sectional analysis of DXA scans of the lumbar spine (GE Lunar iDXA) from 1,533 healthy participants used TBS Osteo Advanced (version 4), which adjusts for directly-measured abdominal soft-tissue thickness. TBS values were stratified by sex and Tanner stage (Sexual Maturity Rating 1–5). TBS was analyzed according to Tanner stage, and age-specific percentile curves were constructed using Generalized Additive Models for Location, Scale, and Shape (GAMLSS).

RESULTS*:*

Data from 781 females and 752 males (aged 5-18 years) were analysed. In girls, TBS accrual started from age 9, whereas in boys this was ages 10 to 11. Both sexes demonstrated significantly increased TBS throughout pubertal maturation, with the most pronounced accrual occurring between Tanner stages 2 and 3 (13.2% in females and 9.9% in males).
Females demonstrated larger TBS increases at this stage, from a median of 1.05 at Stage 2, to 1.21 at Stage 3, whereas males showed a more gradual increase from Tanner stage 2 (median TBS, 1.09) to 5 (median TBS, 1.28). Sex- and Tanner stage–specific TBS percentile curves, along with age-referenced curves, were established.

CONCLUSIONS:

In Mexican children and adolescents, trabecular bone score exhibits distinct, sex-dependent pubertal patterns, with substantial increases during mid-pubertal stages. These findings underscore the importance of incorporating pubertal stage alongside chronological age, when interpreting TBS in pediatric populations. The provision of Tanner stage–specific reference data supports the use of TBS as a complementary tool for assessing bone microarchitecture during growth and skeletal maturation.

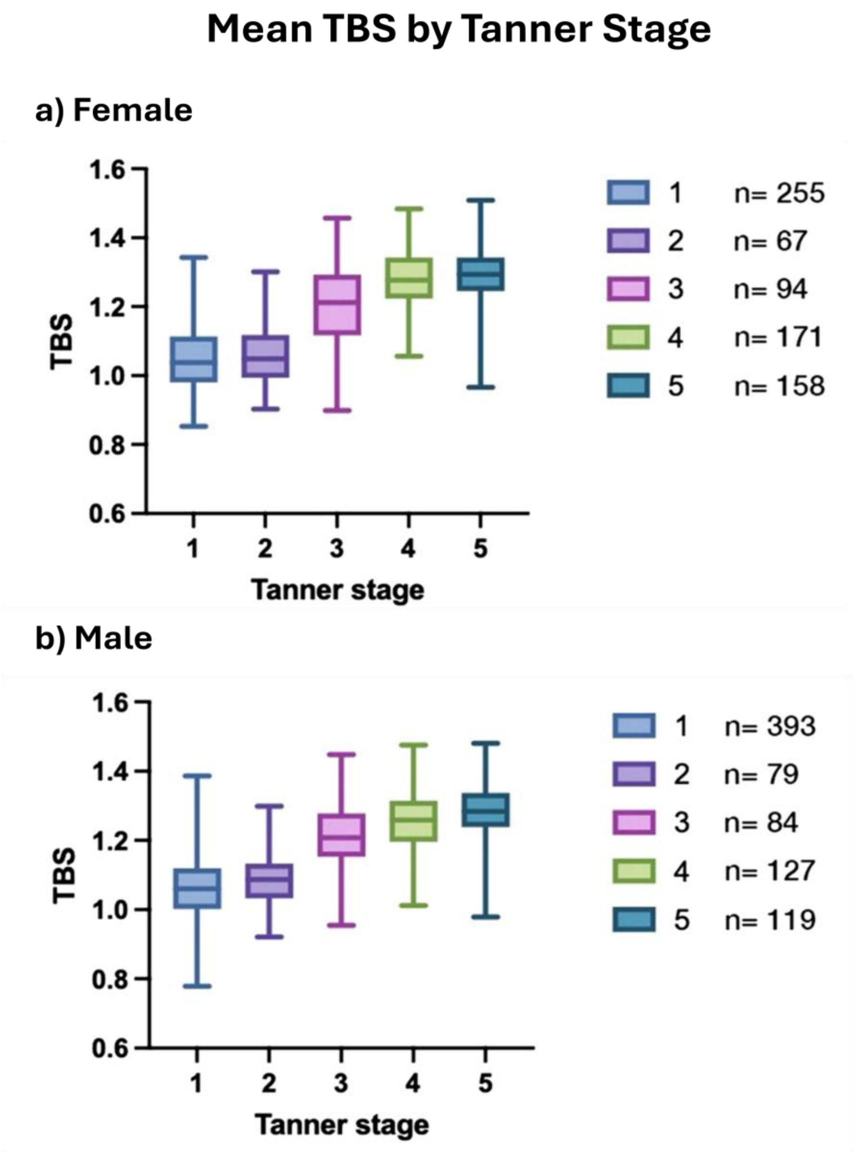

***Figure 1*** *Boxplot representing TBS distributions by Tanner stage for females (a) and males (b).*

### Disclosure

MG, DLG and PC declare they have no conflicts of interest. AW, MD and KH are employees of Medimaps Group SA, developers of TBS iNsight™ software. DH is co-owner of the TBS patent, has corresponding shares, and is part-time CEO at Medimaps Group.

### P6

### Sex-specific Differences in Bone Morphology and Response to Bisphosphonate Treatment Indicate Osteogenesis Imperfecta may not be Simply Labeled as a Low Bone Mass Condition

Chloe Derocher[1], Matthew Bernhard[2], Erin Carter[1], Karl Jepsen[3], Cathleen Raggio[1]

[1]Kathryn O. and Alan C. Greenberg Center for Skeletal Dysplasias, Hospital for Special Surgery, New York, USA. [2]College of Osteopathic Medicine, New York Institute of Technology, New York, USA. [3]Department of Orthopedic Surgery, University of Michigan, Ann Arbor, USA

### Abstract

#### Objectives:

Osteogenesis imperfecta (OI) is often classified as a low bone mass disorder, yet fracture risk may stem from structural abnormalities rather than reduced bone mass alone. This study aimed to define the structural basis of altered bone mass in OI using second metacarpal morphology and to evaluate differences in bone robustness and relative cortical area (RCA) by age, sex, OI type, and bisphosphonate treatment.

#### Methods:

This IRB-approved retrospective study analyzed anterior-posterior hand radiographs from individuals with OI and age-matched controls. Second metacarpal length and cross-sectional geometry were used to calculate robustness (total area/bone length) and RCA (cortical area/total area). The study included 276 radiographs from individuals with OI and 701 control radiographs. Multiple linear regression models assessed effects of age, sex, OI diagnosis, OI type, and bisphosphonate treatment within two years of imaging. Nonparametric tests compared morphologic parameters across common OI types.

#### Results:

Across the lifespan, individuals with OI had significantly lower metacarpal robustness compared to controls, indicating a slender bone phenotype ($R = -0.071$, $p < 0.05$). Robustness was positively associated with age and male sex. In contrast, OI diagnosis showed minimal correlation with RCA ($R = -0.013$), suggesting cortical bone mass relative to bone size is largely preserved. Within the OI cohort, bisphosphonate treatment was not associated with increased robustness or RCA. OI type was not a significant predictor of robustness, although type 3 OI tended toward lower robustness, while types 3 and 4 exhibited higher RCA than type 1. Sexual dimorphism was observed for robustness but not RCA.

#### Conclusions:

OI should be considered a slender bone phenotype rather than uniformly low bone mass. The weak association between OI and RCA challenges its classification as a low bone mass disorder and may explain the limited structural benefits of bisphosphonate therapy. These findings support a treatment paradigm guided by bone structural phenotype rather than bone mass alone and underscore the need for therapies that enhance periosteal expansion and structural strength in OI.

### Disclosure

None declared

### P7

### Infant Rib Material Properties Assessed Using Monotonic Loading to Failure, Microindentation and Raman Spectroscopy

Koushikreddy Allam[1], D Nicole Meyers[1], Miriam Soto Martinez[2], Jason Wiersema[2], Christian Crowder[3], Catherine Ambrose[1]

[1]University of Texas Health Science Center at Houston, Houston, USA. [2]Harris County Institute of Forensic Sciences, Houston, USA. [3]Tarrant County Medical Examiner's Office, Ft. Worth, USA

### Abstract

**Objectives**:

There is little data in the literature on the material properties of infant bone as bone specimens from this population are difficult to obtain and mechanical testing requires bone biopsies to be tested destructively. Here we present data from excised rib sections obtained from 50 decedents under the age of 1 year with no known bone disorders using 3 point bending (3PB) to failure, Raman spectroscopic imaging, and microindentation.

**Methods:**

All specimens were retrieved as antero-lateral cross sections from one rib between levels 2 and 8, wrapped in saline-soaked gauze, and frozen until imaging and testing. Each rib was imaged using microCT to obtain bone cross-sectional area (CSA), cross-sectional moments of inertia (second moments of area) about the major and minor axes (Imin and Imax), and the diameter in the testing direction. The specimens were tested in 3PB in the pleural to cutaneous direction at a displacement rate of 0.1mm/s until failure followed by Raman spectroscopy to evaluate bone composition (inVia confocal microscope, Renishaw) using a continuous 785 nm laser beam and a spectral range of 300 – 1800 $cm^{-1}$. The spectra were normalized to the v1 phosphate peak near 959 $cm^{-1}$ and averaged for each sample before important spectral peaks were isolated and peak ratios were calculated. Average microindentation results were obtained from at least 10 cyclical tests performed on the cutaneous surface of each specimen at locations near the fracture line.

**Results**:

Although the age range in this study is small (12 months), significant increases in parameters associated with cross-sectional geometry, mechanical strength, and bone mineralization increased with age while measures associated with bone material ductility decreased with increasing age (Table1). All of the microindentation measures correlated significantly with decedent age and many 3-point bending measures, indicating that this minimally-invasive technique for measuring infant bone material properties might be useful for predicting measures that are usually only obtained from destructive testing of biopsied bone samples.

**Conclusion:**

Our data provide valuable additional information about the normal bone material properties in infants and may indicate a method for obtaining material properties from infants with known bone diseases.

| Selected Correlations Between 3-point Bending and Raman Spectroscopy | | | | | | |
|---|---|---|---|---|---|---|
| | AmideIII/AmideI | Carbonate/Phosphatev4 | Proline/AmideIII | Proline/Ch2 | Phosphatev1/Proline | Hydroxyproline/Proline |
| ***Subject Age (months)*** | -0.075 | **0.416**** | **0.305*** | 0.060 | 0.174 | -0.228 |
| ***Ultimate Load*** | -0.131 | **0.386**** | 0.225 | 0.029 | 0.137 | -0.177 |
| ***Stiffness*** | -0.112 | **0.465**** | 0.274 | 0.106 | 0.115 | -0.221 |
| ***Elastic Energy*** | -0.254 | 0.215 | 0.237 | -0.042 | 0.161 | **-0.350*** |
| ***Plastic Energy*** | 0.036 | **0.316*** | 0.104 | 0.082 | 0.040 | -0.196 |
| ***Elastic Strain*** | **-0.418**** | 0.058 | 0.049 | **-0.292*** | 0.053 | -0.276 |
| | | | | | | |
| **Selected Correlations Between 3-point Bending and Microindentation** | | | | | | |
| | TID (µm) | IDI (µm) | aveCID (µm) | aveED (µJ) | aveUS (N/µm) | aveLS (N/µm) |
| ***Subject Age (months)*** | ***-0.661**** | ***-0.614**** | ***-0.646**** | ***-0.631**** | ***0.560**** | ***0.621**** |
| ***Ultimate Load*** | **-0.587**** | **-0.565**** | **-0.580**** | **-0.576**** | **0.456**** | **0.571**** |
| ***Stiffness*** | **-0.654**** | **-0.608**** | **-0.620**** | **-0.629**** | **0.421**** | **0.601**** |
| ***Elastic Energy*** | **-0.414**** | **-0.401**** | **-0.400**** | **-0.387**** | **0.363*** | **0.380**** |
| ***Plastic Energy*** | **-0.477**** | **-0.453**** | **-0.471**** | **-0.452**** | **0.398**** | **0.433**** |
| ***Ultimate Strength*** | -0.229 | **-0.293*** | **-0.280*** | -0.251 | 0.262 | 0.239 |
| ***Elastic Modulus*** | -0.238 | **-0.301*** | -0.265 | **-0.291*** | 0.135 | 0.273 |
| ***Plast. Toughness*** | **-0.299*** | **-0.306*** | **-0.324*** | **-0.293*** | **0.315*** | 0.277 |

### Disclosure

None declared

### P8

### Unilateral Cerebral Palsy and Bilateral Bone Health: Are Both Limbs Compromised?

Erin Hodgson[1,2,3,4,5], Elizabeth Condliffe[1,2,4,6,7,5], Leigh Gabel[1,2,3,4,5]

[1]Department of Biomedical Engineering, University of Calgary, Calgary, Canada. [2]Faculty of Kinesiology, University of Calgary, Calgary, Canada. [3]McCaig Institute for Bone and Joint Health, University of Calgary, Calgary, Canada. [4]Alberta Children's Hospital Research Institute, University of Calgary, Calgary, Canada. [5]Hotchkiss Brain Institute, University of Calgary, Calgary, Canada. [6]Department of Clinical Neurosciences, University of Calgary, Calgary, Canada. [7]Department of Pediatrics, University of Calgary, Calgary, Canada

**Abstract**

**Objectives:** Bone health is a significant concern in adolescents with cerebral palsy (CP). Despite known deficits, uncertainty remains about the implications of unilateral CP on bilateral musculoskeletal health. Terminology in the literature often assumes that the contralateral limb acts as a "control"; however, asymmetries from the diagnosis result in compensatory loading, balance challenges, and altered gait, creating muscle weakness between limbs. This unique within-person model enables exploration of between-limb differences in bone under identical systemic factors (e.g. genetics, medication, diet), allowing us to assess whether the less-impaired limb can serve as a control. This preliminary analysis explored differences in 1) bone geometry, density and strength, and muscle area between the more- and less-impaired limbs in individuals with CP and 2) between the more- and less-impaired limb Z-scores to typically developing peers.

**Methods:** Nine participants aged 10-18 years with unilateral CP underwent peripheral quantitative computed tomography scans of both tibiae at the distal metaphysis (3%) and diaphysis (38% and 66%). Data were converted to age-, sex-, and ethnicity-matched Z-scores from typically developing normative data. Paired t-tests evaluated differences between the more- and less-impaired limb Z-scores. One-sample Wilcoxon signed-rank tests compared each limb's Z-scores against zero.

**Results**: The more-impaired limb was significantly shorter and had lower metaphyseal trabecular bone mineral density (Tb.BMD), total bone mineral content (Tt.BMC), and diaphyseal muscle cross-sectional area (MCSA) than the less-impaired limb ($p<0.05$ to $p<0.01$). No differences were observed between limbs at the 38% slice in cortical content, density, thickness, area, circumference, or section modulus ($p=0.353$ to $p=0.095$). Compared to typically developing reference data, both limbs had lower Tb.BMD, cortical BMC, cortical thickness, cortical area, endosteal circumference, and MCSA ($p<0.05$ to $p<0.01$), but Tt.BMC and section modulus were only lower in the more-impaired limb ($p=0.021$ and $p=0.042$). Cortical BMD did not differ compared to the reference population in either ($p=0.655$ and $p=0.944$).

**Conclusion**: Most outcomes were compromised in adolescents with unilateral CP compared to typically developing peers. However, the between-limb loading difference in adolescents with unilateral CP appears to affect only trabecular and muscle measures, not cortical outcomes. Findings will be confirmed in a larger cohort of adolescents with unilateral CP.

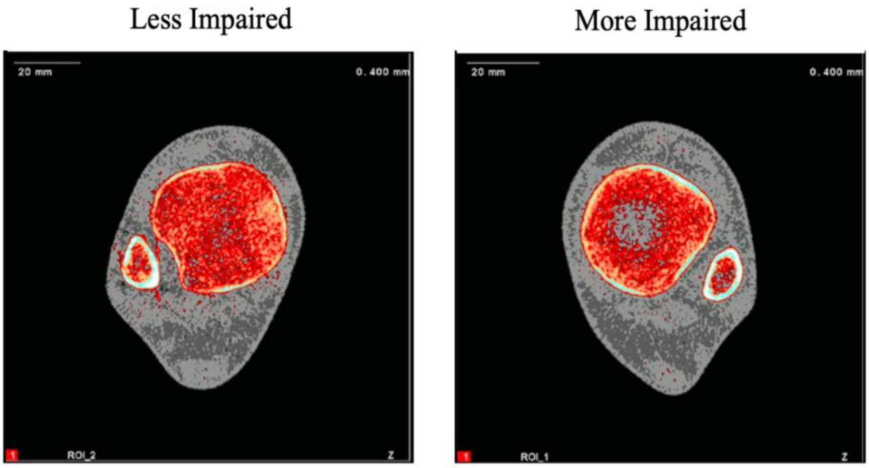

**Figure 1**: Peripheral quantitative computed tomography (pQCT) scan from the 3% distal tibia of the less-impaired (left) and more-impaired (right) limb of a 13-year-old participant with unilateral cerebral palsy (CP), GMFCS level I.

### Disclosure

None declared

### P9

### Severe Vertebral Fragility Fractures Following Chronic High-Potency Topical Corticosteroid Exposure in a Child with Undiagnosed Multisystem Disorder, Ichthyosiform Rash, and JAK1 Variant of Uncertain Significance

Eman Kamaleldeen[1], Vicky Diba[2], Nicola Crabtree[1,3], Mansoor Safdar[4], Vrinda Saraff[5], Ana Paula Merelez-Gonsalez[5], Ruchi Nadar[5]

[1]Department of Endocrinology, Birmingham Women's and Children's Hospital, Brimingham, UK. [2]Department of Dermatology , Birmingham Women's and Children's Hospital, Brimingham, UK. [3]Department of Metabolism and Systems Science, University of Birmingham, Brimingham, UK. [4]Department of Paediatrics, Northampton General Hospital, Northampton, UK. [5]Department of Endocrinology, Birmingham Women's and Children's Hospital,, Brimingham, UK

### Abstract

BACKGROUND:

Osteoporotic vertebral compression fractures associated with long-term topical corticosteroid use in children are rarely reported. We describe a child with a complex multisystem disorder who developed severe, progressive vertebral fractures following high cumulative topical steroid exposure.

PRESENTING PROBLEM:

A 14.3-year-old female with a longstanding undiagnosed complex multisystem disorder and an ichthyosiform rash presented with 6–8 weeks of worsening lower back pain and reduced mobility. Retrospective estimation revealed a cumulative exposure exceeding 2300 g of mometasone (a potent topical steroid) equivalent, related to frequent disease exacerbations. Acitretin and oral hydrocortisone replacement therapy were ongoing. Comorbidities included dilated cardiomyopathy, chronic diarrhoea, colonic ulceration, malnutrition, and gastrostomy feeding with elemental formula. There was no history of long-bone fractures or familial bone disease. A *JAK1* variant of uncertain significance was identified; however, there was no definitive unifying diagnosis. Examination revealed Cushingoid facies, severe growth restriction (height 120 cm, Z-score -6.43; weight 27.7 kg, Z-score -4.38; BMI 19.36 kg/m$^2$, Z-score -0.1), extensive inflammatory skin lesions, pubertal delay (Tanner breast stage II-III, no pubic or axillary hair), lower thoracolumbar tenderness and pain on forward flexion, without spinal deformity or neurological deficit. She was normotensive. MRI spine demonstrated mild–moderate anterior vertebral compression fractures from T11–L4 with marrow oedema indicating acute injury. Vertebral fracture assessment three months later showed rapid progression with new severe (grade III) fractures at T8–T10, worsening of T11 to severe, progression of T12 and L3–L4 to moderate (grade II) , while L1–L2 remained moderate. (Fig 1 A: MRI and B: VFA images). DXA confirmed severe osteoporosis (lumbar spine BMAD Z-score -4.8; whole body less head Z-score -4.0). Inflammatory markers were elevated (CRP 16 mg/L, ESR 70 mm/hr). LHRH showed pubertal response, with low oestradiol (12 pmol/L). Bone age was delayed by three years.

CLINICAL MANAGEMENT:

After extensive MDT discussion, intravenous half-dose zoledronate (0.025 mg/kg) was initiated, with prednisolone premedication to reduce risk of cutaneous flare. Treatment was well tolerated. Further cyclical zoledronate and pubertal monitoring are planned.

DISCUSSION:

Osteoporosis was multifactorial, with topical steroid exposure, delayed puberty, chronic inflammation, multisystem involvement and malnutrition, contributing to fracture risk. There is a need for active osteoporosis screening in children receiving long-term topical corticosteroids.

Figure 1:A MRI and B VFA images showing worsening vertebral fractures

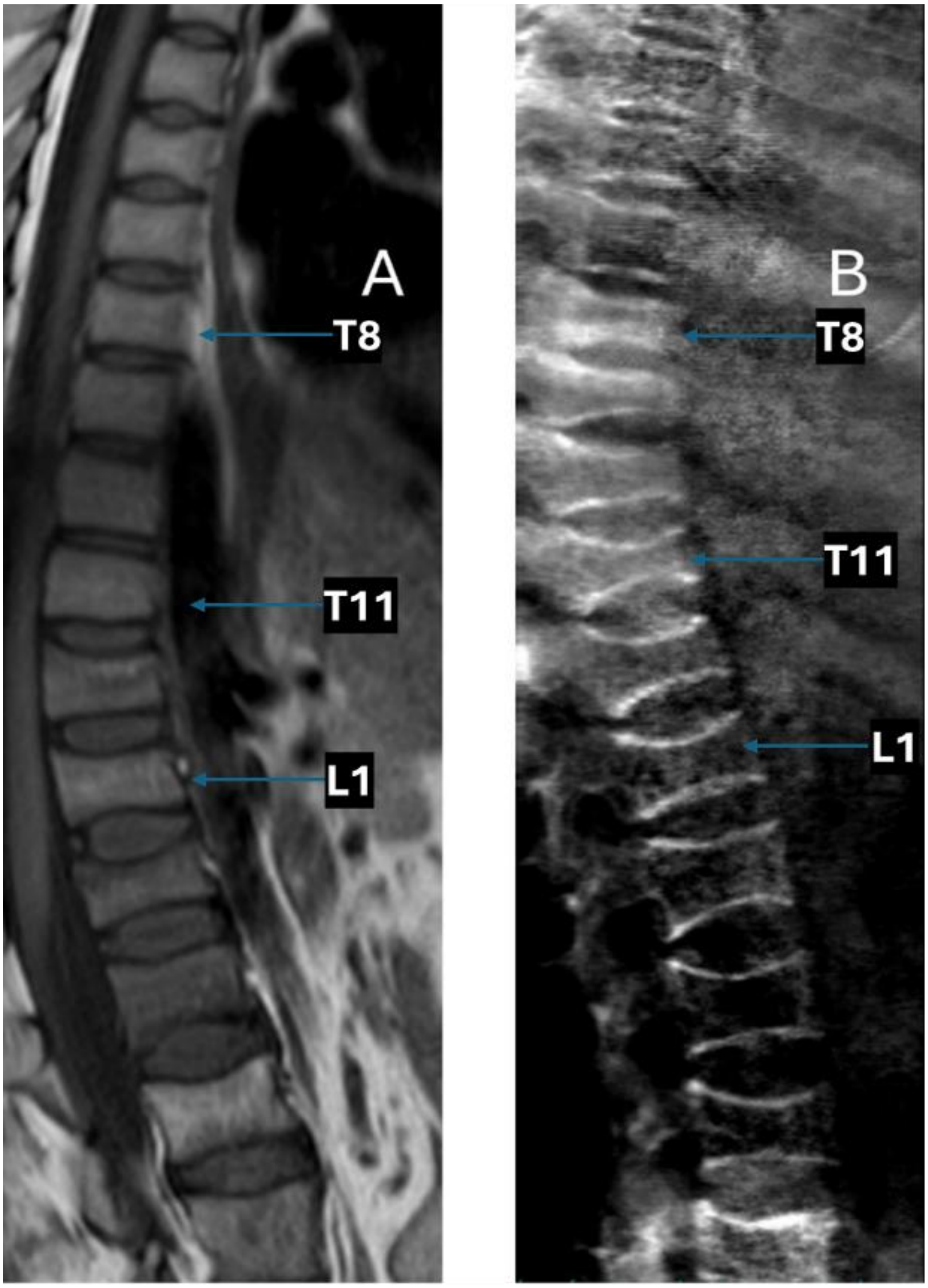

### Disclosure

None declared

### P10

### Primary Osteoporosis and Intrafamilial Variability in Hajdu–Cheney Syndrome: Longitudinal Skeletal Evolution and Differential Therapeutic Responses

Ruggero Lanzafame[1], Thomas Zoller[1], Angelo Pietrobelli[1,2], Giorgio Piacentini[1], Rossella Gaudino[1], Alessandra Guzzo[3], Franco Antoniazzi[1]

[1]University of Verona, Department of Surgical Science, Dentistry, Gynecology and Pediatrics, Verona, Italy. [2]Pennington Biomedical Research Center, Baton Rouge, Louisiana, USA. [3]University of Verona, Department of Pathology and Diagnostics, Verona, Italy

### Abstract

**Background:**

Hajdu–Cheney syndrome (HCS) is an rare skeletal dysplasia caused by gain-of-function truncating variants in exon 34 of the *NOTCH2* gene, leading to sustained Notch signaling and profound disruption of bone remodeling. Progressive osteoporosis, vertebral fractures, and acro-osteolysis are hallmarks of the disorder, yet its natural history, age-dependent evolution, and therapeutic responsiveness remain poorly defined due to the scarcity of longitudinal data, particularly in pediatric patients.

**Presenting problem:**

We report a unique two-generation family with HCS carrying the same *NOTCH2* Gln2223Ter variant, offering a rare opportunity to investigate intrafamilial phenotypic variability and disease evolution across life stages. Despite identical genotypes, the mother and her son exhibited markedly divergent skeletal trajectories: a severe adult phenotype with advanced osteoporosis, multiple vertebral fractures, acro-osteolysis, and splenomegaly, versus a pediatric-onset form characterized by initially preserved bone mineral density (BMD), but early vertebral fractures and progressive spinal deformities. Notably, vertebral fragility in the child preceded overt densitometric impairment, challenging the reliance on BMD as a sole marker of skeletal risk in HCS.

**Clinical management:**

The mother was treated with denosumab (60 mg subcutaneously every 6 months), achieving significant BMD gains at the lumbar spine and femoral neck, without preventing progression of acro-osteolysis. The son received neridronate (2 mg/kg every 3 months) from early adolescence, resulting in stabilization of BMD and partial reduction in fracture incidence, but continued vertebral remodeling. These contrasting therapeutic responses highlight the limits of current antiresorptive strategies in modifying the core skeletal pathology driven by aberrant Notch signaling.

**Conclusions:**

This report is among the first to provide longitudinal, side-by-side characterization of pediatric and adult HCS within the same family, demonstrating that bone fragility in HCS reflects impaired bone quality rather than density alone. Our findings underscore HCS as a model of genetically determined primary osteoporosis, in which vertebral fractures may arise despite near-normal BMD. The documentation of age-dependent skeletal progression, intrafamilial heterogeneity, and differential treatment responses represents a novel contribution to the field and reinforces the need for disease-specific monitoring strategies that extend beyond densitometry.

### Disclosure

None declared

### P11

### Severe Acute Phase Reactions and Fracture-Free Interval Following Short-Course Zoledronate Therapy in Children with Immobility-Related Osteoporosis Presenting with Pathological Fractures

Ana Paula Gonzalez Mereles, Saraff Vrinda, Tugce Kandemir, Eman Kamaleldeen, Nicola Crabtree, Ruchi Nadar

Birmingham Women´s and Children´s NHS Foundation Trust, Birmingham, UK

### Abstract

Children with immobility are at high risk of pathological fractures. Short courses of bisphosphonates have been shown to improve bone density; however, their effect on reducing fracture risk is not well described. Zoledronic acid (ZA) related adverse events also require further investigation in this medically complex population.

**Objectives:**
To describe severe acute phase reactions (APR) of intravenous (IV) ZA in children with immobility-related osteoporosis and to estimate fracture-free survival following a course of ZA.

**Methods:**
Retrospective review of referrals for pathological fractures in children with immobility between 2018 and 2020, who received a course of ZA. ZA was administered at a dose of 0.035–0.05 mg/kg/infusion. A course of ZA was defined as three six-monthly infusions, while severe APR as a life-threatening event, or requiring medical intervention or hospital admission following ZA.

**Results:**
Seventeen patients (8 females and 9 males) were referred during the study period. One was excluded because they were unable to start treatment. At referral, eight patients had sustained one long-bone fracture, while the remainder had sustained two or more fractures. The femur was the most common fracture site (88.5%), followed by the tibia (7.7%).

The mean age at first ZA infusion was 7.44 ± 2.7 years. Adverse effect data were only available following the first ZA infusion.

Three patients had a severe APR after the first ZA infusion, including increased seizure frequency, chest pain with tachycardia, and a sepsis-like presentation. Patients were followed up for 13-82 months after completing a ZA course. During follow-up, seven patients sustained further fractures (four femur, one tibia, and one tibia-fibula). The time to the first post-ZA course fracture ranged from 13 to 51 months, with an estimated fracture-free survival of 0.58 (95% CI 0.37–0.90) at 48 months (Figure 1).

**Conclusions:**
Severe acute-phase reactions were frequent after the first ZA infusion. Cautious patient selection, multidisciplinary planning, and close monitoring are required in this population. Only 41% of treated children remained fracture-free after four years of completing treatment. Further studies are needed to clarify the effectiveness and safety of ZA in this complex population.

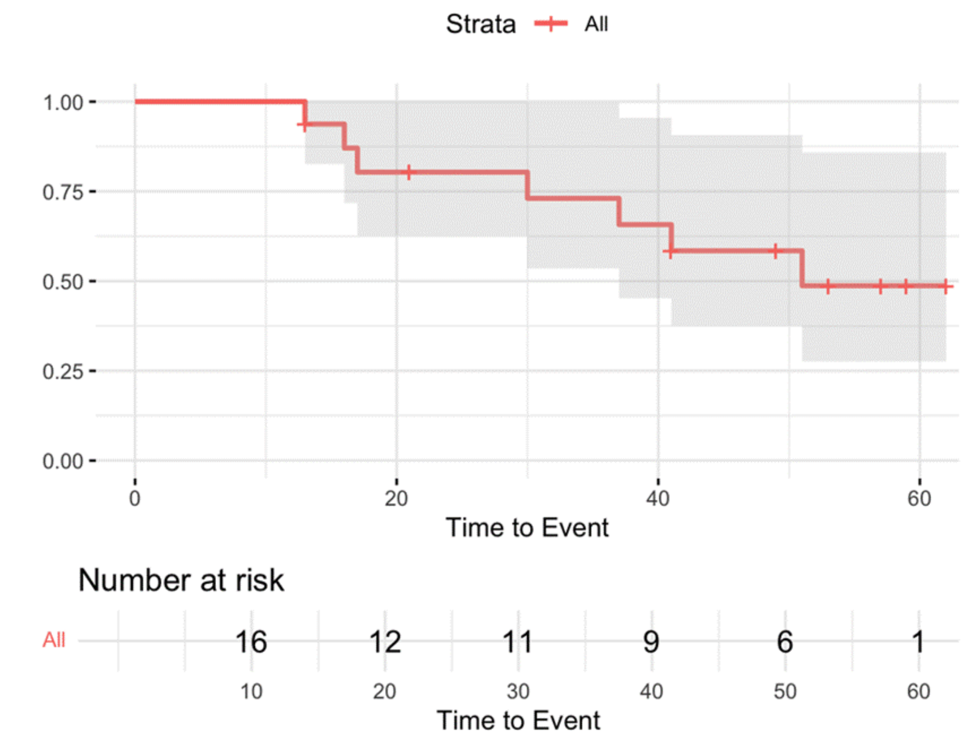

| Time (months) | Patients at Risk | Number of Events | Survival Probability | Standard Error | 95% CI Lower | 95% CI Upper |
|---|---|---|---|---|---|---|
| 48 | 7 | 6 | **0.584** | 0.131 | 0.377 | 0.906 |

Figure 1: Kaplan–Meier curve for fracture-free survival probability after a course of IV ZA

### Disclosure

None declared

### P12

### Osteogenesis Imperfecta and Movement Disorders – A Challenging Association

Belinda Crowe[1], Emilie Hupin[1], Zoe Bowman Bayles[1], Mark Heathfield[1], Alice Walker[1], Abigail Bolland[1], Jo Dartnell[2], Dominic Thompson[3], Alistair Calder[4], Andreas Roposch[5], Catherine DeVile[1]

[1]Osteogenesis Imperfecta Service, Great Ormond Street Hospital for Children NHS Foundation Trust, London, UK. [2]Orthopaedic Department, East Sussex Healthcare NHS Trust, East Sussex, UK. [3]Neurosurgery Department, Great Ormond Street Hospital for Children NHS Foundation Trust, London, UK. [4]Department of Radiology, Great Ormond Street Hospital for Children NHS Foundation Trust, London, UK. [5]Department of Orthopaedic Surgery, Great Ormond Street Hospital for Children NHS Foundation Trust, London, UK

### Abstract

**BACKGROUND:**

Movement disorders in osteogenesis imperfecta (OI) can be multifactorial – resulting from complications of OI, secondary to gene variants causing both bone fragility and neurological features, or co-occurring in children and young people (YP) with a dual diagnosis of OI and a movement disorder. Having both conditions presents management challenges which require coordinated input from a multidisciplinary team (MDT) with sufficient expertise. We describe three patients known to our paediatric neurodisability MDT OI Service.

**PRESENTING PROBLEM:**

Patient 1: A 16-year-old girl with *COL1A1*-related OI type 4 acquired a brain injury with skull fracture following a fall aged 6. She has a resultant unilateral dystonic motor disorder affecting her right leg, which limits her mobility, causing painful dystonic spasms and posturing.

Patient 2: A 9-year-old girl with OI type 15 secondary to a *WNT1* homozygous pathogenic duplication has presented with a bilateral dystonic motor disorder since infancy related to her gene variant.

Patient 3: A 7-year-old boy was born preterm at 34 weeks' gestation, with a dual diagnosis of OI type 1 secondary to a *COL1A2* gene variant and mild bilateral asymmetric cerebral palsy.

Additional clinical features are tabulated below:

| Clinical Features | Patient 1 | Patient 2 | Patient 3 |
|---|---|---|---|
| Sex | Female | Female | Male |
| Year of birth | 2009 | 2017 | 2018 |
| Ethnicity | White British | White British | Asian British - Bangladeshi |
| OI diagnosis | OI type 4 | OI type 15 | OI type 1 |
| Gene variant | *COL1A1:* c.2461G>A p.Gly821Ser exon 37 | *WNT1:* c.[216dup];[216dup] p.[Arg73fs)]; [(Arg73fs)] | *COL1A2:* missense variant c.343G>A p.(Gly115Arg) |
| Family history (FH) of | Negative FH of OI – looked after child in full | Father heterozygous for WNT1 variant. No | Positive paternal FH of |

| | | | |
|---|---|---|---|
| OI | guardianship | previous FH of OI.(Uniparental isodisomy for chromosome 12) | OI |
| Movement disorder diagnosis | Unilateral right dystonic motor disorder mainly affecting right leg and foot | Bilateral dystonic motor disorder with extensor posturing in infancy; orolingual dyskinesia leading to recurrent tongue and lip biting (improved over time); currently stereotypies and mild dystonic posturing of limbs | Bilateral dystonic, asymmetrical cerebral palsy secondary to prematurity, Gross Motor Function Classification System (GMFCS) level II |
| Age at movement disorder onset | 6 years 8 months | Infancy | Infancy |
| Movement disorder aetiology | Acquired parenchymal brain injury with skull fracture following a fall at home | Pathogenic mechanisms not fully understood; *WNT1* variants may affect signalling pathways critical for central nervous system development | Born preterm at 34 weeks' gestation |
| Neuroimaging | CT Head 2016: left parietal fracture with extradural haematoma causing mass effect and midline shift, basilar invagination (BI). MRI Brain 2017: BI and mature brain injury | MRI Brain: Normal (Incidental bilateral incomplete hippocampal rotation) | MRI Brain: Significant white matter volume loss with associated ventriculomegaly |
| Fracture history | Multilevel vertebral compression fractures and long bone fractures | Multilevel vertebral compression fractures and long bone fractures, ongoing upper limb fractures | Likely in-utero right $7^{th}$ rib fracture, fracture of right $4^{th}$ +/- $5^{th}$ metatarsals 2022 |
| Other clinical features | Dentinogenesis imperfecta, BI on MRI brain 2015, occipito-cervical fusion 2019, left convex lumbar scoliosis Cobb angle 30°, ongoing headaches and chronic pain | Bilateral congenital ptosis, Type 2N von Willebrand disease, from 3 months old left convex thoracic scoliosis, Cobb angle 22°. Progressive - aged 8 years Cobb angle 51° | Nosebleeds, mild tightness right knee extension. In standing: bilateral calcaneovalgus of feet, right > left, with midfoot broaching and Achilles tendon tightness |

| | | | |
|---|---|---|---|
| OI management | Bisphosphonate infusions commenced 2012 – ongoing | Bisphosphonate infusions commenced 2018, last given 2024. Compliance difficult due to autism spectrum disorder (ASD), finds hospital attendance stressful | Bisphosphonate infusions commenced 2024 for management of musculoskeletal pain and fatigue – ongoing |
| Most recent bone mineral density (BMD) Z-score | BMD Z score = -2.3, BMAD Z score = -0.31 | BMD Z score = -3.7, BMAD Z score = -4.64 | BMD Z score = -1.7, BMAD Z score = -1.51 |
| Movement disorder management | Repeated ultrasound guided intramuscular Botulinum toxin injections to right leg, ankle and toes with topical local anaesthetic / nitrous oxide sedation. Oral gabapentin for symptomatic relief. Provision of ankle foot orthosis and post-injection physiotherapy | As a wheelchair user experiences ongoing involuntary dystonic posturing of mouth, tongue and limbs and stereotypies. Since these are not causing functional difficulties or pain, our MDT management has focused on 24-hour postural management and provision of a Lycra suit | Orthotic management with bilateral fixed ankle foot orthoses, occasional Kaye walker use over longer distances. Ongoing physiotherapy and occupational therapy input. No other tonal management currently required |
| Orthopaedic management | Left femoral intramedullary rod insertion. Screw fixation and tibialis posterior lengthening for recent right 5th metatarsal fracture non-union | Bilateral lower limb intramedullary rodding | Hip surveillance and monitoring of foot posture |
| Neurodevelopment | Slow processing and significant verbal memory difficulties post brain injury. Pain Management Team input for symptoms of chronic pain | ASD – scores well above chronological age on cognitive assessment. Recently referred to OI Team psychologist due to hospital-related anxiety | Global developmental impairment but making steady progress. Receives one-to-one input at school |

**CLINICAL MANAGEMENT:**

Treatment of co-occurring OI and movement disorders requires special consideration e.g. Patient 1 requires careful handling during toxin injections to minimise fracture risk. Movement disorder management is based on functional goals and optimising participation – frameworks such as the World Health Organization's International Classification of Functioning, Disability and Health – Children & Youth Version (ICF-CY) and

CanChild's F-words (Functioning, Family, Fitness, Fun, Friends and Future) are appropriate goal-setting tools for both OI and movement disorders.

**DISCUSSION:**

Osteogenesis imperfecta and movement disorders can be associated for various reasons, and co-occurrence makes clinical management more complex. Co-ordinated input from an MDT with expertise in both domains is required to adequately assess and manage children and YP with these presentations, taking a holistic approach across the lifespan to treat both OI and movement disorder symptoms. Reasonable adjustments may be required with careful consideration of longer-term outcomes. Tools such as the ICF-CY and F-words are useful to set meaningful personalised goals for management, optimising participation.

### Disclosure

None declared

### P13

### Assessment of Bone Mass and Fracture Risk Using Trabecular Bone Score in Children with Autoimmune Gastrointestinal Diseases

Anna Łupińska[1,2], Sara Aszkiełowicz[1], Ewelina Sośnierz[1], Arkadiusz Zygmunt[1,2], Renata Stawerska[1,2]

[1]Department of Endocrinology and Metabolic Diseases, Polish Mother's Memorial Hospital-Research Institute, Łódź, Poland. [2]Department of Developmental Age and Adult Endocrinology, Medical University of Lodz, Łódź, Poland

### Abstract

### Objectives:

Trabecular Bone Score (TBS) is widely used to assess bone microarchitecture and fracture risk in adults; however, its clinical utility in pediatric populations remains unclear due to limited reference data. This study aimed to evaluate TBS in children with autoimmune gastrointestinal diseases and to investigate its associations with clinical, laboratory, and densitometric parameters.

### Methods:

This retrospective study included 152 children aged 5–18 years (mean age 13.15 ± 3.24 years; 61 girls, 91 boys). Participants were divided into three groups: children with autoimmune gastrointestinal diseases (n = 45; ulcerative colitis, Crohn's disease, and celiac disease), children with a history of fractures (n = 37), and controls without fractures or gastrointestinal disease (n = 70). Data collected included anthropometric measurements, pubertal stage, and serum 25-hydroxyvitamin D (25OHD) levels. Dual-energy X-ray absorptiometry (DXA) was performed at the total body less head (TBLH) and lumbar spine. Areal bone mineral density (aBMD) Z-scores were adjusted for height-for-age Z-score (HAZ). TBS values were normalized for pubertal stage and expressed as TBS Z-scores.

### Results:

Low bone mass (aBMD Z-score ≤ −2) was observed in 30.3% of participants at TBLH and 11.1% at the lumbar spine; after height adjustment, prevalence was 19.1% and 5.2%, respectively. A TBS Z-score < −2 was found in 5.2% of children, between −1 and −2 in 20.4%, and > −1 in 74.3%. No significant differences in TBS or TBS Z-scores were observed among the three groups. In the overall cohort, TBS correlated positively with age, height SDS, weight SDS, BMI SDS, and DXA-derived parameters. TBS Z-scores were positively associated with aBMD Z-scores and HAZ-adjusted aBMD at both skeletal sites. No association was found between TBS and serum 25OHD levels. Children with fractures had significantly lower HAZ-adjusted lumbar spine aBMD compared with the other groups ($p < 0.05$).

### Conclusions:

TBS is associated with growth-related and densitometric parameters but not with vitamin D status in children. This study did not demonstrate increased fracture risk assessed by TBS in children with autoimmune gastrointestinal diseases. Larger, prospective pediatric studies are needed to define the clinical role of TBS in fracture risk assessment.

### Disclosure

None declared

**P14**

Abstract withdrawn

### P15

### Burden of Sleep Disturbance and Daytime Sleepiness in Osteogenesis Imperfecta and its Impact on Quality of Life

Asli Derya Kardelen Al[1,2], Cansu Koc Calisgan[1], Guven Toksoy[3], Firdevs Bas[1]

[1]Division of Pediatric Endocrinology, Department of Pediatrics, Istanbul Faculty of Medicine, Istanbul University, Istanbul, Turkey. [2]Department of Genetics, Institute of Graduate Studies in Health Science, Istanbul University, Istanbul, Turkey. [3]İstanbul University, İstanbul Faculty of Medicine, Department of Medical Genetics, Istanbul, Turkey

### Abstract

### Objective:

To assess sleep quality and excessive daytime sleepiness, with particular emphasis on chronic pain, and to examine their associations with patient- and parent-reported health-related quality of life (HRQoL) in individuals with osteogenesis imperfecta (OI) aged 8–23 years.

#### Methods:

This single-center, cross-sectional observational study analyzed questionnaire data from 20 patients with OI aged 8–23 years and their primary caregivers (n=20), and compared them with 16 age-matched healthy controls and their primary caregivers (n=16). Participants completed the Pittsburgh Sleep Quality Index (PSQI) and the Epworth Sleepiness Scale (ESS) to assess sleep quality and daytime sleepiness, respectively. Health-related quality of life was assessed using age-appropriate Pediatric Quality of Life Inventory (PedsQL 4.0) self-report forms, and caregivers completed the PedsQL 4.0 parent-proxy report. Clinical data of OI patients (OI type, fracture burden, treatment status, pain, and mobility) were obtained from medical records.

### Results:

There were no significant differences between the OI and control groups in total sleep quality (PSQI total, p=0.859), daytime sleepiness (ESS total, p=0.293), or sleep efficiency (p=0.543). The prevalence of poor sleep (PSQI ≥5) was similar between groups (p=1.000), as was excessive daytime sleepiness (ESS >10; p=1.000). PSQI Component 5 (sleep disturbances) did not differ between groups (p=0.088). Child self-reported overall HRQoL differed between groups (PedsQL child total, p=0.015), and parent-proxy psychosocial health was lower in the OI group (p=0.046). Within the OI group, PSQI total was inversely correlated with child-reported HRQoL total (ρ=−0.65, p=0.0045), and pain-related nocturnal sleep disruption (PSQI item 5i) was also inversely correlated with child-reported HRQoL (ρ=−0.61, p=0.0088).

#### Conclusion:

Overall sleep quality and daytime sleepiness did not differ between children with OI and healthy controls. However, within the OI group, sleep disturbances—particularly pain-related nocturnal disruption—were strongly associated with poorer child-reported HRQoL, indicating that sleep quality is clinically relevant to well-being in children with OI.

### Disclosure

None declared

### P16

### Impact of Trabecular Bone Score Changes on Vertebral Fracture and Lumbar Spine Bone Mineral Density in Systemic Lupus Erythematosus Children and Adolescents

Jittra Ruktrirong[1], Kulisara Wangwarawut[1], Pemiga Sukhupanyarak[2], Monthira Chowichian[3], Trirat Boonya-ussadorn[4], Voraluck Phatarakijnirund[1]

[1]Division of Endocrinology, Department of Pediatrics, Phramongkutklao College of Medicine and Phramongkutklao Hospital, Bangkok, Thailand. [2]Division of Endocrinology, Department of Pediatrics, Buddhachinaraj Hospital, Phitsanulok, Thailand. [3]Division of Rheumatology, Department of Pediatrics, Phramongkutklao College of Medicine and Phramongkutklao Hospital,, Bangkok, Thailand. [4]Department of Radiology, Phramongkutklao College of Medicine and Phramongkutklao Hospital, Bangkok, Thailand

### Abstract

**Background:**

Glucocorticoid-induced osteoporosis (GIO) is one of the most common complications occurring in systemic lupus erythematosus (SLE) children and adolescents receiving glucocorticoid treatment. Trabecular bone score (TBS) is a non-invasive technique for bone quality assessment.

**Objectives:**

To compare the incidence of vertebral fracture (VF) and changes in lumbar spine bone mineral density (BMD) in pediatric patients with systemic lupus erythematosus receiving glucocorticoid therapy who had decreased versus increased trabecular bone score over a 1-year follow-up.

**Methods:**

This retrospective cohort included SLE patients treated with glucocorticoids who underwent at least two TBS assessments separated by ≥12 months. TBS was acquired from DXA-BMD lumbar spine imaging. Clinical parameters, VF and BMD were compared between those who decreased TBS Z-score and increased TBS Z-score.

**Results:**

Thirsty-one SLE patients were included. Of them, 3 (9.6%) were male. Fourteen patients experienced a decrease in TBS Z-score, while 17 showed an increase. The interval between examinations was 16 months (13, 21) in the decreased TBS Z-score group and 17 months (15, 24) in the increased TBS Z-score group, with no statistically significant difference between groups (p 0.24). Three cases of VF were identified; two patients were in the decreased TBS Z-score group and one was in the increased group, however, the difference in VF incidence between two groups was not statistically significant (p 0.43). Both LS BMD Z-score and LS BMAD Z-score were significantly lower in the decreased TBS Z-score group compared to the increased group.

**Conclusions:**

Pediatric patients with SLE receiving glucocorticoid treatment who had a decreased TBS Z-score were at higher risk of vertebral fractures and had a significantly greater decrease in lumbar spine bone mineral density compared with those with an increased TBS Z-score.

### Disclosure

None declared

### P17

### Fracture Burden and Predictors Among Hong Kong Children with Chronic Illness

Joanna Yuet-ling Tung, Sarah Wing-yiu Poon, Gloria Shir-wey Pang

Hong Kong Children's Hospital, Hong Kong SAR, China

### Abstract

**Objectives:**

Children with chronic illness have a constellation of adverse factors predisposing them to develop fractures. This study aimed to assess fracture burden in children with chronic illness at risk of osteoporosis. It also aimed to identify predictors of incident fractures using clinical data, bone density parameters and bone turnover markers.

**Methods:**

This was a longitudinal cohort study conducted at Hong Kong Children's Hospital. Patients aged 6-19 years with conditions predisposing to secondary osteoporosis—such as immobility-related disorders (e.g., spinal muscular atrophy [SMA], Duchenne muscular dystrophy [DMD] etc.) or long-term glucocorticoid exposure (≥5 mg/day or 0.1 mg/kg/day prednisolone equivalent for ≥3 months)—were included. Patients on prior history of fractures, bone-targeted therapies (e.g., bisphosphonates), or with estimated glomerular filtration rate <90 mL/min/1.73 $m^2$ were excluded. Participants underwent annual assessments over two years, with the primary endpoint of new long bone or vertebral fractures. Evaluations included high-resolution peripheral quantitative computed tomography (HR-pQCT) at distal radius, dual-energy X-ray absorptiometry (DXA) with trabecular bone score (TBS), vertebral morphometry, bone age, serum bone profile, 25-hydroxyvitamin D, C-terminal telopeptide of type 1 collagen (CTX), procollagen type 1 N-terminal propeptide (P1NP), fibroblast growth factor 23 (FGF23)], CUHK-PARCY (Chinese University of Hong Kong Physical Activity Rating for Children and Youth), and Vignos scores (lower extremity function scale).

**Results:**

In total, 118 children were recruited; median age was 14.4 years (IQR 9.8-16.9); 59 were male. Median CUHK-PARCY score was 2 (IQR 1-3), Vignos score was 1 (IQR 1-2), and 75 (63.6%) had long-term steroid exposure. Eighteen were lost to follow-up; 100 completed two years. Twenty-two (22%) developed incident fractures (1 long bone [left femur], 21 vertebral). Those with fracture were more often male (81.3% vs 51.2%; p=0.026), with lower DXA LS BMD Z-scores (-2.3 vs -1.2; p=0.025) and TBS (1.300 vs 1.352; p=0.044). No significant differences were observed in steroid exposure, HR-pQCT parameters, biomarkers, CUHK-PARCY scores, or Vignos scales.

**Conclusions:**

A high incident fracture rate was observed in this cohort, with male sex, lower DXA LS BMD, and TBS as key predictors. These findings may facilitate future stratification of patients at highest risk.

### Disclosure

This project is funded by Health and Medical Research Fund (HMRF) Research Grant, the Health Bureau, HKSAR Government

### P18

### Longitudinal Changes in Lumbar Spine Bone Mineral Density in Indian Children with Beta Thalassemia Major

Anuradha Khadilkar[1], Chirantap Oza[2], Ripal Shah[3], Rubina Mandlik[1], Sharvani Patil[1]

[1]Hirabai Cowasji Jehangir Medical Research Institute, Jehangir Hospital,, Pune, India. [2]Consultant Pediatric Endocrinologist, EndoGrow, Ahmedabad, India. [3], Prathama Blood Centre, Ahmedabad, India

### Abstract

**Introduction:**

Bone health is significantly affected in Beta Thalassemia Major(BTM) because of ineffective erythropoiesis leading to marrow hyperplasia, iron overload, hypogonadism, vitamin D deficiency and renal dysfunction. While we have previously reported that prevalence of fractures in children with BTM is higher, repeated assessments are required to characterise the longitudinal course. Therefore, our objective was to assess changes in lumbar spine bone mineral density (LSBMD) between baseline and 1-year follow-up DXA scans in children with BTM.

**Method:**

This retrospective observational study included 40 children with BTM who had lumbar spine DXA assessments at baseline and at 1-year follow-up. Clinical, anthropometric, and laboratory data were extracted. DXA scans were performed using a GE Lunar Prodigy system (software version 13.31). Changes in lumbar spine bone mineral density (LSBMD) over one year were analysed using univariate analysis of covariance (ANCOVA).

**Results:**

Of the 40 participants, 8 were girls; mean age was 12.5 ± 7.4 years. At baseline, 28.2% (n = 11) had poor LSBMD (Z-score < –2.0). Children with poor LSBMD were older, lighter, and poorly chelated ($p < 0.05$). At 1-year follow-up, there was a significant decline in mean LSBMD Z-score, serum calcium, and vitamin D levels (Fig 1). During follow-up, 25% of those with normal baseline LSBMD (7/28) developed poor LSBMD, while 54.5% (6/11) with low baseline LSBMD improved to Z-scores > –2.0, coinciding with optimisation of transfusion, chelation, and calcium/vitamin D supplementation. LSBMD Z-score correlated positively with weight Z-score ($r = 0.53$, $p < 0.05$) and negatively with serum ferritin ($r = -0.46$, $p < 0.05$). A significant effect of vitamin D levels was observed, $F (5,1) = 6.756$, $p = 0.016$, $\eta^2 = 0.227$) on change in LSBMD Z score in univariate general linear regression.

**Conclusion:**

Children with BTM demonstrate a high burden of low LSBMD, particularly those who were underweight, poorly chelated, or vitamin D deficient. Over one year, deterioration in bone density was observed in a substantial proportion, despite improvement in others following optimisation of clinical care. Our findings highlight the dynamic nature of bone health in BTM and support the need for regular longitudinal monitoring and optimisation of transfusion, chelation, and vitamin D status.

Figure 1: Percent change in biochemical parameters and lumbar spine BMD Z-score over one year in children with beta thalassemia major.

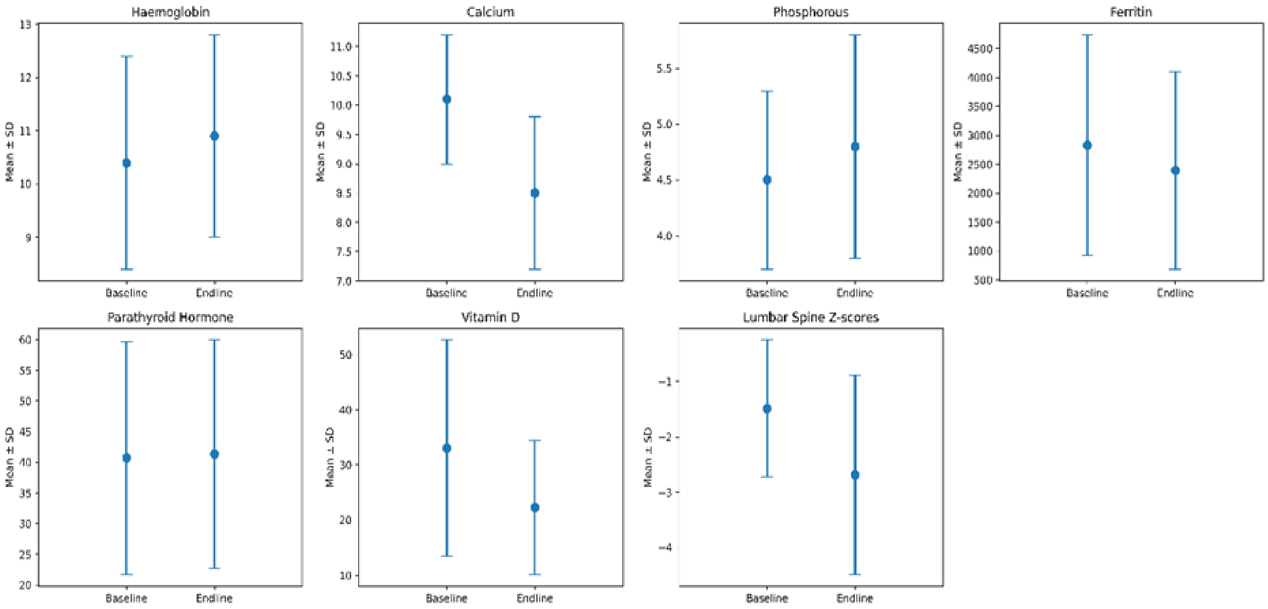

### Disclosure

None declared

### P19

### Reducing Muscle Spasticity in Children with Cerebral Palsy Improved Bone Geometry after 12 Months

Marianne Gagnon[1,2], Louis-Nicolas Veilleux[1,2], Dominique Audet[1], Sarah Ford[3], Rita Yap[1], David Hartal[3], Seyed Hosseinitabatabaei[1,3,4], Camille Costa[1], Eliane Rioux-Trottier[1,5], Bettina Willie[1,3,4], Alessandra Carriero[6], Jean-Pierre Farmer[1,7], Elizabeth Zimmermann[3]

[1]Shriners Hospital for Children, Montreal, Canada. [2]Department of Surgery, McGill University, Montreal, Canada. [3]Faculty of Dental Medicine and Oral Health Sciences, McGill University, Montreal, Canada. [4]Department of Biomedical Engineering, McGill University, Montreal, Canada. [5]Department of Surgery, Centre Hospitalier Universitaire Sainte-Justine, Montreal, Canada. [6]Department of Biomedical Engineering, The City College of New York, New York, USA. [7]Department of Pediatric Surgery, McGill University, Montreal, Canada

### Abstract

**Objectives:** Children with spastic, diplegic cerebral palsy (CP) have stiff muscles resulting in abnormal gait. Our research has shown that their lower limbs adapt to this biomechanical environment resulting in bone deformities, smaller cross-sectional area, and fewer trabeculae. A common treatment to reduce spasticity and improve motor function in young children with CP is selective dorsal rhizotomy (SDR), where sensory nerve rootlets associated with abnormal muscle tone are sectioned. This study investigated gait, bone density, and bone microarchitecture before and up to 12 months after SDR.

**Methods:** Children eligible for a SDR surgery were recruited based on: i) ambulatory spastic diplegia, ii) age of 3-8 years, iii) strength underlying spasticity. Functional outcomes included Modified Ashworth Scale, Gross Motor Function Measure (GMFM) and Gait Deviation Index (GDI). Areal BMD Z-scores at proximal and distal femur were measured with dual x-ray absorptiometry (DXA). High resolution peripheral quantitative computed tomography (HRpQCT) was performed at distal tibia in CP subgroup (n=5) compared to non-CP controls (n=7). Linear mixed models or fixed-effects models investigated effects of group, time, and interaction.

**Results:** In the CP group (n=11 males; n=1 female; age=5.3±0.8 years), SDR eliminated lower limb muscle group spasticity measured with Modified Ashworth scale. GMFM improved from Month12 to baseline by on average 4.7 points ($p<0.01$), while GDI increased by 25% from baseline to Month6 and Month12 ($p<0.001$). Kinematic gait analysis showed significant improvements, primarily in foot dorsiflexion and foot progression angles. Proximal and distal femur aBMD Z-scores transiently declined at Month6 after SDR ($p<0.01$). HRpQCT-measured trabecular area, cortical area, cortical perimeter, and trabecular bone volume fraction increased by 6-18% at the distal tibia from baseline to Month12 in CP and non-CP control groups.

**Conclusions:** The elimination of muscle spasticity with SDR improved gait patterns in children with CP. Even though children with CP had smaller bone geometry than controls, gains in bone geometry over 12 months were similar to non-CP controls. Children with spastic diplegia have fewer trabeculae; longer follow-up may be required to observe new trabeculae adapted to the new biomechanics. These findings highlight the complex but mainly beneficial musculoskeletal adaptations following elimination of spasticity.

### Disclosure

None declared.

### P20

### Assessment of Bone Mineral Density and Trabecular Bone Score in Adolescents and Young Adults with Primary Ovarian Insufficiency

Noah Gruber[1,2], Shai Bar-Shira[2], Myriam Safrai[1,2], Liana Tripto-Shkolnik[1,2], Moran Shapira[1,2], Yael Levy-Shraga[1,2]

[1]Sheba Medical Center, Ramat Gan, Israel. [2]Faculty of Medical and Health Sciences, Tel-Aviv University, Tel Aviv, Israel

### Abstract

**Background:**

Primary Ovarian Insufficiency (POI) in adolescents and young women is associated with reduced bone mineral density (BMD) and elevated fracture risk. While BMD reflects bone quantity, the trabecular bone score (TBS) offers complementary insights into bone quality. Data on bone health parameters in young females with POI, particularly comparing Turner syndrome (TS) and non-TS etiologies, remain scarce. We aimed to evaluate BMD and TBS in this population and assess the impact of hormone replacement therapy (HRT).

**Methods:**

We conducted a retrospective study of girls aged 10–24 years with POI who underwent dual-energy X-ray absorptiometry (DXA) at our center between 2020–2025. POI was defined as irregular menses and FSH >20 IU/L on two occasions. Lumbar spine BMD was assessed (GE Lunar Prodigy) and adjusted for size using bone mineral apparent density (BMAD). Trabecular bone microarchitecture was analyzed using TBS iNsight (Medimaps). Z-scores were calculated from age- and sex-specific reference data. Clinical and hormonal characteristics were retrieved from medical records and compared between TS and non-TS patients.

**Results:**

Twenty-eight girls (mean age 18.4 ± 3.7 years) underwent DXA. POI was diagnosed at a mean age of 13.9 ± 3.1 years, earlier in TS vs. non-TS (11.6 ± 1.6 vs. 15.4 ± 2.9 years, $p = 0.001$). At baseline, mean lumbar spine BMD Z-score was –1.50 ± 1.20, and TBLH Z-score –0.67 ± 0.81, both below normative values. TBS Z-scores were also reduced (–0.73 ± 0.8), and significantly lower in non-TS compared with TS (–1.01 ± 0.74 vs. –0.23 ± 0.70, $p = 0.01$). TS patients had longer HRT exposure before DXA (3.9 ± 3.1 vs. 0.3 ± 1.6 years, $p = 0.03$). In four girls with follow-up DXA, TBS Z-scores improved after ~2 years of HRT (–0.83 ± 0.8 to –0.32 ± 0.62, NS).

**Conclusions:**

Adolescents and young women with POI exhibit impaired bone mass and quality. Non-TS patients are particularly vulnerable, with lower BMD and TBS compared with TS, possibly reflecting shorter HRT exposure. These findings underscore the importance of early diagnosis and timely initiation of HRT to optimize bone health in girls with POI.

### Disclosure

None declared

### P21

### Bone Mineral Density and Bone Formation Markers Are Associated with Degree of Gross Motor Impairment in Children with Cerebral Palsy

Halley Wasserman[1,2], Lindsey Hornung King[1], Jilda Vargus-Adams[1,2], Heidi Kalkwarf[1]

[1]Cincinnati Children's Hospital Medical Center, Cincinnati, USA. [2]University of Cincinnati, Cincinnati, USA

### Abstract

**Objective:** Clarify relationships between bone turnover markers, bone mineral density (BMD) and gross motor impairment in prepubertal children with cerebral palsy (CP)

**Methods:** A cross-sectional study from June 2021-December 2022 enrolled 70 children with CP and 30 neurotypical children. Children with CP were categorized as mild (Gross Motor Function Classification Score (GMFCS) I or II) or moderate-severe (GMFCS III-V). Measurement of osteocalcin, C-terminal telopeptide, sclerostin, Intact N-Terminal Propeptide of Type 1 Procollagen and 25-OH-vitamin D, was obtained via morning blood draw. Whole body less head (WBLH), lumbar spine (LS), total hip (TH), 1/3 distal radius (DR) and lateral distal femur (LDF) BMD was obtained by DXA (Hologic), with Z-scores calculated using age and sex specific reference ranges (Zemel 2025). Caregivers completed a 24-hr dietary recall for participants. The degree of gross motor impairment in those with CP was quantified by the Pediatric Outcomes Data Collection Instrument (PODCI). Pearson correlations were calculated to determine the relationship between bone turnover markers and BMD at each skeletal site.

**Results:** Children with moderate-severe CP (n=36) had lower height for age Z-score (HAZ) than children with mild CP (n=34) or controls (n=30), though BMI was similar among groups. Daily vitamin D intake was higher in children with moderate-severe CP (20.2mcg/day vs. 7.1mcg/day, p=0.003), though most participants were vitamin D sufficient (>30ng/mL). Race-neutral BMD Z-scores positively correlated with global functioning on PODCI: LS (r=0.56), TH (r=0.67), FN (r=0.67) and LDF (R1: r=0.57, R2 r=0.56, R3 r=0.45) (all p<0.001). Bone formation markers (osteocalcin, P1NP) were lower in those with moderate-severe CP. In controls, there was no correlation between serum P1NP and BMD Z-scores, however P1NP was weakly correlated with race neutral BMD Z-score at most skeletal sites (r values LS: 0.45, TH: 0.41, FN: 0.40, DR: 0.36, LDF R1 0.33, LDF R2 0.31, LDF R3 0.34, all p-values < 0.05).

**Conclusions:** In this modern cohort of prepubertal children with cerebral palsy, BMD Z-scores were already low at weight-bearing skeletal sites with evidence of reduced bone formation compared to healthy controls. Further investigation into anabolic pathways is needed to support bone health in children with moderate-severe CP.

Table 1:

| | **Controls** n=30 | **GMFCS I & II** n=34 | **GMFCS III, IV, V** n=36 | **P-value** |
|---|---|---|---|---|
| Age (years) | 8.4 (7.6, 9.5) | 7.9 (6.7, 9.0) | 7.7 (6.6, 9.0) | 0.16 |
| Sex (male) | 16 (53%) | 21 (62%) | 24 (67%) | 0.54 |
| Race | | | | 0.27 |
| White/Caucasian | 25 (83%) | 28 (82%) | 34 (94%) | |

| | | | | |
|---|---|---|---|---|
| Black/African American<br>Other | 2 (7%)<br>3 (10%) | 4 (12%)<br>2 (6%) | 0 (0%)<br>2 (6%) | |
| Height*-for-age Z-score (HAZ) | 0.27 (-0.10, 0.92) | -0.15 (-0.94, 0.79) | -1.51 (-2.81, -0.56) | **<0.001** |
| Weight-for-age Z-score (WAZ) | 0.15 (-0.42, 0.90) | 0.10 (-1.02, 1.42) | -1.10 (-2.14, 0.09) | **<0.001** |
| BMI Z-score | 0.02 (-0.79, 0.85) | 0.45 (-0.82, 1.52) | -0.03 (-0.95, 0.81) | 0.37 |
| History of fracture | 5 (17%) | 7 (21%) | 4 (11%) | 0.55 |
| Estimated daily calcium intake (mg) | 1062 (808, 1218) | 757 (529, 1148) | 997 (763, 1283) | 0.26 |
| On Proton Pump Inhibitor | 2/29 (7%) | 10 (29%) | 14/35 (40%) | **0.006** |
| On seizure medication | 0 (0%) | 13 (38%) | 20 (56%) | **<0.001** |
| **DXA Bone Mineral Density Race Neutral Z-scores** | | | | |
| *Lumbar spine (n)* | *30* | *34* | *30* | |
| Lumbar Spine | 0.21 (-0.54, 0.80) | -0.38 (-1.00, 0.90) | -1.46 (-2.83, -0.85) | **<0.001** |
| Lumbar Spine (height adjusted) | -0.03 (-0.57, 0.49) | -0.04 (-0.76, 0.84) | -0.74 (-1.45, -0.19) | **0.008** |
| *Whole Body Less Head (n)* | *30* | *33* | *9* | |
| Whole Body Less Head | -0.54 (-1.02, 0.29) | -1.04 (-1.85, -0.21) | -2.19 (-3.54, -1.75) | **<0.001** |
| Whole Body Less Head (height adjusted) | -0.63 (-1.22, 0.13) | -0.75 (-1.60, -0.07) | -1.55 (-1.63, -1.13) | **0.03** |
| *Total Hip (n)* | *30* | *34* | *15* | |
| Total Hip | -0.35 (-0.76, 0.34) | -1.10 (-1.73, 0.61) | -2.76 (-4.16, -1.43) | **<0.001** |
| Total Hip (height adjusted) | -0.27 (-0.84, 0.15) | -0.88 (-2.02, 0.13) | -2.20 (-4.04, -1.18) | **<0.001** |
| Femoral Neck | -0.38 (-0.68, 0.40) | -1.08 (-1.80, 0.29) | -3.51 (-5.57, -2.03) | **<0.001** |

| | | | | |
|---|---|---|---|---|
| Femoral Neck (height adjusted) | -0.36 (-0.82, 0.35) | -1.08 (-1.73, -0.10) | -2.73 (-5.03, -1.83) | **<0.001** |
| *1/3 Distal Radius (n)* | *30* | *32* | *30* | |
| 1/3 Distal Radius | -0.35 (-0.83, 0.72) | 0.01 (-0.81, 0.66) | -0.90 (-2.02, -0.29) | **0.002** |
| *Lateral distal Femur (n)* | *30* | *33* | *31* | |
| Lateral Distal Femur R1 | 0.07 (-0.61, 0.59) | 0.02 (-1.13, 0.84) | -2.13 (-3.95, -1.21) | **<0.001** |
| Lateral Distal Femur R2 | -0.43 (-0.89, 0.20) | -0.55 (-1.32, 0.19) | -3.44 (-5.20, -1.39) | **<0.001** |
| Lateral Distal Femur R3 | -0.69 (-1.17, 0.26) | -0.50 (-1.59, 0.37) | -2.86 (-4.63, -1.17) | **<0.001** |
| **Bone Turnover Markers** | | | | |
| *(n)* | *30* | *34* | *36* | |
| Intact N-Terminal Propeptide of Type 1 Procollagen (pg/mL) | 601 (492-713) [343-1200] | 552 (456-729) [203-1082] | 474 (361-558) [187-831] | **0.005** |
| Sclerostin (pmol/L) | 28 (23-34) [8-51] | 23 (20-34) [6-43] | 24 (16-31) [3-48] | 0.10 |
| Osteocalcin (ng/mL) | 95 (77-123) [45-172] | 84 (65-95) [39-138] | 72 (49-92) [28-121] | **0.007** |
| C-terminal telopeptide (pg/mL) | 1400 (1200-2100) [600-2300] | 1200 (1000-1500) [800-2300] | 1200 (900-1400) [400-2100] | **0.01** |
| 25-hydroxyvitamin D (ng/mL) | 34.9 (30.5-40.4) [24.1-60.4] | 38.1 (31.4-44.7) [14.9-89.0] | 42.6 (33.6-52.7) [20.4-90.7] | **0.046** |

*Data presented as median (25th-75th percentile) [min-max] *Estimated height based on tibial length. Complete data unless otherwise specified by stated denominator.*

### Disclosure

No

### P22

### Bisphosphonate Therapy in SGMS2-Associated Autosomal Dominant Osteoporosis with Calvarial Doughnut Lesions – A Two Patient Case Series

Izabela Michałus[1], Anna Łupińska[2,1], Danuta Chlebna-Sokół[3], Arkadiusz Zygmunt[2,1], Renata Stawerska[2,1]

[1]Department of Endocrinology and Metabolic Diseases, Polish Mother's Memorial Hospital—Research Institute, Łódź, Poland. [2]Department of Developmental Age and Adult Endocrinology, Medical University of Lodz, Łódź, Poland. [3]Department of Paediatrics, Neonatal Pathology and Metabolic Bone Diseases, Medical University of Lodz, Łódź, Poland

### Abstract

### Background:

Loss-of-function mutations in *SGMS2*, encoding sphingomyelin synthase 2, have been implicated in Autosomal Dominant Osteoporosis with Calvarial Doughnut Lesions (ADOCDL). Affected individuals present with generalized low bone mineral density, frequent vertebral fractures, and abnormal skull ossification. Data regarding treatment are limited, although individual reports suggest potential benefits of bisphosphonate therapy.

**Presenting problem:**

The aim of this study was to evaluate the effects of bisphosphonate treatment in siblings with osteoporosis associated with ADOCDL.

### Clinical management:

Two female siblings harboring *SGMS2* mutations and presenting with a classical ADOCDL phenotype were included. Clinical examination revealed palpable sclerotic protuberances of the skull consistent with calvarial bone overgrowth. Dual-energy X-ray absorptiometry demonstrated significantly reduced bone mineral density. In patient 1, total body less head (TBLH) Z-score was −2.3 and spine Z-score −3.1; in patient 2, TBLH Z-score was −3.3 and spine Z-score −2.7. Radiological evaluation confirmed multiple vertebral fractures in both patients, leading to a diagnosis of osteoporosis.

Both patients were treated with intravenous bisphosphonates, initially sodium pamidronate (nine cycles), followed by zoledronic acid (two cycles). Treatment resulted in improvement in bone mineral density and radiological signs of vertebral remodeling.

### Summary:

Bisphosphonate therapy appears to be effective in patients with ADOCDL-related osteoporosis, leading to improved bone mineral density, vertebral restoration, and prevention of further fractures. These findings support the use of bisphosphonates as a therapeutic option in this rare genetic disorder.

### Disclosure

None declared

### P23

### Vertebral and Lower Extremity Fractures as the First Sign of Pediatric Scurvy: A Case Series

Jennifer Boulais[1], Regina Duperval[2], Anne Marie Sbrocchi[3,4], Melissa Fiscaletti[5,1]

[1]Université de Montréal, Montreal, Canada. [2]McGill University, Montreal, Canada. [3]Division of Pediatric Endocrinology, McGill University Health Center, Montreal, Canada. [4]Shriners Hospital for Children, Montreal, Canada. [5]Azrieli Research Center of CHU Sainte Justine, Montreal, Canada

### Abstract

BACKGROUND:

Scurvy, caused by vitamin C deficiency, remains a rare but preventable cause of musculoskeletal pathology. Children with severe restrictive dietary patterns often associated with autism spectrum disorder (ASD) are at increased risk. Skeletal manifestations may be severe, and delayed recognition can result in pathological fractures and long-term morbidity. We present a case series of three children with severe food selectivity and pathological vertebral or long bone fractures due to vitamin C deficiency, in whom delayed diagnosis was attributable to under-recognition of scurvy as a cause of pediatric fragility fractures.

PRESENTING PROBLEM:

Three patients aged 7 to 11 years with diagnosed or suspected ASD and long-term restrictive diets presented with pain and manifestations of scurvy. Patients A and B presented with progressive lower extremity pain associated with limp and refusal to bear weight, and patient C presented with progressive back pain. Two patients (B and C) presented with gingival hyperplasia and bleeding.

Imaging revealed low lumbar spine bone mineral density (BMD) with multiple thoracic or lumbar vertebral body fractures in patients A and C, respectively, and an insufficiency fracture at the distal femur in patient B. Serum vitamin C levels were below 10 µmol/L in all cases. Empiric treatment for suspected scurvy based on dietary history and clinical presentation was initiated prior to laboratory confirmation. Alternative causes of secondary osteoporosis were excluded in all cases.

CLINICAL MANAGEMENT:

Empiric vitamin C supplementation for suspected scurvy led to rapid clinical improvement by day 3 of treatment, with significant pain reduction and resumed ambulation in all patients. Treatment included initial high-dose vitamin C (125–300 mg/day), followed by long-term maintenance therapy (90–100 mg/day). Follow-up demonstrated stabilization of fragility fractures and symptom resolution over 6–12 months.

DISCUSSION:

This case series highlights scurvy as an underrecognized cause of pathological fractures in pediatric patients with severe food selectivity, often associated with ASD. Skeletal complications can occur in the absence of classic systemic and mucocutaneous features, contributing to diagnostic delay. These cases underscore the importance of including scurvy, an easily treatable condition, in the differential diagnosis of vertebral and lower extremity fractures in at-risk populations.

### Disclosure

None declared

### P24

Abstract withdrawn

### P25

### Vertebral Fractures Presenting with Back Pain as the First Symptom of Underlying Metabolic Bone Disease in Children

MD Stavroula-Eirini Gantzoula, MD Elpiniki- Maria Parlitsi, MD Polyxeni Barouni, MSc. Ioannis Platanitis, MD Ioannis Karnezis, Ph.D Artemisia Panou, Ph.D Ioanna Paspati

Orthopedic Deparment, Penteli's Children's Hospital, Athens, Greece

### Abstract

OBJECTIVES:

To show that vertebral compression fractures (VCFs) presenting with back pain and no or low energy trauma may be the first sign of underlying bone metabolism disease or a concurrent finding on the basis of a genetic disorder. To highlight the role of orthopedic in early diagnosis and to increase awareness of diseases potentially debilitating if left undiagnosed.

METHODS:

This review summarizes pediatric cases presented in our Orthopedic Department with back pain as sole symptom and history of no or low energy trauma. Imaging depicted VCFs while further bone metabolism and genetic evaluation revealed a metabolic bone disease or a genetic disorder with bone involvement.

RESULTS:

We demonstrate nine pediatric patients presented with back pain on outpatient basis, the careful evaluation of whom revealed VCFs as first sign of underlying bone metabolism diseases. In the table are depicted details of age, sex, history of trauma, imaging and genetic results. Five patients were found with specific mutations related to bone metabolism disorders, one patient had negative genetic examination, one demonstrated VCFs due to autoimmune hepatitis treated with corticosteroids, and two presented with VCFs on the basis of genetic disorders. They were treated with bracing or casting controlling pain and spine deformity. All but one were treated with intravenous or oral bisphosphonates, with subsequent remodeling of vertebral shape.

CONCLUSION:

Vertebral compression fractures in children with no or low energy trauma, presenting with back pain, are an important indication of underlying metabolic bone diseases or a concurrent finding with other genetic disorders. Thus, the role of the orthopedic in early recognition, bone metabolism testing and genetic evaluation is critical. It also enables the initiation of appropriate treatment and prevents skeletal abnormalities in the future.

Table. Pediatric patients presented with back pain with no or low trauma history were found to have VCFs leading to the diagnosis of an underlying metabolic bone disease or a genetic disorder with bone involvement. *No treatment with bisphosphonates.

| Case | Age at examination time | Sex | Vertebral fracture | Trauma | Imaging | Spine BMD Z-score | Metabolic/ genetic disorder |
|---|---|---|---|---|---|---|---|
| 1 Twin with case 2 | 13 | M | L2, L3 | Low energy | X-ray, CT | -1.6 | Mutation c.3107G>A, p.Arg1036Gln in LRP5 gene.Mutation c.1645C>G, p.Pro549Ala in COL1A2 gene |
| 2 | 13 | M | T4,T5, T6 | None | X-ray CT | -2.3 | Mutation c.3107G>A, p.Arg1036Gln in LRP5 gene.Mutation c.1645C>G, p.Pro549Ala in COL1A2 gene |
| 3 | 7 | F | Multiple | None | X-ray | -3.1 | Polymorphism Sp1 in Col1A1 geneMutation p.V667M in LRP5 genePolymorphism Bsm1 in VDR gene |
| 4 | 13 | F | T8, T9, T10, T11 | None | X-ray CT, MRI | -3.7 | Autoimmune hepatitis/ corticosteroids |
| 5 | 14 | M | Multiple | Low energy | X-ray CT | -2.3 | Negative genetic examination |
| 6 | 13 | M | T11, T12, L1 | Low energy | X-ray | -1.42 | Single nucleotide variant in SLC9A3R1 geneSingle nucleotide variant in PDE11A gene |
| 7* | 12 | M | T4 | Low energy | X-ray CT | -0.3 | Nucleotide deletion c.1667delC in COL1A1 gene |

| | | | | | | | |
|---|---|---|---|---|---|---|---|
| 8 | 14 | M | T3, T4, T5, T6, T7, T8 | Low energy | X-ray<br>CT | -3.2 | Retinitis pigmentosa<br>Negative genetic examination for metabolic bone disease |
| 9 | 12 | M | Multiple | None | X-ray | | Pseudoglioma-osteoporosis syndrome |

**Disclosure**

None declared

### P26

### Zoledronic Acid Use in the UK - A Scoping Exercise into the Experiences of Centres Across the UK

Emma Snow, Robyn Gilbey Cross

Evelina London, London, UK

### Abstract

**Objective:**

Anecdotally nurses from the UK Paediatric Rare Bone Nurse Network (RBNN) noted significant disparity across all aspects of zoledronic acid treatment. The objective was to identify differences in practice, and if possible the evidence base for these. The long term objective is to generate a UK consensus for zoledronic acid treatment in paediatrics for the UK.

**Method:**

A mixed multiple-choice and free text questionnaire was sent electronically to all members of the RBNN and endocrine nurse members of the British Society of Paediatric Endocrinology and Diabetes. Questions covered all aspects of the patient journey from pre-infusion, infusion to post-infusion.

**Results:**

17 centres responded, 12 of which were tertiary referral centres.

Results confirmed large differences in practice, but similar barriers to patients receiving timely infusions.

Three main themes emerged; patient safety, resource utilisation and patient experience.

Patient safety:

Bloods required prior to first and subsequent infusions differed in type of blood test required and timeframe before infusion. Vitamin D testing and supplementation had large variability. Only 72% of sites required a pregnancy test despite zoledronic acid being teratogenic. There was an even split of centres that advocated for and against the use of Ibuprofen post-infusion.

For patients with known or presumed adrenal insufficiency the guidance around sick day dosing varied from PRN to for 72 hours after every infusion.

A majority of sites advocated the use of calcium supplementation post infusion but dosing varied drastically.

Resource Management:

28% of sites required all patients to stay overnight for the first infusion, 33% never required an overnight stay and at 38% of sites this was dependent on co-morbidities.

11% of sites required a renal ultrasound prior to treatment.

Patient experience:

Barriers to timely infusions were commonly cited as lack of bed availability, travel, uncertainty about treatment and blood test practicalities, all of which can be impacted by these differences in practice.

**Conclusions:**

Although there were limitations to this initial scoping survey, it clearly presents a disparity in practice across the UK, with many citing a poor evidence base as to the reason behind this. It is crucial to work towards a consensus to improve patient safety, resource utilisation and patient experience.

### Disclosure

None declared

### P27

### From Panic to Preparedness: How Emergency Splint Kits Improve Fracture Care in Osteogenesis Imperfecta

Lori VandenBroek, Nadia Narducci, Argerie Tsimicalis

Shriners Hospital for Children - Canada, Montreal, Canada

### Abstract

**Background:**
Osteogenesis imperfecta (OI) is a genetic disorder characterized by increased bone fragility, low bone mass, and other connective tissue manifestations[1]. Individuals with severe OI may experience multiple fractures annually from birth through adulthood. These fractures range from minor microfractures to severe fractures necessitating surgical intervention. Proper fracture management is essential for pain control, optimal bone healing, reduction of complications, and restoration of function and mobility.

In 2019, Shriners Hospitals for Children–Canada (Shriners), in collaboration with one of our OI patients, introduced a fracture splint kit. This kit contains the tools, materials, and educational resources required to appropriately immobilize a fracture during an emergency[2]. Approximately 130 fracture kits have been distributed to OI patients at the Shriners, ensuring immediate access to supplies when needed. Families are educated upon receiving their fracture kits by the OI nursing care team.

**Objective:**
Since the implementation of the splint kits, we opted to evaluate the kits via a quality improvement initiative to understand the perceived usefulness, and impact on the autonomy and quality of life of the patients and their families.

**Methods:**
Over a four-month period patients with OI or their family members were invited to complete an anonymous questionnaire while attending a clinic visit at Shriners. Data were descriptively analyzed.

**Results:**
To date 20 patients with OI or their family members responded. Overall, they expressed a significant perceived usefulness of the kit, which has become an essential item for the immobilization of fractures. Patients and families' responses were overwhelmingly positive, highlighting increased confidence, reassurance, and improved fracture management at home. The families also reported a reduction in anxiety during fracture emergencies.

**Conclusion**

This initiative improved patient and family autonomy, confidence, and perceived quality of life while promoting safer early fracture management. The program shows strong potential for expansion to other osteogenesis imperfecta care settings.

### Disclosure none declared

### P28

### Fatal Pulmonary Hypertension in an Infant with Severe Osteogenesis Imperfecta

Janet Legare, Neil Paloian, Blaise Nemeth, Robert Steiner, Vivek Balasubramaniam

University of Wisconsin, Madison, USA

### Abstract

BBACKGROUND: Osteogenesis imperfecta (OI) is associated with cardiopulmonary complications related to abnormal type I collagen, a structural component of airways and pulmonary vasculature. Lethal OI (type II) usually results in stillbirth or neonatal death from pulmonary insufficiency due to a markedly small thoracic cage. However, cardiopulmonary dysfunction may also occur independent of skeletal features, as demonstrated by bone-independent restrictive lung disease in adults with OI. We describe an infant with a pathogenic *COL1A1* variant and normal thoracic circumference who developed rapidly progressive pulmonary hypertension (PH) and died at 5 months of age.

PRESENTING PROBLEM: The mother was referred at 25 4/7 weeks gestation for suspected life-limiting skeletal dysplasia. Prenatal evaluation confirmed OI due to a pathogenic *COL1A1* variant (c.1777G>A, p.Gly593Ser), which has been associated with variable but generally severe phenotypes.

CLINICAL MANAGEMENT: The infant was born at 36 5/7 weeks and required non-invasive respiratory support for 48 hours, weaning to room air by day of life five. Skeletal survey showed fractures of all long bones and poor mineralization without evidence of pulmonary hypoplasia (normal thoracic size). He was diagnosed with severe OI, between types II and III, and discharged at five weeks of age with enteral tube feeds and a normal echocardiogram.

Between 2 and 4.5 months, he had multiple admissions for hypoxemia attributed to possible micro-aspiration and required continuous supplemental oxygen by three months. Echocardiography at that time showed normal cardiac anatomy with mildly elevated pulmonary artery pressure (31 mmHg). At five months, he presented with severe hypoxemic respiratory failure requiring mechanical ventilation and was found to have suprasystemic PH with estimated pressures of 92 mmHg (Figure 1). After discussion of prognosis, life-sustaining therapy was withdrawn and the patient expired.

DISCUSSION: Pulmonary hypertension has not previously been reported in pediatric OI. This case demonstrates that severe, rapidly progressive PH can develop postnatally in infants with OI despite an adequately sized thoracic cage. Abnormal extracellular matrix integrity and impaired pulmonary vascular development due to defective type I collagen may underlie this phenotype. Early recognition of PH in severe OI may have important prognostic and therapeutic implications and warrants further investigation.

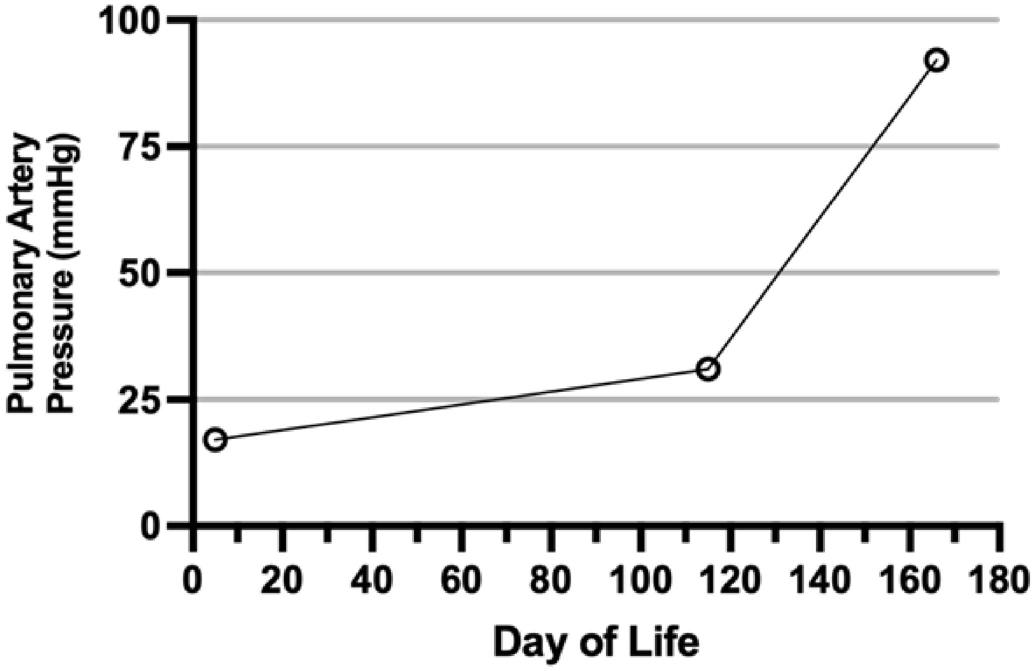

Figure 1

**Disclosure**

None declared

### P29

### Reducing Inpatient Fracture Rate for Patients at Increased Risk Through Quality Improvement Methodology, Automated Electronic Alerts, and an Agentic AI System

Alexander Chesover[1], Lucy Turriff[1], Haiqah Amjad[2], Johanna Andersson[1], Kevin Jones[1], Mark Heathfield[1], Andrew Turnbull[1], Sebin Sebu[3], Pavithra Rajendran[3], Shiren Patel[3], Nuwanthi Yapa Mahathanthila[1]

[1]Great Ormond Street Hospital for Children, London, UK. [2]University College London Medical School, London, UK. [3]DRIVE, Great Ormond Street Hospital for Children NHS Foundation Trust, London, UK

### Abstract

### OBJECTIVES

Our patient safety team highlighted concern about inpatient fracture incidence, and that some fractures may be unrecognised and preventable. The quality improvement (QI) team led a project that aimed to decrease inpatient fractures in patients at increased risk. We identified three primary drivers: (1) identification of risks, (2) alerting high risk, and (3) and staff education.

### METHODS

The methodology was grounded in the Institute for Healthcare Improvement's Model for Improvement. There was stakeholder engagement across multiple specialities, disciplines, and wards, and collaboration with our electronic patient record (EPR), data analyst, and data research teams.

To identify and alert fracture risk, we built an EPR alert banner (see figure), automatically triggered by extractable patient data variables. This was iteratively developed, informed by validation cycles and staff feedback.

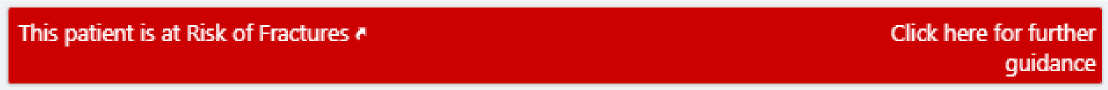

**Figure 1.** Alert Banner that automatically triggers in electronic health record for patients at increased risk of fracture.

We utilised surveys and semi-structure interviews across different professional disciplines to identify staff education needs.

The manual review of radiology reports to measure inpatient fracture rate was unfeasible. We developed an agentic AI system to process unstructured radiology report text and identify fractures.

### RESULTS

Alert banner validation reduced flagging inpatients at risk of fracture from 44% to 8% of all inpatients. Staff feedback showed improved awareness and confidence in the care of patients at risk of fracture. The EPR alert banner is now implemented Trust-wide.

Survey and interview data (84 and 12 participants, respectively) highlighted the need for standardised guidance. We have now linked a leaflet on patient handling to the alert banner and published a Trust guideline on reducing risk of inpatient fractures.

The AI system autonomously outputs inpatients with new fractures. We prioritised the system's sensitivity to avoid missing inpatient fractures. A monthly AI-generated report is reviewed within 15 minutes, compared with the first manual review of 1332 reports over 2 days.

**CONCLUSIONS**

Through QI methodology, interdisciplinary stakeholder engagement, and innovative collaborations we have successfully implemented (1) an automated EPR alert system, (2) new guidance, and (3) an agentic AI system, aiming to reduce inpatient fractures. Our interventions increased staff awareness and confidence. The project has now moved to business as usual for the Trust with ongoing monitoring of its effectiveness.

### Disclosure

None declared

### P30

### When Late Effects are not the Whole Story: PLS3-Related Osteoporosis in a Childhood Cancer Survivor

Danielle Eddy, Ian Tucker, Elizabeth Crowne, Toby Candler, Manju Chandwani

Bristol Children's Hospital, Bristol, UK

### Abstract

**Background**

Childhood-onset osteoporosis is rare but clinically significant. Pathogenic variants in the X-linked *PLS3* gene, encoding plastin-3, are an uncommon cause of primary early-onset osteoporosis. Plastin-3 is widely expressed in solid tissues and plays a role in cytoskeletal remodelling. We report a male adolescent with severe early-onset osteoporosis who was treated for acute lymphoblastic leukaemia (ALL) in early childhood and later found to have a pathogenic *PLS3* variant.

**Presenting Problem**

A 16-year-old male was re-referred to a tertiary paediatric endocrine service after low bone mineral density (BMD) was identified on dual-energy X-ray absorptiometry (DXA). He had a history of ALL, diagnosed at 2 years of age and treated with the UKALL 2003 Regimen A, without radiotherapy.

He had been followed from 9 years of age in an endocrine late-effects clinic for short stature and delayed puberty, with a bone age delay of 1.5 years. Growth hormone stimulation testing at 13 years showed an adequate peak GH level (33.9 µg/L). Pubertal induction with testosterone was commenced at 14 years and discontinued at 15 years following satisfactory pubertal progression.

At 16 years, he was re-referred due to recurrent long-bone fractures. His fracture history included five fractures between 3 and 4 years of age, initially attributed to ALL, and a further two low-trauma long-bone fractures at 15 years. Comorbidities included focal epilepsy treated with levetiracetam, mild pulmonary stenosis, and learning difficulties.

DXA demonstrated a total body less head BMD Z-score of −4.9 and a lumbar spine bone mineral apparent density Z-score of −2.0, with no vertebral fractures. His height was 162.1 cm (−2.0 SDS) and BMI-SDS +2. Evaluation excluded common causes of secondary osteoporosis, and treatment-related bone disease was considered less likely given the long interval since cancer therapy. Genetic testing identified a truncating variant in the *PLS3* gene.

**Clinical Management**

Management included optimisation of vitamin D status followed by intravenous bisphosphonate therapy with zoledronic acid.

**Discussion**

This case highlights the difficulty of distinguishing treatment-related bone disease from monogenic osteoporosis in childhood cancer survivors. While chemotherapy exposure and endocrine sequelae may reduce bone mass, the severity and early onset of osteoporosis in this patient were disproportionate. Identification of a pathogenic *PLS3* variant emphasises the importance of considering genetic causes of osteoporosis in patients with marked skeletal fragility, even in the presence of significant acquired risk factors.

### Disclosure

None declared

### P31

### Assessing the Impact of Short Stature on Paediatric Patients with Osteogenesis Imperfecta using the Brief Quality of Life in Short Stature Youth (QoLISSY-Brief)

Mark Heathfield, Zoe Bowman Bayles, Emilie Hupin, Agueda Moreno Lopez, Belinda Crowe, Catherine DeVile, Moira Cheung

Osteogenesis Imperfecta Service, Great Ormond Street Hospital for Children NHS Foundation Trust, London, UK

### Abstract

**Background/Objectives:**

Osteogenesis Imperfecta (OI) is associated with short stature (SS) but there are few data on the impact of SS on health-related quality of life (HrQoL). The aim of the study was to assess HrQoL in patients and parents from a large paediatric OI centre using the Brief Quality of Life in Short Stature Youth (QoLISSY-Brief).

**Methods:**

Children with a *COL1A1* or *COL1A2* gene variant aged 8-18 years with a height Z score <-2 were identified over a 2-year period. Parents and children were asked to complete the QoLLISY-B questionnaire. Exclusion criteria included inability/difficulty completing the questionnaire and patients who did not respond or were not contactable.

**Results:**

From a cohort of 302 children, 41 were identified (20 female/21 male, 13.58% of total population) with a mean age of 12.9 (3.0 SD) years. 10 patients were excluded: 2 deceased, 2 due to learning difficulties, 6 uncontactable. The distribution across clinical types was: OI-I 43% (N=18), OI-III 17% (N=7), OI-IV 37% (N=16). Of those eligible, 22 child and 23 parent questionnaires were completed (response rate 73%). There was significantly reduced HrQoL with the Total QoLISSY-Brief mean (SD) scores of child 59.9 (15.0) and parent 53.7 (17).

Table 1.

The scores for all three domains were low with the physical impairment scoring the lowest but the emotional toll of this also being important. There were no significant differences between child and parent total or subscale scores (all p > .05).

**Conclusions:**

The impact of SS can be easily overlooked by clinicians. Using the QoLISSY-Brief, our results suggest impacts on quality of life across physical, social and emotional domains with the physical domain being the area of most significance. These results highlight the importance of considering SS in clinical practice. Results also show consistency between parent and child reporting across all domains suggesting parents appeared to have a good understanding of their child’s strengths and difficulties. Further study on the impact of short stature in relation to clinical presentation, age and sex, as well as across other OI genotypes is recommended.

Table 1

| Domain | Item | Short Label | Child - Mean (SD) | Parents - Mean (SD) | p-value |
|---|---|---|---|---|---|
| Physical | Q1 | Physical | 3.1 (2.0) | 2.7 (0.9) | 0.14 |

| | | | | | |
|---|---|---|---|---|---|
| | | limitations | | | |
| | Q2 | Reach difficulties | 2.0 (1.0) | 2.0 (1.0) | 1.00 |
| | Q3 | Depend on others | 2.6 (1.5) | 2.8 (1.2) | 0.64 |
| Social | Q4 | Feeling small | 2.0 (1.2) | 2.4 (1.2) | 0.34 |
| | Q5 | Laughed at /teased | 4.0 (0.9) | 3.4 (1.1) | 0.09 |
| | Q6 | Being treated differently | 3.7 (1.1) | 2.7 (1.3) | 0.01 |
| Emotional | Q7 | Feel different | 3.3 (1.2) | 2.7 (1.3) | 0.15 |
| | Q8 | Insecure | 2.3 (1.3) | 2.9 (1.3) | 0.36 |
| | Q9 | Bothers me | 2.9 (1.3) | 2.7 (1.4) | 0.61 |
| Total | QoLISSY Brief (0-100) | | 59.9 (15.0) | 53.7 (17.0) | |

*Table 1 Values are reported as median (interquartile range). Individual items are scored on a 5-point Likert scale (1 = extremely, 5 =never). The total QoLISSY Brief score was calculated by summing item scores (maximum 45) and linearly transforming the result to a 0–100 scale (score/45 × 100), with higher scores indicating better quality of life. Comparisons between patient and parent-proxy responses were performed using the t-test. Items Q1–Q9 correspond to the QoLISSY Brief form (2015) with permission and licensing from IQVIA. License number QUO-06615-J5N4N6.*

### Disclosure

None declared

### P32

### When Bones aren't the Only Problem: Botulinum Toxin as a Novel Adjunctive Therapy for Knee Flexion Contractures in Osteogenesis Imperfecta Type V

Nadia Narducci, Lori VandenBroek, Reggie Hamdy, Corinne Mercier, Frank Rauch, Camille Costa

Shriners, Montreal, Canada

### Abstract

Osteogenesis Imperfecta (OI) is a rare heritable connective tissue disorder characterized by bone fragility and marked clinical heterogeneity. OI type V, caused by a recurrent pathogenic variant in *IFITM5*, represents a phenotypically distinct subtype classically associated with radial head dislocation, interosseous membrane calcification, metaphyseal banding, and hyperplastic callus formation. Joint contractures are not a recognized feature of this subtype, and bilateral knee flexion contractures are exceptionally rare. Among more than 40 individuals with OI type V followed at Shriners Hospital for Children–Canada, this is the first documented case with knee involvement, underscoring the rarity and clinical significance of this presentation.

We describe a 15-year-old boy with genetically confirmed osteogenesis imperfecta (OI) type V who experienced an unexpectedly rapid and debilitating decline in mobility. Over a short period, he developed progressive bilateral knee flexion contractures reaching 70–80 degrees of flexion, culminating in the loss of independent ambulation. Comprehensive evaluation—including laboratory studies, advanced imaging, and exome sequencing—did not reveal an alternative neuromuscular, inflammatory, or genetic cause beyond the known *IFITM5* mutation. These findings suggest that severe progressive knee contractures may represent a rare and underrecognized manifestation of OI type V.

Management required a coordinated multidisciplinary approach involving physiatry, orthopedics, and intensive physiotherapy delivered both in clinic and through a structured home program. In the context of minimal response to standard conservative measures and challenges with treatment adherence, intramuscular botulinum toxin injections were implemented as a deliberate and innovative adjunctive intervention. To our knowledge, this represents the first reported use of botulinum toxin to treat knee flexion contractures in OI type V. Following treatment, the patient demonstrated clear, clinically meaningful improvements in knee extension and functional mobility, highlighting the therapeutic impact of neuromuscular modulation in this setting.

This case identifies a previously undocumented therapeutic application of botulinum toxin in OI type V and establishes a novel management paradigm for refractory knee flexion contractures in this rare disorder. These findings support consideration of botulinum toxin as a targeted adjunctive therapy and warrant further investigation to define its role in preserving mobility and improving quality of life in patients with rare musculoskeletal conditions.

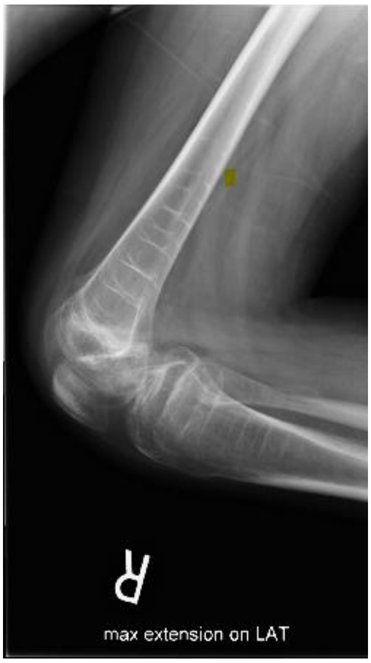

**Disclosure**

none

### P33

### A Longitudinal Study of Bone Mineral Density During Liraglutide-Induced Weight Loss in an Adolescent with Myelomeningocele

Carin Skogastierna[1,2], Lovisa Sjögren[1], Diana Swolin-Eide[1,2]

[1]Department of Pediatrics, Sahlgrenska University Hospital, Region Västra Götaland, Gothenburg, Sweden. [2]Department of Pediatrics, Institute for Clinical Sciences, Sahlgrenska Academy, University of Gothenburg, Gothenburg, Sweden

### Abstract

**Background:** Childhood obesity is increasingly prevalent and poses major health challenges, particularly in children with physical disabilities. Glucagon-like peptide-1 (GLP-1) receptor agonists have emerged as effective treatments for pediatric obesity, but concerns remain regarding potential adverse effects on bone mass, especially during growth and in patients with pre-existing skeletal fragility. Evidence on bone health outcomes during GLP-1–induced weight loss in children is limited.

**Presenting problem:** A 17-year-old girl with myelomeningocele and osteoporosis was increasingly disabled because of her raising weight. She had a history of six low-trauma fractures, longstanding reduced mobility, and obesity. At seven years of age, when vitamin D supplementation and yearly DXA assessments were initialized, she had BMI 21.1 kg/m$^2$, TBLH Z-score -0.4, and total fat mass 49.7%. At age 12 years, she had BMI 30.7 kg/m$^2$, TBLH Z-score -1.1, and total fat mass 61.6%. At age 17 years (pre-treatment), she had BMI 32.9 kg/m$^2$, TBLH Z-score -1.1, and total fat mass 60.1%. Given her skeletal vulnerability, there was concern that pharmacologically induced weight loss could further compromise bone mineral density.

**Clinical management:** The patient was managed by a multidisciplinary team with expertise in pediatric endocrinology and obesity. Liraglutide therapy was introduced and titrated according to clinical guidelines, alongside individualized lifestyle support. DXA measurements were repeated at 12 and 24 months to monitor bone mineral density and body composition.

Over 24 months of treatment, the patient lost 22 kg and achieved a BMI of 24.6 kg/m$^2$. TBLH Z-score was -1.4 and total fat mass was 54.7%. Serial DXA assessments demonstrated no clinically significant decline in bone mineral density during follow-up. No new fractures occurred. In addition to metabolic improvement, the patient experienced increased mobility, greater functional independence, and increased quality of life.

**Discussion:** This case suggests that liraglutide can be an effective and “bone-safe” weight-loss intervention in adolescents with complex medical conditions and pre-existing osteoporosis when carefully monitored. The findings underscore the importance of individualized treatment and regular skeletal surveillance when managing obesity. pharmacologically in vulnerable pediatric populations. Larger studies are needed to clarify the long-term skeletal effects of GLP-1 receptor agonists in children.

### Disclosure

None declared

### P34

### Early-Onset Medication-Related Osteonecrosis of the Jaw in Duchenne Muscular Dystrophy: First Cases in Adolescents on Antiresorptives

Agathe Rio[1,2], Patricia Olivier[3], Mélanie Dupuis[1], Coralie Leblicq[1], Nathalie Alos[1,4], Melissa Fiscaletti[1,4]

[1]Sainte-Justine Hospital, Montreal, Canada. [2]Université de Montréal, Montréal, Canada. [3]Sainte-Justine Hospital, Montréal, Canada. [4]Azrieli Research Center of the Sainte-Justine University Hospital, Montreal, Canada

### Abstract

BACKGROUND:

Youth with Duchenne muscular dystrophy (DMD) have the highest prevalence of acquired osteoporosis in pediatric populations due to the progressive muscle weakness and chronic glucocorticoid (GC) therapy. Current guidelines recommend routine screening for vertebral fractures to detect the earliest signs of bone fragility. As a result, antiresorptives are initiated at much younger ages than in previous decades.

PRESENTING PROBLEM:

Medication-related osteonecrosis of the jaw (MRONJ) is a well-established complication of antiresorptive therapy in adults but is exceedingly rare in children, and no cases have been reported in youth with DMD.

CLINICAL MANAGEMENT:

We describe two adolescents with DMD who developed MRONJ following dental extraction during antiresorptive therapy for GC-induced osteoporosis. Clinical presentation, management and outcomes are summarized. A focused review was conducted to contextualize these observations within current pediatric data on antiresorptive treatment related osteonecrosis. Case 1: A 16-year-old on long-term deflazacort began intravenous bisphosphonate therapy at age 10 for vertebral compression fractures (zoledronic acid 0.05mg/kg 6-monthly). MRONJ developed at age 15 after dental extraction and six months after his last zoledronic acid infusion. Case 2: An 18-year-old receiving bisphosphonates from age 8 years (zoledronic acid 0.05 mg/kg 6 monthly, adjusted for ideal weight), was later switched to denosumab (1 mg/kg SC 6-monthly) at age 18 years. MRONJ occurred six months after the transition and following tooth extraction. In both cases, antiresorptives were suspended and conservative management led to complete healing of MRONJ within three months. Antiresorptive therapy was later resumed for pain and fracture prevention, and no recurrence of MRONJ was noted after one-year follow-up.

DISCUSSION:

Review of more than 1,000 pediatric patients treated with bisphosphonates or denosumab identified no prior reports of MRONJ in children with DMD. To our knowledge, this is the first and youngest reported cases of MRONJ in adolescents with DMD. They highlight a potential vulnerability of neuromuscular patients with prolonged GC exposure and long-term antiresorptive therapy. Although the absolute risk of MRONJ in children remains extremely low, systematic dental evaluation before antiresorptive initiation and close multidisciplinary follow-up, especially around dental extraction, are essential to minimize preventable complications.

### Disclosure

None declared

### P35

### Biological and Behavioral Predictors of Bone Turnover Markers in Children and Adolescents

Hyunwoo Kim[1], Leanne M Ward[2,3], Lehana Thabane[1], Alexandra Papaioannou[1,4], Jonathan D Adachi[1], Jinhui Ma[1], Frank Rauch[5,6]

[1]McMaster University, Hamilton, Canada. [2]University of Ottawa, Ottawa, Canada. [3]Children's Hospital of Eastern Ontario, Ottawa, Canada. [4]Geras Centre for Aging Research, Hamilton, Canada. [5]McGill University, Montreal, Canada. [6]Shriners Hospital for Children, Montreal, Canada

### Abstract

**Background**: Procollagen type 1 N-terminal propeptide (P1NP) and C-terminal telopeptide of type 1 collagen (CTX) are dynamic indicators of skeletal metabolism. However, evidence remains limited regarding their biological and behavioral correlates across developmental stages.

**Methods**: We analyzed serum levels of P1NP in 3,386 and CTX in 1,530 individuals aged 6 to 20 years who participated in the Canadian Health Measures Survey (Cycles 5–6, 2016–2019). Weighted linear regression models examined associations between age- and sex-specific z-scores of P1NP and CTX and obesity status, physical activity, parathyroid hormone (PTH), serum 25-hydroxyvitamin D [25(OH)D3] status, and high-sensitivity C-reactive protein (hs-CRP). Subgroup analyses were conducted using sex-specific age bands as proxies for pubertal stages to assess heterogeneity.

**Results**: Body mass index-based overweight and obesity were associated with lower P1NP z-scores (overweight: −0.320, $p<0.001$; obesity: −0.378, $p<0.001$, compared to normal & underweight) and lower CTX z-scores (overweight: −0.378, $p<0.001$; obesity: −0.278, $p=0.048$). Analyses by age bands showed that obesity-related differences were greatest during pubertal ages. For example, during puberty, obesity was associated with markedly lower P1NP z-scores in both males (−0.825, $p<0.001$) and females (−0.786, $p<0.001$), and lower CTX z-scores (males: −0.857, $p=0.005$; females: −0.676, $p=0.008$). These associations were more persistent among males into the post-pubertal stage (CTX in obese children: −0.836, $p<0.001$; P1NP in obese children: −0.527, $p=0.004$). Systemic inflammation showed inverse associations with bone turnover markers, particularly among females: high hs-CRP (>3 mg/L) was associated with lower P1NP overall (−0.577, $p<0.001$) and lower CTX among females (−0.589, $p=0.001$), whereas the corresponding association was not evident in males. PTH was positively associated with both P1NP (0.117 per pmol/L, $p<0.001$) and CTX (0.103 per pmol/L, $p=0.006$) z-scores across analyses. Physical activity showed modest associations in pubertal age group, whereas 25(OH)D3 status was not associated with either marker in unadjusted or adjusted models.

**Conclusions**: Puberty represents a period when obesity and systemic inflammation are most strongly reflected in bone turnover markers, with notable sex differences, especially for CTX. These findings support interpreting pediatric bone turnover markers in relation to pubertal-related age group, sex, and metabolic context.

### Disclosure

Dr. Papaioannou reports a past consulting relationship with Amgen, which ended more than two years ago (as of 2024). No other conflicts of interest are declared.

### P36

### IGF-1 Restores Primary Cilia Architecture and Microtubule Polyglutamylation in AIS Osteoblasts

Pardis Behzadi, Mohamed Elbakry, Alain Moreau, Stefan Parent, Florina Moldovan

Research Center CHU Sainte Justine, Université de Montréal, Montreal, Canada

### Abstract

**Introduction:**

Adolescent idiopathic scoliosis (AIS) is a multifactorial spinal disorder whose progression is closely associated with the rapid growth occurring during puberty. Among key hormonal drivers of this growth, insulin-like growth factor 1 (IGF-1) has been implicated in AIS progression. Emerging evidence points to primary cilia dysfunction and altered microtubule post-translational modifications—particularly polyglutamylation—as potential contributors to scoliosis pathogenesis. This study aimed to determine how IGF-1 influences primary cilia architecture and microtubule polyglutamylation in osteoblasts derived from AIS patients.

**Methods:**

Primary osteoblasts isolated from spinal tissue of AIS patients were cultured and exposed to graded concentrations of IGF-1. Immunofluorescence was used to quantify cilia length, orientation, tubulin content, and levels of microtubule polyglutamylation.

**Results:**

AIS osteoblasts exhibited shorter primary cilia and altered microtubule organization. IGF-1 exposure significantly increased cilia length and enhanced both ciliary tubulin expression and polyglutamylation in a dose-dependent manner. These findings link IGF-1 signaling to restoration of structural features that are typically impaired in AIS cells.

**Conclusion:**

IGF-1 promotes elongation of primary cilia and stimulates microtubule polyglutamylation in AIS osteoblasts, highlighting its key role in ciliary microtubule organization. These results shed new light on how pubertal growth factors may modulate cellular mechanisms underlying AIS and suggest potential therapeutic avenues targeting ciliary dysfunction.

Supported by Yves Cotrel Foundation - Institut de France

### Disclosure

None declared

### P37

### Assessment of Bone Metabolism in Children With SMA

Wojciech Stelmach, Anna Fabiś- Strobin, Krzysztof Małecki

Polish Mother's Memorial Hospital Research Institute, Łódź, Poland

### Abstract

#### Objectives:

Before the introduction of gene therapy, patients suffering from SMA often died in early childhood. While the therapy not only gives hope to patients, it also creates new challenges for physicians. One of these challenges is the development of osteopenia and osteoporosis in pediatric patients. The aim of this study is a comprehensive assessment of bone metabolism in children with SMA.

#### Methods:

The study included 22 patients aged 3 to 17 years (mean age 8.6 years) with SMA. In 17 patients, laboratory tests were performed to assess: albumin levels, Beta CrossLaps, ALP, phosphate levels, magnesium levels, total calcium and ionized calcium levels, sodium levels, osteocalcin levels, PTH levels, and vitamin D3 levels. Additionally, bone density was assessed using densitometry in 16 patients.

#### Results:

The mean ion levels were as follows: phosphate 1.7 mmol/L; magnesium 0.88 mmol/L; sodium 138 mmol/L; total calcium 2.49 mmol/L; ionized calcium 1.25 mmol/L. No statistically significant differences were observed between ambulatory and non-ambulatory patients.

Markers of bone metabolism were: Beta CrossLaps 1052 pg/mL; ALP 159.78 IU/L; osteocalcin 66.69 ng/mL. The mean parathyroid hormone level was 22.9 pg/mL, and the mean vitamin D3 level was 38.3 ng/mL. In two patients, vitamin D3 levels were suboptimal (>30 ng/mL).

The mean BMD Z-score in the studied population was −2.9: −1.49 in ambulatory patients and −3.91 in non-ambulatory patients.

#### Conclusions:

Assessment of bone metabolism in patients with SMA is particularly important for preventing the development of osteopenia and, consequently, for reducing the risk of low-energy fractures. The significant difference in bone density between ambulatory and non-ambulatory patients highlights the crucial importance of verticalization and weight-bearing in patients with SMA.

### Disclosure

None declared.

### P38

### Pre-Existing Enthesopathy in a Child with X-linked Hypophosphatemic Rickets: Clinical Course After Initiation of Burosumab

Stavroula-Eirini Gantzoula, Elpiniki- Maria Parlitsi, Polyxeni Barouni, Ioannis Karnezis, Ioannis Platanitis, Artemisia Panou, Ioanna Paspati

Orthopedic Department, Penteli's Children's Hospital, Athens, Greece

### Abstract

### Background

X-linked hypophosphatemic rickets (XLH) is a rare inherited disorder characterized by excessive fibroblast growth factor 23 (FGF23) activity, resulting in chronic hypophosphatemia and impaired bone mineralization. Clinical manifestations include rickets, skeletal deformities, and musculoskeletal pain. Enthesopathy refers to pathological changes at the enthesis, the site of tendon or ligament attachment to bone. In XLH, enthesopathy is thought to result from defective mineralization combined with increased mechanical stress secondary to skeletal deformities and altered biomechanics. Although enthesopathy is common in adults with XLH, it is infrequently reported in pediatric patients, and data regarding its prevalence and clinical course in children are limited.

### Case Presentation

We report a 6-year-old girl with genetically confirmed XLH who developed localized musculoskeletal pain consistent with enthesopathy prior to the initiation of burosumab therapy. The patient had been receiving conventional treatment with oral phosphate supplementation and alfacalcidol without adequate improvement in serum phosphate levels. Physical examination revealed localized tenderness at the pes anserinus insertion, with no features suggestive of inflammatory arthritis. Plain radiographs demonstrated bilateral knee enthesopathy (Figure).

Burosumab therapy was initiated according to standard pediatric dosing guidelines and subsequently adjusted based on biochemical response. Following treatment initiation, serum phosphate levels improved. During follow-up, the patient experienced no recurrence or progression of enthesopathy-related symptoms and reported no limitations in daily activities. Anti-inflammatory medication was not required.

### Conclusion

This case demonstrates that enthesopathy may occur in pediatric patients with XLH despite conventional therapy for hypophosphatemia. The absence of recurrent or progressive entheseal symptoms following burosumab initiation suggests that early targeted treatment may stabilize musculoskeletal involvement. Early correction of mineralization defects and healing of rickets in children with XLH may reduce abnormal mechanical stress at the tendon–bone interface, potentially limiting progressive entheseal and other skeletal structural changes during growth.

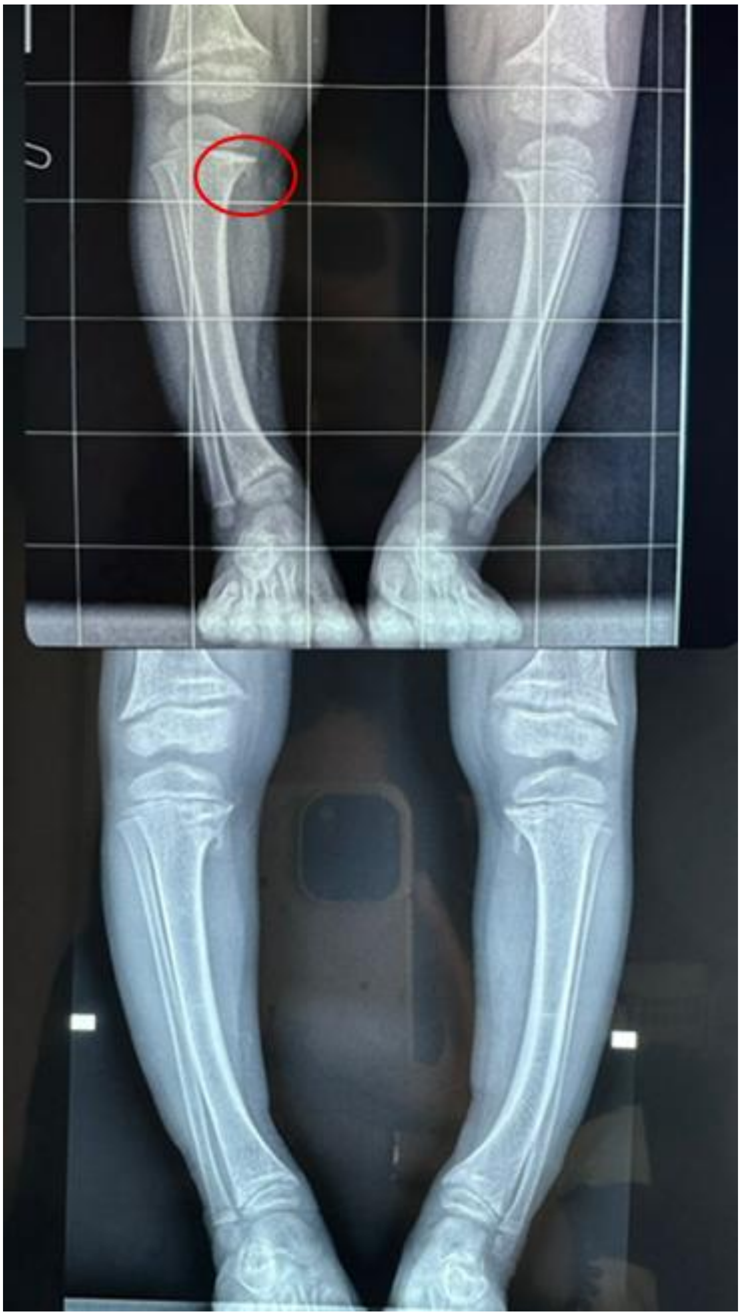

**Figure.** As shown in the top of the image, there are radiographic findings of rickets, as long as incipient enthesopathy (noted in circle). That time the child was under conventional treatment. By comparison, the bottom image illustrates established enthesopathy. We notice improved radiographic appearance of rickets. The patient had been in burosumab treatment for 7 months.

### Disclosure

None declared

### P39

### PHEX Gene Alteration as a Cause of Hypophosphatemic Rickets in Pediatrics: Case Reports

Liliana Mejia de Beldjenna[1,2,3], Jorge Endo[1,2], Maria Valentina Mejia Santamaria[4], Jose David Mejia Z[5]

[1]Foundation Clinic infantil Club Noel, Cali, Colombia. [2]UNILIBRE.GRINPED, Cali, Colombia. [3]Colombian College of Pediatric Endocrinology Association, Bogota, Colombia. [4]University of Buenos aires, Buenos Aires, Argentina. [5]ICESI, Cali, Colombia

### Abstract

BACKGROUND:

X-linked hypophosphatemic rickets (XLR) is a mineral homeostasis disorder caused by renal phosphate wasting. It is the most common form of hereditary hypophosphatemic rickets. Caused by a loss-of-function variant in the PHEX gene located at Xp22.1. X-linked autosomal dominant inheritance. Leads to an isolated tubular defect in phosphate reabsorption and decreased intestinal absorption of phosphate.

Clinical manifestations include gait delay, waddling gait, bowing of the legs, widening of the metaphyses of the wrists and ankles, bone pain, craniostenosis, dental abscesses, lower limb involvement (coxa vara, genu valgum, genu varum) and disproportionate short stature.

The therapy aims to counteract the consequences of excess FGF23, using oral phosphorus supplements to compensate for renal phosphate wasting and active vitamin D analogs to counteract 1,25-diOH-vitamin D deficiency. New therapies with FGF23 monoclonal antibodies that neutralize FGF23 activity have proven effective.

PRESENTING PROBLEM:

We report three patients, two males and one female, with: short stature, genu varum, hypophosphatemia, low tubular reabsorption of phosphates (<75%), low 25-OH, elevated alkaline phosphatase, and the PEHX gene alteration , as were their mothers.

CLINICAL MANAGEMENT:

They received treatment with fibroblast growth factor blocking antibody 23 due to lack of response to conventional management, with an excellent response.

DISCUSION:

Phosphate-wasting kidney disorders are important causes of rickets that must be considered within the appropriate clinical context. Correct identification of these disorders is important for determining treatment. Treatment with fibroblast growth factor blocking antibody 23 is a great hope

### Disclosure

None declared

### P40

### Vitamin D Metabolomic Profiling in Infants with Histological Signs of Rickets at the Growth Plate Post-Mortem

Suma Uday[1,2,3], Martin Hewison[3], Carl Jenkinson[3], Fozia Shaheen[3], Marta Cohen[4], Tamas Marton[1], Wolfgang Högler[5,3]

[1]Birmingham Children's Hospital, Birmingham, UK. [2]Narayana Health City, Bengaluru, India. [3]University of Birmingham, Birmingham, UK. [4]Sheffield Children's Hospital, Sheffield, UK. [5]Johannes Kepler University, Linz, Austria

### Abstract

**Background/ Objectives:**

The Vitamin D metabolome involves multiple circulating metabolites beyond total 25-hydroxyvitamin D (25OHD), including from activation to 1,25-dihydroxyvitamin D, catabolism via 24,25-dihydroxyvitamin D ($24,25(OH)_2D$), and C3-epimerisation of 25OHD. While $24,25(OH)_2D$ has been implicated in growth plate regulation in animal models, the biological relevance of the epimers in human skeletal development remains poorly understood. We investigated vitamin D metabolome in children with and without histological rickets at autopsy.

**Methods:**

Children undergoing autopsy following sudden unexpected death (between 2017–2022) were recruited with parental consent and regulatory approvals. Those with known bone-affecting conditions or medications were excluded. Post-mortem blood samples (3–7 days) were analysed for vitamin D metabolites using LC–MS/MS at a DEQAS accredited laboratory. Metabolites measured included total 25OHD ($25OHD_3$+$25OHD_2$), 3-epi-$25OHD_3$, $24,25(OH)_2D_3$, and $1,25(OH)_2D_3$. Vitamin D metabolite ratio (VMR; $25OHD_3/24,25(OH)_2D_3$) and the percentage of epimerised $25OHD_3$ (3-epi $25OHD_3/25OHD_3$ *100) were calculated. Rib growth plates were assessed for histological signs of rickets (as defined in **Table 1)** and scored independently by two raters.

**Results:**

Twenty-three children [median age of 83 days, 83% (19/23) infants, 16 males] were included; 15 had histological rickets and 8 had normal growth plates. 25OHD<30 nmol/L (deficiency) was recorded in 37.5% (n=9/24). Inter-rater reliability for histology was high (mean $\kappa = 0.79$). Total 25OHD, $24,25(OH)_2D_3$, and VMR did not differ between groups (all $p>0.05$) **Table 1**. VMR was incalculable in two subjects with rickets and undetectable $24,25(OH)_2D_3$. Serum $1,25(OH)_2D_3$ levels were undetectable, owing to its very short half-life. The percentage of 3-epi-$25OHD_3$ was significantly higher in children with rickets ($p < 0.05$). Total 25OHD correlated strongly with $24,25(OH)_2D_3$ ($r = 0.8$, $p<0.001$), and epimer proportion was inversely associated with age ($r = -0.5$, $p=0.02$).

**Conclusions:**

The higher epimer proportion observed in infants with histological rickets, despite similar total 25OHD, supports the current notion that epimers are biologically active with less calcaemic potency. Our findings suggest that vitamin D epimers may have a biologically relevant role in growth plate physiology and rickets pathogenesis. Further mechanistic studies are underway to define the functional significance of epimers in skeletal biology.

| Measure | Statistic | Total cohort (n=23) | [&]Rickets (n=15) | No rickets (n=8) | P value |
|---|---|---|---|---|---|

| | | | | | |
|---|---|---|---|---|---|
| Total 25OHD (nmol/L) | Mean (SD) | 31.5 (20.4) | 32.5 (25.7) | 34.2 (7.8) | 0.4 |
| | Median (IQR) | 31.7 (7, 46.3) | 42.2 (5.7, 51.8) | 32.6 (31, 41.8) | |
| $25OHD_3$ (nmol/L) | Mean (SD) | 31.15 (20.5) | 32.2 (25.8) | 33.7 (7.8) | 0.4 |
| | Median (IQR) | 31.11 (7, 46.0) | 42.2 (4.8, 51.7) | 32.0 (30.3, 41.4) | |
| 3-epi-$25OHD_3$ (nmol/L) | Mean (SD) | 3.7 (2.8) | 4.2 (3.4) | 3.2 (1.7) | 0.4 |
| | Median (IQR) | 3.2 (1.4, 6.1)) | 3.5 (0.8, 7.3) | 3.2 (1.7, 4.2) | |
| $24,25(OH)_2D_3$ (nmol/L) * | Mean (SD) | 2.4 (2.02) | 2.6 (2.5) | 2.6 (1.5) | 0.4 |
| | Median (IQR) | 2.1 (1.0, 3.0) | 2.2 (0.7, 3.8) | 2.2 (1.7, 3.3) | |
| Vitamin D Metabolite Ratio (VMR) * | Mean (SD) | 16.2 (8.4) | 17.1 (9.6) | 15.5 (6.9) | 0.3 |
| | Median (IQR) | 13.8 (12.1, 20.7) | 15.9 (12.1, 21.0) | 13.2 (12.4, 17.8) | |
| Percentage of 3-epimer | Mean (SD) | 14.2 (7.6) | 15.4 (5.4) | 9.8 (6.0) | **0.004** |
| | Median (IQR) | 13.1 (8.6, 19.4) | 14.9 (9.7, 19.5) | 8.0 (5.7, 16.3) | |

[&] Rickets was defined as the presence of two or more features on a 6 point score 1=increased height of hypertrophic zone, 2=Loss of columnar arrangement of hypertrophic chondrocytes with disarray, 3=loss of distinction between hypertrophic and proliferative zones, 4=blood vessels penetrating the chondrocytes, 5=tongues of hypertrophic chondrocytes extending into primary spongiosa and 6=bulbous expansion of the costo-chondral junction.

*$24,25(OH)_2D_3$ was undetectable in two subjects with very low 25OHD which precluded VMR assessment.

### Disclosure

None declared

### P41

### Trabecular Bone Score in Children with Osteopetrosis: TBS Osteo Advanced for Pediatrics (Version 4)

Julie Briody[1], Anna Walstra[2], Kevin London[1], Madeleine Davies[2], Didier Hans[2,3], Karen Hind[2,4], Craig Munns[5]

[1]The Children's Hospital at Westmead, Sydney, Australia. [2]Medimaps Group SA, Geneva, Switzerland. [3]Center of Bone Diseases, Rheumatology Unit, Bone and Joint Department, Lausanne University Hospital & University of Lausanne, Lausanne, Switzerland. [4]Faculty of Health and Medicine, Lancaster University, Lancaster, UK. [5]The University of Queensland, Brisbane, Australia

### Abstract

OBJECTIVES:

Osteopetrosis is a rare genetic disorder characterised by impaired osteoclastic bone resorption, resulting in increased bone mass with paradoxical skeletal fragility. Dual-energy X-ray absorptiometry (DXA) is routinely used to quantify elevated bone mineral density (BMD) in affected children; however, BMD alone does not fully characterise the abnormal skeletal phenotype. Trabecular bone score (TBS), a DXA-derived index reflecting trabecular texture and structural complexity, may provide complementary information on disease-related characteristics. This study evaluated TBS in children with osteopetrosis and examined its potential role as a marker of disease-specific trabecular pathology.

METHODS:

Children with osteopetrosis referred for clinically indicated DXA at a tertiary paediatric hospital were included. Lumbar spine DXA scans were analysed using TBS Osteo Advanced for Pediatrics (Version 4). Patients outside the validated age range (5-20 years) or with abdominal soft tissue thickness outside the manufacturer-recommended limits (7.0-30.0 cm) were excluded. TBS Z-scores were derived using sex- and age-matched reference data and analysed separately by sex.

RESULTS:

A total of 48 patients with osteopetrosis were included, comprising 8 females (age 9.97 ± 4.4 y; 100% White; BMI 21.8 ± 7.5 kg/m$^2$; tissue thickness 16.7 ± 4.9 cm) and 40 males (age 10.9 ± 3.4 y; 90.0% White, 10.0% Asian; BMI 18.7 ± 3.8 kg/m$^2$; tissue thickness 15.6 ± 3.0 cm). TBS Z-scores were elevated in both sexes, with higher values observed in males. The mean TBS Z-score was 1.30 ± 1.91 in female patients and 5.35 ± 2.20 in male patients. TBS Z-scores greater than 2 were observed in 90% of male patients.

CONCLUSIONS:

Children with osteopetrosis demonstrate markedly elevated TBS Z-scores, particularly in males. These elevated values likely reflect disease-related trabecular accumulation and impaired skeletal remodeling inherent to osteopetrosis rather than improved bone quality. When interpreted alongside DXA-derived BMD, TBS may serve as a complementary marker of the distinctive trabecular phenotype associated with this condition.

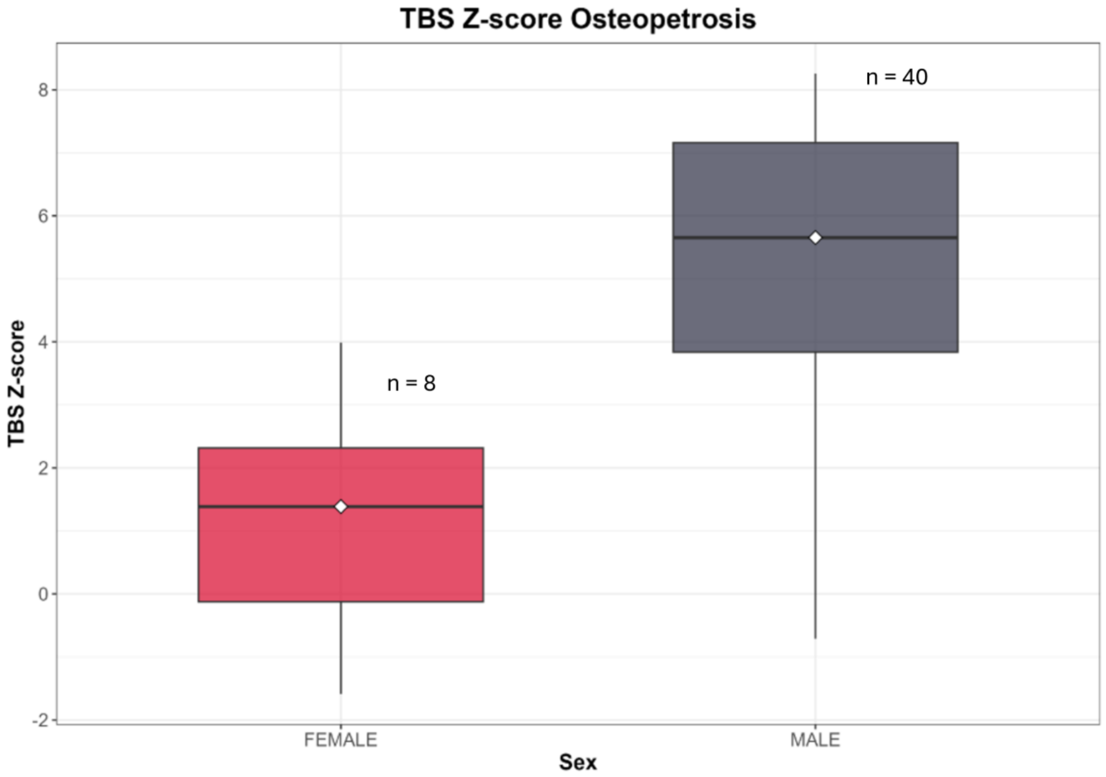

***Figure** 1 Boxplot of Trabecular Bone Score (TBS) in children with osteopetrosis.*

### Disclosure

JB, KL and CM declare they have no conflicts of interest. AW, MD and KH are employees of Medimaps Group SA, developers of TBS iNsight™ software. DH is co-owner of the TBS patent, has corresponding shares, and is part-time CEO at Medimaps Group.

### P42

### Trabecular Bone Score in Children with Duchenne Muscular Dystrophy: TBS Osteo Advanced for Pediatrics (Version 4)

Julie Briody[1], Anna Walstra[2], Kevin London[1], Madeleine Davies[2], Didier Hans[2,3], Karen Hind[2,4], Craig Munns[5]

[1]The Children's Hospital at Westmead, Sydney, Australia. [2]Medimaps Group SA, Geneva, Switzerland. [3]Center of Bone Diseases, Rheumatology Unit, Bone and Joint Department, Lausanne University Hospital & University of Lausanne, Lausanne, Switzerland. [4]Faculty of Health and Medicine, Lancaster University, Lancaster, UK. [5]The University of Queensland, Brisbane, Australia

### Abstract

OBJECTIVES:

Duchenne Muscular Dystrophy (DMD) is the most common form of muscular dystrophy and is associated with progressive muscle degeneration and secondary skeletal deterioration. Reduced mobility, muscle weakness, and prolonged glucocorticoid exposure contribute to impaired bone health and increased fracture risk in this population. Bone mineral density (BMD) is routinely used to assess skeletal status in children with DMD; however, BMD alone may not fully capture disease- and treatment-related changes in bone microarchitecture. Trabecular bone score (TBS), a DXA-derived, validated index of bone microarchitecture, may provide additional insight into skeletal alterations associated with DMD and glucocorticoid exposure. This study evaluated TBS in a pediatric male cohort with DMD relative to an age-matched normative reference population.

METHODS:

Male patients with DMD referred for clinically indicated DXA at a tertiary children's hospital were included. Lumbar spine DXA scans were analysed using TBS Osteo Advanced for Pediatrics (Version 4). Patients outside the validated age range (5-20 years) or with abdominal soft tissue thickness outside the manufacturer-recommended limits (7.0-30.0 cm) were excluded. TBS Z-scores were derived relative to an age-matched reference population. One-sided statistical testing assessed whether TBS Z-scores in patients with DMD were lower than reference values.

RESULTS:

245 males with DMD were included (age 12.2±3.5 years; 71.4% White; BMI 23.9 kg/m²; abdominal soft tissue thickness 18.9±4.2 cm). TBS Z-scores were significantly lower than those of the age-matched reference population (–0.94±1.34; $p<0.001$). Overall, 23.3% (n = 57) of patients had TBS Z-scores ≤-2.0, and a further 23.3% (n = 57) had Z-scores between -2.0 and -1.0. An additional 29.0% (n = 71) had TBS Z-scores between -1.0 and 0.0, while 24.5% (n = 60) had Z-scores >0.0.

CONCLUSIONS:

Boys with DMD demonstrate significantly reduced TBS Z-scores relative to age-matched reference data, consistent with the known skeletal consequences of reduced mechanical loading, progressive muscle loss, and long-term glucocorticoid exposure. These findings are aligned with the expected bone phenotype in DMD and support TBS as a complementary DXA-derived measure to characterise disease-related impairment of trabecular microarchitecture. When interpreted alongside BMD, TBS may help better define the extent and severity of skeletal involvement in this population.

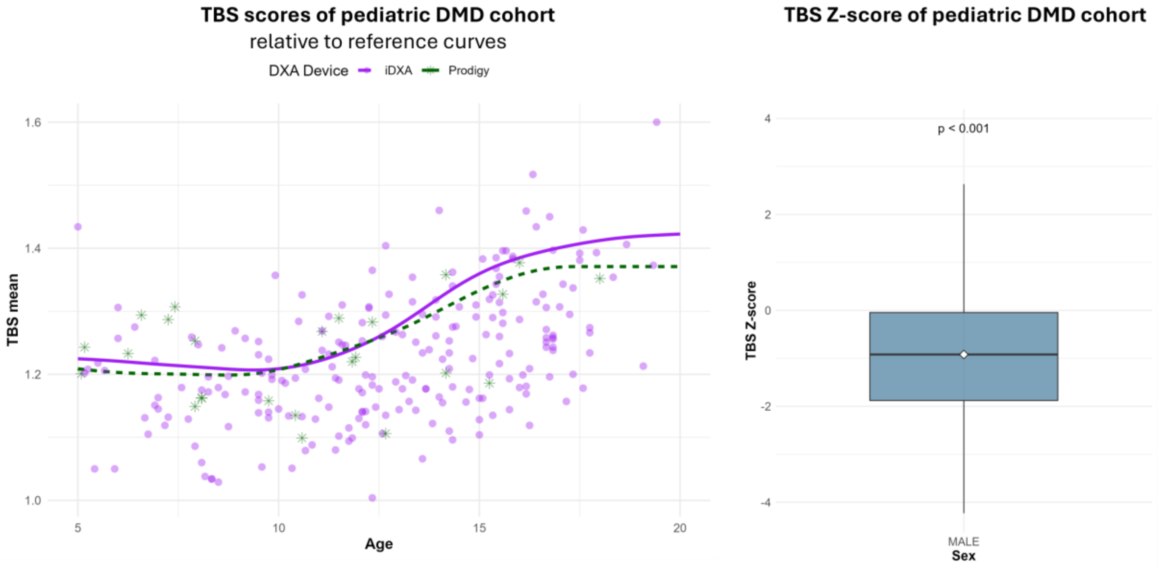

***Figure 1*** *Trabecular Bone Score (TBS) values of children with Duchenne muscular dystrophy (DMD) (data points) plotted against the reference curves (purple/green lines). Corresponding TBS Z-scores (right panel) significantly lower than reference population (p < 0.001).*

### Disclosure

JB, KL and CM declare they have no conflicts of interest. AW, MD and KH are employees of Medimaps Group SA, developers of TBS iNsight™ software. DH is co-owner of the TBS patent, has corresponding shares, and is part-time CEO at Medimaps Group.

### P43

### Trabecular Bone Score in Children with Thalassemia: TBS Osteo Advanced for Pediatrics (Version 4)

Julie Briody[1], Anna Walstra[2], Kevin London[1], Madeleine Davies[2], Didier Hans[2,3], Karen Hind[2,4], Craig Munns[5]

[1]The Children's Hospital at Westmead, Sydney, Australia. [2]Medimaps Group SA, Geneva, Switzerland. [3]Center of Bone Diseases, Rheumatology Unit, Bone and Joint Department, Lausanne University Hospital & University of Lausanne, Lausanne, Switzerland. [4]Faculty of Health and Medicine, Lancaster University, Lancaster, UK. [5]The University of Queensland, Brisbane, Australia

### Abstract

OBJECTIVES:

Thalassemia is a group of inherited hemoglobin disorders caused by reduced or absent β-globin chain synthesis, commonly requiring long-term blood transfusion therapy. While transfusions improve survival, associated complications contribute to impaired bone mineralization, resulting in skeletal deformities, bone pain, cortical thinning, and increased fracture risk in affected children. Dual-energy X-ray absorptiometry (DXA)-derived bone mineral density (BMD) is routinely used to assess bone health in thalassemia but does not capture bone structure or microarchitectural alterations. Trabecular bone score (TBS), a DXA-derived index of bone microarchitecture. This study evaluated TBS in paediatric patients with thalassemia relative to age- and sex-matched reference populations.

METHODS:

Children with thalassemia referred for clinically indicated DXA at a tertiary children's hospital were included. Lumbar spine DXA scans were analysed using TBS Osteo Advanced for Pediatrics (Version 4). Patients outside the validated age range (5–20 years) or with abdominal soft tissue thickness outside the manufacturer-recommended limits (7.0–30.0 cm) were excluded. TBS Z-scores were derived relative to sex- and age-matched reference data.

RESULTS:

175 patients were included: 92 boys (age 14.6 ± 2.8 y; 40.2% White, 32.6% Asian; BMI 18.7 ± 3.8 kg/m²; tissue thickness 16.3 ± 2.5 cm) and 83 girls (age 13.0 ± 3.1 y; 48.2% White; BMI 19.6 ± 3.7 kg/m²; tissue thickness 16.2 ± 2.3 cm). TBS Z-scores were significantly lower than reference values in both boys (-1.07 ± 1.07; $p < 0.001$) and girls (-1.21 ± 1.33; $p < 0.001$). Among boys, 17.4% had TBS Z-scores ≤ –2.0 and 42.4% between –2 and –1, while only 15.2% had Z-scores > 0.0. Among girls, 20.5% had TBS Z-scores ≤ –2.0, 37.3% between –2.0 and –1.0, and 19.3% had Z-scores > 0.0.

CONCLUSIONS:

Children with thalassemia demonstrate significantly reduced TBS Z-scores relative to age- and sex-matched reference data, consistent with altered trabecular bone microarchitecture. The majority of patients had TBS Z-scores below –1.0, with a notable proportion below –2.0, particularly among girls. TBS may provide valuable insights into disease-related skeletal involvement and support a more comprehensive assessment of bone health in this high-risk pediatric population.

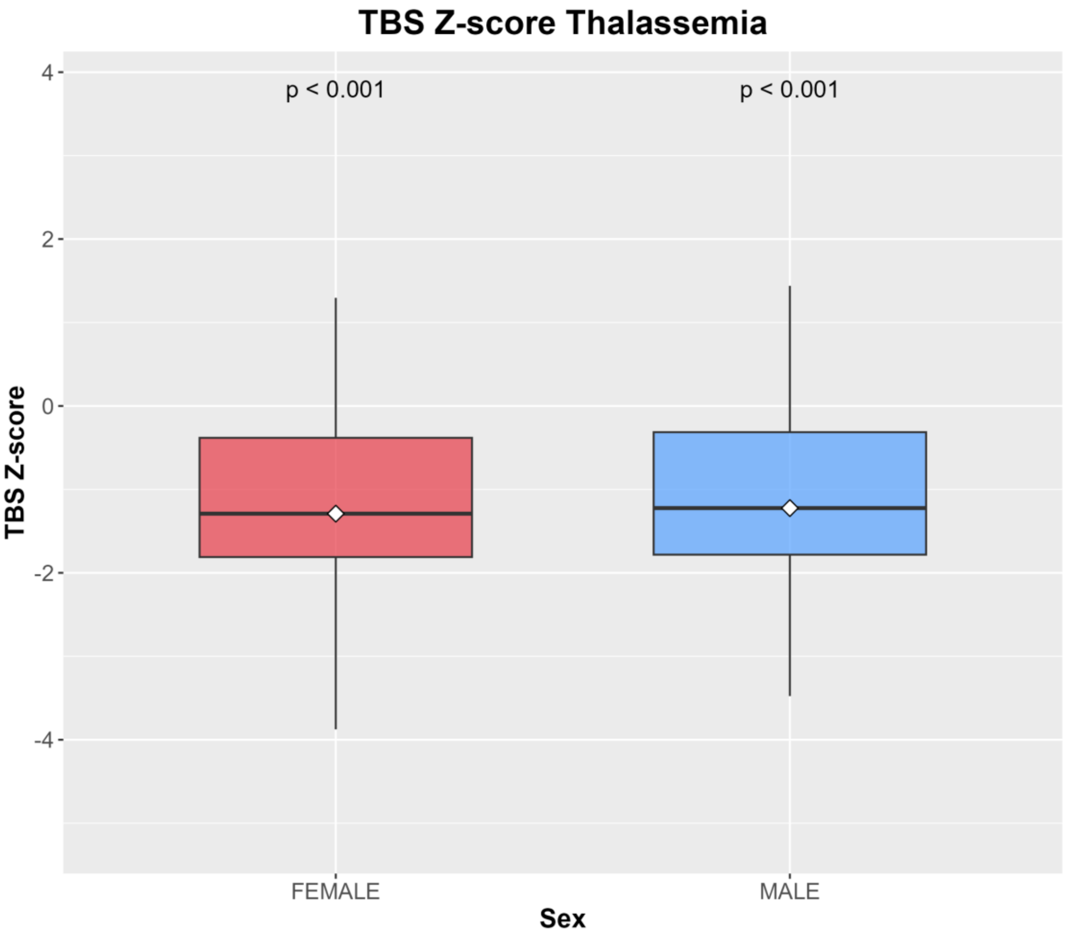

***Figure** 1 Trabecular Bone Score (TBS) in a pediatric population with Thalassemia, stratified by sex. Relative to the normative population, TBS Z-score is significantly lower for both sexes ($p < 0.001$).*

### Disclosure

JB, KL and CM declare they have no conflicts of interest. AW, MD and KH are employees of Medimaps Group SA, developers of TBS iNsight™ software. DH is co-owner of the TBS patent, has corresponding shares, and is part-time CEO at Medimaps Group.

### P44

### The Use of 18F-Choline PET-CT as a Superior Diagnostic Tool in Paediatric Patients with Hyperparathyroidism

Louise Apperley[1], Charlotte Jarvis[1], Suzanne Armitage[1], Nagabhushan Seshadri[2], Samantha Goh[1], Neil Houghton[2], Renuka Ramakrishnan[1]

[1]Alder Hey Children's Hospital, Liverpool, UK. [2]Royal Liverpool University Hospital, Liverpool, UK

### Abstract

### Introduction

Hyperparathyroidism has multiple aetiologies, and failure to identify the cause may lead to significant complications including hypercalcaemia, hypophosphataemia, renal stones, osteoporosis and impaired graft function in renal transplant recipients. Accurate diagnostic investigations are essential to optimise management and enable minimally invasive surgery where appropriate.

### Case 1

An 8-year-old male with a background of left kidney dysplasia (stable renal function) had an incidental finding of hypercalcaemia. He did not respond to first line treatment, so was commenced on Cinacalcet, which normalised calcium levels. A 99mTc-MIBI scan identified possible increased uptake in the left submandibular gland, but no parathyroid lesions. Magnetic Resonance Imaging (MRI) scan reported no abnormal soft tissue lesions in this area and neck ultrasonography showed a suspicious area, which was inconclusive. An 18F-Choline Positron Emission Tomography-Computed Tomography (PET-CT) later confirmed a left parathyroid adenoma posterior to the lower pole of the left thyroid lobe.

### Case 2

A 10-year-old male with autism spectrum disorder was referred with hypercalcaemia secondary to hyperparathyroidism. He had a background of bilateral renal dysplasia and successful renal transplant at 3 years of age. Hypercalcaemia causing impaired renal function persisted despite hyperhydration and low-calcium diet, necessitating treatment with Cinacalcet. Neck ultrasonography did not identify parathyroid masses. Further investigation to exclude a parathyroid adenoma was required, but due to his neurodivergence, MRI and a 99mTc-MIBI scans were unsuitable because of prolonged imaging time and limited sensitivity. 18F-Choline PET-CT was successfully performed under a short general anaesthetic, demonstrating bilateral parathyroid adenoma. Surgical management has been discussed and is under consideration.

### Discussion

Localising parathyroid adenomas in children can be challenging. Choline PET-CT scans have high sensitivity and positive predictive value for identifying parathyroid adenomas. This enables more precise localisation than other currently used imaging modalities, resulting in more efficient, minimally invasive parathyroidectomy.

Choline PET-CT imaging is only available at limited locations. Challenges include patient transfer and the management of paediatric patients in adult hospitals, requiring additional staff and specialised equipment as well as logistics of radiopharmaceutical supply. If these barriers are addressed and availability improves, 18F-Choline PET-CT may become an earlier investigation of choice in such patients.

Figure 1 shows the images and findings for Case 1 (top) and Case 2 (bottom)

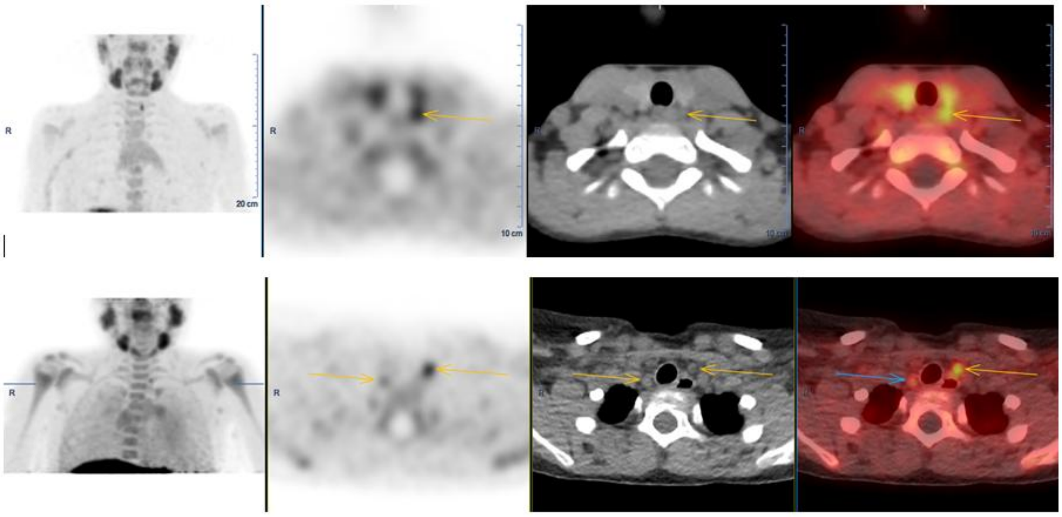

### Disclosure

None declared

### P45

### Relative Cortical Density: A Novel pQCT Measure of Cortical Homogeneity?

Julie Briody[1,2], Craig Munns[3]

[1]University of Sydney, Sydney, Australia. [2]The Children's Hospital at Westmead, Westmead, Australia. [3]The University of Queensland, Brisbane, Australia

### Abstract

**Objectives:** Cortical "defects", regions of reduced density within the cortical shell, are frequently observed on our pQCT scans (~8%) but are not reported in the pQCT literature, despite evidence that reduced cortical homogeneity compromises bone strength. We aimed to quantify cortical defects using a novel measure of density homogeneity - Relative Cortical Density (RCD), describe RCD in a healthy paediatric population, and demonstrate clinical application.

**Methods:** pQCT scans from research and clinical controls (4-18yo; Radius 65%-site, Tibia 66%-site) were analysed using Stratec software (v6.00B). RCD was defined as the proportion of cortical voxels ≥710mg/cm³. Comparative 'Distribution analysis' using a modified ImageJ pQCT plugin generated a peeled RCD ($RCD_P$), reducing the partial volume effect by edge voxel removal. Automatic and manual analysis precision (coefficients of variation (CV), least significant change (LSC)) were assessed. Sex-specific reference curves and Z-scores were generated using the Cole's LMS method and clinical utility examined in cohorts with a history of ≥2 forearm fractures, or untreated osteogenesis imperfecta (OI) type 1.

**Results**

A total of 317 Radius (150 female) and 316 Tibia (162 female) scans were analysed. RCD was significantly age-associated (see example Figure 1). Precision was lowest in manually analysed, poorer-quality Radius scans (CV 0.94% [RCD], 0.68% [$RCD_P$]; LSC 0.018 and 0.016); all other CVs were ≤0.14%.

Subjects with historical forearm fractures (N=47) had reduced RCD and $RCD_P$ despite a normal cortical density (Z-scores= -1.05, -1.18 and -0.13, respectively, p<0.001). In contrast, OI subjects (N=46) demonstrated elevated $RCD_P$ and cortical density (Z-scores=1.03, p<0.002).

**Conclusions:** The age-related increases in RCD are consistent with histomorphometric reports of age-related reductions in drifting osteons, remodelling spaces, and cortical porosity, but may also reflect a higher density.

Defects or increased porosity in bone may elevate fracture risk by enhancing crack propagation and increasing the remodelling surface. Although individual pores are below the resolution of clinical pQCT, their cumulative effect reduces voxel density and is captured by RCD. By capturing cortical heterogeneity related to macroporosity and mineralisation, RCD may improve paediatric fracture risk assessment and monitoring of bone disease beyond conventional pQCT.

Figure 1: LMS models of RCD and $RCD_P$ (Radius)

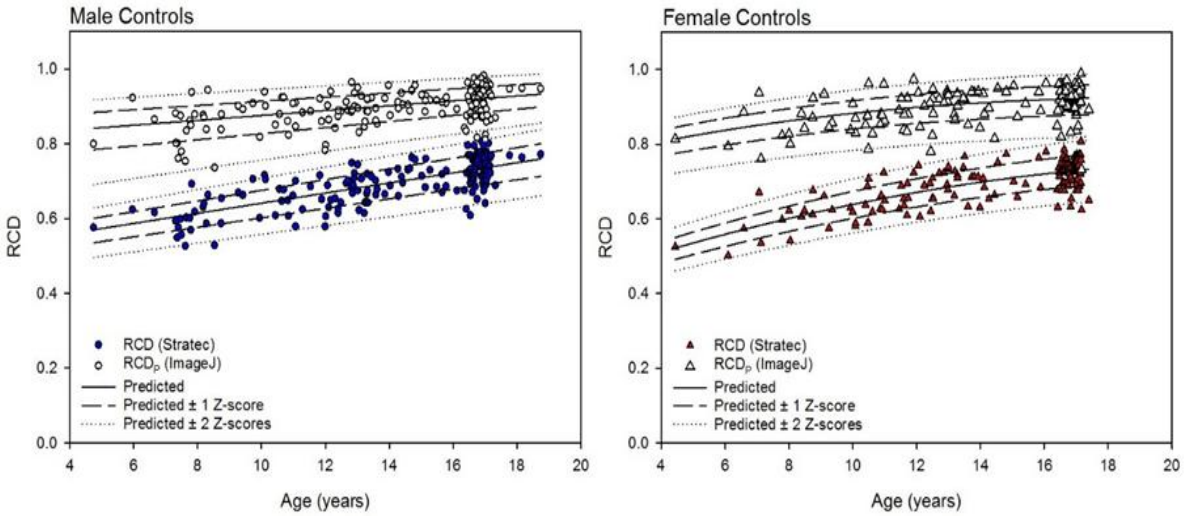

### Disclosure

None declared

### P46

### Progressive Vertebral Height Loss Is a Significant Marker for Early Initiation of Bisphosphonates Treatment in Boys with Duchenne Muscular Dystrophy (DMD) Receiving Long-Term Corticosteroid Therapy

Nicola Crabtree[1,2], Sarah McCarrison[3,4], Vrinda Saraff[1,2], Sheila Shepherd[3,4], Suma Uday[1,2], Claire Wood[5], Sze Choong Wong[3,4]

[1]Birmingham Women's and Children's NHS Trust, Birmingham, UK. [2]Institute of Metabolism and Systems Research, University of Birmingham, Birmingham, UK. [3]Royal Hospital for Children, Glasgow, UK. [4]Human Nutrition, University of Glasgow, Glasgow, UK. [5]Royal Victoria Infirmary, Newcastle University, Newcastle, UK

### Abstract

**Objectives:**

Vertebral fractures (VF) are a recognised complication in boys with Duchenne muscular dystrophy (DMD) receiving corticosteroid therapy. Intravenous (IV) bisphosphonates reduce fracture progression and improve pain. Current guidelines recommend treatment following identification of moderate or severe VF, or any VF associated with back pain. This study aimed to identify early imaging markers prior to identification of VF to support earlier, targeted intervention.

**Methods:**

This dual-centre retrospective study included boys with DMD on IV bisphosphonates for VF identified through spine monitoring between 2015 and 2025. Inclusion criteria were spine imaging immediately prior to bisphosphonate initiation and at least one lateral spine image within the preceding 12 months. Annual spine monitoring was performed using lateral dual-energy X-ray absorptiometry or lateral spine radiographs. All images were centrally reviewed by a single observer. Vertebral height loss (VHL) was graded using modified Genant scoring system: 10–20% (height loss; HL), 20–25% (mild VF), 25–40% (moderate VF), and >40% (severe VF). Spine deformity index (SDI) was calculated by assigning scores of 0.5, 1, 2, and 3 for HL, mild, moderate, and severe VF, respectively.

**Results:**

Forty-eight boys with DMD receiving corticosteroids were included. A total of 195 spine images were reviewed, comprising 2,535 vertebrae (T4–L4), with a mean of 4 (range 2–7) images per participant. Overall, 254/2,535 (10%) vertebral fractures were identified: 148 mild, 92 moderate, and 14 severe. Comparison with prior imaging demonstrated that 63% of mild and moderate VF and all severe VF were preceded by detectable 10–20% VHL. Only 10% of VF were incident, while 27% remained stable during follow-up. Progression of VHL was significantly associated with higher SDI 12 months prior to treatment initiation (SDI 4.7 [3.6] vs 2.1 [2.1] in those without prior VHL; $p<0.001$). SDI at 12 months prior also strongly predicted SDI at treatment initiation ($p<0.001$) (figure 1).

**Conclusion:**

Progressive VHL and increasing SDI strongly predict future VF risk and support earlier initiation of bisphosphonate therapy, even before overt VF development. This challenges current care recommendations but could help prevent severe, painful VF cascade often seen in boys with DMD receiving corticosteroids.

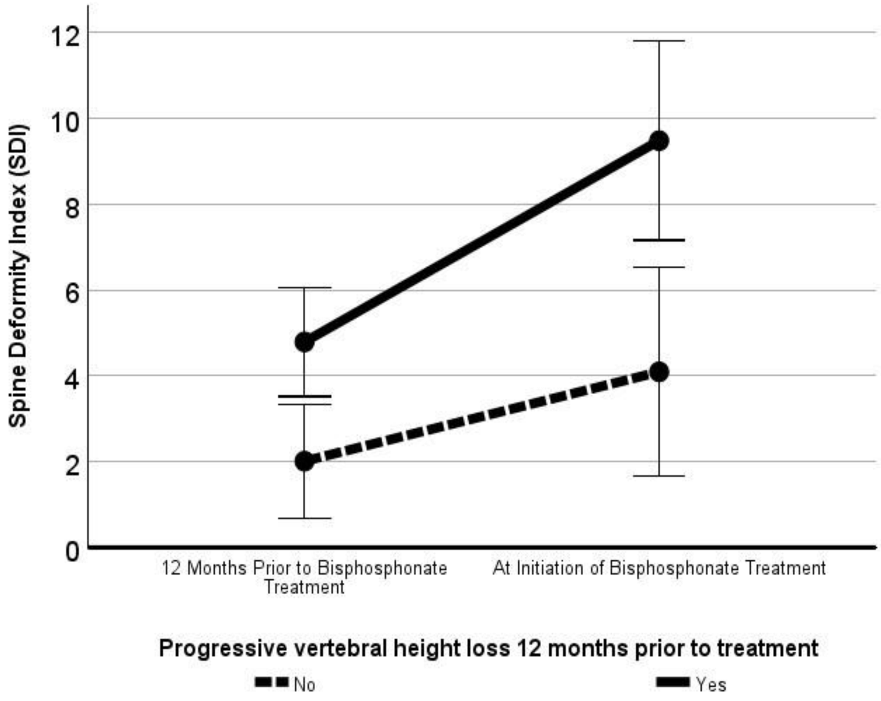

### Disclosure

None

**P47**

Abstract withdrawn

### P48

### Radiofrequency Echographic Multi-Spectrometry (REMS) for Bone Health Assessment in Pediatric Populations

Carla Caffarelli[1], Caterina Mondillo[1], Alessandro Versienti[2], Guido Cavati[1], Antonella Al Refaie[2], Sara Gonnelli[3], Paola Pisani[4]

[1]Section of Internal Medicine, Department of Medicine, Surgery and Neuroscience, University of Siena, Siena, Italy. [2]1. Section of Internal Medicine, Department of Medicine, Surgery and Neuroscience, University of Siena, Siena, Italy. [3]Department of Geriatrics, University Hospital of Nice, Nice, France. [4]Institute of Clinical Physiology, National Research Council, Lecce, Lecce, Italy

### Abstract

**OBJECTIVES:** Assessing bone health in pediatric populations is essential for preventing and monitoring skeletal fragility, especially in children and adolescents with genetic conditions (such as osteogenesis imperfecta) or undergoing chronic treatments that affect bone metabolism. However, ionizing radiation-based techniques, such as dual-energy X-ray absorptiometry (DXA), present several limitations in pediatrics, including reduced suitability for repeated examinations and bone size-dependent measurements that may lead to bone mineral density (BMD) underestimation. In this context, Radiofrequency Echographic Multi-Spectrometry (REMS), a non-ionizing ultrasound-based technology, represents a promising alternative. This study aimed to acquire REMS data from a pediatric population to develop age-specific reference curves and spectral reference models for BMD assessment throughout skeletal growth.

**METHODS:** Pediatric individuals aged 5 to 20 years who underwent REMS acquisitions at the proximal femur and lumbar spine were included. REMS analyzes native, unfiltered radiofrequency ultrasound signals acquired during a conventional echographic scan. Based on existing REMS models for adult populations, a dedicated pediatric-specific algorithm was developed to estimate BMD and Z-score. These estimates were compared with DXA measurements obtained at the corresponding skeletal sites. The association between REMS- and DXA-derived measurements at both sites was assessed using Pearson's correlation coefficient (r). Agreement between the two techniques, as well as concordance with DXA reference distributions, was further evaluated using Bland–Altman analysis.

**RESULTS:** Assessment included 105 femoral and 120 lumbar spine examinations. At the femoral site, REMS-derived measurements showed strong correlations with DXA, with $r = 0.92$ ($p < 0.0001$) and $r = 0.80$ ($p < 0.0001$) for BMD and Z-score, respectively. Likewise, lumbar spine analysis demonstrated strong correlations between REMS and DXA for both BMD ($r = 0.93$, $p < 0.0001$) and Z-score ($r = 0.90$, $p < 0.0001$) values. REMS-derived BMD distributions showed good concordance with DXA reference curves at both skeletal sites.

**CONCLUSIONS:** These findings indicate that REMS represents a highly promising tool for bone health assessment in vulnerable populations, including pediatric patients. By analyzing native radiofrequency signals and leveraging age-specific spectral models, REMS offers a reliable, radiation-free approach for the early identification of altered bone health and longitudinal monitoring throughout growth in pediatric populations.

### Disclosure

Not declared

### P49

### Development of Radiographic References for Skeletal Maturity in Indian Children

Anuradha Khadilkar[1], Vaman Khadilkar[1], Chirantap Oza[1], Shruti Mondkar[2], Nikhil Shah[1,3], Rubina Mandlik[1], Sharvani Patil[1]

[1]Hirabai Cowasji Jehangir Medical Research Institute, Jehangir Hospital, Pune, India. [2]Endo Nurture Pediatric Endocrine & Growth Clinic, Mumbai, India. [3]Department of Pediatric Endocrinology, MRR Children's Hospital, Mumbai, India

### Abstract

**Background:**
The Tanner–Whitehouse (TW) method is a globally recognized system for assessing skeletal maturity using radiographs of the hand and wrist. However, its existing reference (TW3) are based primarily on Western and East Asian populations, limiting their accuracy when applied to Indian children who differ in genetic, nutritional, and environmental backgrounds.

**Objective:**
To develop population-specific reference values for Skeletal Maturity Scores (SMS) in Indian children aged 1–18 years using the TW radius–ulna–short bones (RUS-13) system, and to validate these data against the current TW3 system.

**Methods:**
This retrospective study analyzed 3,549 left-hand radiographs (1,741 boys; 1,808 girls) from healthy Indian children obtained from previously approved bone age studies conducted between 2021 and 2024. Radiographs were scored using the TW3 RUS-13 method by two trained observers. Age- and sex-specific median SMS values were computed and smoothed using Gaussian curve fitting. The Indian SMS was validated prospectively in 50 children (20 healthy, 30 with endocrine disorders) and retrospectively for adult height prediction by substituting Indian bone ages into TW3 adult height prediction equations.

**Results:**
SMS values increased progressively with age, showing earlier skeletal maturation in girls than boys. Indian children reached full skeletal maturity (SMS = 1000) at ~16.5 years in girls and ~17.5 years in boys. Comparison with TW2 and TW3 references indicated that Indian maturation patterns lie between these datasets. Validation demonstrated strong agreement between Indian and TW3 bone ages (ICC > 0.95) and improved accuracy in adult height prediction using Indian SMS (Mean Absolute Error = –0.1 cm for Indian vs –0.9 cm for TW3).

**Conclusion:**
This study provides the first comprehensive Indian reference for skeletal maturity assessment. The Indian SMS is likely to enhance clinical accuracy for growth evaluation, endocrine diagnosis, and height prediction, supporting region-specific references for pediatric skeletal maturity.

Fig 1: **Age- and sex-specific skeletal maturity score (SMS) curves for Indian boys and girls compared with TW2 and TW3 reference data.**

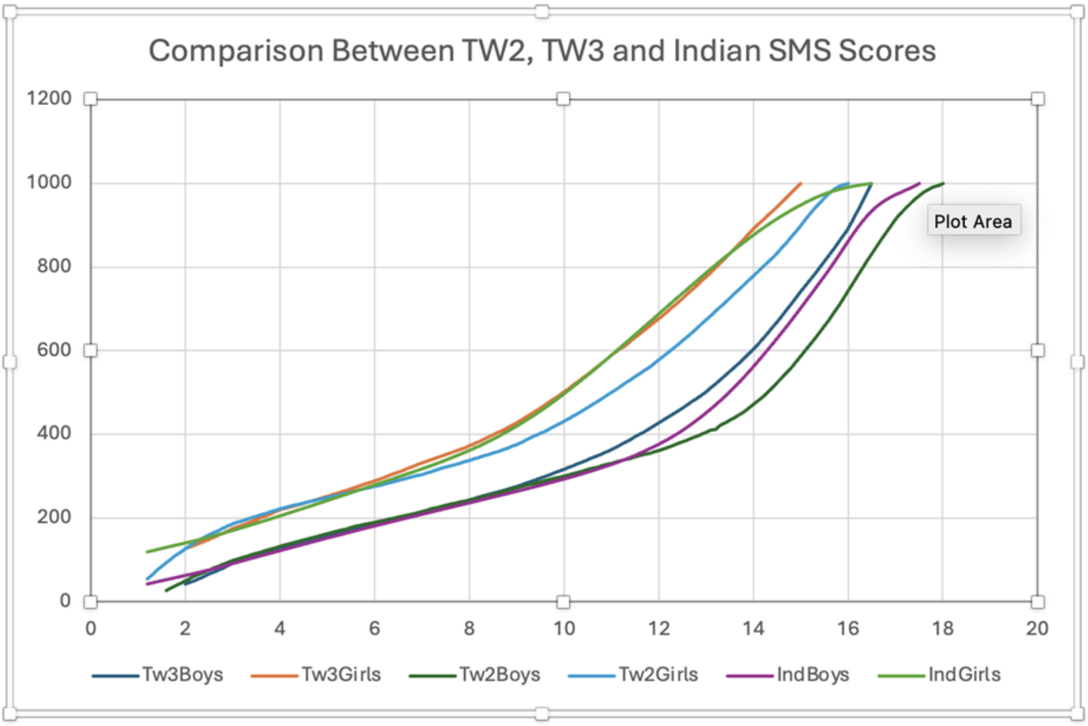

### Disclosure

NA

### P50

### Quantification of Fracture Healing in Adolescents Using High-Resolution Peripheral Quantitative Computed Tomography: A Pilot Study

Camryn McMillan[1,2], Nicole Boisvert[1,2], Steven Boyd[1], Graham Thompson[2,3], Danielle Whittier[1,2]

[1]McCaig Institute for Bone and Joint Health, Calgary, Canada. [2]Alberta Children's Hospital Research Institute, Calgary, Canada. [3]Departments of Pediatrics and Emergency Medicine, Calgary, Canada

### Abstract

### Objectives

This study aims to determine 1) the feasibility of investigating fracture healing in adolescents and 2) if bone microarchitecture changes can be tracked through healing using high-resolution peripheral quantitative computed tomography (HR-pQCT).

### Methods

Adolescents aged 10-15 years with a stable distal radius fracture requiring closed reduction were recruited from the Alberta Children's Hospital Emergency Department over a 6-month period. The participants' fractured radii were imaged using HR-pQCT (XtremeCT II, Scanco Medical) at 1-, 2-, 4-, 8-, and 12-weeks post fracture. Scan regions were acquired at a relative offset of 4% of the ulna length. The scan region was shifted proximally as necessary to capture the fracture or avoid the physes. Each scan consisted of a 20.4mm long region with a 61µm nominal isotropic resolution. To incentivize retention, 3D printed models of the participants' fracture were given at the conclusion of the final study visit. Total volumetric bone mineral density (Tt.BMD), total bone volume fraction (BV/TV), and total bone area (Tt.Ar) were measured. Paired sample t-tests were used to determine if significant differences ($p<0.05$) exist between 1- and 12-weeks post fracture.

### Results

A total of 11 adolescents (2 female, 9 male, mean age of 12.6 ± 1.2) have been enrolled in the study, of which eight have completed the study protocol. Only two of 40 study visits have been missed, and no participants were lost to follow-up. Over the 12-week healing period, participants had a 15.6% average increase in Tt.BMD ($p=0.02$) and an 18.4% increase in BV/TV ($p=0.003$). There was no significant change in Tt.Ar between these timepoints.

### Discussion and Conclusion

This is the first study to use HR-pQCT to study fracture healing in a pediatric population. These results suggest that despite the additional complexity in recruitment and retention of pediatric populations, it is feasible to study fracture healing *in vivo* using HR-pQCT. The use of providing 3D printed models of the participants' fractured bones as an incentive appeared to help with retention of this younger population. Changes in Tt.BMD and BV/TV alongside visual inspection of images (Figure) confirmed the healing process could be quantified in adolescents over this timeframe.

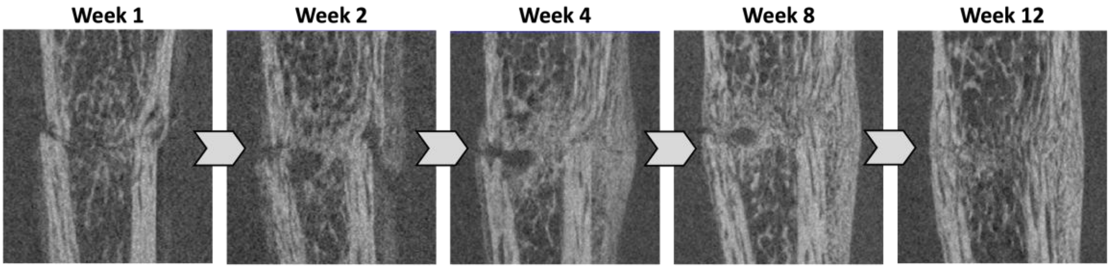

Figure 1 - Example of fracture healing progression captured by HR-pQCT in 12-year-old male. Slices shown of the radii are in the coronal plane.

### Disclosure

None declared

### P51

### Revised Reference Ranges for Trabecular Bone Score in Children 5 to 20 Years of Age

David Weber[1,2], Halley Wasserman[3,4], Guillaume Gatineau[5,6], Heidi Kalkwarf[3,4], Michael Levine[1,2], Babette Zemel[1,2]

[1]The Children's Hospital of Philadelphia, Philadelphia, USA. [2]Perelman School of Medicine at the University of Pennsylvania, Philadelphia, USA. [3]Cincinnati Children's Hospital Medical Center, Cincinnati, USA. [4]University of Cincinnati, Cincinnati, USA. [5]Lausanne University Hospital & University of Lausanne, Lausanne, Switzerland. [6]Medimaps group SA, Geneva, Switzerland

### Abstract

### Objectives:

Trabecular bone score (TBS) is a textural index derived from lumbar spine (LS) dual exergy X-ray absorptiometry (DXA) images. TBS predicts fractures independently of bone mineral density in adults, but pediatric use remains limited. We aimed to develop pediatric TBS reference ranges using the new TBS Insight v4.0 software, determine if these differed from those from the prior software version published by Kalkwarf, et al., 2022, and investigate associations with age, sex, anthropometrics, and fracture.

### Methods:

We analyzed TBS data from 9898 scans (5060 F) obtained on Hologic densitometers in healthy US children enrolled in the Bone Mineral Density in Childhood Study. TBS reference ranges for youth 5-20y by age and sex were generated using the Lambda, Mu, Sigma method. Precision, percent coefficient of variation (%CV), was determined on a sub-sample of subjects with repeat scans. Group differences were assessed by paired t-tests, associations between variables by Spearman's rho and Cox regression.

### Results:

Revised TBS reference ranges differed significantly from previously published values (Figure 1), $p<0.0001$. The %CV for TBS L1-L4 (2.4%) was improved from the prior data (2.9%). TBS values increased with age, with the greatest increases during puberty. TBS values were higher in males at younger ages, and higher in females during adolescence. There were nonlinear associations between TBS Z-scores and height- and BMI Z-scores, peaking at rho=0.43 at 12y (F) and 0.34 at 14y (M) for height ($p<0.0001$ for both), and rho=0.38 at 15y (F) and 0.31 at 15y (M) for BMI ($p<0.0001$ for both). TBS Z-scores were not associated with risk of incident low-to-moderate impact fracture over 6 years (HR 0.97, 95%CI: 0.84,1.12).

### Conclusions:

Revised pediatric reference ranges for TBS differed from those previously published and should be used for scans obtained using TBS Insight v4.0 software. TBS values increased during childhood in a sex dependent manner, were associated with height and BMI but not incident fracture in this cohort of healthy children. Future studies of TBS in children at risk of skeletal fragility are needed.

Figure 1. Revised pediatric TBS reference ranges (solid line) differed from those from the prior software version (dashed line).

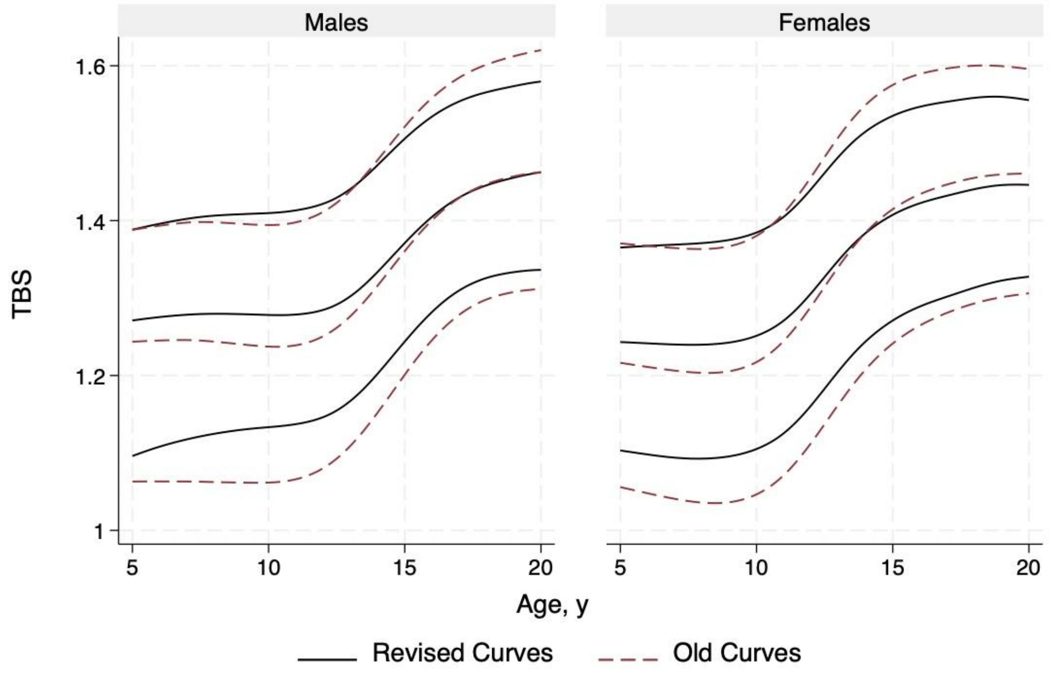

### Disclosure

DRW has been consultant for Ascendis, Catalyst, and Merck. HMW has been a consultant for Alexion and Kyowa Kirin. The other authors report no disclosures.

### P52

### Normative DXA- Derived Reference Data for Vertebral Morphometry in Healthy Children and Adolescents Aged 5–18 Years

Anuradha Khadilkar[1], Nicola Crabtree[2], Dipali Ladkat[1], Suma Uday[3], Raja Padidela[4]

[1]Hirabai Cowasji Jehangir Medical Research Institute, Pune, India. [2], Department of Endocrinology and Diabetes, Birmingham Women's and Children's NHS Foundation Trust (Birmingham Children's Hospital), Birmingham, UK. [3]Senior Consultant-Paediatric Diabetes, Endocrinology and Metabolic Bone Disease, Mazumdar Shaw Medical Centre, Narayana Healthcity, Banglore, India. [4]Royal Manchester Children's Hospital, Manchester, UK

### Abstract

**Background:**

In children and adolescents, osteoporosis commonly presents as vertebral fractures, which may be asymptomatic. Accurate identification of vertebral deformities requires appropriate vertebral morphometry reference data, which remain limited, in growing children. Our objective was to establish normative reference data for vertebral morphometry from T4 to L4 using DXA-derived Vertebral Fracture Assessment(VFA) in healthy children and adolescents( 5–18years), and to examine variation by age, sex, and anthropometry.

**Methods:**

Cross-sectional, observational study in healthy children and youth (5-18 years) from Pune, India. Participants demonstrated normal growth and had no history of chronic illness/conditions affecting bone. DXA scans were performed using a GE-Lunar iDXA system (GE Healthcare, Wisconsin, enCOREV18). VFA images were analysed using a standardized six-point morphometric technique to derive anterior, middle, and posterior vertebral heights and ratios. Reader precision (reviewed by two clinicians experienced in paediatric VFA and adjudicated by a senior DXA clinical scientist) and inter-observer reliability were assessed. Age- and sex-specific reference values were generated, and associations between vertebral dimensions and anthropometric parameters were examined.

**Results:**

420 participants (226 males) were analysed. The mean inter-reader reliability was 3.0% (range:1.7–4.5%).Vertebral height increased with age and stature, with acceleration during adolescence. Girls demonstrated larger vertebral dimensions for both height and age, particularly at the lumbar level. Vertebral heights plateaued around age 13 years in girls, but continued to increase in boys until 18 years. By 17 years, boys had significantly greater vertebral heights compared to girls. Coefficients of proportionality for average vertebral height increased caudally from T4 to L4, with a significant interaction between age and gender **(Figure 1)**. Older girls had proportionally smaller thoracic vertebrae compared to younger girls or male counterparts. Vertebral height ratios remained relatively stable across age groups, reflecting the preservation of vertebral shape during growth, although mild physiological anterior wedging was observed in thoracic vertebrae.

**Conclusions:**

This study establishes the first normative reference dataset for DXA-derived vertebral morphometry in children and adolescents, providing a critical benchmark for differentiating physiological variation from pathological deformity. These data strengthen the diagnostic framework for vertebral assessment and support earlier and reliable identification of vertebral fractures.

### Vertebral Height Ratio to T4-L4 by Gender and Age

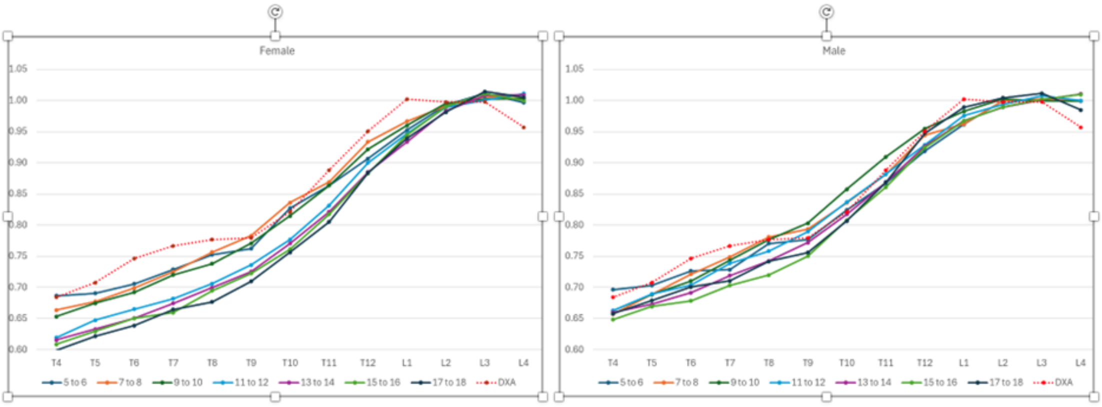

Figure 1 depicts age- and sex-specific trends in coefficients of proportionality for average vertebral height from T4 to L4. Coefficients increased caudally across vertebral levels, with variation by age and sex. The red line represents the coefficients of proportionality derived from the manufacturer-provided iDXA reference data and is shown for comparison with values obtained from the study cohort.

### Disclosure

None declared

### P53

### Impaired Bone Health in Pediatric ALL Survivors Despite Normal DXA: Insights from HR-pQCT

Nina Lenherr-Taube[1], Nicole Bodmer[2], Aziz Chaouch[3], Etienne Sochett[4], Flavia Wehrle[1], Marianne Rohrbach[1], Camilla Saladin[1], Hans-Jörg Häuselmann[5], Daniel Konrad[1]

[1]University Children's Hospital Zurich, Zurich, Switzerland. [2]University Children's Hospital Zurich, Zürich, Switzerland. [3]Department of Epidemiology and Health Systems, Lausanne, Switzerland. [4]The Hospital for Sick Children, Toronto, Canada. [5]Center for Rheumatology and Bone disease, Zurich, Switzerland

### Abstract

*Objectives:* Acute lymphoblastic leukemia (ALL) is the most common type of childhood cancer. With improved survival rates more attention has turned to long-term outcomes such as impaired bone health. DXA is the standard diagnostic tool to assess bone mineral density (BMD) in children at risk for bone health complications. High-resolution peripheral-quantitative-computed-tomography (HR-pQCT) is a three-dimensional diagnostic tool which evaluates both, cortical and trabecular bone microarchitecture and may have advantages over DXA. The aim of this study was to evaluate whether HR-pQCT can detect early bone health impairment in children and adolescents following ALL treatment even if lumbar spine BMD (LSBMD) z-score from DXA is normal.

*Methods*: In this cross-sectional observational study at the University Children's Hospital in Zurich, Switzerland HR-pQCT findings in children and adolescents within 5 years after ALL treatment were compared to healthy controls. All participants underwent HR-pQCT scans of radius and tibia and completed a bone health-related questionnaire. Additional assessments for ALL patients included LSBMD from DXA, vertebral fracture (VF) assessment from lateral spine radiographs (EOS) and bone health-related laboratory measures. Comparisons of HR-pQCT parameters between the two groups were tested using Welch t-test or Mann-Whitney U test.

*Results*: Complete HR-pQCT data of 30 ALL survivors (16 females; 14 males; mean age 9.7 ± 2.9 years) and 60 healthy controls (32 females; 28 males; mean age 9.9 ± 3.1 years) were analyzed. Five children and adolescents after ALL treatment (17%) showed evidence of one or more VF. LSBMD z-scores were within normal range for all patients (mean 0.09 ± 0.85 SDS). Compared with healthy controls, pediatric ALL survivors showed significantly reduced cortical area (p=0.013), cortical BMD (p=0.001) and cortical thickness (p=0.006) at the tibia, as well as reduced cortical area (p=0.046) and cortical BMD (p=0.008) at the radius in HR-pQCT.

*Conclusion:* Despite normal LSBMD DXA z-scores, a substantial proportion of children and adolescents after ALL treatment demonstrated vertebral fractures and several lower cortical parameters in both, the radius and tibia in HR-pQCT. These findings suggest that HR-pQCT may serve as a valuable adjunctive tool for the early detection of bone health impairment in pediatric ALL survivors.

### Disclosure

None declared

### P54

### A Novel Use of the Genant Semi Quantitative Method, Adapted to MRI, to Assess Vertebral Fractures in Pediatric Patients with Chronic Recurrent Multifocal Osteomyelitis (CRMO) at Baseline

Ross Fraleigh[1,2,3], Xingchang Wei[4], Paivi Miettunen[5,2,3]

[1]University of Limerick, Limerick, Ireland. [2]Alberta Children's Hospital and University of Calgary, Pediatrics, Calgary, Canada. [3]McCaig Institute for Bone and Joint Health, Calgary, Canada. [4]Alberta Children's Hospital, Diagnostic Imaging, Calgary, Canada. [5]Addenbrooke's University Hospital, Cambridge, UK

### Abstract

OBJECTIVES:

Chronic Recurrent Multifocal Osteomyelitis (CRMO), also known as Chronic Nonbacterial Osteomyelitis, is a rare autoinflammatory bone condition, typically affecting children. Whole body MRI is used to identify CRMO lesions, including vertebral ones. Our objective is to present a novel use of the Genant Semiquantitative technique to assess the severity of vertebral fractures in pediatric patients with vCRMO by using MRI.

METHODS:

Patients: Data was prospectively collected on all consecutive patients with childhood onset (<18years) CRMO, (data collection 2003-2023). Those patients with vertebral lesions were included for analysis.

Measurements: Whole body or focused magnetic resonance imaging (MRI) was used to identify CRMO lesions at baseline. Patients with vertebral fractures had also plain radiographs of the spine. All vertebral fractures were analyzed by Genant Semiquantitative technique which was adapted to MRI to grade the involvement of the vertebra. The vertebral fractures identified by MRI were then compared to vertebral fractures identified by plain radiographs and the Genant grading was compared between the MRI and plain radiographs.

RESULTS:

Nine CRMO patients had vertebral lesions at diagnosis; 55.5% (5/9) were female. Median (range) age at diagnosis was 11.4 (7.3-13.9) years. There were a total of 72 CRMO lesions in 9 patients, with 52.8% (38/72) located in the spine: 71.1% (27/38) in thoracic, 21.1% (8/38) in sacral and 5.3% (2/38) in the lumbar spine. Of 38 vertebral lesions, 14/38 (36.8%) (in four patients) had baseline vertebral fractures, all located in the thoracic spine. Of these fractures, 6/14 (42.9%) were Genant 1 (4 Biconcave, 2 Crush), 2/14 (14.3%) were Genant 2 (2 biconcave), and 6/14 (42.9%) were Genant 3 (3 wedge, 3 biconcave). There was no difference in the vertebral fracture severity by the Genant technique applied to plain radiographs versus MRI.

CONCLUSIONS

1. The MRI adapted Genant Semiquantitative method is effective for identifying vertebral fractures in vCRMO and classifying their severity.
2. It provides a standardized and reproducible method to record and follow vertebral CRMO fractures.

Future studies are now needed to validate this method in a prospective vCRMO cohort to confirm its use to study treatment efficacy and vertebral fracture outcomes.

### Disclosure

None declared

### P55

### The Relationship Between Phosphopenic Rickets and Bone Age in South African Children Treated at a Single Clinic Between 2006 and 2022

Fabrice Bakwene Mboma[1], Kebashni Thandrayen[1], Ansuyah Magan[1,2]

[1]Department of Paediatrics and Child Health, Chris Hani Baragwanath Academic Hospital, Faculty of Health Sciences, University of the Witwatersrand, Johannesburg, South Africa. [2]Developmental Pathways for Health Research Unit, Johannesburg, South Africa

### Abstract

*Objective:*

To assess the relationship between phosphopenic rickets and bone age.

*Methods:*

This was a retrospective descriptive study of children with phosphopenic rickets, attending the Metabolic Bone Clinic at Chris Hani Baragwanath Academic Hospital between January 2006 and December 2022. Bone ages from the initial clinic visit radiographs and on subsequent follow-up visits during treatment were assessed by Greulich-Pyle method. Hand-wrist and knee radiographs taken at initial presentation were assessed for the radiological severity of rickets using the Thacher score.

*Results:*

Seventy-five patients' records diagnosed with phosphopenic rickets were reviewed. Ten (n=10/49; 20%) patients presented with delayed bone age at diagnosis. The Thacher score at the initial presentation was higher in the delayed bone age group compared to the normal bone age group (p=0.04). There was a negative correlation between the Thacher score and height for age z-score (HAZ) at presentation, thus children with more severe Thacher scores had lower HAZ scores (r=-0.36; $r^2$=0.13; p=0.013).

*Conclusion:*

Phosphopenic rickets negatively impacts stature and BA which is associated with higher radiological severity score of rickets. The clinical application of bone age assessments in addressing the short stature observed in this condition and mitigating possible associated risk factors is warranted to improve the management, quality of life and outcomes for these affected patients.

### Disclosure

None declared

### P56

### Referral Practices and Trends in Bone Densitometry at a Tertiary Paediatric Centre

James Moloney[1,2], Margaret Zacharin[1,2], Kung-Ting Kao[1,2], Peter Simm[1,2]

[1]Royal Children's Hospital, Melbourne, Australia. [2]Murdoch Children's Research Institute, Melbourne, Australia

### Abstract

OBJECTIVES:

This study is a retrospective audit looking at the utilisation of bone densitometry scanning at a tertiary paediatric hospital and trends in referral practices.

METHODS:

All patients who underwent dual-energy x-ray absorptiometry (DXA) scanning using a Horizon (Hologic, Bedford, MA) densitometer between 01/01/2019 and 15/02/2024 at the Royal Children’s Hospital (RCH) were included. Data regarding the referring team, indication, and bone density were collected from individual DXA referrals and the electronic medical record. Statistical analysis was performed using Microsoft Excel (Microsoft Corporation, Redmond, WA, USA).

RESULTS:

There were 3833 bone densitometry scans performed on 2406 different patients.

The most common indications were eating disorders (n=645, 16.83%), gender dysphoria (GD) (n=565, 14.74%), Duchenne muscular dystrophy (DMD) (n=289, 7.54%) and osteogenesis imperfecta (n=245, 6.39%). Endocrinology (n=1501, 39.16%) and Adolescent Medicine (n=1094, 28.54%) together accounted for over two-thirds of all bone densitometry referrals.

The annual proportion of referrals from Adolescent Medicine increased from 23.34% in 2019 to 32.77% in 2023 (Figure 1). This is accounted for by a significant increase in referrals for scans for patients with GD from 8.60% in 2019 to 22.93% in 2023, representing a 266.63% increase in the proportion of total scans done in children with GD during the study timeframe.

1546 patients (64.26%) had a single scan, whilst 860 patients (35.74%) had 2 or more scans. The highest rate of repeat scans was observed in children with achondroplasia (84.09% of patients), osteogenesis imperfecta (72.63%) and DMD (72.32%). All scans for achondroplasia were performed in the setting of clinical research.

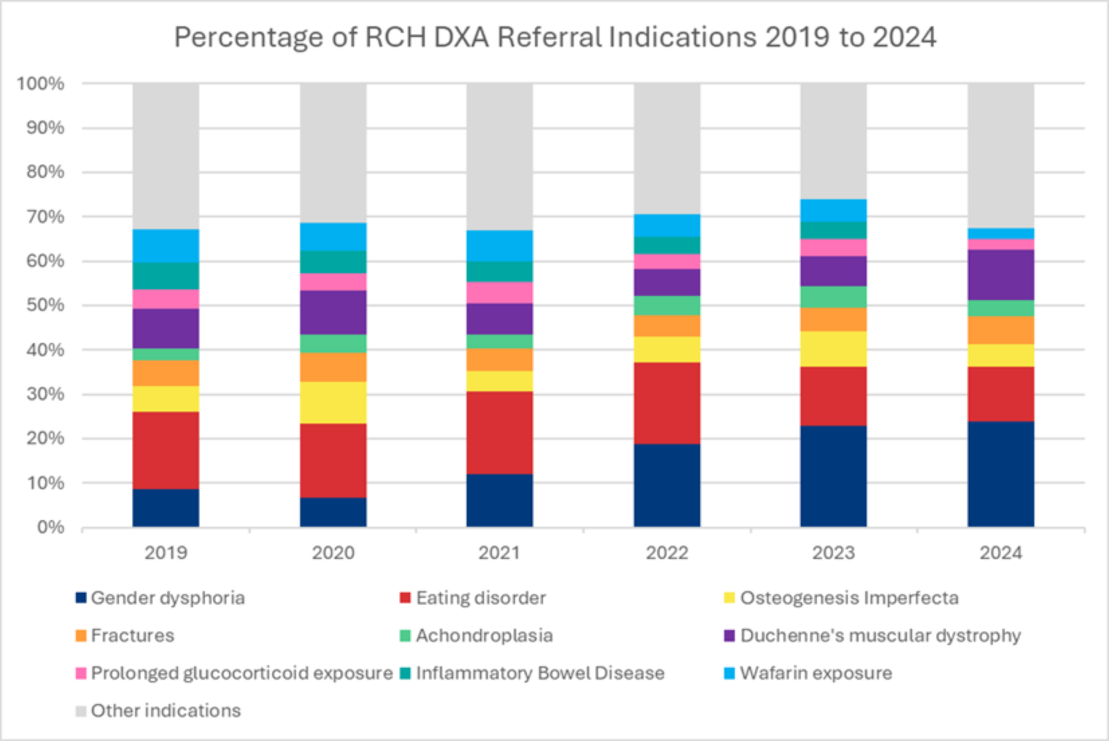

*Figure 1. Percentage of RCH DXA Referral Indications 2019 to 2024*

CONCLUSIONS: Bone densitometry is being used to screen for low bone density in a variety of different childhood illnesses, and for an increasing array of clinical research. This study's findings suggest an increasing demand for bone densitometry among children with gender dysphoria, highlighting the need for additional imaging capacity to ensure timely and equitable access for other clinical indications.

### Disclosure

None declared

### P57

### Rare MRI Features of Vitamin D Deficiency Rickets in a Veiled Teenager: New Insights

Victoria Schuhmann[1,2], Julian Schirl[1,2], Rodrigo Montero-Lopez[1,2], Wolfgang Högler[1,2]

[1]Department of Paediatrics and Adolescent Medicine, Johannes Kepler University Linz, Linz, Austria. [2]Centre for Growth and Osteology, Department of Paediatrics and Adolescent Medicine, Kepler University Hospital, Linz, Austria

### Abstract

### Background:

Nutritional rickets and osteomalacia due to vitamin D deficiency remain a serious global public health burden. Adolescents with low calcium intake and limited sun exposure, including those wearing full-body veils, are at increased risk. Diagnosis in late puberty can be challenging because growth plates are narrowing, and classical radiographic signs of rickets may be subtle. MRI findings in nutritional rickets are rarely reported.

### Presenting problem:

A 15-year-old veiled female presented to the paediatric surgery emergency department with a one-month history of atraumatic, activity-related pain in the left knee. Initial X-ray demonstrated metaphyseal fraying and widening of the epiphyseal line adjacent to nearly closed growth plates of the distal femur (Figure A, B). Subsequent MRI imaging revealed pronounced metaphyseal signal alterations of the femur and tibia (Figure C, D). Routine laboratory tests obtained prior to MRI already showed mild hypocalcaemia. With these findings, the endocrinological department was consulted and further evaluation revealed the characteristic diagnostic profile of severe vitamin D deficiency rickets, including markedly elevated serum alkaline phosphatase (1155 U/L; [50 - 117 U/L]) and parathyroid hormone levels (1293 pg/ml; [18.5 - 88 pg/ml]), undetectable 25-hydroxy vitamin D, hypocalcaemia (2.00 mmol/l; [2.10 - 2.55 mmol/l]), and hypophosphataemia (0.85 mmol/l; [0.90 - 1.55 mmol/l]).

### Clinical Management:

The diagnosis of nutritional rickets was established based on the combination of clinical, biochemical, and imaging findings. High-dose cholecalciferol and calcium supplementation were initiated. Family screening revealed vitamin D deficiency in all female members tested, although none showed biochemical or clinical evidence of active rickets or osteomalacia.

### Discussion:

In adolescents nearing skeletal maturity, physiological narrowing of the growth plates can attenuate typical radiographic manifestations of rickets, increasing the risk of underrecognition. In this case, MRI contributed to the diagnosis by revealing distinctive metaphyseal signal changes reflecting defective osteogenesis and growth plate elongation caused by hypertrophic cartilage extension, which were only subtle on conventional radiography. This highlights the importance of considering rickets in at-risk adolescents and supports the use of MRI as an additional diagnostic tool in late pubertal patients when conventional imaging is inconclusive.

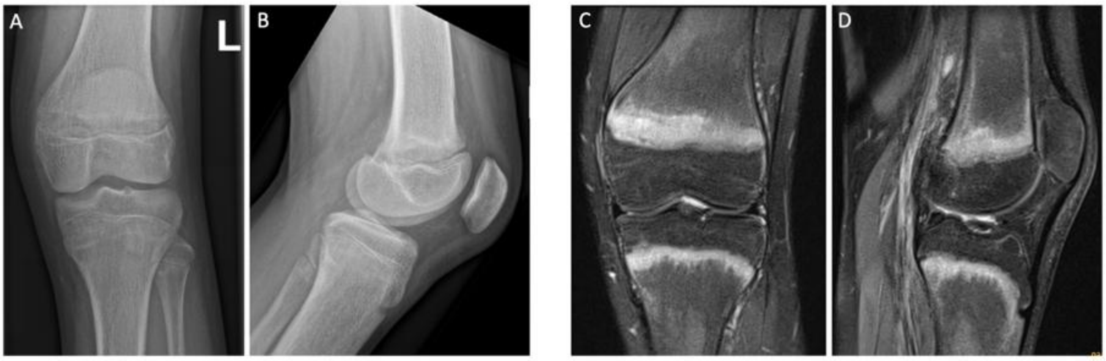

Figure: A, B: X-ray of the left knee - metaphyseal fraying and widening of the epiphyseal line of the distal femur in anteroposterior (A) and lateral (B) projections.
C, D: MRI scan of the left knee (proton density weighted Turbo Spin Echo sequence with fat saturation) - accentuated signal alterations in the tibial and femoral metaphysis in coronal (C) and sagittal (D) planes.

### Disclosure

None declared

### P58

### Quantitative Ultrasound May Serve as a Radiation-Free, Point of Care Test for Bone Fragility in Infants

Angela Bachim[1,2], James Crowe[1,3]

[1]Baylor College of Medicine, Houston, USA. [2]Texas Children's Hospital, Houston, USA. [3]Texas Children's Hosptial, Houston, USA

### Abstract

Objectives: To assess whether children under 2 years old with either genetic or radiographic evidence of bone fragility would have negative tibial speed of sound z-scores.

Methods: Cases with known bone fragility were identified by consultation to the child abuse team and had either radiographic or genetic evidence of bone fragility at a quaternary children's hospital. Child abuse evaluation included a skeletal survey as part of routine care. Caregivers were consented to participate in the study to obtain the tibial speed of sound (SOS) measurement. Mid-tibial (SOS) by quantitative ultrasound (QUS) was obtained on each subject using the MiniOmni (BeamMed). A repeated tibial SOS was obtained in 1 infant 1 month after definitive treatment of the underlying cause of her rickets. Per the device instructions, the CM probe was used as the first line to obtain tibial SOS. If the CM probe would not register any measurement, the CR probe was then used to obtain the measurement. The device only provides z-scores for the CM probe, as the CR probe is intended for use in premature infants. Therefore z-scores were also calculated using the age-specific reference value and standard deviation provided by the device manufacturer. This study was approved by the institution's IRB.

Results: All 5 of the infants with evidence bone disease had negative z-scores. The 4 infants that presented with multiple fractures had z-scores lower than the 1 infant who presented with osteopenia but no fractures. The 1 infant who was re-measured after definitive treatment of the underlying cause of her rickets had a markedly improved z-score.

Conclusion: Quantitative ultrasound, tibial speed of sound may be a useful radiation-free, point-of-care test to screen for bone fragility in infants. More study is needed, but this would fill a crucial gap in pediatric bone fragility evaluation.

| Chron Age (months) | Gestational age at birth (weeks) | Sex | Bone problem and diagnosis modality | Current fractures | Tibial SOS (m/s) | Device Z-score | Calculated Z-score |
|---|---|---|---|---|---|---|---|
| 6* | 38 | F | Rickets on x-ray | 2 lateral ribs, 2 femur, 1 fibula; total= 5 | 2531 | n/a | -4.3 |
| 8 | 33 | M | Rickets on x-ray | 10 ribs, 1 fibula; total= 11 | 2321 | n/a | -6.0 |
| 8 | 33 | F | Rickets on x- | 8 ribs, 1 humerus, | 2351 | -7.5 | -6.6 |

| | | | | | | | |
|---|---|---|---|---|---|---|---|
| | | | ray | 1 fibula; total = 10 | | | |
| 13 | 31 | M | Osteopenia on x-ray | None | 3100 | -1.0 | -0.99 |
| 1 | 35 | F | OI type IV-genetic and x-ray | 2 ribs, 1 ulna, 1 humerus; total = 4 | 2729 | n/a | -3.36 |
| 11* | 38 | F | Resolved rickets | n/a | 2808 | n/a | -0.96 |

*Notes the same patient with 2 different measurements

### Disclosure

None declared

### P59

### Incidence of Serum Biochemical Abnormalities Associated with Metabolic Bone Disease of Prematurity in Preterm Neonates

Charlotte Maden[1,2], Rebecca J Moon[1,2,3], Aneurin Young[2,4], Nicholas C Harvey[1,2], Mark Johnson[2,4]

[1]MRC Lifecourse Epidemiology Centre, University of Southampton, Southampton General Hospital, Tremona Road, Southampton, UK. [2]NIHR Southampton Biomedical Research Centre (NIHR203319), University of Southampton and University Hospital Southampton NHS Foundation Trust, Southampton General Hospital, Tremona Road, Southampton, UK. [3]Regional Centre for Paediatric Endocrinology, Southampton Children's Hospital, University Hospital Southampton NHS Foundation Trust, Tremona Road, Southampton, UK. [4]Neonatal intensive care unit (NICU), Princess Anne Hospital, University Hospital Southampton NHS Foundation Trust, Coxford Road, Southampton, UK

### Abstract

**Objectives:** Preterm infants are at increased risk of metabolic bone disease of prematurity (MBDP), a condition characterised by radiographic abnormalities occurring late in the clinical course, including osteopenia, rickets and fractures. Early identification relies on serum alkaline phosphatase (ALP) and phosphate. However, various thresholds for these markers have been suggested in existing literature, ranging from an ALP≥500 to 900iu/L with and without simultaneous low phosphate (<1.3 or <1.8mmol/l). We determined the incidence of biochemical MBDP in preterm infants, and described associated maternal and neonatal characteristics.

**Methods:** A retrospective analysis of real-world clinical data from preterm infants (born <30 weeks' gestation (2009–2018) or <32 weeks (2018–2024)) at a tertiary UK (UK) neonatal unit. Routinely collected serum ALP and phosphate, neonatal characteristics (gestation age (GA) at birth, birthweight, sex) and maternal characteristics (parity, smoking and ethnicity) were extracted from clinical records.

We explored the incidence of biochemical MBDP using ALP thresholds of 500, 600, 700, 800 and 900iu/L alone and in combination with low serum phosphate (<1.3 or 1.8mmol/l). Only patients with ≥2 consecutive abnormal biomarker measurements after the first week of life were considered to have biochemical MBDP.

**Results:** Data for 391 infants were included (median (IQR); GA 28 weeks (26, 29), birthweight 935g (765, 1185)). Incidence of biochemical MBDP ranged from 1.0% when defined as ALP≥900iu/L with phosphate <1.3mmol/l to 51.7% when defined by only ALP≥500iu/L.

Using a definition for biochemical MBDP of ALP≥500iu/L, more infants with biochemical MBDP were male (63% vs 48%, p<0.01) and they were born earlier (median 27 vs 29 weeks, p<0.01). Similar trends were observed for definitions of biochemical MBDP combining ALP and phosphate, but with weaker associations as the threshold for ALP increased. Maternal parity, smoking and ethnicity did not differ for infants with and without biochemical MBDP.

**Conclusions:** The incidence of biochemical MBDP varied markedly depending on the thresholds for ALP and phosphate used, highlighting the need for clear clinical guidelines and diagnostic criteria. Irrespective of the definition used, biochemical MBDP was associated with male sex and earlier gestation at birth, highlighting infants who may benefit from nutritional strategies to prevent MBDP.

### Disclosure

None declared.

### P60

### Bisphosphonates, Bone Density and Fractures in Paediatric Osteogenesis Imperfecta: 23 Years of Longitudinal Data

Chris Worth, Lauren Rayner, Amish Chinoy, Raja Padidela

Royal Manchester Children's Hospital, Manchester, UK

### Abstract

OBJECTIVES

Osteogenesis imperfecta (OI) is a heterogeneous genetic disorder characterised by bone fragility and reduced bone mineral density (BMD). The natural history of bone density and fractures through childhood remains incompletely described, with longitudinal descriptions of effects of bisphosphonates (BP) limited.

We describe these in a large cohort of children with OI, and explore associations between bisphosphonate treatment, fracture frequency, and skeletal density over long-term follow-up.

METHODS

Retrospective review of all patients with OI, in a single specialist centre, over a 23-year period. Electronic notes were reviewed for clinical descriptions, treatment regimes and fracture incidence. Annual DEXA and pQCT scans, reported by consultants in paediatric radiology or metabolic bone, were reviewed for BMAD and volumetric density. Data were collated in Excel and analysed in Python 3.1.1.

RESULTS

Data were collected from 135 children (66 female) with OI (nosology types 1 = 90, 3 = 14, 4 = 28), representing 1276 years of longitudinal data. Median age at diagnosis: 1.5 years. Median age at measurement: 7.5 years.

Amongst patients with paired pre and on-treatment BMD data (n=41), BP use was associated with increased BMD scores (Figure1). Immediate-pre vs first on-treatment scores were: lumbar spine BMAD z –1.37 to –0.11 ($\Delta = +1.26$, $P<0.001$); WBLH z –2.48 to –0.49 ($\Delta = +1.99$, $P<0.001$); pQCT cort/trab -1.02 to +0.06 ($\Delta = +1.08$, $P<0.001$).

For those with pre and on-treatment fracture data (n=84), median long bone annualised fracture rate (AFR) was associated with BP treatment, decreasing from 0.81 pre-treatment to 0.18 on-treatment ($\Delta = -0.46$ ($P<0.001$ via Wilcoxon Rank)). Total AFR reduced from 1.56 to 1.15, ($\Delta = -0.63$ ($P<0.001$)). Those with Nosology Type 3 showed the greatest reduction (long bone AFR 5.5 to 2.15, $\Delta = -2.43$, $P<0.001$).

CONCLUSIONS

Within this large, and comprehensive, patient cohort, we have demonstrated a positive, and rapid, association between BMD scores and bisphosphonate treatment. This positive association plateaus with time, possibly reflecting weaning of BP dose to target appropriate BMD scores. BP also associates with fracture rates; the rate of clinically meaningful fractures reducing to a median of only 0.18 long bone fractures per year.

### Disclosure

CW, LR, AC and RP received financial support from Mereo Pharmaceuticals for the collection and analysis of this data. Statistical analysis input was received from Jason Wilson at Numerus.

### P61

### Normal Weight Obesity in Childhood Is Associated with Reduced Bone Mineral Density

Rebecca J Moon[1,2,3], Stefania D'Angelo[1], Sarah R Crozier[1,3,4], Keith M Godfrey[1,3], Nicholas C Harvey[1,3]

[1]MRC Lifecourse Epidemiology Centre, University of Southampton, Southampton, UK. [2]Regional Centre for Paediatric Endocrinology, Southampton Children's Hospital, University Hospital Southampton NHS Foundation Trust, Southampton, UK. [3]NIHR Southampton Biomedical Research Centre (NIHR203319), University of Southampton and University Hospital Southampton NHS Foundation Trust, Southamptpn, UK. [4]NIHR Applied Research Collaboration Wessex, Southampton, UK

### Abstract

### Objectives

Children living with obesity, as defined by body mass index (BMI), typically have higher bone mineral density (BMD) than healthy weight peers. Normal weight obesity (NWO) describes the combination of a BMI within the "healthy" range but with high body fat percentage (%BF). NWO has been associated with increased cardiovascular risk, but the effect on bone health is poorly described. We compared BMD in children with NWO to those with a healthy BMI and low %BF (LL) and a high BMI and %BF (HH).

### Methods

In the Southampton Women's Survey mother-offspring cohort study, children had anthropometry and whole body (WB) and lumbar spine (LS) DXA (Hologic Discovery) at age 8-9 years. Overweight/obese was defined by a BMI z-score (British 1990 reference data) ≥1. WB %BF was adjusted for age and sex and standardised within the cohort; %BF z-score (%BFz) ≥1 was classed as high.

For each sex, propensity score matching was used to match 1:1 NWO (BMIz<1 & %BFz≥1) to LL (BMIz<1 & %BFz<1) by BMIz and age, and to HH (BMIz≥1 & %BFz≥1) by %BFz and age. Whole body less head (WBLH) and LS bone mineral content (BMC), BMD and bone mineral apparent density (BMAD) were compared across groups using ANOVA. Results are shown as mean (SD).

#### Results

Twenty-four of 807 (3%) participants had NWO (9 male, mean BMIz 0.55 (0.36), %BF 34.2 (2.6)). The matched LL had similar BMIz (0.63 (0.29) p=1.0) but lower %BF (25.2 (3.2) p<0.001); HH had higher BMIz (1.65 (0.51), p<0.001) but similar %BF (34.5 (2.7) p=1.0).

WBLH BMC was similar for each group (p=0.92). WBLH BMD was lowest in children with NWO (0.620g/cm$^2$ (0.053), LL (0.667g/cm$^2$ (0.062), HH (0.679g/cm$^2$ (0.052), p=0.001 across groups), with a similar distribution for WBLH BMAD (NWO 0.018 g/cm$^3$ (0.002), LL 0.020 (SD 0.002), HH 0.020 (SD 0.002), p<0.001 across groups). LS BMD and BMAD had similar trends (p>0.05).

### Conclusions

Children with NWO had the lowest BMD. This finding suggests that excess adiposity without the additional loading weight may impair bone mineral accrual. Consideration of how to identify children with NWO and promote bone health is needed.

### Disclosure

None declared

### P62

### Within-Patient Growth Trajectories in Paediatric Osteogenesis Imperfecta: A 23-Year Cohort

Chris Worth, Avanti Saoji, Lauren Rayner, Amish Chinoy, Raja Padidela

Royal Manchester Children's Hospital, Manchester, UK

**Abstract**

**OBJECTIVES**

Linear growth rate is often impaired for children with osteogenesis imperfecta (OI); the degree differing by nosology type and severity. Previous large cohorts have generated cross sectional growth curves, but dense longitudinal data are lacking.

We aimed to describe longitudinal, within-person, changes in height and weight in a single centre, real world cohort, with analysis of effects from subgroups and treatment regimes.

METHODS

Retrospective review of all patients with OI, cared for by a single specialist centre, over a 23-year period. Height and weight z-scores were calculated from UK 1990 and WHO dataset hybrids. Data were collated in Excel and analysed in Python 3.1.1.

**RESULTS**

Over 1000 yearly height and weight measurements were available from 130 children with OI. Median (IQR) annual measurements, per patient, were 8 (4-11), with highest density from 3y-11y.

Those with nosology types 1 and 4 had mean height z-scores below population mean, at –0.74 and –1.62, but stable growth rates (+0.02 and +0.05 z-scores/year). Those with nosology type 3 had height z-scores significantly below average, at –4.06, as well as lower growth *rate* (–0.14 z-scores/year) (Figure 1).

In those with paired pre and on-treatment height z-scores (n=54), time series mixed modelling showed an upward change in trajectory after bisphosphonate start (+0.07 z-scores/year slope change, *P*=0.002). Restricted to type 1 patients (n = 46), slope seemed to improve by +0.05 z-scores/year (*P*=0.04) with effect reducing over time.

Weight was higher than height; mean weight z-scores for nosology groups 1, 4 and 3 were 0.0, -0.82 and –1.96 with longitudinal slopes of +0.04, 0.00 and –0.9 z scores per year. BMI z scores (slope) were +0.54 (-0.04), +0.36 (+0.103) and +1.15 (0.00) for each group.

**CONCLUSIONS**

These within-patient longitudinal data confirm previous cross-sectional studies' findings of below average height for most patients with OI. Height is particularly low in nosology type 3 and decreases over time. Bisphosphonates may confer some benefit to height, but effect is uncertain. Weight is closer to population mean than height in all groups and efforts must be made to monitor BMI in these patients.

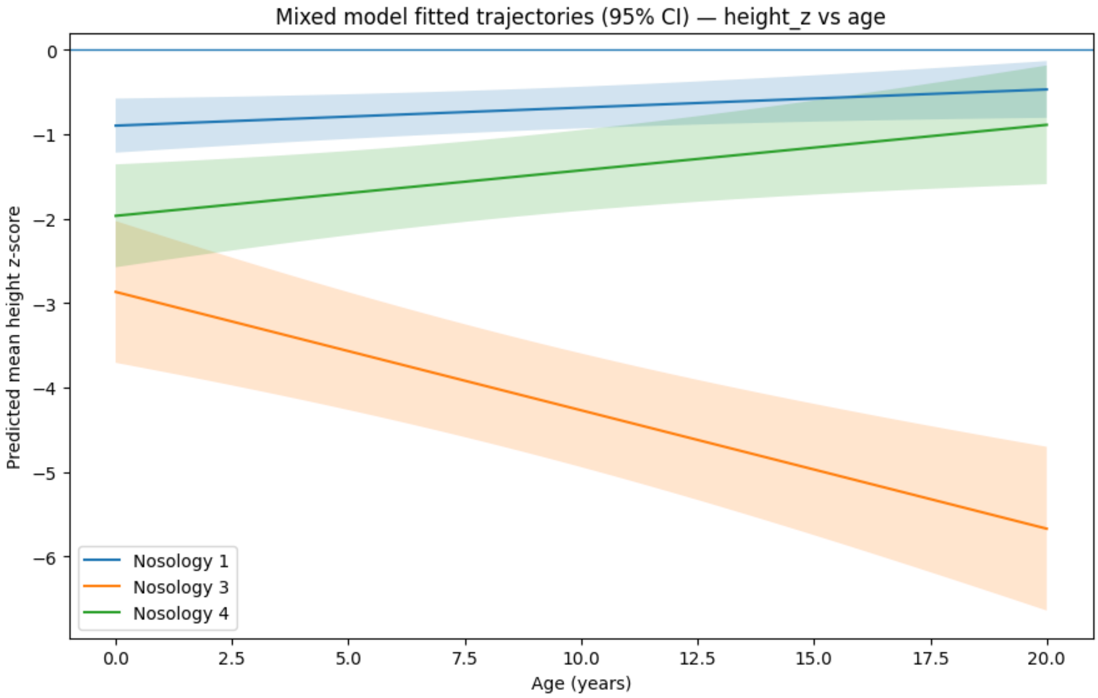

### Disclosure

CW, LR, AC and RP received financial support from Mereo Pharmaceuticals for the collection and analysis of this data

### P63

### Iatrogenic Vitamin D Toxicity within the Paediatric Population: An Acute Surge in National Cases

Isobelle Blinkhorn[1], Lucy Hoskyns[2], Katherine Aitken[3], Ana Gonzales Mereles[4], Ruchi Nadar[4], Renuka Ramakrishnan[5], Guftar Shaikh[6], Scott Williamson[7], Claire Wood[3], Amish Chinoy[8], Raja Padidela[8], Chris Worth[8]

[1]Stockport Hospital trust, Stockport, UK. [2]Stockport Hospital Trust, Stockport, UK. [3]Great North Children's Hospital, Newcastle, UK. [4]Birmingham Children's Hospital, Birmingham, UK. [5]Alder Hey Children's Hospital, Liverpool, UK. [6]Royal Hospital for Children, Glasgow, UK. [7]University Hospital Ayr, Aryshire and Arran, UK. [8]Royal Manchester Children's Hospital, Manchester, UK

### Abstract

**OBJECTIVE**

Vitamin D plays a key role in calcium homeostasis within the body and deficiency is common. However excessive consumption can result in toxicity and hypercalcaemia. With increased awareness around Vitamin D supplementation within the UK, there has been an increase in cases of Vitamin D toxicity (VDT) amongst the paediatric population.

Although cases of VDT remain uncommon, relaxed regulatory standards for colecalciferol supplements have led to an acute surge in cases.

**METHODS**

We present data from 12 children who presented across the UK with hypercalcaemia due to VDT between December 2024 - March 2025. Standardised data collection sheets were used in each centre, with data then amalgamated by a central team.

**RESULTS**

Age of presentation ranged from 2 months - 12 years, with 4 (33%) patients ≤1 year. Common presenting symptoms were vomiting (50%), lethargy (42%), altered bowel habits (33%), and poor feeding with weight loss (33%).

Patients had hypercalcaemia [mean 3.64mmol/L], a raised 25[OH]VitaminD [1281nmol/L] and suppressed parathyroid hormone (PTH) levels [0.28pmol/L].

Urinary calcium:creatinine (measured in 9 patients); showed mild-moderate hypercalciuria mean (range) 2.13mmol:mmol (1.08 – 4.7).

Treatment was initiated in all cases. The majority (92%) received intravenous hyper-hydration (92%) and pamidronate infusions (58%), with three (25%) requiring repeat doses. Seven (58%) patients adhered to a strict low calcium diet. One patient received furosemide and another was treated with prednisolone. Two patients (17%) developed nephrocalcinosis, and four (33%) experienced acute kidney injury (AKI). Median time to calcium normalisation (2.20-2.60 mmol/L) across the cohort was 37 days (Figure 1).

All patients were receiving Vitamin D treatment, with colecalciferol, prior to admission. Eleven patients (92%) were receiving a defective batch of colecalciferol, subsequently withdrawn by the manufacturer, following a safety alert.

**CONCLUSION**

This case series serves to remind clinicians of VDT as a potential cause of hypercalcemia in children and highlight effective treatment options. To minimise the risk of VDT to the paediatric population, we recommend stricter licensing laws for colecalciferol supplementation within the UK.

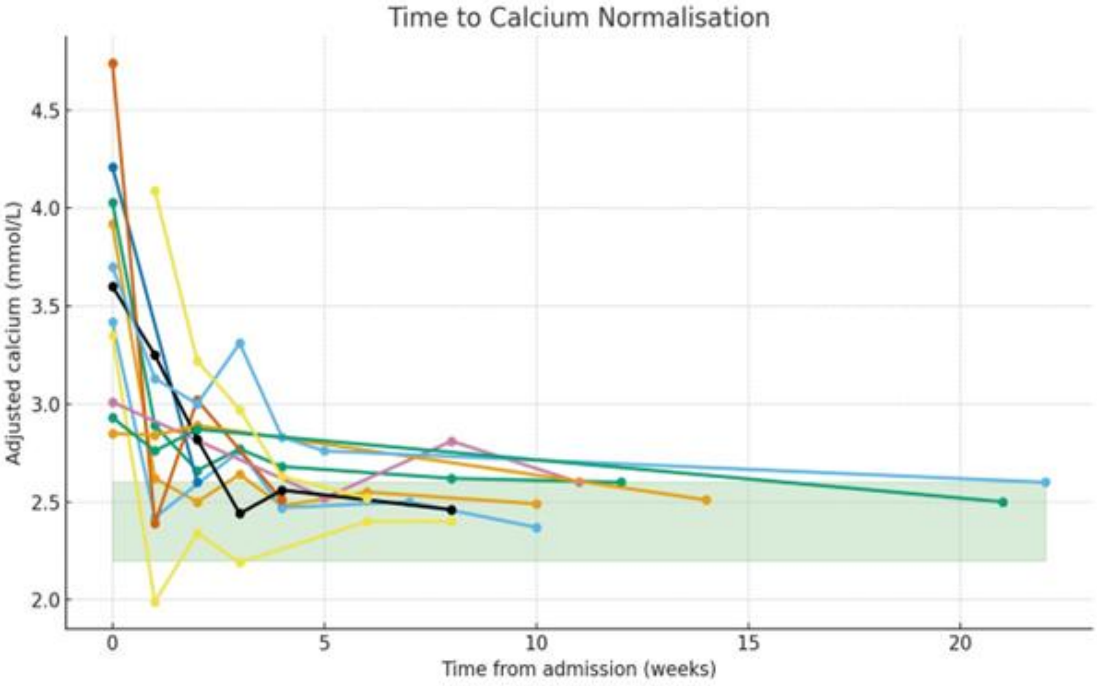

### Disclosure

None declared

### P64

### Incidence of Fractures in Youth with Cystic Fibrosis Increases Progressively with Age

Malinda Wu, Cynthia Wang, Sofia Harrison, Peter Mogayzel, Scott Blackman, Janet Crane

Johns Hopkins University School of Medicine, Baltimore, USA

### Abstract

OBJECTIVES:

People with cystic fibrosis (CF) experience early-onset osteoporosis diagnosed in young adulthood. Compared to the general population, they have a 100-fold and 2-fold increased risk for vertebral and non-vertebral fractures respectively. The objective of this study was to examine age-specific fracture incidence to establish if children with CF experience skeletal fragility before young adulthood, which is when most people with CF begin screening for osteoporosis.

METHODS:

Fractures were identified by manual chart review of patients in the Johns Hopkins pediatric CF clinic, followed by surveying patients (age 0-21Y) presenting to CF clinic to minimize bias from missing documentation. Medical charts were reviewed for additional fracture and demographic information.

RESULTS:

We identified 209 youth with CF for chart review; 52 (25%) fractured 70 bones by age 18Y. In 2025, we surveyed 156 patients, of whom 133 were in the chart review. Baseline characteristics of youth at survey completion revealed a median age of 12Y, 39% female, sufficient nutritional status (BMI +0.2 SDS) and normal lung function (FEV1 99 %predicted). Children who had fractured were older (15Y) than children who had not fractured (11Y), ($p < 0.001$). Of surveyed patients, 36 (23%) fractured 48 bones during childhood. The cumulative incidence of fracture by age 18Y was 62.4% (chart review) and 41.3% (survey) (Figure 1). There was no difference by sex. The incidence of fracture per 1000 person-years was 14-15 (age 0-4Y), 21-33 (age 5-9Y), 36-67 (age 10-14Y), and 74-75 (age 15-17Y). Upper long bones were the most common fracture site; two children experienced a vertebral fracture. By chart review, 73% of fractures documented mechanism of injury but lacked sufficient details to grade trauma level; 15% of fractures captured by survey were low/no trauma.

CONCLUSIONS:

Cumulative incidence of fracture in children with CF was 2.3-3.4-fold higher than reported in healthy US children. The incidence of fractures in CF youth remains high, similar to rates published between 1994-2018, despite tremendous improvements in CF care. Our study is limited by recall bias. Next steps are focused on identifying genetic, imaging and other clinical predictors of osteoporosis in people with CF.

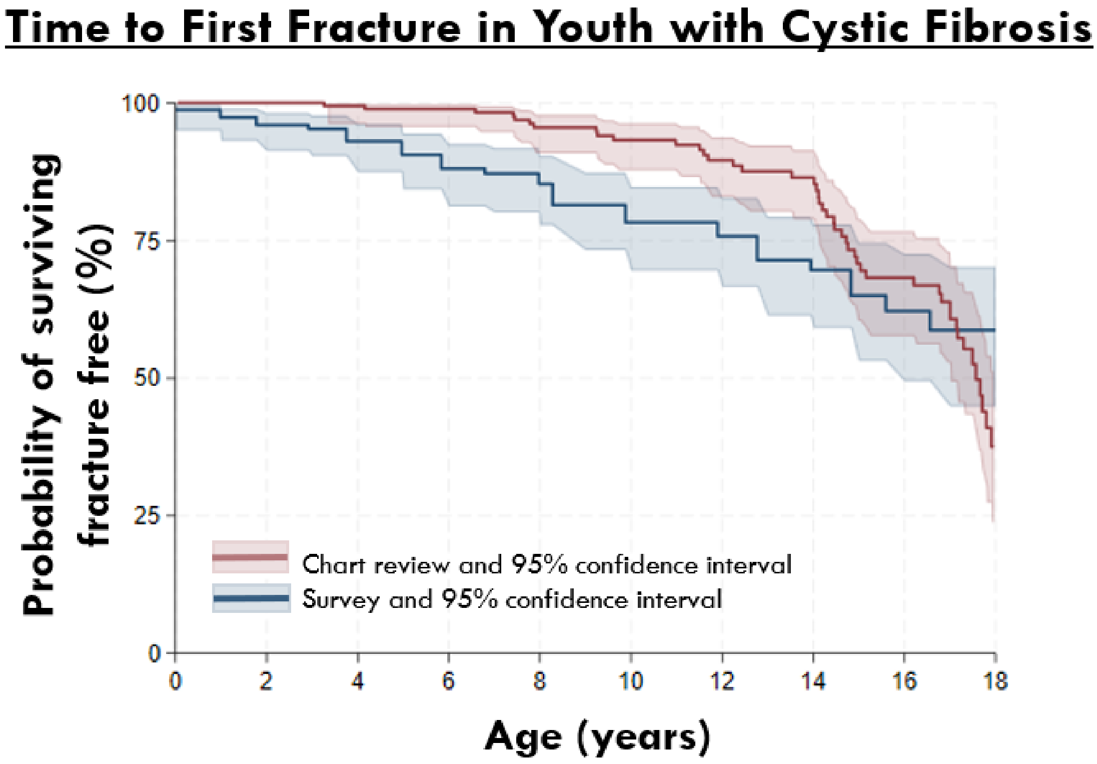

**Disclosure**

This work was supported by the Cystic Fibrosis Foundation [WU23GE0, WU21Q0, SHARR24H0] and the National Institutes of Health [1K23AR084615]. Role of funder: salary support.

### P65

Abstract withdrawn

### P66

Abstract withdrawn

### P67

### Determination of Parathyroid Hormone Reference Interval Using Laboratory Data in a UK Paediatric Population

Leanne Wherrett, Anna Robson, Amish Chinoy, Raja Padidela, Chris Worth

Royal Manchester Children's Hospital, Manchester, UK

### Abstract

**Objectives**:

Appropriate reference intervals (RI) for parathyroid hormone (PTH) are essential for the meaningful interpretation of bone biochemistry. Current practice uses paediatric RI from the literature, derived from healthy volunteers in the community. The current, age based RI, from this method, is from 0.7-1.6 to 6.3-7.2pmol/L (Figure 1). However, vitamin D status and calcium levels were not considered as part of these studies and could affect RI. As recruitment of healthy controls from the community is challenging, we examined pre-existing in-house laboratory data to establish paediatric specific PTH RI for a large, diverse population.

**Methods**:

PTH results were extracted from our laboratory IT system, covering a 24-month period from September 2022, for all patients under the age of 18 years. Analysis was performed using Roche methods. Biochemical exclusions were applied based on the plasma adjusted calcium, creatinine, urea, phosphate, alkaline phosphatase and serum 25[OH]VitD. Only one PTH result was included per patient. Clinical details were reviewed to exclude patients with bone or renal pathology and gestation < 37 weeks in patients < 6 months old. Outliers were removed using the Tukey method on Box-Cox transformed data, upper and lower RI were calculated by nonparametric analysis of the central 95% of values.

**Results**:

A total of 8450 PTH results, from 4368 patients, were extracted. After exclusions were applied, 620 PTH results were used to derive a RI of 1.6 (90% CI 1.5 – 1.7) to 7.4 (90% CI 7.0 – 8.2) pmol/L. Data closely matched existing reference data but did not support partitioning by sex or into separate age groups (Figure 1).

**Conclusions**:

This study demonstrates that laboratory data, combined with the application of strict exclusion criteria, may be used to derive local RI. These local RI showed general agreement with direct RI reported in the literature and support their continued use for our population. Uncertainty (90% CI) around the upper limit of PTH RI is relatively high (Figure 1) and must be taken into account during clinical interpretation.

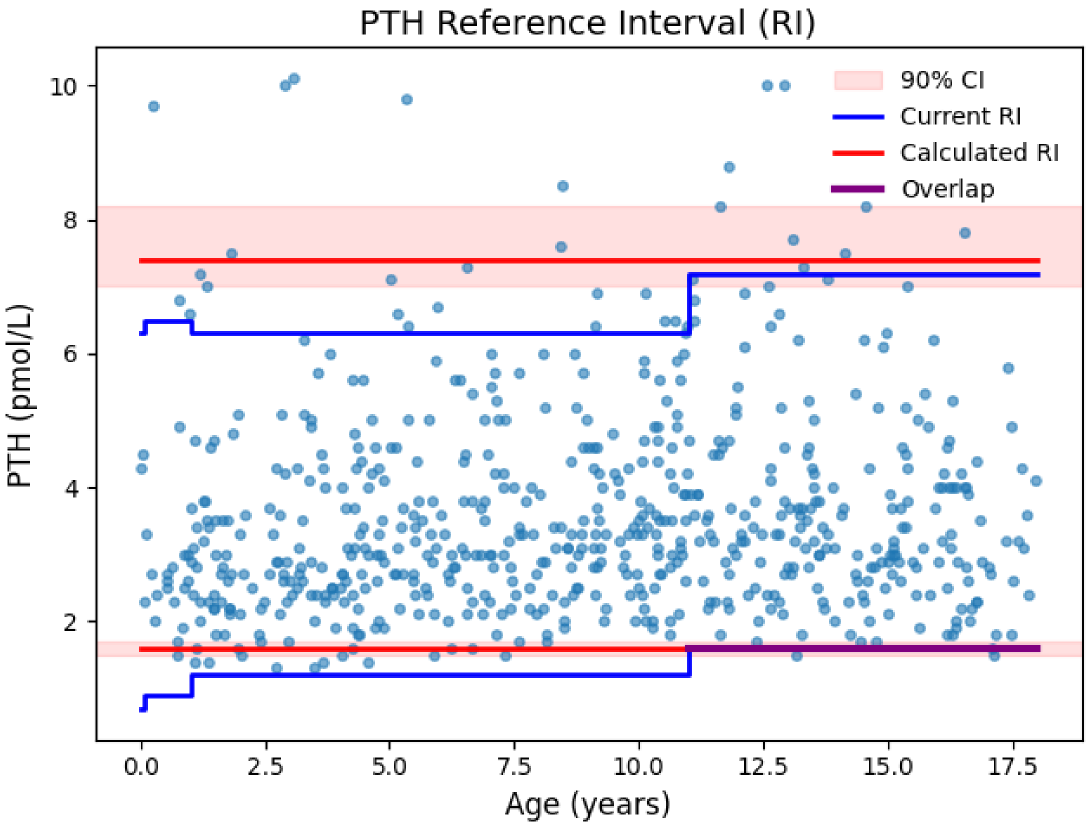

**Disclosure**

None declared

### P68

### Estimating the Global Birth Prevalence of Achondroplasia (ACH): A Systematic Literature Review and Meta-Analysis

Subhara Raveendran[1], Rachel Mathu[2], Devon Morgan[2], Chelsea Catsburg[2], Alden Smith[1]

[1]Ascendis Pharma, Inc., Palo Alto, CA, USA. [2]BluePrint Orphan, Inc, New York, NY, USA

### Abstract

OBJECTIVES:

Achondroplasia (ACH) is a rare genetic condition driven by a systemic fibroblast growth factor receptor 3 (FGFR3) variant, resulting in skeletal dysplasia and a range of serious musculoskeletal, neurological, and cardiorespiratory complications. To date, studies describing the epidemiology of achondroplasia globally are limited, and vary widely. This study aimed to estimate ACH birth prevalence, both globally and by region, based on a systematic literature review and meta-analysis.

METHODS:

A systematic literature review of PubMed and Embase was conducted to identify all studies reporting ACH birth prevalence across all geographies. Studies were reviewed against exclusion criteria in accordance with PRISMA guidelines. Birth prevalence estimates were standardized to reflect rates per 100,000 live births; studies that combined live births and stillbirths/abortions were adjusted using the live birth-to-total birth ratio (0.82) from a recent study [1], and studies not specifying the population were assumed to reflect live births only. Pooled global birth prevalence and 95% confidence intervals (CI) were estimated using a random-effects meta-analysis, with subgroup analyses by region. Study heterogeneity was assessed using forest plots, and Cochran's Q and I2 statistics.

RESULTS:

The literature review identified 55 studies that met the inclusion criteria. 29 (53%) studies required adjustment to limit the denominator to live births only. Random-effects meta-analysis estimated a pooled ACH global birth prevalence estimate of 3.7 per 100,000 live births (95% CI: 3.1, 4.2) with considerable study heterogeneity (I2 = 79.7%). Pooled birth prevalence estimates varied by region, with studies conducted within the Middle East and North Africa (MENA) reporting the highest estimates (23.1 [95% CI: 13.4, 32.8]) and Northern America the lowest (2.8 [95% CI: 1.8, 3.8]) (Figure 1).

CONCLUSIONS:

Pooled ACH global birth prevalence is estimated at 3.7 per 100,000 live births with notable heterogeneity between studies. This global estimate was lower than a recently published meta-analysis (4.6 per 100,000 births) [2], however this previous study did not distinguish between live births and stillbirths. While data gaps remain in some regions and methodologies vary across studies, these findings provide an updated foundation for understanding global and regional ACH prevalence patterns.

**Figure 1.** Results from Meta-Analyses of Pooled Achondroplasia Birth Prevalence (per 100,000 live births), Overall Global and Sub-Analysis by Region

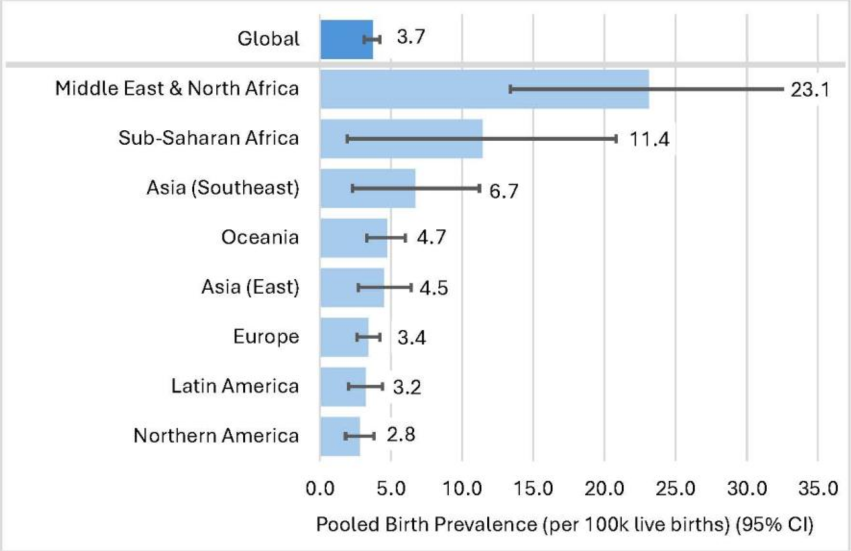

**References:**

1. Coi A, Santoro M, Garne E, Pierini A, Addor MC, Alessandri JL, et al. Epidemiology of achondroplasia: A population-based study in Europe. Am J Med Genet A. 2019;179(9):1791-8. doi: 10.1002/ajmg.a.61289.
2. Foreman PK, van Kessel F, van Hoorn R, van den Bosch J, Shediac R, Landis S. Birth prevalence of achondroplasia: A systematic literature review and meta-analysis. Am J Med Genet A. 2020;182(10):2297-316. doi: 10.1002/ajmg.a.61787.

### Disclosure

RM, DM, CC: Employees of BluePrint Orphan, Inc., which received funding from Ascendis Pharma A/S to support this research. SR, AS: Employees of and may hold stock in Ascendis Pharma A/S

### P69

### Osteogenesis Imperfecta Caused by Ultra-Rare Variants in IFITM5 – New Cases and Mechanistic Studies

Thania Ordaz-Robles[1], Ghalib Bardai[2], Frank Rauch[2,3], Pierre Moffatt[2,3]

[1]Shriners Children's, Mexico, Mexico. [2]Shriners Children's, Montreal, Canada. [3]McGill University, Montreal, Canada

### Abstract

OBJECTIVES:

*IFITM5* codes for the transmembrane protein BRIL that is expressed in osteoblasts. A recurrent variant in the 5’UTR of *IFITM5* (NM_001025295.3; c.-14C>T) leads to osteogenesis imperfecta (OI) type V, which is characterized by hyperplastic callus formation. Other *IFITM5* variants are exceedingly rare, and their mechanistic effects have not been studied. Here we describe the phenotype of three Mexican boys with different missense variants in *IFITM5* and elucidate the associated molecular and cellular defects.

METHODS:

The three patients were followed at the Shriners Hospitals for Children – Mexico. Genomic DNA was analyzed using a 613-gene Skeletal Disorders Panel.

RESULTS:

By 13 years of age, Individual 1 had approximately 30 fractures, mostly affecting femurs (Figure). Femoral rodding was performed bilaterally, and he was able to ambulate thereafter. Height was 135 cm (z-score -3.1). Individual 2 had bilateral deformities of femur, tibia and fibula at birth. By the age of 8 years, he had sustained about 15 fractures. Corrective surgery with intramedullary rodding was performed in both femurs and tibias at 7 years of age and he was able to ambulate with assistance. Individual 3 was 11 years old and had a total of 6 fractures, all in the left femur. At 3 years of age, the left femur deformity was corrected by intramedullary rodding surgery. His height was 132 cm (z-score -1.9). Genetic testing revealed de novo heterozygous variants in *IFITM5* for the three cases, respectively: c.119C>G (p.Ser40Trp), c.119C>T (p.Ser40Leu), and c.143A>G (p.Asn48Ser). When expressed in cultured osteoblasts, all three BRIL protein variants were localized intracellularly in the endoplasmic reticulum-Golgi, but not the plasma membrane. The variants did not induce a MEF2-luciferase signaling readout and had a dominant negative effect when co-transfected with wild type. Biochemical analysis indicated that mutated BRIL was not palmitoylated.

CONCLUSIONS:

All three patients with *IFITM5* missense variants had severe bone fragility, but none had hyperplastic callus. On the molecular and cellular level, the variants were mislocalized and had dominant negative effects.

### Disclosure

None declared

### P70

### Tracing Arthrogryposis in Mexico to a 1,700-Year-Old ECEL1 Founder Variant

Paul McKay[1], Daniel Ramírez-García[2], Sena Tavukcu[1], Ghalib Bardai[1], Anxhela Gjyshi Gustafson[3], Philip Giampietro[4], Noémi Dahan-Oliel[1,5], Frank Rauch[1,5], Thania Ordaz-Robles[2]

[1]Shriners Hospital for Children - Canada, Montreal, Canada. [2]Shriners Children's Mexico, Mexico City, Mexico. [3]Shriners Children's Genomics Institute, Tampa, USA. [4]Shriners Children's Chicago, Chicago, USA. [5]Faculty of Medicine and Health Sciences, Montreal, Canada

### Abstract

OBJECTIVES:

ECEL1-related arthrogryposis (also called distal arthrogryposis type 5D) is a subtype of arthrogryposis multiplex congenita (AMC) characterized by joint contractures predominantly in the distal extremities, ocular features and absence of central nervous system involvement. Previous sequencing studies, focused on individuals from Europe, the Middle East and Australia, found biallelic ECEL1 mutations in 2% to 4% of individuals with AMC. In the present study, we investigated ECEL1-related distal arthrogryposis in a cohort of individuals with AMC from Mexico.

METHODS:

The study cohort comprised 89 children and adolescents with AMC residing in Mexico. Whole-genome sequencing was performed on saliva-derived DNA samples from all participants and their available parents.

RESULTS:

We found that 37 patients (42% of the Mexican AMC study cohort; age range 1 year to 17 years; 15 females) had biallelic variants in ECEL1. Of these, 21 individuals were homozygous for a 46-bp frameshift deletion in ECEL1 (c.110_155del; p.Phe37CysfsTer151). In addition, 8 study participants were compound heterozygous for the 46-bp deletion. Thus the 46-bp frameshift deletion accounted for 50 of the 74 mutant ECEL1 alleles (68%) observed in the cohort. The remaining 34 ECEL1 alleles had 14 different pathogenic or likely pathogenic variants. Based on data from the Mexico City Prospective Study, the estimated carrier rate of the ECEL1 c.110_155del variant is approximately 1/600 in Indigenous Mexican ancestries, which is about 30 times more frequent than the prevalence of the variant in European ancestries. To investigate the origin of the ECEL1 46-bp deletion, we performed runs of homozygosity (ROH) analyses among homozygous individuals to assess evidence for a founder effect. Eighteen of the 21 homozygotes shared a common ROH spanning chr2:232,348,814–232,610,186, a region of 261 kb encompassing the ECEL1 locus (Figure). Based on ROH length, we estimate a time to the most recent common ancestor of approximately 68 generations, or about 1,700 years ago, assuming a mean generation time of 25 years.

CONCLUSIONS:

ECEL1-related distal arthrogryposis is relatively common in Mexico and appears to be caused by a 1,700-year-old founder effect of a 46 bp deletion in this gene.

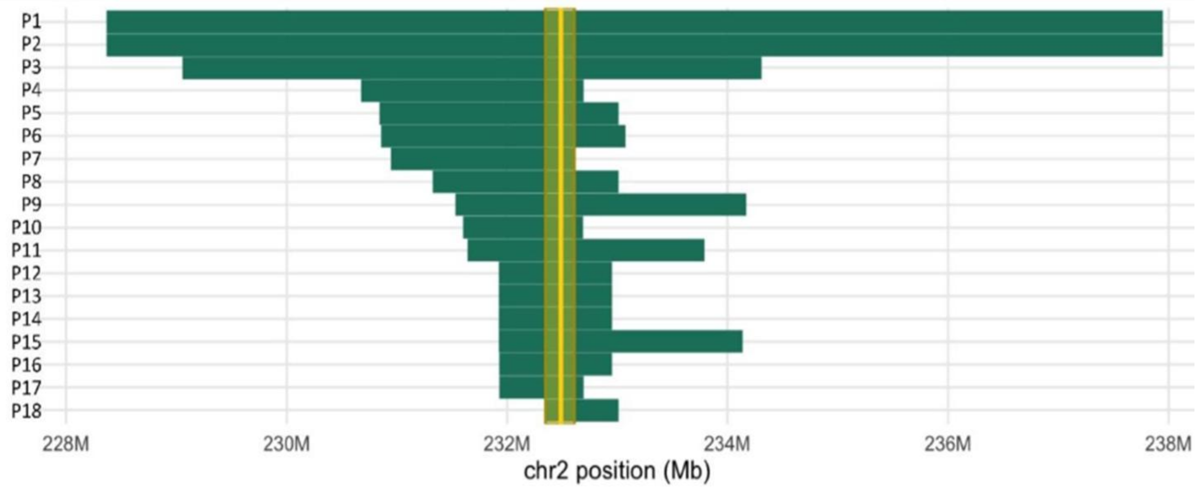

**Figure. Shared run of homozygosity (ROH) among 18 Mexican individuals homozygous for the *ECEL1* c.110_155del variant supports inheritance from a common ancestor.**
Following whole-genome sequencing, ROH were visualized in Integrative Genomics Viewer. ROHs across chromosome 2 are shown in genomic coordinates (Mb). Dark green horizontal bars indicate the homozygous region for each individual. The solid yellow line marks the *ECEL1* locus, while the shaded yellow region denotes the shared 231 kb ROH flanking *ECEL1*, present in all 18 homozygous individuals, consistent with a founder effect.

### Disclosure

None declared

### P71

### Severe Osteogenesis Imperfecta Due to Homozygous Glycine Substitutions in COL1A1 and COL1A2: Expanding the Spectrum Beyond Dominant Inheritance

Alexandra Blaschitz[1,2], Rodrigo Montero-Lopez[1,2], Tamer El-Sobky[3,4], Mostafa M. Barak[3], Hala Abdulhady[5], Heba Dawoud[6], Amira Mobarak[6], Mohamed M.H. El-Sayed[7], Mostafa Elsebai Hammad[7], Gerald Webersinke[8], Theodora Malli[8], Wolfgang Högler[1,2]

[1]Department of Paediatrics and Adolescent Medicine, Johannes Kepler University, Linz, Austria. [2]Centre for Growth and Osteology, Department of Paediatrics and Adolescent Medicine, Kepler University Hospital GmbH, Linz, Austria. [3]Division of Paediatric Orthopedics, Department of Orthopedic Surgery, Faculty of Medicine, Ain-Shams University, Cairo, Egypt. [4]Surgical-based Department, Orthopedic Unit, Faculty of Medicine, Universiti Kuala Lumpur, Royal College of Medicine Perak, Ipoh, Malaysia. [5]Department of Physical Medicine & Rheumatology and Rehabilitation, Faculty of Medicine, Ain Shams University, Cairo, Egypt. [6]Department of Paediatrics, Faculty of Medicine, Tanta University, Tanta, Egypt. [7]Department of Orthopedic Surgery, Faculty of Medicine, Tanta University, Tanta, Egypt. [8]Laboratory for Molecular Genetic Diagnostics, Ordensklinikum Linz, Linz, Austria

### Abstract

**METHODS**:

Within a collaborative study on paediatric bone fragility in Egyptian patients, we performed whole-exome sequencing and segregation analysis in families with suspected OI. Furthermore, we systematically analyzed triple-helical-domain glycine substitutions found in unaffected individuals using gnomAD 4.1.

**RESULTS**:

Two unrelated females with severe OI were identified carrying homozygous glycine substitutions: *COL1A1* (c.1012G>A; p.Gly338Ser) and *COL1A2* (c.1730G>C; p.Gly577Ala). Both presented with OI type III/IV features, including multiple low-impact fractures, long-bone deformities, dentinogenesis imperfecta, scoliosis, and markedly reduced bone mineral density. The heterozygous parents carrying the *COL1A1* variant were asymptomatic, whereas those carrying the *COL1A2* variant had OI type I.

Analysis of triple-helical-domain glycine substitutions found in unaffected individuals in gnomAD revealed 41 *COL1A1* and 122 *COL1A2* heterozygous glycine substitutions. It showed that glycine residues in the N-terminal region of the *COL1A1* triple-helix domain exhibit greater tolerance to substitutions ($\chi^2 = 8.67$, $p = 0.013$) which was not found for *COL1A2* ($p = 0.376$). Most triple-helix-glycine-substitutions involved replacement of glycine with amino-acids with small side chains (serine, alanine, cysteine), as seen in our cases.

**CONCLUSION:**

We report the first homozygous glycine substitution within the triple-helical domain of *COL1A1* associated with severe OI, and the sixth described in *COL1A2*. GnomAD data support the notion that *COL1A1* is less tolerant to glycine substitutions in the triple helix than *COL1A2*. This difference is likely explained by the stoichiometry of type I collagen, in which two α1(I) chains (encoded by *COL1A1*) associate with one α2(I) chain (encoded by *COL1A2*). Consequently, glycine substitutions in *COL1A1* are predicted to exert a greater destabilizing effect on helix formation than substitutions in *COL1A2*.

In summary, glycine substitutions in type I collagen genes are classically dominant and fully penetrant, nevertheless some can cause severe OI with autosomal recessive or double-dominant inheritance patterns, highlighting the variable expressivity and complexity of type I collagen–related disorders.

### Disclosure

WH is principal investigator in clinical trials for Amgen, investigating efficacy and safety of romosozumab in children with osteogenesis imperfecta. AB und RML are sub-investigators investigator in clinical trials for Amgen, investigating efficacy and safety of romosozumab in children with osteogenesis imperfecta. The other authors report no conflict of interest.

### P72

### RNA Analysis of a Novel Splice Variant in *ERGIC1*

Lauren Kerr[1,2], Paul McKay[1,2], Juliana Marulanda[1,3], Zenghui Wu[1], Reggie Hamdy[1], Sena Tavukcu[1], Noémi Dahan-Oliel[1,4], Frank Rauch[1,5]

[1]Shriners Hospital for Children, Montreal, Canada. [2]Department of Human Genetics, McGill University, Montreal, Canada. [3]Faculty of Dental Medicine and Oral Health Sciences, McGill University, Montreal, Canada. [4]School of Physical and Occupational Therapy, McGill University, Montreal, Canada. [5]Department of Pediatrics, McGill University, Montreal, Canada

### Abstract

BACKGROUND:

Arthrogryposis multiplex congenita (AMC) is defined as the presence of joint contractures affecting at least two body regions at birth. Three different *ERGIC1* variants have been reported in individuals with AMC.

PRESENTING PROBLEM:

Here, we report on a 16-year-old male with a homozygous *ERGIC1* c.250+1G>A variant that was classified as a variant of uncertain significance on trio genome analysis. RNA extracted from urine-derived cells showed a splice defect of exon 4. This caused a deletion of two base pairs from the end of the exon, expected to result in a frameshift and nonsense-mediated decay.

CLINICAL MANAGEMENT:

The proband's phenotype resembled that of previously reported individuals with ERGIC1 variants, but also included an absent patella and early puberty, which have not been reported in ERGIC1-related AMC.

DISCUSSION:

This report expands the number of ERGIC1 variants linked to AMC and demonstrates the utility of RNA-based diagnostic methods.

### Disclosure

The authors state that they have the following disclosures: Lauren Kerr: None. Paul McKay: None. Juliana Marulanda: None. Zenghui Wu: None. Reggie Hamdy: None. Sena Tavukcu: None. Noémi Dahan-Oliel: None. Frank Rauch: Angitia, BioMarin, Kirin, Ultragenyx: consulting fees.

### P73

### Multigenic Basis of Adolescent Idiopathic Scoliosis Revealed Through Exome Sequencing

Helene Mathieu, Aurelia Spataru, Stefan Parent, Philippe Campeau, Florina Moldovan

Research Center Azrieli, CHU Sainte justine, Université de Montréal, Montreal, Canada

### Abstract

**Objectives:** Scoliosis is a complex disorder characterized by marked phenotypic heterogeneity and an unknown etiology. Several forms of scoliosis, including Adolescent Idiopathic Scoliosis (AIS), show evidence of a genetic contribution. Epidemiological studies have highlighted the involvement of genetic factors, particularly genes related to ciliary structure and function. The aim of this study was to identify disease-associated variants by performing exome sequencing in affected individuals from multigenerational families.

**Methods:** French-Canadian families were selected based on the presence of at least two members diagnosed with AIS (Cobb angle ≥15°). Exome sequencing was performed on at least two affected individuals per family. Variants were filtered using multiple criteria, including minor allele frequency, over-representation relative to controls, and the Gene Damage Index (GDI). Candidate variants were validated by Sanger sequencing, and their segregation with the phenotype was assessed within each family. To prioritize genes for functional studies, pathogenicity was evaluated using in-silico prediction tools. Given the established involvement of ciliary pathways in AIS, additional analyses were conducted to identify and evaluate variants in ciliary genes. Pathway enrichment was carried out using Panther, and ciliary genes were identified using Gene Ontology annotations.

**Results:** Thirty-seven families with either recessive or dominant inheritance patterns were analyzed. Exome sequencing yielded over 253,425 variants per family. Filtering, co-segregation validation, and pathogenicity prediction allowed us to refine the list to seven candidate genes: **FN1, VLDLR, CA4, GSL2, OGFR, PTGS1**, and **SIPA1L3**. Pathway analysis revealed that 28% of identified variants were associated with organelle-related functions, including 17 variants located in genes involved in ciliary biology.

**Conclusion:** The identified genes are strong candidates for involvement in AIS and may play important roles in spine development by influencing bone and cartilage morphogenesis. Our findings reinforce the contribution of ciliary pathways to AIS and support the multigenic nature of the disorder. A SKAT-O analysis is underway to further examine the combined effects of these variants. Functional validation studies will be conducted *in vitro* using patient-derived cellular models and *in vivo* using animal models such as zebrafish. Identifying causative genes will contribute to a deeper understanding of AIS etiopathogenesis and may ultimately help guide targeted therapeutic strategies.

Supported by Fondation Yves Cotrel- Institut de France

### Disclosure

None declared

### P74

### Integrating Genetic Counselling into a Multidisciplinary Pediatric Bone Health Clinic: A Descriptive Evaluation

Valancy Miranda, Marie-Josée Giguère, Marie-Ève Robinson, Anne Marie Sbrocchi, Frank Rauch

Shriners Hospital for Children-Canada, Montreal, Canada

### Abstract

OBJECTIVES:

The metabolic and genetic bone disorders clinic at Shriners Hospital for Children–Canada provides multidisciplinary bone health care for children. Although many of these disorders are of genetic origin, the clinic traditionally did not include a genetic counsellor. A part-time genetic counsellor joined the team in November 2020. Here we quantify the contributions of genetic counselling within the clinic.

METHODS:

We evaluated genetic counselling activities in a quaternary pediatric bone health clinic for the years 2021 to 2025.

RESULTS:

The genetic counsellor attended clinic two days per week. Counselling activities varied according to patient needs. For patients with suspected genetic conditions, the genetic counsellor obtained detailed medical and family histories and delivered comprehensive pre-test counselling, including informed consent (431 individuals, 250 families). Post-testing, the genetic counsellor communicated genetic test results and provided counselling, ensuring consistent follow-up while substantially reducing the workload for physicians and nurses (606 individuals, 368 families; post-test counselling was more frequent than pre-test counselling, as the counsellor was not present on all clinic days when genetic testing was offered to patients). For families with confirmed diagnoses (N = 86 families), the genetic counsellor provided education on disease etiology and inheritance, offered preconception counselling including reproductive options, and coordinated familial screening. The number of individuals and families who received genetic counselling services of any kind increased more than two-fold during the observation interval (Figure) and overall represented 38% of patients who were seen in the clinic during the observation period. The relative frequency of suspected diagnostic categories of patients who received genetic counselling services were osteogenesis imperfecta/bone fragility (35%), skeletal dysplasia (29%), connective tissue disorders (20%), mineralization defects (9%) and other (7%).

CONCLUSIONS:

Genetic testing is an increasingly important aspect of pediatric bone health clinics. Integrating a genetic counsellor into a multidisciplinary pediatric bone health clinic streamlined genetic testing processes, improved interpretation and communication of results, and enhanced family-centred care by addressing a previously unmet need for dedicated genetic counselling within the care team.

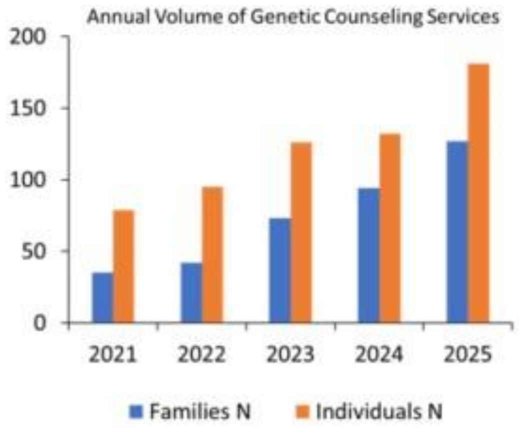

Figure. Number of individuals and families who received genetic counselling services in the Pediatric Bone Health Clinic from 2021 to 2025.

### Disclosure

None declared.

### P75

### Papillary Thyroid Carcinoma in a Child with a CDC73 Mutation: Association or Causation?

Munibah Bashir[1], James Blackburn[1], Charlotte Jarvis[1], Suzanne Armitage[2], Neil Houghton[3], Samantha Goh[4], Francesca McDowell[5], Renuka Ramakrishnan[1]

[1]Department of Paediatric Endocrinology, Alder Hey Children's Hospital, liverpool, UK. [2]Department of Biochemistry, Alder Hey Children's Hospital, liverpool, UK. [3]Department of Endocrine Surgery, Liverpool University Foundation Trust., liverpool, UK. [4]Ear, Nose & Throat Department, Alder Hey Children's Hospital, liverpool, UK. [5]Department of Paediatric Histopathology, Alder Hey Children's Hospital, liverpool, UK

### Abstract

**Background:**

Papillary thyroid carcinoma (PTC) is uncommon in childhood, although it is the most frequent thyroid malignancy in this age group. Although PTC has been reported in adults with CDC73 mutations, there are no previous reports of this occurring in children.

**Presenting Problem:**

An asymptomatic 15-year-old male under surveillance for unilateral pelvi-ureteric junction obstruction was found to have persistent hypercalcaemia and primary hyperparathyroidism.

**Investigations:**

| | |
|---|---|
| Adjusted Ca | 3.08 mmol/L (2.15-2.74) |
| PTH | 26.4 pmol/L (1.1-6.9) |
| Urine calcium:creatinine ratio | 1.13 mmol/mmol (<0.60) |
| USS neck & Tc-99m sestamibi | Localized parathyroid adenoma |
| Familial hyperparathyroidism gene panel | Heterozygous pathogenic CDC73 variant |
| Jaw X-ray | Normal |
| Family Genetic Panel | Negative |

Tumour panel testing of thyroid identified the germ line pathogenic CDC73 mutation; but a second somatic mutation wasn't identified. A parathyroid adenoma gene panel is awaited.

### Clinical Management:

He underwent a right sided neck exploration for parathyroid adenoma excision. Due to central neck lymphadenopathy and his genetic risk, a right hemithyroidectomy and central neck dissection was also performed. Histology confirmed a right parathyroid adenoma with metastatic PTC in the lymph nodes. The patient subsequently underwent completion of thyroidectomy which demonstrated multiple microscopic foci of PTC throughout the specimen.He then received radioiodine therapy. Postoperatively, he recovered well and is on replacement medications.

**Discussion:**

PTC accounts for 90% of childhood thyroid cancers. Associated gene mutations include *RET, BRAF, RAS, NTRK, DICER1, TP53,* and *PTEN.*

*CDC73* mutations are autosomal dominant, at times arising de novo as in our patient. CDC73 related disorders include HPT-JT syndrome, familial isolated hyperparathyroidism (FIHP), and parathyroid carcinoma. Presentation often occurs in late adolescence or early adulthood with a single parathyroid adenoma. This condition predisposes to tumours in various sites including the jaw bones, kidneys, uterus and ovaries. Our case offers a rare and valuable insight into the potential role of CDC73 mutations in paediatric thyroid cancer.

Although there are no established national guidelines for management of patients with *CDC73* mutations, studies recommend offering genetic testing to parents and first-degree relatives and regular biochemical and radiological screening to the affected members.

The presence of PTC raises the question of whether this represents a coincidental finding or reflects a broader tumour predisposition related to *CDC73* dysfunction. There may be a second somatic CDC73 variant that remained undetected by the screening protocol used.

### Disclosure

None declared

### P76

### Expanding the FBN1 Spectrum Beyond Marfan: Overlapping Weill–Marchesani and Stiff Skin Syndromes

Amna Ansari[1], Shua Basalom[2]

[1]McGill University, Montreal, Canada. [2]CHU du Quebec, Quebec city, Canada

### Abstract

**Background:**
*FBN1* (OMIM 134797) encodes fibrillin-1, an extracellular matrix glycoprotein essential for microfibril structure and tissue elasticity in cardiovascular, skeletal, ocular, and dermal tissues. Pathogenic variants cause a spectrum of connective tissue disorders known as fibrillinopathies. While Marfan syndrome (MFS) is classically associated with tall stature, joint laxity, ectopia lentis, and progressive aortic dilation, other *FBN1*-related disorders such as Weill–Marchesani syndrome (WMS; OMIM 608328**)** present with nearly opposite features including short stature, brachydactyly, joint stiffness, and microspherophakia. Stiff skin syndrome (SSS; OMIM 184900) represents another rare *FBN1* phenotype characterized by skin thickening and induration with progressive joint contractures. Both WMS and SSS are exceptionally rare, each reported in only a small number of families worldwide, with true prevalence unknown but estimated to be around or below 1 per million.

**Presenting Problem:**
A 5-year-old boy presented with motor delay, hip and elbow contractures, macrocephaly (>97th percentile), plagiocephaly, micrognathia, myopia, microspherophakia, eczema, asthma, and recurrent otitis media. Echocardiography showed mild mitral regurgitation without aortic root dilation. His mother had short stature, brachydactyly, joint stiffness, microspherophakia, and thick, indurated skin of the hands with flexion contractures, without aortic enlargement. These findings suggested overlapping WMS and SSKS phenotypes. There was no family history of Marfan syndrome.

**Clinical Management:**
Copy number variant analysis identified a heterozygous 20.3 kb *FBN1* deletion encompassing exons 10–15, resulting in in-frame loss of 282 amino acids. This variant is absent from population databases and has not been previously reported. Maternal testing confirmed inheritance. The variant was classified as likely pathogenic. Both patients are undergoing multidisciplinary surveillance including cardiology, ophthalmology, orthopedics, dermatology, and genetics follow-up, with counseling regarding autosomal dominant inheritance and variable expressivity.

**Discussion:**
This family demonstrates striking intrafamilial variability within the acromelic and cutaneous spectrum of FBN1-related disease, combining features of WMS (short stature, brachydactyly, microspherophakia) and SSS (skin induration, joint contractures). Recognition of these blended, “opposite” fibrillinopathy presentations broadens the phenotypic spectrum beyond classic MFS and is essential for accurate diagnosis, anticipatory guidance, and surveillance.

### Disclosure

None declared

### P77

### Looking Beyond the Spine: Early-Onset Scoliosis as a Marker of Rare Genetic Disease

Elise Nauwynck, Nathalie Smeets, Boyan Dimitrov, Aurélie Andrzejewski, Eva De Wachter, Nils Goovaerts, Caroline Ernst, Jean De Schepper

UZ Brussel, Jette, Belgium

### Abstract

### Background

Early-onset scoliosis (EOS) is often idiopathic but may be related to connective tissue and bone disorders, as well as neurological and muscular disorders. Given its marked etiological heterogeneity, careful evaluation of family history, dysmorphic features, growth failure or acceleration and developmental delay is essential. We present three cases of EOS where specific clinical features guided the identification of distinct underlying genetic diagnoses.

### Presenting problem

Case 1 concerned a 10-month-old girl referred for scoliosis, first noted when she began sitting independently at 7 months. Pregnancy and delivery were uneventful, and there was no family history of scoliosis. She showed mild motor delay and tall stature (+3.1 SDS). Clinical examination revealed right-sided thoracolumbar scoliosis and dysmorphic features, including arachnodactyly, abnormal pinnae, and pectus carinatum; similar features were observed in her mother and older sister. Full-spine radiography confirmed mild dextroconvex thoracolumbar scoliosis (Cobb angle 14°) as well as ovoid vertebral bodies. A skeletal dysplasia gene panel identified a heterozygous class 4 variant p.(Cys1384Tyr) in FBN2, maternally inherited, consistent with congenital contractural arachnodactyly (Beals syndrome). Case 2 involved a 2-year-7-month-old boy with delayed motor development and rather short stature (−1.6 SDS). Clinical examination showed thoracic kyphosis without other dysmorphic features. Imaging revealed vertebral anomalies at L1–L2 and lumbosacral spina bifida occulta. His father had scoliosis and marked short adult stature. Genetic testing identified a heterozygous class 4 variant p.(Cys123Tyr) in FN1, also present in the father, consistent with spondylometaphyseal dysplasia, corner fracture type. Case 3 was a 1-year-7-month-old boy with global developmental delay, severe bilateral foot paresis, and absent Achilles reflexes. Family history was negative for neuropathy and scoliosis. Clinical and radiographic evaluation showed moderate thoracolumbar scoliosis (Cobb angle 28.7°) and pectus excavatum. A neuromuscular gene panel revealed compound heterozygous class 5 variants p.(Cys496*) and p.(Arg790*) in IGHMBP2, consistent with Charcot–Marie–Tooth disease type 2.

### Discussion

A multidisciplinary approach involving pediatric orthopedics, neurology, endocrinology, and clinical genetics is crucial in EOS to ensure accurate diagnosis, which is needed for prognostic evaluation and optimal management.

### Disclosure

None declared

### P78

### Continuous Subcutaneous PTH (1-34) Infusion in Severe ADH1: A Decade of Experience in Children and Young Adults

Aikaterini Perogiannaki[1,2], Mohammad Meshari Alattar[2], Rebecca J Gorrigan[3], Debbie Pullen[4], Oladimeji Smith[5], Sailesh Sankaranarayanan[6], Evelien Gevers[1,2]

[1]Centre for Endocrinology, William Harvey Research Centre, Barts and the London Medical School, Queen Mary University of London, London, UK. [2]Department of Paediatric Endocrinology, Royal London Hospital, Barts Health NHS Trust, London, UK. [3]Department of Endocrinology, Royal London Hospital, Barts Health NHS Trust, London, UK. [4]Department of Paediatrics, East Surrey Hospital, Surrey and Sussex Healthcare NHS Trust, Redhill, UK. [5]Department of Paediatrics, William Harvey Hospital, East Kent Hospitals University NHS Foundation Trust, Ashford, UK. [6]Department of Endocrinology, Coventry Hospital, University Hospitals Coventry and Warwickshire, Coventry, UK

### Abstract

### Objectives

Autosomal dominant hypocalcaemia type 1(ADH1) is a rare disorder of calcium homeostasis caused by activating variants in the calcium-sensing receptor gene. Conventional therapy with alfacalcidol and calcium often fails to maintain normocalcaemia in severe disease. Continuous subcutaneous parathyroid hormone (1-34) infusion(CSPI) offers an alternative, though long-term outcomes remain unclear. We previously published outcomes from the first 3.2±0.6 years of treatment. This study presents extended follow-up in one adult and four children treated with CSPI at a single centre.

### Methods

Data were collected from five patients with ADH1 receiving CSPI as routine care for 6.8-9.7 years. Children started CSPI at 1-10 months of age. Most patients also received magnesium and vitamin D supplementation. Monitoring included blood tests 2–3 weekly and clinic review 4–6 monthly. Outcomes assessed were serum calcium, phosphate, magnesium, renal function, urinary calcium, seizures, admissions, growth, bone mineral density, and nephrocalcinosis over a mean 8.9±1.2 years (December 2018-December 2024).

### Results

Mean adjusted calcium remained within or near target in all patients, although intermittent hypo- and hypercalcaemia occurred. Phosphate was often elevated in paediatric patients. 24-hour urinary calcium excretion was normal in all cases. Creatinine was mildly elevated in three patients, likely secondary to polyuria and inadequate fluid intake (Table 1). Admissions for calcium disturbances were infrequent, and hypocalcaemic seizures occurred once in two patients. PTH dose requirements varied between and within patients, with most recent doses ranging 0.28-0.68 mcg/kg/day.

Growth and neurodevelopment were normal in children, whereas the adult who started treatment in adulthood was short and suffered from anxiety. Bone mineral apparent density remained within the age-adjusted reference range. Nephrocalcinosis remained stable in four patients and mildly progressed in one, without significant renal impairment. Treatment emergent adverse events included autoimmune hypothyroidism(P1), gastrointestinal symptoms(P2), anxiety/depression(P3), central precocious puberty and enamel defects(P5). It is difficult to determine whether these were related to CSPI, *CASR* activation, or coincidental comorbidity.

### Conclusions

CSPI was effective and well tolerated long-term, providing sustained biochemical control without severe adverse effects. It represents a viable option for severe ADH1 refractory to conventional therapy, though ongoing bone density monitoring is recommended.

| | P1 | P2 | P3 | P4 | P5 |
|---|---|---|---|---|---|
| **Sex** | Female | Male | Female | Male | Female |
| **Age at start of CSPI** | 5 wk | 10 mo | 22 yr | 7 wk | 11 wk |
| **Current age** | 9 years | 10 years | 31 years | 10 years | 7 years |
| ***CASR* variant** | F821L | T828N | T828N | Y829C | A843E |
| **Urea (NR 2.5-6.5 mmol/L)** | 5.4 (4.4) n=47 | 4.2 (0.6) n=11 | 5.2 (1.5) n=65 | 4.0 (1.1) n=15 | 5.1 (1.6) n=216 |
| **Creatinine (umol/L)** | 32.9 (4.7) n=46 96% in NR | 48.6 (5.1) n=11 0% in NR | 93.5 (6.8) n=65 7.7% in NR | 37.0 (5.1) n=47 93.6% in NR | 38.3 (5.9) n=216 17.8% in NR |
| **Adjusted Calcium (TR 1.8-2.5 mmol/L)** | 2.13 (0.35) n=288 | 2.13 (0.31) n=181 | 1.91 (0.28) n= 80 | 2.15 (0.19) n=70 | 2.14 (0.24) n=241 |
| **Phosphate (NR 0.9-1.8 mmol/L)** | 1.82 (0.31) n=286 | 2.12 (0.32) n=158 | 1.29 (0.2) n=22 | 2.08 (0.18) n= 59 | 2.04 (0.27) n=240 |
| **Magnesium (NR 0.7-1 mmol/L)** | 0.71 (0.06) n=36 | 0.69 (0.07) n=13 | 0.65 (0.11) n=38 | 0.74 (0.03) n=61 | 0.62 (0.17) n= 174 |
| **Osmolality Serum (NR 275-295 mmol/kg)** | 289.4 (5.1) n=12 | 288.7 (3.4) n=10 | | 287.8 (5.1) n=9 | 288.8 (5.1) n=31 |
| **Serum Aldosterone (NR 250-950 pmol/L upright)** | 660.6 (289.4) n=10 | 1111.6 (347) n=8 | | 340 (145.1) n=6 | 615 (314.7) n= 22 |
| **Renin Activity (nmol/L/h)** | 3.6 (1.6) n=11 | 2.2 (1.1) n=9 | | 3.1 (1.5) n=6 | 2.4 (1.0), n=17 |
| **Adjusted Calcium Phosphate Product mmol2/L2 (NR<5)** | 3.8 (0.6) n=286 | 4.5 (0.5) n=158 | 2.4 (0.4) n=22 | 4.4 (0.7) n=59 | 4.3 (0.6) n=239 |
| **Urine Calcium: Creatinine Ratio (NR<0.7)** | 1.2 (0.7) n=41 | 0.8 (0.3) n=10 | 0.5 (0.2) n=6 | 0.5 (0.2) n=11 | 0.5 (0.3) n=10 |
| **24hr(U)Ca** | 4.8 (0.9) n= | 2.2 (0.7) n=3 | 2.8 (0.9) n=2 | 1.7 (0.8) n=4 | 1.5 (n=1) |

| | | | | | |
|---|---|---|---|---|---|
| **(NR<7.5 mmol/d)** | 6 | | | | |

Data presented in mean (SD) n=number of measurements. NR= normal range, TR=target range

NR creatinine: 45-84 (adult), 29-56 (>10yo), 30-47 (8-10 yo), 25-42 (6-8yo), 23-37 (4-6 yo), 15-31 (2-4yo), 14-34 (<2yo) umol/L

NR renin activity: <8nmol/L/hr (1-6years), 0.5-3.5nmol/L/hr (>8years)

### Disclosure

None declared

### P79

### Endochondral Bone Growth with Weekly BMN 333, a Long-Acting Vosoritide Investigational Product, in Juvenile Nonhuman Primates

Heather Wenzel, Jeremy Arens, Madhav Sachar, Russell Soon, Huiyu Zhou, Daniel Wendt, Yulan Qi

BioMarin Pharmaceutical Inc., Novato, CA, USA

### Abstract

**OBJECTIVES:**

BMN 333 is an investigational, fully biodegradable treatment designed to extend exposure to vosoritide, its active moiety. This study evaluated the effects of weekly BMN 333 administration on bone growth in juvenile cynomolgus monkeys and compared growth outcomes with those previously observed following daily vosoritide dosing in the same model and treatment duration. Juvenile cynomolgus monkeys represent a rapidly growing model, providing a stringent context in which to assess growth effects of CNP–based therapies.

**METHODS:**

Male and female 2-year-old cynomolgus monkeys (n=52) received subcutaneous vehicle or BMN 333 (75, 200, or 350 µg/kg once weekly [QW]) for 26 weeks. Tibial and femoral lengths were measured radiographically at baseline and during treatment. Annualized growth rates were calculated from baseline to Week 24. Bone microarchitecture and growth plate morphology were assessed using computed tomography and histomorphometry. Growth outcomes were compared with historical data from a 26-week study of daily vosoritide (90 or 300 µg/kg/day) in age-matched cynomolgus monkeys.

**RESULTS:**

BMN 333 produced dose-dependent increases in longitudinal bone growth. Tibial and femoral length increased significantly at 350 µg/kg QW in both sexes, while femoral length increased significantly at ≥ 200 µg/kg QW in females. Annualized growth rates for both bones were also significantly increased at the highest dose in both sexes. These increases were associated with transient widening of the growth plate and increases in metaphyseal bone area consistent with enhanced endochondral ossification. There were no adverse effects observed at any dose.

Despite lower weekly vosoritide exposure with BMN 333 compared with daily vosoritide administration, BMN 333 at 350 µg/kg QW achieved tibial growth comparable to that produced with 90 µg/kg/day vosoritide and femoral growth that was similar to or exceeded results observed with 300 µg/kg/day vosoritide over the same 26-week treatment period in this model.

**CONCLUSIONS:**

Weekly BMN 333 administration for 26 weeks resulted in sustained, dose-dependent increases in endochondral bone growth in juvenile monkeys. Growth achieved with BMN 333 was comparable to or exceeded results observed with higher daily doses of vosoritide without adverse effects or apparent saturation of the growth effect with sustained CNP exposure.

### Disclosure

All authors are employees of and shareholders of BioMarin Pharmaceutical Inc.

**P80**

Abstract withdrawn

### P81

### To Evaluate the Utility of Bisphosphonate Treatment in Halting Disease Progression and Improving Long-Term Outcomes in Patients with Steroid-Induced Avascular Necrosis (AVN) Following Treatment for Acute Lymphoblastic Leukemia (ALL)

Vrushali Takalikar[1], Leo Donnan Donnan[1], Peter Simm[1], Margaret Zacharin[2]

[1]Royal Childrens' Hospital and Murdoch Childrens' Research Institute, Melbourne, Australia. [2]Royal Childrens' Hospital and Murdoch Childrens' Research Institute, Melbourne, Austria

### Abstract

**Objectives:**
To evaluate the utility of bisphosphonate treatment in halting disease progression and improving long-term outcomes in patients with steroid-induced avascular necrosis (AVN) following treatment for acute lymphoblastic leukemia (ALL).

**Methodology:** This retrospective study of ALL survivors treated at the Royal Children's Hospital, Melbourne, was conducted after obtaining institutional ethical approval. Data was obtained from hospital records. Those who developed steroid-induced AVN, treated with bisphosphonates, were identified. Clinical characteristics, extent of joint involvement, duration of bisphosphonate treatment and long term outcomes were assessed. Pain was assessed retrospectively by its impact on activities of daily living (ADL) at first visit and at the end of treatment and graded using a pre-specified four-point ordinal scale. [1]as follows:
0 = no limitation, 1 = limitation of sports only, 2 = limitation of school routine/need to avoid stairs or prolonged walking, 3 = limitation of basic ADL and/or requirement for mobility aid.

**Results:** Of the twenty ALL survivors with AVN (12 females) treated with bisphosphonates, all but 2 (who relapsed and expired before completion of treatment) were included. Multiple subchondral osteonecrosis was observed in all but three patients. Sixteen patients received zoledronic acid (0.05 mg/kg/dose, administered six-monthly) for 2 years, while two received pamidronate (1 mg/kg/dose, administered three-monthly) for 4 years.Click or tap here to enter text.Mean follow-up duration was 5.3 years. Apart from typical acute-phase reactions following the first dose, no major adverse effects were observed. Pain assessment was available for 15/18.[1] [2]

Following initiation of bisphosphonate, necrotic changes in affected subchondral regions remained largely radiologically stable, with AVN progression in only two patients and confined to only one subchondral region. Nine of 15 reported no limitation of movement or activities (fall in ADL score from 3/2 to 0), 5 reported reduction in pain to solely limitation in sporting activity and minimal analgesic use (fall in ADL score from 3/2 to 1). Of five patients requiring single joint replacement, three had joint collapse and structural distortion at initial presentation.

**Conclusion:** Bisphosphonate treatment for AVN in ALL survivors was associated with sustained pain control, improved functional activities, stabilization of radiological changes and reduced need for major orthopedic interventions during follow-up.[2]

References-

1. 1. Haase I. Accuracy of retrospective pain measurement in patients with chronic pain. Medicine International [Internet]. 2023 Jul 4 [cited 2026 Jan 30];3(4):35. Available from: https://pmc.ncbi.nlm.nih.gov/articles/PMC10336923/

2.. 2. Daneshdoost SM, El Abiad JM, Ruble KJ, Jones LC, Crane JL, Morris CD, et al. Bisphosphonate Therapy for Treating Osteonecrosis in Pediatric Leukemia Patients: A Systematic Review. J Pediatr Hematol Oncol. 2021 Apr 1;43(3):e365–70.

**Table 1- Characteristics of ALL survivors affected with acute vascular necrosis (AVN)**

| Sr. No | Sex | Joints involved | Number of joints involved | Orthopedic intervention | Limitation of activity at initial visit | Limitation of activity at the end of treatment |
|---|---|---|---|---|---|---|
| 1 | M | Both knees and hips | 4 | No | 2 | 0 |
| 2 | M | Both hips | 2 | No | 2 | 0 |
| 3 | M | Left ankle | 1 | No | 2 | 0 |
| 4 | M | Left hip | 1 | No | 2 | 1 |
| 5 | F | Both shoulders, hips, knees and left ankle | 7 | Right hip replacement and shoulder drilling | NA | NA |
| 6 | F | Both ankles | 2 | No | 2 | 0 |
| 7 | F | Both knees | 2 | No | 2 | 0 |
| 8 | F | both knees, hips, shoulders | 6 | Right THR | 2 | 1 |
| 9 | M | Both hips and knees | 4 | Left hip replacement in 2019 | 3 | 2 |
| 10 | F | Both hips, left knee, left ankle | 4 | Needs orthotics for ankles | 3 | 1 |
| 11 | M | Both hips | 2 | No | 2 | 0 |
| 12 | F | Both ankles | 2 | Right THR | 3 | 1 |
| 13 | F | Both shoulders, both hips, left knee | 5 | No | 2 | 1 |

| | | | | | | |
|---|---|---|---|---|---|---|
| 14 | M | Both hips | 2 | No | 3 | 0 |
| 15 | F | Both knees | 2 | No | 2 | 0 |
| 16 | F | Both hips and knees | 4 | No | NA | NA |
| 17 | F | Both hips, both knees, right ankle | 5 | Right THR | NA | NA |
| 18 | F | Both hips, knees and ankles | 6 | No | 2 | 0 |

**Disclosure**

None declared

### P82

### Comparison of Peripheral Quantitative Computed Tomography Bone Mineral Density Measurements of Vosoritide-Treated Children with Achondroplasia to an Average-Stature Reference Population

Klaus Engelke[1,2,3], Thomas Fuerst[4], Cheng-Chia (Kathy) Tang[5], Andrea Low[5], Jeanette White[5]

[1]Department of Medicine 3, Friedrich-Alexander-University Erlangen-Nürnberg and Universitätsklinikum Erlangen, Erlangen, Germany. [2]Institute of Medical Physics, Friedrich-Alexander-University Erlangen-Nürnberg, Erlangen, Germany. [3]Clario Inc., Estenfeld, Germany. [4]Clario Inc., Philadelphia, USA. [5]BioMarin Pharmaceutical Inc., Novato, USA

### Abstract

**OBJECTIVES:** Achondroplasia is a skeletal dysplasia that impairs endochondral bone growth. Vosoritide, a C-type natriuretic peptide analog that stimulates bone growth, is the first approved precision therapy for children with achondroplasia. Previously, we demonstrated that long-term vosoritide treatment in children with achondroplasia was associated with increased radial and tibial bone growth parameters using peripheral quantitative computed tomography (pQCT). However, lack of a control arm limited the ability to differentiate changes in bone parameters due to expected growth from treatment effect. Here, we compare our pQCT radius and tibia parameter results to published age- and sex-stratified reference curves from an average-stature population (Jaworski M, et al. 2018 and 2021).

**METHODS:** Thirty participants (13 males, 17 females), aged ≥5 to <15 years, who completed up to 10 years of vosoritide treatment in the phase 2 dose-escalation (CANOPY ACH-2, NCT02055157) and extension (CANOPY ACH-EXT, NCT02724228) studies were included. pQCT scans were acquired at least annually with whole-body clinical CT scanners. At baseline, distal and proximal volumes of interest (VOIs) were positioned in the radius and tibia. To account for growth during follow-up, affine registration based on bone length changes was applied. For each VOI, bone mineral density (BMD) of the integral, trabecular, and cortical compartments was determined, and individual participant data were plotted against average-stature references.

**RESULTS:** Participants receiving long-term vosoritide showed longitudinal increases from baseline in BMD and geometrical parameters. Key pQCT parameters, such as integral and trabecular BMD, increased with age for most participants (**Figure**). Due to different anatomical positions of the VOIs analyzed in this study compared with those used for the reference population, absolute BMD values could not be directly compared; however, increases were largely comparable to the age-related trends observed in the reference population.

**CONCLUSIONS:** We demonstrate that the increase in BMD with age in children with vosoritide-treated achondroplasia mostly followed the same trend as the average-stature population, implying similar trajectories. Along with the increase in bone length (data not shown), this study is the first to demonstrate sustainable endochondral bone growth over time via pQCT for children with achondroplasia receiving vosoritide.

**Figure.** Representative overlays of QCT parameters for the distal tibia comparing individual female study participants with vosoritide-treated achondroplasia to an average-stature population. **(A)** Integral and **(B)** trabecular volumetric BMD

**A.**

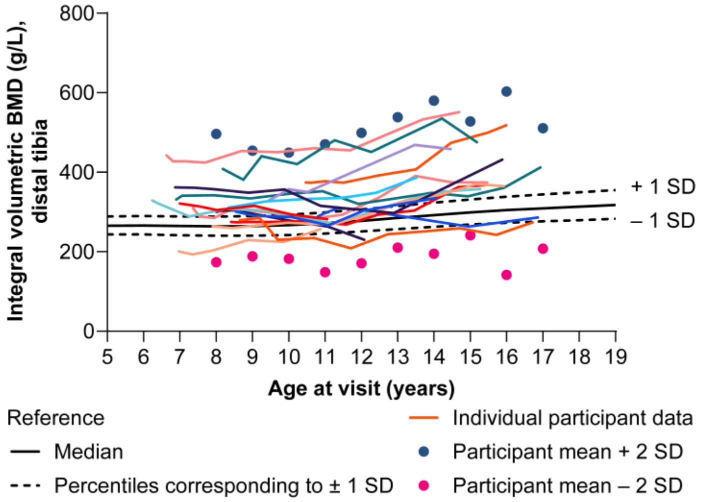

**B.**

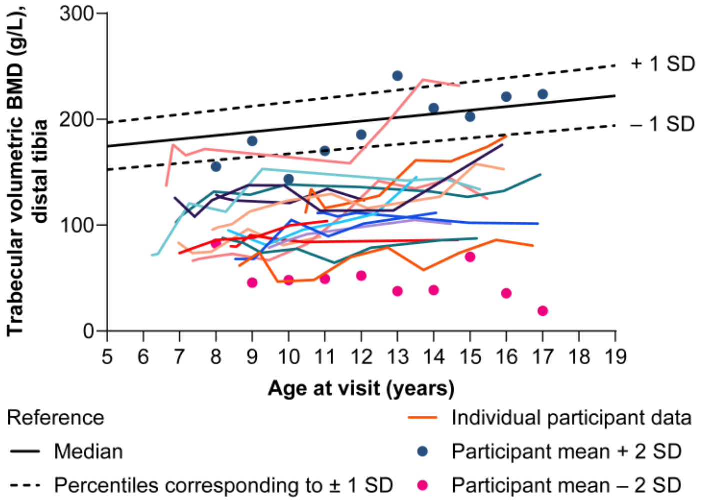

Colored lines represent individual participant QCT measurements during the study period. Results for the distal tibia in males and for the distal radius of females and males were similar. Reference curves (4% VOI; depicted as solid black line) for the average-stature pediatric population are based on median QCT measurements of the tibia (Jaworski M, et al. *J Musculoskelet Neuronal Interact*. 2021;21(2):215–236) or the radius (Jaworski M, et al. *J Musculoskelet Neuronal Interact*. 2018;18(2):237–247).

BMD, bone mineral density; QCT; quantitative computed tomography; SD, standard deviation; VOI, volume of interest.

### Disclosure

KE and TF are employees of Clario Inc., which was contracted by BioMarin Pharmaceutical Inc. to perform this analysis. C-CT, AL, and JW are employees and potential stockholders of BioMarin Pharmaceutical Inc.

### P83

### Long-Term Impact of Vamorolone on Bone Health Compared to Standard of Care Glucocorticoids (SoC-GC) in Boys with Duchenne Muscular Dystrophy (DMD)

Michela Guglieri[1], Anne-Marie Childs[2], Liesbeth De Waele[3,4], Ekaterina Gresko[5], Jana Haberlova[6], Shabir Hasham[5], Jacob Jaremko[7], Marina Katsalouli[8], Khaldoun Koujok[9], Mika Leinonen[5], Aki Linden[5], Laura McAdam[10], Craig McDonald[11], Hugh McMillan[9], Eugenio Mercuri[12,13], Francesco Muntoni[14,15], Yoram Nevo[16,17], Erik Niks[18], Leanne Ward[9]

[1]The John Walton Muscular Dystrophy Research Centre, Newcastle University and Newcastle Hospitals NHS Foundation Trust, Newcastle upon Tyne, UK. [2]Leeds Teaching Hospitals NHS Trust, Leeds, UK. [3]University Hospitals Leuven, Child Neurology, Leuven, Belgium. [4]KU Leuven, Department of Development and Regeneration, Leuven, Belgium. [5]Santhera Pharmaceuticals, Pratteln, Switzerland. [6]Department of Pediatric Neurology, Second Faculty of Medicine, Charles University and Motol University Hospital, Prague, Czech Republic. [7]University of Alberta, Edmonton, Canada. [8]Pediatric Neurology Department, St. Sophia's Children's Hospital, Athens, Greece. [9]Children's Hospital of Eastern Ontario, University of Ottawa, Ottawa, Canada. [10]Holland Bloorview Kids Rehabilitation Hospital, Bloorview Research Institute, University of Toronto, Toronto, Canada. [11]University of California Davis Health, Davis, USA. [12]Catholic University of Sacred Heart, Rome, Italy. [13]Fondazione Policlinico Universitario Agostino Gemelli IRCCS, Rome, Italy. [14]Dubowitz Neuromuscular Centre, Great Ormond Street Institute of Child Health, London, UK. [15]NIHR Great Ormond Street Hospital Biomedical Research Centre, London, UK. [16]Faculty of Medicine, Tel Aviv University, Tel Aviv, Israel. [17]Institute of Pediatric Neurology, Schneider Children's Medical Center of Israel, Petach Tikva, Israel. [18]Department of Neurology, Leiden University Medical Center, Leiden, Netherlands

### Abstract

**OBJECTIVES:** Boys with DMD on standard of care glucocorticoids (SoC-GC) have high vertebral fracture (VF) risk and bone age delay. Indirect comparison of VF and bone age delay in DMD boys after long-term treatment with vamorolone vs SoC-GC.

**METHODS:** Baseline data from 37 patients enrolled in GUARDIAN were used for propensity score matching with a SoC-GC cohort (N=60), previously published (Phung K & Ward LM. Osteoporosis Int. 2023 Jan; mostly on daily deflazacort). VF were evaluated on lateral spine x-rays according to the Genant semi-quantitative methodology, the Spinal Deformity Index (SDI) was calculated (sum of the Genant grades), and bone age was evaluated by BoneXpert™ on hand X-rays.

**RESULTS:** At the time of the X-rays, mean ±SD ages in vamorolone (N=37) and matched GC (N=31) cohorts were 10.9±1.25 and 11.7±1.53 years. Mean drug exposure was 5.2±0.95 years for vamorolone (190.6 patient-years) vs 5.4±2.06 years on SoC-GC (167.5 patient-years). Mean doses at the time of X-ray were 4.89±1.28 mg/kg/day and 0.65±0.21 mg/kg/day for vamorolone and deflazacort respectively. 3/37 (8.1%) patients had 9 VF on vamorolone vs 13/31 (41.9%) with 30 VF on SoC-GC (Odds Ratio (95%CI) 0.1296 (0.02903, 0.5789), p=0.0082). The mean SDI for subjects with and without VF was lower on vamorolone vs SoC-GC (0.32 vs 1.06). In vamorolone cohort, the 3 patients with VF had SDI scores of 1, 3 and 8. In SoC-GC cohort, among the 13 patients with VF, 7 had SDI of 1, while the remaining patients had SDIs of 3, 4 (n=3), 5 and 6. Mean bone age delay was 0.8±1.5 years on vamorolone vs 1.6±1.6 years on SoC-GC.

**CONCLUSIONS:** The prevalence of VF was significantly lower with vamorolone compared to SoC-GC after ~5 years of exposure. Patients on vamorolone should continue to be monitored for VF.

### Disclosure

EG, SH, ML, and AL are employees or consultants of Santhera Pharmaceuticals. Various other authors have provided paid consultancy to pharmaceutical companies with no specific conflict of interest regarding the submitted abstract.

### P84

### BMN 333 Clinical Development Program in Achondroplasia: Phase 1 Results and Phase 2/3 Study Design Overview

Ravi Savarirayan[1], Julie Hoover-Fong[2], Melita Irving[3], Lynn Han[4], Christine Rivat[5], Yulan Qi[4], Nina Mitchell[5]

[1]Murdoch Children's Research Institute, Royal Children's Hospital, and University of Melbourne, Parkville, Victoria, Australia. [2]McKusick-Nathans Department of Genetic Medicine, Johns Hopkins University, Baltimore, MD, USA. [3]Guy's and St Thomas' NHS Foundation Trust, London, UK. [4]BioMarin Pharmaceutical Inc., Novato, CA, USA. [5]BioMarin (UK) Ltd, London, UK

### Abstract

**OBJECTIVES**: Achondroplasia is a skeletal dysplasia caused by excess FGFR3 signaling. Vosoritide, a recombinant CNP analog, is approved to treat achondroplasia by counteracting excess FGFR3 signaling to stimulate endochondral bone growth. BMN 333 is an investigational, fully biodegradable treatment designed to extend exposure to vosoritide, its active moiety. Preclinically, BMN 333 demonstrated sustained vosoritide release, minimized hemodynamic effects, and enabled higher CNP exposure to promote skeletal growth. In healthy adults, BMN 333 was well-tolerated with no dose-limiting toxicities identified; sustained exposure to released vosoritide and associated pharmacodynamic target engagement responses were observed. We present here the BMN 333 clinical development program.

**METHODS**: Phase 1 evaluated BMN 333 safety and pharmacokinetics in healthy adults (Figure). ASPEN is an active-controlled, operationally seamless phase 2/3 study of BMN 333 in treatment-naïve children with achondroplasia and the first head-to-head superiority trial against vosoritide (Figure). The phase 2 part will evaluate once-weekly doses of BMN 333 (150, 250, 350 µg/kg) vs once-daily vosoritide (1:1:1:1-randomized) for 52 weeks in children aged ≥2 to <11 years. An interim analysis of phase 2 preliminary safety, pharmacokinetics, and efficacy, which may be augmented with historical vosoritide trial data, will inform phase 3 dose selection and continuation. The phase 3 part will examine superiority of BMN 333 vs vosoritide for improving growth in children aged ≥2 to <18 years receiving once-weekly BMN 333 or once-daily vosoritide (1:1-randomized) for 52 weeks.

**RESULTS**: In phase 1, BMN 333 demonstrated linear pharmacokinetics, with dose-proportional increases in systemic exposure. Phase 2 dosing was therefore selected as 150 µg/kg to match and 250/350 µg/kg to exceed vosoritide efficacy. The phase 3 primary endpoint is week-52 annualized growth velocity; key secondary endpoints are week-52 change from baseline in standing height, height Z-score, and upper-to-lower body ratio. Novel, data-rich digital health technology-based endpoints will objectively evaluate functional benefits. ASPEN was submitted for regulatory approval in late 2025, and phase 2 recruitment is planned for early 2026.

**CONCLUSIONS**: BMN 333 is designed to advance the therapeutic approach established with vosoritide. ASPEN will evaluate BMN 333 safety, efficacy, and superiority vs vosoritide for improving growth in children with achondroplasia.

**Figure.** Overview of BMN 333 clinical development program

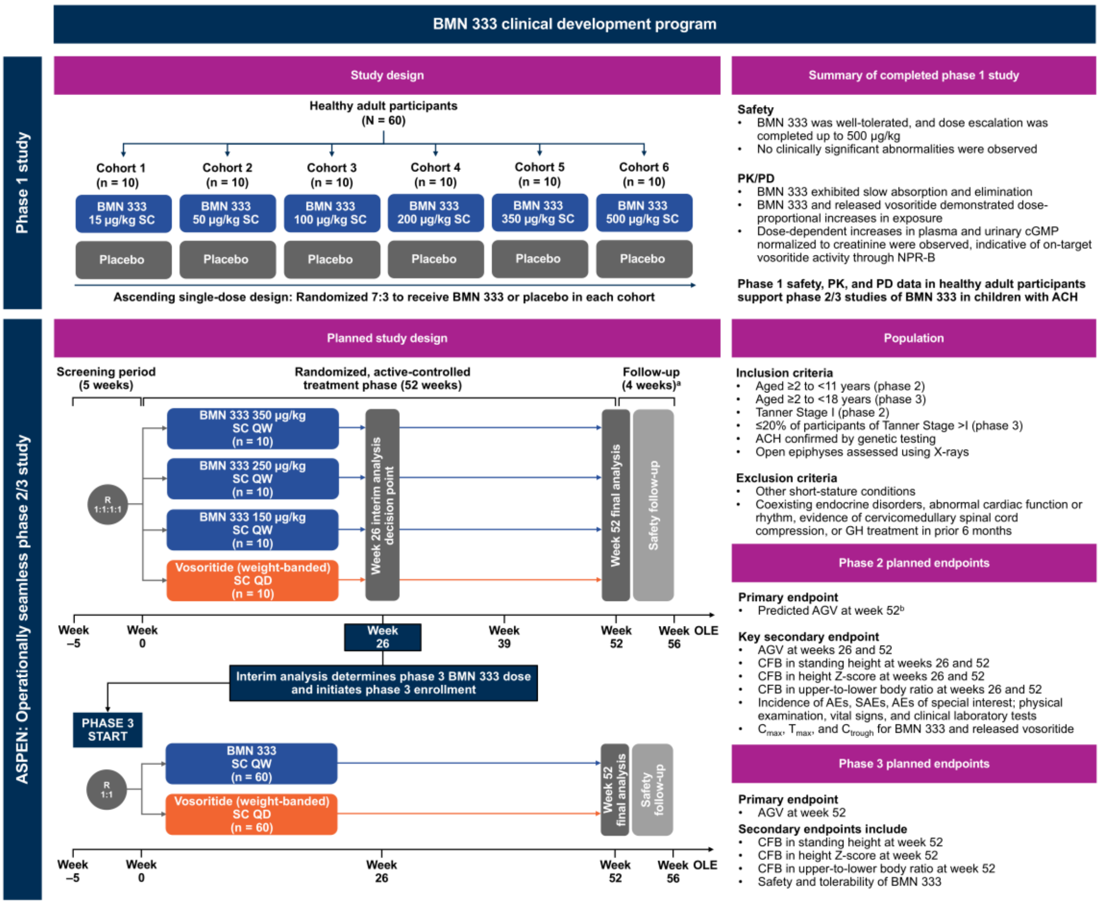

[a]The safety follow-up visit at week 56 will be waived for participants who enroll into the OLE within 2 weeks of receiving the last dose of the study drug.
[b]AGV at week 52 is predicted based on AGV at weeks 26, 39, and 52 (available cumulative data).

ACH, achondroplasia; AE, adverse event; AGV, annualized growth velocity; CFB, change from baseline; cGMP, cyclic guanosine monophosphate; $C_{max}$, maximum plasma concentration; $C_{trough}$, trough plasma concentration; GH, growth hormone; OLE, open-label extension; NPR-B, natriuretic peptide receptor B; PD, pharmacodynamic; PK, pharmacokinetic; QD, once daily; QW, once weekly; R, randomized; SAE, serious AE; SC, subcutaneous; $T_{max}$, time to maximum plasma concentration.

### Disclosure

R.S. has received consulting fees and grants from BioMarin Pharmaceutical Inc., participated on advisory boards for Ascendis and BioMarin Pharmaceutical Inc., and consulted for BridgeBio. J.H.-F. has received grants or contracts for clinical trials from BioMarin Pharmaceutical Inc., Pfizer, and QED; received consulting fees from BioMarin Pharmaceutical Inc., Innoskel, Novo Nordisk, and QED; received honoraria from BioMarin Pharmaceutical Inc. and Medscape; and participated in advisory boards for BioMarin Pharmaceutical Inc., MCDS-Therapy Clinical Trial, and QED. M.I. has received payments and honoraria from the European Achondroplasia Forum and support for attending meetings and travel from the European Achondroplasia Forum. L.H., C.R., Y.Q., and N.M. are employees and stockholders of BioMarin Pharmaceutical Inc.

### P85

### A Comparison of Longitudinal BMD Change with and without Bisphosphonate Treatment in Children with Osteogenesis Imperfecta

Heidi Kecskemethy, Julia Gray, Jeanne Franzone, Cristina McGreal, Shannon Bonner, Mahim Jain

Nemours Children's Health, Wilmington, USA

### Abstract

OBJECTIVES:

Evaluate serial BMD results of the lumbar spine (LS) and LDF in children with OI who are both naïve to bisphosphonate (BP) and who have been treated to evaluate the performance of the LDF DXA to LS and compare the rates of change over time.

METHODS:

Retrospective chart review of children with OI treated at our tertiary pediatric hospital from 2002 through 2024. Included subjects had valid LS and LDF scans with at least one year of evaluation between scans and with no metal hardware at either site. Annual percent change was calculated for each participant by DXA site (R1 – R3). Data were grouped by untreated and treated with bisphosphonate. Data were normally distributed and analyzed via student's t-test with Welch's correction; one-way ANOVA was used to evaluate differences between treated and untreated groups for each site.

RESULTS:

Sixteen children (8 girls) with mean age of 7.3 years at first DXA were evaluated. Ten received treatment. Individual observation ranged from 2.8 to 19.9 years for a total of 152.55 years or 9.53 person-years. Age of BP initiation was 8.4 (2 – 16.2) years. Average annualized rate of change differed between the treated and untreated groups: untreated gained 8.68% vs treated gained 15.2%. There were significant differences in percent change at all DXA sites between the groups (LS p=0.0166; LDF R1 p=0.0172; LDF R2 p=0.0054; LDF R3 p=0.0139). LS and LDF R1showed the greatest BMD gains over time followed by the LDF R1 – R3, respectively. There were no significant differences between DXA sites within groups.

CONCLUSIONS:

All DXA sites showed gains in BMD over time; the BP treated group had the greatest rate of change, as expected. LDF BMD reflected similar patterns in change as the LS. This is the first longitudinal report of lower extremity BMD measured at the distal femur in children with OI. The LDF DXA offers an additional body site to use for serial measure of BMD in children with OI.

### Disclosure

None

### P86

### Safety Profile of Zoledronic Acid Infusions in Pediatric Patients with Osteogenesis Imperfecta

Juan Llerena Jr[1,2,3], Ruth Paulino[1,3], Heloisa dos Santos[1,3], Ana Laura Medeiros[1,3], Sandra Pimentel[1,3], Thais Fernandes[1,3], Roberta Tuche[1,3], Tatiana Magalhães[1,3]

[1]Centro de Genética Médica & Serviço de Referência para Doenças Raras – Instituto Nacional Fernandes Figueira (Fiocruz), Rio de Janeiro, Brazil. [2]Faculdade de Medicina de Petrópolis (UNIFASE), Petrópolis, RJ, Brazil. [3]Centro de Referência para Osteogênese Imperfeita - IFF/Fiocruz, Rio de Janeiro, Brazil

### Abstract

### BACKGROUND:

Zoledronic acid (ZD) has emerged as an effective alternative for managing Osteogenesis Imperfecta (OI), a rare genetic disorder characterised by bone fragility and recurrent fractures. For the past two decades, our Reference Centre has primarily adopted the Pamidronate (PD) protocol as the first-line treatment for OI patients. In April 2024, a transition to the ZD protocol was implemented in 119 patients following the guidelines established by Evelina Children's Hospital (Dr Moira Cheung, London). All infusions were administered in a controlled setting at the Day-Hospital unit to ensure patient safety and close monitoring during the initial phases of treatment.

**PRESENTING PROBLEM:**

The primary objective of this study was to evaluate the safety profile of ZD infusions in pediatric and adolescent patients with OI, particularly focusing on the incidence and severity of adverse events during and after administration.

### CLINICAL MANAGEMENT:

The study population was divided into three distinct groups: (1) treatment-naïve patients, (2) patients transitioning from PD to ZD, and (3) patients previously treated with oral bisphosphonates (alendronate) switching to ZD.

Dosage regimens were tailored according to age categories:

- 0–2 years: 0.025 mg/kg every three months
- 2–5 years: 0.035 mg/kg every four months
- >5 years: 0.05 mg/kg every six months

For treatment-naïve patients, the initial infusion consisted of half the calculated dose, with most cases managed under inpatient observation. Across all groups, adverse events were mild and transient, including fever, gastrointestinal discomfort, muscle cramps, and bone pain. Supportive care was sufficient for symptom resolution, and no severe complications were reported. Importantly, subsequent infusions were well tolerated, with no recurrence of adverse reactions.

### DISCUSSION:

Previous literature has consistently documented mild adverse events associated with bisphosphonate therapy. Our findings corroborate these reports, demonstrating that ZD infusions are generally safe when administered under appropriate protocols. The absence of significant complications and the favourable tolerability observed in follow-up treatments reinforce ZD as a reliable and effective option for OI management. Consequently, ZD has been adopted as the first-choice therapy in our unit, replacing PD as the standard of care for this patient population.

**Table: Adverse Events Following Zoledronic Acid Infusions in OI Patients**

| **Patient Group (n)** | **No Adverse Event (n)** | **Adverse Event** | **Non-informed (n)** | **Type of Events*** |
|---|---|---|---|---|
| Virgin of treatment (54) | 31 | 13 (29.5%) | 10 | Fever, cramps, bone pain |
| PD → ZD (33) | 25 | 6 (19.4%) | 2 | Diarrhoea, irritability, drowsiness, muscle cramps, bone pain |
| Oral drug → ZD (32) | 22 | 6 (21.4%) (*) | 4 | Fever, headache, bone pain |

*Most events occurred during the first infusion only, except for two patients who lasted for two to three days (*).

**Disclosure**

None declared

### P87

### Bisphosphonate Monitoring and Treatment Practices in Pediatric Neuromuscular Conditions: A Survey of US Bone Health Clinicians

Heidi Kecskemethy, Julia Gray, Laura Owens

Nemours Children's Health, Wilmington, USA

### Abstract

OBJECTIVES: Despite decades of bisphosphonate (BP) use for bone fragility in children with neuromuscular conditions, bone health screening and treatment practices vary. We aim to describe current monitoring and treatment practices around BP use among bone health clinicians treating children with neuromuscular conditions, worldwide.

METHODS: An IRB-approved online survey was distributed via professional society listservs and meetings to collect detailed information about practices around BP use, screening practices, definitions of low BMD, treatment indications, BP selection, dosing, and drug holiday practices. This is a preliminary assessment of US-based respondents. Descriptive statistics were calculated for all variables.

RESULTS: Twenty-three US bone health clinicians (21 MD/DO, 2 APN/PA) from diverse specialties: pediatric endocrinology (n=7), general pediatrics (n=8), rehabilitation medicine (n=5), orthopedics (n=2), nephrology (n=2), and neuromuscular pediatrics (n=1) completed the survey. Respondents had an average of 12.8 years (range 1.5-25) in bone health practice, with 17 reporting formal bone health training. Most clinicians (n=16) treated children and young adults (0-21 years).

Conditions treated included cerebral palsy (n=17), Duchenne muscular dystrophy (n=16), spinal muscular atrophy (n=15), glucocorticoid use (n=14), and osteogenesis imperfecta (n=12). Reported DXA sites include total body (n=22), lumbar spine (n=20), forearm (n=11), lateral distal femur (n=9), proximal femur/hip (n=8). Criteria for low BMD was described as Z-score ≤-2.0 by 21/23 respondents. Seventeen clinicians (74%) reported actively treating patients with BP, managing an average of 45.7 patients annually. Zoledronic acid was the most frequently used agent, followed by pamidronate, and alendronate. Treatment indications included low-energy fractures (n=13), symptomatic vertebral fractures (n=11), asymptomatic vertebral fractures (n=13), and low BMD (n=7). DXA monitoring frequency varied from every 7-12mo up to every 3-5 years if no BP or up to every 19-24mo if using BP. Drug holiday practices varied with 7 providing routine holidays, 7 some providing none, 4 using condition-dependent approaches.

Additional information includes practices on screening and laboratory monitoring, dosing, and concurrent supplementation practices and BP holidays related to orthopedic surgery.

CONCLUSIONS: Practices vary widely by US providers using BP in children with neuromuscular conditions. These findings highlight the need for evidence regarding optimal BP use in pediatrics. This survey is ongoing to collect global practices.

### Disclosure

None

### P88

### Bone Mineral Density Following Bisphosphonate Administration in Pre and Post Pubertal Children with Neuromuscular Conditions

Laura Owens[1], Julia Gray[2], Heidi Kecskemethy[2]

[1]Nemours Children's health, Wilmington, USA. [2]Nemours Children's Health, Wilmington, USA

### Abstract

OBJECTIVES:

We examine serial change in areal bone mineral density (aBMD) during and after bisphosphonate (BP) treatment in non-ambulatory pre- and postpubescent children with neuromuscular conditions to assess response to treatment relative to pubertal status.[CA1]

METHODS:

IRB-approved cross-sectional retrospective review of aBMD results for children 2 – 18 years, who received at least 1 year of BP at our children's hospital. Data grouped by Tanner stage during BP treatment: Group 1 Prepubescent (1-2), Group 2 Postpubescent (3-5). aBMD values & Z-scores for lumbar spine (LS) & Lateral Distal Femur (LDF) R1-R3 were evaluated. Percent change in BMD was evaluated, then annualized to account for differing observation lengths. Non-paired t-tests were performed with Welch's correction for group comparisons.

RESULTS:

Twenty-two children (11 f, mean age 11.6 y at BP initiation) were included; 12 were prepubescent. Total observation: 107 years (4.9 person years). Average treatment duration was 2.3 years & observed post-treatment duration was 4.5 years.

During treatment, aBMD and Z-scores increased at all sites in both groups. LDF R1 aBMD rose significantly more in prepubescents than postpubescents (36% vs 17% annual change, p=0.0275). No significant aBMD differences were seen but Z-score patterns differed between the groups at R2, R3 and LS.

After treatment, LDF R1 aBMD in prepubescents decreased 7.4%, while postpubescents increased 3.8% (p=0.0009). R1 Z-scores decreased in both groups after stopping BP. No significant differences in aBMD change occurred between the groups at R2, R3 and LS, but serial a BMD and Z-score patterns differed. Post treatment, Z-scores worsened in all prepubescents but stayed the same or decreased slightly postpubescently.

CONCLUSIONS:

aBMD & Z-scores increased at all DXA sites during BP treatment as expected, but in differing ways in pre- and postpubescents. Response to BP at LDF R1 was notably different: prepubescents showed a marked increase in aBMD with BP treatment, followed by a commensurate decrease after discontinuation. Postpubescents showed more modest response to BP initiation and discontinuation at R1. Significant differences were not seen at other sites, perhaps due to small sample size. Pattern of Z-score change across sites suggests differing response to BP by pertal status; this bears further research.

### Disclosure

None

### P89

### Burosumab Treatment in Infants Under One Year of Age with X-Linked Hypophosphatemic Rickets (XLH): Safety, Efficacy, and Real-World Evidence

Kelly Maury[1], Veronica Figueroa Gacitua[2], Macarena Llanos[3], Oscar Brunetto[3]

[1]Hospital de Niños Pedro de Elizalde, Ciudad Autonoma Buenos Aires, Argentina. [2]Hospital de Niños Pedro de Elizalde, CiudadAutonoma de Buenos Aires, Argentina. [3]Hospital de Niños Pedro de Elizalde, Ciudad Autonoma de Buenos Aires, Argentina

### Abstract

**Background:**

X-linked hypophosphatemic rickets (XLH) typically manifests when a child begins weight-bearing. While conventional therapy with phosphate salts and calcitriol has been the historical standard, it carries significant risks of nephrocalcinosis and hyperparathyroidism. Burosumab, an anti-FGF23 biological therapy, has shifted this paradigm. However, long-term follow-up data in patients starting treatment before twelve months of age in a real-world clinical setting remains limited.

**Presenting Problem:**

We present a real-world case of a male infant, born to a mother with XLH. Genetic testing confirmed a mutation in the *PHEX* gene at birth. Due to normal phosphorus levels during the neonatal period, conventional treatment was deferred in favor of close monitoring to initiate biological therapy directly. His medical history included pulmonary valve stenosis, which was successfully resolved. At 6 months, biochemical alterations appeared: decreased serum phosphorus and elevated alkaline phosphatase (ALP), without clinical deformities. Burosumab (0.8 mg/kg) was initiated at 8 months of age.

**Clinical management:**

After the first year, the patient showed sustained normalization of plasma phosphorus. Motor development was excellent; he achieved independent walking without skeletal deformities and maintained an adequate growth velocity without adverse events. During the second year of treatment, growth remains within normal percentiles and his genetic target range. Biochemically, he maintains normal phosphorus and ALP levels for his age. Regarding safety, there was an absence of nephrocalcinosis in renal ultrasounds and no skin reactions. Tolerance has been optimal despite his cardiological history.

**Discussion:**

This case represents one of the longest follow-up studies of an infant treated with Burosumab from 8 months of age without prior conventional therapy. Two years of complication-free follow-up reinforce the safety of the drug during the window of high bone plasticity. This successful outcome suggests that early biological intervention as first-line therapy can change the natural history of XLH, ensuring normal skeletal development

### Disclosure

None declared

### P90

### Clinical Benefit of Burosumab in Gene-Negative X-Linked Hypophosphataemia: A Case Report

Louise Apperley, Charlotte Jarvis, Suzanne Armitage, Renuka Ramakrishnan

Alder Hey Children's Hospital, Liverpool, UK

### Abstract

### Background

X-linked hypophosphataemia (XLH) is challenging to manage with conventional therapy. Compliance can be an issue due to multiple medications, frequent dosing and poor tolerability. Burosumab, a human monoclonal antibody, acts by suppressing fibroblast growth factor 23 (FGF23), which results in increased phosphate levels and improved pain. It has been approved for XLH treatment in children aged over 1 year.

### Presenting Problem

An 8-year-old female was referred with varus bowing in the distal third of both tibia (left more pronounced than the right), waddle-like gait and internal rotation when moving. She was born at term with no antenatal or postnatal history. Parents were non-consanguineous and there was no family history. They reported bowing of legs from a few months of age, which worsened at 15-months when she started walking. She had no hearing or dental concerns.

### Clinical Management

On review, the patient was receiving treatment for hypophosphataemic rickets (alfacalcidol and oral phosphate), but no improvement was seen over 2 years. She experienced persistent leg pain and struggled to walk fast and run, which resulted in psychological distress. Genetic testing for hypophosphataemic rickets was negative. Lumbar spine BMD Z-score was +1.2 and a BMAD z score was +2.91. Bone biopsy and expert radiological review supported a diagnosis of XLH.

The conventional treatment was optimised for several months, but the patient continued to have abnormal biochemistry (Table 1), significant symptoms and progressive genu varum. The decision was made to discontinue her oral medications for a washout period and initiate a trial of subcutaneous Burosumab. Biochemical parameters improved within weeks (Table 1), and by six months there was marked improvement in symptoms and quality of life.

### Discussion

This case demonstrates the beneficial effects of Burosumab in a paediatric patient with probable gene-negative XLH following prolonged conventional therapy. As approximately 20% of cases lack an identifiable pathogenic *PHEX* variant, Burosumab should be considered in patients who exhibit characteristic clinical, biochemical, and radiological features. Treatment is associated with improvements in lower limb alignment, growth, pain, and overall quality of life.

**Table 1 shows the biochemistry results on conventional versus Burosumab therapy**

| | At referral | On optimal oral medications | Off medications (washout period) | 1-month post-Burosumab | 6-months post-Burosumab |
|---|---|---|---|---|---|

| | | | | | |
|---|---|---|---|---|---|
| Adjusted Calcium (1.15-2.74 mmol/L) | 2.22 | 2.24 | 2.22 | 2.17 | 2.24 |
| Phosphate (0.97-1.94 mmol/L) | 0.87 | 0.64 | 0.65 | 1.71 | 1.29 |
| Alkaline Phosphatase (93-367 iu/L) | 570 | 676 | 761 | 527 | 298 |
| Fibroblast Growth Factor 23 (0-100 RU/ml) | 99 | X | 126 | X | X |
| Vitamin D (>50 nmol/L) | 36 | 38 | 46 | X | 129 |
| Parathyroid Hormone (1.1-6.9 pmol/L) | 10.2 | 19.9 | 20.1 | 17.6 | 7.5 |
| TmP/GFR (1.11-1.70) | X | X | 0.6 | X | X |

### Disclosure

None declared

### P91

### Clinical Improvement of Childhood Hypophosphatasia with Aasfotase Alfa Treatment

Myra Poon[1,2], Christie-Lee Wall[1], Suzie Taylor[1], Andrew Biggin[3], Craig Munns[4]

[1]The Children's Hospital at Westmead, Westmead, Australia. [2]Discipline of Paediatrics and Child Health, The University of Sydney, Sydney, Australia. [3]Children's Health Queensland, South Brisbane, Australia. [4]Child Health Research Centre, Faculty of Health, Medicine and Behavioural Sciences, Brisbane, Australia

### Abstract

BACKGROUND: Hypophosphatasia (HPP) has a broad phenotypic spectrum ranging from severe infantile forms to minimally symptomatic adult phenotypes, caused by ALPL gene variants which result in reduced TNS-alkaline phosphatase (ALP) activity (1). The phenotype can be improved with asfotase alfa enzyme replacement therapy (2,3). We present a case of childhood-onset HPP demonstrating significant clinical improvement.

PRESENTING PROBLEM: The subject had a history of hypotonia, genu varum, ankle and foot pain, delayed gross motor milestone acquisition and premature exfoliation of deciduous dentition (atraumatic loss of four teeth soon after eruption) on presentation at 23 months. He was delivered at term following an uneventful pregnancy, with no perinatal issues and no history of seizures. Examination revealed short stature (height –2.44SD), significant hypermobility with genu recurvatum, patent anterior fontanelle.  Biochemistry demonstrated low ALP 39mIU/L (NR 120-450), normal calcium, magnesium, phosphorus, parathyroid hormone and 25-OH vitamin D, elevated pyridoxal-5-phosphate 553nmol/L (NR 35-110) and urinary phosphoethanolamine (PEA, PEA:Cr 59umol/mmol NR 0-19). Plain radiographs demonstrated reduced cortical thickness, expanded metaphyses and metaphyseal lucencies of all long bones.  Genetic testing confirmed two pathogenic heterozygous missense ALPL variants: c.526G>A, p.(Ala176Thr) and c.1471G>A, p.(Gly491Arg).

CLINICAL MANAGEMENT: Asfotase alfa 2mg/kg was administered 3 times/week via subcutaneous injection from age 28 months (3). Biochemical monitoring demonstrated marked ALP elevation 8476IU/L (120-370) as expected, intermittent phosphorus elevation (max 2.66mmol/L, NR 1.1-2.2) with otherwise normal bone homeostasis.

The patient progressed from bottom shuffling to crawling after 3 months treatment, to walking after 9 months. Lower limb pain resolved and genu recurvatum improved from +45 degrees to +10-15 degrees. These changes were accompanied by radiographic improvement in skeletal mineralisation. He developed minor injection site hypersensitivity reactions and required surgically guided growth to correct genu valgum between 4-5 years of age. After 60 months treatment, height z-score increased by 0.65SD and he participates in age-appropriate activities with no functional impairment.

Serial renal ultrasounds and opthalmology assessment have excluded nephrocalcinosis and corneal calcifications.

DISCUSSION: Asfotase alfa enzyme treatment can significantly improve gross motor function, musculoskeletal pain and skeletal mineralisation in childhood HPP allowing age-appropriate physical participation. At present, treatment is limited by cost and availability depends on financial support or geographical location.

### Disclosure

None declared

### P92

### Treatment with Burosumab in Two Adolescents with X-Linked Hypophosphatemia who Previously Received Well-Controlled Conventional Therapy: Clinical Cases

Cristina Tau

Pediatric Department, Clinical Hospital, Buenos Aires University, Buenos Aires, Argentina

### Abstract

**Background**:

X-linked hypophosphatemia (XLH) is a rare heritable skel-etal disorder characterized by excess production and circu-lating concentrations of fibroblast growth factor 23 (FGF23), resulting in renal phosphate wasting, hypophosphatemia, and rickets. Two teenage girls, aged 15 and 14 years 8 months affected by XLH, are presented.

**Presenting problem and clinical management:**

**Patient 1** began when she was 1 year old with instability and falls when standing up. At 1 year and 7 months old, severe rickets was diagnosed in the metaphyses of the tibias, femurs, and wrists (SRR *6),* ankles, costal rosary, genu varum and short stature (z-score -2.08), hypophosphatemia (2.8 mg/dl (normal value 3.9-5.7), and hyperphosphaturia (TmP/GFR 2.4 mg/dl (normal value 2.9-6). She began conventional therapy (phosphate salts and calcitriol), which received for 10 years. Exhausted from taking phosphate salts 5 times/day, at 12 years 11 months she was switched to Burosumab s.c. 0.8 mg/kg every 2 weeks. Height z-score was -0.67, SRR 0. Burosumab therapy immediately corrected and kept for 2 years the phosphatemia (mean±SD 3.67±0.39 mg/dl) and stopped the loss of phosphate through the urine (TmP/GFR 3.44±0.49 mg/dl).

**Patient 2** began at 1 year old when her parents noted deformity of her limbs. At 1 year 8 months, severe rickets was diagnosed (SRR *3*), genu varum and short stature (z-score -2.24), hypophosphatemia (2.8 mgdl), hyperphosphaturia (TmP/GFR 1.57 mg/dl). She began conventional therapy when she was 2 years old, which she received for 11 years. Growth improved to a height score of -0.33. She had mild nephrocalcinosis. When she was 13 years and 8 months old, she switched to burosumab SC 0.8 mg/kg every 2 weeks. At the start of burosumab, her z-score of height was -0.86. Burosumab immediately corrected and kept for a year the phosphatemia (4.1±0.33 mg/dl) and stopped the loss of phosphate through the urine (TmP/GFR 3.98±0.66 mg/dl). Burosumab therapy also corrected functional tests showing more energy without pain or fatigue. No adverse effects were observed except for some redness at the injection site.

**Discussion:** Burosumab therapy corrected biochemical parameters, pain, physical function, and mobility compared with conventional therapy. These results reinforce the favorable effect of burosumab on adolescents with XLH.

### Disclosure

No conflicts of interests

### P93

### Correlating Vitamin D Level with Changes in Volumetric Bone Qualities from Adolescence to Early Adulthood - 6-Year Prospective Longitudinal Cohort Study of 110 Girls with Adolescent Idiopathic Scoliosis

Kenneth Guangpu Yang, Eric Cheuk-Kin Kwan, Wayne Yuk-Wai Lee, Alec Lik-Hang Hung, Vivian Wing-Ying Hung, Tsz-Ping Lam, Jack Chun-Yiu Cheng, Adam Yiu-Chung Lau

The Chinese University of Hong Kong, Hong Kong, China

### Abstract

### Objectives:

Patients of Adolescent Idiopathic Scoliosis (AIS) were reported to have systemic low bone density and impaired bone qualities. The impact of Vitamin D (Vit-D) deficiency on bone mineral accrual during adolescence and peak bone mass (PBM) remains uncertain. This prospective cohort study investigated the association between serum 25-hydroxyvitamin D (25OHD) and bone density and bone qualities from early adolescence to PBM in AIS.

### Methods:

A total of 110 Chinese girls with AIS (aged 11–14 years) were recruited at baseline (Visit-B) and followed at one year (Visit-1), two years (Visit-2), and six years (Visit-6, early adulthood). Bone density and quality were assessed using high-resolution peripheral quantitative computed tomography (HR-pQCT). Serum 25OHD was measured in the same season at each visit using liquid chromatography–tandem mass spectrometry (LC-MS/MS).

### Results:

Patients were followed from age 12.9±0.8 to 19.2±0.9 years. During early adolescence (Visit-B to Visit-2), higher serum 25OHD was significantly associated with greater increases in total volumetric BMD (0.24 mgHA/$cm^3$ per nmol/L, $p = 0.046$), estimated bone stiffness (57.4 N/mm per nmol/L, $p = 0.020$), and failure load (2.07 N per nmol/L, $p = 0.042$), as well as greater reduction in trabecular area (-0.159 $mm^2$ per nmol/L, $p = 0.024$). Of the 110 patients, 70 (64%) completed all four visits. At Visit-6, patients with severe Vit-D deficiency (25OHD < 30 nmol/L) in ≥2 visits had significantly lower total volumetric BMD (353.55±65.67 vs 381.05±60.02 mg/$cm^3$, $p = 0.034$), and higher cortical perimeter (57.54±5.20 vs 55.45±4.69 mm, $p < 0.001$) and trabecular area (140.76±32.03 vs 126.94±30.10 mm, $p < 0.001$) compared with those with ≤1 measurement of severe Vit-D deficiency.

### Conclusions:

Vitamin D deficiency is associated with lower bone mineral accrual and impaired bone qualities during peripubertal period among patients with AIS. Persistent severe Vit-D deficiency (≥2 visits) may result in suboptimal PBM in early adulthood. Ensuring adequate Vitamin D status, including supplementation, should be considered in this population.

### Disclosure

None declared

### P94

### Longitudinal Biochemical Control in Childhood Hypoparathyroidism and Pseudohypoparathyroidism Using Alfacalcidol Titration with Minimal Long-Term Oral Calcium: A 22-Year Observational Cohort

Ashmit Rao[1], Aashima Dabas[2], Lauren Rayner[3], Amit F Maniyar[3], M Zulf Mughal[4], Amish Chinoy[3], Raja Padidela[3,5]

[1]University of Oxford, Oxford, UK. [2]Maulana Azad Medical College, New Delhi, India. [3]Royal Manchester children's Hospital, Manchester, UK. [4]Al Jalila Children's Hospital, Dubai, UAE. [5]University of Manchester, Manchester, UK

### Abstract

### Background:

Conventional management of hypoparathyroidism and pseudohypoparathyroidism (PHP/iPPSD) combines active vitamin D (aVD) with oral calcium supplementation (OCS), but adherence and tolerability of OCS can be challenging. We describe longitudinal biochemical control in a single-centre cohort managed with an approach prioritising dietary calcium, minimising long-term OCS, and titrating alfacalcidol to target ranges.

**Methods:**

Retrospective observational study of 120 children/adolescents treated between 2003–2025 (hypoparathyroidism-n=80; PHP/iPPSD-n=40). All underwent genetic testing; renal safety was monitored by eGFR, urine Ca/Cr and annual renal ultrasonography for nephrocalcinosis. All were started on aVD (predominantly alfacalcidol; 25–50 ng/kg/day initially) plus vitamin-D supplementation; aVD was titrated (typically 4–6 monthly) to maintain albumin-adjusted calcium (adjCa) 1.9–2.3 mmol/L in hypoparathyroidism and 2.0–2.7 mmol/L in PHP/iPPSD, while avoiding PTH oversuppression in PHP/iPPSD. Dietary calcium intake was encouraged, and OCS was tapered once stable/asymptomatic. Time-weighted averages (TWA) of biochemical parameters and proportions of observed time in target ranges were used to summarise longitudinal biochemistry.

### Results:

In hypoparathyroidism, follow-up was 8.2 years (IQR4.0–16.0). Median time in the adjCa target range (1.90–2.30) was 63.3% (44.2–86.8), with 9.7% (1.6–26.3) below and 9.2% (1.0–29.0) above target; TWA adjCa was 2.12 mmol/L (2.03–2.21). Among those ≥12 years, TWA Ca–phosphate product was 3.52 $mmol^2/L^2$ (3.11–3.98; adult target <4.4). OCS was typically short-term (median-1month[0–8]); 12/80 required ongoing OCS for low dietary intake; median aVD dose was 31.8 ng/kg/day (19.6–54.1). In PHP/iPPSD, follow-up was 9.1 years (4.6–14.0) with TWA adjCa 2.21 mmol/L (2.16–2.26). The estimated proportion of time within the acceptable range (2.00–2.70) was 92% (81–99); Ca–phosphate product (≥12 years) was 3.60 (3.08–3.88). Only 5 patients remained on OCS at follow-up. Nephrocalcinosis occurred in 23/80 (28.8%) hypoparathyroidism patients, statistically significant in severe genotypes and higher urine Ca/Cr and aVD dose/kg; unrelated to OCS usage.

**Conclusions:**

In this monitored, single-centre observational cohort, alfacalcidol titration with emphasis on dietary calcium and limited long-term OCS was associated with sustained biochemical control in both disorders. Observational design precludes causal inference; findings are hypothesis-generating and warrant comparative studies to test effectiveness and safety.

Table:

| Cohort | n | Age at follow-up, y | Follow-up duration, y | Ca at follow-up, mmol/L | TWA Ca, mmol/L | TWA Ca % time in range | TWA Ca–$PO_4$ (>12y), $mmol^2/L^2$ | aVD dose ng/kg/day |
|---|---|---|---|---|---|---|---|---|
| Hypoparathyroidism | 80 | 16.9 (11.0–19.7) | 8.2 (4.0–16.0) | 2.17 (2.07–2.28) | 2.12 (2.03–2.21) | 63.3 % (44.2–86.8) | 3.52 (3.11–3.98) | 31.8 (19.6–54.1) |
| PHP/iPPSD (all) | 40 | 17.7 (13.0–25.6) | 9.08 (4.55–14.0) | 2.31 (2.23–2.37) | 2.21 (2.16–2.26) | 92% (81–99) | 3.60 (3.08–3.88) | 28.2 (20.4–41.7) |
| PHP Type 1a | 15 | 17.8 (12.8–19.8) | 10.2 (5.57–16.1) | 2.28 (2.23–2.43) | 2.23 (2.19–2.27) | 94% (85–100) | 3.65 (3.15–4.18) | 23.3 (19.6–37.8) |
| PHP Type 1b | 25 | 17.4 (14.1–26.9) | 8.18 (3.41–13.0) | 2.32 (2.24–2.36) | 2.19 (2.16–2.26) | 92% (78–96) | 3.59 (2.97–3.83) | 28.6 (23.3–41.6) |

TWA=Time weighted average; Ca=Albumin-adjusted calcium; Ca- $PO_4$= Adj Calcium-Phosphate product; aVD=Active Vitamin D

### Disclosure

Raja Padidela has received honorarium and research support from Bridgebio.

### P95

### X-Linked Hypophosphatemic Rickets Due to a Novel Inversion Involving *PHEX*

Prerana Chatty, Victoria Sanders, Andrew Calabria, Michael Levine, Edna Mancilla, Cheryl McClean, David Weber

Children's Hospital of Philadelphia, Philadelphia, USA

### Abstract

**Background:**

X-linked hypophosphatemic rickets (XLH) is a rare, inherited disorder of phosphate-wasting caused by pathogenic variants in the *PHEX* gene leading to increased FGF23-mediated renal phosphate-wasting and impaired bone mineralization. Burosumab, an anti-FGF23, is now considered first-line treatment based upon superior outcomes compared to conventional therapy. Recent clinical practice guidelines emphasize genetic testing to confirm the molecular diagnosis of XLH, as the clinical phenotype can be indistinguishable from other forms of FGF23-mediated hypophosphatemic rickets.

**Presenting problem:**

We report the case of delayed diagnosis of XLH in a medically complex 12-year-old male with an inversion involving *PHEX* that was not identified on a GeneDx multi-gene exon-based sequencing panel for hypophosphatemic disorders. He initially presented at age 6 years with imaging findings of rickets including diffuse osteopenia, fraying and cupping of the distal radius and ulna and periosteal reactions in the bilateral tibias. PMH was notable for prematurity, anoxic brain injury and global developmental delay. Initial biochemical evaluation demonstrated hypophosphatemia, elevated alkaline phosphatase, elevated intact-FGF23 with normal calcium and 25-hydroxyvitamin D.

**Clinical Management:**

In the absence of a genetic diagnosis, treatment was initiated with oral phosphate and then zoledronic acid i.v for suspected osteoporosis. At re-presentation at age 12 years, he had persistent hypophosphatemia and signs of rickets and osteomalacia including metaphyseal widening of long bones and diffusely undermineralized bones. Intact-FGF23 was inappropriately elevated for the degree of hypophosphatemia. In consultation with a genetic counselor, the *PHEX* gene was sequenced on a Variantyx genome platform which identified a novel hemizygous, paracentric inversion of 7.68 Mb within the region of Xp22.11 to Xp21.2 disrupting *PHEX* at the 5’-breakpoint (Figure 1). Following the diagnosis of XLH he was initiated on burosumab.

**Discussion:**

This case highlights that negative initial genetic testing does not exclude the diagnosis of XLH when there is a high clinical index of suspicion. The initial panel utilizing next-generation sequencing was unable to detect balanced inversions, a known limitation of that methodology. Sequencing performed on genome platforms allows for detection of additional variant types and should be considered as second line when a diagnosis would directly impact management, as in this case.

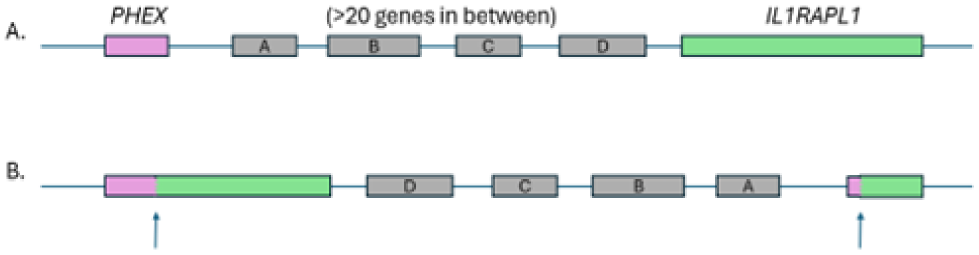

Figure Legend: Pink box: *PHEX* gene; green box: *IL1RAPL1* gene; gray boxes (A-D): placeholder for >20 genes located between *PHEX* gene and *IL1RAPL1* gene. A. Depiction of wildtype region of chromosome region Xp22.11p21.2. B. Depiction of inversion affecting chromosome region Xp22.11p21.2 with arrows indicating breakpoints of inversion.

**Disclosure**

DRW has been a consultant for Ascendis, Catalyst, and Merck. ACC served on the advisory board for Kyowa Kirin. EM is a research investigator for Clementia/IPSEN pharmaceuticals and Incyte.

### P96

### Nephrocalcinosis in Childhood Hypoparathyroidism: Prevalence, Severity and Longitudinal Risk Factors in 80 Patients

Ashmit Rao[1], Aashima Dabas[2], Lauren Rayner[3], Amit F Maniyar[3], M Zulf Mughal[4], Amish Chinoy[3], Raja Padidela[3,5]

[1]University of Oxford, Oxford, UK. [2]Maulana Azad Medical College, New Delhi, India. [3]Royal Manchester Children's Hospital, Manchester, UK. [4]Al Jalila Children’s Hospital, Dubai, UAE. [5]Univrsity of Manchester, Manchester, UK

### Abstract

### Background:

Nephrocalcinosis (NC) is an important complication of treatment in childhood hypoparathyroidism. Paediatric longitudinal data integrating genotype, disease severity, and longitudinal biochemical exposure remain limited. We aimed to characterise the prevalence, severity, natural history, and determinants of NC in a large, genetically characterised cohort managed using a standardised treatment protocol.

### Methods:

A retrospective observational cohort (2003–2025) of 80 children/adolescents with hypoparathyroidism. Patients were managed on active vitamin D (aVD), vitamin D supplementation and dietary calcium optimisation; calcium supplementation was minimised. Renal outcomes were monitored using annual renal ultrasound and spot urine calcium/creatinine (UCa/Cr). Longitudinal albumin-adjusted calcium, calcium–phosphate product and UCa/Cr analysed by time-weighted averages (TWA) as trapezoidal area under the curve divided by time from first to last measurement (linear change assumed); intervals >12 months (7.0%) were censored. Time to first NC was analysed from diagnosis using Kaplan–Meier survival analysis and ridge-penalised Cox models.

### Results:

NC occurred in 23/80 (28.8%): grade 1 (n=11), grade 2 (n=4), grade 3 (n=7), and nephrolithiasis (n=1). Risk varied by aetiology: lowest in 22q11.2 deletion 1/26 (3.8%; RR 0.09) followed by gene-negative 3/16 (18.8%), *GCM2* 9/12 (75.0%; RR 3.64), *CASR* 3/4 (75.0%), and Sanjad–Sakati 3/3 (100%). Kaplan–Meier analyses showed higher NC risk in mechanistically severe genotypes (*GCM2*/*CASR*/Sanjad–Sakati) versus others [log-rank p<0.001 (Figure)] and in children diagnosed <5 years versus ≥5 years (p=0.014). In complete cases (n=61), NC was associated with younger diagnosis age (median 0.16 vs 10.30 years;p<0·001), lower TWA-calcium (2·047 vs 2·14;p=0·026), higher % TWA-Ca below target (15.4 vs 4.53;p=0·017) higher TWA-UCa/Cr (0.74 vs 0.30;p=0.017) and higher aVD dose/kg (52.6 vs 25.9 ng/kg/day;p=0.003). In multivariable ridge Cox models, age of diagnosis (HR 0.59;p=0.032), TWA-UCa/Cr (aHR 2.54, p=0.0002) and aVD dose/kg (aHR 2.15;p=0.0073) remained independently associated. eGFR did not differ by NC status/grade; crude risk of nephrocalcinosis was 35% in males vs 20% in females (risk ratio ≈ 1·75).

### Conclusions:

Nephrocalcinosis affects nearly one-third of children with hypoparathyroidism and is strongly influenced by genotype, early disease onset, and aVD dose. These findings support genotype-informed renal surveillance and UCa/Cr-guided individualisation of therapy to mitigate long-term renal risk.

### Figure

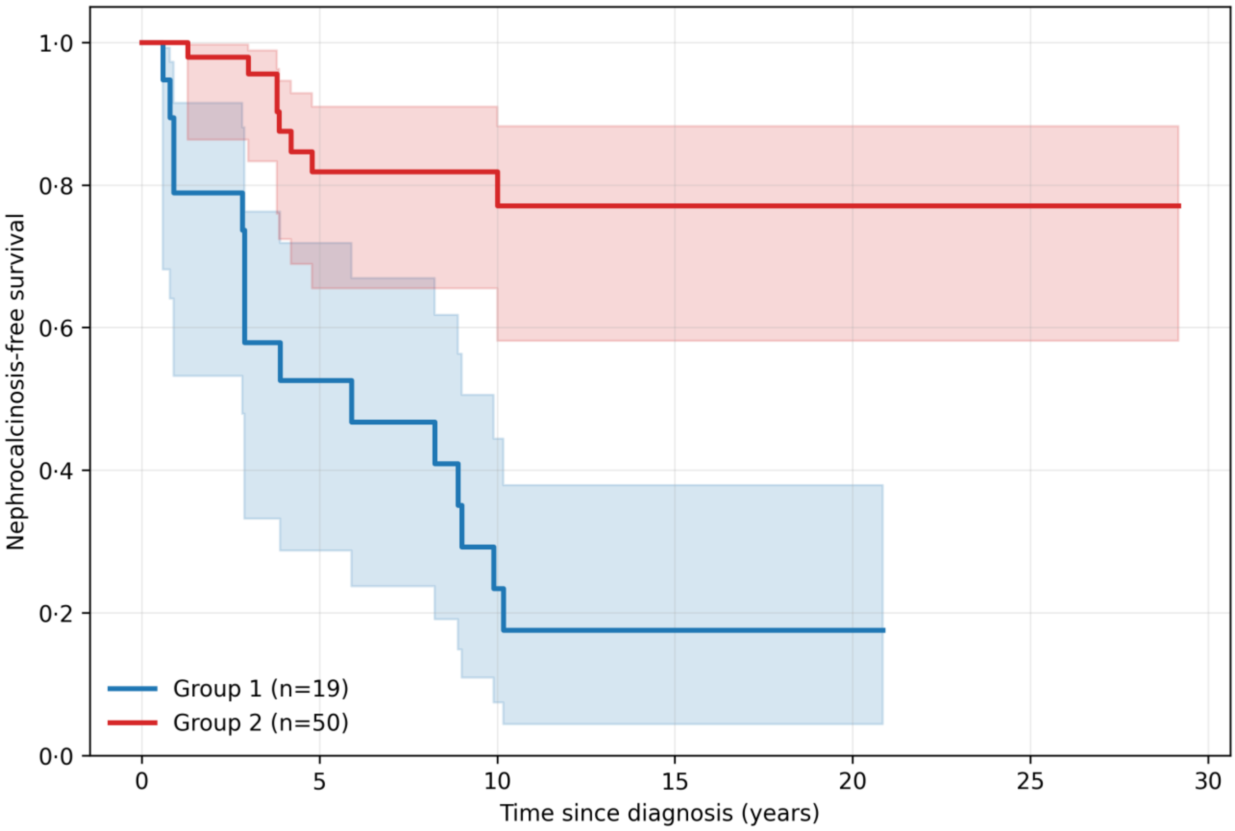

| | 0 | 5 | 10 | 15 | 20 | 25 | 30 |
|---|---|---|---|---|---|---|---|
| Group 1 | 19 (0) | 10 (1) | 4 (0) | 3 (2) | 1 (1) | 0 (0) | 0 (0) |
| Group 2 | 50 (16) | 27 (10) | 17 (8) | 8 (6) | 2 (1) | 1 (1) | 0 (0) |

Time origin is age at hypoparathyroidism diagnosis; event is first nephrocalcinosis; patients without nephrocalcinosis are censored at the last follow-up. Log-rank (Group 1 vs Group 2): $\chi^2(1) = 20{\cdot}599$; $p < 0{\cdot}001$.

**Group 1**: *GCM2/CASR*/Sanjad Sakati

**Group 2**: 22q11.2DS/*AIRE/GATA3*/NegGen/*GNA11*/iatrogenic-MEN3/mitochondrial

**Shaded areas denote 95% CIs**

### Disclosure

Raja Padidela has received an honorarium and a research grant from BridgeBio.

### P97

### When Prevention Turns into Harm: Vitamin D Intoxication Causing Severe Hypercalcemia in an Infant

Marcell Tamas, Rodrigo Montero-Lopez, Elisabeth Laurer, Alexandra Blaschitz, Victoria Schuhmann, Tanja Fritz, Julian Schirl, Thomas Hörtenhuber, Kerstin Wimmer, Wolfgang Högler

Centre for Growth and Osteology, Department of Paediatrics and Adolescent Medicine, JKU Linz, Linz, Austria

### Abstract

BACKGROUND:

Vitamin D supplementation is routinely recommended in infancy. However, dosing errors with highly concentrated preparations can result in severe hypervitaminosis D and life-threatening hypercalcemia. Potential complications include neurological manifestations, nephrocalcinosis and acute kidney injury. Early recognition and targeted management are essential to prevent sequelae.

PRESENTING PROBLEM:

A 4.5-month-old, term-born, formula-fed girl was admitted with poor general condition and suspected infection. On examination, she had truncal hypotonia. Laboratory testing revealed severe hypercalcemia (total calcium 3.43 mmol/L; reference 2.25–2.75; iCa 1.67 mmol/L; reference 1.15–1.35) with elevated 25-hydroxyvitamin D [25OHD] (326 ng/mL; reference 20–80) and suppressed parathyroid hormone [PTH] (<2.4 pg/mL). Renal function showed a mild creatinine elevation (max 0.5 mg/dL; reference 0.16–0.39).

CLINICAL MANAGEMENT:

Clinical history revealed an iatrogenic overdose: a high-dose vitamin D product (10,000 IU per drop) had been administered at 2–3 drops weekly for three months due to a caregiver dosing error.

Vitamin D was stopped and intravenous fluids initiated. Despite this, serum calcium rose to 4.01 mmol/L (iCa 1.87 mmol/L), prompting escalation with hyperhydration and forced diuresis (0.9% NaCl, furosemide), prednisolone and a dietary switch to a calcium-reduced, vitamin-D-free formula. As calcium levels subsequently plateaued despite partial improvement (total calcium 2.55 mmol/L; iCa 1.42 mmol/L), a single dose of pamidronate (1 mg/kg) was administered on hospital day 12, resulting in normalisation. Renal ultrasound demonstrated bilateral medullary nephrocalcinosis, while creatinine normalised during the hospital stay. At discharge, one quarter of intake was calcium-reduced formula, adjusted during follow-up.

At three months, calcium levels were stable with normal 25OHD and PTH. At five months, renal function was normal, nephrocalcinosis remained unchanged and normal feeding was resumed after sustained biochemical stability.

DISCUSSION:

This case highlights that dosing errors with concentrated vitamin D drops can rapidly lead to severe hypercalcemia with neurological and renal complications. Management requires immediate cessation of vitamin D, aggressive hydration, careful use of diuretics with electrolyte monitoring, calcium restriction, and escalation therapy with glucocorticoids or bisphosphonates in refractory cases. This case underscores the need for clear prescribing, appropriate supervision of supplementation, caregiver education and awareness of high-concentration vitamin D preparations to prevent harm.

### Disclosure

None declared.

### P98

### Resolution of Hypoparathyroidism in Children with 22q11.2 Deletion Syndrome: Frequency and Longitudinal Biochemical Predictors in a Tertiary Cohort

Aashima Dabas[1], Ashmit Rao[2], Lauren Rayner[3], M Zulf Mughal[4], Amish Chinoy[3], Raja Padidela[3]

[1]Maulana Azad Medical College, New Delhi, India. [2]University of Oxford, Oxford, UK. [3]Royal Manchester Children's Hospital, Manchester, UK. [4]Al Jalila Children's Hospital, Dubai, UAE

### Abstract

### Background:

Hypoparathyroidism in 22q11.2 deletion syndrome (22q11.2DS) may be transient; the likelihood, timing, and predictors of resolution in childhood remain poorly defined. The aim of this study was to quantify the frequency of resolution of hypoparathyroidism in 22q11.2DS and to identify longitudinal clinical and biochemical predictors of recovery.

### Methods:

Retrospective cohort of children with genetically confirmed 22q11.2DS and hypoparathyroidism managed at a tertiary paediatric centre (2003–2025). Treatment used active vitamin D (aVD) with oral calcium as required, targeting albumin-adjusted calcium (adjCa) of 2.0-2.2mmol/l. Resolution was defined as sustained adjCa >2.2 mmol/l after discontinuation of treatment for ≥1 year with no restart by the last follow-up. Serial biochemistry was summarised using time-weighted averages (TWA). Groups were compared using Mann–Whitney tests with false-discovery rate adjustment. Time to resolution was assessed using Kaplan–Meier methods and ridge-penalised Cox models.

### Results:

Twenty-six children had 22q11.2DS-associated hypoparathyroidism; 11/26 (42.3%) achieved resolution. Twenty-three had sufficient follow-up for time-to-event analyses (events=9; censored=14). Persistent cases were diagnosed later than resolved cases (median age 11.0 [IQR 5.49–13.4] vs 0.20 [0.10–1.30] years; p=0.031; q=0.080), while baseline adjCa and PTH were similar. Resolved cases showed higher longitudinal adjCa (TWA 2.31 [2.23–2.37] vs 2.14 [2.07–2.17] mmol/L; p=0.025; q=0.080) and a higher proportion of adjCa values above target (0.81 [0.145–0.913] vs 0 [0–0.0495]; p=0.002; q=0.010). TWA UCa/Cr did not differ (0.11 [0.054–0.30] vs 0.28 [0.157–0.778]; p=0.864). In ridge Cox models, the proportion of adjCa above target was associated with faster resolution (HR 2.06, 95%CI 1.23–3.45; p=0.006). Kaplan–Meier analyses suggested higher resolution probability when diagnosis occurred before age 5 years [log-rank p=0.05 (Figure)].

### Conclusions:

Around 40% of children with 22q11.2DS experienced resolution of hypoparathyroidism. Recurrent adjCa above target during therapy may signal recovering parathyroid function and should prompt supervised tapering with close biochemical monitoring. Reducing and discontinuing treatment avoids the risk of nephrocalcinosis. These findings support proactive reassessment and carefully supervised tapering of therapy in selected children to avoid unnecessary long-term treatment while maintaining safety.

Figure

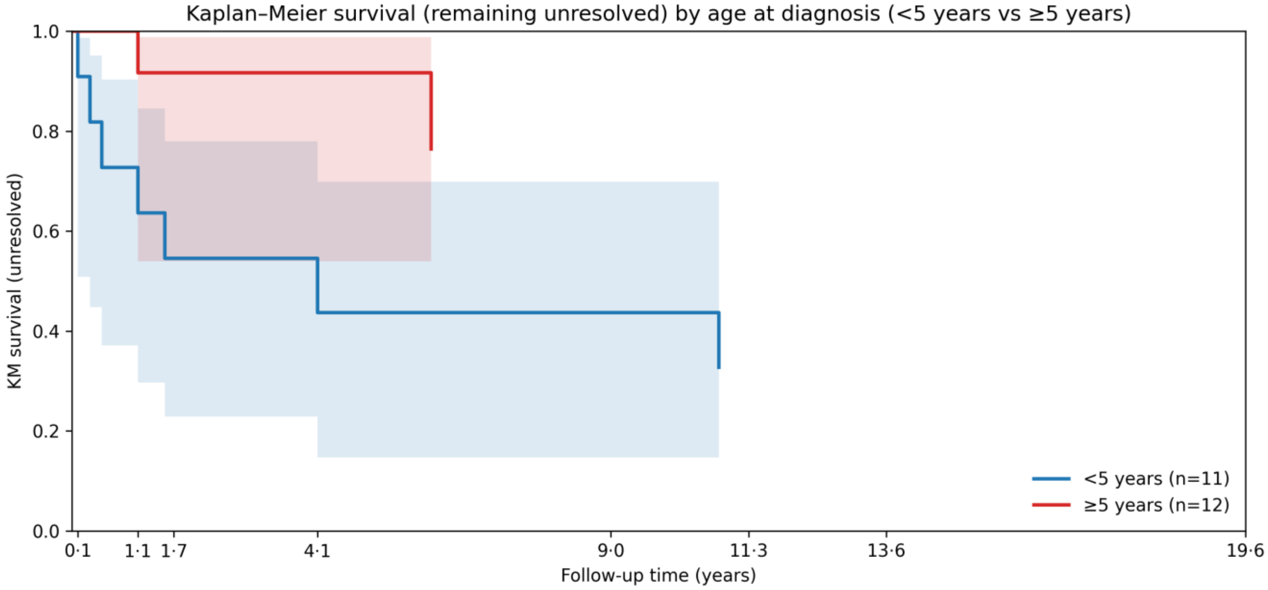

| Group | t=0·1 | t=1·1 | t=1·7 | t=4·1 | t=9·0 | t=11·3 | t=13·6 | t=19·6 |
|---|---|---|---|---|---|---|---|---|
| <5 years | 11 (1) | 7 (3) | 6 (1) | 5 (0) | 4 (1) | 3 (1) | 1 (0) | 0 (0) |
| ≥5 years | 12 (0) | 11 (1) | 9 (0) | 7 (0) | 4 (1) | 2 (0) | 2 (0) | 1 (0) |

Two-sided log-rank test ($\chi^2$=3·85; p=0·05). Total cohort n = 23 (events n = 9; censored n = 14). Shaded areas denote 95% CIs. The event is the resolution of hypoparathyroidism; patients without resolution are censored at the last follow-up. Numbers below the plot indicate participants at risk (events) at each timepoint.

**Disclosure**

None

### P99

### Generalized Arterial Calcification of Infancy (GACI): A Case of Incidental Diagnosis, Multi-System Manifestations and Treatment with Pamidronate

Cherie Chua, Rashida Vasanwala, Poay Joo Kooi

KK Women's and Children's Hospital, Singapore, Singapore

### Abstract

### Background

GACI is a rare disorder characterized by widespread arterial calcifications and intimal proliferation. Biallelic inactivating mutations of ENPP1 gene account for most cases. Infant mortality rate is high and there is limited data on its natural history after infancy. The effect of bisphosphonates in reducing calcifications and mortality is uncertain.

### Presenting Problem

Patient was born at term via normal vaginal delivery at 3.5kg. Parents are non-consanguineous. Antenatal scans showed polyhydramnios. She had respiratory distress at birth that resolved with transient non-invasive ventilation. Chest radiograph incidentally revealed vascular calcifications along the aortic arch and bilateral upper limbs. Further investigations showed diffuse intraabdominal arterial calcifications and reduced cardiac function, suspicious of GACI. Sanger sequencing revealed maternally-inherited pathogenic ENPP1 variant (c.298del p.Ser100AlafsTer35) and paternally-inherited ENPP1 variant of uncertain significance (c.1164+5G>A). Given the clinical phenotype, the *VUS* was reclassified as *likely pathogenic*. Her post-natal course was complicated by cardiac dysfunction, cerebrovascular event and hypophosphatemia.

### Clinical Management

Table 1 shows her clinical progress and management.

**Table 1. Clinical Progress and Management**

| Age | 4-day | 1 month* | 2 month | 3 month | 4 month | 5 month | 6 month | 8 month | 10 month | 12month** | 15month*** | 18 month |
|---|---|---|---|---|---|---|---|---|---|---|---|---|
| Systemic Manifestations# | | | | | | | | | | | | |
| **Arterial calcifications** | | | | | | | | | | | | |
| • Coronary arteries | - | - | | | | | | | | | | |
| • Aorta | + | + | | | | | | | | | | |
| • Renal arteries | + | + | + | | | | + | | | | | |
| • Mesenteric arteries | + | + | | | | | | | | | | |
| • Hepatic arteries | + | + | | | | | | | | | | |
| • Cerebral | - | | | | | | | | | + | | |
| **Extravascular calcifications** | | | | | | | | | | | | |
| • Brain | - | | | | | | | | | + | | |
| • Heart valves | - | | | | | | | | | | | |
| • Kidneys | + | | + | | | | + | | | + | | |
| • Liver | - | | | | | | | | | | | |
| • Periarticular | - | | | | | | | | | | | |
| • Entheses | - | | | | | | | | | | | |
| **Cardiac** | | | | | | | | | | | | |
| • Valve defect | - | - | - | - | - | - | - | | | - | - | |
| • Reduced EF | - | + | - | - | - | - | - | | | - | - | |
| - LVEF (M-mode) | 53% | 32% | 51% | 60% | 56% | 64% | 72% | | | 70% | 53% | |
| - LVFS | 25% | 14% | 21% | 30% | 27% | 33% | 39% | | | 38% | | |
| • Cardiomegaly | - | - | - | - | - | - | - | | | - | - | |
| • Pericardial effusion | - | - | - | - | - | - | - | | | - | - | |
| • Pulmonary hypertension | - | - | - | - | - | - | - | | | - | - | |
| **Hypophosphatemia** | + | + | + | + | + | + | + | + | + | + | + | |
| - PO4 (mmol/L) | 1.20 | 1.40 | 1.40 | 1.10 | 1.10 | 1.10 | 1.10 | 0.9 | 0.9 | 0.9 | 1.0 | |
| - Ca, adjusted (mmol/L) | 2.60 | 2.61 | 2.51 | 2.58 | 2.55 | 2.58 | 2.52 | 2.45 | 2.45 | 2.43 | 2.39 | |
| **Hearing loss** | - | | | | | | | | | + | | + |
| **Retinal abnormality** | - | | | | | | | - | | - | | |
| **Pseudoxanthoma Elasticum** | - | - | - | - | - | - | - | - | - | - | - | - |
| **Cervical spine fusion** | - | | | | | | | | | - | | |
| Clinical Parameters | | | | | | | | | | | | |
| **Weight (kg)** | 3.50 | 3.86 | 4.20 | 4.93 | 5.37 | 5.89 | 6.50 | 6.50 | 7.10 | 7.38 | 7.80 | |
| **Hypertension** | - | - | - | - | - | - | - | - | - | - | - | |
| SBP (mmHg) | 65 | 83 | 88 | 94 | 97 | 85 | 95 | 80 | 90 | 80 | 92 | |
| DBP (mmHg) | 47 | 44 | 53 | 58 | 58 | 55 | 64 | 40 | 54 | 48 | 57 | |
| Medications and Doses | | | | | | | | | | | | |
| Pamidronate | | 0.25mg/kg x 2 doses | 0.5mg/kg x 2 doses | 1mg/kg | 1mg/kg | 1mg/kg | 1mg/kg | 1mg/kg | 1mg/kg | 1mg/kg | 1mg/kg | 1mg/kg |
| Captopril | | 0.5mg TDS | 4mg TDS | 0.5mg TDS | 0.5mg TDS | 0.5mg TDS | 0.5mg TDS | **Stopped** | | | | |
| Carvedilol | | | 0.5mg BD | 0.7mg BD | 0.7mg BD | 1mg BD | 1mg BD | 1mg BD | | 1mg BD | 1mg BD | |
| Hydrochlorothiazide | | | 2mg BD | 2mg BD | 2mg BD | 2mg BD | 2mg BD | 2mg BD | | 2mg BD | 2mg BD | |
| Hydralazine | | | 0.5mg TDS | 0.5mg TDS | 0.5mg TDS | 0.5mg TDS | 0.5mg TDS | 0.5mg TDS | | 0.5mg TDS | 0.5mg TDS | |
| Aspirin | | 15mg OM | 15mg OM | 30mg OM | 30mg OM | 30mg OM | 30mg OM | **Stopped** | | **30mg OM** | 30mg OM | |
| Phosphate (mmol/kg/day) | | | 0.2 | 0.3 | 0.3 | 0.3 | 0.32 | 0.38 | 0.44 | 0.42 | 0.48 | |

*At 37-day-old, intravenous pamidronate was initiated (0.25mg/kg/dose for 2 days) and repeated monthly for 6 months (increased to 1mg/kg), 2-monthly for 6 months, then 3-monthly.

**At 12-month-old, patient suffered a cerebrovascular ischemic event, she presented with status epilepticus and neuroimaging showed left parieto-temporal lobe acute infarct, collateral vessels formation in external and internal carotid arteries suggesting proximal stenosis, and calcifications along basilar and internal carotid arteries.

***At 16-month-old, patient developed bilateral conductive hearing loss and is on close surveillance

#Modalities of assessment - *Arterial calcifications*: evaluated with ultrasonography at 4 day old and CT angiography at 1 month old. Ultrasound renal doppler at 2nd and 6th month old to review renal artery stenosis. *Extravascular calcifications:* nephrocalcinosis seen in serial ultrasonography; intracranial calcifications seen on computed tomography scan performed during cerebral ischemic event. *Cardiac manifestations:* assessed using transthoracic echocardiogram. *Hearing:* AABR at birth, ERA/SSEP at 12 and 18 months old. *Retinopathy:* pediatric ophthalmologist at outpatient setting. *Pseudoxanthoma Elasticum*: on regular physical examination. *Cervical spine fusion*: Chest X-rays performed for other clinical indications.

**Discussion**

Our patient had cardiac dysfunction without hypertension. However, CT angiography showed significant abdominal aorta calcifications that could cause increased afterload, and coronary calcifications and intimal proliferation may not be easily visualized due to tiny vessel caliber. It is known that patients who survive beyond infancy have less significant cardiac manifestations over time. Our patient had normal LVEF by 2-month-old, suggesting that recovery of cardiac function can be rapid.

During the cerebrovascular event, neuroprotective measures were implemented, and aspirin was resumed. Our patient recovered without neurological deficit. Neurological complications in GACI are uncommon but can be reversible with early recognition and timely neurocritical care.

There is no definitive recommendation on bisphosphonate regimen for GACI. It may have to be individualized depending on clinical severity and balancing potential benefits-risks. We adopted an approach that is intensive at initiation with stepwise reduction in frequency of infusion after 6- and 12-months. It is uncertain how long the therapy should be continued and if it could prevent or delay occurrence of stroke or hearing loss.

### Disclosure

None declared

### P100

### Endocrinological Challenges and Renal Expression of Lowe Syndrome: Comprehensive Analysis of a Pediatric Series

Liliana Mejia de Beldjenna[1,2,3,4], Ana Katerine Minota[1,3], Jorge Endo[1,3], Mabel Libreros[1,3]

[1]Foundation children´s clinic Club Noel, cali, Colombia. [2]University Libre, cali, Colombia. [3]Group Investigation in pediatricas.UNILIBRE . GRINPED, CALI, Colombia. [4]COLOMBIAN COLLEGE ASSOCIATION OF PEDIATRIC ENDOCRINOLOGY.ACCEP, Bogota, Colombia

### Abstract

INTRODUCTION:

Lowe oculocerebrorenal syndrome (OCRS) is an X-linked genetic disorder caused by variants in the OCRL gene. It is characterized by congenital cataracts, neurological abnormalities, and proximal tubular dysfunction, with endocrine involvement including growth and bone-mineral metabolism abnormalities due to renal phosphorus and bicarbonate loss. In Latin America, there are few clinical series that systematically integrate the endocrinological and nephrological approaches.

OBJECTIVE:

To characterize the endocrine-renal involvement and phenotypic variability in a Colombian pediatric series with Lowe syndrome, in order to identify early patterns of involvement for diagnosis and follow-up.Analysis of a retrospective case series of pediatric patients with a clinical diagnosis of Lowe syndrome, treated at a pediatric hospital in Cali, Colombia, between January 1, 2017, and December 31, 2025.

RESULTS:

Six pediatric patients were included, five of whom were male. The average age at diagnosis was 10 months. All patients (100%) presented with global neurodevelopmental delay, hypotonia, incomplete Fanconi-type proximal tubular dysfunction (hyperchloremic metabolic acidosis, tubular proteinuria, hyperphosphaturia, and variable hypercalciuria, with preserved glomerular filtration rate (GFR >85–90 ml/min/1.73 m$^2$)), and bilateral congenital cataracts. All patients exhibited bone and mineral metabolism abnormalities, including hypophosphatemia, elevated alkaline phosphatase, and 25-hydroxyvitamin D deficiency, with normal calcium levels. One patient presented with severe hypophosphatemic rickets. In all cases, the molecular diagnosis was confirmed by alteration of the OCRL gene. Treatment consisted of citrates.

CONCLUSION:

In this pediatric series, endocrine-skeletal involvement was universal, of early onset, preceding the deterioration of glomerular renal function. The coexistence of severe short stature, hyperchloremic metabolic acidosis, and hypophosphatemia in infants with congenital cataracts constitutes a clinical pattern highly suggestive of Lowe syndrome. An integrated endocrinology-nephrology approach facilitates early recognition and timely correction of metabolic abnormalities, promoting functional and growth trajectories, even before molecular confirmation.

### Disclosure

None declared

### P101

### Hereditary Vitamin D-Resistant Rickets (HVDRR): A Case Report

Anju Jacob[1,2], M Zulf Mughal[1]

[1]Al Jalila Children's specialty hospital, Dubai, UAE. [2]Mohammed Bin Rashid University of medicine and health sciences, Dubai, UAE

### Abstract

**Background:**

Hereditary Vitamin D-Resistant Rickets (HVDRR), or Vitamin D-Dependent Rickets Type II, is a rare autosomal recessive disorder caused by mutations in the Vitamin D Receptor (VDR) gene, leading to end-organ resistance to active vitamin D. It typically presents in early childhood with severe rickets, bony deformities, and frequently, alopecia.

**Case Presentation:**

We report a case of HVDRR in a child without alopecia but with florid rickets. Bowing of the legs was noted at 9 months during his admission in view of repeated chest infections. Initially treated for nutritional vitamin D deficiency, he was referred to endocrinology at 22 months. Despite normal early development (aside from speech and motor delays) and adequate dietary calcium, he had recurrent respiratory infections. Treatment for hypophosphatemic rickets (oral phosphate and alphacalcidol) failed to improve clinical, radiological, or biochemical signs of rickets. Investigations revealed markedly elevated PTH and 1,25-dihydroxyvitamin D levels, suggesting HVDRR, which was later supported by genetic testing.

**Management:**

Management involved continuous intravenous calcium infusions under cardiac monitoring in a High Dependency Unit (HDU). Monitoring incidentally identified Wolff-Parkinson-White syndrome and cardiomyopathy. Following IV therapy and gradual introduction of cinacalcet (Fig1: normalisation of parathyroid hormone levels), his rickets resolved, and he was successfully weaned off infusions and currently is on only oral calcium, magnesium, vitamin D as well as cinacalcet. Genetic counseling was provided to the family regarding the 1 in 4 recurrence risk.

**Conclusion:**

This case highlights that HVDRR, characterized by elevated 1,25-dihydroxyvitamin D and resistance to standard vitamin D therapy, is often misdiagnosed. The absence of alopecia can delay recognition. Successful treatment may require high-dose or intravenous calcium regimens. The lack of established treatment guidelines can lead to poor disease control, significant morbidity, and reduced quality of life, underscoring the need for early genetic diagnosis and standardised therapeutic protocols

Fig1: normalisation of parathyroid hormone levels

### Disclosure

None declared

### P102

### Unmasking Primary Hyperparathyroidism Following Vitamin D Repletion in an Adolescent Male with Obesity and Bilateral Slipped Capital Femoral Epiphysis

Melody Shi, Andrea Estrada

Children's National Hospital, Washington, USA

### Abstract

**Background:**

Bilateral slipped capital femoral epiphysis (SCFE) in adolescents should prompt evaluation for underlying endocrine and metabolic bone disease. While obesity is a common risk factor for SCFE, primary hyperparathyroidism (PHPT), though rare in pediatrics, is a recognized cause of physeal instability. Severe vitamin D deficiency may mask biochemical manifestations of PHPT, delaying diagnosis until repletion unmasks overt disease.

**Case Presentation:**

A 13-year-old male with a history of obesity presented with bilateral SCFE requiring surgical repair. Further history revealed chronic bilateral lower extremity pain for one year prior to presentation, initially attributed to mechanical factors related to body habitus. Radiographs of the knees demonstrated diffusely demineralized bone with sclerotic metaphyseal bands, raising concern for an underlying metabolic bone disorder. Initial laboratory evaluation revealed Vitamin D 25 hydroxyvitamin D <4.0 ng/mL with calcium of 10.3 mg/dL and phosphorus 2.8 mg/dL.

The patient was started on 50, 000 IU weekly vitamin D repletion. Two weeks later, his calcium rose to 13.7 mg/dL with inappropriately elevated parathyroid hormone (PTH) 544 pg/mL. The presence of hypophosphatemia with normocalcemia on initial evaluation represented an early biochemical clue to an underlying PTH-related disorder, which was obscured by concurrent severe vitamin D deficiency. Sestamibi parathyroid imaging identified a parathyroid adenoma, confirming the diagnosis of primary hyperparathyroidism.

**Management and Outcome:**

Vitamin D supplementation was discontinued, and the patient was managed medically with cinacalcet until definitive surgical management with parathyroidectomy. Orthopedic recovery following SCFE repair was uncomplicated. Ongoing bone health management focused on normalization of calcium–phosphate homeostasis and prevention of further skeletal morbidity.

**Discussion:**

This case highlights the importance of evaluation for potential underlying metabolic etiologies of SCFE, even when known risk factors are present, and underscores the importance of recognizing prodromal skeletal symptoms and radiographic evidence of impaired mineralization. Correction of severe vitamin D deficiency may unmask occult PHPT, precipitating hypercalcemia. SCFE is a distinct skeletal manifestation of pediatric PHPT, highlighting the need for endocrine evaluation in atypical or bilateral presentations.

**Conclusion:**

Adolescents presenting with bilateral SCFE warrant comprehensive metabolic bone and endocrine evaluation. Careful biochemical assessment and monitoring during vitamin D repletion are essential to avoid diagnostic delays, potential complications of repletion and identify treatable causes of significant skeletal disease.

**Table 1.**

| Lab | Baseline | Day 14-Post Vitamin D3 Repletion |
|---|---|---|
| | | |
| Glucose (mg/dL) | 97 | 119 |
| BUN (mg/dL) | 7 | 9 |
| Cr (mg/dL) | 0.3 | 0.37 |
| Na (mmol/l) | 139 | 136 |
| K (mmol/L) | 3.8 | 4.3 |
| Cl (mmol/L) | 110 | 102 |
| CO2 (mmol/L) | 21 | 20 |
| Ca (mg/dL) | 10.3 | **13.7** |
| Albumin (gm/dL) | | 4.5 |
| Alkaline Phosphatase (U/L) | 595 | 570 |
| Phosphorus (mg/dL) | 2.8 | 3.8 |
| 25 – Hydroxyvitamin D (ng/mL) | < 4 | 7.2 |
| PTH (pg/mL) | | **544** |
| TSH (mcU/mL) | 1.58 | |
| Free T4 (ng/dL) | 0.98 | |

### Disclosure

None declared

### P103

### Prolonged QTc as the Presenting Feature for Parathyroid Disorders

Chaitanya Sambangi, Andrea Estrada

Children's National Hospital, Washington DC, USA

### Abstract

BACKGROUND:

We describe two pediatric cases of parathyroid hormone (PTH) disorders with delayed diagnosis due to atypical yet well-described symptoms of hypocalcemia, highlighting the importance of maintaining a broad differential diagnosis for common chief complaints.

PRESENTING PROBLEM:

Case 1: A 14-year-old female presented to cardiology after two episodes of syncope during physical activity at school, with rapid return to baseline. Initial electrocardiogram (EKG) revealed a prolonged QTc of 470 ms. A third episode with dizziness and altered mental status prompted initiation of beta blocker therapy. Despite treatment, she experienced additional episodes prompting loop recorder implantation and eventually ED evaluation where QTc was prolonged to 519 ms. Laboratory evaluation revealed hypocalcemia, hyperphosphatemia, low 25-hydroxyvitamin D, low urine calcium-to-creatinine ratio, normal 1,25-dihydroxyvitamin D, and significantly elevated intact parathyroid hormone (iPTH).

Case 2: A 7-year-old male was referred to genetics for evaluation of congenital hearing loss. A screening EKG identified prolonged QTc, prompting referral to cardiology. Given recent experience with case 1, cardiology conducted laboratory screening, revealing hypocalcemia, hyperphosphatemia, low 25-hydroxyvitamin D, and inappropriately low iPTH.

CLINICAL MANAGEMENT:

In case 1, due to acute symptoms, the patient required inpatient admission for hypocalcemia management, including IV calcium gluconate, initiation of oral elemental calcium, calcitriol, and cholecalciferol. QTc normalized by discharge, allowing discontinuation of beta blocker therapy. iPTH remained inappropriately elevated after normalization of vitamin D deficiency. In case 2, oral elemental calcium, calcitriol, and cholecalciferol were initiated outpatient. After two months of therapy, QTc normalized.

DISCUSSION:

Case 1 exhibits PTH resistance (pseudohypoparathyroidism), a rare cause of impaired calcium retention and phosphorus excretion. Although urinary calcium was appropriately low in the setting of hypocalcemia, elevated serum phosphorus reflected ineffective PTH action. Case 2 illustrates inadequate PTH secretion despite significant hypocalcemia. Genetic testing revealed a GATA3 mutation, associated with hearing loss and hypoparathyroidism.

Due to wide recognition that hypocalcemia results in prolonged QTc, routine evaluation of electrolytes is imperative but may be missed due to framing bias. Deviations from expected clinical progression or atypical responses to treatment should prompt reassessment of the initial diagnosis, as common presentations may reflect rare endocrine etiologies.

TABLE

| Lab | Case 1 Presentation | Case 1 One Year After Diagnosis | Case 2 Presentation | Case 2 One Year After Diagnosis |
|---|---|---|---|---|
| Calcium | < 5 mg/dL (Low) | 8.6 mg/dL | 7.2 mg/dL | 9.0 mg/dL |
| Albumin | 3.4 mg/dL | 4.6 mg/dL | 4.2 mg/dL | 4.5 mg/dL |
| Corrected Calcium | | 8.1 mg/dL | 7 mg/dL | 8.6 mg/dL |
| Magnesium | 1.6 mg/dL | | | |
| Phosphorus | 8.6 mg/dL (High) | 4.6 mg/dL | 7.3 mg/dL (High) | 4.9 mg/dL |
| Alkaline Phosphatase | 196 IU/mL | | | |
| 25-hydroxyvitamin D | 8.8 ng/mL (Low) | 23 ng/mL | 6 ng/mL (Low) | 27 ng/mL |
| 1,25-dihydroxyvitamin D | 32 pg/mL | | | |
| Urine Calcium/Creatinine Ratio | < 0.05 mg/g (Low) | | 6 mg/g (Low) | 29 mg/g |
| Intact Parathyroid Hormone (iPTH) | 1,084 pg/mL (High) | 599 pg/mL | 17 pg/mL (Low) | |

### Disclosure

None declared

### P104

### Intramuscular Adiposity and Bone Density in Childhood: Patterns in Healthy Children, and Alterations in Type 1 Diabetes and Thalassemia

Sharvani Patil, Neha Kajale, Rubina Mandlik, Anuradha Khadilkar

Hirabai Cowasji Jehangir Medical Research Institute, Jehangir Hospital, Pune, India

### Abstract

**Background:**

Intramuscular adipose tissue (IMAT) is an emerging marker of poor muscle quality and is associated with adverse metabolic and skeletal outcomes. Peripheral quantitative computed tomography (pQCT) derived muscle density provides a surrogate measure of intramuscular adipose tissue (IMAT). However, age- and sex-specific data on IMAT assessed by pQCT in healthy children and chronic disease states such as type 1 diabetes mellitus and thalassemia remain limited in the Indian population.

**Objective:**

To characterise age- and sex-specific patterns of pQCT-derived intramuscular fat. To compare these patterns between healthy children and those with chronic disorders such as type 1 diabetes mellitus(T1D) and β-thalassemia major, and their association with bone health in all three cohorts.

**Methods:**

Muscle cross-sectional area and muscle density were assessed using peripheral quantitative computed tomography (pQCT; XCT-2000, Stratec Inc., Pforzheim, Germany). Intramuscular adipose tissue (IMAT%) was estimated from pQCT muscle density. Sex-wise analyses were performed among the three cohorts, i.e., healthy controls (n = 961; male children = 501), children with type 1 diabetes mellitus (n = 289; male children = 136), and children with β-thalassemia major (n = 179; male children = 105), using SPSS.

**Results:**

Among the three cohorts and all age groups, male children had significantly higher BMI, bone density, geometry, and strength than female children ($p < 0.05$). In contrast, Intramuscular adipose tissue (IMAT) was higher in female children than male children across all groups (healthy: 34.12 ± 9.63 vs 25.68 ± 14.49; T1DM: 34.99 ± 9.09 vs 21.44 ± 11.32; thalassemia: 43.89 ± 7.71 vs 32.92 ± 8.58; all $p< 0.05$). Children with thalassemia had higher IMAT than healthy controls and those with T1DM in both sexes ($p< 0.05$). IMAT was negatively associated with bone density, geometry, and strength (SSIPOL) across all groups ($p< 0.05$) except among female children with thalassemia. (Table 1).

**Conclusion:**

Although female children exhibited higher IMAT across groups, the expected association between IMAT and bone outcomes was absent in girls with β-thalassemia. This suggests that the relationship between intramuscular fat and skeletal health may vary by disease context.

Table 1: Correlations of IMAT with bone strength, density, and geometry parameters

| *Table: Partial Correlations of IMAT with Bone strength, Density, and Geometry parameters* | | | | | | | |
|---|---|---|---|---|---|---|---|
| | Parameters | Bone Strength | Bone Density | | Bone Geometry | | |
| Gender | Correlations with IMAT | **SSIPOL2 (mm3)** | **Trabecular Density (mg/cm3)** | **Cortical Density (mg/cm3)** | **Cortical Thickness (mm)** | **Periosteal circumference (mm)** | **Endosteal circumferenc (mm)** |
| Healthy (Boys) | Correlation | **-0.362** | **-0.147** | 0.086 | **-0.132** | **-0.348** | **-0.222** |
| | Significance (2-tailed) | **<.001** | **0.001** | 0.057 | **0.003** | **<.001** | **<.001** |
| | df | 489 | 489 | 489 | 489 | 489 | 489 |
| T1DM (Boys) | Correlation | **-0.536** | -0.143 | 0.052 | **-0.23** | **-0.529** | **-0.269** |
| | Significance ((2-tailed) | **<.001** | 0.223 | 0.661 | **0.049** | **<.001** | **0.02** |
| | df | 72 | 72 | 72 | 72 | 72 | 72 |
| Thalassemia (Boys) | Correlation | **-0.578** | **-0.308** | -0.243 | **-0.396** | **-0.335** | -0.051 |
| | Significance (2-tailed) | **<.001** | **0.014** | 0.055 | **0.001** | **0.007** | 0.693 |
| | df | 61 | 61 | 61 | 61 | 61 | 61 |
| Healthy (Girls) | Correlation | **-0.349** | -0.072 | 0.078 | **-0.115** | **-0.348** | **-0.206** |
| | Significance (2-tailed) | **<.001** | 0.122 | 0.095 | **0.014** | **<.001** | **<.001** |
| | df | 454 | 454 | 454 | 454 | 454 | 454 |
| T1DM (Girls) | Correlation | **-0.287** | -0.135 | 0.021 | -0.079 | **-0.281** | **-0.255** |
| | Significance (2-tailed) | **0.007** | 0.208 | 0.845 | 0.463 | **0.008** | **0.017** |
| | df | 86 | 86 | 86 | 86 | 86 | 86 |
| Thalassemia (Girls) | Correlation | -0.118 | **-0.391** | -0.084 | -0.258 | 0.149 | 0.264 |
| | Significance (2-tailed) | 0.519 | **0.027** | 0.648 | 0.154 | 0.415 | 0.144 |
| | df | 30 | 30 | 30 | 30 | 30 | 30 |
| Controlling variables: Age (years) and BMI, figures in bold indicate $p<0.05$ | | | | | | | |

### Disclosure

None declared

### P105

### Development of Muscle Function in Children with XLH Under Burosumab Treatment

Ibrahim Duran[1,2], Mirko Rehberg[2], Johanna Heistermann[3], Lisa Neuburg[2], Heike Hoyer-Kuhn[2], Oliver Semler[2], Susanna Reincke[2]

[1]Center for Pediatric and Adolescent Rehabilitation, University Hospital of Cologne, Cologne, Germany.
[2]Department of Pediatrics and Adolescent Medicine, University Hospital of Cologne, Cologne, Germany.
[3]Department of Pediatrics and Adolescent Medicine, University Hospital of Cologne, Cologne, Germany, Cologne, Germany

### Abstract

### Objectives:

X-linked hypophosphatemia (XLH) is a hereditary disorder caused by mutations in *PHEX*, leading to elevated levels of fibroblast growth factor 23 (FGF23). Excess FGF23 results in renal phosphate wasting and consequent impairment of bone mineralization. Clinically, patients usually present with skeletal deformities, short stature, dental abnormalities, and muscle weakness. Burosumab, a monoclonal antibody targeting FGF23 was approved as medical treatment in 2018 and has shown to correct phosphate wasting. This study investigated the effects of Burosumab on muscle function in children with XLH, in addition to its known skeletal benefits.

### Methods:

In this prospective observational study, muscle function and body height development were assessed in 10 children with XLH after initiation of Burosumab therapy. All patients had previously received conventional treatment. Muscle function was evaluated using a single two-leg jump (S2LJ) on a ground reaction force plate at baseline, one year, and two years after treatment initiation. Maximum velocity, maximum power/body mass, maximum force/body mass, and nerve-muscle index (NMI) were calculated and converted into age- and sex-adjusted Z-scores based on unaffected children. Longitudinal changes were analyzed using mixed-effects models with time as fixed effect.

### Results:

10 patients (8 females) with a mean age of 10.8 ± 2.7 years were included. At baseline, all muscle function parameters and body height were markedly below reference values. After one year of Burosumab therapy, significant improvements were observed in maximum power/body mass ($\beta = 0.95$, $p = 0.045$) and maximum force/body mass ($\beta = 0.77$, $p = 0.030$), whereas maximum velocity and nerve-muscle index remained unchanged. After two years, no further improvements in muscle parameters were observed, although values remained higher than baseline. Body height Z-scores increased significantly after two years of therapy ($\beta = 0.46$, $p = 0.011$).

### Conclusions:

Prior to therapy, this cohort exhibited muscle function substantially below the mean of unaffected children. Burosumab treatment led to significant improvements in muscle force and power during the first year, indicating enhanced muscle strength and approaching normalization of function, while the NMI remained stable. Increases of functional parameters plateaued after two years, whereas height Z-scores increased significantly. Larger and longer-term studies are needed to confirm these findings.

### Disclosure

None declared

### P106

### Vertebral Fractures and Muscle Function in Glucocorticoids-Treated Patients with Duchenne Muscular Dystrophy: A Cohort Study

Chiara Arpaia[1,2], Anna Capasso[1,2], Chiara Panicucci[3], Marianna Villa[2], Consolato Gulli'[4], Agnese Repetto[3], Giorgia Coratti[1,2], Gianpaolo Cicala[1,2], Anna Marzoli[5], Natascia Di Iorgi[6], Claudio Bruno[3], Marika Pane[1,2], Leanne Ward[7], Eugenio Mercuri[1,2]

[1]Pediatric Neurology Unit, Catholic University, Rome, Italy. [2]Centro Clinico Nemo, U.O.C. Neuropsichiatria Infantile Fondazione Policlinico Universitario Agostino Gemelli IRCCS, Rome, Italy. [3]Center of Translational and Experimental Myology, IRCCS Istituto Giannina Gaslini, Genoa, Italy. [4]Advanced Radiology Center, Department of Diagnostic Imaging and Radiation Oncology, Fondazione Policlinico Universitario A. Gemelli IRCCS,, Rome, Italy. [5]Radiology Unit, IRCCS, IRCCS Istituto Giannina Gaslini, Genoa, Italy. [6]Department of Pediatrics, IRCCS Istituto Giannina Gaslini,, Genoa, Italy. [7]Division of Endocrinology and Metabolism, Children's Hospital of Eastern Ontario and University of Ottawa, Ottawa, Canada

### Abstract

### Objectives:

To describe the prevalence of vertebral fractures (VFs) in Duchenne Muscular Dystrophy (DMD) and to establish the role of several risk factors, focusing on functional status.

### Methods:

Cross-sectional, single-centre study to assess the number and site of VF in association with anthropometric, clinical, functional and densitometric (total body less head bone mineral density [TBLH BMD] Z-scores by Dual Energy X-ray Absorptiometry [DXA]) assessments. Logistic and linear regression analyses were conducted to identify factors associated with prevalent VFs and predictors of the Spinal Deformity Index (SDI, sum of the Genant Grades) in children with DMD.

### Results:

Of the 149 individuals with DMD (7-26 years, mean ± SD age 14.76 ± 4.28) were studied, 62/149 (42%) with ≥1 VF.  Thirty-one of the 62 (50%) were ambulant, and 31 (50%) were non-ambulant DMD individuals. Prevalence was   not associated with functional scores ($p > 0.10$). Higher total body BMD Z-score was associated with a 51% reduction in the odds of VFs (OR=0.49, 95% CI [0.30, 0.77], p=0.003). In a linear regression analysis predicting spinal deformity index (SDI), only height Z-score was significantly associated with SDI, such that greater height was related to a lower spinal deformity index (b=–0.57, SE=0.25, t=–2.28, p=0.024). In the ambulant subgroup, a lower BMI was associated with a reduced risk of VFs. In the overall cohort, each one-year delay in starting glucocorticoids (GCs) reduced the risk of VFs by 27% (p=0.007), and each additional unit increase in TBLH BMD Z-score reduced the risk of VFs by 54% (p=0.0007).

### Conclusion:

Our results suggest that functional status alone may not be a reliable predictor for identifying patients at risk of VFs and confirm the association with known risk factors, such as early initiation of GCs therapy and low BMD Z-scores (in this case, for total body), highlighting the need to implement careful surveillance of possible VFs from the time of GC initiation.

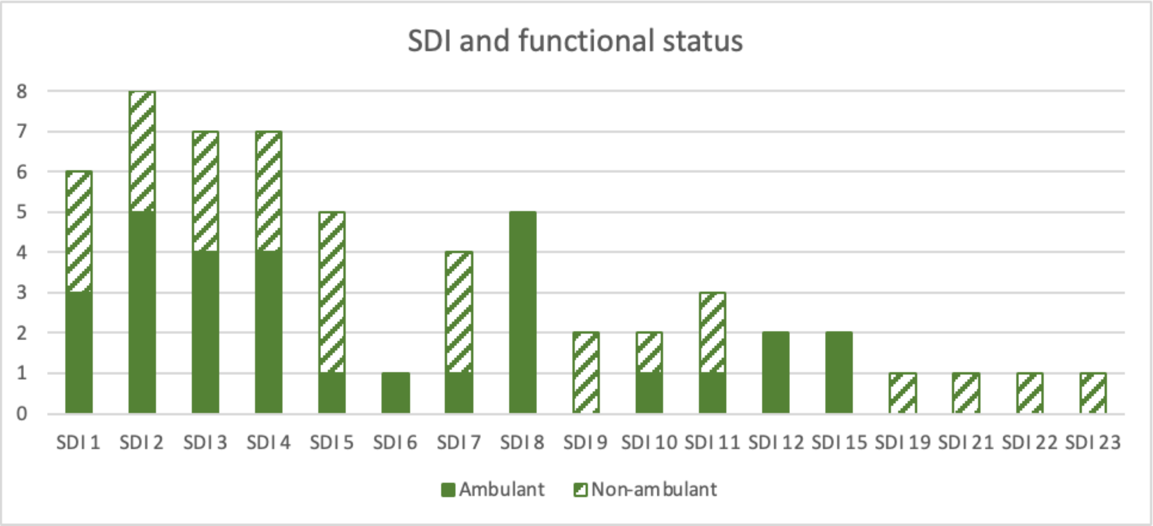

**Fig. 1** Spinal Deformity Index (SDI) and functional status in the Vertebral Fractures (VFs) cohort.

### Disclosure

GC participated to Advisory Board for Italfarmaco, ROCHE and Sarepta; CB, participated to Advisory Board for ROCHE; EM participated to Advisory Board for ROCHE, Sarepta, PTC, Italfarmaco, Pfizer, Dyne, NS Pharma, Santhera, Avidity, Entrada, Edgewise and Solid. MP participated to Advisory Board for ROCHE, Sarepta, PTC, Italfarmaco, Pfizer and Dyne

### P107

### Altered One-Year Changes in Bone Microarchitecture and Estimated Strength in Children and Youth with Type 1 Diabetes: An HR-pQCT Study

Ali Rezaei[1], Munier A Nour[2], James D Johnston[3], Saija A Kontulainen[1]

[1]College of Kinesiology, University of Saskatchewan, Saskatoon, Canada. [2]College of Medicine, University of Saskatchewan, Saskatoon, Canada. [3]College of Engineering, University of Saskatchewan, Saskatoon, Canada

### Abstract

**OBJECTIVES:**

To compare one-year changes in bone and muscle outcomes between children and adolescents with type 1 diabetes (T1D) and their typically developing peers (TDP), and to evaluate whether physical activity and muscle-related measures contribute to annual bone changes.

**METHODS:**

We compared changes in bone and muscle outcomes between children with T1D (N=20, 13 female, mean age at baseline: 12.3, SD 2.7) and TDP (N= 30, 18 female, mean age at baseline: 10.8, 1.7). We annualized 1-year changes for HR-pQCT derived trabecular and cortical microarchitecture, density, and estimated strength at the distal radius and tibia, as well as pQCT assessed muscle area and bone outcomes at the forearm and lower leg. Physical activity, grip strength, and long jump performance were recorded. We used hierarchical regression models to test if T1D status contributed to bone outcomes, after adjusting for sex, baseline maturity offset and body mass. We explored the role of baseline muscle and physical activity measures to annualized bone changes that differed between T1D and TDP.

**RESULTS:**

At the distal radius, T1D status was associated with a smaller gain in adjusted trabecular thickness ($\beta$ = −3.35, 95% CI: −6.69 to −0.01), as illustrated for both groups relative to baseline maturity (Fig 1). T1D status was also associated with a greater gain in trabecular number (0.12, 0.02 to 0.22), and a smaller reduction in load-to-strength ratio (0.08, 0.01 to 0.14). At the tibia, T1D status was associated with smaller gains in shaft cortical density (−0.34, −64.69 to −6.71), distal tibia failure load (−0.55, −1.04 to −0.05), and stiffness (−11.33, −21.20 to −1.46). In models with group and confounders, muscle area (0.76, 0.22 to 1.31) and grip force (0.38, 0.03 to 0.73) were positively associated with radius trabecular thickness change.

**CONCLUSIONS:**

Children and youth with T1D had smaller gains in trabecular thickness, cortical density, and bone strength. Greater baseline muscle area and grip strength were positively associated with gain in radius trabecular thickness. Further follow-up is needed to examine bone development in T1D and to clarify the interplay between muscle and bone.

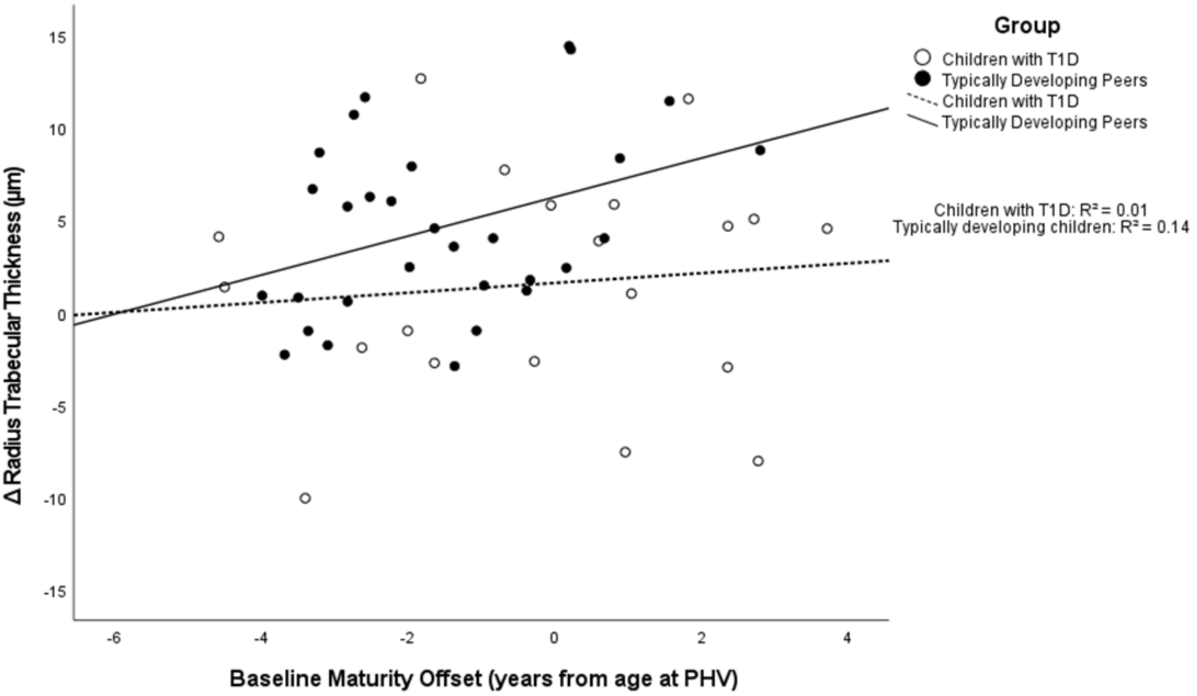

Figure 1. Change in trabecular thickness at the distal radius relative to baseline maturity offset in both groups.

### Disclosure

None declared

### P108

### Skeletal Adaptation and Bone Geometry in Pre-Pubertal Rural Indian Children with a Sarcopenia-Like Phenotype

Sharvani Patil, Rubina Mandlik, Dipali Ladkat, Vivek Patwardhan, Chidvilas More, Nikhil Shah, Anuradha Khadilkar

Hirabai Cowasji Jehangir Medical Research Institute, Jehangir Hospital, Pune, India

### Abstract

Background:

Sarcopenia is primarily researched in adults and currently lacks a singular, global definition in children. Its application to children remains undefined due to their ongoing growth and development, as well as the dynamic nature of muscle mass and strength. There is limited research investigating the sarcopenia-like phenotype in children and its implications for bone health, particularly among seemingly healthy children. Studies indicate that rural Indian children often experience compromised bone health and reduced muscle function in comparison to their urban peers, which can negatively impact the accrual of bone. Furthermore, if these rural children exhibit a sarcopenia-like phenotype, they may face additional disadvantages. Accordingly, this study examined the association between a sarcopenia-like phenotype and bone density and geometry in 8–10-year-old rural Indian children.

Methods:

A cross-sectional was conducted among 426 rural Indian children aged 8-10 years (boys= 250). Anthropometric measurements were assessed. Body composition and bone health were evaluated using the Lunar iDXA system (GE Healthcare, Encore version 18) and pQCT (XCT-2000 scanner, Stratec, Inc., Pforzheim, Germany), respectively. Hand grip strength was estimated using the Jamar hand dynamometer. A sarcopenia-like phenotype was defined as children exhibiting grip strength and appendicular skeletal muscle mass (ASMI) below the 10th percentile when compared to a reference population.

Results:

The prevalence of a sarcopenia-like phenotype within the population was found to be 16.9%. Assessment of bone volumetric density parameters and ulnar length-adjusted bone geometry parameters revealed that trabecular density (μ=168.5 vs 177.1), periosteal circumference (μ=32.2 vs 34.5) and endosteal circumference (μ=25.5 vs 26.6) were significantly lower among children who exhibited a sarcopenia-like phenotype as compared those without sarcopenia.

Conclusion:

Bone parameters, as assessed by trabecular density and bone geometry, which are metabolically active and responsive to mechanical loading, were lower in children exhibiting a sarcopenia-like phenotype. These findings suggest less favourable skeletal adaptation during growth, which may have implications for peak bone mass attainment in this population.

Figure 1 – pQCT Muscle and Bone area scans (non-dominant hand) for No Sarcopenia vs Sarcopenia-like phenotype. Image 1 showing greater muscle-bone cross-sectional area and higher muscle-bone density compared to Image 2.

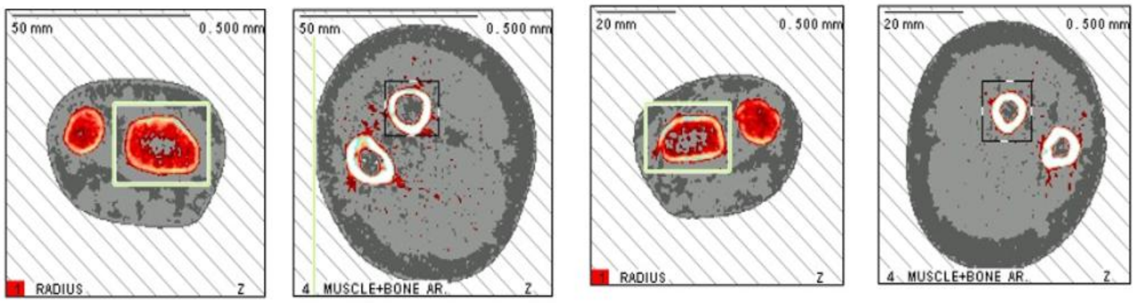

Image 1- No Sarcopenia

Image 2- Sarcopenia-like phenotype

### Disclosure

None declared

### P109

### Deficits in Areal and Volumetric Bone Mineral Density Persist After Adjustment for Body Size in a Cohort of Young Individuals with Turner Syndrome

Sabina Strashun[1], Vaneeta Bamba[1,2], Andrea Kelly[1,2], Jacquelyn Hatch-Stein[1,2], Denise Gruccio[1], David Weber[1,2]

[1]The Children's Hospital of Philadelphia, Philadelphia, USA. [2]The Perelman School of Medicine at the University of Pennsylvania, Philadelphia, USA

### Abstract

### Objectives:

To characterize relationships between bone density, lean mass, and body size in individuals with Turner Syndrome (TS) compared to healthy peers.

### Methods:

Dual energy X-ray absorptiometry (DXA) data from 92 individuals with TS (aged 14-33 years) were extracted from the clinical record while DXA and high-resolution peripheral quantitative computed tomography (HR-pQCT) were obtained prospectively in a pilot study of individuals with TS and age-matched controls (n=10/group, aged 16-23 years). Areal bone mineral density (aBMD), appendicular lean soft tissue mass (ALSTM), and leg lean mass (LLM) from DXA were converted into Z-scores for age and sex and adjusted for height Z-score (HAZ) using Bone Mineral Density in Children Study reference data. Tibial and radial total, cortical, and trabecular volumetric (vBMD) and cross-sectional area (CSA) were obtained from HR-pQCT. The clinical TS cohort was compared to reference data by one-sample t-test (expected mean=0); linear regression was used to test for pilot group differences following adjustment for lean mass and body size parameters.

### Results:

Mean height-Z of TS individuals in the clinical cohort was -2.14±1.19. Median [interquartile range] LS and WBLH aBMD-Z and ALSTM-Z increased following HAZ-adjustment (-0.96 [-1.89,-0.14] vs -0.41 [-1.44,0.36]; -2.18 [-2.88,-1.29] vs -1.55 [-1.95,-0.45]; and -2.12 [-3.01,-1.43] vs -0.98 [-1.66,-0.32], respectively), Fig.1. Adjusted parameters remained significantly lower than the reference population, ($p<0.01$). Mean HAZ was lower in pilot TS (-1.92±0.93) vs controls (-0.50±0.62), p=0.0008. Tibial total distal vBMD (β=-55.1, 95%CI:-98.1 to -12.2), p=0.015), trabecular vBMD (β=-46.1, 95%CI:-73.1 to -19.2), p=0.002), and proximal cortical vBMD (β=-25.9, 95%CI:-44.2 to -7.5, p=0.009) were lower in TS vs controls. Differences remained significant following adjustment for leg-length and LLM. Proximal cortical CSA (β=-40.3, 95%CI: -63.1 to -17.5, p=0.002) was also lower in TS, but was dampened with adjustment for leg-length and LLM (β=-29.8, 95%CI: -63.3 to 3.7, p=0.07). Total and trabecular tibial CSA did not differ from controls. Findings at the radius were similar.

### Conclusions:

Young individuals with TS had cross-sectional deficits in areal and volumetric BMD which were attenuated but persisted following adjustment for body size and lean mass. Further studies to investigate clinical significance of these findings on bone health are warranted.

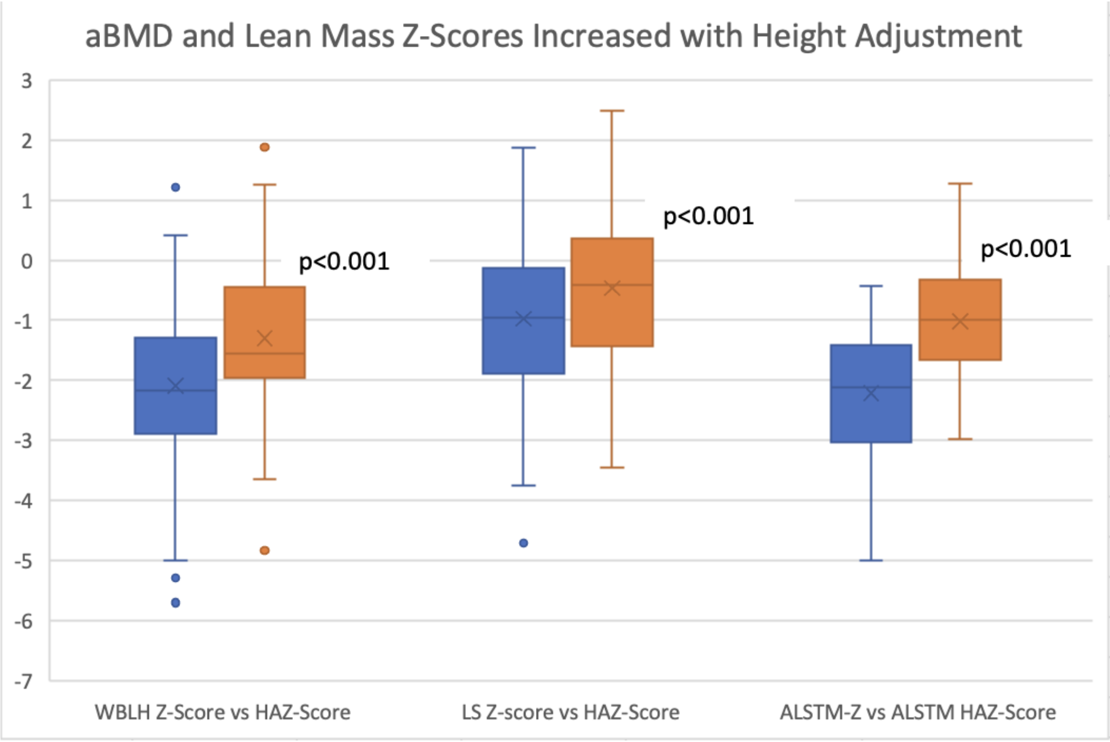

**Disclosure**

Disclosures: DRW has been consultant for Ascendis, Catalyst, and Merck. None of the other authors have any relevant disclosures to report

### P110

### Total Body and Appendicular Lean Mass by Dual-energy X-ray Absorptiometry are Highly Associated with Muscle Function and Bone Mineral Density in Becker Muscular Dystrophy

Rana Halloun[1], Stefan Jackowski[2], Maya Scharke[2], Utkarsh J Dang[3], Fahd Alshammri[1], Jinhui Ma[4], Ken Gaither[5], Thomas Fuerst[5], Hugh McMillan[1], Kerry Siminoski[2], James MacDougall[6], Joanne Donovan[6], Leanne Ward[1]

[1]Children's Hospital of Eastern Ontario, Ottawa, Canada. [2]Children's Hospital of Eastern Ontario Research Institute, Ottawa, Canada. [3]Carleton University, Ottawa, Canada. [4]McMaster University, Hamilton, Canada. [5]Clario, Philadelphia, USA. [6]Edgewise Therapeutics, Colorado, USA

### Abstract

### Objectives:

Becker muscular dystrophy (Becker) is an X-linked disorder caused by decreased or dysfunctional dystrophin. Disease progression occurs later and is more heterogeneous than Duchenne muscular dystrophy. Data are scarce on the skeletal phenotype of Becker, although low-trauma fractures are reported. Dual-energy X-ray absorptiometry (DXA)-derived lean mass may complement functional assessments and inform the areal bone mineral density (aBMD) phenotype.

### Methods:

Data on ambulatory steroid-naive adolescents and adults with Becker enrolled in a multicenter phase 2 clinical trial (NCT05291091) were analyzed in this cross-sectional study. Participants underwent motor assessments, including 10-meter walk/run test (10MWRT), time-to-rise test (TTR), and North Star Ambulatory Assessment (NSAA). DXA was performed to assess body composition with height-adjusted lean and fat mass and spine and total hip aBMD. In adolescents, spine aBMD was size corrected to bone mineral apparent density (BMAD). Values were expressed as age- and sex-matched z-scores.

### Results:

Twenty-eight adolescents, age (median, [interquartile range, IQR]) 15.0 [14.0-16.0] years and 38 adults, age 28.5 [24.3-37.8] years, were included. Greater total body (TB) and lower limb (LL) lean mass indices (LMI) were associated with better motor function, whereas TB and LL fat mass indices (FMI) showed inverse associations, explaining 20–75% of variance. In both age groups, the mean total hip aBMD z-scores were significantly below the healthy mean, while lumbar spine aBMD and BMAD did not differ. Sixteen (57%) adolescents and 12 (31.5%) adults had total hip aBMD z-scores below the expected range for age (z-score ≤ -2.0). Multivariable linear regression models including either TB- or LL-LMI, along with the respective FMI and age, explained 26–49% of the variance in total hip aBMD. TB-LMI and LL-LMI were positively associated with total hip aBMD z-scores in both age groups.

### Conclusion:

DXA-derived height-adjusted lean mass is a complementary marker of motor function in Becker and may be explored as a clinical endpoint for evaluating muscle-preserving therapies. Total hip aBMD appeared particularly affected in ambulatory treatment-naive individuals with Becker, with values significantly below the healthy mean in both age groups, suggesting sensitivity to myopathic changes. These findings underscore the importance of routine bone health surveillance in Becker.

### Disclosure

Rana Halloun: No conflict of interest. Stefan Jackowski: No conflict of interest. Maya Scharke: No conflict of interest. Utkarsh Dang: Participated in clinical trials with ReveraGen and has been a consultant to ReveraGen, Lupin. Fahd Alshammri: No conflict of interest. Jinhui Ma: No conflict of interest. Ken Gaither: Employee and equity holder at Clario, Inc. Thomas Fuerst: Employee and equity holder at Clario, Inc. and consultant to Edgewise. Hugh J. McMillan: Consultant for Roche, Novartis, Kye Pharma, Regenxbio and Solid Biosciences. He has been a site principal investigator for clinical trials (funds to institution) sponsored by: Roche, Novartis, Biogen, Sarepta, Italfarmaco, Dyne Therapeutics, PepGen, Regenxbio, Pfizer and Reveragen. Kerry Siminoski: No conflict of interest. James MacDougall and Joanne Donovan: Employees of Edgewise Therapeutics and hold stock/stock options. Leanne M. Ward: Participated in clinical trials with Roche, Santhera, ReveraGen and Edgewise, and has been a consultant to Roche, Santhera, Quince, Kye, and Grunenthal, with funds to Dr. Ward's institution.

### P111

### Age and Sex Specific Reference Data for Appendicular Skeletal Mass Index (ASMI) for Indian Children and Adolescents

Rubina Mandlik, Sharvani Patil, Vaman Khadilkar, Anuradha Khadilkar

Hirabai Cowasji Jehangir Medical Research Institute, Pune, India

### Abstract

**OBJECTIVES:** There is a lack of a uniform definition for the recently identified paediatric sarcopenia due to the absence of normative data for skeletal muscle mass. Thus, this study aimed to establish age and sex specific reference values for appendicular skeletal muscle mass index (ASMI) for Indian children and adolescents.

**METHODS:** This was a cross-sectional study involving 1157 (599 boys) healthy participants aged 1 to 18 years from Pune, India, conducted between November 2017 and July 2022. Length / height and weight were measured by standard procedures. Appendicular skeletal mass (ASM) in kg was computed as the sum of arm and leg lean mass assessed by the GE-Lunar iDXA narrow-angle fan beam scanner. ASMI was calculated as ASM in kg divided by length / height in meters squared. Age and sex-specific reference percentile curves were generated for ASMI using the LMS method.

**RESULTS:** Figure 1 illustrates the reference centiles for ASMI for boys (fig 1a) and girls (fig 1b). Distinct sex-specific growth patterns were observed. Percentile curves for boys dipped between the ages of 3 to 5 years following which they increased steadily. Percentile curves for girls increased between the ages of 5 to 9 years and then subsequently plateaued. Median ASMI values were higher in boys than in girls across all ages, except among 8 to 10 year olds, where girls had higher values. In boys, median ASMI increased by an average of 4% from 3 to 7 years after which the rate of increase slowed. For girls the median ASMI increased by 4% from 3 to 9 years, after which the curves showed minimal further increase.

**CONCLUSIONS:** We have established age and sex specific references for appendicular skeletal muscle mass index for Indian children and adolescents. These reference data provide a population-specific framework for evaluating skeletal muscle development and may be used for early identification of paediatric sarcopenia.

**Figure 1: Reference Centiles for Appendicular Skeletal Mass Index (ASMI – kg/m$^2$)**

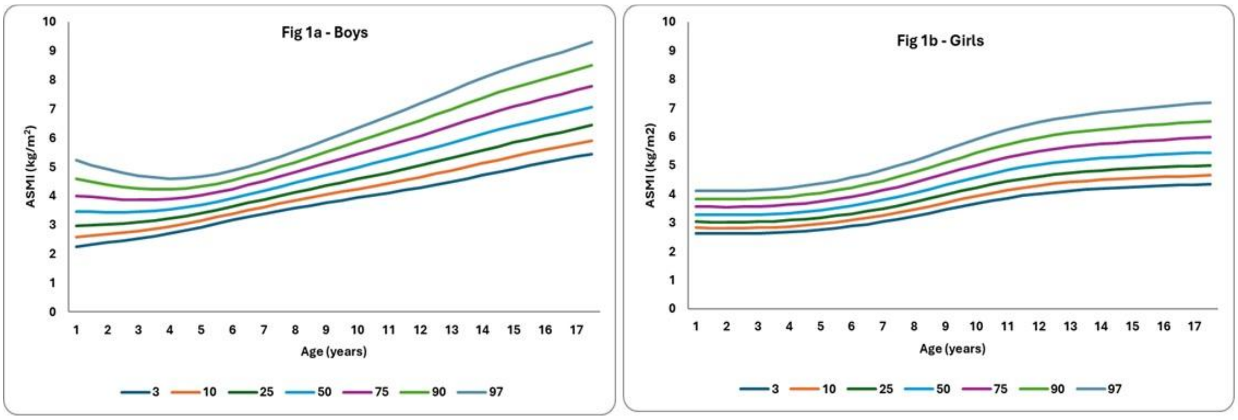

### Disclosure

None declared.

### P112

### Body Composition and Body Mineral Density Change Following Vertebral Fractures in Children with Leukemia

Moonbae Ahn[1,2], Maya Scharke[2], Nathalie Alos[3], Anne Marie Sbrocchi[4], Khaldoun Koujok[5], Jacob Jaremko[6], Mary-Ann Matzinger[5], Nazih Shenouda[5], Nagwa Wilson[5], Rui Santos[7], Ramy El-Jalbout[8], Ronald Barr[9], Frank Rauch[4], Kerry Siminoski[6], Stefan Jackowski[2], Jacqueline Halton[10], Jinhui Ma[11], Leanne Ward[12,2]

[1]Department of Pediatrics, The Catholic University of Korea, Seoul, Korea, Republic of. [2]The Ottawa Pediatric Bone Health Research Group, Children's Hospital of Eastern Ontario Research Institute, Ottawa, Canada. [3]Department of Pediatrics, Hôpital Sainte-Justine, Montreal, Canada. [4]Department of Pediatrics, Faculty of Medicine and Health Sciences, McGill University, Montreal, Canada. [5]Department of Radiology, Children's Hospital of Eastern Ontario, Faculty of Medicine, University of Ottawa, Ottawa, Canada. [6]Department of Radiology and Diagnostic Imaging, University of Alberta, Edmonton, Canada. [7]Evelina London Children's Hospital, Guy's and St Thomas' NHS Foundation Trust, London, UK. [8]Department of Radiology, Sainte-Justine Hospital and Research Center, University of Montrea, Montreal, Canada. [9]Department of Pediatrics, McMaster University, Hamilton, Canada. [10]Department of Pediatrics, Children's Hospital of Eastern Ontario, Faculty of Medicine, Ottawa, Canada. [11]Department of Health Research Methods, Evidence, and Impact, McMaster University, Hamilton, Canada. [12]Department of Pediatrics, Faculty of Medicine, Division of Endocrinology and Metabolism, University of Ottawa, Children's Hospital of Eastern Ontario, Ottawa, Canada

### Abstract

### Objective:

Children with acute lymphoblastic leukemia (ALL) experience skeletal morbidity; however, the long-term associations among body composition, fractures, and skeletal recovery remain unclear. To determine whether longitudinal changes in lean mass index (LMI) and fat mass index (FMI) *z*-scores are associated with fracture incidences and bone mineral density (BMD) *z*-scores changes in pediatric ALL.

### Methods:

Prospective, observational cohort study of 134 children with ALL enrolled in the STeroid-Associated Osteoporosis in the Pediatric Population study followed for 6 years post-diagnosis with BMD, body composition, and spine x-ray evaluations. Associations among longitudinal LMI and FMI *z*-score changes, vertebral and non-vertebral fractures (VF), and BMD *z*-score trajectories were examined using random-effects regression models (with appropriate corrections for height *z*-scores).

### Results:

The annual VF and non-VF incidences over 6 years were highest at 12 months (15.7% and 4.5%, respectively), at which time hip and total body less head (TBLH) BMD *z*-scores reached their nadirs (mean ± SD −1.9 ± 1.3 and −1.1 ± 1.7, respectively). LMI and FMI *z*-scores over 6 years were not associated with incident VF or non-VF. However, higher LMI *z*-scores over 6 years were associated with greater BMD *z*-scores at the LS (β = 0.09, $P$ = .014), hip (β = 0.19, $P$ < .001) and TBLH (β = 0.36, $P$ < .001) over the same period. In contrast, FMI *z*-scores showed site-specific associations with BMD *z*-scores, with a positive association at the LS (β = 0.08, $P$ = .002), negative association at the TBLH (β = −0.05, $P$ < .001), and no association at the hip. In addition, LMI *z*-score increases after the BMD nadir at 12 months were associated with greater recovery in TBLH BMD *z*-scores (β = 0.29, $P$ < .001), whereas FMI *z*-score increases after 12 months were not associated with post-12-month increases in BMD *z*-scores at any skeletal site.

### Conclusion:

LMI *z*-scores were associated with greater recovery in TBLH BMD *z*-scores after 12 months of leukemia therapy in children. Whether post-diagnosis lean mass recovery is associated with lower fracture rates later in life remains to be determined.

**Table 8. Association between body composition and change in bone mineral density *z*-scores beyond 12 months using random-effects linear regression**

| | [ab]Change in BMD *z*-score | | | | | |
|---|---|---|---|---|---|---|
| | LS | | Hip | | TBLH | |
| | β (95% CI) | *P*-value | β (95% CI) | *P*-value | β (95% CI) | *P*-value |
| [bc]LMI *z*-score at 12 months | 0.00(–0.09-0.10) | *.938* | 0.09(–0.02-0.19) | *.126* | –0.08(–0.22-0.05) | *.236* |
| [bc]LMI *z*-score change after 12 months | 0.06(–0.05-0.16) | *.290* | –0.07(–0.19-0.06) | *.288* | 0.29(0.19-0.39) | *<.001* |
| [bc]FMI *z*-score at 12 months | –0.01(–0.13-0.02) | *.160* | 0.01(–0.08-0.10) | *.816* | 0.03(–0.09-0.15) | *.047* |
| [bc]FMI *z*-score change after 12 months | 0.05(–0.02-0.12) | *.174* | –0.01(–0.09-0.08) | *.909* | –0.04(–0.11-0.04) | *.335* |

Abbreviations: BMD, bone mineral density; CI, confidence interval; FMI, fat mass index; LMI, lean mass index; LS, lumbar spine; TBLH, total body less head.

Each predictor was analyzed in a separate random-effects linear regression model adjusted for age, sex, average daily glucocorticoid dose, and BMD *z*-score at 12 months.

[a]All BMD *z*-scores are height-adjusted. Change in BMD *z*-scores was calculated at each follow-up time point relative to the 12-month assessment.

[b]All variables in the models were treated as time-varying covariates based on repeated measurements throughout the 6-year follow-up (baseline to 72 months), except where otherwise indicated.

[c]LMI and FMI *z*-scores were calculated only for children aged 5 years and older, as normative reference data are unavailable for younger children.

### Disclosure

None declared

### P113

### Muscle Function in Children with Achondroplasia – A Cross-Sectional Retrospective Study

Amish Chinoy, Lauren Rayner, Nicola Panchbhaya, Raja Padidela

Royal Manchester Children's Hospital, Manchester, UK

### Abstract

### Objectives

Achondroplasia is due to a gain-of-function mutation in *FGFR3*, resulting in growth impairment. Although limited data exists regarding reduced muscle strength, muscle function has not been explored in detail. Within our specialist achondroplasia multiprofessional multidisciplinary clinics, clinical assessment of muscle function is routine practice using jumping mechanography (peak jumping power, jumping efficiency, peak hopping force and grip force). This allowed us to retrospectively analyse muscle function parameters of children with achondroplasia, compared to normative data.

### Methods

Children attending for annual achondroplasia clinic visits (Dec 2024-Sept 2025) had muscle function routinely measured as part of physiotherapy assessments. Leonardo mechanography was utilised, with patients undertaking single 2-legged jump (to calculate peak jumping power and jumping efficiency), multiple 1-legged hop (to calculate peak hopping force) and grip force. Z-scores were calculated by Leonardo software, accounting for body mass, age and sex. Mean z-scores were compared against the normative reference mean (z=0) using two-sided one-sample t-tests. Associations between mechanography outcomes were assessed using Pearson's correlation coefficient (r). Significance threshold was p<0.05. Sensitivity analyses used height-adjusted z-scores.

### Results

15 children (8 female, mean age 10.5 years) demonstrated significantly reduced z-scores [95%CI] for jumping power (-2.99 [-3.63, -2.34], p<0.001), jumping efficiency (-2.61 [-3.27, -1.95], p<0.001), hopping force (n=9, -2.33 [-3.49, -1.18], p=0.002) and grip force (n=7, -3.33 [-3.71, -2.96], p<0.001) (Figure 1). This was across age ranges and sexes, and was essentially unchanged when corrected for height (mean adjusted z-score [95%CI]: jumping power -2.99 [-3.62, -2.35], p<0.001; jumping efficiency -2.61 [-3.26, -1.96], p<0.001; hopping force -2.53 [-3.92, -1.15], p=0.003; grip force -3.29 [-3.74, -2.83], p<0.001). Z-scores of jumping power correlated strongly with jumping efficiency (r=0.77, p<0.001) and hopping force (r=0.75, p=0.02).

### Conclusion

This data demonstrates that children with achondroplasia have significantly reduced dynamic muscle function globally, independent of sex, age and short stature. This would suggest an intrinsic feature of achondroplasia. Further studies would be needed to identify the impact this has on daily functioning and physical activities. Clinical evaluation of objective dynamic muscle function may help identify those with greater deficits, to direct resources accordingly and assess response to targeted new therapies.

**<u>Figure 1</u>**. Mean (95% CI) z-scores for muscle function measures in children with achondroplasia, relative to population reference mean of 0.

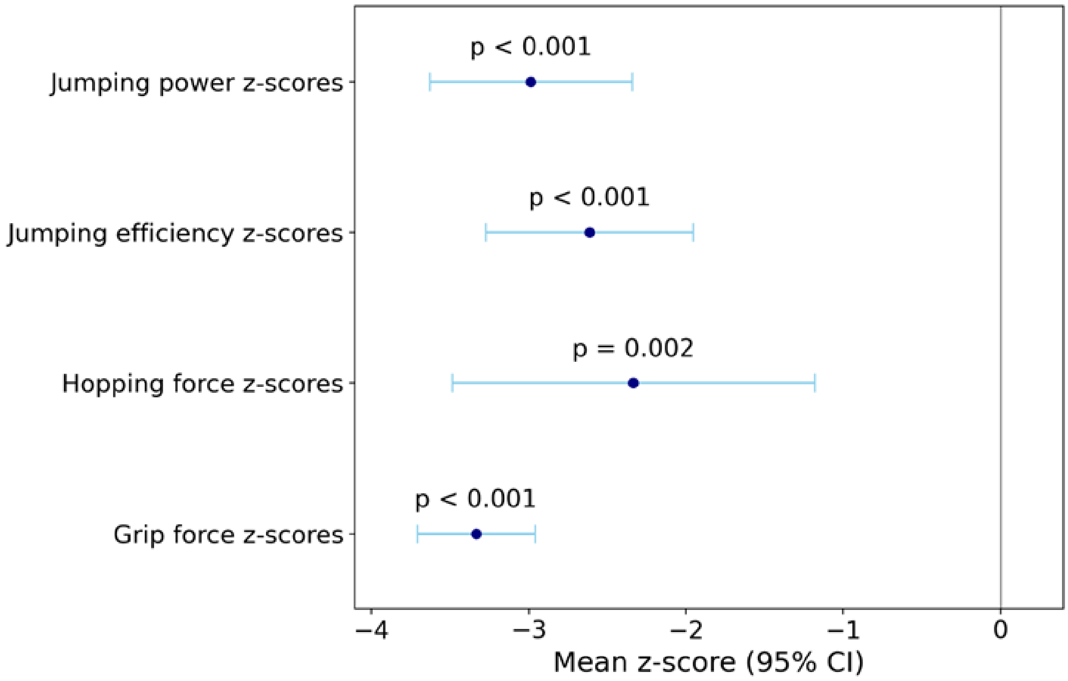

### Disclosure

None declared

### P114

### Prevalence and Determinants of a Sarcopenia-Like Phenotype in Indian Children and Adolescents with Type 1 Diabetes

Rubina Mandlik, Sharvani Patil, Vaman Khadilkar, Anuradha Khadilkar

Hirabai Cowasji Jehangir Medical Research Institute, Pune, India

### Abstract

**Background:**
Type 1 diabetes (T1D) is associated with accelerated muscle ageing and with muscle dysfunction linked to insulin deficiency and glycaemic control. Sarcopenia is increasingly recognised in paediatric populations and has important implications for skeletal development and musculoskeletal health. Given the critical role of muscle in bone health and the paucity of data from India, this study aimed to estimate the prevalence of a sarcopenia-like phenotype and identify its determinants among Indian children and adolescents with T1D.

**Methods:**
A cross-sectional, observational study was conducted among 210 Indian children and adolescents with T1D (85 boys,10–18years). Anthropometry (height and weight), body composition (using dual-energy X-ray absorptiometry, Lunar iDXA; GE Healthcare, Encore v18) and handgrip strength (Jamar hand dynamometer) were assessed. A sarcopenia-like phenotype was defined as grip strength and appendicular skeletal muscle index below the 10th percentile relative to a reference population. Glycaemic control was assessed using glycosylated haemoglobin (HbA1c) measured by high-performance liquid chromatography, and haemoglobin concentration was measured using automated spectrophotometry.

**Results:**
One in five participants with T1D exhibited a sarcopenia-like phenotype. Longer disease duration (OR = 1.28; 95% CI: 1.06–1.36; $p = 0.004$) and poor glycaemic control (OR = 1.26; 95% CI: 1.02–1.56; $p = 0.03$) were independently associated with higher odds of a sarcopenia-like phenotype. In contrast, higher body mass index (OR = 0.41; 95% CI: 0.28–0.59; $p < 0.01$) and haemoglobin levels (OR = 0.65; 95% CI: 0.46–0.91; $p = 0.01$) were associated with lower odds. The final multivariable model demonstrated good overall performance ($\chi^2 = 77.3$; $p < 0.05$), explaining 52% of the variance (Nagelkerke $R^2 = 0.52$) and correctly classifying 86% of participants.

**Conclusion:**
A significant proportion of the children and adolescents with T1D exhibit a sarcopenia-like phenotype, with risk increasing with poor glycaemic control and longer disease duration. These findings underscore the need for integrated diabetes management strategies that prioritise optimal glycaemic control alongside muscle-strengthening interventions initiated early in the disease course to mitigate progressive muscle deterioration and support bone health while preserving muscle mass, strength, and function across childhood, adolescence, and into adulthood.

### Disclosure

None declared

### P115

### Greater Embryonic Movement Leads to Enhanced Postnatal Bone Mass in Chickens

Setareh Khorshidparast[1,2], Renee Garant[3], Anthony Helou[1], Tamara Droual[1], Karim Elsayed[1], Maya Arab Makansi[1], Tina Widowski[3], Alexandra Harlander[3], Bret Tobalske[4], Bettina M. Willie[1,2,5]

[1]Shriners Hospitals For Children - Canada, Montreal, Canada. [2]McGill University, Montreal, Canada. [3]University of Guelph, Guelph, Canada. [4]University of Montana, Missoula, USA. [5]Utah State University, Utah, USA

### Abstract

**Objectives:**

Fetal movement generates mechanical stresses essential for skeletal development and bone strength. While human clinical data and paralysis models (chick/mouse) demonstrate that restricted or absent movement impairs joint and bone structure, the specific effects of more or less embryonic movement on bone structure and strength have not been examined. This study aims for the first time, to quantify embryonic movements *in ovo* and compare bone outcomes after hatch. We evaluated bone mineral density, microarchitecture, and strength, as well as biochemical markers of bone turnover and muscle mass.

**Methods:**

A total of 73 fertile eggs from white and brown-feathered hens were incubated under commercial conditions at the University of Guelph. Embryonic movement was recorded via digital monitor (Egg Buddy, Avitronics) on days 15 and 18. After hatch (day 21), chicks were euthanized to assess bone outcomes through micro-CT (left tibiotarsus), three-point bending (left femur), serum P1NP (ELISA), and leg muscle weights. Multivariable regression and ANOVA analysis evaluated the influence of strain, sex, and embryonic movement changes between days 15 and 18 on the recorded skeletal and muscular parameters.

**Results:**

Sex, genetic strain, and embryonic movement (Fig. 1A) were significant predictors of metaphyseal trabecular bone volume fraction (Tb.BV/TV) ($p < 0.05$). Higher embryonic movement, brown-feathered strain, and female sex were each associated with increased Tb.BV/TV. Genetic strain was also a significant predictor of diaphyseal total area (Tt.Ar), with brown-feathered chicks exhibiting greater Tt.Ar (Fig. 1B). Embryonic movement demonstrated a positive association with Tt.Ar as well ($p = 0.095$). Additionally, brown chicks exhibited a significantly higher maximum load (17%; $p < 0.001$) compared to white chicks. ANOVA results did not show a significant difference in P1NP levels and leg muscle in different sexes, genetic strains, or movement status.

**Conclusions:**

Our results indicate embryonic movement, sex, and genetic strain modulate trabecular and cortical bone mass, while genetic strain influences the bone strength. These data provide evidence that embryonic movement leads to improved bone mass and microstructure.

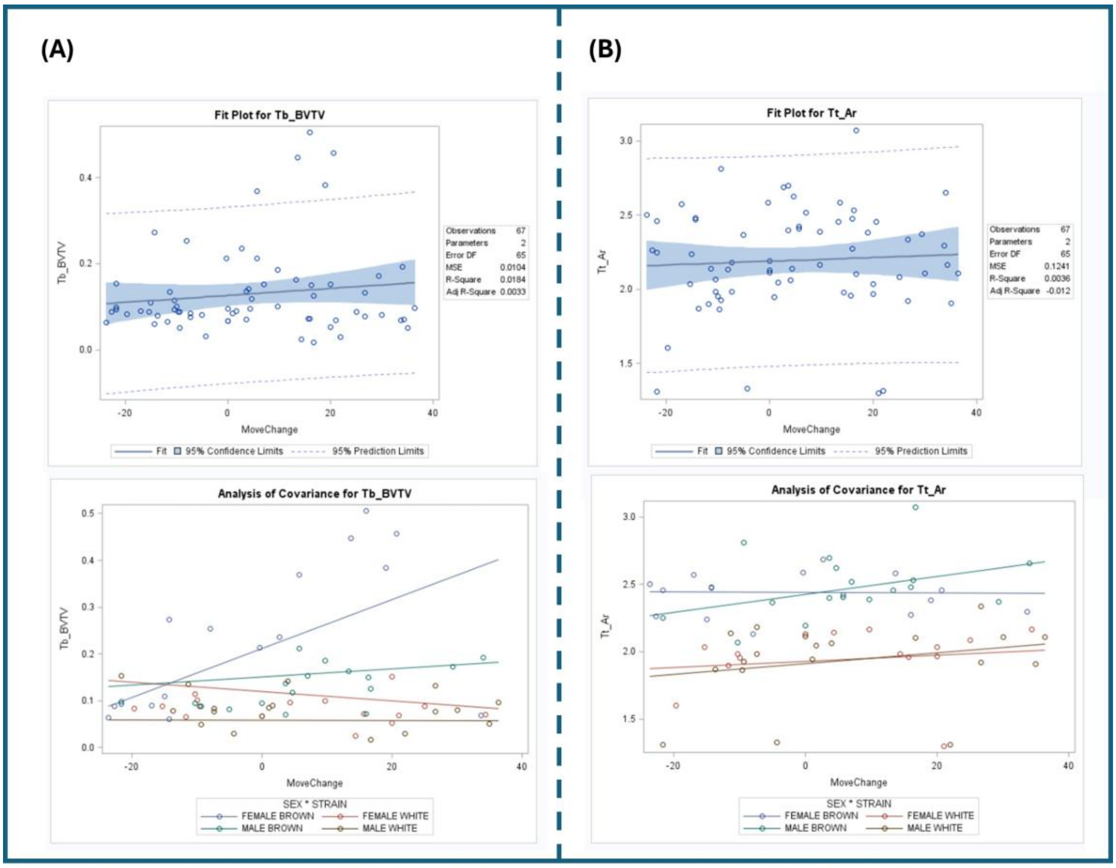

**Figure 1**: Univariable (top row) and ANCOVA (bottom row) regression plots for trabecular bone volume fraction (Tb_BV/TV) (A) and total area (Tt_Ar) (B); sex, strain, movement change (MoveChange = EM18 – EM15) are significant predictors of the Tb_BV/TV. In addition, movement change is a positive indicator of Tt_Ar.

### Disclosure

None declared

### P116

### Using Lower Body Power to Predict Upper Body Bone Strength in Thalassemia Patients

Emily Van Horn[1], Vanessa Yingling[2], Ellen Fung[3], Jennifer Sherwood[1]

[1]Cal State East Bay, Hayward, USA. [2]Occidental University, Burbank, USA. [3]UCSF, Oakland, USA

### Abstract

Thalassemia is a genetic disorder that impairs hemoglobin production, causing fatigue, reduced physical activity, and decreased bone mass. Peak power predicted from a vertical jump (PPVJ) has been shown to be associated with tibial bone strength, while emerging evidence suggests that peak power estimated from the sit-to-stand (STSp), a commonly used clinical measure, may be associated with radial bone strength. OBJECTIVE: To compare STSp and PPVJ as predictors of radial and tibial bone strength in individuals with thalassemia.

METHODS:

Peak power was assessed in individuals aged 14-40 years using body mass and linear velocity measured with a linear position transducer during the STS test (n=11), and estimated from countermovement vertical jump height using the Sayers equation (n=16). Bone strength was assessed using peripheral quantitative computed tomography (pQCT) via moment of inertia (MoI), cortical area (Ct.Ar.), strain index (SSI) and cortical bone mineral density (cBMD) at the 33% radial, and 38% tibial sites. Muscle-bone relationships were evaluated using linear regressions.

RESULTS:

Pearson correlations demonstrated strong positive associations between STSp and tibial measures ($p<0.02$, $r = 0.85-0.88$), and between PPVJ and both tibial ($p<0.01$, $r = 0.61-0.71$) and radial site measures ($p<0.36$, $r = 0.45-0.67$). In contrast, neither STSp nor PPVJ was significantly associated with tibial or radial cBMD.

CONCLUSION:

Preliminary data suggests that PPVJ is associated with both radial and tibial bone strength, whereas STSp was associated with tibial, but not radial, bone strength. Collectively, these findings suggest that both measures relate to architectural and geometric properties of bone rather than bone density.

### Disclosure

None declared

### P117

### Bone Health in Children and Youth with Cerebral Palsy and Duchenne Muscular Dystrophy: A Retrospective Comparison from a Tertiary Centre in India

Anuradha Khadilkar[1], Chirantap Oza[2], Prashant Patil[3], Aniata Hegde[4], Suhani Shah[4], Amulya A D[5]

[1] Hirabai Cowasji Jehangir Medical Research Institute, Jehangir Hospital, Pune, India. [2]Consultant Pediatric Endocrinologist, EndoGrow, Ahmedabad, India. [3]SRCC Narayana Children's Hospital and Apollo hospital, Navi Mumbai, India. [4]SRCC Narayana Children's Hospital and Jaslok Hospital & Research centre, Mumbai, India. [5]SRCC Narayana Children's Hospital, Mumbai, India

### Abstract

### Introduction:

Cerebral palsy(CP) and Duchenne muscular dystrophy(DMD) are common neuromuscular disorders associated with chronic muscle weakness and increased risk of secondary osteoporosis. Awareness and evaluation of bone health remain limited in low and middle income countries like India. Therefore, our objective was to describe and compare bone health (DXA-derived) in children and youth(CY) with CP and DMD, and examine clinical factors associated with low bone density.

### Method:

Design:Retrospective, observational, single-centre study including data on 44 CY, 6–25years with CP/DMD, referred for bone health assessment (January 2022-December 2025). History, anthropometry, and dual-energy X-ray absorptiometry (DXA, Lunar Prodigy)–derived measures were obtained using standard protocols. Participants with a recent fracture, orthopaedic surgery or those with implants were excluded.

### Results:

Data on 22 subjects each with DMD/CP, mean age 2.4±4.3years were analysed (n=6 were girls with CP). Only 1 child (DMD) had a documented significant fracture while 3 were on bisphosphonates (2 with DMD, 1 with CP). Mean total body less head (TBLH) and lumbar spine (LS) bone mineral density (BMD) Z-score was -2.3. Poor LS BMD was noted in significantly higher number of subjects with CP while proportion of poor TBLH BMD was higher in children with DMD (figure 1). Vertebral fracture was noted on VFA assessment in 2 children (1 DMD and 1 CP) with BMD Z-score more than -2.0. Weight, body mass index (BMI) and lumbar spine bone mineral apparent density (LSBMAD) were significantly lower in the CP group. LSBMAD was significantly lower in subjects with DMD on steroids than not on steroids (0.11±0.3 vs 0.14±0.02). Both height and weight Z score had significant positive correlation with TBLH and LS BMD but only weight Z score correlated with BMAD.

### Conclusion:

CY with CP and DMD show a high burden of low bone mineral density, with differing skeletal involvement between the two conditions. Vertebral fractures were identified in children with BMD Z-scores>−2.0, suggesting that fracture risk may be underestimated when relying on BMD or fracture history alone. Our findings underscore the need to improve recognition of secondary osteoporosis and the selective use of vertebral fracture assessment in low-resource settings.

### Disclosure

None declared

### P118

### Trabecular Bone Score (TBS) in Females with Anorexia Nervosa: TBS Osteo Advanced for Pediatrics (Version 4)

Anna Walstra[1], Julie Briody[2], Kevin London[2], Madeleine Davies[1], Didier Hans[1,3], Karen Hind[1,4], Craig Munns[5]

[1]Medimaps Group SA, Geneva, Switzerland. [2]The Children's Hospital at Westmead, Sydney, Australia. [3]Center of Bone Diseases, Rheumatology Unit, Bone and Joint Department, Lausanne University Hospital & University of Lausanne, Lausanne, Switzerland. [4]Faculty of Health and Medicine, Lancaster University, Lancaster, UK. [5]The University of Queensland, Brisbane, Australia

### Abstract

OBJECTIVES*:*

Anorexia nervosa involves prolonged energy deficiency and reduced body mass, compromising skeletal development during critical growth phases, and increasing fracture susceptibility. While DXA-measured bone mineral density serves as the standard bone health indicator, it fails to capture microarchitectural properties. Trabecular bone score (TBS), a validated DXA-derived index, provides indirect evaluation of bone microstructure and nutrition-related skeletal deterioration. This study sought to assess TBS in pediatric females with anorexia nervosa and explore its relationship with disease severity, reflected by body mass index (BMI).

METHODS*:*

Female patients with anorexia nervosa (AN), referred for clinically indicated DXA at a tertiary children's hospital were included. Lumbar spine DXA scans were analysed using TBS Osteo Advanced for Pediatrics (Version 4). Patients outside the validated age range (5-20 years) or with abdominal soft tissue thickness outside the manufacturer-recommended limits (7.0-30.0 cm) were excluded. TBS Z-scores were derived using an age-matched reference cohort. Statistical evaluation determined whether AN participants exhibited lower TBS relative to the reference group, and compared TBS Z-scores among BMI classifications (underweight <18.5, normal weight 18.5–24.9, overweight ≥25 kg/m²).

RESULTS*:*

The cohort comprised 2,774 females (mean age 15.43 ± 1.77 years; 86.6% White; mean BMI 17.80 ± 2.30 kg/m²). Mean TBS Z-scores were significantly lower compared with age-equivalent reference data (−0.36 ± 1.00; $p < 0.001$). The majority of AN participants were underweight (64.2%), with 35.2% classified as normal weight and minimal representation in the overweight category. Underweight individuals showed substantially lower TBS Z-scores versus normal-weight group (mean difference −0.35; $p < 0.001$). Results suggest a graded association between declining BMI and impaired trabecular bone microarchitecture.

CONCLUSIONS:

This extensive AN cohort exhibited significantly lower trabecular bone scores compared with age-matched normative data, reflecting compromised trabecular microarchitecture. Reduced TBS Z-scores among underweight participants underscores the detrimental skeletal consequences of low body weight during critical periods of bone development. These findings confirm the use of TBS as a complementary DXA-derived measure to enhance the assessment of skeletal impairment and disease sequalae in young patients with anorexia nervosa.

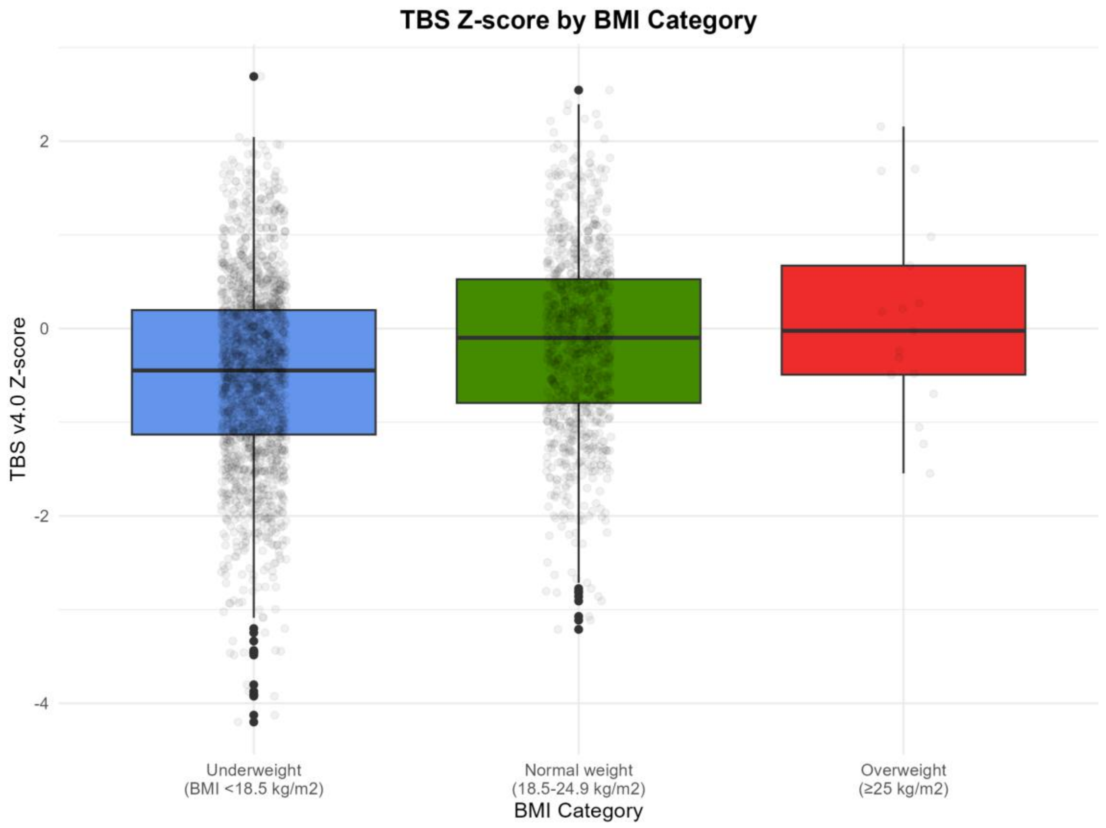

***Figure 1.*** *Trabecular bone score (TBS) by BMI category for pediatric females with anorexia nervosa*

### Disclosure

JB, KL and CM declare they have no conflicts of interest. AW, MD and KH are employees of Medimaps Group SA, developers of TBS iNsight™ software. DH is co-owner of the TBS patent, has corresponding shares, and is part-time CEO at Medimaps Group.

### P119

### Three Diet Quality Indices (NRF 4.3.3, NRF 9.3, HEFI-2019) for Predicting Bone Health in Canadian Children Aged 5 to 11 Years

Karim Karbin[1], Hassan Vatanparast[1], Ginny Lane[2], Mojtaba Shafiee[2], Zoe Longworth[1], Adam Baxter-Jones[1], Marta Erlandson[1]

[1]University of Saskatchewan, Saskatoon, Canada. [2]University of Idaho, Moscow, USA

### Abstract

**OBJECTIVES:**

Adequate intake of nutrient-dense foods supports growth and skeletal development during childhood. The objective of this study was to compare the predictive validity of three diet quality indices, the Nutrient Rich Food Index 9.3 (NRF9.3), Nutrient Rich Food Index 4.3.3 (NRF4.3.3), and Healthy Eating Food Index-2019 (HEFI-2019), in relation to whole-body and site-specific bone mineral content (BMC), bone mineral density (BMD), and lumbar spine bone mineral apparent density (BMAD) in Canadian children aged 5 to11 years, to determine which diet quality indices predict pediatric skeletal health.

**METHODS:**

Baseline data from 112 children participating in the Nutrition and Growth Study were analyzed. Dietary intake was assessed using three 24-hour recalls spaced at least 10 days apart. Diet quality was evaluated with the NRF9.3, NRF4.3.3, and HEFI-2019 indices. Bone health outcomes, including whole-body and site-specific BMC, BMD, and BMAD, were measured using dual-energy X-ray absorptiometry (DXA). Multivariable linear regression models were used to evaluate whether diet quality predicts bone outcomes, adjusting for total energy intake, age, sex, height, physical activity, and skin colour.

**RESULTS:**

Higher NRF9.3 scores were positively associated with total BMC ($\beta = 0.37$, SE = 0.15, $p = 0.014$) and BMD ($\beta = 0.0002$, SE = 0.00007, $p = 0.005$) after multivariable adjustment. NRF4.3.3 or HEFI-2019 scores were not significant predictors of bone health outcomes (all $p > 0.05$). Biological maturation was evaluated as a potential confounder and was not found to significantly influence the outcomes.

**CONCLUSIONS:**

NRF9.3 was a significant diet quality predictor of total BMC and BMD in Canadian children aged 5 to 11 years. The lack of significant findings for NRF4.3.3 and HEFI-2019 suggests that neither hybrid nutrient density scoring nor dietary guideline-based adherence indices adequately capture the dietary factors influencing bone accrual during middle childhood. NRF9.3 appears to be a superior predictor because it specifically assigns greater weight to micronutrients essential for bone development, including calcium, vitamin D, protein, and magnesium.

### Disclosure

None declared

### P120

### Self-Reported Gastrointestinal Symptoms in Children and Adults with a Skeletal Dysplasia

Laurel Baxter, Caitlin Brown, Erin Carter, Deborah McInerney, Cathleen Raggio

Kathryn O. and Alan C. Greenberg Center for Skeletal Dysplasias, Hospital for Special Surgery, New York, USA

### Abstract

**Objectives:**

This study aims to better understand gastrointestinal (GI) symptoms in the skeletal dysplasia population, focusing on osteogenesis imperfecta (OI). Though limited, existing literature emphasizes under-reporting, under-diagnosis, and under-treatment of skeletal dysplasia-related GI issues. We hypothesize that the adult and pediatric skeletal dysplasia populations experience increased GI symptoms compared to the general population, and that symptoms correlate with lifestyle factors such as medication usage and ambulatory status.

**Methods:**

We performed a retrospective review of standard-of-care GI symptom surveys and patient charts at the Kathryn O. and Alan C. Greenberg Center for Skeletal Dysplasias at the Hospital for Special Surgery. Adults (>18 years old) completed the Gastrointestinal Symptom Rating Scale (GSRS), which assesses severity of 15 GI symptoms across 5 domains: reflux, abdominal pain, indigestion, diarrhea, and constipation. Pediatric patients and/or caregivers completed the Questionnaire on Pediatric Gastrointestinal Symptoms, Rome III Version (QPGS-RIII), screening for 10 functional GI disorders. Demographic and lifestyle data was collected from charts and a supplementary survey.

**Results:**

Fifty-one adults with skeletal dysplasias were enrolled. Compared to GSRS reference data, the group scored higher on reflux and indigestion, and lower on abdominal pain, diarrhea, and constipation; higher scores indicated greater symptom severity. Twenty-one participants had an OI diagnosis and 30 had other skeletal dysplasias, including achondroplasia (n=10), diastrophic dysplasia (n=3), and pseudoachondroplasia (n=3). The OI group scored higher on reflux and lower on constipation compared to reference and non-OI dysplasia groups. Females scored higher than males across all domains, driven by non-OI females. In the non-OI dysplasia group symptoms decreased with age, whereas symptoms increased with age in the OI group. Individuals using power wheelchairs reported more severe symptoms than those using manual wheelchairs, or walking independently or with assistive devices. Higher usage of medications with known GI-effects was positively correlated with symptom severity.

**Conclusions:**

These findings demonstrate the clinical relevance of GI symptoms for the skeletal dysplasia population. Further research is needed to better understand the roles of sex, age, dysplasia type, ambulatory status, and medications, to help providers offer individualized support managing GI symptoms.

### Disclosure

None declared

### P121

### Self-Reported Gastrointestinal Symptoms and Support in the Osteogenesis Imperfecta Community

Laurel Baxter[1], Caitlin Brown[1], Erin Carter[1], Michael Stewart[2], Erika Carter[2], Tracy Hart[2], Cathleen Raggio[1]

[1]Kathryn O. and Alan C. Greenberg Center for Skeletal Dysplasias, Hospital for Special Surgery, New York, USA. [2]Osteogenesis Imperfecta Foundation, Gaithersburg, USA

### Abstract

**Objectives:**

Gastrointestinal (GI) symptoms are a patient- and provider-reported concern amongst the Osteogenesis imperfecta (OI) community, yet data and resources are lacking. This project aims to better understand GI concerns amongst the OI community to inform future research questions and clinical practice.

**Methods:**

The Osteogenesis Imperfecta Foundation (OIF) administered a 44-question survey assessing GI symptoms and management. The survey was open worldwide to the entire OI community. Responses were self-reported and unable to be verified by medical providers, with the survey designed to capture broad conversations and concerns.

**Results:**

Two hundred ninety-nine responses were analyzed, including individuals under 18 years old (n=32), 18-30 (n=50), 31-50 (n=91), 51-70 (n=101), and over 70 (n=25). Respondents were from 23 countries, including the USA (n=192), the UK (n=31), Canada (n=18), and Germany (n=13). Fifty-two percent of participants reported GI symptoms they believed were related to OI, and 35% of participants reported GI symptoms but were unsure if they were OI-related. Only 13% of participants reported no GI symptoms. Constipation (65%), reflux (62%), gas (57%), and bloating (51%) were the most frequently reported GI symptoms. Thirty-three percent of respondents experienced GI symptoms daily and 34% experienced symptoms several times per week, indicating chronic GI issues. Reported symptoms were similar across ages, genders, and countries. Symptoms were reported at the following rates by OI type: type I (53%), type III (63%), and type IV (43%), with reflux and constipation most frequent in types I and III and reflux and gas most frequent in type IV. Symptoms increased with decreased ambulation, with the highest rates amongst individuals with electric wheelchairs (65% reported symptoms), scooters (60%), and manual wheelchairs (58%), compared to 46% of individuals with no mobility aids reporting symptoms. Forty percent of participants reported receiving enough support from healthcare providers in managing GI symptoms.

**Conclusions:**

The high frequency of symptoms, uncertainty about the connection between GI symptoms and OI, and the lack of perceived support in managing symptoms indicate unanswered questions in the OI community and the need for further research and resources for patients.

### Disclosure

None declared

### P122

### Posterior Spinal Fusion (PSF) Outcomes and Complications in Pediatric Neuromuscular Scoliosis (NMS) Patients Receiving Preoperative Bisphosphonates (BP)

Lauren Gadek[1], Ilona Lukina[2], Jamie Burgess[2], Suna Paek[2], Brett Lullo[2,1], Joseph Janicki[2,1], Jennifer Miller[2,1]

[1]Northwestern University Feinberg School of Medicine, Chicago, USA. [2]Ann & Robert H. Lurie Children's Hospital (LCH), Chicago, USA

### Abstract

**Objectives:**

The objective of this study was to compare perioperative characteristics and hardware related complication rates in pediatric NMS patients receiving BPs prior to PSF to national trends in NMS PSF patients not receiving preoperative BPs.

**Methods:**

Retrospective chart review of 19 patients <18 years treated with BPs prior to PSF at a tertiary care hospital was performed. Data collected was part of a multicenter BP utilization database including demographics, glucocorticoids, fractures, BPs, and adverse events to BPs. Surgical time, intraoperative complications, length of stay (LOS), emergency department (ED) visits, hospital readmission, reoperation, and other postoperative complications were included. Descriptive data analysis was completed.

**Results:**

Median age at surgery was 12.4 years. Etiologies of NMS included cerebral palsy, muscular dystrophy, myopathy, or spinal muscular atrophy. 89.5% were non-ambulatory. Median time from BP administration to PSF was 4.6 months. All BP adverse events were minor, including fever, electrolyte abnormalities, and post-infusion bone pain, managed with observation and/or supplementation. Eighteen patients had BMD data via Dual-energy X-ray Absorptiometry (DXA), all of which were low at all time points. Median aBMD Z-score = -3.42.

Perioperative characteristics of our sample, as well as the published literature, are summarized in Table 1. Within 90 days of surgery, 5 patients had ED visits, 2 were readmitted, and no reoperations were needed. All hardware-related complications were minor and none were attributed to low BMD. No patients had scoliosis progression after PSF.

**Conclusions:**

This study evaluated perioperative characteristics and complication rates in NMS patients with preoperative BP exposure prior to PSF. To our knowledge, this is the first case series describing perioperative characteristics of this population. While our results mirror published data in children undergoing PSF without preoperative BPs, further work with a larger sample size and prospective study design is needed to elucidate the effects of preoperative BPs on PSF outcomes.

Table 1. Summary of Perioperative Data at LCH (preoperative BPs) and Published Data (no BPs)

| Variable | LCH cohort (n= 19) | Published Data, range (n=16-15,218) |
|---|---|---|
| Median Duration of surgery (minutes) | 406 | 258-574.8 |
| Median Estimated Blood Loss (ml) | 800 | 612.1- 3003 |
| Median LOS (days) | 7 | 4.9-17 |
| Mean Preop curve (°) | 95.05 | 63.4-84.0 |
| Mean Postop curve (°) | 54.07 | 25-39.1 |
| Return to OR (%) | 0 | 0.66-14.9 |
| Hardware-related complications (%) | 38.9 (all minor) | 0.70-33 (any complication) |

References: Beckman et al. 2016, Fletcher et al. 2020, Gaume et al. 2023, Holt et al. 2017, Master et al. 2011, Modi et al. 2020, Rumalla et al. 2016, Sharma et al. 2013, Toll et al. 2018

### Disclosure

Yes. Only Dr. Jennifer Miller has disclosures. Alexion (Research Grant includes principal investigator, collaborator or consultant and pending grants as well as grants already received). Catalyst Pharmaceuticals (Consultant/Advisory Board). Element Bars, Inc (Stockholder/Ownership Interest (excluding diversified mutual funds)). Inozyme, Inc (now Biomarin) (Research Grant includes principal investigator, collaborator or consultant and pending grants as well as grants already received). Inozyme, Inc (now Biomarin) (Speaker/Honoraria includes speakers bureau, symposia, and expert witness). Ipsen (Consultant/Advisory Board)

### P123

### Which Anthropometric Measure Best Reflects Adiposity and Functional Impact in Achondroplasia?

Ruggero Lanzafame[1], Emma Bewick[2], Catherine Ali[3], Moira Cheung[3]

[1]University Hospital of Verona, Department of Surgical Science, Dentistry, Gynecology and Pediatrics, Verona, Italy. [2]Evelina London Children's Hospital, Paediatric Endocrine and Bone Service, London, UK. [3]Great Ormond Street Hospital, Paediatric Endocrinology Department, London, UK

### Abstract

### OBJECTIVES

Assessment of obesity in achondroplasia is challenging due to disproportionate short stature, and it remains unclear which anthropometric measure best reflects adiposity and its impact on physical function in this population. Using dual-energy X-ray absorptiometry (DXA)–derived fat mass as reference, we aimed to (1) compare the ability of commonly used anthropometric measures to reflect adiposity and (2) explore their associations with functional performance and obesity-related clinical outcomes.

### METHODS

In a cohort of children and adolescents with achondroplasia (n=24, age 9-18), anthropometric measures (weight, BMI, waist and hip circumference, waist-to-height ratio) were compared with DXA-derived fat mass. Associations with functional capacity (6-minute walk test [6MWT]), impact on everyday activities (Screening Tool for Everyday Mobility and Symptoms [STEMS score]), orthopaedic complications and sleep apnoea status were explored using regression models adjusted for age and sex. Each anthropometric marker was evaluated for its ability to reflect adiposity and predict clinical burden.

### RESULTS

All anthropometric measures were significantly associated with DXA-derived fat mass after adjustment for age and sex, confirming their validity as markers of overall adiposity in achondroplasia.

BMI and waist-to-height ratio were independently associated with reduced performance on the 6-minute walk test (6MWT), whereas weight, waist circumference, and hip circumference were not. This suggests that measures accounting for body proportionality may better reflect the mechanical impact of excess adiposity on mobility.

BMI was the only anthropometric measure independently associated with STEMS score, although the effect size was small. None of the anthropometric measures were independently associated with orthopaedic complications or sleep apnoea after age- and sex-adjustment.

| | **DXA total fat mass (β, p)** | **6MWT Z-score (β, p)** | **STEMS score (β, p)** | **Orthopaedic complications (β, p)** | **Sleep apnoea (β, p)** |
|---|---|---|---|---|---|
| **Weight (kg)** | ✅ (0.70, <0.05) | ❌ (−2.44, 0.22) | ❌ (-0.0, 0.59) | ❌ (-0.02, 0.86) | ❌ (−0.09, 0.35) |
| **BMI (kg/m²)** | ✅ (1.28, <0.05) | ✅ (-7.45, <0.05) | ✅ (1.02, <0.05) | ❌ (-0.03, 0.89) | ❌ (-0.18, 0.32) |
| **Waist circumference** | ✅ (0.69, <0.05) | ❌ (−3.29, 0.10) | ❌ (−0.0, 0.58) | ❌ (0.08, 0.46) | ❌ (−0.14, 0.19) |

| | | | | | |
|---|---|---|---|---|---|
| **(cm)** | | | | | |
| **Hip circumference (cm)** | ✅ (0.53, <0.05) | ❌ (−1.18, 0.53) | ❌ (0.0, 0.29) | ❌ (0.06, 0.52) | ❌ (−0.06, 0.46) |
| **Waist/height (absolute)** | ✅ (82.55, <0.05) | ✅ (-610.5, <0.05) | ❌ (-0.0, 0.78) | ❌ (12.05, 0.37) | ❌ (−18.24, 0.19) |

*Table 1.* Comparative performance of anthropometric measures (age- and sex-adjusted).

**CONCLUSIONS**

In achondroplasia, no single anthropometric measure fully captures obesity-related disease burden; however, while all measures reflect DXA-derived fat mass, **BMI** and **waist-to-height ratio** emerge as the most informative indicators of functional impairment, particularly reduced mobility. The limited associations with orthopaedic complications and sleep apnoea highlight that these outcomes are likely influenced by factors beyond adiposity alone.

These findings support the use of simple anthropometric measures accounting for body proportionality to assess obesity-related functional burden in achondroplasia, while underscoring the need for larger studies to refine risk stratification and outcome prediction in this population.

### Disclosure

Non declared

### P124

### Surgical Outcomes and Complications of Joint Replacement in Osteogenesis Imperfecta

Caitlin Brown[1], Chloe Derocher[1], Jack Mulcrone[1], Erin Carter[1], Mathias Bostrom[2], Mark Figgie[2], Cathleen Raggio[3]

[1]Kathryn O. and Alan C. Greenberg Center for Skeletal Dysplasias, Hospital for Special Surgery, New York, USA. [2]Department of Orthopedic Surgery, Hospital for Special Sugery, New York, USA. [3]Kathryn O. and Alan C. Greenberg Center for Skeletal Dysplasias, Department of Orthopedics, Hospital for Special Surgery, New York, USA

### Abstract

**Objectives:** Osteogenesis imperfecta (OI) is a genetically diverse disorder of type I collagen. Individuals with OI frequently experience bone fragility, deformities, recurrent fractures, and joint laxity, which may predispose them to early degenerative joint disease requiring surgical management. Existing reports on joint arthroplasty in OI remain sparse and are generally limited by small cohorts. To address this gap, we conducted a retrospective assessment of joint replacement outcomes in individuals with OI treated at the Hospital for Special Surgery (HSS).

**Methods:** As part of an IRB-approved retrospective study of patients with skeletal dysplasias undergoing arthroplasty at HSS from 2011–2025, we identified 15 individuals with OI (12 female, 3 male), all with mild-to-moderate disease, including 9 with type I and 6 with type IV OI. Clinical records were reviewed for surgical and medical history, radiographic and advanced imaging findings, DEXA results, SF-36 scores, indications for arthroplasty, pre- and post-operative pain and function, range of motion, and intra- and post-operative complications.

**Results:** Across these 15 individuals, 25 arthroplasty procedures were performed, including 16 hip, 6 knee, and 3 shoulder replacements. Mean age at primary joint replacement was 51.7±17.6 years (range 25–73). Average height was 143.2±37.2 cm, weight 61.3±22.0 kg, and BMI 29.9±8.8. Twelve individuals presented with acetabular protrusio. SF-36 scores were generally lower than population norms, except in the emotional well-being category. Twelve procedures (48%) were performed in patients with pre-existing cardiovascular or pulmonary conditions, and seven procedures (28%) required custom implants. All patients reported reduced pain and improved mobility post-operatively. Three procedures (12%) were revisions due to osteolysis with polyethylene wear, recurrent hip dislocation, or acute subscapularis tear. One intra-operative subtrochanteric fracture occurred. Post-operatively, there was one orthopedic complication (periprosthetic fracture requiring ORIF) and one neurological complication (femoral neuropraxia). Six procedures (24%) were associated with medical complications, including active incisional drainage, urinary retention requiring catheterization, dizziness or nausea, persistent heartburn, and adhesive-related erythema with blistering.

**Conclusions:** By characterizing complications, associated risk factors, and clinical outcomes, this study informs pre-operative evaluation and strategic planning for joint arthroplasty in individuals with OI.

### Disclosure

None declared

### P125

### Surgical Spine Procedures in Patients with Osteochondrodysplasias: A Review of the Literature and the Experience at One Institution

Caitlin Brown[1], Erin Carter[1], James Farmer[2], Cathleen Raggio[1]

[1]Kathryn O. and Alan C. Greenberg Center for Skeletal Dysplasias, Hospital for Special Surgery, New York, USA. [2]Department of Orthopedics, Hospital for Special Surgery, New York, USA

### Abstract

**Objectives:**

Osteochondrodysplasias encompass over 400 genetically distinct skeletal disorders, many involving complex spinal pathology, including congenital stenosis, instability, and multilevel deformity. These features, combined with abnormal connective tissue and high rates of medical comorbidity, contribute to elevated surgical risk. Literature is limited to small, heterogeneous cohorts, with few reports describing contemporary complication and revision rates. Building on a previously published series of 25 patients, this study evaluates an expanded cohort of 56 individuals with skeletal dysplasias who underwent spinal surgery over 18 years.

**Methods:**

We conducted a retrospective review of pediatric and adult patients with confirmed osteochondrodysplasias who underwent spinal surgery at the Hospital for Special Surgery (HSS) between 2007 and 2025. Ten patients had prior spine procedures at outside institutions before their index surgery at HSS. Demographics, diagnoses, surgical characteristics, and postoperative outcomes, including complications and subsequent spine procedures, were collected.

**Results:**

The cohort included 56 patients (mean age 45.4 ± 15.2 years; mean BMI 33.6 ± 9.0 kg/m$^2$), with achondroplasia as the most common diagnosis (n = 42; 75%). Primary indications for index surgery included neurogenic claudication (50%), cervical or thoracic myelopathy (23%), and lumbar radiculopathy (18%). Seventeen patients (30%) underwent a second spinal surgery, of which twelve were staged or planned. Seven required a third procedure, four a fourth, and two a fifth or sixth procedure. Complications following index surgery included intraoperative durotomy (n = 13), cardiovascular events (n = 6), neurologic deficits (n = 6), acute infections requiring debridement (n = 3), genitourinary (n = 3), thromboembolic (n = 3), pulmonary (n = 1), and gastrointestinal (n = 1). Complication rates decreased with subsequent surgeries, although durotomy remained most frequent.

**Conclusions:**

This expanded dataset provides updated evidence to guide surgical planning in patients with osteochondrodysplasias. Given the progressive nature of many skeletal dysplasias and their multilevel spinal involvement, a substantial proportion required multiple procedures or experienced worsening neurologic symptoms over time. These findings underscore the importance of long-term multidisciplinary surveillance and individualized surgical planning in this complex population.

### Disclosure

None declared

### P126

### Use and Perceived Utility of an Individualized School Plan for Optimal Inclusion of Students with Osteogenesis Imperfecta

Jessica Collins[1], Corinne Mercier[2], Argerie Tsimicalis[3]

[1]Shriners Hospital for Children - Canada, Montreal, Canada. [2]Shriners Hospital for Children-Canada, Montreal, Canada. [3]McGill University, Montreal, Canada

### Abstract

**Background:**

Children with Osteogenesis Imperfecta (OI) experience fractures, chronic pain, fatigue, and mobility limitations that can significantly affect participation and safety in school environments. Families often report challenges communicating OI-specific needs to school personnel and navigating educational systems. In 2021, Shriners Hospitals for Children – Canada (SHC-Canada), in collaboration with SUNNAAS Rehabilitation Hospital and a patient-parent partner, developed the *Individualized School Plan for Optimal Inclusion of Students with OI* to support school planning and inclusion.

**Objective:**

To evaluate the use and perceived utility of this school-integration tool over a two-year period.

**Methods:**

SHC-Canada physiotherapists and occupational therapists distributed the tool to families requesting school-related support. Descriptive data were collected on child characteristics and plan completion. Caregiver and therapist feedback regarding satisfaction, relevance, and areas for improvement was obtained.

**Results:**

From June 2022 to May 2024, the tool was provided to 25 children with OI aged 2–15 years (mean 7.3). Diagnoses included OI Types I (n=3), III (n=3), IV (n=12), V (n=3), VI (n=3), and VII (n=1). Plans were completed collaboratively by caregivers and SHC clinicians in 32% of cases (n=8); remaining plans were completed by caregivers and/or local therapists. Feedback was received from 12 caregivers and five therapists. Overall satisfaction was reported by 94% of respondents. One caregiver identified a need for additional guidance related to physical education. Three caregivers reported using the tool primarily to prepare for school integration meetings rather than sharing it directly with school staff.

**Discussion:**

The Individualized School Plan was perceived as a useful and comprehensive resource to support school inclusion for students with OI. It appeared particularly beneficial for children with moderate to severe OI, while a streamlined version may be more appropriate for milder phenotypes. Further evaluation of phenotype-specific school accommodation needs is warranted.

### Disclosure none declared

### P127

### Collaborative Practice-Based Creation of a Podcast Series on Virtual Reality for Pain and Anxiety in Children's Orthopaedic Healthcare

Jessica Ding[1], Alisha Michalovic[2], Emily K Drake[3], Argerie Tsimicalis[1,2]

[1]Shriners Hospitals for Children - Canada, Montreal, Canada. [2]McGill University, Montreal, Canada. [3]Mount Allison University, Sackville, Canada

### Abstract

**Objectives:**

Significant levels of pain and anxiety are experienced by children undergoing medical procedures, such as orthopaedic surgeries. Pain and anxiety may be lessened by distraction techniques such as virtual reality (VR). However, barriers have contributed to a significant lag in implementing VR into clinical practice. This practice-based study sought to collaboratively create a podcast series to promote knowledge dissemination and VR implementation into practice.

**Methods:**

A practice-based study, guided by the principles of andragogy and cognitive theory of multimedia learning (CTML), was conducted. Twelve experts in the field of VR participated in a one-time, audio-recorded interview and contributed to the creation of their respective episode, on topics related to VR evidence, use, integration, creation, troubleshooting and decision-making. Across the episodes, a thematic analysis was conducted to inform a summary episode to the podcast series. The process of creating the podcast series was documented.

**Results**:

Nine podcast episodes were created with 12 participants including a child life specialist, a clinical research coordinator, doctors, decision-makers, nurses and clinicians. Main themes identified included VR benefits and future use, clinician difficulties, immersion, interaction with users, non-universality of VR, adverse effects and practice implementation.

**Conclusions:**

Podcasts offer a novel way to disseminate knowledge but there has been limited understanding of their optimal structure. This paper delineates the systematic approach undertaken to produce a podcast series to disseminate science. The created podcast series will help disseminate knowledge on VR use in children's orthopaedic healthcare, consequently promoting its implementation into practice.

### Disclosure

None declared

### P128

### The Experience of Spinal Bracing at a UK-Based Paediatric Osteogenesis Imperfecta Centre

Emilie Hupin, Alice Walker, Zoe Bowman Bayles, Abigail Bolland, Mark Heathfield, Alex Chesover, Moira Cheung, Belinda Crowe, Catherine DeVile

Osteogenesis Imperfecta Service, Great Ormond Street Hospital for Children NHS Foundation Trust, London, UK

### Abstract

**BACKGROUND:**

Osteogenesis Imperfecta (OI) is a bone fragility disorder typically caused by a defect in the genes which produce type 1 collagen. Features include propensity to fracture and skeletal deformities. The development of scoliosis is common, with overall prevalence ranging from 39% - 80%. Evidence shows that in children, untreated spinal curves may progress, and timely intervention is key in preventing complications such as pain and reduced pulmonary function. Conservative, medical and surgical management options should be considered for best outcomes.

**PRESENTING PROBLEM:**

The use of spinal bracing for scoliosis management in OI appears to vary internationally. Previous literature suggests the risk of rib fractures and chest wall deformities is high, tolerance poor, and bracing is not effective in stabilising spinal curves.

Some of the research pre-dates bisphosphonate therapy and more recently spinal bracing is being considered across OI centres.

**CLINICAL MANAGEMENT:**

At a UK-based paediatric OI centre, spinal bracing is being trialled to support scoliosis management (N=15). Orthotic devices varied depending on clinical presentation, age, mobility, and the decision-making process involved the multidisciplinary team, orthotist and patient /family. Details regarding function, comfort and tolerance post-orthotic intervention were recorded. Interval radiology to evaluate progression of curves and spinal orthopaedic monitoring continued as normal.

Table 1.

**DISCUSSION:**

Our data suggest that using spinal bracing in OI is not without complications, with tolerance issues and discomfort frequently reported. In some cases, mobility was negatively impacted, and wear was interrupted by limb fractures or surgery. Orthosis modifications were often required. 6 patients achieved prolonged periods of wear (> 8 hours per day), and others continue to build up use gradually. Orthotic management was discontinued in 3 cases; one following skeletal maturity, one due to poor tolerance and one who proceeded to spinal surgery. To date, no serious adverse events such as fractures have occurred.

Further investigation is needed to determine the effect of bracing on spinal curve progression and chest wall shape over time. Bracing in paediatric OI requires careful MDT consideration and monitoring. We continue to explore spinal bracing as a safe and appropriate adjunct for scoliosis management.

Table 1

| ***Sex*** | ***Age at initial bracing*** | ***OI type/ (Genetics)*** | ***Cobb angle*** | ***Orthotic device*** | ***Adverse effects*** | ***Wear > 8 hours*** |
|---|---|---|---|---|---|---|

| | | | | | | |
|---|---|---|---|---|---|---|
| | ***(yrs)*** | | | | | |
| M | 16 | Type 4 (*COL1A1*) | 24° | Rigid TLSO[1] | Discomfort, poor tolerance | No* |
| F | 12 | Type 1 (*COL1A1*) | 38° | Rigid TLSO | Discomfort, reduced balance | Yes (night) |
| M | 15 | Type 1 (*COL1A1*) | 27° | Rigid TLSO | Discomfort, self-conscious | No |
| F | 7 | Type 8 (*P3H1*) | 65° | Soft Boston brace | Negative impact on floor mobility | Yes |
| M | 12 | Type 3 (*COL1A1*) | 48° | Bivalve soft Boston brace | Overheating, poor fit in wheelchair | No |
| M | 10 | Type 3 (*COL1A2*) | 34° | Soft Boston brace | Discomfort, poor tolerance | No |
| F | 2 | Type 7 (*CRTAP*) | 57° | Semi-rigid TLSO | Negative impact on floor mobility | Yes |
| M | 15 | Type 3 (*COL1A1*) | 67° | Rigid TLSO | Nil reported | Yes (night) |
| M | 5 | Type 8 (P3H1) | 31° | DEFO[2] | Carer anxiety | No |
| F | 2 | Type 15 (*WNT1*) | 32° | DEFO | Unable to use during limb fractures | Yes |
| M | 10 | Type 7 (*CRTAP*) | 69° | Posterior Boston brace with abdominal binder | Reduced tolerance, skin redness | No |
| F | 8 | Type 4 (*COL1A2*) | 39° | Semi-rigid TLSO | Overheating | Yes |
| F | 8 | Type 11 Bruck (*FKBP10*) | 18° | Soft Boston brace | Reduced tolerance, poor fit in wheelchair | No |

| | | | | | | |
|---|---|---|---|---|---|---|
| F | 6 | Type 1 /HGPPS[3] (*COL1A1*/*ROBO3*) | 78° | Rigid TLSO | Poor tolerance – refusal to wear | No* |
| M | 9 | Type 8 (*P3H1*) | 45° | DEFO | Poor tolerance – risk of fracture when applying | No* |

*[1]TLSO – thoracolumbar support orthosis*

*[2]DEFO- dynamic elastomeric fabric orthosis (Lycra suit)*

*[3]Horizontal gaze palsy with progressive scoliosis*

**Bracing discontinued*

### Disclosure

None declared

### P129

### Quantifying Forearm Deformities in Pediatric Patients with Osteogenesis Imperfecta to Predict the Likelihood of Radial Head Subluxation and Dislocation

Zoe Frias[1], Kory Johnson[1], Kevin Mesinas[1], Maegen Wallace[2,1]

[1]The University of Arizona-Phoenix, Phoenix, USA. [2]Phoenix Children's Hospital, Phoenix, USA

### Abstract

**OBJECTIVES:**

In pediatric patients with osteogenesis imperfecta (OI), forearm deformities and radial subluxation or dislocation can cause functional deficits and increase fracture risk. Previous studies have shown that bowing of the ulna is associated with both subluxation and dislocation of the radial head in multiple hereditary exostosis[1]. This study aimed to determine which factors are associated with radial head instability. These findings may help identify patients who would benefit from early surgical intervention.

**METHODS:**

A retrospective review was conducted on pediatric patients with OI from a single institution from January 2004 to July 2024. Data collected included patient age, OI type, and forearm surgeries if applicable. Radiographs of the forearm were also collected and the status of the radial head as located, subluxed, or dislocated was documented. Measurements for the radius and ulna on both the anteroposterior and lateral views were measured on all 125 films. Multivariable regression models were performed to determine the association between forearm deformity and radial head subluxation/dislocation.

**RESULTS:**

Subluxation and/or dislocation of the radial head was present in 10.4% of forearms. Bivariate analyses found that increased measured deformity on radiographs was associated with increased likelihood of both radial head subluxation and dislocation, seen on AP images of the radius (OR=1.08, $p<0.05$), lateral images of the radius (OR=1.04, $p<0.05$), as well as AP and lateral images of the ulna (OR=1.09, $p<0.05$); (OR=1.09, $p<0.05$). Age, OI type, and presence of a fracture line on x-ray were not significant factors. Multi-level deformity at the radius showed the strongest association with instability of the radial head on both AP (OR=7.9, $p<0.05$) and lateral x-rays (OR=9.6, $p<0.05$). Radius AP deformity (OR=1.19, $p<0.05$) and ulnar lateral deformity (OR 1.16, $p<0.05$) remained significant predictors for radial head instability within subsequent multivariable logistic regression.

**CONCLUSIONS:**

Subluxation and dislocation of the radial head are associated with severe forearm deformities in pediatric patients with OI. These findings demonstrate the possible benefit of early surgical intervention in pediatric OI patients with multilevel deformity of the radius.

### Disclosure

None declared in relationship to this work

### P130

### Crossing a New Bridge: Understanding the Abyss of Transition of Care for Teenagers and Young Adults with Osteogenesis Imperfecta

Ali Siddiqui, Mahim Jain, Cristina McGreal, Sarah Little, Ricki Carroll, Jeanne Franzone

Nemours Children's Hospital Alfred I. duPont Campus, Wilmington, USA

### Abstract

#### Objectives

Osteogenesis imperfecta (OI) is a genetic condition with bowing of the long bones and non-orthopaedic manifestations. OI care is best performed by a multidisciplinary team typically seen in pediatric hospitals. Transition of care to the adult system is a challenge and limitation. At our pediatric center we care for OI patients through age 35 and here seek to define 1) what orthopaedic surgeries are needed from age 18-35 and 2) how does the complication rate compare to the less-than-18 age group?

#### Methods

This single-center retrospective study included patients 18-35 with OI undergoing orthopaedic surgeries from 2018-2024 with minimum one-year follow-up. Patients were matched with an OI patient less than 18 years by bone segment and OI severity moving retrospectively, going back no further than needed. Chart review identified patient demographics and surgical data. Comparisons were evaluated by chi-square test.

#### Results

Twenty-one patients with OI ages 18-35 (11 females/10 males, mean-age 22.0, SD 3.7), OI Type I-1, III-12, IV-7, VIII-1) underwent 29 procedures: Realignment/rodding of the femur-7, tibia-4, humerus-11; olecranon ORIF-1, femoral neck fixation-1, removal prominent screw/part of rod-3, greater trochanteric resection-1, rod back-up-1. Seventeen/23 rodding procedures were revisions. Of 29 young-adult procedures, 6 were for acute fracture, 4 for subacute fracture, 19 for discomfort/deformity/nonunion. Matches were made for 23 of the procedures for which new implants were placed (average age 13.3, SD 2.5). The young adult group demonstrated 13/23 complications (Table) and 6/23 required reoperation. The matches had 6/23 complications ($p<0.05$) and 2/23 reoperations ($p=0.12$). Nonunion or delayed union was observed in 7 individuals in the 18-35 group and 1 in the matched group ($p<0.05$).

#### Conclusions

Patients with OI in the age 18-35 category continue to require both urgent and elective orthopaedic surgeries with a higher complication rate, highlighting the need to improve transition of OI care into young adulthood and electively perform indicated OI rodding and revision procedures prior to the age of 18. This investigation sheds new light on the orthopaedic needs of OI patients approaching age 18 and in their young adult years who continue to require multidisciplinary OI surgical care with higher complication rates.

| | 18-35 group | Matches |
|---|---|---|
| Nonunion/rod prominence | 1 – 1 reoperation | |
| Delayed union | 2 – no reoperation | |
| Nonunion | 4 – 1 no reoperation; 3 reoperation | |
| Peri-implant fracture with nonunion | | 1 – 1 reoperation |
| Persistent pain, self-resolved | 1 – no reoperation | |
| Rod prominence/migration | 2 – 1 reoperation | 3 – 1 reoperation |
| Transfixion pin prominence | 1 – no reoperation | |
| Peri-implant fracture | 1 – 1 reoperation | 1 – no reoperation |
| Transient radial nerve palsy | | 1 – no reoperation |
| Elbow stiffness | 1 – no reoperation | |

### Disclosure

Franzone - Consultant, Orthopediatrics

### P131

### Air Displacement Plethysmography in Children and Adults with Osteogenesis Imperfecta

Ms Kinsley Wang[1], Dr Danita Velasco[2], Dr Joan Eckerson[3], Dr Maegen Wallace[4]

[1]University of Arizona-Phoenix, Phoenix, USA. [2]University of Nebraska Medical Center, Omaha, USA. [3]Creighton University, Omaha, USA. [4]Phoenix Children's Hospital, Phoenix, USA

### Abstract

**Objectives:**

Osteogenesis Imperfecta (OI) is a genetic disorder resulting in brittle bones, extremity deformities, short stature, scoliosis and varying extrasekeletal features. Traditional measures of health via body mass index are not reliable in children with OI. Previous studies have utilized dual-energy x-ray absorptiometry (DEXA) to measure body fat percentage (BF%) and have been found to correlate with nutritional status and arm circumference in children with OI.(1) Air displacement plethysmography (ADP) is a non-invasive way to measure body composition and has been found to be reliable in children(2) and adults. This is particularly relevant when assessing children with conditions like OI, where traditional measurement techniques might be challenging due to mobility issues or discomfort.(2)

**Methods:**

Children and adults with OI were recruited to participate in the study at two pediatric OI programs in the United States. Demographic data, age, gender, OI type, anthropometric measures skin caliper measurements, and ADP measurements were collected and analyzed.

**Results:**

Demographic and BF% data is in table 1. Average height 114 cm, average arm span 115 cm, average arm circumference 22.4 cm, average weight 37.2 kg (range 12.5-89.6 kg). BF% was found to increase with age and was significantly different (p=0.014); ages 3-7 16%, ages 8-11 26% and >11 31.2%. We did find a correlation between body fat percentage and skinfold measurements with a correlation coefficient of 0.78 and p value <0.001.

**Conclusions:**

ADP is a reliable method of assessing body composition in individuals with OI. There was no difference in BF% between OI types, ambulation status or gender. There was a significant increase in BF% as children age, especially between the 3-7 age group (16%) and the 8-11 age group (26%). This information can help physicians and parents of children in this age group to be aware of these common changes. Utilizing this technology, we will be able to monitor children over time and evaluate dietary and physical activity interventions for effectiveness. Early identification of abnormal body composition, not just changes in weight, can lead to timely interventions, potentially preventing long-term health issues.

References

1. Zambrano MB, Brizola ES, Refosco L, Giugliani R, Félix TM. Anthropometry, Nutritional Status, and Dietary Intake in Pediatric Patients with Osteogenesis Imperfecta. J Am Coll Nutr. 2014 Feb;33(1):18–25.

2. Fields DA, Allison DB. Air-Displacement Plethysmography Pediatric Option in 2–6 Years Old Using the Four-Compartment Model as a Criterion Method. Obesity. 2012 Aug;20(8):1732–7.

| Table 1 Demographic and Body Fat Percentage Data | | | | | | | | |
|---|---|---|---|---|---|---|---|---|
| **Gender** | | | | | | | | |
| | **Male** | **Female** | **Total** | | | | | |
| | 19 | 22 | 41 | | | | | |
| **OI Types** | | | | | | | | |
| | **I** | **III** | **IV** | **V** | **VIII** | **XV** | **unknown** | **Total** |
| | 10 | 12 | 14 | 2 | 1 | 1 | 1 | 41 |
| **BF%** | | | | | | | | |
| | **Male** | **Female** | **p value** | | | | | |
| | 25.70% | 26.50% | 0.87 | | | | | |
| | **Mild OI** | **Moderate/severe OI** | **p value** | | | | | |
| | 25% | 28.60% | 0.47 | | | | | |
| | **Ambulators** | **Non-ambulators** | **p value** | | | | | |
| | 25% | 29.90% | 0.43 | | | | | |

### Disclosure

None declared

### P132

### A Quality Improvement Initiative – Optimizing Post Surgical Discharge Planning of Paediatric Orthopaedic Patients Through Case Studies

Jessica Ding[1], Androniki Tsoybariotis[1], Margaret Lee[1], Mariane Kieski Martins[1], Rosa Alicia Paradelo[1], Sabrina Horan[1], Argerie Tsimicalis[1,2]

[1]Shriners Hospitals for Children - Canada, Montreal, Canada. [2]McGill University, Montreal, Canada

### Abstract

### Objectives:

Effective post-surgical discharge planning of paediatric orthopaedic patients is essential to prevent complications and familial distress. Poorly executed discharges may lead to increased resource utilization, emergency department readmissions, and compromise future health care encounters. The purpose of this quality improvement initiative was to apply the latest evidence to optimize the nursing-led discharge practices by reviewing the literature, delineating concerns, troubleshooting pathways of care, and creating new training material for incoming nurses.

### Methods:

A single-site, three-step, quality improvement initiative was undertaken on an award-winning, orthopaedic, paediatric, post-surgical inpatient unit. The first step consisted of a rapid review to understand the discharge needs of patients and caregivers. The second step entailed interviewing 15 nurses from both day and evening shifts to delineate the main discharge concerns of patients and their family, which have led to: further follow up, resource utilization or hospital readmission. Pathways were identified, which could address these concerns, with the goal of optimizing the navigation of the healthcare system from a patient/family perspective. The final step triangulated the findings, which were used to collaboratively create five case studies for training incoming new nurses on the post-operative, discharge practices and processes.

### Results:

Situated within the real-world practice of the study setting, and the broader context of patients and families navigating the health care system with limited health literacy, five discharge case studies were collaboratively created with four nurses. These cases seek to guide incoming new nurses on how to troubleshoot post-surgical, orthopaedic patient discharges. A multitude of topics were covered: signs and symptoms of infection, compartment syndrome, wound and cast issues, pain control, symptomatic hardware, fractures on patients with osteogenesis imperfecta and postoperative gastrointestinal complications. The case studies also addressed broader social realities that patients may experience including language barriers, limited access to resources such as transportation and mental health issues. This material will be used to train all incoming new nurses with the goal of optimizing the discharges practices and pathways.

### Conclusions:

Five discharge case studies were collaboratively created and are now being used to train incoming new nurses. Tracking of post-operative discharge cases requiring further follow up, resource utilization or hospital readmission are being tracked to determine whether other quality improvement initiatives are needed for optimization.

### Disclosure

None declared

### P133

### Investigating the Effects of Group Aquatic Therapy for Children with Osteogenesis Imperfecta

Mallory Gardner, Anthony Puglisi

Children's Hospital of Philadelphia, Philadelphia, USA

### Abstract

OBJECTIVES:

Aquatic physical therapy is routinely recommended for children with Osteogenesis Imperfecta (OI) as a safe modality for muscle strengthening, weight-bearing, and balance exercise. The buoyancy of the water protects from falls while the viscosity provides resistance for strengthening. Despite this recommendation, there is little evidence on the effects of aquatic physical therapy on muscle strength, muscle power, or function in children with OI. The objectives of this case series are to outline specific exercises appropriate for ambulatory children with OI and to review the effects on muscle strength, muscle power, and function after a six-week aquatic group therapy program.

METHODS:

The 50 minute weekly aquatic therapy group will focus on swimming, lower extremity strengthening activities, and balance exercise in floating, kneeling, and standing. Prior to the initiation of the program and within two weeks after the last session, leg strength will be assessed by handheld dynamometry, muscle power by broad jump distance and vertical jump height, and functional mobility with timed up and down stairs. The Water Orientation Test Alyn 2 will be used to assess function and swimming skills in the water. Baseline and post-intervention outcomes will be converted into and age and sex specific Z-scores using standard references and paired t-tests will be used to determine significance.

RESULTS:

To date, four patients with OI have agreed to participate in this pilot aquatics group (two females, two males, ages 8-11). Comparison of baseline and post 6-week intervention outcomes will be completed upon cessation of the program. Description of aquatic exercises will include walking activities, swim strokes, floatation exercises, balancing in varied positions, step ups, jumping, and lunging.

CONCLUSIONS:

We plan to supplement the evidence on aquatic physical therapy for ambulatory children with OI, by outlining specific exercises performed and the effects on muscle strength, muscle power, function, and swimming skills after six weeks in the group setting in the pool.

### Disclosure none declared

### P134

### Exploratory Predictive Modelling Using Parental Measures to Estimate DXA-Derived Bone Mass in Rural Indian Prepubertal Children

Sharvani Patil[1], Vivek Patwardhan[1], Rubina Mandlik[1], Nikhil Shah[1,2], Anuradha Khadilkar[1]

[1]Hirabai Cowasji Jehangir Medical Research Institute, Pune, India. [2]Department of Pediatric Endocrinology, MRR Children's Hospital, Mumbai, India

### Abstract

### Background:

Heritability accounts for roughly 50–80% of the variation in bone mineral density (BMD). We have previously shown that parental bone health influences that of children, even in cases of poor nutrition and also after adjusting for modifiable factors. This suggests that parental bone measures may provide useful information for estimating skeletal status in children, particularly in settings where routine DXA assessment/interpretation in children is limited or not readily available. Consequently, we aimed to develop an exploratory machine learning model to estimate DXA-derived total body less head bone mass in rural prepubertal children, incorporating parental areal bone mineral density(aBMD) as a predictor.

### Methods:

This cross-sectional observational study included 1,289 children(679 boys,218 sibling) and their 2,142 parents (1,071 mother–father pairs). Total body less head(TBLH) bone measures were assessed using a Lunar iDXA system (GE Healthcare; Encore version 18), with TBLHaBMD used for model development. Data were preprocessed and analysed using Python 3.10 with scikit-learn library. The dataset was split into training (n = 894) and test (n = 384) sets. Twenty regression models were evaluated, following which the best-performing model was selected, hyperparameter-tuned, and ensembled to optimise performance. Predictive accuracy was assessed on the test dataset using standard regression metrics. Models incorporated relevant child and parental variables, including parental aBMD.

### Results:

Linear regression demonstrated the best overall performance, with the lowest error metrics (MAE 0.0356, MSE 0.0021, RMSE 0.0459, MAPE 0.0599) and the highest coefficient of determination ($R^2$ = 0.578) among the evaluated models. Bayesian ridge and ridge regression showed comparable performance but did not outperform linear regression. Parental bone measures, particularly maternal aBMD, emerged as the strongest predictors. Predictive accuracy on the test dataset was 52%.

### Conclusion:

This exploratory model demonstrates stable generalisation with moderate predictive accuracy in unseen data(test dataset); as the model is primarily based on parental bone measures, individual bone measures may vary due to lifestyle factors. While current performance supports its potential use as a population-level screening/risk-stratification tool, validation in larger and more diverse cohorts across age groups, and ethnicities is required before clinical application.

Figure 1- A] comparing prediction models B] Residual Analysis of Linear Regression Model (Train vs Test)

Compare Models

| | Model | MAE | MSE | RMSE | R2 | RMSLE | MAPE | TT (Sec) |
|---|---|---|---|---|---|---|---|---|
| **lr** | Linear Regression | 0.0356 | 0.0021 | 0.0459 | 0.5615 | 0.0285 | 0.0599 | 1.6470 |
| **br** | Bayesian Ridge | 0.0357 | 0.0021 | 0.0459 | 0.5613 | 0.0285 | 0.0599 | 0.0150 |
| **ridge** | Ridge Regression | 0.0358 | 0.0021 | 0.0459 | 0.5602 | 0.0286 | 0.0601 | 0.0130 |
| **gbr** | Gradient Boosting Regressor | 0.0367 | 0.0022 | 0.0466 | 0.5479 | 0.0290 | 0.0615 | 0.0540 |
| **huber** | Huber Regressor | 0.0365 | 0.0022 | 0.0469 | 0.5417 | 0.0292 | 0.0612 | 0.0240 |
| **rf** | Random Forest Regressor | 0.0382 | 0.0024 | 0.0484 | 0.5127 | 0.0300 | 0.0640 | 0.1550 |
| **et** | Extra Trees Regressor | 0.0382 | 0.0024 | 0.0487 | 0.5076 | 0.0302 | 0.0639 | 0.1010 |
| **catboost** | CatBoost Regressor | 0.0382 | 0.0024 | 0.0488 | 0.5057 | 0.0303 | 0.0639 | 0.7990 |
| **lightgbm** | Light Gradient Boosting Machine | 0.0389 | 0.0024 | 0.0492 | 0.4954 | 0.0306 | 0.0651 | 0.3690 |
| **ada** | AdaBoost Regressor | 0.0391 | 0.0025 | 0.0496 | 0.4887 | 0.0308 | 0.0659 | 0.0460 |
| **xgboost** | Extreme Gradient Boosting | 0.0396 | 0.0025 | 0.0503 | 0.4728 | 0.0313 | 0.0663 | 0.0490 |
| **omp** | Orthogonal Matching Pursuit | 0.0437 | 0.0031 | 0.0553 | 0.3618 | 0.0344 | 0.0736 | 0.0130 |
| **par** | Passive Aggressive Regressor | 0.0472 | 0.0035 | 0.0593 | 0.2676 | 0.0369 | 0.0785 | 0.0140 |
| **knn** | K Neighbors Regressor | 0.0476 | 0.0036 | 0.0597 | 0.2580 | 0.0371 | 0.0798 | 0.0210 |
| **lasso** | Lasso Regression | 0.0551 | 0.0049 | 0.0700 | -0.0168 | 0.0434 | 0.0924 | 0.5170 |
| **en** | Elastic Net | 0.0551 | 0.0049 | 0.0700 | -0.0168 | 0.0434 | 0.0924 | 0.0130 |
| **llar** | Lasso Least Angle Regression | 0.0551 | 0.0049 | 0.0700 | -0.0168 | 0.0434 | 0.0924 | 0.0130 |
| **dummy** | Dummy Regressor | 0.0551 | 0.0049 | 0.0700 | -0.0168 | 0.0434 | 0.0924 | 0.0130 |
| **dt** | Decision Tree Regressor | 0.0552 | 0.0049 | 0.0698 | -0.0203 | 0.0433 | 0.0926 | 0.0140 |
| **lar** | Least Angle Regression | 0.0524 | 0.0078 | 0.0666 | -0.5964 | 0.0409 | 0.0882 | 0.0140 |

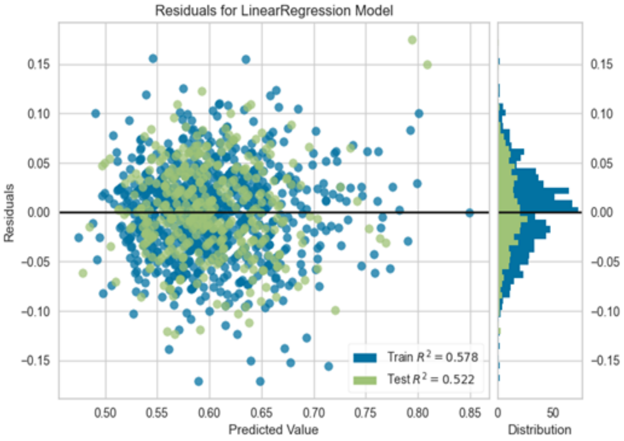

### Disclosure

None declared

### P135

### The Impact of an Achondroplasia MDT clinic - A Two-Cycle Audit

Robyn Haysom[1], Amish Chinoy[2]

[1]University Hosptials of the North Midlands, Stoke-on-Trent, UK. [2]Royal Manchester Children's Hospital, Manchester, UK

### Abstract

**Objectives:**

Achondroplasia (ACH) is characterised by disproportionate short stature, and a range of multisystem complications which necessitate regular clinical surveillance, as per international guidelines. This audit aimed to assess adherence to these recommendations at a tertiary paediatric centre and evaluate the impact of introducing a multi-speciality multidisciplinary team (MDT) clinic.

**Methods:**

A retrospective two-cycle audit was conducted. Monitoring practices were assessed across seven key domains (as recommended in published guidelines: auxology, neurology, respiratory/sleep, ENT/audiology, orthopaedic, psychosocial, occupational therapy, dental). Following the first audit cycle (June 2023-Aug 2024) an MDT clinic (including endocrinology, neurosurgery, respiratory, ENT, orthopaedic, occupational therapy, physiotherapy) was introduced to facilitate more coordinated surveillance for these patients. A second cycle (Sept 2024-Dec 2025) was subsequently completed to assess the impact of these clinics on adherence to the guidelines.

**Results:**

Although some domains (e.g. auxology, occupational therapy) already had good adherence to recommendations, the introduction of an MDT clinic was associated with improvements in monitoring across all domains (Table 1), in particular for dental health (34% -> 74%), psychological assessment (71% -> 100%) and sleep/respiratory monitoring (36% -> 100%).   However, further improvements are required, which will form the basis of further actions.

**Conclusion:**

This two-cycle audit demonstrates the value of a multi-speciality MDT clinic in improving adherence to international monitoring recommendations for children with achondroplasia, this improving safety and holistic care for these patients

SUMMARY OF RESULTS:

0-25% 25-50% 50-75% 75-100%

| Average improvement in outcome measure across each domain | CYCLE 1 | CYCLE 2 | % improvement |
|---|---|---|---|
| General/Auxological Data | 90% | 96% | +6% |
| Neurological Assessment | 64% | 65% | +0.5% |
| Sleep/Respiratory Monitoring | 36% | 100% | +64% |
| ENT/Hearing Assessments | 66% | 92% | +26% |
| Orthopaedic Assessment | 66% | 84% | +18 |
| Psychosocial Assessment | 38% | 78% | +41% |
| Occupational Therapy | 71% | 100% | +29% |
| Dental Assessment | 34% | 74% | +40% |

### Disclosure

None Declared

### P136

### Use of Standardised Motor Assessments to Evaluate Developmental Motor Skills in Children with Osteogenesis Imperfecta

Lisa Mills[1], Amy Pilkington[1], Hannah Bassett[1], Toby Candler[1,2]

[1]Bristol Royal Hospital for Children, Bristol, UK. [2]NIHR Biomedical Research Centre (Diet & Physical Activity Theme), University of Bristol, Bristol, UK

### Abstract

Developmental motor skills and praxis underpin performance of functional movement and participation in daily activities. In Osteogenesis Imperfecta (OI), skeletal fragility, hypermobility, pain, and reduced muscle strength may disrupt typical motor development. Clinical monitoring often focuses on motor milestones and mobility, but standardised motor assessments may offer a more detailed evaluation of functional abilities.

### Objectives

- To evaluate the utility and compare outcomes of two widely used standardised motor assessments in children with mild–moderate OI.
- To explore the range of motor skill strengths and difficulties in this group

### Methods

Twenty-three children with mild-moderate OI attending a UK specialist centre were invited to participate. Participants completed the Movement Assessment Battery for Children–3 (MABC3) and the Bruinincks–Oseretsky Test of Motor Proficiency–2 (BOT2) in two separate appointments. Pain and fatigue (0–10) were rated pre and post assessment. Children also rated task difficulty and enjoyment (1–6). Administration and scoring times were recorded. Outcomes were analysed using Bland–Altman plot, Spearman's Rank correlation, and Mann-Whitney U calculations.

### Results

Twenty children completed both assessments; 12F:8M; median age ten years(range=6-14). Median total previous fractures six (range=0-14); six were receiving bisphosphonates and three had prior rodding surgery. Mean composite motor scores were within the average range (BOT2=41.56; MABC3=44.5), with good agreement and correlation between tests (R=0.734). Participants showed weaker performance across several subtests; fine motor precision, co-ordination, strength, and gross motor agility tasks. Mean differences between pre and post-assessment ratings of pain (BOT2=0.8; MABC3=0.6, p=0.182) and fatigue (BOT2=0.5; MABC3=0.85, p=0.399) ratings were low and similar across assessments. Mean enjoyment ratings were comparable (BOT2=1.65/6; MABC3=1.95/6, p=0.342). Scoring time was identical (38 minutes). MABC3 was rated easier (BOT2=2.85/6; MABC3=2.35/6, p=0.016). BOT2 administration was longer (56 vs 45 minutes, p=<0.001). but assessed a broader range and depth of skills.

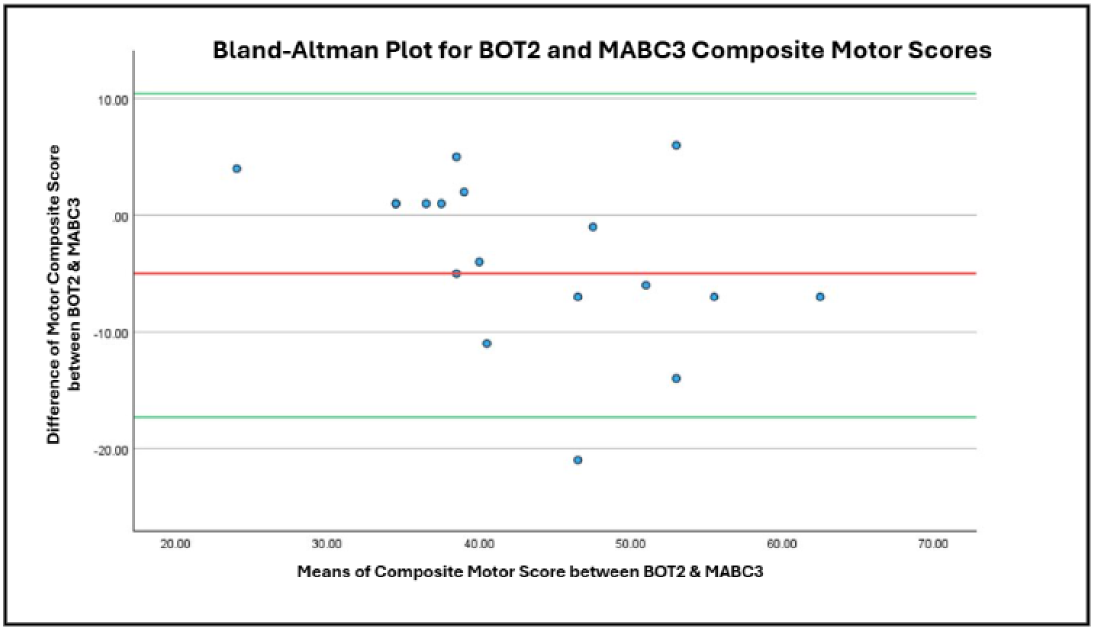

**Conclusions**

Standardised motor assessments are valuable in identifying specific motor difficulties, informing targeted therapy when administered by clinicians experienced with children with OI. Both assessments were comparable across a range of outcome and experience measures. Further research is warranted to evaluate applicability for children with more severe OI phenotype.

### Disclosure

None declared

### P137

### Navigating the Move to Adult Services: Consultation Insights from Skeletal Dysplasia Youth and Their Parents

Anita Freeman, Catherine Ali, Marianna Shiafkou, Emma Bewick, Emilie Hupin, Daniela Melamed, Emily Golding, Rachael Worthen, Moira Cheung

Great Ormond Street Hospital NHS Trust, London, UK

### Abstract

**Objectives:**

Young people with skeletal dysplasia (SKD) face complex physical, psychological, and social challenges as they transition from paediatric to adult healthcare. Despite established transition frameworks, limited research has explored condition-specific needs and priorities for this population.

**Methods:**

A service development consultation was conducted at Great Ormond Street Hospital for Children between July and November 2025. Parallel focus groups with young people (aged 15–20) and parents explored experiences of gaining independence, priorities for transition preparation, and perspectives on multidisciplinary care. Transcriptions and facilitator notes underwent thematic analysis, followed by multidisciplinary team (MDT) review and parent feedback.

**Results:**

Nine young people expressed an interested in joining the consultation, and a total of 4 young people and 5 parents were able to join the focus groups (Figure 1). The thematic analysis from the focus groups highlighted systemic, psychological and practical barriers to independence, including navigating visible difference, fluctuating support needs in education, challenges using public transport, and difficulties advocating in healthcare settings. Parents emphasised concerns about readiness for medical decision-making, inconsistent coordination across services, and the need for structured preparation. Young people prioritised understanding their condition, developing confidence in appointments, and accessing meaningful peer support. Across groups, early preparation, clear information-sharing, and holistic, well-coordinated care were identified as essential.

***Figure 1.*** Participant Demographics

| Sex | Age | Diagnosis | Young Person Group | Parent group |
|---|---|---|---|---|
| Female | 20 | Hajdu-Cheney Syndrome | Yes | Yes |
| Male | 17 | Pseudoachondroplasia | Yes | Yes |
| Female | 15 | Achondroplasia | Yes | Yes |
| Female | 16 | Chondrodysplasia | Yes | Yes |
| Male | 17 | Achondroplasia | No | Yes |

**Conclusions:**

Findings informed a set of targeted service developments, including independence-focused outpatient practices with pre-clinic preparation calls with the young person, annual peer workshops, joint OT–Physiotherapy sessions with patient led goal-based to enable independence (e.g. use of public transport), enhanced psychological support, and strengthened transfer-of-care processes. The consultation underscores the need for a developmentally attuned MDT support, with a SKD-informed, and family-inclusive transition pathway that promotes autonomy while recognising the unique social and emotional contexts faced by young people with SKD.

### Disclosure

None Declared

### P138

### A Global Pediatric Bone Diseases Curriculum with Initial Implementation in West Africa

Reggie Hamdy, Kelly Thorstad, Marie-Eve Robinson

Shriners Hospital for Children - Canada, Montreal, Canada

### Abstract

**OBJECTIVES:**

Children living with pediatric bone disorders in low- and middle-income countries (LMICs) face major gaps in access to safe, affordable, and sustainable care. The objective of this initiative is to develop and implement an international pediatric bone health curriculum for medical providers in LMICs, with an initial focus on West Africa.

**METHODS:**

We are developing a two-year, modular learning curriculum addressing high-impact pediatric bone disorders in LMICs. The curriculum will include a common core delivered to all participants, covering osteogenesis imperfecta, rickets, skeletal dysplasias and other pediatric bone diseases, followed by two tailored streams: a medical stream for non-surgical providers and a surgical stream for orthopedic surgeons. The initial cohort will aim to include 10 participants, comprising 5 medical providers and 5 orthopedic surgeons. Capacity building will use a blended model combining virtual knowledge transfer (biweekly webinars, bidirectional case discussions, and journal clubs), hands-on skills development (workshops using saw bones, visiting scholars, and six-week mini-fellowships), and support for local infrastructure development.

To inform curriculum design, a two-tiered needs assessment is being conducted in Abidjan, Côte d'Ivoire. Members of Shriners Hospitals for Children completed an initial on-site visit and identified persistent gaps in access to complex pediatric bone care despite established clinical infrastructure. A medical team from Shriners Hospitals for Children–Canada is scheduled to return in April 2026. Planned activities include collaborative pediatric orthopedic surgeries with local surgeons at CHU Cocody, alongside information acquisition initiatives.

**RESULTS:**

The curriculum framework is being established through international partnerships which will include McGill University, Shriners Hospitals for Children, Limb Lengthening and Reconstruction Society (LLRS) North America, Care4Bones, the Global Initiative for Children's Surgery, and patient advocacy organizations. Early engagement in Côte d'Ivoire demonstrates strong local interest and feasibility for piloting and refinement, and will help identify potential participants from West Africa while further informing curriculum development.

**CONCLUSIONS:**

This global pediatric bone health curriculum represents a scalable and sustainable approach to capacity building, with an initial focus on West Africa. By integrating needs-based curriculum development and skills training, the program aims to improve equitable access to pediatric bone care in LMICs.

### Disclosure

No relevant disclosures

### P139

### OPTIMIZE DMD: An International Learning Health Network Advancing Bone and Endocrine Health in Duchenne Muscular Dystrophy

Anne Marie Sbrocchi[1], David R Weber[2], Sze Choong Wong Wong[3], Melissa Fiscaletti[4], Natascia Di Iorgi[5], Nadia Merchant[6], Maria Fernanda Ochoa Molina[7], Nora Renthal[8], Claire Wood[9], Mei Wang[10], Roozbeh Manesh[10], Rana Halloun[10], Fernanda De Angelis[11], Filippo Buccella[11], Ilario Zito[11], Rachel Schrader[12], Pat Furlong[12], Leanne Ward[10,13]

[1]Montreal Children's Hospital, McGill University Health Centre, Montreal, Canada. [2]Montreal Children's Hospital of Philadelphia and the Perelman School of Medicine at the University of Pennsylvania, Philadelphia, USA. [3]Royal Hospital for Children; School of Medicine, Dentistry and Nursing, University of Glasgow, Glasgow, UK. [4]CHU Sainte-Justine, Université de Montréal,, Montreal, Canada. [5]IRCCS Istituto Giannina Gaslini, Genoa, Italy. [6]University of Texas Southwestern Medical Center, Dallas, USA. [7]Pontificia Universidad Católica de Chile, Santiago, Chile. [8]Boston Children's Hospital and Harvard Medical School, Boston, USA. [9]Great North Children's Hospital, University of Newcastle-Upon-Tyne, Newcastle Upon Tyne, UK. [10]Children's Hospital of Eastern Ontario Research Institute, Ottawa, Canada. [11]Parent Project APS, Rome, Italy. [12]Parent Project Muscular Dystrophy, Washington, USA. [13]Children's Hospital of Eastern Ontario, University of Ottawa, Ottawa, Canada

### Abstract

**Objectives:**

Individuals with Duchenne muscular dystrophy (DMD) experience a high burden of endocrine and bone health complications; however, management remains highly variable across clinical settings, reflecting gaps in evidence and a need for expanded, harmonized clinical guidance.

**Methods:**

Founded in 2023, the OPTIMIZE DMD Consortium was established to improve health outcomes by advancing consensus-driven, evidence-informed bone and endocrine care through international collaboration, education, research, advocacy, and knowledge dissemination.

**Results:**

The OPTIMIZE DMD Consortium is a learning health network that currently includes representatives from seven countries, including Canada, the United States, Italy, the UK, Chile, Belgium, and the Netherlands, with efforts underway to develop new partnerships. The Consortium consists of a Steering Committee (N=6) and six Working Groups (WGs): Adrenal Insufficiency (N=8), Growth and Puberty (N=10), Osteoporosis Prevention (N= 16), Sexuality and Adult Well-Being (N=11), Weight Management (N = 13), and Research (N = 11). The various WGs draw on expertise from endocrinologists (n=28), neuromuscular experts (n=11), psychologists (N=2), and a special educational needs and disability expert (N=1). Seven patient advocates from PPMD contribute to the WGs; in addition, Parent Project APS [Italy, N=5) and PPMD have supported three Annual OPTIMIZE DMD Consortium workshops held in Rome, Italy (2023, 2024 and 2025). These workshops have been instrumental in facilitating international consensus-building and dissemination of best practices through professional education and accessible knowledge translation. As of 8 February 2026, the Consortium's outputs have included patient- and clinician-centred hand-outs on endocrine-bone implications of a novel therapeutic, one clinician-targeted hand-out addressing updated adrenal insufficiency management guidelines, one peer-reviewed, published manuscript on adrenal insufficiency, two manuscripts under revision/submission, and six manuscripts in preparation or submission for peer review.

**Conclusions:**

OPTIMIZE DMD is a novel international model for addressing endocrine and bone health complications of DMD that is guided, and supported, by people with lived experiences and their advocates. Going forward, OPTIMIZE DMD is focused on publishing high-quality, peer-reviewed scholarly works, patient- and clinician-targeted knowledge dissemination efforts, and expanding global partnerships with other clinicians, scientists, and patient advocates who are passionate about optimizing endocrine and bone health care for individuals with DMD.

### Disclosure

AMS has served as a site investigator in vamorolone clinical trials funded by Reveragen and as a consultant to Catalyst Pharmaceuticals. DRW has served as a consultant to Catalyst, Inozyme, and Santhera. SCW has served as a consultant to Santhera, Roche, and Novartis and has received speaker honoraria from Nutricia, Sandoz, Novo Nordisk, and Roche. MF has received grant support from the University of Montreal, the Azrieli Research Centre of CHU Sainte-Justine, and Muscular Dystrophy Canada, and has participated in an Advisory Board meeting for Catalyst Pharmaceuticals. NDL has received consulting fees and lecture fees from Santhera Pharmaceuticals. NM has served on the advisory boards of BioMarin, Catalyst, Pfizer, BridgeBio, Ascendis, Kyowa Kirin, and Alexion. CW is an investigator for the GUARDIAN study (An open-label study to collect long-term safety and efficacy data from boys with DMD who have completed prior studies with vamorolone). She has received speaker fees from Pfizer and consultancy fees from PTC Therapeutics, Roche, Santhera, and Pfizer. LMW has been a consultant to Amgen, Ultragenyx, Kyowa Kirin, Roche, Angitia, Santhera, Catalyst, BioMarin, and Ipsen, with funds to Dr. Ward's institution, and has participated in clinical trials with Alexion, QED, Ultragenyx, Edgewise, ReveraGen, Ascendis, Roche, and Catalyst, with funds to Dr. Ward's Institution. MFOM, NR, MW, RM, FDA, FB, IZ, RS, and PF have nothing to declare.

### P140

### Characterization of Children and Adolescents with Classic CAH who had Slowed Bone Age Progression and Improved Height Prediction With Crinecerfont

Maria Vogiatzi[1], Martine Cools[2], Régis Coutant[3], Ayca Erkin-Cakmak[4], Oksana Lekarev[5], Laetitia Martinerie[6], Ron Newfield[7], Kyriakie Sarafoglou[8], Jean Chan[9], George Jeha[9], Sanjay Keswani[9], Rachel Manahan[9], Eiry Roberts[9], Julia Sturgeon[9], Mimi Kim[10]

[1]Children's Hospital of Philadelphia, Philadelphia, USA. [2]Ghent University Hospital and Ghent University, Ghent, Belgium. [3]CHU d'Angers, Angers, France. [4]University of California San Francisco and Benioff Children's Hospital, San Francisco, USA. [5]Weill Cornell Medicine/New York Presbyterian Hospital, New York, USA. [6]Robert Debré Hospital, APHP, Paris Cité University, and Inserm, Physiologie et Physiopathologie Endocriniennes, Paris, France. [7]Rady Children's Hospital and University of California San Diego, San Diego, USA. [8]University of Minnesota Medical School and School of Pharmacy, Minneapolis, USA. [9]Neurocrine Biosciences, Inc., San Diego, USA. [10]Center for Endocrinology, Diabetes, and Metabolism, Children's Hospital Los Angeles, University of Southern California, Los Angeles, USA

### Abstract

**Objectives:** In pediatric patients with classic congenital adrenal hyperplasia (CAH), excess androgens cause accelerated bone age (BA) maturation, leading to reduced adult height. Crinecerfont is a first-in-class corticotropin-releasing factor type 1 receptor antagonist FDA-approved as an adjunct to glucocorticoid replacement to control androgens in patients (≥4 years) with classic CAH. In the phase 3 crinecerfont CAHtalyst™ Pediatric trial (NCT04806451), no mean improvements in predicted adult height (PAH) were observed at 2 years. However, most participants were mid-to-late puberty at baseline with limited growth potential. Here, we characterize changes in BA advancement and PAH in a subset of CAHtalyst Pediatric participants.

**Methods:** Participants from the 28-week double-blind placebo-controlled period continued crinecerfont or were switched from placebo for the open-label and ongoing open-label extension. Participants with available assessments (baseline BA<14 yr [females], <16 yr [males]) who were not on an aromatase inhibitor at baseline were included for post hoc analysis; baseline was defined as start of crinecerfont treatment. Mean change from baseline in BA minus chronological age (BA-CA) and PAH (Bayley-Pinneau BA-based method) were assessed at Month 24 (M24) of the study.

**Results:** Among 103 randomized participants, 41 (16 female, 25 male) had available assessments at baseline and M24. Baseline characteristics (mean±SD) were: age (9.2±2.4 years), BA (11.0±2.5 years), BA-CA (1.8±2.0 years), PAH (166±9.6 cm); 26 (63%) had an advanced BA ≥1 year at baseline. At M24, changes from baseline in BA-CA in these 26 were: 4 (15%) showed a worsening of BA-CA (≥0.5 years), 8 (31%) showed no change (>-0.5 to <0.5 years), and 7 (27%) had improvement in BA-CA from 6 months up to 1 year. Furthermore, 7 (27%) participants had improvement in BA-CA of 1 year or more, with mean±SEM improvements in PAH observed at M24 in these participants (+6.6±1.6 cm).

**Conclusion:** A substantial proportion of youth with CAH and advanced BA treated with crinecerfont for up to 2 years experienced slowed BA advancement and improved PAH. The percentage of patients with improvement in BA advancement is higher than might be expected based on the natural history of CAH, in which continued worsening of BA advancement is often observed.

**Disclosure**

Maria G. Vogiatzi: has received research support from Neurocrine Biosciences, Spruce Biosciences, Crinetics Pharmaceuticals and Adrenas Therapeutics. She has served as a consultant for Neurocrine Biosciences, Spruce Biosciences, Crinetics Pharmaceuticals, Eton Pharmaceuticals, Adrenas Therapeutics and Lundbeck Pharmaceutical company. Martine Cools: receives research support from the Research Foundation Flanders (unrelated to this work) and has served as a consultant for Sandoz, Novo Nordisk and Pfizer on behalf of the University of Minnesota Medical School but does not receive personal income for these activities. Régis Coutant: nothing to disclose. Ayca Erkin-Cakmak: serves/ed as an investigator on adrenal-related clinical trials with Neurocrine Biosciences. Oksana Lekarev: serves as a Principal Investigator on adrenal-related clinical trials with Neurocrine Biosciences. She has also received consulting fees from Neurocrine Biosciences and Eton Pharmaceuticals. Laetitia Martinerie: has received consulting fees from Neurocrine Biosciences, Crinetics Pharmaceuticals, Amaris Consulting and Lundbeck; and is a principal investigator for the studies conducted by Neurocrine Biosciences, Crinetics Pharmaceuticals and Recordati Rare Diseases. Ron S. Newfield: serves/ed as an investigator on adrenal-related clinical trials with Neurocrine Biosciences, Spruce Biosciences, and Crinetics Pharmaceuticals, and served in the past as a Speaker for the Neurocrine Speakers Bureau. Kyriakie Sarafoglou: has received research support from Office of Orphan Products Development of the Food and Drug Administration (R01FDR0006100), NIH-NCATS (UL1TR002494), NIH NINDS (U54NS115198), Neurocrine Biosciences, Spruce Biosciences, Crinetics Pharmaceuticals, Adrenas Therapeutics (BridgeBio), Alexion Pharmaceuticals, and BioMarin Pharmaceuticals; and has served as a consultant for Novo Nordisk, Eton Pharmaceuticals, Neurocrine Biosciences, Spruce Biosciences, and Crinetics Pharmaceuticals on behalf of the University of Minnesota Medical School but does not receive personal income for these activities. Jean L. Chan: employee of Neurocrine Biosciences, Inc. George S. Jeha: employee of Neurocrine Biosciences, Inc. Sanjay Keswani: employee of Neurocrine Biosciences, Inc. Rachel Manahan: employee of Neurocrine Biosciences, Inc. Eiry Roberts: employee of Neurocrine Biosciences, Inc. Julia Sturgeon: employee of Neurocrine Biosciences, Inc. Mimi S. Kim: has received research support from Neurocrine Biosciences, Spruce Biosciences, Neurocrine UK Limited, and Adrenas Therapeutics and served as a consultant for Neurocrine Biosciences.

### P141

### Empowering Young People with Chronic Musculoskeletal Conditions During Healthcare Transition Through Co-Produced Digital Education: '*Welcome to Adult Care at the RNOH*'

Alexander Chesover[1,2], Sophie Barlow[2], Amy Culham[2], Marianna Shiafkou[1], Mark Healthfield[1], Maryam Sherif[3], Kyrio Brooks[3], Kayleigh Hoey[2], Cillian Gray[1]

[1]Great Ormond Street Hospital for Children, London, UK. [2]Royal National Orthopaedic hospital, Stanmore, UK. [3]Patient Author, London, UK

### Abstract

**OBJECTIVE**

Improving healthcare transition, empowering young people, and ensuring safe transfer of care to adult services are national priorities. We developed transfer pathways from Great Ormond Street Hospital for Children (GOSH) to adult services for endocrine sub-specialty cohorts; most young people with metabolic bone conditions transferred to the Royal National Orthopaedic Hospital (RNOH). We aimed to develop patient-centred educational resources to support this process, resulting in the co-production of a video with young people.

**METHODS**

Young people aged over 13 years attending endocrine clinics were invited to complete a survey exploring their needs and priorities for healthcare transition, informing the development of patient-centred resources. In response to survey findings, we sought to provide accessible, developmentally appropriate information about what to expect when transferring to adult care at RNOH for those with chronic musculoskeletal conditions. A multidisciplinary project team across GOSH and RNOH, including healthcare transition leads, worked in partnership with young people to co-produce a storyboard, script, and video. Feedback was incorporated from clinical teams, the Young People's Forum, and young people attending the metabolic bone clinic. The project was funded by GOSH Charity.

**RESULT**

Survey responses were received from 26 of 83 young people (aged 14-17 years). Thematic analysis identified a clear need for improved information about transfer of care and adult services.

A co-produced video was developed to explain the transition from paediatric to adult care and introduce services and facilities at the RNOH. Filming involved staff, volunteers, and young people with metabolic bone conditions. The video launched on 5 November 2025 and had 490 views by 2 January 2026.

Additional outputs included a 'behind-the-scenes' preview and peer-to-peer interviews with young people reflecting on their healthcare transition experiences and involvement in this project.

**CONCLUSION**

We successfully developed and launched a co-produced educational video to support healthcare transition for young people with chronic musculoskeletal conditions. This work demonstrates the value of empowerment-focused education and co-production, aligning with national strategy and guidance on developmentally appropriate healthcare transition. Ongoing evaluation will assess impact and inform wider adoption across services and charities seeking to empower young people during transition to adult care.

### Disclosure

None declared

### P142

### Complex Treatment Decisions in a Patient with Hydatid Disease and Severe Osteogenesis Imperfecta

Louise Apperley[1], Charlotte Jarvis[1], David Porter[1], Dennis Padi[1], Andrew Healey[1], Nasim Mahmood[1], Stephanie A Borg[2], Poonam Dharmaraj[1]

[1]Alder Hey Children's Hospital, Liverpool, UK. [2]Sheffield Children's Hospital, Sheffield, UK

### Abstract

### Background

Untreated severe osteogenesis imperfecta (OI) results in complications, including multiple bony deformities, chronic pain and scoliosis. Formal diagnosis and treatment of rare conditions may not be available to everyone, leading to a complex and severe disease picture, often spanning multiple disciplines, when healthcare is finally accessed.

### Presenting Problem

A 9-year-old Kuwaiti female, presented soon after arriving in the UK, with short stature and multiple bony deformities due to OI. Parents who were distantly related, reported fractures from the neonatal period, which were managed conservatively without bisphosphonate therapy. She never had access to a wheelchair or attended school. Examination revealed typical facies of severe OI with dentinogenesis imperfecta, blue sclerae, frontal bossing, bilateral upper and lower limb deformities, pectus excavatum and scoliosis. Skeletal survey identified multi-level vertebrae compression fractures and significant long bone abnormalities consistent with OI. A chest x-ray revealed a cavitating lesion in the left lower lung field, with an adjacent well-circumscribed cystic nodule, despite no respiratory concerns other than a single episode of haemoptysis.

### Clinical Management

Hospital admission was arranged for multidisciplinary assessment and management. Bilateral pulmonary hydatid disease was confirmed with imaging and serology; interpreter-assisted history revealed exposure through her father, a sheep farmer in Kuwait, and prolonged ground contact. Medical and surgical options were considered, with risks including cyst rupture and anaphylaxis, fractures, and challenges protecting the cervical spine peri- and post-operatively. MRI brain showed significant platybasia with basilar invagination and foramen magnum narrowing. Bisphosphonates were initially withheld, due to the risk of acute phase reaction and respiratory distress. Following discussions between Endocrinology, Infectious Diseases, Respiratory, Anaesthetics and Interventional Radiology, plus national parasitology MDT and Highly Specialised Service for OI input, the largest cyst was successfully drained under sedation, and oral Praziquantel and Albendazole were commenced. She has subsequently started bisphosphonate treatment, been assessed by dental and orthopaedic services, and referred to neurosurgeons.

### Discussion

This case highlights the complexity of untreated severe OI associated with a second major medical problem, and the challenges of treatment decision-making in the context of bone fragility. Multidisciplinary involvement, while time-consuming, is essential to ensure patient safety and optimal outcome.

Figure 1 shows a variety of radiological images of the patient

Left femur (top left); Left knee (top right); Chest x-ray (bottom left); CT chest (bottom right)

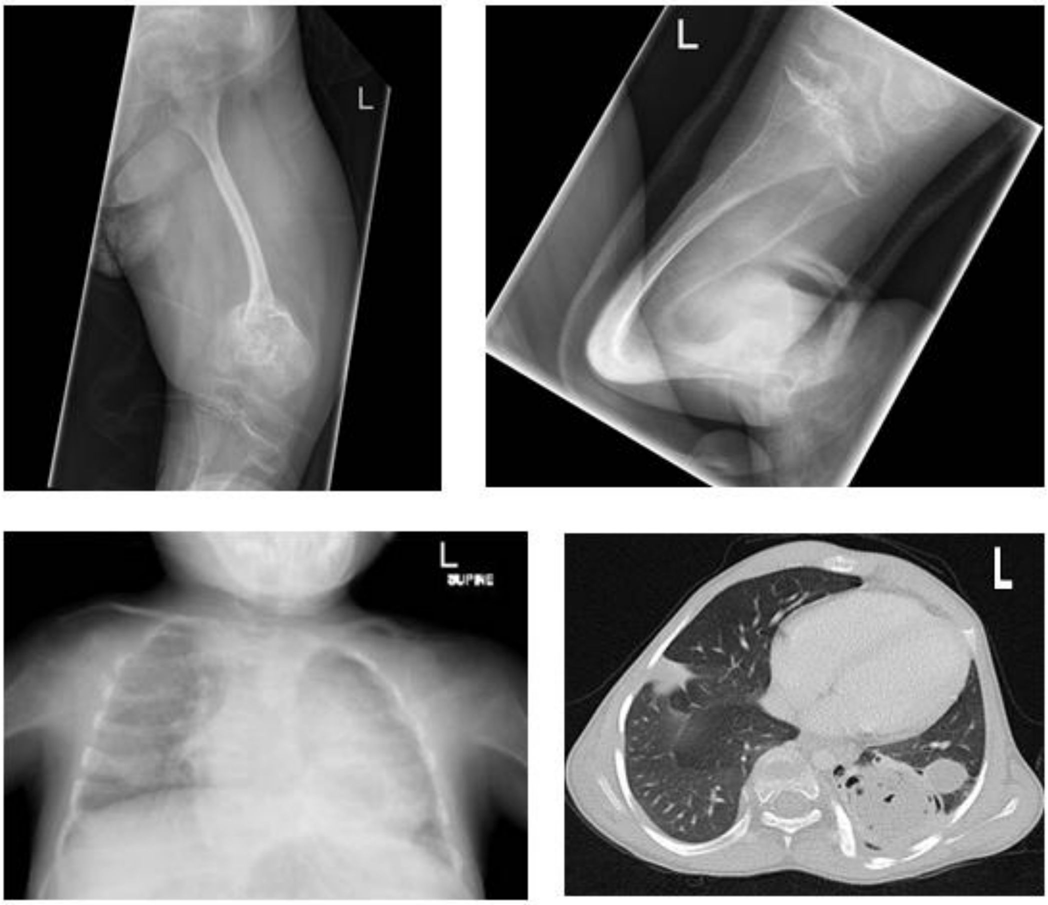

### Disclosure

None declared

### P143

### The Relationship Between Bone Mass and Fat Mass in Young Patients with Congenital Adrenal Hyperplasia: Does Puberty Play a Key Role?

Stefano Mora, Marianna R Stancampiano, Ruggero Lanzafame, Katia Maruca, Elisa Sala, Gianni Russo, Marco Pitea

IRCCS San Raffaele Hospital, Milano, Italy

### Abstract

OBJECTIVES:

Patients with congenital adrenal hyperplasia (CAH) due to 21-hydroxylase deficiency require lifelong glucocorticoid replacement therapy, which may adversely affect body composition and bone health. While the relationship between adiposity and bone mass is well described in the general population, its modulation across pubertal development in patients with CAH remains unclear. We therefore aimed to longitudinally evaluate the relationship between fat mass and bone mass in young patients with CAH, with particular attention to the role of puberty.

METHODS:

We studied 36 young patients with CAH due to 21-hydroxylase deficiency (17 girls and 19 boys) before puberty (median age 7.6 [4.8–10.8] years) and after completing pubertal development (18.3 [15.6–22.6] years). Whole body (WB) bone mass and body composition were assessed using dual-energy X-ray absorptiometry and compared with data from two age-matched healthy cohorts. Multiple regression models were employed to compare the groups and to establish relationships between bone and fat masses.

RESULTS:

WB fat mass (expressed as a percentage of total tissue mass) significantly increased in patients with CAH from prepuberty to postpuberty (0.28 to 0.30%, $p = 0.025$). While prepubertal patients showed fat and bone mass comparable to healthy controls, postpubertal patients exhibited higher fat mass (0.29% vs. 0.23%, respectively; $P = 0.04$). Multivariate analysis revealed no difference in WB bone mass between the two prepubertal groups. In contrast, a significant difference was observed among postpubertal subjects ($\beta = -133$; $P = 0.003$). The relationship between bone mass and fat mass changed after puberty. The relationship in prepubertal subjects was not statistically significant, while WB fat mass was inversely related to bone mass in postpubertal individuals ($\beta = -1893$; $P = 0.018$, Figure 1). No relationship with current glucocorticoid doses was found.

CONCLUSIONS:

Puberty represents a critical period during which increased adiposity negatively impacts bone mass in patients with CAH. These findings suggest that patients with CAH may be predisposed to osteoporosis later in life, highlighting the importance of monitoring bone health and implementing mitigation measures to prevent this occurrence.

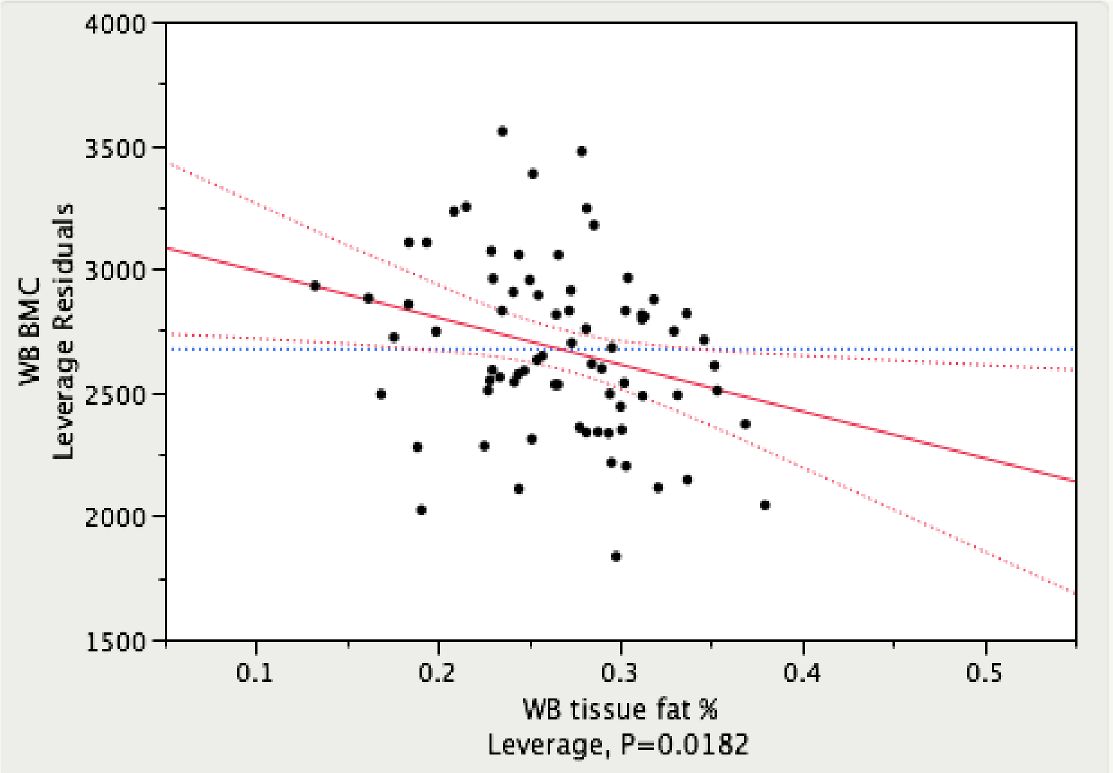

### Disclosure

None declared

### P144

### Clinical Implementation of Virtual Reality for Pain and Anxiety Management in a Pediatric Orthopedic Hospital

Tina Athanasoulias[1], Alexandra De Almeida Vicente[1], Sofia Addab[2], Kelly Thorstad[1], Reggie Hamdy[1], Gianluca Bertolizio[1], Argerie Tsimicalis[1,2]

[1]Shriners Hospitals for Children-Canada, Montreal, Canada. [2]McGill, Montreal, Canada

### Abstract

**Objectives:**

To integrate Virtual Reality (VR) as a therapeutic tool to reduce pain and anxiety in children across the entire care continuum at Shriners Hospitals for Children®-Canada. A multi-level, collaborative, quality improvement initiative will enable the effective implementation of VR from research into clinical practice.

**Methods:**

A multi-level implementation process was adopted, which included: creating an advisory council, understanding needs, pilot studies, market research, engaging patient and family partners, developing a policy and procedure, providing ongoing VR training, cultivating VR network, and sharing expertise via publications, consults, VR expositions, and podcast series on VR implementation, and welcoming research trainees.

**Results:**

The advisory council comprised clinicians, researchers, decision-makers, patients, and family partners. Market research guided the selection of the most appropriate VR equipment and company. Seven clinicians participated in training sessions over the year. A second VR exposition attracted 120 attendees, featured 12 companies, and showcased 16 research posters. Eight podcast episodes highlighted insights from 12 VR experts. Sixteen clinicians utilized VR in 216 documented sessions over the year. Three research studies were completed, with an additional three currently in progress.

**Conclusions:**

To ensure successful adoption of VR in clinical settings, it is important to develop a comprehensive implementation process that incorporates innovative, multi-modal approaches, with a strong focus on training, support, and sustainability. VR has demonstrated its value as a non-pharmacological tool for alleviating pain and managing anxiety in pediatric patients, and it is crucial to expand its application to a wider range of clinical settings, especially for neurodiverse populations.

### Disclosure

None declared.

### P145

### Craniofacial Phenotype and Effect of Sclerostin Inhibition in a Mouse Model of Severe Osteogenesis Imperfecta

Pasquale Mulé, Frank Rauch, Juliana Marulanda

Shriners Hospital for Children-Canada and McGill University, Montreal, QC, Canada

### Abstract

**OBJECTIVES:**

Osteogenesis imperfecta (OI) is associated with craniofacial abnormalities that can impair mastication, airway anatomy, and neurological function. Sclerostin inhibition is being investigated in clinical trials on children with OI, but the craniofacial effects of this treatment approach remain poorly characterized. This study examines the effects of genetic sclerostin inhibition in Jrt mice, a model of severe dominant OI.

**METHODS:**

Jrt mice were crossed with sclerostin knockout mice (*Sost*-KO) to produce sclerostin-deficient Jrt mice (OI;*Sost*-KO). Cephalometric analyses were performed using micro-computed tomography on heads from 8 and 14-week-old mice (N = 123; 6-8 mice per group) across four genotypes (WT, Jrt (OI), *Sost*-KO, and OI;*Sost*-KO). Analyses of the temporomandibular joint (TMJ), foramen magnum cross-sectional area, intracranial area, and cranial base angle were performed only at 14 weeks.

**RESULTS:**

At 8 weeks, OI crania were smaller than WT, including reduced cranial length (3.5%, $p = 0.01$), interzygomatic width (7.6%, $p < 0.001$), and mandibular length (7.6%, $p < 0.001$), with no effect of sclerostin inhibition. At 14 weeks, sclerostin inhibition normalized cranial length in OI mice, while transverse widths (interzygomatic 4.6% decrease, p < 0.001) and mandibular abnormalities persisted. In male OI mice, sclerostin inhibition was associated with localized overgrowth, including increased skull height (+5.0% vs WT, p = 0.02) and interorbital width (9.7%, p < 0.001).

OI mice had shorter mandibular condylar depth (13%, p < 0.001), which was normalized by sclerostin inhibition in females but not males, while TMJ space narrowing persisted. In female OI mice, sclerostin inhibition reduced foramen magnum area (4.7% vs WT, p = 0.04). Intracranial area increased with sclerostin inhibition in both sexes (6.6% vs WT, p = 0.02). Cranial base angle was reduced in male OI; *Sost*-KO mice compared to WT (2.3% , p = 0.008), with no difference in females.

**CONCLUSION:**

Sclerostin inhibition produced sex- and region-specific effects, characterized by localized overgrowth rather than uniform craniofacial restoration, including alterations in cranial base and intracranial architecture. Therefore, close monitoring of craniofacial growth appears prudent in studies assessing anti-sclerostin therapies in children with OI.

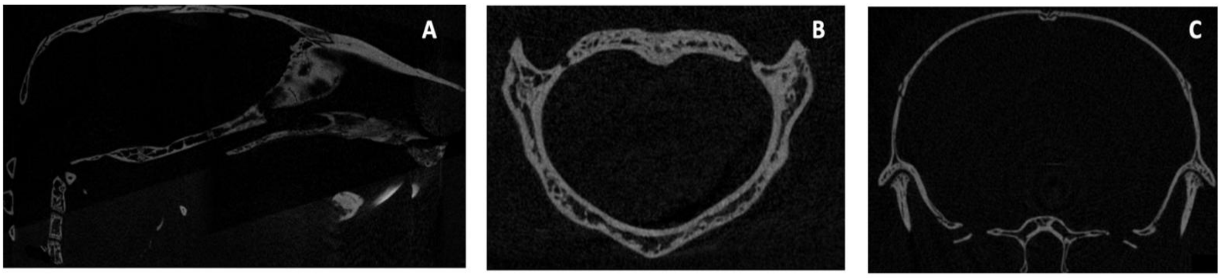

**Figure 1.** Micro-computed tomography (μCT) images of the mouse craniofacial skeleton showing sagittal orientation (A), axial slice at the level of the foramen magnum (B), and coronal slice used for intracranial area assessment (C).

**Disclosure**

None declared

### P146

### TCIRG1 Autosomal Recessive (AR) Infantile Malignant Osteopetrosis (IMO) Presenting with a Rare Feature of Subdural Hematomas and Osteopetrorickets with Restoration of Calcium Homeostasis after Hematopoietic Stem Cell Transplant (HSCT)

Poorvi Agrawal[1], Edward Ziga[1], Matthew Benson[1,2]

[1]Nemours Children's Health, Jacksonville, USA. [2]Mayo College of Medicine, Jacksonville, USA

### Abstract

**BACKGROUND**:

IMO secondary to bi-allelic pathogenic variants in T-Cell Immune Regulator 1 Gene (TCIRG1) accounts for ~50% of cases of AR osteopetrosis. The TCIRG1 gene produces two alternatively spliced protein products; OC116 is the a3 subunit of the V-ATPase proton pump (required for normal osteoclast function).

**PRESENTING PROBLEM**:

An 11-month-old boy of non-consanguineous parents presented at 2 months old with macrocephaly, failure to thrive, and no visual tracking. He presented at 3 months old with bilateral subdural hematomas, anemia (hemoglobin 8.1g/dL), thrombocytopenia ($51x10^3$ /µL), hepatosplenomegaly, and optic atrophy along with calcium x phosphorous product of 16.8 (typically <30 in IMO with osteopetrorickets). Skeletal survey revealed generalized sclerosis with florid rickets. Whole exome sequencing revealed bi-allelic previously known mutation of TCIRG1 (c.1674-1G>A) in the AG splice acceptor site, leading to a frameshift and no protein function. The calcium x phosphorous product nadir was 9.8. His alkaline phosphatase was markedly elevated (895 IU/L) with appropriate secondary hyperparathyroidism (PTH 383 pg/ml) with normal vitamin D level (25 OH Vitamin D 37 IU/L). Stabilization with IV calcium was required (~48 mg elemental calcium/kg/day), with enteral calcium carbonate (~95 mg elemental calcium/kg/day) and calcitriol.

**CLINICAL MANAGEMENT**:

He underwent matched-unrelated donor HSCT at 9 months old with successful engraftment. He continued to have persistent hypocalcemia for about two weeks post-HSCT. Gradually the requirement for calcitriol and calcium supplementation decreased. Six weeks post-HSCT he did not require calcium supplementation. Most recent calcium x phosphorous products were 60-70. Repeat imaging 8 weeks post-HSCT revealed early healing of osteopetrorickets.

**DISCUSSION**:

Our case highlights important considerations: 1) Infants with IMO should be evaluated early in life for the presence of osteopetrorickets and bleeding diathesis. 2) Adequate treatment with calcium and calcitriol to augment maximal calcium absorption from ileum and intestines is necessary. This will increase serum calcium and lower PTH, thereby decreasing urinary phosphorous excretion. 3) It is imperative to manage secondary hyperparathyroidism and osteopetrorickets adequately prior to HSCT to increase the chances of successful restoration of calcium homeostasis afterwards. 4. HSCT is currently the only treatment modality available, though it cannot reverse vision loss.

### Disclosure

None

### P147

### Severe Rickets in a Child with Autosomal Dominant Hypophosphatemia Combined with Sickle Cell Disease

Stephanie Vermeulen[1], Martine T P Besouw[2], Annemieke M Boot[1]

[1]Beatrix Children's Hospital, Department of Pediatric Endocrinology, University Medical Center Groningen, University of Groningen, Groningen, Netherlands. [2]Beatrix Children's Hospital, Department of Pediatric Nephrology, University Medical Center Groningen, University of Groningen, Groningen, Netherlands

### Abstract

**BACKGROUND:**

Autosomal dominant hypophosphatemic rickets (ADHR) is an extremely rare disorder caused by an activating pathological variant in the *FGF23* gene, resulting in failure of FGF23 cleavage in case of increased transcription. It is considered a two-hit disease that requires both an *FGF23*-activating variant and an FGF23-producing trigger like iron deficiency (which is known to stimulate *FGF23* expression). Less well known is that erythropoietin can also increase *FGF23* expression. The age of onset and phenotype of ADHR can be variable.

**CASE PRESENTATION**A

Two years old girl with a history of sickle cell anemia was referred because of severe rickets despite conventional treatment. Genetic testing showed a pathogenic variant in the *FGF23* gene (Arg179Gln), confirming the diagnosis of ADHR. After starting to walk at the age of 18 months, at presentation to our clinic she was no longer able to walk because of severe bone pain. She had lower extremity bone deformities and growth retardation. Laboratory findings showed low serum phosphate (0.46 mmol/L; 1.42 mg/dL), high alkaline phosphatase (1077 U/L), normal serum calcium (2.33 mmol/L; 9.34 mg/dL), high PTH (12.9 pmol/L; 121 pg/mL), low 25(OH)vitD3 (29 nmol/L; 11.6 ng/mL) and high FGF 23 (634 U/mL; 634 pg/mL). Hb was low (4.9 mmol/L; 7.9 g/dL) with normal ferritin (32 µg/L) and MCV (89 fL). On X-ray she had evident signs of rickets. Unfortunately, no urinary sample could be provided.

**CLINICAL MANAGEMENT**

After six months of treatment with oral phosphate, alfacalcidol and vitamin D3 supplementation, her symptoms did not improve and she was referred to our center. We switched her treatment to Burosumab (anti-FGF23 humanized antibody, off-label). Within the next six months her symptoms improved; she started walking again and did not complain of pain anymore; her laboratory results also improved markedly.

**DISCUSSION**

To our knowledge this is the first case with ADHR combined with sickle cell anemia, treated with Burosumab. Our patient had severe rickets, but without iron deficiency. We hypothesize that chronically high erythropoietin levels are responsible for increased FGF23 production, causing the severe phenotype. Treatment with the anti-FGF23 antibody Burosumab resulted in a dramatic improvement of her symptoms.

### Disclosure

None declared.

### P148

### Healthcare Transition in X-linked hypophosphataemia in Australia and New Zealand: Use of a Modified Delphi Approach to Create a Local Consensus Statement

Jessica Sandy[1,2], Peter Simm[3], Craig Munns[4], Maria Craig[2], Andrew Biggin[5], Lara Graves[2], Christine Rodda[6], Craig Jefferies[7], Christian Giurgis[8], Mary White[3], Sherel Levy[9], Verity Pacey[9], Shejil Kumar[8], Selma Torronen[10], Syndia Lazarus[11], Angela Sheu[12], Myra Poon[2], Ben Wheeler[13], Jenny Harrington[10], Lucy Collins[14], Sandy Bevc[15], Naomi Ford[15], Roderick Clifton-Bligh[16], Aris Siafarikas[17]

[1]The University of Sydney, Sydney, Australia. [2]The Children's Hospital at Westmead, Sydney, Australia. [3]Royal Children's Hospital, Melbourne, Australia. [4]University of Queensland, Brisbane, Australia. [5]Queensland Children's Hospital, Brisbane, Australia. [6]University of Melbourne, Melbourne, Australia. [7]The University of Aukland, Aukland, New Zealand. [8]Westmead Hospital, Sydney, Australia. [9]Macquarie University, Sydney, Australia. [10]Women and Children's Hospital, Adelaide, Australia. [11]Royal Brisbane and Women's Hospital, Brisbane, Australia. [12]St Vincents Hospital, Sydney, Australia. [13]University of Otago, Dunedin, New Zealand. [14]Monash Hospital, Melbourne, Australia. [15]XLH Australia, Sydney, Australia. [16]Royal North Shore Hospital, Sydney, Australia. [17]Perth Children's Hospital, Perth, Australia

### Abstract

**Introduction:**

Healthcare transition (HCT) is the purposeful planned movement of adolescents and young adults (AYAs) with chronic diseases from child-centred to adult-oriented healthcare systems[1]. In X-linked hypophosphataemia (XLH), adolescence is a vulnerable time with affected individuals often experiencing both paediatric and adult physical manifestations and a high psychological burden of disease. This is also the age when AYAs are establishing independence and navigating significant life changes.

XLH Australia has recently declared healthcare transition as a key area of importance. Guidelines on management of XLH suggest the importance of optimising healthcare transition[2], but there are no local, specific guidelines to support Australian healthcare professionals managing young people with XLH optimise the healthcare transition process.

**Objectives:**

We aimed to develop a consensus guideline for healthcare transition for AYAs with X-linked hypophosphataemia (XLH) in Australia and New Zealand based on expert consensus.

**Method:**

Modified Delphi study design was used.

1. Panel of experts (including patient representation, paediatric and adult specialists, and allied health professionals) formed from invitations sent through professional organisations.
2. Panel completed modified Delphi surveys with specific questions based on initial literature review.
3. Controlled feedback was presented with primary investigator at online meetings in between survey rounds.
4. Number of survey rounds determined by consensus and stability of responses.
5. Consensus was defined as agreement >70%

**Results:**

26 experts were involved. 46% (n=12) were paediatric endocrinologists, 23% (n=6) adult endocrinologists, 15% (n=4) were consumers (AYAs with XLH or parents/guardians), two physiotherapists, one general practitioner, and one paediatric nephrologist. Mean(SD) time working with people with bone and mineral disorders was 15(11) years. Table shows a summary of the key statements that reached consensus.

**Conclusions:**

Healthcare transition in XLH is a complex process which requires holistic, multidisciplinary management and careful coordination. Most experts agree that a joint transition clinic and a transition coordinator are valuable in the HCT process, although the lack of availability of such resources is a key limitation for clinicians striving to improve HCT in XLH. These data also suggest a need for the creation of locally specific documents and resources to assist in the HCT process.

| **Table: Summary of Key Statements that reached consensus (agreement >70%) during Modified Delphi study on Healthcare Transition in XLH (Australia/New Zealand)** |
|---|
| **Timing and preparation for healthcare transition** |
| The topic of healthcare transition should first be discussed with a young person and their family at least two years before transfer of care to adult services, with ideal time for transfer usually occurring between 16 to 19 years |
| "Transition readiness" should be assessed primarily via a discussion with the young person and their paediatric clinician, but transition readiness questionnaires may be useful |
| **Strategies to engage and care for adolescents and young adults with XLH** |
| The use of psychological and quality of life questionnaires may be useful |
| During paediatric consultations with AYAs with XLH, paediatric clinicians should make an effort to talk directly to the young person themselves, rather than parents or carers. |
| Text message appointment reminders as well as individual and/or after school/work appointments may be beneficial |
| Telehealth medicine should have a role in managing young people with XLH but should not replace face to face review |
| **Joint transition clinics** |
| A joint transition clinic with adult and paediatric clinicians, as well as transition coordinator if available, present is recommended/beneficial in the process of healthcare transition for XLH |
| Telehealth joint transition clinics (where one or all parties join via telehealth) are an alternative when a joint face-to-face clinic is not possible or practical |
| If joint transition clinics are not possible, after the last paediatric appointment, the paediatric team or transition coordinator should follow-up with the young person to ensure that they attended their appointment with the adult clinician and assess if there were any issues. This follow-up could be a phone call, telehealth appointment, or scheduled clinic appointment. |

| |
|---|
| **Transition coordinator** |
| A transition coordinator, if available, is recommended to assist in the healthcare transition process |
| Transition coordinator role should or may include:<br>- Booking appointments and reminding the young person<br>- Being a point of contact with the young person and their family<br>- Advising parents how best to support and empower their adolescent to navigate the adult health system<br>- Educating the adolescent on navigating adult healthcare systems<br>- To assess efficacy of transition through assessing engagement in adult services<br>- To assess efficacy of transition through questionnaires or satisfaction surveys<br>- To empower the young person in managing their disease |
| **Genetic counselling and burosumab safety in pregnancy** |
| Genetic counselling regarding inheritance of XLH and burosumab safety in pregnancy and risk of young person's offspring inheriting XLH should be offered at the time of healthcare transition |
| **HCT and XLH resources** |
| Locally created resources that should be provided to the young person and family include:<br>- A contact card with healthcare professional contacts<br>- Transition checklist<br>- Updated medical summary/letter<br>- Information on healthcare transition<br>- Information on navigating adult services<br>- Information on XLH<br>- Frequency of investigations<br>- Details on relevant societies and patient groups<br>- Information on treatments |
| **Clinical work-up before transfer of care** |
| Prior to transfer to adult care, the following work up should be routinely completed:<br>- Full history and examination to assess symptomatology and lower limb deformity<br>- Biochemistry including PTH, vitamin D, and urinary calcium:creatinine ratio, TmP/GFR<br>- Renal ultrasound<br>- Dental assessment, including dental X-rays if clinically indicated<br>- Quality of life questionnaires |

| |
|---|
| Prior to transfer to adult care, the following work up should be considered on an individual basis:<br>- X-rays to assess leg deformity or rickets<br>- Psychological assessment<br>- Quality of life questionnaire<br>- Functional assessment such as 6MWT<br>- 1,25 vitamin D |
| **Multidisciplinary management team and preferred level of expertise** |
| The primary treating specialist managing young people with XLH should ideally be a specialist bone clinician with experience in managing XLH or other primary bone disorders |
| If a specialist bone clinician is not available, then the patient should be managed by a general endocrinologist, nephrologist or rheumatologist with an interest in bone disorders |
| In the above scenario, joint telehealth or shared care arrangements with specialist bone clinicians may be beneficial. |
| The following healthcare professionals should be involved in care and have experience in managing primary bone disorders:<br>- Orthopaedic surgeon<br>- Dentist<br>- with experience in managing XLH or primary bone disorders should be involved in the care of young people with XLH |
| The following healthcare professionas should be involved in care, preferably with experience in primary bone disorders:<br>- Psychologist<br>- Physiotherapist<br>- Occupational therapist |
| A general practitioner should also be involved in care and does not require experience in managing primary bone disorders. |

References:

1. Robert W. M. Blum, Dale Garell, Christopher H. Hodgman, Timothy W. Jorissen, Nancy A. Okinow, Donald P. Orr, et al. Transition from child-centered to adult health-care systems for adolescents with chronic conditions: A position paper of the Society for Adolescent Medicine. Journal of Adolescent Health. 1993;14(7):570-6.

2. J. L. Sandy, P. J. Simm, A. Biggin, C. P. Rodda, C. L. Wall, A. Siafarikas, et al. Clinical practice guidelines for paediatric X-linked hypophosphataemia in the era of burosumab. J Paediatr Child Health. 2022;58(5):762-8.

### Disclosure

This project is funded by an investigator initiated grant from Kyowa Kirin

### P149

### From Collagen Mutation to Nerve Dysfunction: Investigating the Structural Role of Collagen in the Peripheral Nerve in the Context of Osteogenesis Imperfecta

Lander Manrique[1], Abhimanyu Mertiya[1], Sarah Ford[1], Mariia Mielkozorova[2,1], Bettina Willie[1], Eric Schaible[3], Emerson Krock[1], Elizabeth Zimmermann[1]

[1]McGill University, Montreal, Canada. [2]University of Alberta, Edmonton, Canada. [3]Lawrence Berkeley National Laboratory, Berkeley, USA

### Abstract

**OBJECTIVES:**

Osteogenesis imperfecta (OI) is a connective tissue disorder caused by COL1A1 or COL1A2 mutations, characterized by skeletal dysplasia, chronic pain, and sensitivity. Behaviors related to chronic pain have been reported in the *Col1a1*$^{Jrt/+}$ (JRT) murine model of OI. It is unknown how reduced collagen or altered collagen assembly (a trait of the JRT model) impacts nerves, which rely on collagen to provide structural stability. **The objective of the study** was to elucidate the role of OI-related collagen alterations on structural and multiscale mechanical properties of peripheral nerves.

**METHODS:**

Sciatic nerves were dissected from 16-17-week-old male and female JRT on a FVB background and wild-type littermates (WT). Tissue scale (millimeter scale) and collagen fibril scale (10-100 nanometers) mechanical characterization were carried out using synchrotron small-angle X-ray scattering (SAXS) (n = 5-7 mice/genotype). Tissue structure was characterized by second harmonic generation (SHG) microscopy (n=3 mice/genotype) and immunohistochemistry staining (IHS).

**RESULTS:**

Sciatic nerves from JRT mice were significantly weaker than controls in tension (Figure 1A). JRT male and female nerves had a 41-46% lower tensile strength than male WT group (Figure 1B); male JRT nerves had a significantly lower Young's modulus than WT (Figure 1C). SHG scans qualitatively show thinner and less collagen fibers inside the sciatic nerves of male JRT mice (Figure D-E). SAXS experiments measuring collagen fibril deformation during tensile testing of whole nerve showed that JRT nerves exhibited a higher level of non-uniform collagen deformation compared to WT. During mechanical loading, collagen and myelin orient towards the tensile stress (termed recruitment) in the WT, but are less efficiently recruited in the JRT mice. SAXS diffraction peak amplitudes showed that affected male nerves had significantly less collagen and myelin at the nanoscale ECM compared to WT; this difference was not found for the female JRT mice (Figure 1 F-G).

**CONCLUSIONS:**

Our results show that the structural integrity of the sciatic nerves of the murine OI model are compromised and sex dependent. Further studies are required to understand the link between collagen's role in providing structural integrity to the nerve and neuropathic behavior in OI.

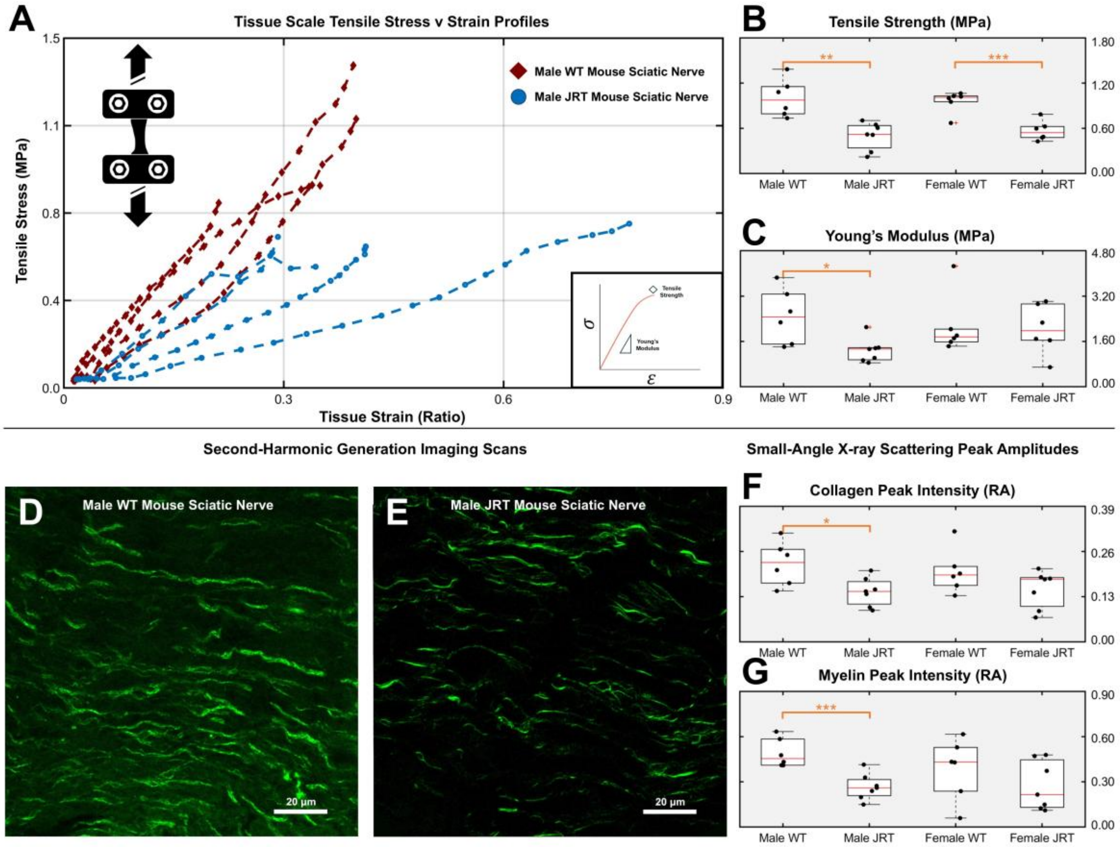

**Figure 1: Mechanical and Structural Characterization of healthy (WT) and affected (JRT) mouse sciatic nerves.** A) Tissue scale stress strain profiles of male WT and JRT sciatic nerves. B) Tensile strength measurements for all groups. C) Young's Modulus measurements for all groups. D-E) SHG scans of collagen fibers inside sciatic nerves of WT and JRT male mice. F-G) Collagen and myelin periodical structure signal intensity counts measured with SAXS.

### Disclosure

None declared

### P150

### Peripheral Quantitative Computed Tomography in Children With X-Linked Hypophosphataemia on Burosumab Therapy

Jessica Sandy[1,2], Aris Siafarikas[3], Sushil Yewale[2], Andrew Biggin[4], Craig Munns[5], Maria Craig[2], Christine Rodda[6], Peter Simm[7], Christie-Lee Wall[2], Julie Briody[2]

[1]University of Sydney, Sydney, Australia. [2]The Children's Hospital at Westmead, Sydney, Australia. [3]Perth Children's Hospital, Perth, Australia. [4]Queensland Children's Hospital, Queensland, Australia. [5]University of Queensland, Brisbane, Australia. [6]University of Melbourne, Melbourne, Australia. [7]Royal Children's Hospital, Melbourne, Australia

### Abstract

### Introduction

In X-linked hypophosphataemia (XLH), excess fibroblast growth factor 23 (FGF23) leads to hypophosphataemia, rickets and osteomalacia. Burosumab, FGF23 monoclonal antibody, improves musculoskeletal features. The effect on bone mineral density (BMD) by XLH and burosumab in children is incompletely understood.

### Methods

Retrospective review of peripheral quantitative computed tomography (pQCT) in children with XLH across two Australian centres. Baseline data were included if within 3 months of starting burosumab and follow-up data were after at least 12months.

Age adjusted Z-scores based on reference populations at tibia and radius were reviewed for: volumetric BMD (vBMD) trabecular, vBMD cortical, cortical thickness, muscle cross-sectional area (CSA), and stress strain index(SSI).

Normality testing was performed with non-parametric data presented as median[interquartile range, IQR]. Wilcoxon Signed Ranks and Sign tests were used to compare baseline and follow-up data.

### Results

Twelve children were included. Median[IQR] age at burosumab commencement was 8.50[6.06-11.63]years. Duration of burosumab at follow-up was 12-35 months (median[IQR]=20.50[12.00-28.50]). Data summarised in table.

Baseline radius trabecular vBMD Z-scores were all above zero. Tibia trabecular vBMD ranged from -4.7 to +5.7. Cortical vBMD of radius and tibia was lower than zero, and lower than trabecular vBMD ($p<0.01$). There was no significant change over time in trabecular or cortical vBMD.

Cortical thickness and muscle CSA data are available for seven tibia and six radius scans. Baseline median cortical thickness for tibia and radius Z-scores were lower than zero (-1.50[-2.1 to -0.70], -0.55[-1.35 to +0.13] respectively) with no change over time.

At baseline, muscle CSA Z-scores were below zero (tibia: -0.60[-0.60 to -0.30], radius: -1.35[-2.30 to -0.83], with improvement seen in radius CSA at follow-up (increase of +0.3, $p=0.03$).

### Discussion/Conclusion

In this cohort of children with XLH, cortical vBMD, cortical thickness and muscle CSA were lower than predicted. Trabecular vBMD was higher than cortical vBMD, and higher in radius than tibia. There was no change seen in vBMD over time with burosumab, although an increase was noted in radius muscle size.

More research is needed with larger cohorts over longer time periods to assess the effect of burosumab on BMD in paediatric XLH, with further exploration of bone microarchitecture and muscle size and composition.

**Table: Peripheral quantitative computed tomography data in children with X-linked hypophosphataemia: comparing results before and 1-3 years post burosumab**

| | Baseline | Follow-up | P value | |
|---|---|---|---|---|
| **Tibia (n=12)** | | | | |
| Trabecular vBMD | -0.70[-1.6 to 0.90] | -0.30[-1.20 to 1.75] | 0.45 | |
| Cortical vBMD | -1.90[-3.60 to -0.95] | -2.20[-3.68 to -0.45] | 0.53 | |
| Cortical thickness(n=7) | -1.50[-2.1 to -0.70] | -1.30[-1.50 to -0.60] | 0.35 | |
| Muscle CSA(n=7) | -0.60[-0.60 to -0.30] | -0.50[-0.60 to 0.20] | 0.52 | |
| Stress Strain Index | 0.25[-0.55 to 2.38] | 0.60[-0.35 to 2.83] | 0.12 | |
| **Radius (n=11)** | | | | |
| Trabecular vBMD | 1.10[0.50 to 2.50] | 1.10[0.00 to 1.3 ] | 0.33 | |
| Cortical vBMD | -2.20[-4.20 to -1.40] | -2.60[-3.90 to -1.80] | 0.93 | |
| Cortical thickness(n=6) | -0.55[-1.35 to +0.13] | -0.25[-1.63 to 0.25] | 0.75 | |
| Muscle CSA(n=6) | -1.35[-2.30 to -0.83] | -1.05[-1.88 to 0.0] | **0.03** | |
| Stress Strain Index | -0.70[-1.00 to 0.40] | 0.20[-0.70 to 0.80] | 0.24 | |

**Data presented as median [IQR]**

### Disclosure

JS, AB, CM, PS, CR, AS have participated in research funded by Kyowa Kirin

### P151

### Bone Mineral Density Measured by Dual-Energy X-ray Absorptiometry in Children with X-linked Hypophosphataemia on Burosumab Therapy

Jessica Sandy[1], Sushil Yewale[1], Maria Craig[1], Craig Munns[2], Peter Simm[3], Andrew Biggin[4], Christine Rodda[5], Aris Siafarikas[6], Christie-Lee Wall[1], Julie Briody[1]

[1]The Children's Hospital at Westmead, Sydney, Australia. [2]University of Queensland, Brisbane, Australia. [3]Royal Children's Hospital, Melbourne, Australia. [4]Queensland Children's Hospital, Brisbane, Australia. [5]University of Melbourne, Melbourne, Australia. [6]Perth Children's Hospital, Perth, Australia

### Abstract

**Introduction:** In X-linked hypophosphataemia (XLH), hypophosphataemia caused by excess fibroblast growth factor 23 (FGF23) leads to rickets and osteomalacia. The effect of burosumab, an FGF-23 monoclonal antibody, on bone mineral density (BMD) in children with XLH has not been described.

**Methods:** Areal BMD data from a cohort of Australian children with XLH were reviewed from dual-energy X-ray absorptiometry scan (Lunar iDXA) prior to starting burosumab and at follow-up.

Height adjusted Z-scores (HAZ) and age adjusted Z-scores (AAZ) were calculated based on locally collected reference data for: Total BMD (including head), right femoral neck, Lumbar spine (L2-L4), arm and leg BMD and bone mineral content for lean tissue mass (BMC for LTM) Z-scores.

Normality testing was performed. Paired t-tests and Wilcoxon sign rank tests were used to compare baseline and follow-up data.

**Results:** Nine children were included. Age at burosumab commencement was 6.60(0.85)years. Follow-up time was 41 to 80months. Height Z-scores (mean(SD)) were -1.93(0.65) at baseline, -1.61(0.68) at two years, and -1.28(0.90) at most recent follow-up.

Mean baseline total BMD Z-scores were above zero (+0) with an upwards trend that was not significant ($p$=0.27).

Mean femoral neck Z-scores were above zero (+0) and increased with time ($p$=0.03).

Baseline lumbar spine BMD for all children were high (HAZ >+2.5, AAZ >+1.5) but there was no significant change over time($p$=0.29,0.91). Lumbar spine BMD was higher than Total and femoral neck($p$<0.001).

At baseline, arm HAZ was lower than total BMD HAZ ($p$=0.04). There were upward trends in arm and leg BMD Z-scores($p$=0.06-0.19).

Mean BMC for LTM Z-score was higher than population mean (+1.42(1.63)) and increased over time (+2.66(1.55),$p$=0.04).

**Discussion/Conclusions:** In a cohort of children with XLH, BMD was higher than reference population. BMD was highest at L2-L4 and lowest in arms. Burosumab therapy was associated with an upwards trend in BMD in all body sites except for lumbar spine; but only significant at the femoral neck. BMC for LTM, a marker of bone mass relative to muscle mass, was high at baseline and increased with burosumab. Further research is required to better understand the impact of XLH and burosumab on BMD.

**Table: Bone mineral density height and age adjusted Z-scores of children with X-linked hypophosphataemia at baseline and years after starting burosumab therapy**

| | Height adjusted z scores (units SD) | | | Age adjusted z scores (units SD) | | |
|---|---|---|---|---|---|---|
| Total BMD | Mean(SD) | Range | P value | Mean(SD) | Range | P value |
| Baseline | +1.41(0.93) | 0.0 to +2.6 | | +0.70(0.41) | -0.1 to +1.1 | |
| 1 year | +1.63(0.71) | +0.7 to +2.6 | 0.47 | +1.12(0.92) | -0.1 to +2.9 | 0.22 |
| 2 years | +1.63(1.10) | +0.1 to +3.1 | 0.68 | +1.12(1.19) | -0.4 to +3.5 | 0.33 |
| Most recent* | +2.20(1.18) | +0.2 to +3.8 | 0.19 | +1.19(1.07) | -0.3 to +2.8 | 0.27 |
| Femoral Neck | Mean(SD) | Range | P value | Mean(SD) | Range | P value |
| Baseline | +1.28(1.21) | -0.5 to +3.8 | | +0.56(1.06) | -1.4 to +2.6 | |
| 1 year | +2.07(1.15) | +0.7 to +3.9 | **0.05** | +1.27(1.18) | +0.1 to +2.9 | 0.07 |
| 2 years | +2.39(1.95) | -0.3 to +4.5 | 0.19 | +1.37(1.78) | -0.5 to +3.7 | 0.18 |
| Most recent* | +2.84(1.83) | +0.5 to +5.4 | **0.02** | +1.80(1.58) | +0.2 to +4.2 | **0.03** |
| LS Spine | Mean(SD) | Range | P value | Mean(SD) | Range | P value |
| Baseline | +4.08(0.99) | +2.7 to +5.6 | | +2.49(0.81) | +1.3 to +3.5 | |
| 1 year | +3.96(0.96) | +2.7 to +5.6 | 0.70 | +2.88(0.91) | +1.4 to +4.6 | 0.39 |
| 2 years | +3.16(1.25) | +1.3 to +4.9 | 0.26 | +2.57(1.21) | +1.1 to +4.7 | 0.89 |
| Most recent* | +3.51(1.43) | +1.2 to +5.7 | 0.29 | ++2.43(1.0) | +0.9 to +3.9 | 0.91 |
| Arms | Mean(SD) | Range | P value | Mean(SD) | Range | P |

| | | | | | | |
|---|---|---|---|---|---|---|
| | | | | | | value |
| Baseline | +0.61(0.70) | -0.5 to +1.7 | | -0.16(0.90) | -1.1 to +1.9 | |
| 1 year | +1.0(1.0) | -0.2 to +2.4 | 0.21 | 0.39(1.30) | -1.2 to +3.1 | 0.10 |
| 2 years | +1.10(1.66) | -0.5 to +4.6 | 0.33 | 0.59(1.91) | -1.4 to +5.0 | 0.10 |
| Most recent* | +1.70(1.58) | -0.9 to +4.9 | 0.06 | 0.79(1.65) | -1.4 to +4.2 | 0.07 |
| Legs | Mean(SD) | Range | P value | Mean(SD) | Range | P value |
| Baseline | +1.28(1.37) | -0.9 to +3.1 | | -0.28(1.11) | -2.7 to +1.1 | |
| 1 year | +1.60(0.93) | +0.1 to +3.2 | 0.43 | +0.21(1.0) | -0.9 to +2.0 | 0.24 |
| 2 years | +1.60(1.12) | +0.2 to +3.5 | 0.58 | +0.21(1.08) | -1.5 to +2.2 | 0.34 |
| Most recent* | +2.01(1.14) | +0.4 to +3.6 | 0.19 | +0.57(0.79) | -0.5 to +1.8 | 0.10 |
| Bone Mineral Content for Lean Tissue Mass | | | | | | |
| Z scores | Mean(SD) | Range | P value | | | |
| Baseline | +1.42(1.63) | -0.7 to +3.8 | | | | |
| 1 year | +2.02(1.15) | +0.6 to +4.6 | 0.10 | | | |
| 2 years | +2.11(0.93) | +0.7 to +4.0 | 0.22 | | | |
| Most recent | +2.66(1.55) | +1.3 to +5.7 | **0.04** | | | |

*Most recent follow-up = 41 to 80 months

### Disclosure

JS CM CR PS AS AB have all been previously involved in research funded by Kyowa Kirin

### P152

### Maternally Inherited Large GNAS Cluster Deletion Causing Pseudohypoparathyroidism Type 1A and Gastrointestinal Issues in Siblings

Aikaterini Perogiannaki[1,2], Claire Hughes[2], Louise Wilson[3], Evelien Gevers[1,2]

[1]Centre for Endocrinology, William Harvey Research Centre, Barts and the London Medical School, Queen Mary University of London, London, UK. [2]Department of Paediatric Endocrinology, Royal London Hospital, Barts Health NHS Trust, London, UK. [3]Department of Genetics, Great Ormond Street Hospital, London, UK

### Abstract

**BACKGROUND:** Pseudohypoparathyroidism type 1A(PHP1A) is a rare imprinting disorder caused by maternally inherited pathogenic *GNAS* variants. *GNAS* locus is complex with several transcripts and tissue specific imprinting; Gsa in renal tubules, thyroid and pituitary is maternally expressed. Whole *GNAS* cluster deletion is uncommon and may cause additional features depending on genes involved. We describe two siblings with maternally inherited 20q13.32–q13.33 deletion encompassing the entire *GNAS* cluster.

**PRESENTING PROBLEM:** The proband(P1), born at 33+4 weeks (1985g, -0.4SD), had chronic lung disease, laryngomalacia, constipation, feeding difficulties and congenital hypothyroidism. At 4 months, chest X-ray for respiratory symptoms showed posterolateral rib fractures, prompting safeguarding evaluation. He was short with relative macrocephaly, small fontanelle and brachydactyly. Skeletal survey confirmed rib fractures without additional injuries but was suggestive of Albright Hereditary Osteodystrophy. PTH was raised(12.6pmol/l) with borderline low calcium(2.19mmol/l), normal phosphate, ALP, 25- and 1,25-Vitamin D.

His brother(P2) had Hirschsprung disease and mild TSH elevation from infancy. Following P1's diagnosis, he was found to have hypocalcaemia (2.02mmol/l) with elevated PTH (32pmol/l), high phosphate (2.25mmol/l) and insufficient Vitamin D.

**CLINICAL MANAGEMENT:** Microarray showed maternally inherited ~1.68Mb deletion including the whole *GNAS* cluster (*GNAS1* and *STX16*) with loss of methylation at all four *GNAS* differentially methylated regions. The deletion contains 16 protein-coding genes, including *EDN3*, associated with Hirschsprung disease.

P1 had transient mild hypocalcaemia with intermittent PTH elevation. P2 demonstrated PTH resistance (calcium 1.95–2.02mmol/L, phosphate 2.25–2.52mmol/L, PTH 32–35pmol/L) and commenced alfacalcidol, vitamin D and levothyroxine. Both siblings had hyperphagia and extreme early-onset obesity, improving with calorie restriction.

The mother has short stature(141cm), learning disability, obesity/metabolic disease/gestational diabetes, Graves' disease followed by hypothyroidism, and gastroparesis, without PTH resistance, consistent with pseudopseudohypoparathyroidism(PPHP).

**DISCUSSION:** We report a very rare maternally inherited large *GNAS* cluster deletion causing PHP1A in the offsprings and PPHP in the mother, with Hirschsprung syndrome formally diagnosed in one sibling. Two previous reports describe similar deletions and with phenotypes of PHP1A/PPHP, but no Hirschsprung disease, which may be underrecognised. Fractures have not been described before and may be unrelated. This case highlights the importance of considering PHP1A in infants with congenital hypothyroidism.

### Disclosure

None declared

### P153

### A Comparative Analysis of Child- and Parent-Reported Quality of Life: Hypochondroplasia and Achondroplasia

Tommaso Toschetti[1], Anita Freeman[1], Emilie Hupin[1], Catherine Ali[1], Moira S Cheung[2,1]

[1]Great Ormond Street Hospital for Children, NHS foundation trust, London, UK. [2]NIHR Great Ormond Street Hospital Biomedical Research Centre, London, UK

### Abstract

### Objectives

Achondroplasia (ACH) and hypochondroplasia (HCH) and are allelic skeletal dysplasias with HCH often dismissed as a milder version of ACH.  There is limited literature on the quality of life (QoL) in HCH and no comparative data between these conditions. We hypothesised that despite significant height discrepancies, HCH may have similar compromise of height related QoL to ACH. To test this, we utilized the QoLISSY Brief, to explore domain-specific patterns and discrepancies between child- and parent-reported perspective.

### Methods

We conducted a monocentric cross-sectional study including children and adolescents with HCH and ACH attending a tertiary skeletal dysplasia clinic. Seven children with HCH (3 males, 4 females) and ten with ACH (4 males, 6 females), aged 8–18 years, were included. One child with HCH did not complete the questionnaire due to emotional discomfort discussing quality-of-life issues. Both child- and parent-reported QoLISSY Brief questionnaires were analysed. Statistical analyses used non-parametric tests, appropriate for paired and independent samples.

### Results

Domain-level analyses showed a consistent pattern across both conditions, with higher scores in the social domain, suggesting preserved social functioning despite overall reduced quality of life. Paired analyses did not identify statistically significant differences between child- and parent-reported total scores within either condition; however, parents—particularly of children with achondroplasia—tended to report lower quality-of-life scores. In between-group analyses, child-reported total scores did not differ between HCH and ACH. In contrast, parent-reported total scores were significantly lower in ACH compared with HCH ($p = 0.030$), with moderate-to-large effect sizes. Median total scores were consistently around or below the mid-range of the 0–100 scale, indicating a substantial overall impact on quality of life across both conditions. Detailed item- and domain-level results are reported in the accompanying table.

### Conclusions

Overall quality of life appears substantially reduced in both hypochondroplasia and achondroplasia, with median total scores lying around the midpoint of the QoLISSY scale despite preserved social functioning. While children’s self-reported QoL was comparable between conditions, parents—particularly of children with achondroplasia—perceived a greater burden. These findings extend the QoLISSY literature and highlight the importance of including both child and parent perspectives. Although short stature is less pronounced in hypochondroplasia, a comparable impairment in health-related quality of life emerges. Larger studies are needed to confirm these findings and further characterise condition- and domain-specific differences.

| Item | Short label | H – Child | H – Parent | A – Child | A – Parent | p (H vs A) |
|---|---|---|---|---|---|---|
| q1 | Limitations in activities | 3.5 (3.0–4.0) | 3.5 (3.0–4.0) | 2.0 (2.0–4.0) | 2.0 (2.0–3.0) | Child .386; Parent .043 |
| q2 | Difficulty reaching objects | 2.5 (2.0–3.0) | 2.5 (2.0–3.0) | 2.0 (1.8–3.0) | 1.0 (1.0–2.0) | Child .770; Parent .138 |
| q3 | Dependence on others | 3.0 (2.2–3.8) | 3.0 (3.0–3.0) | 3.0 (2.8–4.0) | 2.0 (1.5–2.5) | Child .955; Parent .120 |
| q4 | Feeling small around others | 2.0 (1.2–3.5) | 2.5 (2.0–3.0) | 2.0 (2.0–3.0) | 2.0 (1.0–2.0) | Child .866; Parent .427 |
| q5 | Being teased or laughed at | 4.0 (3.2–4.0) | 4.0 (4.0–4.0) | 3.0 (2.0–3.2) | 3.0 (2.5–3.0) | Child .234; Parent .010 |
| q6 | Being treated differently | 4.0 (3.2–4.0) | 3.5 (3.0–4.0) | 3.5 (2.8–4.0) | 2.0 (2.0–2.5) | Child .778; Parent .113 |
| q7 | Feeling different | 3.0 (2.0–4.0) | 2.5 (2.0–3.8) | 3.0 (2.0–4.0) | 2.0 (1.5–3.5) | Child .910; Parent .822 |
| q8 | Insecurity about height | 4.0 (3.2–4.0) | 3.5 (3.0–4.8) | 3.0 (3.0–4.0) | 2.0 (1.5–3.5) | Child .395; Parent .077 |
| q9 | Being bothered by height | 3.5 (3.0–4.0) | 3.5 (3.0–4.0) | 3.0 (2.8–4.0) | 2.0 (1.0–3.5) | Child .737; Parent .265 |
| Total | | 50.0 | 50.0 | 52.8 | 33.3 | Child |

| | | | | | | |
|---|---|---|---|---|---|---|
| (0–100) | | (40.3–70.1) | (47.9–60.4) | (37.5–56.9) | (13.9–41.7) | .664; Parent .030 |

*Table 1 Values are reported as median (interquartile range). Child- and parent-reported QoLISSY Brief scores are shown for hypochondroplasia (HCH) and achondroplasia (ACH). Individual items are scored on a 5-point Likert scale for analysis, where 1 = extremely and 5 = never. Items Q1–Q3 correspond to the physical domain, Q4–Q6 to the social domain, and Q7–Q9 to the emotional domain of the QoLISSY Brief questionnaire. The total QoLISSY Brief score was calculated by summing item scores (maximum 45) and linearly transforming the result to a 0–100 scale, with higher scores indicating better quality of life. Between-group comparisons between HCH and ACH were performed using the Mann–Whitney U test, with p-values reported in the last column. Within-group comparisons between child- and parent-reported total scores were assessed using Wilcoxon paired tests and showed no statistically significant differences. Item-level analyses should be interpreted as exploratory. The QoLISSY Brief questionnaire (2015) was used with permission and licensing from IQVIA (Licence number:* ***QUO-06615-J5N4N6).***

### Disclosure

None declared

**P154**

Abstract withdrawn

### P155

### Phenotypic Differences among Three Mouse Models of Osteogenesis Imperfecta

Mayumi Umebayashi[1], Abhimanyu Mertiya[1,2], Joan C Marini[3], Frank Rauch[1,4], Bettina M Willie[1,2,5]

[1]Shriners Hospital for Children-Canada, Research Center, Montreal, Canada. [2]Faculty of Dental Medicine and Oral Health Sciences, McGill University, Montreal, Canada. [3]Section on Heritable Disorders of Bone and Extracellular Matrix, NICHD, NIH, Bethesda, USA. [4]Department of Pediatrics, McGill University, Montreal, Canada. [5]College of Veterinary Medicine, Utah State University, Logan, USA

### Abstract

**OBJECTIVE:**

The present study aims to compare the bone phenotype in three commonly used mouse models of osteogenesis imperfecta (OI) associated with type I collagen mutations: for *Col1a1,* Brtl with Gly349Cys substitution, Jrt with 18 amino acid deletion, and for *Col1a2,* oim with G3983 deletion. Brtl and Jrt are caused by dominant mutation, while oim is recessive.

**METHODS:**

Male and female seventeen-week-old Brtl and Jrt wild type (WT, +/+) and heterozygous (+/-) mice, and oim, WT, heterozygous and homozygous (-/-) genotypes (n=8 mice/genotype/sex) were analyzed. The background strain of the mice were: mixed background of SV129/CD-1/C57BL/6S (Brtl), FVB (Jrt), and C57BL/6J (oim). We performed *in vivo* whole body microCT at baseline and in vivo microCT of the tibiae and activity analyses over two weeks, followed by histomorphometry and three point bending analyses. ANOVA, Tukey's post-hoc, and t-tests were performed ($p<0.05$)

**RESULTS:**

At the endpoint, Jrt(+/-) males, Brtl(+/-) and Jrt(+/-) females exhibited shorter right femurs (4–6%) compared with their respective WT controls (**Fig.A**). Brtl (+/-) and Jrt(+/-) had avulsion fractures or deformities at olecranon process or calcaneus, while oim(-/-) mice had avulsion fractures as well as long bone fractures and deformities (**Fig.B**). The oim(-/-) females had lower activity (distance traveled, 40%) compared with WT, and the rearing counts were significantly lower (47%) in Jrt(+/-) males compared to WT. All OI genotypes had lower maximum load (29-54%), work to fracture (48-90%) and compared with their respective WT mice **(Fig.C)**. However, there was selection bias, since only 5 of 18 oim(-/-) male mice and 7 of 9 oim(-/-) female mice could undergo mechanical testing.

**CONCLUSIONS:**

The oim(-/-) of both sexes had the most fractures or deformities, including in long bones. All three mouse models of OI have inferior mechanical properties compared to their WT controls. These data allow for a clear comparison between OI mouse models, based on bone phenotype in skeletally mature mice, although future studies should examine other ages. These comparisons will facilitate the selection of a particular OI mouse model for drug development or investigation of disease mechanisms.

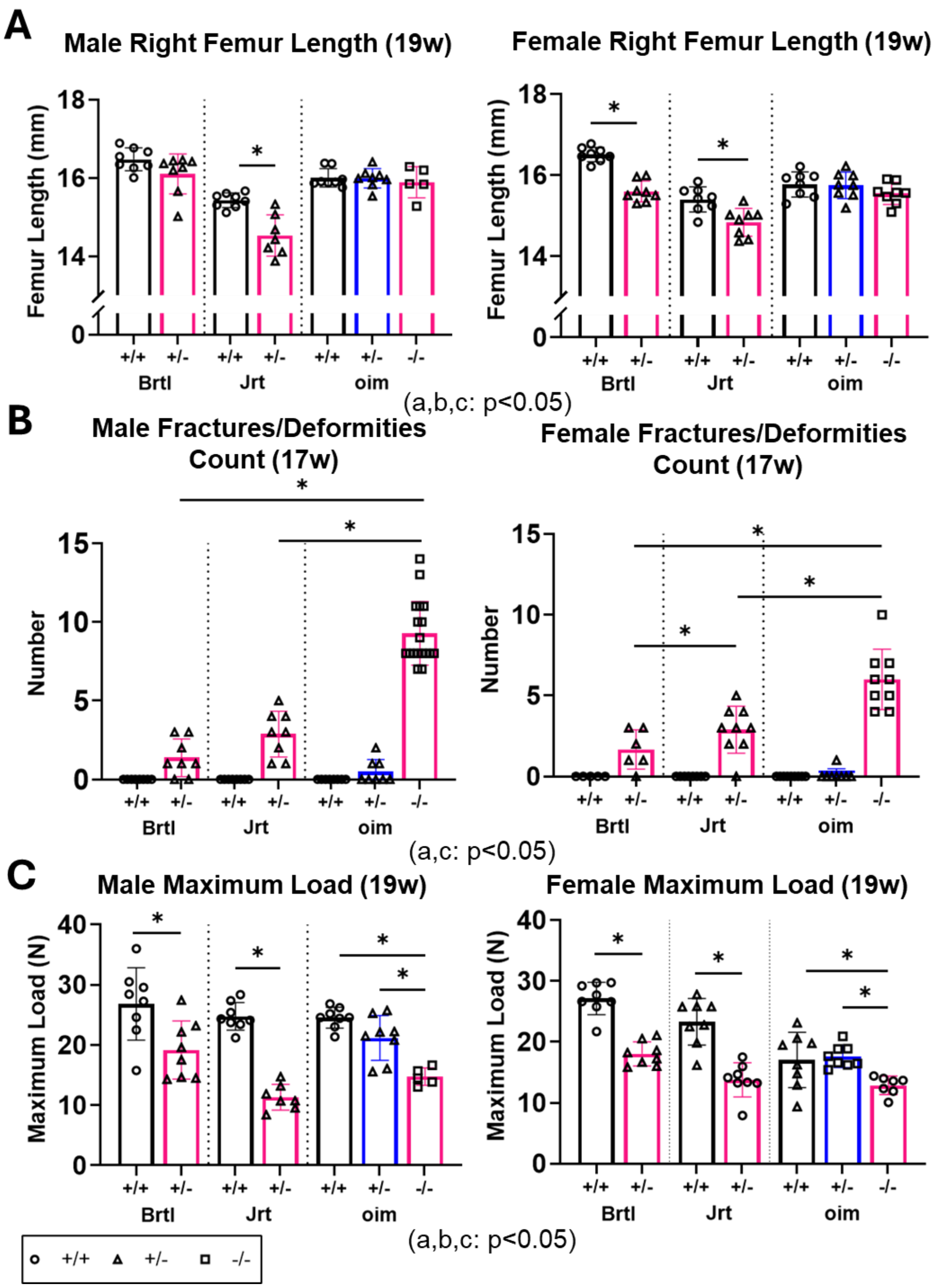

**Figure. Comparison of OI mouse models: (A) Right femur length at end point (19w-old); (B) Fractures or deformities count at baseline (17w-old); (C) Maximum load at end point (19w-okd);** Means ± SD; ANOVA (a=genotype, b=sex, c=genotype*sex), Tukey's post-hoc test; t-test (p<0.05).

**Disclosure**

None declared

### P156

### Age-Specific Toolkits for Osteogenesis Imperfecta: Empowering Patients and Providers from Diagnosis Through Adulthood

Laura Tosi[1], Gabriela Beug[2], Andrew Pennington[1], K Erika Carter[2], Michelle Ronan[2], Mackenzie Coleman[2], Pankhuri Kabthiyal[1], Tracy Hart[2]

[1]Children's National Hospital, Washington, USA. [2]Osteogenesis Imperfecta Foundation, Gaithersburg, USA

### Abstract

**Objectives**:

Osteogenesis Imperfecta (OI) is a rare genetic disorder characterized by bone fragility and multisystem complications that evolve across the lifespan. From diagnosis onward, individuals with OI and their families navigate complex medical, educational, and psychosocial needs within a fragmented healthcare landscape. To address these challenges, the Osteogenesis Imperfecta Foundation (OIF) developed three age-specific, patient-centered toolkits to support newly diagnosed individuals, school-aged children, and adults with OI.

**Methods:**

Toolkit content was derived directly from community-identified needs. The development team analyzed common inquiries received through OIF email and phone contacts, reviewed recurring topics in the OI Facebook Community Forum, and synthesized questions raised during regional meetings, support groups, and national conferences. OIF staff translated these inputs into accessible, practical guidance for individuals with OI, caregivers, and healthcare professionals. Source materials included OIF educational resources, peer-reviewed literature, government publications, and reputable clinical websites. To enhance usability, the toolkits incorporated visual aids and interactive components, including health trackers designed to centralize key medical information. Each toolkit underwent iterative internal and external review by clinical experts and community stakeholders.

**Results:**

People with OI, their caregivers, clinical experts, and OIF staff developed three toolkits – New Diagnosis, School-Aged, and Adult Health – each tailored to a distinct life stage. Designed as accessible, comprehensive resources for individuals with OI, their caregivers, and healthcare providers, the toolkits integrate medically reviewed information, visual aids, and interactive health trackers to facilitate decision-making and communication across care teams. These free, multilingual resources are built to inform, empower, and equip both patients and providers with accurate, actionable guidance.

**Conclusions:**

The OI Foundation’s development of three age-specific toolkits offers a replicable, scalable framework for developing patient-centered resources across other rare diseases – particularly those facing similar challenges of fragmented care availability and limited provider awareness. By integrating clinical expertise and patient lived experience, the OI toolkit model demonstrates how community-driven collaboration can translate complex medical information into practical tools that empower patients and clinicians alike.

### Disclosure

None declared

### P157

### Long-Term Survival, Patient-Reported Outcomes and Quality-of-Life in Children With HPP Treated with Asfotase Alfa Starting Before 2 Years of Age

Cheryl Rockman-Greenberg[1], Priya S Kishnani[2], Lothar Seefried[3], Keiichi Ozono[4], Gabriel Ángel Martos-Moreno[5], Luke K Burke[6], Peter R Ebeling[7], Agnès Linglart[8], Shona Fang[9], Anna Petryk[10], Kathryn M Dahir[11], Wolfgang Högler[12,13]

[1]University of Manitoba, Manitoba, Canada. [2]Duke University Medical Center, Durham, USA. [3]University of Würzburg, Würzburg, Germany. [4]ISEIKAI International General Hospital, Osaka, Japan. [5]Hospital Infantil Universitario Niño Jesús, IIS La Princesa, Universidad Autónoma de Madrid, CIBERobn, ISCIII, Madrid, Spain. [6]Global Scientific Communications, Alexion, AstraZeneca Rare Disease, Boston, USA. [7]School of Clinical Sciences, Monash University, Clayton, Australia. [8]Paris-Saclay University, AP-HP and INSERM, Kremlin-Bicêtre, France. [9]Epidemiology and Real World Science, Alexion, AstraZeneca Rare Disease, Boston, USA. [10]Global Medical Affairs, Alexion, AstraZeneca Rare Disease, Boston, USA. [11]Vanderbilt University Medical Center, Nashville, USA. [12]Department of Metabolism and Systems Science, School of Medical Sciences, College of Medicine and Health, University of Birmingham, Birmingham, UK. [13]Department of Pediatrics and Adolescent Medicine, Johannes Kepler University Linz, Linz, Austria

### Abstract

**Objectives:**

Hypophosphatasia (HPP) is a rare disease caused by deficient tissue-nonspecific alkaline phosphatase (ALP) activity. We describe long-term clinical outcomes and safety in children with HPP who initiated asfotase alfa before 2 years of age.

**Methods:**

Eligible patients from the Global HPP Registry initiated asfotase alfa before age 2 years, treatment duration between 3 and 16 years, had confirmed low ALP activity, and had ≥1 *ALPL* variant. The most recent measurements of height-, weight-, and body mass index (BMI)-for-age *Z*-scores, Pediatric Quality of Life Inventory (PedsQL), and 6-Minute Walk Test (6MWT) after ≥3 years of treatment were ascertained. A Kaplan-Meier curve and an extended Cox model were used to estimate overall survival for all patients regardless of treatment duration. Safety outcomes were summarized.

**Results:**

As of December 2024, 74 patients were treated with asfotase alfa for median [1] (min, max) 6.9 (3.1, 15.9) years. Patient demographics, asfotase alfa treatment information, and outcomes data from most recent assessment are summarized in the **Table**. Most (81%) patients in this population had early-onset HPP (symptomatic before 6 months of age). Overall survival among 124 patients who started asfotase alfa before 2 years of age of any treatment duration was 94.3% (95% CI: 90.0%, 98.9%; **Table**). Six (4.8%) patients died before age 3.5 years, all of whom started treatment at age <1 month; all patient deaths were deemed unrelated to treatment. A total of 8/55 (14.5%) patients had craniosynostosis and 7/55 (12.7%) had injection site reactions; 9 had recorded ectopic calcifications. No patients had severe hypersensitivity reactions or systemic immune complex-mediated reactions.

**Conclusions:**

Patients in this real-world analysis had long-term data showing outcomes after up to 16 years of asfotase alfa treatment. Infants (largely represented by early-onset HPP) treated with asfotase alfa had a high survival rate. Some patients had short stature at last assessment. Median PedsQL scores among treated children were below previously reported mean parent proxy-reported scores from healthy children and similar to scores from children with rheumatic diseases. No new safety signals were identified

**Table.**

| Survival outcomes | Study population with any asfotase alfa treatment(n=124) |
|---|---|
| Overall survival (95% CI) | 94.3 (90.0, 98.9) |
| Patient deaths, n (%) | 6 (4.8) |
| **Baseline characteristics and clinical outcomes** | **Study population with ≥3 years' asfotase alfa treatment(n=74)** |
| Female sex, n (%) | 44 (59.5) |
| White race, n (%) | 34 (45.9) |
| Patients with early-onset HPP, n (%) | 60 (81.1) |
| Age at treatment start, median (min, max), mo | 3.4 (0, 22.1) |
| Age at last treatment follow-up, median (min, max), y | 7.3 (3.1, 16.5) |
| Time from HPP diagnosis to asfotase alfa start median (min, max), mo | 1.0 (0, 27.5) |
| Treatment [2] duration, median (min, max), y | 6.9 (3.1, 15.9) |
| Patients starting asfotase alfa at 6 mg/kg per week, n (%) | 55[a] (76.4) |
| Patients who were dose-stable on asfotase alfa, n (%) | 54[a] (75.0) |
| Height-for-age *Z*-score at most recent assessment | |
| n | 67 |
| Median (min, max) | –2.3 (–6.9, 1.5) |
| Weight-for-age *Z*-score at most recent assessment | |
| n | 67 |
| Median (min, max) | –1.6 (–6.8, 2.0) |
| BMI-for-age *Z*-score at most recent assessment | |
| n | 67 |

| | |
|---|---|
| Median (min, max) | 0 (–3.3, 4.0) |
| PedsQL total score at most recent assessment | |
| n | 59 |
| Median (min, max) | 72.8 (30.6, 100.0) |
| 6MWT percent predicted distance walked at most recent assessment | |
| n | 10[b] |
| Median % (min %, max %) | 83.7 (47.0, 92.0) |

[a]n=72.

[b]One patient used an assistive device during the 6MWT

### Disclosure

CRG, PSK, GÁMM, PRE, AL, and KMD have consulted for and received research funding and honoraria from Alexion, AstraZeneca Rare Disease. LS is a clinical study investigator and has received consultancy fees and institutional research funding and/or grant support from Alexion, AstraZeneca Rare Disease; Amgen; AM-Pharma; BioMarin; Chiesi; Haleon/GSK; Inozyme; Ipsen; Kyowa Kirin; Medipharma; Novartis; STADApharm; Theramex; UCB; and Ultragenyx. KO has received research funding and honoraria, and support for meetings and/or travel from Alexion, AstraZeneca Rare Disease. LKB, SF, and AP are employees of Alexion, AstraZeneca Rare Disease and may hold stock or options in AstraZeneca. WH has consulted for and received research funding and honoraria from Alexion, AstraZeneca Rare Disease and BioMarin; received support for meetings and/or travel from BioMarin; Novo Nordisk; Sandoz; and Alexion, AstraZeneca Rare Disease.

### P158

### Development of BMN 333, a Long-Acting Version of Vosoritide for FGFR3-Related Dwarfism and Other Skeletal Dysplasias

Daniel Wendt, Kathy Tang, Heather Wenzel, Huiyu Zhou, Donnie Mackenzie, Ryan Dell, Sarah Dupras, Lin Xie, Brian Kaplowitz, Rebecca Rowland, Ryan Murphy, Madhav Sachar, Kristin Evans, Yulan Qi, Brett Crawford

BioMarin Pharmaceutical, San Rafael, USA

### Abstract

Objectives: BMN 333 is an investigational, fully biodegradable treatment designed to extend exposure of vosoritide, its active moiety. BMN 333 aims to increase growth velocity in children with achondroplasia (ACH) and hypochondroplasia (HCH) by inhibiting ERK/MAPK signaling from FGFR3 gain-of-function mutations, without the hemodynamic effects of high vosoritide peaks.

Methods: Adult male mice (n=48) received a single subcutaneous injection of BMN 333 at two dose levels (500 and 1600 µg/kg), quantified by LCMS/MS for pharmacokinetic (PK) assessment. Male 3-week-old wild-type (wt) mice (n=40) received subcutaneous vehicle or BMN 333 at 80, 300, or 1000 µg/kg Q2D, or 500, or 1600 µg/kg QD for 5 weeks. Naso-anal and femoral lengths were measured via microCT. Male and female 2-year-old cynomolgus monkeys (n=52) with telemetry implants received subcutaneous vehicle or BMN 333 at 75, 200, or 350 µg/kg once weekly (QW) for 26 weeks. Femoral lengths were measured radiographically at baseline and during treatment, and PK was assessed on Day 1 and Day 176 using LCMS/MS.

Results: BMN 333 demonstrated a half-life of 10-12 hours in mice and 52-67 hours in cynomolgus monkeys. In wild-type mice, naso-anal length increased significantly at all doses, and femoral length increased significantly at 1000 µg/kg Q2D, 500 µg/kg QD, and 1600 µg/kg QD. In cynomolgus monkeys, femoral length increased significantly at 350 µg/kg QW in both sexes, and at 200 µg/kg QW and higher in females.

Conclusions: BMN 333 is a biodegradable treatment that extends vosoritide's half-life. In juvenile mice and monkeys, BMN 333 provides consistent vosoritide release, leading to dose-dependent increases in endochondral bone growth without hemodynamic effects. These findings support the clinical evaluation of BMN 333, a less frequently administered form of vosoritide, in children with achondroplasia or hypochondroplasia.

Disclosure

None declared

### P159

### Next-Generation Sequencing in Pediatric Musculoskeletal Disorders: A Multi-Center Experience

Ghalib Bardai, Zenghui Wu, Elizabeth Parr, Marie-Josee Giguere, Frank Rauch

Shriners Hospital for Children- Canada, Montreal, Canada

### Abstract

**OBJECTIVES:**

The Biomedical Clinical Laboratory at Shriners Hospital for Children – Canada provides College of American Pathologists-accredited molecular diagnostics specializing in heritable musculoskeletal conditions. As genomic technologies evolve, the transition from targeted panels to broad "Mendeliome" approaches aims to increase diagnostic yield. Here, we evaluate the diagnostic yield and mutational spectrum of large-panel sequencing in a diverse pediatric cohort over three years.

**METHODS:**

Exome sequencing was performed on genomic DNA using Illumina NextSeq550, NextSeq1000 or NextSeq2000 devices. Panels were based on genes validated for disease associations by PanelApp. Bioinformatic analysis utilized two primary strategies: 1) A targeted Skeletal Disorders Panel (Jan 2023–Aug 2025), which expanded from 448 to 548 genes over time, and 2) A comprehensive Mendeliome Panel (Aug 2025–Jan 2026) encompassing 4,103 genes. Panels were based on genes validated “green” for disease associations by PanelApp Australia.

**RESULTS:**

Samples were received for clinical molecular diagnostic testing from 9 different Shriners Hospitals for Children in Canada, the US and Mexico. Next-generation sequencing was performed on the index patients of 1176 unrelated families. In 428 index patients (36%), a disease-related variant was detected. The diagnostic yield varied significantly by disorder type ($P < 0.001$) and was highest for osteogenesis imperfecta/bone fragility conditions (Table 1). Disease-causing variants were found in 113 different genes. *COL1A1* and *COL1A2* were the most commonly involved genes (42% combined) followed by *COMP* (3%), *PHEX* (3%), *FBN1* (3%), *DMD* (3%), *FGFR3* (2%) and *COL2A1* (2%).

**CONCLUSIONS:**

The overall diagnostic yield for musculoskeletal disorders is similar to that reported for other conditions but varies significantly by diagnostic category.

**Figure 1.** Diagnostic Yield by Clinical Category for 1,176 unrelated families.

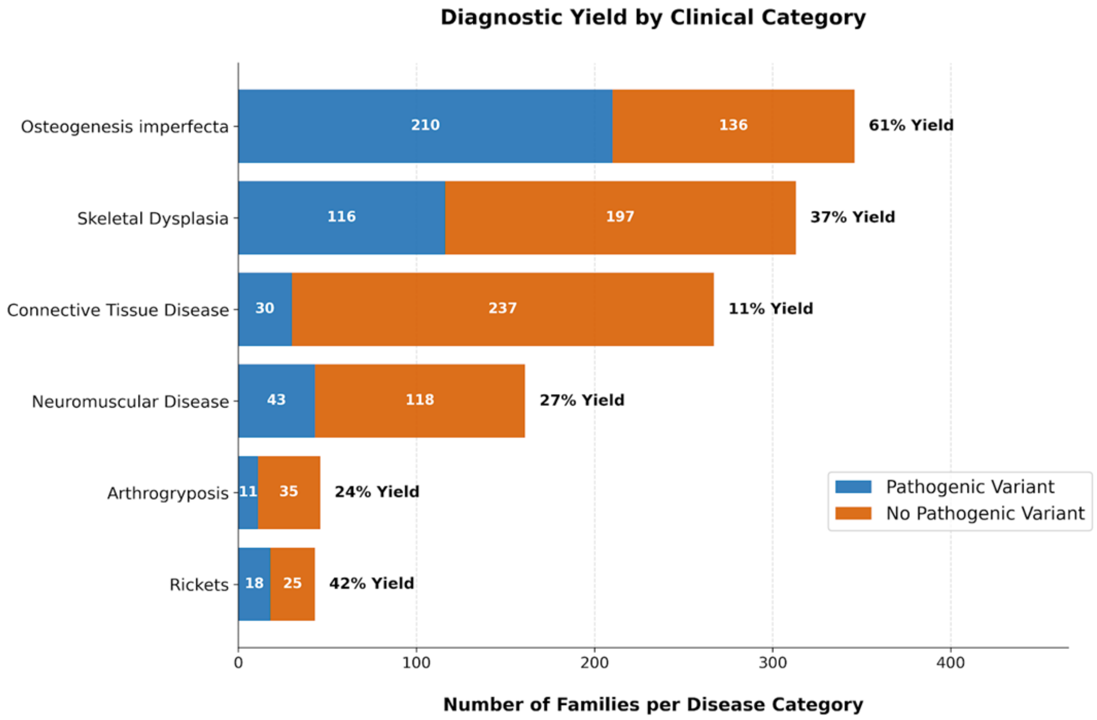

### Disclosure

None declared

### P160

### Guideline for Pediatric-to-Adult Care Transition in Childhood Rare Bone Diseases

Laura Tosi[1], MS Eleanor Perfetto[2], Gabriela Beug[2], K Erika Carter[2], Pankhuri Kabthiyal[1], Andrew Pennington[1], Tracy Hart[2]

[1]Children's National Hospital, Washington, USA. [2]Osteogenesis Imperfecta Foundation, Gaithersburg, USA

### Abstract

**Objectives:**
People living with rare bone diseases (RBDs) face significant challenges moving from pediatric to adult care. Families frequently report that: preparation begins too late, leaving uncertainty about what is involved and who coordinates adult care; adult clinicians expert in RBDs are few; access to multidisciplinary services (e.g., orthopedics, endocrinology, rehabilitation) is more fragmented for adults than in pediatric centers; and critical clinical information gets lost during handover. Without a dedicated transition coordinator, families must manage appointments, records, and insurance issues on their own. To address these gaps, the Rare Bone Disease Alliance (RBDA), in collaboration with the Osteogenesis Imperfecta Foundation (OIF), initiated development of care-transition guidelines for individuals with childhood-onset RBDs. We will describe the process, results, and anticipated impact of this effort.

**Methods:**

An expert committee of patients, clinicians, and researchers from RBDA-member organizations guided all phases of the project. Existing evidence and materials were evaluated through a systematic review of peer-reviewed and gray literature, and web-based resources addressing pediatric-to-adult transitions. Where evidence was limited, expert consensus was used and clearly identified as such. Draft recommendations underwent RBD-community and public review.

**Results:**

The committee adopted Got Transition®'s (GT) Six Core Elements (SCE) as the most appropriate framework for the U.S.-based transition guidelines. It offered a nationally recognized structure, practical implementation tools, and broad professional support. Adaptations were made for RBD transitions. Key adaptations include: 1) RBD transition planning must include patient/family engagement and empowerment; 2) Patients/families need comprehensive knowledge to seek reliable disease-specific information; 3) Educational materials from trusted sources (e.g., patient organizations) should be shared as early as possible; and 4) A tracking tool should be used to record health history and significant events to inform future adult providers.

**Conclusions:**

This initiative produced guideline recommendations on pediatric-to-adult transitions for people with childhood-onset RBDs and practical recommendations for the RBD community. By leveraging GT materials with disease-specific expertise and patient input, the recommendations address critical gaps and promote consistent, informed, and patient-centered transition care for all individuals with RBDs.

### Disclosure

None declared

### P161

### Prevalence of ALPL Variants in Asymptomatic Individuals with Persistently Low Serum Alkaline Phosphatase

Julian Schirl[1,2], Rodrigo Montero-Lopez[1,2], Alexandra Blaschitz[1,2], Victoria Schuhmann[1,2], Tanja Fritz[1,2], Elisabeth Laurer[1,2], Marcell Tamas[1,2], Thomas Hörtenhuber[1,2], Mariam R Farman[1], Wolfgang Högler[1,2]

[1]Department of Paediatrics and Adolescent Medicine, Johannes Kepler University Linz, Linz, Austria. [2]Centre for Growth and Osteology, Department of Paediatrics and Adolescent Medicine, Kepler University Hospital, Linz, Austria

### Abstract

**Objectives:** Hypophosphatasia (HPP) is a rare metabolic disease caused by pathogenic variants in *ALPL* and characterized by phenotypic variability. While severe early-onset forms are easily diagnosed, mild, late-onset forms remain underdiagnosed. An additional diagnostic challenge has recently emerged with the recognition of a previously underrepresented phenotype: individuals carrying pathogenic *ALPL* variants who exhibit the biochemical signature of HPP (low serum alkaline phosphatase [ALP] and elevated pyridoxal-5'-phosphate) but remain clinically asymptomatic. This condition has been referred to as “asymptomatic *ALPL* carriers with a biochemical phenotype” or *“*subclinical HPP*”.* The aim of this study was to assess the prevalence of *ALPL* variants among individuals with persistently low ALP levels and to characterize the associated genotypic and phenotypic spectrum.

**Methods:** In a two-step recall study, all in- and outpatients with at least two reduced ALP measurements (January 2021 to January 2022) at a large general hospital were retrospectively identified. Patients with known secondary causes of low ALP were excluded in the first step. Individuals with persistently low ALP were invited for further evaluation, including clinical assessment, biochemical testing, and molecular genetic analysis.

**Results:** Among 42,304 patients with ALP measurements, 537 individuals (365 adults, 172 children) had at least two reduced ALP values. In 91/537 (16.9%), no secondary cause of low ALP was identified based on medical records and 39/91 (42.9%) showed persistently low ALP. Of these 39, 17 agreed to further evaluation. Pathogenic *ALPL* variants were detected in 12/17 individuals (70.6%). In total, 9/12 (75%) met criteria for asymptomatic carriers with biochemical phenotype, while 1/12 (8.3%) was diagnosed with adult HPP and 1/12 with odonto-HPP. A novel *ALPL* variant (c.376G>A) was identified in two unrelated individuals, and three variants (c.119C>T, c.653T>C, c.997+2T>A) were detected for the first time in association with a subclinical phenotype.

**Conclusions:** This recall-based screening study revealed a remarkable prevalence (9/17; 52,9%) of pathogenic *ALPL* variants in asymptomatic individuals with persistently low serum ALP and uncovered previously unreported genotypic–phenotypic associations. Our findings emphasize the need for increased awareness of low ALP values and careful diagnostic characterization of different phenotypes of HPP, to avoid under- and overdiagnosis of HPP.

### Disclosure

W.H. is consultant for and have received research funding and honoraria from, Alexion, AstraZeneca Rare Disease. M.R.F. is funded through a research grant from Alexion, AstraZeneca Rare Disease and paid by Johannes Kepler University Linz. R.M.L. received travel funding from Alexion, AstraZeneca. The other authors have no conflicts of interest to declare.

### P162

### Inorganic Pyrophosphate Metabolism Modulates XLH Pathologies in *Hyp* Mice

Monzur Murshed

Shriners Hospital for Children, Canada, Montreal, Canada. McGill University, Montreal, Canada

### Abstract

OBJECTIVES:

X-linked hypophosphatemia (XLH) is a hereditary disorder characterized by rickets and osteomalacia caused by loss-of-function mutations in the phosphate-regulating endopeptidase X-linked (PHEX) gene, leading to increased production of fibroblast growth factor 23 (FGF23) and chronic hypophosphatemia. Conventional treatment with phosphate supplement substantially improves bone mineralization; however, certain skeletal and extraskeletal pathologies may persist. This study aimed to investigate the contribution of systemic phosphate levels and local regulators of mineralization, particularly inorganic pyrophosphate metabolism, to XLH pathophysiology.

METHODS:

We used *Hyp* mice, a faithful model of human XLH, transgenic mice overexpressing FGF23, and compound *Hyp;Enpp1*$^{asj/asj}$ double-mutant mice with markedly reduced inorganic pyrophosphate synthesis due to mutation of the ectonucleotide pyrophosphatase 1 (*Enpp1*) gene. Mice were fed a high-phosphorus (high P) diet, and skeletal mineralization, osteomalacia, perivascular mineralization, and cortical porosity were assessed using histomorphometry, fluorescence microscopy, and X-ray–based imaging techniques.

RESULTS:

A high P diet improved bone mineralization in *Hyp* mice but did not fully correct all mineralization-associated abnormalities. In contrast, the same diet was more effective in correcting mineralization defects in mice overexpressing FGF23. In *Hyp;Enpp1*$^{asj/asj}$ mice, high P feeding significantly improved osteomalacia, enhanced mineralization of perivascular lesions, and showed signs of improvement in cortical porosity, suggesting that reduced inorganic pyrophosphate levels modify disease manifestations and treatment response.

CONCLUSIONS:

These findings indicate that while correction of systemic hypophosphatemia substantially improves skeletal mineralization in XLH, local regulators of mineralization also play an important role in disease pathology. Modulation of inorganic pyrophosphate metabolism may therefore represent a complementary therapeutic approach to address residual skeletal and vascular abnormalities in XLH.

### Disclosure

None declared

### P163

### Perceived Barriers to Physio- and Occupational Therapy Needs in Children with Bone Disorders: A Survey-Based Study as Part of the Navigating the Inter-disciplinary Care Experience (NICE) Bone Health Research Program

Stefan Jackowski[1], Jessica Buffam Buffam[1], Serdar Kilicarslan[1,2], Ayushi Regmi[1], Jinhui Ma[3], Mei Wang[1], Nasrin Khan[1], Fahd Alshammri[1,4], Sarah Bakhamis[5], Ulrich Montcho[1,4], Rana Halloun[1,4], Leanne Ward[1,4]

[1]Children's Hospital of Eastern Ontario, Ottawa, Canada. [2]Division of Neurology, Department of Pediatrics, University of Ottawa, Ottawa, Canada. [3]McMaster University, Hamilton, Canada. [4]Division of Endocrinology, Department of Pediatrics, University of Ottawa, Ottawa, Canada. [5]Section of Pediatric Endocrinology, Department of Pediatrics, King Faisal Specialist Hospital & Research Centre, Riyadh, Saudi Arabia

### Abstract

***Objective:*** Pediatric patients with rare bone diseases face bone fragility, chronic pain, and reduced mobility. Physiotherapy (PT), occupational therapy (OT), and mobility devices are often recommended, yet access may be limited by barriers. This study examines the barriers perceived by caregivers of patients with rare bone diseases in accessing rehabilitation services.

***Methods***: A prospective, cross-sectional observational survey study was conducted involving 105 children <19 years of age receiving care in a tertiary care pediatric rare bone disease clinic. Data were collected through patient and caregiver questionnaires and chart reviews. The questionnaires were designed to explore perceived barriers to accessing and participating in rehabilitation services (e.g. logistical, financial, and personal factors). Descriptive analyses summarized participant characteristics and reported barriers.

***Results:*** Overall, 49.5% of caregivers and patients (52/105) reported at least one barrier to accessing PT, OT, or mobility devices as part of rehabilitative care. Among those reporting at least one barrier, 15.4% (8/52) reported feeling overwhelmed when choosing a rehabilitation clinic, and 43.3% (17/52) indicated a need for more information about available rehabilitation services in their community. Among the 62.9% of patients (66/105) requiring PT, 18.2% (12/66) were unable to access services, with 71.2% (47/66) reporting at least one barrier. The most frequently reported barrier was limited appointment availability outside of work or school hours (29/66, 43.9%). OT was required by 27.6% of patients (29/105), of whom 72.4% (21/29) experienced at least one barrier. As for PT, limited appointment availability outside of work or school hours was the most common barrier to OT (11/29, 37.9%). Among patients requiring mobility devices (38.1%, 40/105), 60.0% (24/40) reported barriers, mostly related to family financial constraints (15/40, 37.5%).

***Conclusions:*** Patients with rare bone disorders and their caregivers experienced substantial barriers to accessing rehabilitation services across PT, OT, and mobility devices. Common barriers included limited appointment availability outside of work or school hours and financial constraints. Many families also reported feeling overwhelmed when selecting providers and needing more information. Flexible scheduling, financial support, and patient navigation services may help reduce these barriers and improve access to rehabilitative care.

### Disclosure

None declared

### P164

### The International Osteogenesis Imperfecta Registry: A Collaborative Platform to Advance Orthopaedic Outcomes Research in Osteogenesis Imperfecta

Harpreet Chhina[1,2], Kaylee McGeough[1,2], Andrew Georgiadis[3], Maegen Wallace[4], Jill Flanagan[5], Jeanne Franzone[6], Anthony Cooper[1,2]

[1]Department of Orthopaedics, BC Children's Hospital, Vancouver, Canada. [2]Department of Orthopaedics, UBC, Vancouver, Canada. [3]Gillette Children's Hospital, St. Paul, Minnesota, USA. [4]Phoenix Children's Hospital, Phoenix, Arizona, USA. [5]Children's Healthcare of Atlanta, Atlanta, Georgia, USA. [6]Nemours Children's Hospital, Wilmington, Delaware, USA

### Abstract

### Objective:

Osteogenesis Imperfecta (OI) is a rare genetic disorder characterized by bone fragility, recurrent fractures, and progressive deformities. Despite the clinical heterogeneity and lifelong impact of musculoskeletal manifestations due to OI, there is a paucity of longitudinal, standardized data capturing the nature and evolution of limb differences across the lifespan. To address this gap, under the International Limb Differences Registry (ILDR), we have established a dedicated longitudinal observational registry for OI.

The objective is to collect long-term, standardized, patient-centered data on children and adolescents with OI to support orthopaedic outcomes research, improve clinical care, and inform shared decision-making.

### Methods:

The ILDR-OI registry was developed by an international group of orthopaedic surgeons and researchers with expertise in treating OI. Core data elements include demographics, socio-economic status, OI type, fracture history, surgical interventions (e.g., intramedullary rodding, osteotomies), radiological assessments, bone density, adverse events, and patient-reported outcomes. Numeric pain rating scale, PROMIS Global Health Scale, and LIMB-Q Kids are used to collect patient-reported outcomes at baseline and every six months from baseline.

The registry design allows for prospective longitudinal data collection and entry of any prior treatment for OI before joining the registry. Data are securely collected using REDCap, a web-based electronic data capture system.

### Results:

Since the launch of the OI registry in 2024, 5 sites from Canada and the USA have recruited 116 patients (Table 1). 22 patients have been followed prospectively for at least 3 months since recruitment and 23 surgeries, 13 fractures, 3 bone density scans, and 3 adverse events have been reported.

At the time of abstract submission, 12 sites in total (Germany, the Netherlands, India, Canada, Nigeria, and the United States) have all necessary approvals for recruitment for ILDR. Four sites (Denmark, India, United States, and the UK) that are currently obtaining the required approvals.

### Conclusion:

This comprehensive OI registry represents a significant advancement in the global effort to improve understanding of OI. With a dedicated international scientific advisory committee, research team and data management infrastructure, this platform is well-positioned to generate high-quality, clinically meaningful data. The registry data will support studies aimed at addressing existing gaps in the treatment and long-term management of individuals with OI. Ongoing international expansion of this registry will further enhance its capacity to inform best practices and improve quality of life for patients living with OI.

**Table 1**: Demographic and clinical data at the time of recruitment into the registry by OI severity

| | **Mild** | **Moderate** | **Severe** | **Total** |
|---|---|---|---|---|
| **Total Recruits** | **35** | **24** | **35** | **116**<br>**22 OI severity not reported* |
| **Age at recruitment (years)** | **8.1**<br>(Max = 15.8, Min = 2.7)<br>**1 age not reported* | **8.6**<br>(Max = 18.2, Min = 1.9) | **7.0**<br>(Max = 17.9, Min = 0.7) | **7.8**<br>(Max = 18.2, Min = 0.7)<br>**10 age not reported* |
| **Sex** | Female = **13**, Male = **22** | Female = **14**, Male = **10** | Female = **15**, Male = **20** | Female = **48**, Male = **67**<br>**1 sex not reported* |
| **Type of OI** | Type 1=33, Type 4=2 | Type 1=5, Type 3=2, Type 4=13, Type 5=1,<br>Type 6=2<br>**1 type not reported* | Type 1=3, Type 2=1. Type 3=26, Type 4=1, Type 7=1, Type 8=2, Type 18=1 | Type 1=43, Type 2=1, Type 3=29, Type 4=20, Type 5=1, Type 6=3, Type 7=1, Type 8=2, Type 18=1, Type 21=2<br>**13 type not reported* |
| **Median Number of Previous *Surgeries*Per Patient Reported at Recruitment** | **2**<br>(IQR 2, 1, n=13) | **2**<br>(IQR 4, 2, n=11) | **4**<br>(IQR 6.5, 2.8, n=16) | **2**<br>(IQR 4, 2, n=46) |
| **Median Number of Previous *Fractures* Per Patient Reported at Recruitment** | **4**<br>(IQR 7, 3, n=21) | **6.5**<br>(IQR 11, 2.8, n=12) | **9**<br>(IQR 11, 3, n=21) | **6**<br>(IQR 11, 3, n=65) |
| **Median *PROMIS Pediatric Global Health*Score Reported at Recruitment**<br>**(max 100, 100 reflect a better score)** | Parent = **51.8**<br>(IQR 56.6, 48.4, n=14)<br>Self = **50.4**<br>(IQR 57.7, 45.7, n=10) | Parent = **50.6**<br>(IQR 61.4, 48.1, n=10)<br>Self = **37.2**<br>(IQR 41.45, 34, n=3) | Parent = **49.3**<br>(IQR 54.5, 45.4, n=15)<br>Self = **48.3**<br>(IQR 51.5, 44.5, n=4) | Parent = **51.8**<br>(IQR 54.5, 47.3, n=41)<br>Self = **47.5**<br>(IQR 55.7, 40.4, n=17) |
| **Median *LIMB-Q Kids* Scores** | Leg Appearance | Leg Appearance | Leg Appearance | Leg Appearance = **70** |

| | | | | |
|---|---|---|---|---|
| **Reported at Recruitment** **(Self-report only, max 100, 100 reflects a better score)** | = **70** (IQR 82, 64, n=4) Physical Function = **100** (IQR 100, 90, n=5) | = **63** (IQR 79.8, 49.5, n=5) Physical Function = **83** (IQR 87.3, 69, n=5) | = **73** (IQR 93.3, 54.3, n=3) Physical Function = **69** (IQR 72, 43.5, n=3) | (IQR 82, 53, n=13) Physical Function = **73** (IQR 100, 60, n=12) |
| **Median *Numeric Pain Rating*Score Reported at Recruitment (max 10, 0 reflects a better score)** | **1** (IQR 1.3, 0, n=23) | **0.3** (IQR 3.5, 0, n=10) | **0.8** (IQR 2.4, 0, n=12) | **1** (IQR 1.7, 0, n=49) |

### Disclosure

None declared

### P165

### Patient-Reported Prevalence of Gastrointestinal Symptoms in Children with Osteogenesis Imperfecta: A Retrospective Review

Chloe Derocher[1,2], Laurel Baxter[2], Erin Carter[2], Cathleen Raggio[2], Maegan Wallace[1]

[1]Phoenix Children's Hospital, Phoenix, USA. [2]Hospital for Special Surgery, New York, USA

### Abstract

**OBJECTIVES:**

Osteogenesis imperfecta (OI) is a rare, heritable type I collagenopathy characterized by fragile bones and frequent fractures. The current literature on gastrointestinal (GI) manifestations in OI remains limited, with a handful of studies suggesting a possible link between OI severity and constipation. Thus, the purpose of this study was to better understand the self-reported prevalence and characteristics of GI symptoms in children with OI.

**METHODS:**

This IRB-approved, retrospective review included 42 participants with a clinical and/or genetic diagnosis of OI aged 8 months to 17.9 years old who were seen at Phoenix Children's Hospital in Phoenix, AZ, or Hospital for Special Surgery in New York, NY, between September 2023 and December 2025. Responses to the standard-of-care parent-reported Questionnaire on Pediatric Gastrointestinal Symptoms, Rome III Version (QPGS-RIII) were collected for children ≥ 4 years old, and a self-reported version for those ≥ 10 years old. Statistical analysis consisted of descriptive statistics and Fisher's exact test to compare groups ($p<0.05$).

**RESULTS:**

Forty responses to parent-reported QPGS R-III were collected (22 M, 18 F), with the average age 9.4±3.7 years. A total of 15 responses to the child-reported QPGS R-III were collected (6 M, 9 F), with average age 13.3±2.1 years. Further participant demographics, including OI type, are shown in Table 1. Parents reported that 16 children (40%) qualified for at least one GI diagnosis based on symptoms, with three children meeting criteria for two diagnoses. The most common diagnoses were functional constipation (9/40; 22.5%), irritable bowel syndrome (3/40; 7.5%), and cyclic vomiting syndrome (3/40; 7.5%). Two children qualified for a GI diagnosis based upon the self-reported questionnaire (13.3%). There was no significant difference in having a GI diagnosis based on type of OI or sex. Eleven of 39 children (28.2%) were reported as having seen a pediatric gastroenterologist.

**CONCLUSIONS:**

Overall, a relatively high prevalence of children with OI with GI symptoms met criteria for a functional diagnosis, particularly functional constipation. Interestingly, the discrepancies between parent- vs child-reported survey answers (i.e. children reporting GI symptoms less than parents) may be important, highlighting a potential limitation of these surveys in the OI population.

**Table 1.** Demographic characteristics and parent-reported QPGS-RIII outcomes for study participants (n=40).

| | **Frequency (%)** | **Mean (SD)** |
|---|---|---|
| **Sex** | | |
| Female | 18 (45%) | |
| Male | 22 (55%) | |
| **Age (years)** | | 9.4±3.7 |
| **OI type** | | |
| Type I | 16 (40%) | |
| Type III | 6 (15%) | |
| Type IV | 9 (22.5%) | |
| Type V | 1 (2.5%) | |
| Type VIII | 2 (5%) | |
| Other/unknown | 6 (15%) | |
| **Assistive device use (n=39)** | 17 (43.6%) | |
| **Height, cm (n=38)** | | 122.4±23.5 |
| **Weight, kg (n=39)** | | 29.4±14.9 |
| **BMI (n=39)** | | 21.5±18.6 |
| **BSA (n=39)** | | 0.98±0.34 |
| **Gastrointestinal diagnoses** | | |
| Functional Dyspepsia | 1 (2.5%) | |
| Irritable Bowel Syndrome | 3 (7.5%) | |
| Abdominal Migraine | 1 (2.5%) | |

| | | |
|---|---|---|
| Functional Abdominal Pain | 0 | |
| Functional Abdominal Pain Syndrome | 0 | |
| Functional Constipation | 9 (22.5%) | |
| Nonretentive Fecal Incontinence | 0 | |
| Aerophagia | 2 (5%) | |
| Cyclic Vomiting Syndrome | 3 (7.5%) | |
| Adolescent Rumination Syndrome | 0 | |

**Disclosure**

Author CR is the Medical Advisory Committee Chair and board member of the Osteogenesis Imperfecta Foundation. The other authors have nothing to disclose.

### P166

### Virtual Reality for Children with Osteogenesis Imperfecta: Novel Avenues for Children's Surgical Education

Yi Wen Wang[1], Franco Carnevale[1], Sylvie LeMay[2], Argerie Tsimicalis[1,3]

[1]McGill University, Montreal, Canada. [2]Université de Montreal, Montreal, Canada. [3]Shriners Hospitals for Children Canada, Montreal, Canada

### Abstract

**Background**: Perioperative anxiety is a major issue in pediatric surgery that causes increased pain, trauma, and surgical delays. Children with rare diseases such as Osteogenesis imperfecta (OI; brittle bone disease) are at increased risk for harm due to the chronic nature of the condition and the need for multiple, repeated surgeries. Perioperative anxiety can be reduced through child-centric surgical education. A promising, unexplored avenue is the use of Virtual Reality (VR) software to help children understand complex surgical information and reduce anxiety. However, most existing VR software do not address children's comprehensive information needs.

**Purpose:** To explore how educational digital health resources can be created for children with OI by co-designing a VR software prototype with children, families, and healthcare providers to enhance children's surgical knowledge.

**Methods:** This study will use a three-phase, user-centred design led by children, families, and clinicians (Figure 1). All interviews and focus groups will be supported with artmaking and play to facilitate communication and engagement with children.

In **Phase I (Preliminary work)**, we will conduct a scoping review of existing VR software for children's surgical preparation.

In **Phase II (Preparation)**, we will convene a VR council (children with OI, family members, healthcare providers, childhood educators, software developers; n=13) to guide the research team throughout the study, finalize study materials, and discuss the preliminary VR prototype design via focus groups.

In **Phase III (Co-Design)**, the prototype will be co-designed via 4 to 6 iterative interviews and rapid prototyping cycles with 5 to 6 child, family members, and clinician participants per cycle (Figure 2).

**Implications**: In the end, a VR software prototype to help children grasp complex health information and manage perioperative anxiety will be created *with* end-users. The co-design process will allow us to create a novel, meaningful, and transformative learning experience; leverage the potential and scalability of VR; and fill a significant educational gap in children's surgery. The resulting VR software prototype will be a first step towards establishing a cohesive, comprehensive educational curriculum for children's surgery in Canada by setting the stage for future feasibility testing, randomized controlled trials, and educational VR innovations.

Disclosure

None declared

### P167

### The Co-Design and Co-Development of Engaging Personas to Optimize Transitional Care of Youth with Osteogenesis Imperfecta

Michele Frederike[1,2], Reggie Hamdy[1,2], Mitchell Bernstein[1,2], Laura Massochin[1], Nadia Narducci[1], Lori VandenBroek[1], Frank Rauch[1,2], Argerie Tsimicalis[1,2]

[1]Shriners Hospital for Children Canada, Montreal, Canada. [2]McGill Univeristy, Montreal, Canada

### Abstract

**Objectives:**

To address a significant gap in the transitional care of youth with osteogenesis imperfecta (OI), an online self-management and transitional care program is being co-designed and developed called Teens OI. Youth with OI has expressed a desire for more personas, reflective of them, to be included in Teens OI. Hence, this study objective was to co-design and develop a series of engaging personas with youth with OI.

**Methods:**

A user-centered design is currently underway, where 50 youth with OI are being recruited in person at an orthopaedic hospital and online via OI-networks. Participants are self-completing a Teens OI-branded persona worksheet, which consists of 18-open-ended questions. Interviews to follow, where participants will provide input on the persona prototypes and share further preferences for personas' personalities and motivations, daily life experiences, and aspirations. Worksheet data, followed by interview data will be analyzed using thematic analysis to identify key themes and patterns, and refine the Teens OI personas.

**Results:**

To date, 19 out of the 50 anticipated participants, ages 12 to 20 years, with OI Types I, III, IV, and V, have been recruited. Preliminary results suggest the personas' goals and interests should include music, sports, gaming, arts and creativity, and socialising with friends. They should showcase relationships, like friendship and family, as well as autonomy. Future aspirations should focus on mental and physical well-being, self-confidence, and personal achievement. The personas' concerns should reflect both personal and social challenges, such as physical limitations, daily tasks, motivation, and experiences of ableism or social exclusion. In addition to emotional and social support, they should also inspire improvements in health, accessibility, inclusion, and personal growth, addressing not only individual well-being but also social impact. These details will be need to be integrated into the persona's hobbies and interests.

**Conclusion:**

Youth with OI desire a connection to youth's stories to optimize their transitional care. Creation of engaging personas may aid to enhance user engagement; promote a stronger sense of connection to the content and community being developed; and offer a tailored transitional experience.

### Disclosure

None declared

### P168

### Bone Mechanoregulation is not Altered by Disease Severity and Sex in People with OI

Pedram Akhlaghi[1,2], Seyedmahdi Hosseinitabatabaei[1,2,3], Frank Rauch[1,4], Bettina Willie[1,2,3,5]

[1]Shriners Hospital for Children, Research Center, Montreal, Canada. [2]Faculty of Dental Medicine and Oral Health Sciences, McGill University, Montreal, Canada. [3]Department of Bioengineering, McGill University, Montreal, Canada. [4]Department of Pediatrics, McGill University, Montreal, Canada. [5]College of Veterinary Medicine, Utah State University, Logan, Utah, USA

### Abstract

### Objectives:

Bone continually forms and resorbs to adapt its structure in response to the local mechanical environment. It remains unclear if bone mechanoregulation is altered in individuals with osteogenesis imperfecta (OI). In this study, we combined timelapse high-resolution peripheral quantitative computed tomography (HR-pQCT) with finite element modeling, to investigate how local bone mechanoregulation is affected by OI severity (type I vs type III or IV) and sex.

### Methods:

Forty-nine (N=49) tibial HR-pQCT scans from female (N=32, ages 20–73) and male (N=17, ages 19–66) participants with confirmed COL1A1/COL1A2 mutations (OI type I, N=32; type III or IV, N=17) were analyzed. In the absence of a nontreated group, we examined bone mechanoregulation in participants received a monthly low dose of setrusumab (2 mg/kg) as part of the ASTEROID trial (NCT03118570), a sclerostin-neutralizing antibody, at baseline and 12 months. First, 3D image registration identified local bone formation and resorption sites. Strain energy density (SED) was quantified and (re)modeling events were mapped to local SED values to calculate the likelihood of bone formation, resorption, and quiescence at these different SED values.

### Results:

Two-way ANOVA analysis showed that sex did not have a significant effect on bone formation or resorption over the 12-month period. However, there was a trend of greater bone formation in participants with OI type III or IV compared to OI type I (p=0.09). This trend was also observed in regard to mechanoregulation, where we observed that as SED magnitudes rose, the probability of bone formation increased by an average of 5% in OI type I and 10% in OI types III or IV. However, bone had the highest probability (90%) to be quiescent in both high-SED and low-SED regions, regardless of OI severity and sex (Figure 1B).

### Conclusions:

Our data suggests that mechanoregulation in OI is not dependent on OI disease severity and sex. Also, bone was largely quiescent (unchanged), independent of disease severity (type I versus type III/IV) and sex. These findings correspond to prior observations that show smaller long bone circumference in people with OI, regardless of OI severity.

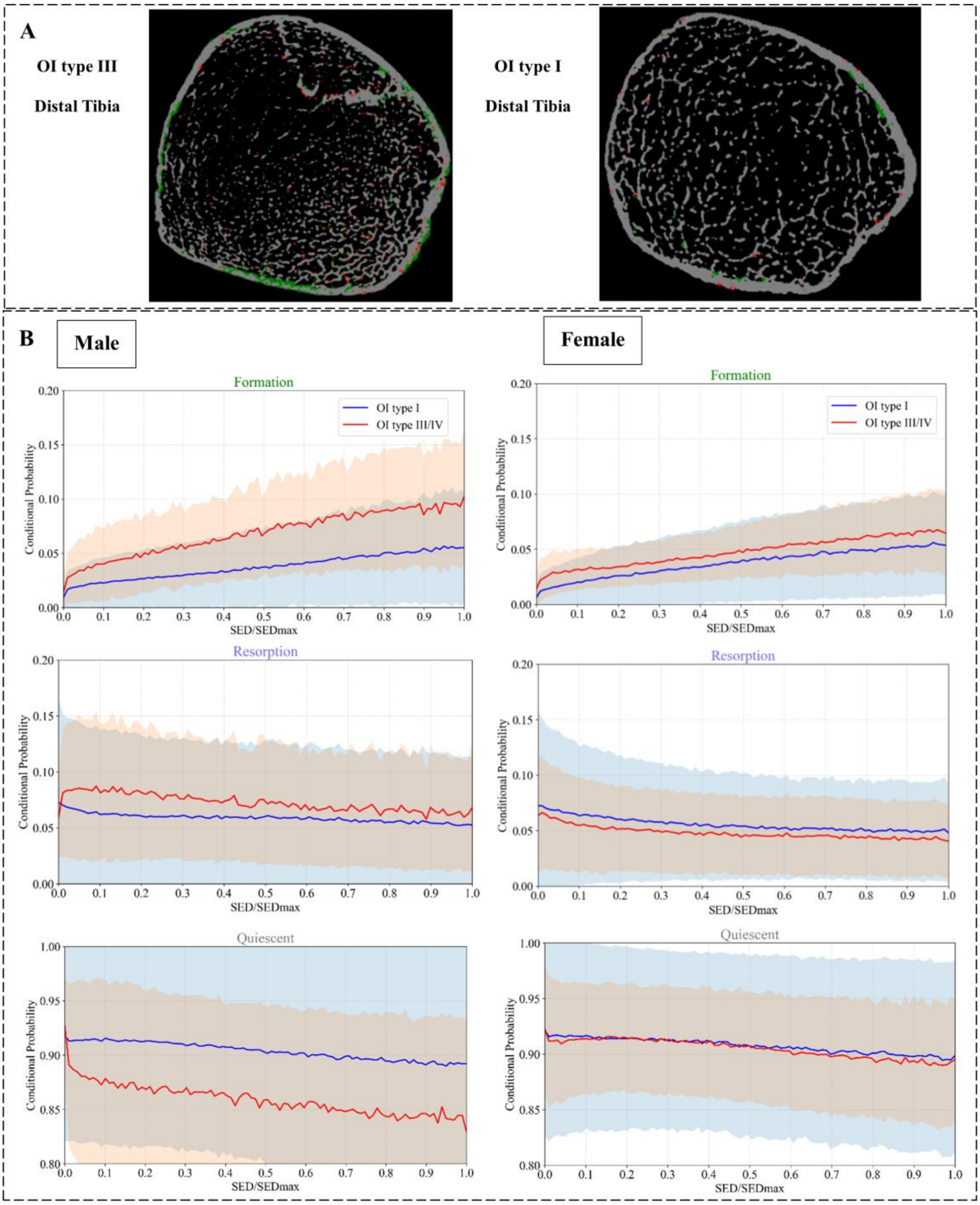

**Figure 1**: A one-year visualization of mechanoregulation in the human distal tibia. **A**: Regions of formed and resorbed bone obtained from timelapse analysis (**left**: OI type III and **Right**: OI type I). **B:** The likelihood curves for bone formation, resorption, and quiescence over 12 months of bone remodeling at varying local strain energy density magnitudes for male and female individuals with OI

### Disclosure

None declared

### P169

### A Phase 2/2b Study of Infigratinib in Children Under 3 Years Old with Achondroplasia: Design of PROPEL Infant and Toddler

Julie Hoover-Fong[1], Ravi Savarirayan[2], Melita Irving[3], Helen McDevitt[4], Supriya Raj[2], Rui Santos[3], Mars Skae[5], Klane White[6], Yun Bai[7], Snow Ge[7], Micaela Rodine[7], Karla Mehl[7], Daniela Rogoff[7]

[1]Johns Hopkins University, Baltimore, USA. [2]Murdoch Children's Research Institute, Parkville, Australia. [3]Guy's and St Thomas' NHS Foundation Trust, London, UK. [4]NHS Greater Glasgow and Clyde, Glasgow, UK. [5]Manchester University NHS Foundation Trust, London, UK. [6]Children's Hospital of Colorado, Aurora, USA. [7]BridgeBio Pharma, San Francisco, USA

### Abstract

### Objectives:

Infigratinib is an oral FGFR1-3 inhibitor in development for achondroplasia (ACH). In the PROPEL 2 study, daily infigratinib (0.25 mg/kg) was well tolerated and led to sustained increases in annualized height velocity and improved upper-to-lower body segment ratio in children aged 3 to 11 years old with ACH. These findings supported further evaluation of infigratinib in children under 3 years old with ACH.

### Methods:

PROPEL Infant and Toddler (NCT07169279) is a phase 2/2b study evaluating infigratinib in children aged 0–3 years with ACH confirmed by genetic testing. The study comprises 4 portions: (1) single ascending dose (SAD), (2) open-label phase 2, (3) double-blind, randomized, placebo-controlled phase 2b, and (4) open-label extension. Approximately 77 children will be enrolled. The SAD portion contains 4 sequential cohorts (Cohort 1: 2–<3 years; Cohort 2: 1–<2 years; Cohort 3: 6 months–<1 year; Cohort 4: 0–<6 months). Within each cohort, ascending doses will be evaluated based on safety and pharmacokinetics to identify the infigratinib dose for phase 2. Each subsequent descending age cohort will open after the prior cohort completes evaluation of all doses and the phase 2 dose has been selected. Participants of completed SAD cohorts then proceed to phase 2 and receive daily infigratinib at the identified dose for 52 weeks. If the phase 2 dose is deemed acceptable, the corresponding phase 2b cohort will commence. Primary endpoints for phase 2b will be safety and change from baseline to week 52 in body length z-score. Developmental milestones will be assessed, and MRIs will be performed to evaluate the potential effects on the foramen magnum. Participants <3 years after completing 52 weeks of treatment in phase 2 or phase 2b will receive open-label treatment with infigratinib until 3 years of age when they (and participants >3 years at study conclusion) will be offered to continue treatment in the ongoing PROPEL OLE study.

### Results:

Recruitment is ongoing at 13 sites across 7 countries, and the first participant was enrolled in November 2025.

### Conclusions:

This study will determine the appropriate dosing and evaluate the safety and efficacy of infigratinib for infants and toddlers with ACH.

### Disclosure

Julie Hoover-Fong has received grants or contracts for clinical trials from BioMarin, BridgeBio Pharma and Pfizer, has received consulting fees from BioMarin, BridgeBio Pharma, Ascendis, Tyra and Novo Nordisk, has received honoraria from Medscape and BioMarin, and has participated on advisory boards with MCDS-Therapy Clinical Trial (unpaid), BioMarin, and BridgeBio Pharma. Ravi Savarirayan has received consulting fees and grants from BioMarin, has participated on advisory boards with Ascendis and BioMarin, and has received consulting fees from BridgeBio Pharma. Melita Irving has received honoraria and travel-associated expenses from BridgeBio Pharma, BioMarin, Ascendis Pharma, and Tyra Biosciences. Helen McDevitt has served as a Principal Investigator for Amgen, Ascendis Pharma, BioMarin, and BridgeBio Pharma, and as a speaker for Kyowa Kirin. Supriya Raj has received consulting fees from BridgeBio Pharma. Rui Santos declares no conflicts of interest. Mars Skae has served as a consultant for and received travel support from BridgeBio Pharma, Sanofi and Rhythm Pharmaceuticals, and received support for conference registration from Pfizer previously. Klane White has received research contracts from Ascendis Pharma, BridgeBio Pharma, Tyra Biosciences, and Ultragenyx, has received consulting fees and honoraria from BioMarin and BridgeBio Pharma, and serves as a medical board member for Little People of America, a scientific advisory board member for the National MPS Society, and a member of the board of directors for Camp Korey. Yun Bai, Snow Ge, Micaela Rodine, Karla Mehl, and Daniela Rogoff are employees and shareholders of BridgeBio Pharma.

### P170

### The ACCEL Observational Study: Diagnostic Features, Medical History and Baseline Characteristics of Children with Hypochondroplasia

Andrew Dauber[1], Josep de Bergua[2], Paul Arundel[3], Philippe Campeau[4], Benjamin Cocanougher[5], Thomas Edouard[6], Svein Fredwall[7], Daniel Hoernschemeyer[8], Henrik Irgens[9], Saumya Jamuar[10], Janet Legare[11], Helen McDevitt[12], Marie-Eve Robinson[13], Massimiliano Rossi[14], Hind Al Saif[15], Mars Skae[16], Sérgio Sousa[17], Leanne Ward[13], Scott Ward[18], Klane White[19], Liza Bakunina[20], Elena Muslimova[20], Wanmei Wang[20], Katrina Wheeler[20], Melita Irving[21], Julie Hoover-Fong[22], Ravi Savarirayan[23]

[1]Children's National Hospital, Washington, DC, USA. [2]Vithas Hospital San Jose, Vitoria-Gasteiz, Spain. [3]Sheffield Children's NHS Foundation Trust, Sheffield, UK. [4]Centre Hospitalier Universitaire Sainte-Justine, Montreal, Canada. [5]Cincinnati Children's Hospital, Cincinnati, USA. [6]Children's Hospital, Toulouse University Hospital, Toulouse, France. [7]Oslo University Hospital, Oslo, Norway. [8]University of Missouri, Columbia, USA. [9]Haukeland University Hospital, Bergen, Norway. [10]KK Women's and Children's Hospital, Singapore, Singapore. [11]University of Wisconsin School of Medicine and Public Health, Madison, USA. [12]NHS Greater Glasgow and Clyde, Glasgow, UK. [13]Children's Hospital of Eastern Ontario, University of Ottawa, Ottawa, Canada. [14]Hôpital Femme Mère Enfant, Hospices Civils de Lyon, Lyon, France. [15]UCSF Benioff Children's Hospital, Oakland, USA. [16]Manchester University NHS Foundation Trust, Manchester, UK. [17]Hospital Pediátrico, Centro Hospitalar e Universitário de Coimbra, Coimbra, Portugal. [18]Vanderbilt University Medical Center, Nashville, USA. [19]Children's Hospital of Colorado, Aurora, USA. [20]BridgeBio Pharma, San Francisco, USA. [21]Myriad Trials, London, UK. [22]Johns Hopkins University, Baltimore, USA. [23]Murdoch Children's Research Institute, Parkville, Australia

### Abstract

### Objectives:

Hypochondroplasia (HCH) is an FGFR3-driven skeletal dysplasia characterized by disproportionate short stature. The ACCEL study (NCT06410976) aims to contribute to the characterization of HCH through documentation of its natural history. Here, we present initial findings on the diagnostic features, medical history, and baseline characteristics of children enrolled in ACCEL.

### Methods:

ACCEL is a global, prospective, observational study actively enrolling children aged 2.5 to <17 years old with the potential to grow and a clinically documented diagnosis of HCH confirmed by a molecular test. HCH-related clinical features were recorded at diagnosis. Medical history included events and conditions occurring prior to signing of the informed consent form and was coded by system organ class. Baseline characteristics were collected at screening. Participants completing at least 6 months in ACCEL may be eligible to enroll in interventional studies with infigratinib.

### Results:

As of 02-Sep-2025, 25 sites across Asia, Australia, Europe, and North America are active. Of the 64 participants enrolled, 36 (56.3%) were male, with a median (range) age at screening of 6.3 years (2.5–13.2). A total of 9 distinct *FGFR3* pathogenic variants were reported, and the most prevalent was N540K (n=55, 85.9%). Among participants, 24 (37.5%) were <1 year of age at diagnosis. The most common HCH-related diagnostic features were short stature (n=57, 89.1%), rhizomelic or mesomelic shortening of extremities (n=48, 75.0%), and macrocephaly (n=40, 62.5%). A history of surgical or medical procedures was reported in 22 (34.4%) participants, the most common of which was adenoidectomy (n=14, 21.9%). Previous infections were documented in 20 (31.3%) participants, with otitis media being the most frequent (n=14, 21.9%). Obstructive sleep apnea syndrome (n=8, 12.5%) accounted for most of the recorded respiratory disorders (n=19, 29.7%). Nervous system disorders were noted in the medical history of 23 (35.9%) participants, most frequently developmental speech disorders (n=9, 14.1%) and seizures (n=8, 12.5%), with one additional case of febrile convulsion (n=1, 1.6%). Attention deficit hyperactivity disorder and autism spectrum disorder were diagnosed in 11 (17.2%) and 3 (4.7%) participants, respectively.

**Conclusions:**

Collectively, these results highlight the clinical and genetic variability of HCH, underscoring the need for continued research to better understand the condition.

### Disclosure

Andrew Dauber has served as a consultant for BioMarin, BridgeBio Pharma, Novo Nordisk, Ascendis and Pfizer, and has grant funding from Pfizer and BioMarin. Josep de Bergua declares no conflicts of interest. Paul Arundel's institution received research funding from BridgeBio Pharma and BioMarin for the conduct of studies in which he is serving and has served as Principal Investigator. Philippe Campeau declares no conflicts of interest. Benjamin Cocanougher declares no conflicts of interest. Thomas Edouard has received consultancy fees from BioMarin and Novo Nordisk. Svein Fredwall has participated in advisory boards and given presentations for BioMarin, Sanofi, Ascendis Pharma, BridgeBio Pharma, and Novo Nordisk, honoraria have been paid to his institution and he is a principal investigator in ongoing clinical trials for BridgeBio Pharma and Ascendis Pharma. Daniel Hoernschemeyer has received research support from Ascendis Pharma, BioMarin, and BridgeBio Pharma for the conduct of studies in which he served as a site Principal Investigator, is a royalty stockholder of OrthoPediatrics, and has served as a faculty speaker for Highridge Medical. Henrik Irgens declares no conflicts of interest. Saumya Jamuar declares no conflicts of interest. Janet Legare has served as a site Principal Investigator for Ascendis Pharma and BridgeBio Pharma, as a paid speaker for Ascendis Pharma, BioMarin, and BridgeBio Pharma, and as an advisory board member for BioMarin and Ascendis Pharma. Helen McDevitt has served as a Principal Investigator for Amgen, Ascendis Pharma, BioMarin, and BridgeBio Pharma, and as a speaker for Kyowa Kirin. Marie-Eve Robinson has received research support from Ascendis Biopharma, Ipsen Biopharmaceuticals, and BridgeBio Pharma, and has served as a consultant for Ultragenyx, BioMarin, and Ipsen Biopharmaceuticals. Massimiliano Rossi has served as a Principal Investigator for clinical trials sponsored by BioMarin and BridgeBio Pharma, has received travel support from BioMarin and BridgeBio Pharma, has participated in advisory board activities for BioMarin, and his institution has received payment for speaking engagements from BioMarin. Hind Al Saif declares no conflicts of interest. Mars Skae has served as a consultant for and received travel support from BridgeBio Pharma, Sanofi and Rhythm Pharmaceuticals, and received support for conference registration from Pfizer previously. Sérgio Sousa has received honoraria for lectures, advisory board participation, and/or travel support from BioMarin, Ascendis Pharma, and Kyowa Kirin, and has served as an investigator in observational studies and/or clinical trials for Pfizer, Ascendis Pharma, BioMarin, and BridgeBio Pharma. Leanne Ward's institution received research support from Ascendis Pharma and BridgeBio Pharma for studies in which she served as a Principal Investigator, and consultancy fees from BioMarin and BridgeBio Pharma. Scott Ward has received consultancy fees from BridgeBio Pharma. Klane White has received research contracts from Ascendis Pharma, BridgeBio Pharma, Tyra Biosciences, and Ultragenyx, has received consulting fees and honoraria from BioMarin and BridgeBio Pharma, and serves as a medical board member for Little People of America, a scientific advisory board member for the National MPS Society, and a member of the board of directors for Camp Korey. Liza Bakunina, Elena Muslimova, Wanmei Wang, and Katrina Wheeler are employees and shareholders of BridgeBio Pharma. Melita Irving has received honoraria and travel-associated expenses from BridgeBio Pharma, BioMarin, Ascendis Pharma, and Tyra Biosciences. Julie Hoover-Fong has received grants or contracts for clinical trials from BioMarin, BridgeBio Pharma and Pfizer, has received consulting fees from BioMarin, BridgeBio Pharma, Ascendis, Tyra and Novo Nordisk, has received honoraria from Medscape and BioMarin, and has participated on advisory boards with MCDS-Therapy Clinical Trial (unpaid), BioMarin, and BridgeBio Pharma. Ravi Savarirayan has received consulting fees and grants from BioMarin, has participated on advisory boards with Ascendis and BioMarin, and has received consulting fees from BridgeBio Pharma.

### P171

### Challenges of Peri-Transplant Management of TCIRG1-Related Autosomal Recessive Osteopetrosis Complicated by Rickets

Maria Zhadina[1], Deepika Burkardt[1], David Jacobsohn[1], Christina Grant[1], Dorothy Bulas[1], Carlos Ferreira[2], Laura Tosi[1]

[1]Children's National Hospital, Washington, DC, USA. [2]National Institutes of Health, Bethesda, MD, USA

### Abstract

### Background

Autosomal recessive osteopetrosis (ARO) is a rare disabling disorder of osteoclast differentiation/function, leading to impaired bone resorption. The resulting dense bone is prone to fracture, and narrowing of cranial nerve foramina and intramedullary spaces leads to severe complications, including vision/hearing loss, bone marrow failure, infection and hemorrhage. Hematopoietic stem cell transplantation (HSCT) is currently the only available clinical treatment for osteoclast-rich forms of osteopetrosis.

*TCIRG1* inactivating variants account for about half of ARO cases presenting in early childhood. Rickets/osteomalacia are common in *TCIRG1*-related ARO, due to impaired gastric acidification, calcium malabsorption, and calcium sequestration in dense bone. Treatment of osteomalacia before HSCT is thought to be essential for therapeutic effect, as donor osteoclasts are not active on unmineralized osteoid.

### Presenting problem

We report the case of an 18-month-old girl who presented at 6 months of age with nystagmus, relative macrocephaly, bulging fontanelles, and poor growth. Brain imaging demonstrated hydrocephalus, narrow posterior fossa and cranial nerve foramina, and sclerotic skull base. Skeletal radiographs showed diffuse sclerosis, bone-within-bone vertebral appearance, and metaphyseal flaring and cupping. Labs showed anemia, thrombocytopenia, mild hypocalcemia (8.5 mg/dL), hypophosphatemia (2.7 mg/dL), elevated alkaline phosphatase (1178 unit/L), deficient 25-hydroxyvitamin D (18.3 ng/ml), and mildly elevated parathyroid hormone (PTH, 106 pg/ml).

### Clinical Management

Rapid Trio whole exome sequencing revealed biallelic pathogenic variants in *TCIRG1* [c.447dup; p.(E150Rfs*77) and c.1674-1 G>A p.(?)]. Rickets was aggressively treated with cholecalciferol, calcium, phosphate, and calcitriol. Alkaline phosphatase declined to 569 unit/L, but hypophosphatemia and metaphyseal abnormalities remained unchanged after seven weeks on treatment, when she underwent haploidentical HSCT. Post-transplant course was complicated by cytokine release syndrome, sinusoidal obstruction syndrome, thrombotic microangiopathy, graft-versus-host disease, and renal dysfunction requiring dialysis. Her minerals, alkaline phosphatase, and PTH levels normalized, and radiographs demonstrated rickets resolution with normal new bone formation by 2 months post-transplant.

### Discussion

This case underscores the challenges of peri-transplant management of *TCIRG1*-related osteopetrorickets. Early transplantation is necessary to reduce mortality and prevent irreversible neurologic injury, but correction of rickets prior to HSCT is generally recommended. This report demonstrates that incomplete correction of rickets and hypophosphatemia pre-transplant does not preclude effective donor osteoclast-mediated bone remodeling.

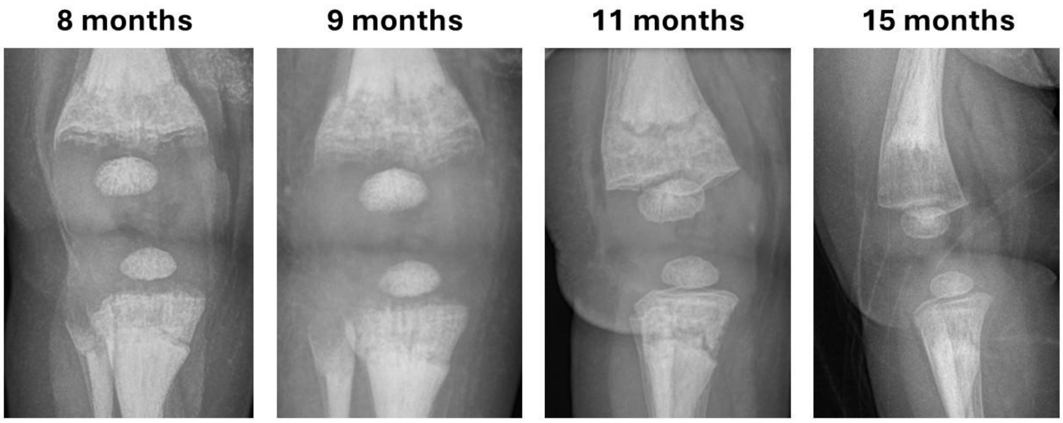

Figure 1. Sequential radiographs of the right knee.
At time of diagnosis at 8 months of age, the diaphyses are unusually sclerotic with irregular striations of the osteopenic metaphyses with physeal cupping and flaring. No improvement following the first weeks of therapy with cholecalciferol, calcium, phosphate, and calcitriol (9 months). At 2-6 months post-transplant (11-15 months of age), the cupping and flaring of the physis have improved with residual metaphyseal osteopenia and periosteal thickening.

### Disclosure

None declared

### P172

### The Rate of Children with Achondroplasia not Responding to Vosoritide – Real World Data from the Czech Reference Center for Rare Bone Diseases

Ondrej Soucek, Klara Maratova, Dana Zemkova, Zdenek Sumnik

Department of Pediatrics, Second Faculty of Medicine, Charles University, University Hospital Motol and Homolka, Prague, Czech Republic

### Abstract

**Objectives:**

Achondroplasia is characterized by markedly disproportionate short stature. So far, the only approved targeted systemic pharmacological treatment, vosoritide, has been widely used to promote growth in achondroplasia. Despite that most individuals respond very well to the treatment, few don't. Understanding this group of patients is crucial to provide individualized treatment options. We report the rate of non-responders in the first two years after vosoritide commencement.

**Methods:**

Children and adolescents with achondroplasia sent for vosoritide treatment at our referral center were consecutively included in this prospective longitudinal observational study. Achondroplasia was genetically confirmed and other bone diseases were excluded in all patients. Hand x-ray was done in each patient to manually assess their bone age. Vosoritide dosing was guided based on the SPC. Clinically significant response to vosoritide was defined as height Z-score increase of > 0.2 / year.

**Results:**

Since April 2022, 43 children (17 girls) aged 0.76-14.6 years (mean 6.6±4.0 years) have been treated with vosoritide. Six have not yet finished the first year, and two were cross-border patients who continued to be treated in their home countries before finishing the first year. From the remaining 35 children, four (11 %) failed to respond to vosoritide treatment. From those who have already finished 24-months treatment period (N=28), only one child (4 %) failed to respond to the treatment. Two of the 4 non-responders at year 1 presented with sufficient growth (i.e., height Z-score increase by > 0.4) at year 2, in one the treatment was withdrawn because of funder-established lack of efficiency, and one failed to show sufficient growth also at year 2. No safety issues were seen besides transient hypertrichosis on the back in two patients.

**Conclusions:**

Vosoritide is a growth promoting medicine with huge potential to improve quality of life of patients with achondroplasia. However, approximately 1:10 patients fail to show clinically significant increase in growth velocity after the first year of treatment. Despite that some of these patients might increase their growth significantly in the second year of treatment, other medicines than vosoritide are needed to help also the non-responders to improve their quality of life.

### Disclosure

None declared.

### P173

### Clinical Features of Mother and Daughter Cases of Osteogenesis Imperfecta with Atypical *IFITM5* Variants

Taichi Kitaoka[1], Hiroyuki Saitou[2], Tatsuya Nakamichi[1], Akio Nakura[2], Keiichi Ozono[2]

[1]Department of Pediatrics, ISEIKAI international General Hospital, Osaka, Japan. [2]Center for Promoting Treatment of Intractable Diseases, ISEIKAI International General Hospital, Osaka, Japan

### Abstract

BACKGROUND:

Interferon-induced transmembrane protein 5 (*IFITM5*) is known to be the causative gene for Osteogenesis imperfecta (OI) type V, characterized by interosseous membrane calcification and hyperplastic calluses as well as bone fragility. Most of reported pathogenic variant is c.-14C>T in the 5' UTR of *IFITM5*, however there are several reports on variants in the coding region of *IFITM5*, including c.119C>T (p.Ser40Leu), leading to different skeletal findings from OI type V. We present a long-term follow-up of a Japanese mother and daughter with the c.143A>G (p.Asn48Ser) variant.

PRESENTING PROBLEM:

The index patient is a 23-year-old female with a heterozygous *IFITM5* variant (c.143A>G, p.Asn48Ser). Throughout childhood, she experienced multiple long-bone fractures requiring surgical intervention but never exhibited hypertrophic calluses or interosseous calcification. Since age 18, she has developed progressive hearing loss requiring bilateral hearing aids. Her 52-year-old mother with the same variant presented short stature (132cm) and scoliosis and experienced multiple fractures phenotype, including the absence of the typical OI type V findings, but both patients possess distinctive calvarial bumps with central radiolucency (Calvarial Doughnut Lesions; CDLs).

CLINICAL MANAGEMENT:

Both patients have undergone multiple intramedullary nailing and corrective surgeries for long-bone deformities and fractures. Current management is supportive, focusing on rehabilitation, orthotics for limb discrepancy, and auditory support. Neither patient has received bisphosphonate therapy to date.

DISCUSSION:

The reported patients with variants in the coding region of *IFITM5* have presented different skeletal phenotype from typical OI type V with variant in the 5’ UTR. And p.Asn48Ser results in a distinct phenotype mimicking Osteoporosis with Calvarial Doughnut Lesions (OP-CDL), typically associated with *SGMS2*, encoding sphingomyelin synthetase 2. It suggests that *IFITM5* coding-region variants might disrupt cranial and skeletal mineralization in a manner similar to *SGMS2* mutations. In our patient, this follow-up reveals that progressive hearing loss, a feature common in other OI types but small numbers of patients with OI type V, can manifest in early adulthood with the p.Asn48Ser variant. Our findings may provide insight into the role of *IFITM5*.

### Disclosure

None declared

### P174

### MyAchonJourney: An Online Educational Resource for Individuals with Achondroplasia and their Families, Developed by Advocacy Leaders and Healthcare Providers

Colleen Gioffreda[1], Chandler Crews[2], Kristen DeAndrade[3], Kim Fine[4], Anne Grumet[5], Kirsten Kiefer[5], Deborah Krakow[6], William Mackenzie[7], Alisa Santiesteban[5], Munira Shamim[8], Mari Groves[9]

[1]The Alan and Kathryn Greenberg Center for Skeletal Dysplasias, Johns Hopkins University, Baltimore, USA. [2]The Chandler Project, Little Rock, USA. [3]The Little Legs Big Heart Foundation, West Palm Beach, USA. [4]Baptist Health South Florida, Coral Gables, USA. [5]BridgeBio Pharma, San Francisco, USA. [6]David Geffen School of Medicine, University of California, Los Angeles, Los Angeles, USA. [7]Nemours Children's Hospital, Wilmington, USA. [8]Growing Stronger, Saratoga, USA. [9]The Johns Hopkins Hospital, Baltimore, USA

### Abstract

**Objectives:** Achondroplasia is a skeletal dysplasia arising from a gain-of-function pathogenic variant in *FGFR3* that suppresses bone growth and leads to disproportionate short stature. Individuals with achondroplasia and their families often navigate potential medical complications and psychosocial challenges that can accompany the condition. However, a centralized and accessible location for educational resources to support them has remained a significant unmet need, especially for newly diagnosed families. Here, we describe the development and impact of MyAchonJourney, a one-stop online resource co-developed by the community and healthcare providers (HCPs), to address this need.

**Methods:** A task force of 4 community advocacy leaders and 5 multidisciplinary HCPs was assembled and convened in October 2023 to determine the most useful format and content of educational resources for newly diagnosed individuals with achondroplasia and their families. At the meeting, there was consensus that an interactive website organized by age group (prenatal, infancy and toddlerhood, and early childhood) that offered positive and empowering resources for families was most needed. The task force agreed that resources for managing complications, promoting independence and acceptance, and fostering connections with community support groups should be included. The recommendations from the task force provided the initial framework for MyAchonJourney, and continued collaboration over 10 months led to the refinement and completion of the final website.

**Results:** The MyAchonJourney website was launched in August 2024 and followed the recommended organization of prenatal (before birth), infancy and toddlerhood (birth to 2 years), and early childhood (3 to 5 years). Within each age group, key information included education on common medical challenges, home and school accommodations, and psychosocial support to help children understand their condition in a confident and healthy manner. Downloadable guides for prenatal testing, medical visits, infant care, and supplemental oxygen use were also created. Further details on the content developed for MyAchonJourney, data on resource usage, and community feedback will be presented.

**Conclusions:** Positive feedback and high engagement from the community and HCPs have validated the utility of MyAchonJourney. Plans to expand the website to offer resources for adolescents and adults with achondroplasia are ongoing.

### Disclosure

Colleen Gioffreda declares no conflicts of interest. Chandler Crews declares no conflicts of interest. Kristen DeAndrade has participated on advisory boards for BioMarin and BridgeBio Pharma, and attended meetings with BioMarin, BridgeBio Pharma, Ascendis Pharma, and Tyra Biosciences. Kim Fine declares no conflicts of interest. Anne Grumet, Kirsten Kiefer, and Alisa Santiesteban are employees and shareholders of BridgeBio Pharma. Deborah Krakow declares no conflicts of interest. William Mackenzie has served as a consultant for BioMarin and BridgeBio Pharma. Munira Shamim declares no conflicts of interest. Mari Groves declares no conflicts of interest.

### P175

### A Case Series of Rothmund Thompson Syndrome Type 2

Anju Jacob[1,2], Nadia Shaukat[1], Layla AlMarzooqi[1], Mohamed Elabiary[1], M Zulf Mughal[1]

[1]Al Jalila Childrens specialty hospital, Dubai, UAE. [2]Mohammed Bin Rashid University of Medicine and Health Sciences, Dubai, UAE

### Abstract

**Background**
Rothmund-Thomson syndrome type 2 (RTS2) is a rare autosomal recessive disorder caused by biallelic pathogenic variants in RECQL4. Phenotypic hallmarks include poikiloderma, proportionate short stature, sparse hair, skeletal dysplasias, and a significantly elevated risk for osteosarcoma. Additionally, osteopenia and increased fracture susceptibility are prominent but poorly characterised as features of the condition.

**Clinical Series**
We describe three pediatric patients (aged 13–16 years) with genetically confirmed RTS2 (RECQL4 homozygous mutations) referred to pediatric endocrinology for evaluation of short stature and recurrent fractures.

Patient A (15y male): Presented with severe short stature (height SDS -6.8) and underweight , extensive poikiloderma, and a history of multiple long-bone fractures refractory to regular zoledronic acid (ZA) infusions. He developed chronic osteomyelitis and a non-union of his left tibia despite intramedullary fixation, ultimately becoming wheelchair-bound.

Patient B (13y female): Presented with severe short stature, extensive poikiloderma, and recurrent fractures. She showed a positive clinical response to a reduced-frequency ZA regimen (annually vs. biannually), with no new fractures for over one year following bilateral femoral nailing. She also had severe insulin resistance and was treated with dupilumab for poikiloderma. She was also found to have long QT syndrome on cardiac evaluation, as her younger sister who was diagnosed with the same RTS2 `passed away suddenly at the age of 10 years.

Patient C (16y female): Notably fracture-free and without poikiloderma. She had a complex concurrent medical history including Graves' disease, Hyper-IgM syndrome (due to a homozygousUNGmutation), and secondary hemophagocytic lymphohistiocytosis (HLH).

**Discussion**
This series highlights the significant phenotypic variability and management challenges in RTS2. While some patients (e.g., Patient B) may benefit from long-term, low-frequency bisphosphonate therapy, others (e.g., Patient A) demonstrate profound bisphosphonate resistance and poor response. The absence of poikiloderma and fractures in Patient C underscores the influence of genetic modifiers and/or allelic heterogeneity on the skeletal phenotype. A bone biopsy for histomorphometric analysis is warranted in these patients to better define the underlying skeletal pathology better and guide targeted therapy. Regular DEXA scans remain essential for routine bone health surveillance. It is also essential to remember that these children need sun protection, given the increased risk of photosensitive rash, and need monitoring for osteosarcoma.

### Disclosure none declared

### P176

### DXA Phenotype and Fracture Risk in Children with Osteogenesis Imperfecta Type 1 – a Longitudinal Cohort Study from Western Australia

Kiranjit Joshi[1,2], Aris Siafarikas[1,2], Richard Prince[2]

[1]Perth Children's Hospital, Perth, Australia. [2]University of Western Australia, Perth, Australia

### Abstract

**Objective:**

The relationship between bone structure and skeletal fragility with the DXA-derived measures of total body less head (TBLH), height (ht-for-age), bone area for height (BA-for-ht) and bone mineral content for bone area (BMC-for-BA) in osteogenesis imperfecta (OI) type 1 remains incompletely defined. Measures of lean mass (LBM-for-ht) and BMC-for-LBM provide insight into the functional muscle-bone unit and may contribute to fracture risk assessment.

**Methods:**

We examined these DXA parameters in a retrospective, observational cohort study, evaluating the total body less head (TBLH) and lumbar spine (LS) bone mineral density from 34 patients (F=17) mean age 10.3 years (95%CI: 9.7 to 10.9) for 157 clinical study year data points. The bisphosphonate naïve group were stratified into a low fracture risk group; (no recurrent fractures, no bisphosphonate N=13; 43 DXA) and a high fracture risk group: (recurrent fractures, prior to commencing bisphosphonate N=21; 34 DXA). The bisphosphonate group comprised of patients who received treatment (N=21; 83 DXA).

**Results:**

Amongst the bisphosphonate naïve patients, those in the high fracture risk group were significantly shorter (ht for age, p=0.002), had narrower bones (BA for ht, p<0.001) and had a marked deficit in their lean mass (LBM for ht, p<0.001) compared to the low fracture risk patients. Following initiation of bisphosphonate therapy these patients showed significant improvements in bone area (BA for ht, p<0.001), lean mass (LBM for ht, p=0.019) and BMC for LBM (p< 0.001). After adjusting for bisphosphonate therapy, TBLH BMD for Ht (p=0.021), BA for ht (p=0.038) and BMC for LBM for ht (p=0.016) were independently associated with fracture occurrence in the same year.

**Conclusion:**

Overall, our findings indicate that DXA-derived measures of bone structure, mineral content and lean mass, the DXA-phenotype, are informative for predicting fracture risk and for monitoring skeletal response to bisphosphonate therapy in children with OI type 1.

### Disclosure

None declared.

### P177

### Empowerment, Autonomy, and Self-Advocacy: Key Pillars for the Co-Design and Development of Teens Taking Charge: Managing Osteogenesis Imperfecta (OI) Online

Laura Massochin[1], Raissa Passos dos Santos[1,2], Jenny Wang[1,3], Jennifer Stinson[4,5], Frank Rauch[1,3], Sofia Addab[1,3], Khadidja Chougui[1,2], Reggie Hamdy[1,3], Telma Palomo de Oliveira[6], Noemi Dahan-Oliel[1,3], Louis-Nicolas Veilleux[1,3], Nadia Narducci[1], Lori VandenBroek[1], Jessica Collins[1], Corinne Mercier[1], Mitchell Bernstein[1,3], Kelly Thorstad[1], Argerie Tsimicalis[1,3]

[1]Shriners Hospitals for Children® – Canada, Montreal, Canada. [2]University of Montreal, Montreal, Canada. [3]McGill University, Montreal, Canada. [4]The Hospital for Sick Children, Toronto, Canada. [5]University of Toronto, Montreal, Canada. [6]Fleury Medicine and Healthcare, Sao Paulo, Brazil

### Abstract

### Objectives

The transition to adult healthcare systems poses significant challenges for youth with osteogenesis imperfecta (OI). The study objective was to understand the experiences of youth with OI during their transition to adult care to inform the ongoing co-design and co-development Teens Taking Children: Managing Osteogenesis Imperfecta Online (Teens OI).

### Methods

Situated with a user-center design aimed to co-design and co-develop Teens OI, a secondary qualitative analysis was conducted at a university-affiliated pediatric orthopedic hospital. The dataset included sociodemographic surveys, field notes, interviews, and focus groups derived from youth, parents and the study advisory council. Transcripts were analyzed using qualitative thematic analysis, involving familiarization, coding, theme development, and iterative refinement. Participants' perspectives were integrated, and visual exemplars were created to operationalize the identified pillars for Teens OI.

### Results

Participants included 10 youth with OI (ages 12–20; types I, III, IV), 6 parents, and 8 Council members (3 nurses, 1 social worker, 2 adults with OI, 1 parent, 1 youth). The three interconnected concepts: empowerment, self-advocacy, and autonomy, emerged as pillars guiding the co-design and co-development of Teens OI. Empowerment involved owning one's condition, gaining confidence, and using experiential knowledge to make informed decisions. Self-advocacy encompassed speaking up, negotiating, and mobilizing resources. Autonomy entailed using information to make independent decisions aligned with personal goals. These concepts informed exemplar Teens OI content such as the "OI Athletes" testimonials, "Pain Journal," "Quick Tips for Talking with Your Healthcare Team," and the "Pixel Spoons" game. The integration of these pillars aims to support youth in transitioning successfully to adult care.

### Conclusion

Transitioning from pediatric to adult care for youth with OI requires the development of empowerment, self-advocacy, and autonomy. These interconnected concepts support youth to gain control over their lives, make informed decisions, and advocate for their needs. Teens OI aims to integrate these pillars supporting youth's successful transition, assisting healthcare providers, and enhancing the overall quality of life for youth with OI.

### Disclosure

None declared

### P178

### Qualitative Research to Evaluate the Content Validity and Relevance of Patient-Reported Outcome Measures for Children and Parents of Children with Hypochondroplasia

Nadia Merchant[1], Liza Bakunina[2], Chandler Crews[3], Hilary Colwell[4], Kristen DeAndrade[5], Anne Grumet[2], Kirsten Kiefer[2], Susan Mathias[4], Alexandra Niemczura[6], Susana Noval Iruretagoyena[7], Melita Irving[8]

[1]University of Texas Southwestern Medical Center, Dallas, USA. [2]BridgeBio Pharma, San Francisco, USA. [3]The Chandler Project, Little Rock, USA. [4]Health Outcomes Solutions, Palm Beach Gardens, USA. [5]The Little Legs Big Heart Foundation, West Palm Beach, USA. [6]Parent Advocate, Cincinnati, USA. [7]Fundación ALPE Achondroplasia, Gijón, Spain. [8]Myriad Trials, London, UK

### Abstract

**Objectives:** Hypochondroplasia (HCH) is a skeletal dysplasia condition caused by pathogenic variants in *FGFR3* that alter endochondral bone growth and lead to disproportionate short stature. Clinical manifestations of HCH are variable, and data describing its impact on functioning and wellbeing remain limited. While certain patient-reported outcome (PRO) measures have been utilized in children with achondroplasia, the clarity and relevance of these tools for children with HCH has not been established. The objective of this qualitative research was to assess the content validity of four PRO measures for HCH.

**Methods:** This study evaluated PedsQL-SF-15, QoLISSY (Core), Patient Global Impression of Severity (PGI-S), and Patient Global Impression of Change (PGI-C) using concept elicitation (CE) and cognitive debriefing (CD) interviews conducted via Zoom with participants living in the United States. Eligible participants included English-speaking children aged 8–<18 years and parents of children aged 2–<18 years, with a confirmed molecular diagnosis of HCH. All interviews were recorded, transcribed, and coded using MAXQDA. Concepts identified during CE were mapped to PedsQL-SF-15, PGI-S, and PGI-C to assess concept coverage, and findings from CD were evaluated to determine the clarity and relevance of each questionnaire.

**Results:** From May 2025 to October 2025, 8 children (age range: 9–17 years) and 17 parents were interviewed. Among children, the most common medical or physical challenges were pain (50.0%), fatigue (37.5%), and ear infections (25.0%), whereas parents most commonly reported bowed legs (52.9%), ear infections (47.1%), and fatigue (47.1%). The most common self-reported impacts of HCH among children were challenges with reaching (87.5%), paying attention (62.5%), and remembering (50.0%), while parents most often noted challenges with reaching (88.2%), remembering (76.9%), and focusing (70.6%). Concept mapping showed that PedsQL-SF-15, PGI-S, and PGI-C covered most key concepts identified by children and parents, with some limitations related to signs and symptoms. Based on detailed CD, children and parents found all four PRO measures to be comprehensive, clear, and relevant.

**Conclusions:** These results demonstrate the content validity, clarity, and relevance of PedsQL-SF-15, QoLISSY (Core), PGI-S, and PGI-C for HCH. Future research may explore whether complementary tools, including streamlined approaches, could improve routine clinical assessment of HCH.

### Disclosure

Nadia Merchant has served on advisory boards for Alexion, Ascendis Pharma, BioMarin, BridgeBio Pharma, Catalyst, Kyowa Kirin, Ultragenyx and Pfizer. Liza Bakunina, Anne Grumet, and Kirsten Kiefer are employees and shareholders of BridgeBio Pharma. Chandler Crews reports no conflicts of interest. Susan Mathias is an employee and Hilary Colwell is a consultant to Health Outcomes Solutions, which received funding from BridgeBio Pharma for undertaking this research. Kristen DeAndrade has participated on advisory boards for BioMarin and BridgeBio Pharma, and attended meetings with BioMarin, BridgeBio Pharma, Ascendis Pharma, and Tyra Biosciences. Alexandra Niemczura reports no conflicts of interest. Susana Noval Iruretagoyena reports no conflicts of interest. Melita Irving has received honoraria and travel-associated expenses from BridgeBio Pharma, BioMarin, Ascendis Pharma, and Tyra Biosciences.

### P179

### Quality of Life and Phenotypic Variability in Patients with Bruck Syndrome Due to *FKBP10* and *PLOD2* Mutations: Insights from a UAE Cohort and Systematic Literature Review

Mohammad Hosny Awad[1,2], Asmahan Tagelsir Abdalla[3], Ibrar Majid[4], Mohamed Kenaway[4], Sattar Alshryda[4], Manal Mustafa[1], Sarah Ehtisham[1], Asma Deep[3], M Zulf Mughal[1]

[1]Pediatric Endocrinology Department, Al Jalila Children's Specialty Hospital, Dubai, UAE. [2]Pediatric Department, Mansoura University, Mansoura City, Egypt. [3]Pediatric Endocrine Division, Sheikh Shakhbout Medical City, Abu Dhabi, UAE. [4]Orthopedic Department, Al Jalila Children's Specialty Hospital, Dubai, UAE

### Abstract

**Background:**
Bruck syndrome is a rare and severe form of osteogenesis imperfecta caused by mutations affecting collagen folding and cross-linking, most commonly *FKBP10* and *PLOD2*. It is classically characterized by bone fragility and congenital joint contractures; however, the full phenotypic spectrum, inter-individual variability, and impact on quality of life remain incompletely understood.

**Objective:**
To characterize clinical features and health-related quality of life in patients with genetically confirmed Bruck syndrome and to contextualize these findings through a systematic review of the published literature.

**Methods:**
Thirteen patients with genetically confirmed Bruck syndrome from two tertiary centres in the United Arab Emirates were evaluated for genotype, fracture history, mobility, and spinal involvement. Health-related quality of life was assessed using the Pediatric Quality of Life Inventory (PedsQL). Institutional findings were integrated with a systematic analysis of 149 previously reported cases.

**Results:**
*FKBP10* mutations predominated in the institutional cohort, with a single recurrent variant accounting for most cases. Despite identical genotypes, marked phenotypic variability was observed, including substantial differences in disease severity, mobility, and daily functioning, even among affected family members. While fractures were frequent, a subset of young children presented primarily with joint stiffness rather than fractures. Mobility ranged from full ambulation to complete non-ambulation. Spinal deformities were commonly observed.

Quality-of-life assessments revealed severe impairment in physical functioning, particularly mobility and endurance. Social participation was moderately affected, whereas emotional well-being and school functioning were relatively preserved.

**Conclusion:**
Bruck syndrome due to *FKBP10* and *PLOD2* mutations is associated with profound phenotypic and functional heterogeneity extending beyond bone fragility alone. The extreme variability observed among individuals with identical mutations suggests the influence of genetic or environmental modifiers. These findings underscore the importance of individualized, multidisciplinary management strategies focused on fracture prevention, mobility support, and long-term functional outcomes.

Disclosure

None declared

### P180

### A Mouse Model Expressing the Osteogenesis Imperfecta Type V Variant (c.-14C>T ) in Chondrocytes Leads to Neonatal Lethality

Pierre Moffatt[1,2], Sohan Rahaman[1]

[1]Shriners Hospitals for Children - Canada, Montreal, Canada. [2]McGill University, Montreal, Canada

### Abstract

OBJECTIVES:

*IFITM5* is an osteoblast-specific gene that encodes the BRIL protein. A recurrent variant in *IFITM5* (c.-14C>T) causes autosomal dominant osteogenesis imperfecta (OI) type V, characterized by hyperplastic callus formation and interosseous mineralization. The underlying mechanisms causing OI type V remain unclear, although aberrant signaling through ERK-SOX9 has been proposed. Here we used our conditional c.-14C>T mouse model to study the consequences of its expression in chondrogenic cells and to characterize the skeletal phenotype.

METHODS:

Mice harboring a conditional floxed c.-14C>T allele (Fl/+) were crossed to a Col2-Cre driver to ensure expression in chondrocytes. The Fl/+:Col2Cre+ mutant mice were compared to control littermates. Neonatal pups and embryos were collected for phenotyping using whole skeletal staining. Limbs and rib cages were processed for histology, immunohistochemistry, western blotting, and RNA extraction for RT-qPCR.

RESULTS:

All mutant Fl/+:Col2Cre+ pups were found dead at birth. Analysis revealed severe malformed and frail rib cages as compared to control littermates. The dorsal segments of the mutant ribs appeared whitish, wavy, and consistently displayed signs of fractures. Histopathological analysis demonstrated an apparent lack of mineralized cortices in the dorsal rib cage segment. Mutant ribs lacked a bone marrow cavity, which was filled with chondrocytes. The femur-tibia looked apparently normal but presented with occasional curvatures. The growth plates architecture and its chondrocyte composition appeared unaffected. Histological analysis of the tibia indicated regionalized cortical thinning, accompanied by an abnormal chondrogenic cellular composition, that was positive for SOX9. Gene expression analysis in embryonic day 18.5 dorsal ribs revealed no significant change in *Bril* expression, reduced levels of *Bglap*, but significant upregulation of *Acan*, *Sox9*, *Ptgs2*, and *Nr4a3*. In the femur-tibia, *Ptgs2* and *Nr4a3* levels were also augmented, with little changes in the other target genes.

CONCLUSIONS:

Our results suggest that Col2-Cre mediated expression of the MALEP-BRIL protein in chondrocytes has a major impact on rib cage development, leading to neonatal death. Long bones are relatively spared with no gross disturbance in the growth plates, but presenting a subtle phenotype on the periosteum, with SOX9+ chondrocytes instead of mature osteoblasts. Gene expression monitoring indicated that both tissues present with a pro-inflammatory signature (*Ptgs2*, *Nr4a3*).

### Disclosure

None declared

### P181

### Vertebral Abnormalities in a Child with Dual PDE4D and ALPL Mutations: A Case of Overlapping Acrodysostosis Type 2 and Hypophosphatasia

Ulrich Montcho[1,2], Anne Marie Sbrocchi1[3,2]

[1]Shriners Hospital, Montreal, Canada. [2]McGill University Health Center, Montreal, Canada. [3]Shriners Hospital for Children, Montreal, Canada

### Abstract

### Background:

Acrodysostosis and hypophosphatasia (HPP) are rare skeletal disorders with distinct molecular bases but overlapping radiological and biochemical features. We report a 13-year-10-month-old European girl with a dual molecular diagnosis de novo ***PDE4D*** mutation (acrodysostosis type 2) and heterozygous pathogenic *ALPL* frameshift variant (HPP) highlighting the clinical, radiologic, and diagnostic complexities of combined skeletal dysplasias.

### Presenting Problem:

The patient was born at full term following an uncomplicated vaginal delivery, with normal birth weight and length, to non-consanguineous parents. Early growth and motor development were normal; she walked at 12 months. However, language development was delayed, with first words at 4 years, accompanied by learning difficulties and reduced social interaction. She has mild cognitive impairment and attends a specialized educational program. Menarche occurred at 13 years with irregular menses. She was seen at 13 years 10 months in our clinic for dysmorphic features and brachydactyly.

### Clinical Management:

On examination, she exhibited characteristic acrodysostosis features including round face, midface hypoplasia, and brachydactyly. Height was 149.5 cm (Z-score −1.6), weight 74.2 kg, and BMI 33.2 (98.6th percentile). Laboratory evaluation showed low serum alkaline phosphatase (40 U/L), normal PTH (3.6 pmol/L), calcium (2.53 mmol/L), and slightly low phosphate (0.98 mmol/L), with normal 25-hydroxyvitamin D (65 nmol/L). Spine imaging revealed mild thoracolumbar scoliosis and anterior concavity of L3–L5 vertebral bodies without cortical disruption or height loss. Bone age demonstrated severe brachymetatarsia, brachymetacarpia, brachydactyly, cone-shaped epiphyses, and advanced bone maturation. Foot x-ray also showed severe brachimetatarsia. DXA of the lumbar spine showed a normal Z-score (+0.8). Genetic testing identified **PDE4D c.679G>T (p.Ala227Ser; likely pathogenic, de novo)** and **ALPL c.1171del (p.Arg391ValfsTer12; pathogenic, heterozygous)**.

**Discussion:** This coexistence of *PDE4D*-related acrodysostosis and ALPL-related hypophosphatasia is exceptionally rare. Notably, vertebral compression fractures have not been previously reported in *PDE4D*-related acrodysostosis, suggesting that the vertebral concavities observed here likely reflect developmental dysplasia rather than fragility. Given her mild, asymptomatic HPP, enzyme replacement therapy with asfotase alfa was not initiated. This case underscores the importance of integrated phenotypic, radiologic, and genetic assessment in children with complex skeletal findings.

### Disclosure

None declared

### P182

Abstract withdrawn

### P183

### Patient Reported Pain in Children with Osteogenesis Imperfecta

Kieran Murphy, Claire Hill, Chidambaram Sethuraman

Sheffield Childrens Hospital foundation trust, Sheffield, UK

### Abstract

**OBJECTIVES**

To understand the prevalence of pain in children with Osteogenesis Imperfecta (OI) under the care of the metabolic bone disease team at Sheffield Children's Hospital.

To gain greater understanding of patient reported pain in paediatric OI population

To understand if children and families are interested in pain education

**METHODS**

Retrospective analysis of the pain domain (Q15-19) on 362 completed OI Quality of Life (OIQOL) questionnaires completed by patients under the care of the metabolic bone disease team between September 2016 and October 2024.

A scoping pain questionnaire was handed out to patients with OI and their families during their clinic appointments or admissions for bisphosphonates between January and March 2025.

**RESULTS**

Analysis of the OIQOL identified 6.9% of patients reporting a high frequency of pain with a further 17.1% reporting moderate frequency of pain.

The self-reported pain questionnaire was completed by 23 patients age between 8-17

26% reported daily pain, 9% weekly pain. 43% monthly pain with the remaining 22% never reporting pain. There was no correlation between OI type and reported incidence of pain. Back 32% and legs 28% were the most common areas for pain.

8 participants (35%) wanted pain education with 2 patients (9%) being unsure and 13 (56%) were not interested in pain education. There was a strong correlation between participants who reporting daily or weekly pain and wanting pain education of 62.5%.

**CONCLUSIONS:**

The service evaluation highlighted the reported prevalence of pain on children under the metabolic bone disease team at Sheffield Children's Hospital with 24% of patients who completed the OI QOL reporting pain having a significant impact on quality of life. 35% of patients who completed the scoping pain questionnaire reported daily or weekly pain with 35% of completed questionnaires wanting pain education.

As a service we aim to offer a larger program of support on pain management and pain education to all patients with a diagnosis of OI for them to opt in to.

### Disclosure

Dr Claire Hill developed OI quality of life questionnaire

### P184

### The Co-Design and Co-Development of an Online Self-Management and Transitional Care Program for Youth with Osteogenesis Imperfecta

Laura Massochin[1], Jennifer Stinson[2,3], Frank Rauch[1,4], Sofia Addab[1,4], Khadidja Chougui[1,5], Reggie Hamdy[1,4], Telma Palomo de Oliveira[6], Noemi Dahan-Oliel[1,4], Louis-Nicolas Veilleux[1,4], Nadia Narducci[1], Lori VandenBroek[1], Jessica Collins[1], Corinne Mercier[1], Mitchell Bernstein[1,4], Raissa Passos dos Santos[1,5], Kelly Thorstad[1], Argerie Tsimicalis[4]

[1]Shriners Hospitals for Children® – Canada, Montreal, Canada. [2]The Hospital for Sick Children, Toronto, Canada. [3]University of Toronto, Toronto, Canada. [4]McGill University, Montreal, Canada. [5]University of Montreal, Montreal, Canada. [6]Fleury Medicine and Healthcare, Sao Paulo, Brazil

### Abstract

### OBJECTIVES

Transition from pediatric to adult care is a critical period for youth with osteogenesis imperfecta (OI). Unprepared transitions can lead to poor treatment adherence, increased complications, and reduced quality of life. eHealth interventions show promise in optimizing transition. The study objectives were to co-design and co-develop "Teens Taking Charge: Managing OI Online (Teens OI)" with youth and young adults with OI, parents, and healthcare professionals.

### METHODS

A user-centered design guided by Garrett's Theory of User Experience and UX principles was conducted at a university-affiliated, not-for-profit, pediatric, orthopedic hospital in Canada. An advisory council oversaw the study. Participants participated via interviews or focus groups or completed a survey. Two iterative semi-structured interview cycles, followed by focus groups, and persona characters aided to further understand needs, inform the content and refine the prototypes. Descriptive statistics summarized demographics with persona characters depicting the targeted users of Teens OI. Content and thematic analyses were conducted to inform iterative design decisions moving from a low to a high-fidelity prototype.

### RESULTS

A 8-member advisory council including 3 nurses, 1 social worker, 2 adults with OI, 1 parent, and 1 youth oversaw the co-design and co-development of Teens OI followed by 10 youth with OI, 6 parents, 8 young adults with OI, and 35 survey respondents from 16 countries. Six main themes guided website development: [1] Purpose (community, positivity, awareness), [2] Functionalities (interactive features), [3] Resources (clinician contacts, self-management, emergencies), [4] Learning (multi-format content and peer testimonials), [5] Visual Design (inclusive and engaging), and [6] Content (condition-specific information, self-management, transition, empowerment). Three engaging personas captured heterogeneity in age, education, family context, and goals, were created to facilitate the co-design and development.

### CONCLUSIONS

A user-centered design approach was used to co-develop Teens OI with the end-users. Future work will focus on usability testing, content development, and rigorous evaluation to ensure youths with OI are knowledgeable, supported, and prepared for transition to adult healthcare.

### Disclosure

None declared

### P185

### ARSB Gene Variant as a Cause of Mucopolysaccharidosis Type VI Maroteaux-Lamy

Liliana Mejia de Beldjenna[1,2,3], Maria Valentina Mejia Santamaria[4], Jose David Mejia Z[5]

[1]Foundation children´s clinic Club Noel, cali, Colombia. [2]UNILIBRE,GRINPED, CALI, Colombia. [3]Colombian College of Pediatric Endocrinology Association, cali, Colombia. [4]University Buenos Aires, Buenos Aires, Argentina. [5]University ICESI, cali, Colombia

### Abstract

BACKGROUND

The ARSB gene (arylsulfatase B), located on chromosome 5q14.1, encodes the lysosomal enzyme arylsulfatase B, responsible for breaking down dermatan sulfate and chondroitin sulfate. Variants in this gene reduce enzyme activity, leading to the accumulation of glycosaminoglycans (GAGs) and causing mucopolysaccharidosis type VI (MPS VI), also known as Maroteaux-Lamy syndrome.

It manifests in infancy with macrocephaly, hydrocephalus, corneal opacity, hearing impairment, heart valve abnormalities, and hepatosplenomegaly, both due to the accumulation of glycosaminoglycans, and musculoskeletal system disorders, with multiple dysostosis, short stature, and motor dysfunction. It has a low prevalence, although high rates of inbreeding and parental consanguinity can lead to an increase of up to 20 per 100,000 live births in Brazil.

Diagnosis is based on clinical presentation and GAG assays, followed by evaluation of ASB enzyme activity in leukocytes or fibroblasts. Molecular analysis of ARSB gene damage confirms the diagnosis. Management consists of enzyme replacement therapy with recombinant human N-acetylgalactosamine 4-sulfatase.

PRESENTING PROBLEM:

Female, 5 years 6 months old, height -4 SD, dorsolumbar kyphoscoliosis since age 2, macrocephaly, exophthalmos, bell chest, genu valgum, claw hand, multiple joint contractures, echocardiogram with aortic insufficiency and mitral prolapse

CLINICAL MANGEMENT:

Due to suspected MPSVI, the following were found: altered GAGs in urine and decreased ASB enzyme activity in fibroblasts, and a homozygous variant in the ARSB gene c.1208C>A. The patient received enzyme replacement therapy with human N-acetylgalactosamine 4-sulfatase and is in stable condition.

DISCUSSION:

MPSVI is a rare condition found in populations with high levels of inbreeding, with a significant impact on the lives, bone metabolism, and systemic health of patients. Early suspicion and confirmation will allow for appropriate management, reduce comorbidities, improve quality of life, and provide genetic counseling to families.

### Disclosure none declared

### P186

### Caregivers and Child Preferences for Patient-Reported Outcome Measures (PROMs) in Skeletal Dysplasias

Michelle Savchenko[1], Jacob Louis Nicodemo[2], Saunya Dover[1], Adam Khalif[1], Matthew Boston[1], Joanna Lazier[3], Sasha Carsen[4,1], Kevin Smit[1,4], Mary-Ann Matzinger[5], Nazih Shenouda[5], Khaldoun Koujok[5], Leanne Marie Ward[1,6], Marie-Eve Robinson[1,6,7]

[1]Children's Hospital of Eastern Ontario Research Institute, Ottawa, Canada. [2]Faculty of Medicine and Health Sciences, McGill University, Montreal, Canada. [3]Department of Genetics, Children's Hospital of Eastern Ontario, Ottawa, Canada. [4]Division of Pediatric Orthopedic Surgery, Department of Surgery, Children's Hospital of Eastern Ontario, Ottawa, Canada. [5]Department of Radiology and Diagnostic Imaging, Children's Hospital of Eastern Ontario, Ottawa, Canada. [6]Division of Endocrinology, Department of Pediatrics, Children's Hospital of Eastern Ontario, Ottawa, Canada. [7]Shriners Hospital for Children - Canada, McGill University, Montreal, Canada

### Abstract

### Objectives:

Patient-reported outcome measures (PROMs) capture the patient's perspective on symptoms and functioning. While patient input is recommended during PROMs selection, studies on direct preference assessment among patients are lacking. Here, we evaluate which PROMs are most favoured by pediatric patients with skeletal dysplasias and their caregivers.

### Methods:

Data were collected through the SkeleTal Research on Novel Genes (STRONG) program, a multidisciplinary research program for children with skeletal dysplasias at the Children's Hospital of Eastern Ontario (CHEO). PROMs were completed by patients aged ≥8 years and by caregiver proxies for all participants. They were selected based on validation status, prior use or recommendation in pediatric chronic conditions (1-5), and consultation with the CHEO Patient and Family Advisory Council.

Preference questionnaires used a 5-point Likert scale to assess relevance, ease of understanding, and perceived usefulness for other patients with the same condition. For each survey and respondent group, results were summarized as the proportion of positive responses (agree/strongly agree) relative to total responses. The study was approved by the CHEO Research Ethics Board, and informed consent was obtained for all participants.

### Results:

Between April 2021 and May 2024, 38 pediatric patients (13.6 years, SD 3.4, range [7.9, 18.9]) and 65 caregiver proxies (mean age for their children 8.8 years, SD 5.2, range [0.3, 18.4]) completed PROMs, followed by standardized preference questionnaires. Overall, all instruments were well accepted by both groups. The average proportion of agree or strongly agree responses indicating surveys were easy to complete was 96% among caregivers (up to n = 56 responses per questionnaire) and 89.3% among patients (n = 35). Perceived relevance averaged 78.3% for caregivers and 68.6% for patients, while recommendation for use with other children averaged 87.3% and 87.1%, respectively. Among caregivers, the most highly rated instruments for perceived relevance were PROMIS Depressive Symptoms (90.0%), PROMIS Mobility (88.0%), and Child Sleep Habits (84.0%) questionnaires. Among pediatric patients, PROMIS Anxiety (76.5%), PedsQL Core (75.8%), and the Pediatric Pain Questionnaire (73.1%) were rated as most relevant.

### Conclusion:

This work can inform clinicians' decisions when selecting PROMs for routine use among children with skeletal dysplasias.

https://drive.google.com/file/d/1C8dpAXJTgEvfaV3PZbb-c2VkOLlJphLO/view?usp=drivesdk

Figure 1: Distribution of survey preference ratings among caregivers (a) and pediatric patients (b) based on perceived relevance of PROMs.

### Disclosure

Marie-Eve Robinson reports the following disclosures: Ascendis Biopharma, Ipsen Biopharmaceuticals, QED therapeutics: Study grant to institution; Ultragenyx, Biomarin, Ipsen Biopharmaceuticals: Consultancy fees to institution. Saunya Dover reports the following disclosures: full time employment at Canada's Drug Agency outside the submitted work. Leanne Ward declares consultancy to Ultragenyx, Kyowa Kirin, Amgen, BioMarin, Angitia, and Ipsen, with funds to Dr. Ward’s institution. Dr. Ward declares participation in clinical trials with Ascendis, QED, Amgen, Alexion, and Ultragenyx, with funds to Dr. Ward’s institution.

### P187

### Service Evaluation of a Paediatric MDT Achondroplasia Clinic: Parent and Patient Satisfaction Outcomes

Helen Collins, Riya Bisht, Rebecca Doodson, Nicola Panchbhaya, Lauren Rayner, Antonia McBride, Varun Parnami, Emma Gosnell, Roberto Ramirez, Shafqat Bukhari, Amish Chinoy

Royal Manchester Children's Hospital, Manchester, UK

### Abstract

**Objectives**:
Achondroplasia requires coordinated input from multiple paediatric specialties, often resulting in numerous hospital visits for families. To streamline care and improve patient experience, an annual paediatric multidisciplinary (MDT) Achondroplasia Clinic was established, bringing key professionals together into a single appointment (endocrinology, neurosurgery, ENT, respiratory, orthopaedics, occupational therapy, physiotherapy). Our objective was to evaluate parent and patient satisfaction with the new MDT clinic model and explore perceived benefits, challenges, and service-improvement priorities.

**Methods**:
Following attendance at the MDT clinic, families were invited to complete a feedback questionnaire. This captured satisfaction scores (1–10), perceived helpfulness of the MDT model, estimated number of future appointments saved, and qualitative feedback regarding strengths and areas for improvement. Quantitative responses were summarised descriptively, and qualitative data were thematically analysed.

**Results**:
Twenty-seven responses were collected from 32 families (84% response rate). Overall satisfaction was high (mean 9.63/10), with 20 respondents scoring 10/10. Most (23/27) described the MDT clinic as “very helpful”. Families also estimated a median of 4 appointments saved over the following 12 months. Analysis of the themes highlighted four major strengths:

1. A ‘one-stop’, integrated MDT format;
2. improved communication and clarity with opportunities to ask all questions in one setting;
3. reduced travel, time burden, and stress;
4. accelerated clinical decision-making (e.g. ‘timely ENT’ and ‘sleep-studies’ arrangements).

Areas for improvement included inconsistent speciality attendance, potential overwhelm for neurodiverse children, and environmental factors (room temperature, signage).

**Conclusion**:
The Achondroplasia MDT clinic demonstrates high acceptability and significant perceived benefits for families, reducing appointment burden and enhancing coordinated care. We will review the clinic room layout and create a pre-clinic social story for new patients. Future development should also address a minimum MDT composition, including the absence of psychology within the clinic’s MDT structure, as the new UK national consensus guideline identifies psychological support as a core component of minimum MDT provision.

### Disclosure

None declared

### P188

Abstract withdrawn

### P189

### X-Linked Hypophosphatemia and Craniosynostosis; Experience from a Regional UK Centre

Ian Tucker, Tabitha Hoole, Manju Chandwani, Toby Candler

Department of Paediatric Endocrinology, Bristol Royal Hospital for Children; University Hospitals Bristol NHS Foundation Trust, Bristol, UK

**Abstract**

BACKGROUND

Craniosynostosis is a reported complication of X-Linked Hypophosphatemia (XLH). A recent meta-analysis by Fisch *et al* (2025) reported a prevalence of craniosynostosis among children with XLH of 22% with males disproportionately affected. This reports a cohort in a tertiary paediatric centre in Southwest England (SWE).

PRESENTING PROBLEM

Twenty-four children <19 years with XLH (male=11, female=13) are in our service. Six patients were identified (male=6) with craniosynostosis from SWE (see table 1). This equates to 25% (n=6/24) of the total cohort and 54% (n=6/11) of the male patients. In all cases scaphocephaly was suspected during routine examination of head-shape and circumference. Craniosynostosis was confirmed by CT in all cases. Craniosynostosis was already present in 50% of cases prior to initiation of burosumab. Mean age of clinical suspicion was 5.0 years [range=1-10.5yrs] with mean age of craniosynostosis diagnosis was 5.5 years [1.2- 10.5yrs]. The majority had sagittal sutures affected (n=5 had sagittal sutures affected, n=1 had bicoronal and sagittal sutures affected). Case 6 (a late diagnosis of XLH), also had Chiari malformation.

| | **Case 1 Male** | **Case 2 Male** | **Case 3 Male** | **Case 4 Male** | **Case 5 Male** | **Case 6 Male** |
|---|---|---|---|---|---|---|
| **Age at Clinical suspicion craniosynost.** | 1 year | 1y6m | 2y4m | 4y10m | 10years | 10y5m |
| **Age at confirmation craniosynost.** | 1y2m | 2y4m | 2y6m | 6y10m | 10y3m | 10y5m |
| **Age at XLH Dx** | Neonatal (Family Hx) | Neonatal (Family Hx) | Neonatal (Family Hx) | 3 Years | Neonatal (Family Hx) | 10y 5m |

| | | | | | | |
|---|---|---|---|---|---|---|
| **Age of burosumab initiation** | 1y4m | 1 year | 1y 2m | 5years | 3 years | 10 y 5m |
| **Other significant features** | | Intercranial Hypertension | Developmental delay, Speech delay | | Parental concern with head shape | Chiari malformation |
| **Outcome** | Ongoing surveillance | SURGERY @2y11m | Ongoing surveillance | Ongoing surveillance | Ongoing surveillance | Ongoing surveillance |
| Table 1. Summary of Cases with craniosynostosis and XLH in SWE | | | | | | |

CLINICAL MANAGEMENT:

All identified patients were referral to a craniofacial specialist centre. One child had developmental and speech delay. To date, only one child has required surgical intervention with 5 having ongoing surveillance and monitoring.

DISCUSSION:

The SWE prevalence is 25% is consistent with recent reports in the literature. Males are disproportionately represented. Monitoring of head shape and circumference, coupled with a low threshold for radiological assessment to identify craniosynostosis is key to early diagnosis. Prompt referral to specialist centres for ongoing surveillance/management is essential. More data is required to understand if Burosumab alters clinical course with regards craniosynostosis.

**Disclosure**

None declared

### P190

### Healthcare Service Provision Evaluation for Children and Young People with Fibrodysplasia Ossificans Progressiva in the UK

Kayleigh Hoey[1], Amy Culham[1], Helen Bedford-Gay[2], Kunal Patel[3], Hui-Leng Tan[4], Richard Keen[1], Alexander Chesover[1]

[1]The Royal National Orthopaedic Hospital, London, UK. [2]FOP Friends, Manchester, UK. [3]Great Ormond Street Hospital for Children, London, UK. [4]The Royal Brompton Hospital, London, UK

### Abstract

### OBJECTIVE

Fibrodysplasia ossificans progressiva (FOP), a rare genetic disorder, causes irreversible and progressive immobility from bone accumulation in soft tissues. International guidance recommends occupational therapy (OT), audiology, respiratory and dental assessments. Hydrotherapy, physiotherapy and psychology can also be beneficial. We aimed to evaluate access to healthcare services for children and young people (CYP) with FOP.

### METHODS

We invited the 20 known CYP (<18 years) with FOP and their parents in the UK to complete a survey - developed using Microsoft Forms and distributed by e-mail (for patients known to our national paediatric FOP clinic) and the UK charity, FOP Friends. Respondents consented to participate. They self-reported healthcare service utilisation and needs. This is a registered service evaluation project.

### RESULTS

Responses were received from 18/20 (90%) known patients; 10/18 (55%) female, and median age 10.5 years (range 2-18). Median age at diagnosis was 3.0 years (range 0.3-12). Median time to diagnosis from first symptom/signs of FOP was 1.1 years (range 0-12). Respondents reported mobility aid use in 14/18 (77%), respiratory assessment in 11/18 (61%), hearing loss in 9/18 (50%), mental health support in 9/18 (50%), and slightly restricted jaw opening in 5/18 (27%). All had previous dental review.

Of 15/18 (83%) respondents who completed a hearing screen, 10/15 (67%) had follow-up. For therapy services: 15/18 (83%) had OT, and two who hadn't thought this would be helpful; 11/18 (61%) had physiotherapy, and one who hadn't thought this would be helpful; 7/18 (39%) respondents had hydrotherapy, and 8/10 (80%) who hadn't thought this would be helpful. Therapy helped respondents with "gaining independence", "finding solutions," "accessibility at home and school," and to "make life a bit more comfortable."

### CONCLUSION

Median age at diagnosis is younger than previously reported, 3.0 vs 5.6 years, possibly reflecting improved access to genetic diagnosis, information, and support. This study demonstrates the perceived impact of FOP on mobility, hearing, and mental health. Access to dental care appears good.  Physical therapy improves access and inclusion. Improved audiology and OT provision is still required to meet international recommendations. The potential benefits for physiotherapy, hydrotherapy, and mental health support needs further investigation.

### Disclosure

R.K. is a clinical investigator for Ipsen, Incyte and Regeneron. A.C. is a clinical investigator for Regeneron.

### P191

### Integrating Research into Multidisciplinary Care for Skeletal Dysplasia: The STRONG Model

Jacob L Nicodemo[1], Adam Khalif[2], Saunya Dover[2], Matthew Boston[2], Joanna Lazier[3], Sasha Carsen[2,4], Kevin Smit[2,4], Mary-Ann Matzinger[5], Khaldoun Koujok[5], Nazih Shenouda[5], Leanne M Ward[2,6], Marie-Eve Robinson[1,2,6,7]

[1]Faculty of Medicine, McGill University, Montreal, Canada. [2]Children's Hospital of Eastern Ontario Research Institute, Ottawa, Canada. [3]Department of Genetics, Children's Hospital of Eastern Ontario, Ottawa, Canada. [4]Division of Pediatric Orthopedic Surgery, Department of Surgery, Children's Hospital of Eastern Ontario, Ottawa, Canada. [5]Department of Radiology and Diagnostic Imaging, Children's Hospital of Eastern Ontario, Ottawa, Canada. [6]Division of Endocrinology, Department of Pediatrics, Children's Hospital of Eastern Ontario, Ottawa, Canada. [7]Shriners Hospital for Children - Canada, McGill University, Montreal, Canada

### Abstract

**Objectives**:

To describe the development of the SkeleTal Research on Novel Genes (STRONG) Program and assess patient and provider satisfaction.

**Methods**:

The STRONG Program was developed as a multidisciplinary clinic and research program for children with skeletal dysplasia seen at the Children's Hospital of Eastern Ontario (CHEO). Patient perspectives were integrated through partnership with the CHEO Patient and Family Advisory Committee. Iterative Plan–Do–Study–Act cycles were used to guide program development.

Following initial STRONG visits, families completed a STRONG-specific satisfaction questionnaire, adapted from the Measures of Processes of Care questionnaire (1). Health care professionals (HCPs) completed questionnaires assessing their STRONG experience. Questionnaires employed a 5-point Likert scale. The study was approved by CHEO's research ethics board and participants provided informed consent.

**Results**:

Patients with skeletal dysplasia (age 0-18 years) were eligible to enroll. STRONG clinics were held monthly with four patients per half-day (Figure 1). During clinics, patients were evaluated by a multidisciplinary team. Following case discussions among HCPs, families were invited to discuss a unified care plan and participation in diagnostic (2) or interventional research studies. Clinical data were captured directly via structured flowsheets within the electronic medical record (3), eliminating duplicate research data entry and enabling discrete data extraction for downstream analyses.

From April 2021 to May 2024, 35 patient caregiver proxies completed satisfaction questionnaires (n=35 children, 40% female, mean age 8.1 years (SD=5.0), range 5 months to 17 years). Participants' diagnoses included achondroplasia (14%), juvenile osteoporosis (6%), osteogenesis imperfecta (11%), and other skeletal dysplasia (69%). Caregivers reported that providers listened to their views (mean score=4.7) and gave answers they could understand (4.8). They felt engaged in decision-making (4.3), that care was efficient (4.6), and satisfied overall with their care (4.6).

HCPs (n=15) felt that STRONG added value for patients (4.7) and providers (4.3), educational rounds were helpful for patients (4.6) and providers (4.0), the time they cared for patients was more efficient (3.5), and back-and-forth communication with colleagues decreased (3.8).

**Conclusions**:

STRONG can serve as a scalable model for integrated care and research for children with skeletal dysplasia or other rare diseases.

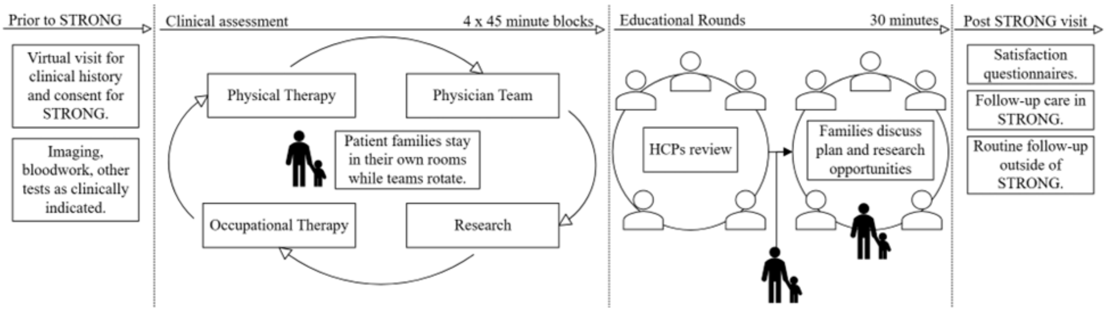

**Figure 1**: Clinic flow for STRONG visits. Pediatric endocrinologists, geneticists, orthopedic surgeons, and other medical specialists, as indicated by a patient's condition, assessed patients together. A pediatric radiologist is present during the Educational rounds to review imaging.

**References:**

1. King S, King G, Rosenbaum P. Evaluating Health Service Delivery to Children With Chronic Conditions and Their Families: Development of a Refined Measure of Processes of Care (MPOC−20). Children's Health Care. 2004;33(1):35-57.
2. Boycott KM, Hartley T, Kernohan KD, Dyment DA, Howley H, Innes AM, et al. Care4Rare Canada: Outcomes from a decade of network science for rare disease gene discovery. Am J Hum Genet. 2022;109(11):1947-59.
3. Epic. Epic Systems Corporation; Accessed 2025.

### Disclosure

Marie-Eve Robinson reports the following disclosures: Ascendis Biopharma, Ipsen Biopharmaceuticals, QED therapeutics: Study grant to institution; Ultragenyx, Biomarin, Ipsen Biopharmaceuticals: Consultancy fees to institution. Saunya Dover reports the following disclosures: full time employment at Canada's Drug Agency outside the submitted work. Leanne Ward declares consultancy to Ultragenyx, Kyowa Kirin, Amgen, BioMarin, Angitia, and Ipsen, with funds to Dr. Ward's institution. Dr. Ward declares participation in clinical trials with Ascendis, QED, Amgen, Alexion, and Ultragenyx, with funds to Dr. Ward's institution.

### P192

### Assessing the Functional Relationships Between Grip Strength, Handwriting Performance and Typing in X-Linked Hypophosphataemia

Kate Ali, Emma Bewick, Alexander Chesover, Moira Cheung

Great Ormond Street Hospital, London, UK

### Abstract

**Background:**

The relationship between X-linked hypophosphataemic rickets (XLH) and muscle function is well recognised but characterisation is limited to lower limbs and motor outcomes. A literature search did not identify XLH studies exploring the relationship between hand function and functional outcomes, such as writing and typing, in patients with XLH.

**Objectives/Aims**

A random sample of 15 children with XLH, aged 9 to 16 years, were assessed for grip strength and functional hand tasks using a dynamometer to calculate grip strength, the Detailed Assessment of Speed of Handwriting (DASH) 2nd edition, and a typing speed test.

**Results**

Grip strength was lower in 40% (6/15) and mean values were 1 standard deviation below normative values (corrected for age and sex). Four of these 6 were prepubertal girls, with a normative mean of 18.6 kg expected, this prepubertal population presented with a mean of 13.3 kg and standard deviation mean of 4.1kg.

The DASH results were largely within the 'no difficulty' range - only two participants performed within the 'slow and moderately slow' range. The '*copy fast*' DASH activity results showed a handwriting speed mean of 25 words per minute (wpm). Comparative typing speed activities showed a typing speed mean of 33 wpm. Typing was faster than handwriting for 86.6% (13/15).

Handwriting was illegible for 2 of 15 participants and on the '*free writing*' DASH task (a 10 minute task), handwriting speed decreased to a mean of 15 wpm, and 40% (6/15) participants reported discomfort in their dominant hand.

**Discussion**

Although grip strength was below the mean for the 40% of the sample, performance on handwriting did not correlate with lower muscle function and did not indicate poor handwriting speed or legibility in XLH.

Reduced grip strength does not indicate that a motor strengthening programme is required. Specific difficult to perform activities should be practiced in natural contexts. This is consistent with systematic review findings; interventions that promote activity or specific skills have stronger outcomes. For this group technology, provides an advantage in speed and clarity of documented work. Of note, 93% (14/15) participants were treated with burosumab and it is unclear how they would have performed without treatment.

### Disclosure

None declared

### P193

### Intravenous Pamidronate Therapy in an Infant with Severe Autosomal Recessive Cutis Laxa Type 1B Caused by a Novel *EFEMP2* Variant: A First Report

Stephanie Elizarde[1,2], Linda Randolph[3,4], Anna Ryabets-lienhard[1,3,4,2]

[1]Pediatric Metabolic Bone and Mineral Disorders Program, Division of Pediatric Endocrinology, Diabetes, and Metabolism, Children's Hospital Los Angeles, Los Angeles, USA. [2]The Saban Research Institute, Children's Hospital Los Angeles, Los Angeles, USA. [3]Keck School of Medicine, University of Southern California, Los Angeles, USA. [4]Division of Medical Genetics, Children's Hospital Los Angeles, Los Angeles, USA

### Abstract

BACKGROUND: Autosomal recessive cutis laxa type 1B (ARCL1B) is an ultra-rare connective tissue disorder caused by pathogenic variants in EGF-containing fibulin-like extracellular matrix protein 2 (EFEMP2) resulting in skin, vascular, and skeletal abnormalities. Though fractures have been reported in ARCL1B, there is lack of information on bone fragility treatment in these patients. We describe a unique case of a 20-month-old toddler with severe ARCL1B due to a novel EFEMP2 variant presenting with congenital fractures treated with intravenous pamidronate since the neonatal period.

PRESENTING PROBLEM: A 2-day-old female was born at 37 weeks gestation (weight [-1.72 SD], length [1.08], and head circumference [-2.01 SD]) with multiple congenital fractures requiring pain management. Skeletal survey revealed a left parietal fracture and multiple healing long-bone fractures. Physical exam showed loose skin, contractures, and arachnodactyly. Vascular anomalies included a tortuous right brachial artery, a displaced left aortic arch, and hypoplastic right pulmonary artery. By 22 days of life, she developed hypercalcemia (iCal 6.2 mg/dl, iPTH 12.0mg/dL), likely from immobility and multiple fractures. Clinical exome sequencing revealed a novel homozygous EFEMP2 missense pathogenic variant, c.226G>A (p.Gly76Arg), confirming ARCL1B.

CLINICAL MANAGEMENT: At 23 days of life, she received intravenous pamidronate (0.25 mg/kg/dose) with resolution of hypercalcemia. At 2 months, she was found to have new bilateral tibial and fibular fractures and was started on a 3-day pamidronate protocol (0.25 mg/kg/dose) every 2–3 months. Radiographs at 4 months showed healing fractures and a new right humeral fracture. She transitioned to zoledronic acid every 6 months at 19 months of age. She has tolerated bisphosphonates well and has remained fracture-free for 16 months since her last fracture.

DISCUSSION: We report a unique case of ARCL1B caused by a novel EFEMP2 variant associated with a severe skeletal phenotype treated with intravenous bisphosphonates. Existing literature on ARCL1B largely focuses on cardiovascular and cutaneous manifestations, with limited guidance on skeletal management. In this case, bisphosphonates were well tolerated and associated with resolution of hypercalcemia, pain, and a reduction in fractures. Further studies are needed to evaluate long-term efficacy and safety of bisphosphonates in ARCL1B and to determine whether this novel EFEMP2 variant contributes to skeletal disease severity.

### Disclosure

Anna Ryabets-Lienhard, DO is a Consultant with BioMarin and Alexion; clinical trials PI with Ultragenyx and Takaeda Pharmaceuticals Linda Randolph, MD: Clinical trials and Consultant with BioMarin Stephanie Elizarde, BS: None

### P194

### From Congenital Hallux Valgus to Fibrodysplasia Ossificans Progressiva: An Evolving Skeletal Phenotype in Childhood

Ali Afrasiabi[1], Shuaa Abdulwahab M Basalom[2], Noa De Louya[4], Pascale Sabeh[3,1], Philippe Campeau[3]

[1]Université de Montréal, Montreal, Canada. [2]CHU de Québec, Montreal, Canada. [3]CHUSJ, Montreal, Canada. [4]McGill University Health Centre, Montreal, Canada

### Abstract

BACKGROUND:

Fibrodysplasia ossificans progressiva (FOP) (OMIM135100) is an ultra-rare, genetic disorder caused by gain-of-function variants in *ACVR1*(102576), leading to progressive heterotopic ossification. With an estimated prevalence of approximately 1 in 1.5–2 million individuals worldwide, early diagnosis is critical to guide management and limit preventable morbidity. Recognition remains challenging when early manifestations are subtle or interpreted as isolated congenital skeletal findings.

PRESENTING PROBLEM:

We report the case of a boy born from a dichorionic–diamniotic twin pregnancy who presented at birth with bilateral congenital hallux valgus. His sister was unaffected, and there was no family history of skeletal or genetic disease. Initial investigations included chromosomal studies which non-diagnostic. Over childhood, the phenotype evolved gradually, with additional skeletal abnormalities including severe congenital cervical vertebral fusion consistent with a Klippel–Feil–like phenotype, progressive musculoskeletal stiffness, and functional limitation. At 9 years of age, the patient developed progressive limitation of mouth opening (trismus), prompting further assessment for temporomandibular pathology or ectopic ossification and referral to clinical genetics was re-initiated

CLINICAL MANAGEMENT:

Craniofacial CT and MRI demonstrated complex osseous morphology involving the mandibular ramus and adjacent skull base, with pseudo-articulations and bony bridging, initially considered possibly congenital. As multisystem skeletal features progressed alongside congenital great-toe abnormalities, a broadened genetic evaluation using a multi-gene skeletal disorder panel was pursued. This identified a pathogenic heterozygous *ACVR1* variant, NM_001105.4:c.617G>A (p.Arg206His), confirming classic FOP. Parental segregation studies identified it's a de novo variant. Following diagnosis, invasive procedures were avoided, and coordinated multidisciplinary care was established, emphasizing trauma avoidance, anticipatory guidance, and supportive management.

DISCUSSION:

This case illustrates the evolving nature of FOP and how congenital hallux valgus, when isolated, may not immediately prompt recognition of a unifying diagnosis. The progressive nature of skeletal features across regions underscores the importance of longitudinal phenotypic integration, multidisciplinary communication, and early molecular confirmation to inform management decisions and minimize preventable morbidity over time. Increased awareness of this presentation may aid clinicians worldwide in identifying comparable early skeletal patterns and incorporating FOP into diagnostic considerations.

### Disclosure

None declared

### P195

### Osteogenesis Imperfecta in a Boy with Multiple Severe Comorbidities

Stepan Kutilek[1,2], Richard Pikner[3,2]

[1]Department of Pediatrics, Klatovy Hospital, Klatovy, Czech Republic. [2]Bone Disease Center, Klatovy Hospital, Klatovy, Czech Republic. [3]Dept. of Clinical Biochemistry , Klatovy Hospital, Klatovy, Czech Republic

### Abstract

**Background:**

Osteogenesis imperfecta (OI) represents a heavy burden on the patient, especially when other significant comorbidities are present. We present a 4-year old boy with a complicated history of severe multimorbidity, recurrent fractures and OI.

**Case Report, Presenting Problem & Clinical Management:**

The boy was born of in vitro fertilisation (IVF) pregnancy, by spontaneous delivery on 38th gestational week, birthweight was 2030 g, length 49 cm. Apgar score was 10-10-10 points. Prenatally, urinary bladder exstrophy and bilateral clubfeet were diagnosed by ultrasound. Surgical correction of the bladder exstrophy was performed at the age of 3 days and 10 months (neobladder reconstruction), respectively. The clubfeet were managed by Ponseti method. Since the age of six months, recurrent pyelonephritis occurred and high-grade vesicoureteral reflux (VUR) was diagnosed. Surgical correction of VUR was performed at one and four years of age, respectively. The boy also manifests generalized joint hypermobility. He is short (93 cm, i.e. third percentile). In spite of being handicapped, his psychomotor development is normal. Between the age of three and four years he suffered four low-energy trauma fractures of the lower extremities. Biochemical parameters (S-creatinine, AST, ALT, Ca, P, Mg, ALP, PTH, blood urea nitrogen and creatinine), together with creatinine clearance (Schwartz formula) are all within normal reference ranges. His spinal bone mineral density (BMD measured by DXA) is low (0.331 g/cm2; -2.5 SD Z-score). Genetic examination revealed pathogenic heterozygous sequential variant in COL1A2:c.594+1G>T and heterozygous variant in COL1A1 : c.2095G>Tp. (Ala699Ser), which corresponds to OI type I and Ehlers-Danlos syndrome (EDS). Therefore, treatment with intravenous zoledronate (0.05 mg/kg twice a year) is underway. Currently he is doing well, followed-up by a pediatric urologist and nephrologist, orthopedic surgeon, paediatric osteologist and geneticist.

**Discussion:**

The patient has a very painful combination of significant comorbidities, further complicated by recurrent fractures and collagenopathies (OI and EDS). Application of i.v. bisphosphonates should improve his bone health. Due to the severely compromised urinary system, such treatment might be considered as a risky one. However, as his renal functions are currently normal, the bisphosphonate (zoledronate) therapy is feasible.

### Disclosure none declared

### LB1

### Stage-Specific Effects of IGF1 on hiPSC-Derived Chondrocytes Revealed by RNA-seq

Masanobu Fujimoto, Shintaro Senoo, Yuko Isoda, Kengo Kurosawa, Yukiko Yamaguchi, Noriyuki Namba

Division of Pediatrics & Perinatology, Faculty of Medicine, Tottori University, Yonago, Japan

### Abstract

**OBJECTIVES:**

Differentiation of chondrocytes has been proposed to involve multiple molecular mechanisms, including the BMP, RUNX, and WNT/β-catenin pathways. IGF1 signaling is also vital for bone growth and is important for the hypertrophic zone of the growth plate (GP) in mouse models. However, the details of IGF1 signaling and its role in human GP remain unclear. This study investigates changes in RNA expression induced by IGF1 stimulation in human induced pluripotent stem cell (hiPSC)-derived chondrocytes and elucidates IGF1 actions in chondrocytes.

**METHODS:**

Normal hiPSCs (201B7-Ff) were differentiated into chondrocytes (Day 23–24 and Day 39) via the sclerotome lineage. Safranin O staining confirmed chondrogenic differentiation. After a 6-hour starvation period, the pellets were further incubated with or without IGF1 (100 ng/mL). RNA was extracted, and bulk RNA sequencing was performed. Genes associated with chondrocytes were retrieved from public databases, and their expression changes in response to IGF1 stimulation were evaluated.

**RESULTS:**

Histological analysis indicated that Day 23–24 pellets resembled proliferative chondrocytes, whereas Day 39 pellets displayed features of prehypertrophic chondrocytes. RNA-seq revealed elevated levels of proliferative markers (*SOX9*, *COL2A1*, *COL9A1*, *MATN3*) in Day 23–24 pellets, while prehypertrophic markers (*RUNX2*, *IHH*, *SP7*) were increased in Day 39 pellets. IGF1 stimulation at Day 23–24 reduced the expression of *COL2A1*, *ACAN*, and *COL9A1* and increased levels of *IHH*, *SP7*, and *COL10A1* ($p < 0.05$). Gene ontology analysis revealed altered expression of genes regulating ossification, osteoblast differentiation, cell fate specification, cartilage development, and planar cell polarity in pellets stimulated with IGF1 on Day 23-24. Moreover, IGF1 stimulation increased *CCND1* and *CDKN1C* in Day 23–24 pellets. In contrast, it altered the expression of *BMP2*, *BMP4*, and *SMADs* involved in BMP signaling in Day 39 pellets.

**CONCLUSIONS:**

Chondrocyte pellets correspond to proliferative chondrocytes at Day 23-24 and to prehypertrophic chondrocytes at Day 39. Our findings suggest that IGF1 facilitates the differentiation of proliferative chondrocytes toward hypertrophic chondrocytes, at least in part, by modulating cell-cycle and extracellular matrix genes. In contrast, it affects BMP signaling-related factors in prehypertrophic chondrocytes, indicating stage-specific responses.

### Disclosure

None declared

### LB2

### Metabolic Risk, Adiposity, and Bone Mineral Density: Method-Dependent Associations in Term- and Preterm-Born Young Adults

Li Feng Xie[1], Anik Cloutier[2], Nathalie Alos[2,3], Mélissa Fiscaletti[2,3], Anne-Monique Nuyt[2,3], Thuy Mai Luu[2,3]

[1]Université Laval, Quebec, Canada. [2]Azrieli Research Center of CHU Sainte-Justine, Montreal, Canada. [3]Department of Pediatrics, CHU Sainte-Justine, Université de Montréal, Montreal, Canada

### Abstract

### Background:

The relationship between bone mineral density (BMD) and metabolic health remains inconsistent; how preterm birth influences the interplay between these conditions in adulthood is incompletely understood. This study examined the associations between metabolic risk and BMD in young adults, comparing term- and preterm-born individuals.

### Methods:

Young adults born <30 weeks of gestational age in Quebec, Canada, and term controls were recruited for this cross-sectional study. BMD at the lumbar spine, femoral neck, and whole body was measured using dual-energy X-ray absorptiometry. Metabolic risk was assessed using composite scores (log-based and z-score), derived from waist circumference, blood pressure, HDL cholesterol, and fasting glucose, with and without waist circumference (WC). Pearson correlation analyses were conducted to evaluate associations between BMD and metabolic components and composite scores, stratified by prematurity status. Multivariable linear regression models were used to assess the independent associations of WC, non-adiposity metabolic risk, and preterm birth with BMD.

### Results:

A total of 193 young adults were included (101 term, 92 preterm-born; mean age 23.2 ± 2.4 years; 43% male). In term-born individuals, WC was positively correlated with BMD across all skeletal sites (lumbar spine $r = 0.369$, femoral neck $r = 0.381$, whole body $r = 0.595$; all $p < 0.001$). In preterm individuals, these associations were weaker but remained significant for femoral neck and whole-body BMD ($r = 0.302$ and 0.368; $p \leq 0.004$). Other metabolic components showed weak or inconsistent associations with BMD. In multivariable models, WC was independently and positively associated with BMD ($\beta = 0.246$–0.524, all $p \leq 0.001$) in models using composite scores excluding WC, while preterm birth was associated with lower BMD ($\beta = -0.142$ to $-0.290$, $p \leq 0.024$). In contrast, metabolic risk excluding adiposity was not associated with BMD (all $p > 0.05$). Associations between metabolic health and BMD varied by score definition, weakening after removal of WC and differing between log- and z-score approaches.

### Conclusion:

Associations between metabolic risk and BMD are influenced by adiposity and score definition; metabolic dysfunction without adiposity shows limited effects. Preterm birth remains associated with lower bone mass.

Table 1: Associations Between Metabolic Risk Score Definitions and Bone Mineral Density by Birth Status in Young Adults

| Metabolic Risk Score | Group | Lumbar Spine BMD r (p) | Femoral Neck BMD r (p) | Whole-Body BMD r (p) |
|---|---|---|---|---|
| Log (with WC)[1] | Term | 0.294 (0.003)* | 0.353 (<0.001)* | 0.554 (<0.001)* |
| | Preterm | 0.108 (0.308) | 0.278 (0.009)* | 0.368 (<0.001)* |
| Log (no WC)[2] | Term | 0.086 (0.391) | 0.182 (0.077) | 0.296 (0.003)* |
| | Preterm | 0.073 (0.491) | 0.160 (0.136) | 0.244 (0.020)* |
| Z-score (with WC)[3] | Term | 0.185 (0.064) | 0.179 (0.081) | 0.415 (<0.001)* |
| | Preterm | −0.039 (0.712) | 0.205 (0.054) | 0.216 (0.039)* |
| Z-score (no WC)[4] | Term | 0.077 (0.443) | 0.065 (0.532) | 0.274 (0.005)* |
| | Preterm | −0.102 (0.333) | 0.128 (0.234) | 0.108 (0.309) |

[1] Metabolic Risk Score = WC + (SBP + DBP)/2 − log(HDL) + log(TG) + log(Glu)

[2] Metabolic Risk Score = (SBP + DBP)/2 − log(HDL) + log(TG) + log(Glu)

[3] Metabolic Risk Score = z(WC) + z[(SBP + DBP)/2] − z(HDL) + z(TG) + z(Glu)

[4] Metabolic Risk Score = z[(SBP + DBP)/2] − z(HDL) + z(TG) + z(Glu)

Where WC, waist circumference; SBP, systolic blood pressure; DBP, diastolic blood pressure; HDL, high-density lipoprotein cholesterol; TG, triglycerides; Glu, fasting plasma glucose; z(·), standardized value (z-score calculated as (value − mean) / standard deviation)

*Statistically significant at a $p < 0.05$

### Disclosure

None declared

### LB3

### Foramen Magnum Stenosis Imaging in Infants with Achondroplasia

Emma Snow[1], Alessandra Cocca[1], Dominic Thompson[2], Moira Cheung[2], Rhoda Akilapa[1], Philippa Prentice[1], Robyn Gilbey Cross[1]

[1]Evelina London, London, UK. [2]Great Ormond Street Hospital, London, UK

### Abstract

**Objective:**
Current literature supports the use of magnetic resonance imaging (MRI) in infants with achondroplasia to screen for foramen magnum stenosis (FMS). However, the optimal timing for first screening and follow-up MRI remains uncertain, particularly when balancing resource utilisation, the potential requirement for anaesthesia in older infants, and parental anxiety. This study aimed to evaluate the natural history of FMS in infants undergoing early MRI (≤14 weeks of age) and subsequent follow-up imaging, in order to inform optimal timing of repeat scans.

**Methods:**
A retrospective cohort study was conducted between 2020-25. Inclusion criteria comprised genetically confirmed diagnosis of Achondroplasia and first MRI performed before 3 months of age Participants were stratified into two groups based on timing of follow-up MRI: <50 weeks (n = 13) and ≥50 weeks (n =11). Changes in AFMS grading between initial and follow-up imaging were analysed, with statistical comparisons performed using the Wilcoxon signed-rank test and Fisher's exact test.

**Results:**
71 infants with Achondroplasia were evaluated during the study period. Exclusions were 38 due to intial MRI performed after 3 months, 3 who proceeded to urgent surgical decompression and 6 who did not undergo second MRI. The remaining 24 patients were analysed.

In the <50-week follow-up group, the mean age at initial MRI was 6.5 weeks and at follow-up was 40.2 weeks. AFMS remained stable in 61.5% of infants, progressed in 30.8%, and improved in 7.7%. Median AFMS remained unchanged (AFMS1), with no statistically significant difference between scans (p 0.63).

In the ≥50-week follow-up group, the mean age at initial MRI was 11.3 weeks and at follow-up was 63.7 weeks. AFMS progressed in 36.4% of infants and remained stable in 63.6%. Median AFMS increased from AFMS1 to AFMS2, with a statistically significant difference observed between scans (p 0.03).

Comparison between groups demonstrated similar proportions of progression (<50 weeks: 30.8% vs ≥50 weeks: 36.4%), with no statistically significant difference (Fisher's exact test p 1.0).

**Conclusions:**
While the proportion of infants demonstrating progression of FMS did not differ significantly between early and later follow-up groups, a significant increase in stenosis severity was observed in those undergoing follow-up MRI after 50 weeks of age. These findings suggest that earlier follow-up imaging may detect progression before the development of more severe stenosis. Multidisciplinary discussion is required to determine the utility of MRI screening before 14 weeks of age to define the optimal timing of repeat imaging.

### Disclosure

None declared

**LB4**

Abstract withdrawn

### LB5

### Changes in Circulating Matrix GLA Protein During the Pubertal Transition in Healthy Children and Females with Central Precocious Puberty

Maria Bentz Damholt[1], Stine A Holmboe[1,2], Jørgen Holm Petersen[1,3], Martin Blomberg Jensen[4,5], Anders Juul[1,2,5], Li Juel Mortensen[1,2]

[1]Department of Growth and Reproduction, Copenhagen University Hospital - Rigshospitalet, Copenhagen, Denmark. [2]International Centre for Research and Research Training in Endocrine Disruption of Male Reproduction and Child Health (EDMaRC), Copenhagen University Hospital - Rigshospitalet, Copenhagen, Denmark. [3]Section of Biostatistics, Institute of Public Health, Faculty of Health Sciences, University of Copenhagen, Copenhagen, Denmark. [4]Department of Endocrinology and Internal Medicine, Division of Translational Endocrinolo-gy, Copenhagen University Hospital-Herlev and Gentofte, Herlev, Denmark. [5]Department of Clinical Medicine, University of Copenhagen, Copenhagen, Denmark

### Abstract

**Changes in circulating Matrix GLA Protein During the Pubertal Transition in Healthy Children and Females with Central Precocious Puberty**

Maria Bentz Damholt, Stine Agergaard Holmboe, Jørgen Holm Petersen1, Martin Blomberg-Jensen, Anders Juul, Li Juel Mortensen

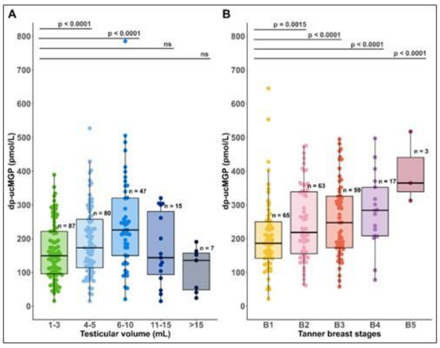

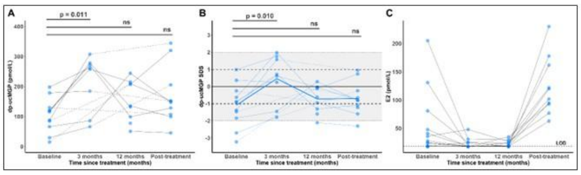

**Figure 5:** Levels of dp-ucMGP in girls with CPP before (baseline), during (3 and 12 months) treatment with GnRH analogue and 6 months after ending treatment (post-treatment). **(A)** shows progression of dp-ucMGP in pmol/L. **(B)** shows progression of dp-ucMGP SDS. **(C)** shows progression of E2 (pmol/L). Points connected by lines represent longitudinal measurements for each child. Dashed lines indicate that a measurement is missing between the connected points. Differences in dp-ucMGP concentrations across timepoints were compared to baseline (baseline vs. 3 months, 12 months, and post-treatment) using Dunnett's post hoc test.

**dp-ucMGP across pubertal stages in healthy children.** Box plot shows median and IQR. Individual measurements are shown as dots. Statistical analysis: Generalised Estimated Equations (GEE), Bonferroni-adjusted. **(A)** Levels of dp-ucMGP (pmol/L) by testicular volume (mL) in boys. **(B)** Levels of dp-ucMGP (pmol/L) by Tanner breast stages (B1-B5) in girls.

### Background:

Longitudinal bone growth occurs at the growth plate, where chondrocytes proliferate, differentiate, finally undergo apoptosis, enabling cartilage mineralisation. Matrix Gla protein (MGP) regulates extracellular matrix mineralisation including bone formation. MGP is expressed by chondrocytes, fibroblasts, and vascular smooth muscle cells.Expression in adipocytes has been suggested. Evidence suggests that plasma concentrations of dephosphorylated undercarboxylated MGP (dp-ucMGP) may vary across pubertal stages, potentially reflecting its role in bone remodelling.

### Methods:

This study examines longitudinal changes in circulating plasma dp-ucMGP in two cohorts: 111 healthy Danish children (52% girls) aged 6–15 years from the Copenhagen Puberty Study and 15 female patients with central precocious puberty (CPP) before, during, and after GnRH analogue treatment. Blood samples were obtained every 6 months, and pubertal stage was assessed using Marshall and Tanner criteria, including breast development and testicular volume. Dp-ucMGP was quantified using a commercially available automated immunoassay (IDS-iSYS InaKtif MGP) by IDS Immunodiagnostic Systems, UK. Age- and sex-specific reference curves for dp-ucMGP were generated using Generalised Additive Models for Location, Scale and Shape (GAMLSS), with standard deviation scores (SD), based on all healthy participants.

**Results:**

Dp-ucMGP increased significantly with age and pubertal progression, while it declined from mid-puberty exclusively in boys. In girls only, dp-ucMGP SD were associated with body weight and BMI. In girls with CPP, baseline dp-ucMGP concentrations were lower compared to healthy puberty-matched girls (Tanner B3: -1.93 SD vs. 0.05 SD, p<0.0001). Initiation of GnRH agonist in girls with CPP resulted in expected inhibition of estradiol; however, dp-ucMGP increased significantly (-1.21 SD (baseline) vs 0.30 SD (3 months), p=0.010), but declined towards baseline levels after 12 months of treatment.

**Conclusion:**

We provide sex- and age-specific reference intervals for dp-ucMGP concentrations in healthy children. Circulating dp-ucMGP increases with age and normal pubertal progression in healthy children. Dp-ucMGP trajectories in late puberty differed between sexes, and associations with BMI and body weight were evident only in girls. Dp-ucMGP was suppressed by GnRH agonist. Our findings support that circulating concentrations of dp-ucMGP parallels pubertal progression, suggesting a potential role in bone remodelling and linear growth.

### Disclosure

None declared

### LB6

### The Vertebral Fracture Phenotype Across the Age Spectrum in Osteogenesis Imperfecta Type 1: Results of the Brittle Bone Disorders Longitudinal Study

Rana Halloun[1], Rui Santos[2], Eoghan Laffan[3], Ramy El-Jalbout[4], Maya Scharke[1], Roozbeh Manesh[1], Rodrigo Montero-Lopez[1], Kerry Siminoski[1], Frank Rauch[5], Andrew Tice[1], Kevin Smit[1], Stefan Jackowski[1], Eric Orwoll[6], Cathy Raggio[7], Sandesh Nagamani[8], Reid Sutton[8], Brendan Lee[8], Leanne Ward[1], The Brittle Bone Disorders Consortium[9]

[1]Children's Hospital of Eastern Ontario, Ottawa, Canada. [2]Guy's and St Thomas NHS Foundation Trust, London, UK. [3]Children's Health Ireland at Crumlin, Dublin, Ireland. [4]Sainte-Justine Hospital and Research Center, Montreal, Canada. [5]Shriners Hospital for Children-Canada, Montreal, Canada. [6]Oregon Health & Science University, Portland, USA. [7]Hospital for Special Surgery, New York, USA. [8]Baylor College of Medicine, Houston, USA. [9]Brittle Bone Disorders Consortium, Houston, USA

### Abstract

**Objectives:**

Vertebral fractures (VF) are a key feature of osteogenesis imperfecta (OI), associated with back pain, height loss, and spine deformity. We sought to characterize VF prevalence, incidence, severity, and determinants of VF burden in treatment-naïve individuals with OI type I.

**Methods:**

This longitudinal multicenter study included treatment-naive participants with OI type I from the OI Linked Clinical Research Centers. Lateral spine radiographs were assessed according to the Genant semi-quantitative method for prevalent and incident VF. Fracture burden was quantified using the Spinal Deformity Index (SDI; sum of Genant-defined VF from T4–L4).

**Results:**

171 participants were included (median age 14.9 years (IQR 8.3–36.3, range 0.7–63.6, 36% male). Median height and BMI z-scores were -0.8 (IQR -1.4 to 0.02) and 0.5 (IQR -3.8 to 1.2), respectively. One hundred and fifty-seven prevalent VF (PVF) were present in 62/171 individuals (36%), including 29/80 children (36%, median age 9.5 years, range 0.7–14.9) and 38% of adults (median age 42.7 years, range 20.8–63.6). Of these, 51/157 PVF (32%) were moderate or severe. PVF clustered in the mid-thoracic and thoracolumbar junction regions, with anterior wedge morphology predominating (Figure 1). Twenty-four incident VF (IVF) occurred in six individuals (6%) after a median of 17 months (IQR 12–24). Among individuals with VF, the median SDI was 2.0 for PVF (IQR 1–5, range 1–26) and 4.5 for IVF (IQR 1–7, range 1–12). Thirty-nine percent (24/62) of participants with PVF had an SDI ≥4 (median 6.0, IQR 4–7, range 4–26); all of the severe VF were observed in this sub-group (median age 12.8, IQR 9.8–39.2, range 3.7–60.4). In age-stratified analyses, female sex and lower BMI z-scores were associated with a higher prevalent SDI in children ($p=0.01$ and $p=0.02$, respectively), while lower height z-scores were associated with a higher prevalent SDI in adults ($p=0.006$).

**Conclusion:**

VF were present in one-third of individuals with treatment-naïve OI, including 36% of children (youngest age <1 year). The finding of advanced vertebral collapse across the age spectrum underscores the importance of timely VF identification through routine surveillance in this high-risk population.

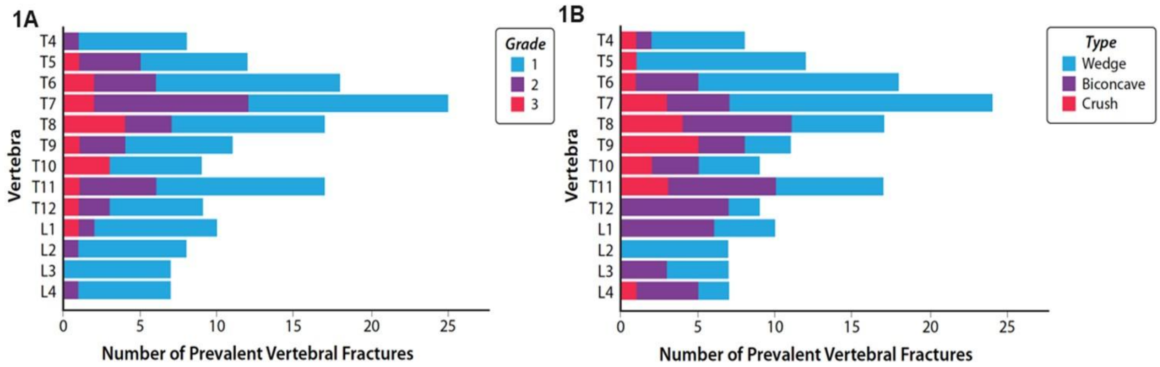

**Figure 1. Vertebral fracture distribution by severity (A) and morphology (B).**
(A) Distribution and severity (Genant semi-quantitative grades) of prevalent vertebral fractures across vertebral levels.
(B) Distribution of prevalent vertebral fractures by morphology type (wedge, biconcave, crush).

### Disclosure

None declared

### LB7

### Spine Deformity Across the Age Spectrum in Osteogenesis Imperfecta Type 1: Results of the Brittle Bone Disorders Study

Rana Halloun[1], Rui Santos[2], Eoghan Laffan[3], Ramy El-Jalbout[4], Maya Scharke[1], Roozbeh Manesh[1], Rodrigo Montero-Lopez[1], Kerry Siminoski[1], Frank Rauch[5], Andrew Tice[1], Kevin Smit[1], Stefan Jackowski[1], Eric Orwoll[6], Cathy Raggio[7], Sandesh Nagamani[8], Reid Sutton[8], Brendan Lee[8], Leanne Ward[1], The Brittle Bone Disorders Consortium[9]

[1]Children's Hospital of Eastern Ontario, Ottawa, Canada. [2]Guy's and St Thomas NHS Foundation Trust, London, UK. [3]Children's Health Ireland at Crumlin, Dublin, Ireland. [4]Sainte-Justine Hospital and Research Center, Montreal, Canada. [5]Shriners Hospital for Children-Canada, Montreal, Canada. [6]Oregon Health & Science University, Portland, USA. [7]Hospital for Special Surgery, New York, USA. [8]Baylor College of Medicine, Houston, USA. [9]Brittle Bone Disorders Consortium, Houston, USA

### Abstract

**Objectives:** Spine deformities in osteogenesis imperfecta (OI) are associated with surgical intervention and functional limitations. We aimed to characterize the spine deformity phenotype and its determinants in treatment-naïve individuals with OI type I.

**Methods:** This cross-sectional multicenter study included treatment-naïve individuals with OI type I from the OI Linked Clinical Research Centers. Lateral spine radiographs were assessed for spondylolysis, spondylolisthesis, hyperkyphosis (Cobb ≥ 40° in adults or z-score ≥ 2.0 in children), and hyperlordosis (Cobb ≥ 60° in adults or z-score ≥ 2.0 in children). Vertebral fracture (VF) burden was quantified using the Spinal Deformity Index (SDI; sum of Genant-defined VF from T4 to L4). Anteroposterior spine radiographs were assessed for scoliosis (Cobb ≥ 10°).

**Results:** 171 participants were included (median age 14.9 years, IQR 8.3–36.3, 36% male). Median height z-score was -0.8 (IQR -1.4–0.02). Thirty-six percent (35/96) had scoliosis, with similar prevalence in children (median age 10.2 years, IQR 8.2–11.9) and adults (median age 37.9 years, IQR 30.6–46.9). Scoliosis was predominantly mild (median Cobb angle 19.0°, IQR 15.0–28.5, range 11.0–46.0). Hyperkyphosis occurred in 8% (3/40) of children (median age 8.2 years, IQR 6.9–12.6) and 59% (16/27) of adults (median age 38.6 years, IQR 25.0–44.8). Hyperlordosis was present in 24% of children (median age 10.2 years, IQR 6.9–11.4) and 30% of adults (median age 35.2 years, IQR 28.8–43.0). In adjusted analyses, SDI was not associated with scoliosis, hyperkyphosis, or hyperlordosis, whereas age was associated with higher odds of hyperkyphosis (OR 1.08 per year, 95% CI 1.04–1.10; $p<0.001$). Hyperkyphosis was more frequent in those with hyperlordosis (48% vs 14%; $p=0.004$). Spondylolisthesis was observed in 8% (13/168), predominantly at L5/S1 (92%; all Grade 1 [75%] or Grade 2 [25%]). Spondylolysis was identified in 7% (11/165) and was strongly associated with spondylolisthesis (64% vs 4%; $p<0.001$).

**Conclusions:** Scoliosis was prevalent across the age spectrum, whereas hyperkyphosis increased with age and was associated with hyperlordosis, suggesting coupled and compensatory sagittal plane deformities. Spine deformities appeared to evolve independently of VF burden. These findings support evaluation of spine deformities across the age spectrum.

### Disclosure

None declared

### LB8

### Multicenter Real-World Evidence: Efficacy of Neridronate in Children affected by Osteogenesis Imperfecta - A Useful Starting Point for Future Research

Thomas Zoller[1], Alessandro Barbato[2,3], Ruggero Lanzafame[1], Chiara Zoller[1], Laura Zusi[1], Rossella Gaudino[1], Angelo Pietrobelli[1], Giulia Del Medico[2], Chiara Mengoni[2], Stefano Stagi[2,3], Franco Antoniazzi[1]

[1]Pediatric Unit, Department of Surgical Science, Dentistry, Gynecology and Pediatrics, Verona, Italy. [2]Endocrinology Unit, Meyer Children's Hospital IRCCS, Florence, Italy. [3]Department of Health Sciences, University of Florence, Florence, Italy

### Abstract

**Objectives:**

Bisphosphonates represent the cornerstone of medical treatment for children with Osteogenesis Imperfecta, although their use in pediatric patients is largely off-label. Among them, neridronate is approved in Italy for the treatment of children with OI.
This study aims to confirm the efficacy of neridronate in improving bone mineral density (BMD) and reducing fracture incidence in a multicenter pediatric cohort.

**Methods:**

A retrospective multicenter observational study was conducted including 69 pediatric patients with OI — 53 followed at the Azienda Ospedaliera Universitaria Integrata di Verona and 16 at the Ospedale Pediatrico Meyer in Florence, Italy. Clinical, genetic, and anthropometric data were collected. BMD was assessed by dual-energy X-ray absorptiometry (DXA) at the lumbar spine (LS), femoral neck (FN), and total femur (TF) at baseline (within 12 months before starting neridronate) and during follow-up (12, 24, and 36 months after therapy initiation). Data were analyzed using repeated-measures ANOVA. Fracture incidence was compared between the 24 months preceding and the 24 months following the start of neridronate therapy.

**Results:**

The cohort consisted mainly of patients with type I OI (80.9%) and type IV OI (14.7%), with a predominance of males (60.9%). Median age at diagnosis was 5.62 ± 4.13 years, with early disease onset and a mean age at first fracture of 3 ± 3.3 years. Genetic diagnosis was confirmed in all cases.
Treatment with neridronate led to a significant improvement in BMD at all sites (LS, FN, TF) over 36 months. Z-scores improved as follows:

- LS: $F(3,75) = 52.36$, $p < 0.001$; mean $LS_0 = -3.16 \pm 1.26$ → mean $LS_3 = -1.11 \pm 0.96$
- FN: $F(3,60) = 20.94$, $p < 0.001$; mean $FN_0 = -3.06 \pm 1.54$ → mean $FN_3 = -1.03 \pm 0.87$
- TF: $F(3,63) = 17.45$, $p < 0.001$; mean $TF_0 = -3.01 \pm 1.55$ → mean $TF_3 = -0.87 \pm 0.74$

Fracture incidence decreased from 112 events in the 24 months before treatment to 54 events in the 24 months after initiation (−51%, p = 0.002). The reduction was particularly significant at the femur (−60%, p = 0.017), while other sites showed a non-significant trend toward reduction. (Figure 1)

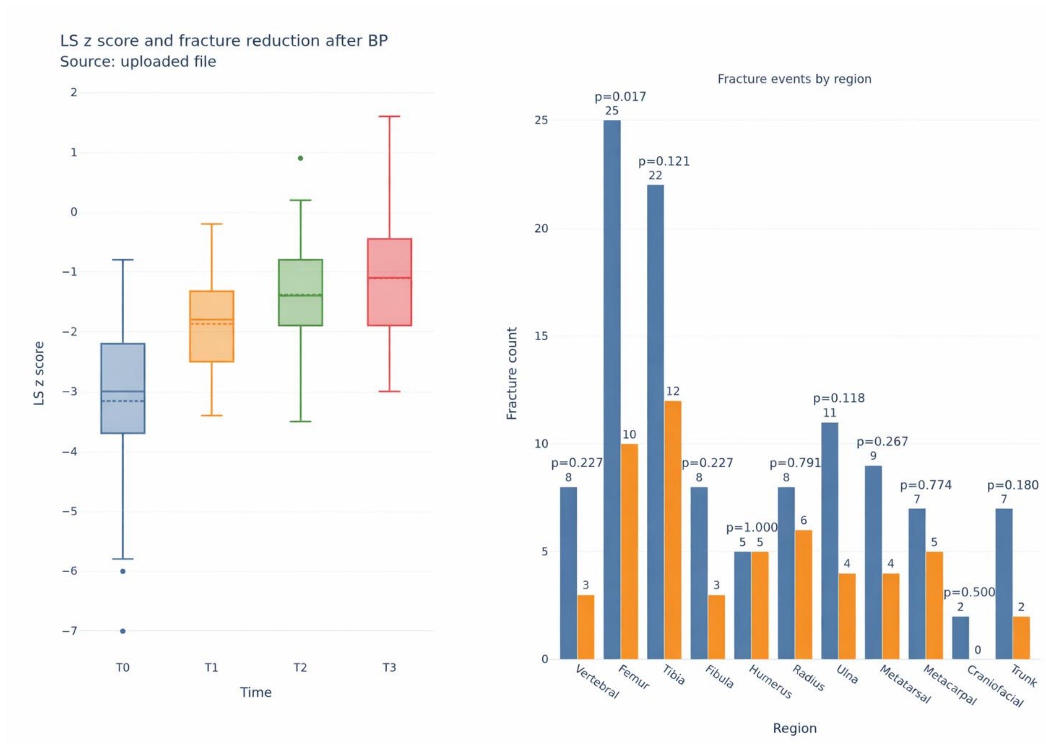

### Conclusion:

This multicenter real-world study demonstrates that neridronate is highly effective in children with OI, producing significant improvements in BMD and a substantial reduction in fracture incidence. These findings support its use as a valid therapeutic option in pediatric OI and provide robust real-world evidence to guide clinical practice and future prospective research.

### Disclosure

None declared

### LB9

### Clinically Significant Skeletal Deficits in Adults with Duchenne Muscular Dystrophy: Implications Beyond Childhood

Rana Halloun, Fahd Alshammri, Rodrigo Montero-Lopez, Nasrin Khan, Maya Scharke, Damian Rauch, Scott Walker, Stefan Jackowski, Leanne M Ward

Children's Hospital of Eastern Ontario, Ottawa, Canada

### Abstract

**Objectives:**

Osteoporosis among individuals with Duchenne muscular dystrophy (DMD) is a high-burden comorbidity associated with fractures, bone pain, and spinal deformity. We aimed to characterize skeletal health outcomes in adults with DMD, including fracture burden, areal (a) and volumetric (v) bone mineral density (BMD), and long-bone microarchitecture.

**Methods**:

This case series included the first six adults with DMD enrolled in an ongoing single-center cross-sectional study. Vertebral fracture (VF) burden was quantified using the Spinal Deformity Index (SDI; sum of the Genant-defined VF from T4 to L4). aBMD was assessed by DXA at the lumbar spine (LS; L1–L4), total hip, and total body. vBMD and microarchitecture were evaluated by high-resolution peripheral quantitative computed tomography (HR-pQCT) of the radius at the 4% (trabecular) and 30% (cortical) sites.

**Results:**

Median age was 25.1 years (IQR 24.5–30.7; range 18.8–32.8), median height z-score -2.6 (IQR -3.2 to -1.8; range -6.3 to -1.8). Five participants were receiving daily deflazacort; one was ambulatory. Nineteen VF were present in five participants (83%), of which 68% were moderate or severe. Median SDI was 2.0 (IQR 1.25 to 2; range 0 to 29); one participant had a markedly elevated SDI of 29. Three participants sustained four low-trauma long-bone fractures in adulthood (three femoral, one tibial). Three participants received intravenous zoledronic acid (ZA) therapy, one denosumab, one had discontinued ZA, and one was bone protection naïve. Median aBMD z-scores were as follows: LS -1.4 (IQR -3.0 to -0.3), total hip (n=4) -2.1 (IQR -2.3 to -2.0), and total body -3.5 (IQR -3.8 to -3.1) (Figure 1A). HR-pQCT was available in five participants (all reported as z-scores): median trabecular thickness -5.0 (IQR -9.0 to -1.1), median cortical thickness -4.0 (IQR -7.6 to -3.2), and median cortical vBMD 3.1 (IQR 2.0 to 4.2) (Figure 1B).

**Conclusions:**

Adults with DMD demonstrated substantial VF and non-VF burden and reduced BMD.
HR-pQCT revealed marked inter-individual variability, including profound trabecular and cortical thickness deficits. Variability in treatment exposure and skeletal outcomes supports further study to inform optimal bone health management in this high-risk group at a particular vulnerable phase of life.

**Figure 1: Skeletal Health Z-scores Across Participants by Imaging Modality**

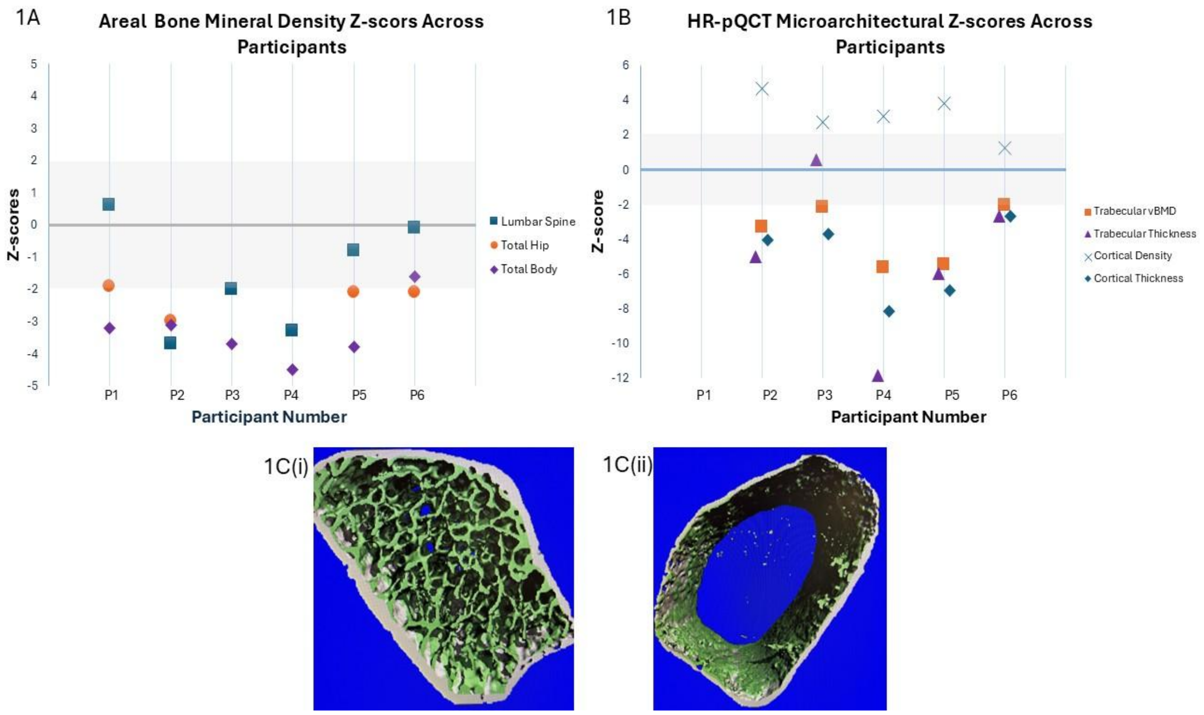

(1A) DXA-derived aBMD z-scores at the lumbar spine, total hip, and total body for all six participants.

(1B) HR-pQCT-derived density and microarchitectural z-scores at the distal radius, including trabecular vBMD and trabecular thickness at the 4% site plus cortical vBMD and cortical thickness at the 30% site. HR-pQCT data were available for five participants (participants 2–6)

(1Ci–ii) Representative distal radius (4% site) HR-pQCT reconstructions from participants 2 and 4 demonstrating variability in bone microarchitecture

### Disclosure

None declared

### LB10

### Osteocalcin Differentially Regulates Bone Marrow Fat Accumulation and Its Metabolic Coupling in Osteogenesis Imperfecta

Monica Sageder[1], Mathieu Ferron[2,3], Svetlana V Komarova[4,5,6], Josephine T Tauer[1,4]

[1]Department of Pediatrics and Adolescent Medicine, Johannes Kepler University, Linz, Austria. [2]Unité de Recherche en Physiologie Moléculaire, Institut de Recherches Cliniques de Montréal, Montréal, Canada. [3]Départements de Médecine et de Biochimie et Médecine Moléculaire, Université de Montréal, Montréal, Canada. [4]Research Centre, Shriners Hospital for Children-Canada, Montréal, Canada. [5]Faculty of Dental Medicine and Oral Health Sciences, McGill University, Montréal, Canada. [6]Department of Biomedical Engineering, Faculty of Engineering, University of Alberta, Edmonton, Canada

### Abstract

**Objective**: It is well established that the bone-derived hormone osteocalcin (OCN) plays a key role in systemic energy metabolism, influencing glucose homeostasis and adipose tissue function. However, its potential influence on bone marrow adiposity (BMA) remains unclear. Thus, we investigated whether OCN impacts BMA similarly to peripheral fat and how this relationship changes in the context of osteogenesis imperfecta (OI).

**Method**: Tibial BMA was quantified as the ratio of osmium-stained fat volume to tissue volume (OV/TV) in wildtype (WT), OCN-deficient (OCN-KO), OI, and OI/OCN-KO mice of both sexes after 26 weeks on a low-fat (LFD) or high-fat diet (HFD).

**Results:** Three-way ANOVA revealed significant effects of genotype, diet, and sex, with females showing higher OV/TV than males. In sex-specific analyses, no significant genotype- or diet-effects were observed in males, although HFD caused a modest increase in OV/TV across all groups. Conversely, females showed notable genotype- and diet-dependent differences: OCN-KO in WT mice led to a significantly increased OV/TV under HFD. Interestingly, OI females on HFD did not show HFD-driven bone marrow fat accumulation, but this resistance was lost after OCN-KO.

To investigate the relationship between BMA and systemic metabolic parameters, Pearson correlation was performed, revealing genotype- and sex-specific patterns. In WT and OCN-KO male mice, BMA showed no significant association with metabolic measures; while OI males displayed positive correlations between OV/TV and body mass, abdominal fat, and adipose tissue weights (inguinal WAT, BAT), suggesting a close metabolic link. These correlations were absent in male OI/OCN-KO mice. Among females, BMA's associations varied by genotype: WT mice showed no significant correlations, but OCN-KO linked OV/TV to abdominal adiposity. In OI, OV/TV positively correlated with BAT mass, and OCN-KO resulted in a positive correlation between OV/TV and fasting glucose levels, along with a trend toward positive correlation with abdominal fat.

**Conclusion**: Overall, these findings suggest that OCN functions as a context-dependent regulator of BMA in OI. It promotes metabolic integration of BMA in males while restraining BMA in females, indicating a dual, sex-specific role. Therefore, OCN likely acts within a sex hormone–dependent network, exerting distinct effects on peripheral fat and BMA.

### Disclosure

Funding source: Project was funded by a CIHR research grant (#74421). No conflicts of interest.

### LB11

### Atypical Bilateral Slipped Capital Femoral Epiphysis as the Initial Presentation of Primary Hyperparathyroidism in an Adolescent

Vanessa Sambati[1,2], Milena Walther[1,2], Diana-Alexandra Ertl[1,2,3], Khadija Fouatih[1,2], Laurène Delacourt[1,2], Julia Royer[4], Valerie Merzoug[5], Anya Rothenbuhler[1,2], Agnès Linglart[1,2,3]

[1]AP HP, Department of Endocrinology and Diabetology for Children and Department of Adolescent Medicine, Bicêtre Paris-Saclay University Hospital, Le Kremlin Bicêtre, France. [2]AP HP, Reference Center for Rare Disorders of The Calcium and Phosphate Metabolism, Filière OSCAR and Platform of Expertise for Rare Diseases Paris-Saclay, EndoRare and BOND ERN, Bicêtre Paris Saclay Hospital, Le Kremlin Bicêtre, France. [3]Université Paris-Saclay, Inserm UMR-S 1185, Physiologie et Physiopathologie Endocriniennes, Le Kremlin Bicêtre, France. [4]Department of Pediatric Orthopaedic and Reconstructive Surgery, Hôpital Armand-Trousseau, AP-HP, Sorbonne University, Paris, France. [5]Department of Pediatric Radiology, Paris-Sud Rare Diseases Expert Center, OSCAR Network, Bicêtre Paris-Sud Hospital, AP-HP, Paris-Saclay University, Paris, France, Le Kremlin Bicêtre, France

### Abstract

**Background:** Primary hyperparathyroidism (PHPT) is rare in children but may significantly affect bone metabolism. Slipped capital femoral epiphysis (SCFE) is an uncommon presenting feature and may delay diagnosis.

**Presenting problem:** A 12-year-old boy presented with low body weight (BMI 14.9 kg/m²), reported weight loss and progressive limping associated with evident lower limb deformity. Pelvic radiography demonstrated bilateral SCFE, which was surgically treated. Postoperatively, a biochemical workup prescribed by a pediatrician, who was consulted due to weight loss and abnormal radiographic findings, revealed hypercalcemia, with a peak level of 4.32 mmol/L. Hypercalcemia prompted further investigations, leading to the diagnosis of primary hyperparathyroidism due to a parathyroid adenoma, localized by choline PET scintigraphy and parathyroid ultrasonography.

**Clinical management of hypercalcemia**

Hypercalcemia was managed with intravenous bisphosphonates. Parathyroidectomy is planned, and genetic testing is ongoing to guide surgical management in this suspected HRPT2-related case.

**Discussion:** Atypical SCFE is a rare manifestation of paediatric PHPT with only a limited number of cases reported in the literature. Most cases involve adolescents frequently presenting with bilateral disease and delayed endocrine diagnosis. This case is notable for atypical features, including low BMI and weight loss, contrasting with the typical association between SCFE and obesity. It underscores the importance of an endocrine evaluation in orthopaedic conditions potentially related to metabolic bone disorders. Early recognition of PHPT is essential to guide appropriate management and prevent further skeletal complications. This case also emphasizes the importance of genetic evaluation, which may influence surgical strategy in paediatric PHPT.

Orthopaedic surgeons should be aware that bilateral slipped capital femoral epiphysis may be associated with hyperparathyroidism; therefore, a metabolic workup, including measurement of serum calcium levels, should be performed.

### Disclosure

None declared

### LB12

### Tibial Length Measurement Via Dual Energy X-ray Absorptiometry Based Images vs. Standing Height in Young Ambulatory Children with Duchenne Muscular Dystrophy

Fahd Alshammri[1,2], Rana Halloun[1,2], Stefan Jackowski[2], Maya Scharke[2], Utkarsh Dang[3], Scott Walker[2], Kerry Siminoski[2], Joanne Donovan[4], Eric Hoffman[5,6], Leanne Ward[1,2]

[1]University of Ottawa, Ottawa, Canada. [2]Children's Hospital of Eastern Ontario Research Institute, Ottawa, Canada. [3]Carleton University, Ottawa, Canada. [4]Edgewise Therapeutics, Boulder, USA. [5]ReveraGen BioPharma, Rickville, USA. [6]School of Pharmacy and Pharmaceutical Sciences, Binghamton University - State University of New York, Binghatmon, USA

### Abstract

**Background:** Accurate height measurement in Duchenne muscular dystrophy (DMD) can be challenging, particularly in non-ambulatory children with contractures. Tibial length (TL) derived from dual-energy X-ray absorptiometry (DXA) may offer an imaging-based approach for estimating standing height.

**Objective:** To conduct a pilot study evaluating a DXA-based TL equation for estimating standing height in young ambulatory boys with DMD.

**Methods:** Participant-level data were pooled from two DMD clinical trials (NCT03703882 and NCT02760264). Ambulatory boys aged 4–8 years underwent concurrent standing height measurement and total body DXA at baseline (Visit 1 [V1]; n=185), 24 weeks (V2; n=71), and 48–52 weeks (V3; n=133). Mean bilateral DXA-derived TL was regressed on standing height at V1 using ordinary least squares. Cross-sectional accuracy was assessed as the percent deviation of the DXA-derived estimated height from the nominal standing height of each child, expressed as mean ± %. Internal validity was assessed by five-fold cross-validation. Temporal validity was assessed by applying the baseline equation to V2 and V3 without re-fitting. Bland–Altman analysis evaluated agreement at each visit.

**Results:** TL correlated strongly with standing height across all visits (r = 0.938–0.956; all $p<0.001$). The derived equation — Estimated Height (cm) = 33.04 + 3.48 × TL (cm) — explained 87.9% of height variance ($R^2 = 0.879$). The mean accuracy was ±2.0% at V1, indicating high accuracy. Cross-validation showed consistent performance (root mean square error (RMSE) = 2.86 cm; mean bias = 0.01 cm). When applying the equation longitudinally to V2 and V3 without modification, mean accuracy remained stable at ±2.0% and ±2.2%, with RMSE of 2.74 cm and 3.07 cm and mean biases of 0.54 cm and 1.31 cm, respectively. Approximately 91% and 89% of estimates fell within 5 cm of measured height at V2 and V3.

**Conclusion:** A simple linear equation derived from DXA-based TL demonstrated high cross-sectional accuracy (±2.0%) for estimating standing height in ambulatory boys with DMD, with stable performance maintained longitudinally over approximately one year without equation re-fitting.

### Disclosure

UJD has in the past served as an ad-hoc consultant for ReveraGen Biopharma. All other authors have no disclosures to report.

### LB13

### Prevalence of Autosomal Dominant Osteopetrosis Type 2 (ADO; Albers-Schönberg disease) Calculated from the Allele Frequency of ADO-Causing Chloride Channel 7 (CLCN7) Variants in gnomADv4.1.1

Deborah Wenkert[1], Michael Econs[2], Ravi Savarirayan[3], Erik Imel[2]

[1]ashibio, Inc., Burlingame, USA. [2]Indiana University School of Medicine, Indianapolis, USA. [3]Royal Children's Hospital, and University of Melbourne, Parkville, Australia

### Abstract

OBJECTIVES:

ADO is typically caused by heterozygous loss-of-function variants in *CLCN7* with poorly functioning osteoclasts & pathognomonic x-ray findings (rugger jersey spine, bone-in-bone, Erlenmeyer flask-shaped distal femurs). Fragility fractures in a dense skeleton, chronic pain, & osteoarthritis are common; dental issues, visual loss, deafness, facial nerve paralysis, bone marrow failure, & osteonecrosis can occur. The published ADO prevalence (1/20,000) is based on Funen County, Denmark (N~450,000) hospital records (Bollerslev 1989). We estimated CLCN7-related ADO disease prevalence using the Genome Aggregation Database (gnomAD™), a genetic database from worldwide, large-scale sequencing projects.

METHODS:

ADO-related *CLCN7* variants were identified from the literature and supplemented with Pathogenic & Likely Pathogenic variants from ClinVar (with patient data supportive for ADO, excluding those related only to recessive disease). Allele frequencies (AF) were determined in gnomAD v.4.1.1 for each identified variant [gnomAD has >30 sequencing reads across CLCN7 (gene or exome) for ~80% of 807,162 unique individuals].

Clinical ADO prevalence was calculated as: [(# individuals with a *CLCN7* ADO variant)/(# sequenced for *CLCN7)*] x penetrance (66%). Note: published ADO penetrance=56-90%.

RESULTS:

18 (17 missense, 1 splice acceptor site) of 53 previously published ADO *CLCN7* variants, & 3 additional ClinVar Pathogenic or Likely Pathogenic ADO *CLCN7* variants were identified in gnomAD. The 18 published variants were either ClinVar classified as Pathogenic &/or Likely Pathogenic (n=9) &/or associated with pathognomonic ADO x-rays (n=9) & those with DXAs had BMD Z-scores >7.7. R674Q, classified as conflicting in ClinVar, was 3x more common in gnomAD than the next most common variant.

ADO disease prevalence using three different calculations were:

- 0.7/20,000 (using only the 12 ClinVar category Pathogenic (P) or Likely Pathogenic (LP) ADO CLCN7 variants in gnomAD (9 published & 3 additional from ClinVar)
- 3.8/20,000 (using only the 18 published variants in gnomAD)
- 4.1/20,000 (using 18 published & 3 additional P or LP variants in ClinVar)
    1. 1.7/20,000 (excluding the most common ADO CLCN7 variant in gnomAD (R674Q), in case this variant has a different penetrance)

CONCLUSIONS:

Our analysis of gnomAD v.4.1.1, using different variant inclusion criteria, estimates worldwide *CLCN7*-related ADO disease prevalence to be ~1-4/20,000, a range consistent with prior publications.

| Publications sources for ADO-related *CLCN7* variants | |
|---|---|
| **First author, year** | **PIMD** |
| Cleiren 2001 | 11741829 |
| Frattini 2003 | 14584882 |
| Waguespack 2003 | 12929941 |
| Letizia 2004 | 14564431 |
| Campos-Xavier 2005 | 15633192 |
| Waguespack 2007 | 17164308. |
| Pangrazio 2009 | 19953639 |
| Wang 2012 | 21947783 |
| Rashid 2013 | 23302420 |
| Zheng 2014 | 23953223 |
| Deng 2016 | 27325559 |
| Pang 2016 | 26395888 |
| Zhang 2017 | 28975865 |
| Li 2019 | 30942407 |
| Kang 2019 | 30759959 |
| Bug 2020 | 33105733 |
| Wang 2022 | 35370969 |
| Jiang 2025 | 40276109 |
| Econs 2025 | 40913471 |

**Disclosure**

DW: Is a fulltime employee of āshibio MJE: Has received research funding and consulting fees from āshibio. He has also received past research funding and consulting fees from Alexion. RS: Has received consulting fees and grants from BioMarin, Ascendis, QED, and Tyra EAI: Has received consulting fees from āshibio unrelated to this work

### LB14

### X-Chromosome Inactivation and Genetic Diagnosis of Musculoskeletal Disorders in Females: The Case of *PHEX*

Juliana Marulanda[1], Zenghui Wu[1], Frank Rauch[1,2]

[1]Shriners Hospital for Children, Montreal, Canada. [2]McGill University, Montreal, Canada

### Abstract

**OBJECTIVES:**

X-chromosome inactivation is the mechanism by which mammalian cells balance gene dosage in females by silencing one X chromosome. This process varies across cell types, individuals, and age. This inactivation event not only plays a role in the pathogenesis of many diseases but interferes with the process of molecular diagnosis of genetic conditions linked to the X chromosome. Here, we describe a case in which a female with X-linked hypophosphatemia (XLH) was genetically diagnosed using amplicon-based RNA analysis from blood, whereas analysis of RNA from urine-derived cells (UDCs) failed to detect the variant.

**METHODS:**

RNA was extracted from both UDCs and blood and transcribed to cDNA. The cDNA was amplified by PCR, followed by sequencing on a NextSeq 2000 device (Illumina) and bioinformatics analysis.

**RESULTS:**

The proband is a 14-year-old girl with a clinical diagnosis of XLH. DNA sequencing using rickets and skeletal dysplasia gene panels did not reveal any disease-causing variants. Analysis of the *PHEX* transcript in RNA extracted from UDCs did not reveal any abnormalities. In contrast, the same approach applied to blood RNA revealed a pseudoexon within intron 21, corresponding to an 84 bp insertion between exons 21 and 22 (**Figure 1**). Subsequent Sanger sequencing of genomic DNA identified a heterozygous *PHEX* (NM_000444.6) c.2147+1197A>G variant, which creates a cryptic splice donor site.

**DISCUSSION:**

Analysis of the *PHEX* transcript from UDCs failed to detect the pathogenic splice variant even though the area was covered by thousands of reads in the sequencing of cDNA. This likely reflects clonal expansion of UDCs from a single progenitor cell, resulting in a colony that is genetically identical to the original founder cell. Consequently, all UDCs in the test sample have inactivation of the same X-chromosome. In contrast, RNA analysis from blood revealed the presence of the pseudoexon in more than 50% of sequencing reads, consistent with the heterogeneous X-chromosome inactivation pattern present in blood. These findings highlight a pitfall in the molecular diagnosis of X-linked disorders when RNA is analyzed from clonally expanded cells.

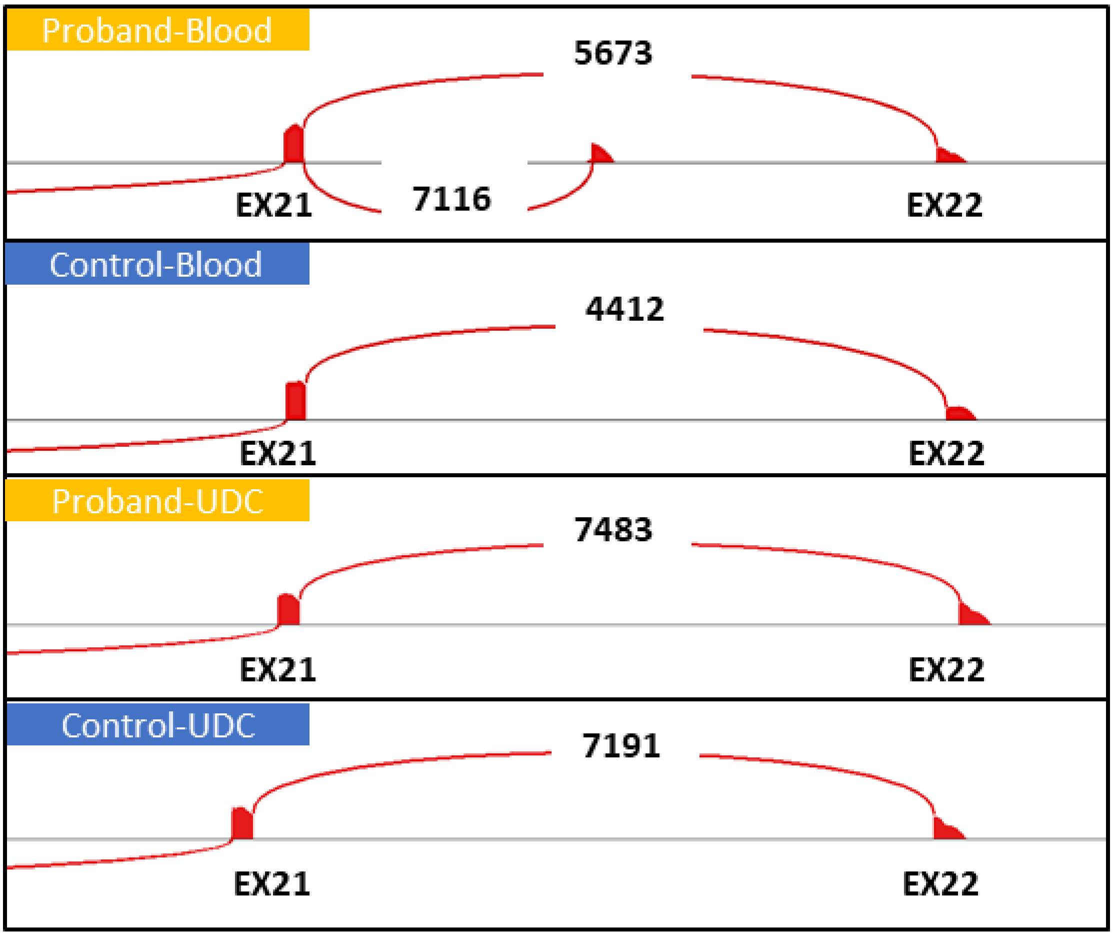

**Figure 1:** Sashimi plot showing inclusion of a pseudoexon within *PHEX* intron 21 in the proband's blood sample. The pseudoexon accounts for approximately 55% of reads in the proband and is absent in the control blood sample. In contrast, splicing appears normal in the proband's urine-derived cells (UDCs), with no reads supporting pseudoexon inclusion.

### Disclosure

None declared

### LB15

### Burosumab vs Conventional Therapy in Prepubertal XLH: Catch-Up Growth and Prognostic Value of an Early Response

Rodrigo Montero-Lopez[1,2], Damian Rauch[2], Maya Scharke[2], Rana Halloun[1,2], Lisa Dilworth[3], Joel Hetzer[3], Stefan Jackowski[2], Thomas O Carpenter[4], Jill H Simmons[5], Erik Imel[6], Nasrin Khan[2], Leanne M Ward[1,2]

[1]Department of Pediatrics, University of Ottawa, Division of Endocrinology, Children's Hospital of Eastern Ontario, Ottawa, Canada. [2]The Ottawa Pediatric Bone Health Research Group, Children's Hospital of Eastern Ontario Research Institute, Ottawa, Canada. [3]Ultragenyx Pharmaceutical Inc., Novato, CA, USA. [4]Yale University School of Medicine, New Haven, CT, USA. [5]Vanderbilt University, School of Medicine, Nashville, TN, USA. [6]Indiana University School of Medicine, Indianapolis, IN,, USA

### Abstract

**Objective**:

Children with X-linked hypophosphatemia (XLH) have impaired linear growth from early childhood. Long-term comparative growth data for burosumab versus conventional therapy (CT) are limited. This study compared long-term prepubertal growth trajectories in children with XLH treated with burosumab or CT and explored predictors of growth response to burosumab.

**Methods:**

This sub-analysis used data from the XLH Disease Monitoring Program (NCT03651505). Baseline was defined as the first study visit ±0.5 years of treatment initiation (burosumab group), and the first observation at age ≥2 years (CT group). Eligible participants were prepubertal (Tanner stage 1) and aged 2–11 years at baseline, with at least 2 years of follow-up. For this sub-analysis, follow-up ended at age 12 years or Tanner stage ≥2. Height Z-score (HtZ) trajectories were compared between groups using inverse probability of treatment weighting (IPTW). Predictors of burosumab response were assessed by age- and sex-adjusted linear regression and Pearson correlation.

**Results:**

Among 257 prepubertal children with XLH, 125 received burosumab and 132 CT; 56% and 72% were female, respectively. Mean (SD) baseline age was 6.1 (2.2) years with burosumab and 5.2 (3.0) with CT; mean follow-up was 3.5 years (SD 1.4). Median (range) baseline HtZ was -1.5 (-5.6 to 1.2) and -1.6 (-5.4 to 1.8), respectively. Median (range) baseline total RSS was 1.0 (0.0 to 5.5) with burosumab and 2.0 (0.0 to 6.0) with CT. Longitudinal unadjusted analyses showed greater improvement in HtZ from baseline with burosumab than CT (Figure 1A). In the IPTW-adjusted model, mean (SD) change in HtZ from baseline to year 3 was 0.47 (0.10) for burosumab and 0.16 (0.10) for CT (Figure 1B). Lower baseline HtZ ($\beta$=-0.12; $p$=0.002) and longer treatment duration ($\beta$=0.24; $p$<0.0001) independently predicted greater HtZ improvement, whereas baseline RSS was not associated with outcome. Early response to burosumab, measured by change in HtZ at 1 year after treatment initiation, correlated with change at last observation ($r$=0.381, $p$=0.001).

**Conclusions:**

Among prepubertal children with XLH, burosumab was associated with greater improvement in HtZ than CT over time, consistent with catch-up growth. Early HtZ response appears to be informative in predicting sustained long-term growth benefit.

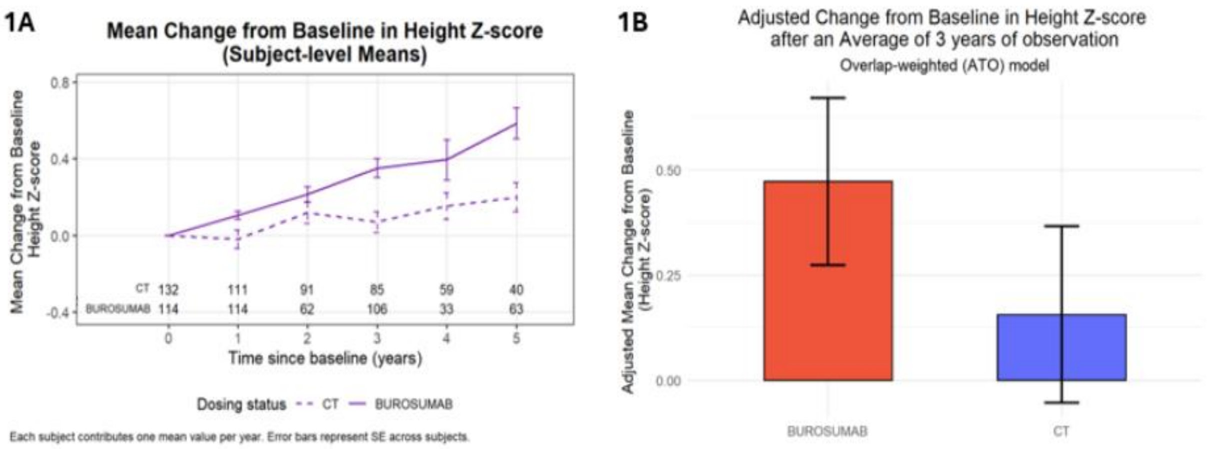

### Disclosure

LMW: Consultant to Ultragenyx and Kyowa Kirin, and participation in clinical trials with Ultragenyx (with funds to Dr. Ward's institution). LD, JH: Employee, Stockholder: Ultragenyx Pharmaceutical Inc. TOC: Consultant to Kyowa Kirin, BioMarin and Ultragenyx, and received research support (through grants to Yale University) from Ultragenyx and BioMarin. J.H.S.: Consultant fees and research grants from Kyowa Kirin and Ultragenyx. EAI: Consultant fees and research grants from Kyowa Kirin and Ultragenyx. No other disclosures.

### LB16

### GH-treatment increased Osteocalcin and Bone Mineral Content in short children born SGA (NESGAS)

Li Juel Mortensen[1,2], Edna Roche[3,4], Hilary MCV Hoey[3], Ajay Thankamoney[5], Jeremy Kirk[6], Rikke Beck Jensen[7,8], Anders Juul[1,2,8]

[1]Department of Growth and Reproduction, Copenhagen University Hospital - Rigshospitalet, Copenhagen, Denmark. [2]International Centre for Research and Research Training in Endocrine Disruption of Male Reproduction and Child Health (EDMaRC), Copenhagen University Hospital - Rigshospitalet, Copenhagen, Denmark. [3]Department of Paediatrics, School of Medicine, The University of Dublin Trinity College, Dublin, Ireland. [4]Paediatric Endocrinology and Diabetes, CHI at Tallaght, Dublin, Ireland. [5]Children’s Services, University Hospitals, Cambridge, UK. [6]Department of Endocrinology, Birmingham Children's Hospital, Birmingham, UK. [7]Department of Paediatrics, Herlev University Hospital, Herlev, Denmark. [8]Department of Clinical Medicine, University of Copenhagen, Copenhagen, Denmark

### Abstract

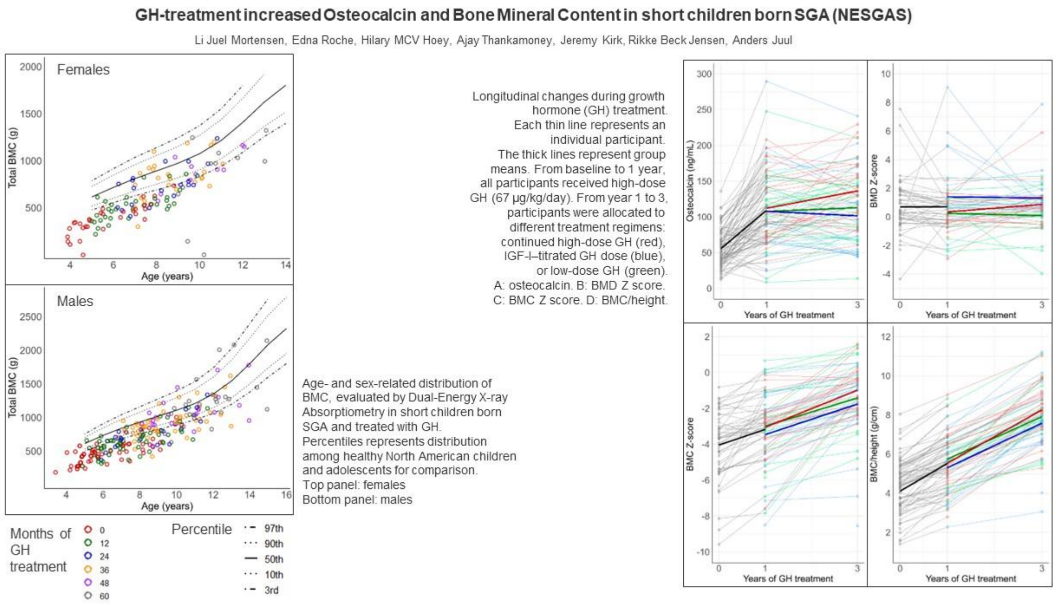

### Introduction:

Children born small for gestational age (SGA) without catch-up growth have preserved endogenous growth hormone (GH) secretion. However, treatment with GH to supraphysiological levels promotes linear growth and increases muscle- and bone mass. Additionally, GH treatment in children born SGA significantly reduces insulin sensitivity. Osteocalcin (OC), produced by osteoblasts, reflects bone formation, and increases with bone growth. OC is also inversely linked to insulin sensitivity. We hypothesized that OC was lower in children born SGA, and positively associated with growth response and increase in bone mass during GH treatment.

### Methods:

Prepubertal short children born SGA from the North European Small for Gestational Age Study (NESGAS) received a high-GH dose (67µg/kg/day) during first year. In years 2 and 3, they were randomized to 1) continued high-dose, 2) standard GH dose (35 µg/kg/day), or 3) IGF-I titrated GH dose. In 86 participants (33 females), we explored OC at baseline, after 12, and 36 months, in 292 samples, range 9-289 ng/mL; and determined bone mineral content (BMC) and bone mineral density (BMD) by dual-energy X-ray absorptiometry (DXA) at baseline, after 12, 24, and 36 months.

**Results:**

OC increased significantly from baseline (mean 56 ng/mL [47–64 ng/mL]) to 1 year (109 ng/mL [100–117], p<0.001). However, stratifying according to treatment between 1 year and 3 years, OC further increased significantly only in the high-dose group (from 112 ng/mL [93–131] to 137 ng/mL [120–155], p = 0.008), whereas remaining groups plateaued. 42% of baseline OC samples were below reference-interval for age, while 1.5% were above; after 12 months, 10% were below and 16% above.

Mean baseline BMD Z-score was 0.78 (0.39–1.24) and did not change significantly during the study in any treatment group. Baseline, BMC Z-score was -4.41 (-4.64 to -3.63) and BMC/height was 4.1 g/cm (3.7–4.5); both increased significantly after 1 year to respectively -3.23 [-3.62 to -2.83], p = 0.008; and 5.5 g/cm [5.1—5.9], p< 0.001. Both BMC Z-score and BMC/height increased further from 1 to 3 years in all groups, with no between-group differences in delta-values (36 months: -1.41 [-1.84 to -0.98]; 7.9 g/cm [7.4 – 8.4], both p < 0.001)

**In conclusion:**

Despite preserved endogenous GH secretion, short children born SGA did benefit from GH treatment with increased bone formation assessed by OC and normalized BMC, regardless of treatment regimen.

### Disclosure

None declared

### LB17

### Prolonged Hypercalcemia in an Adolescent with Diabetes Inspidus on the Ketogenic Diet

Rachel Marks[1], Lauren Yauch[2]

[1]University of Minnesota, Minneapolis, USA. [2]Gillette Children's Hospital, Saint Paul, USA

### Abstract

The ketogenic diet (KD) is a common therapy for refractory epilepsy in children. Hypercalcemia has been reported as a rare complication. A prior case series reported KD-associated moderate-severe hypercalcemia with suppressed parathyroid hormone (PTH); most resolved rapidly with hydration followed by calcitonin and bisphosphonates based on severity. We present a case of prolonged mild hypercalcemia on KD complicated by diabetes insipidus (DI).

**Case Report**

A 14 year old male with Hartsfield syndrome, DI, holoprosencephaly, G-tube dependence, and epilepsy began KD at age 10. He developed nephrolithiasis within 6 months. Mild hypercalcemia (Calcium (Ca) 11.9mg/dL) noted four years later during an acute kidney injury(AKI) and improved with intravenous fluids(IVF). Feeds adjusted and DDAVP increased for polyuria.

A year later he was hospitalized for AKI and hypercalcemia (Ca 12.0 mg/dL) due to feeding intolerance, polyuria, and reduced free water (FW). PTH and vitamin D 1,25 were suppressed, alkaline phosphatase was low. Ca normalized with IVF. FW and DDAVP were increased. He was readmitted three days later with worsening AKI and hypercalcemia. Again FW was increased. He developed feeding intolerance and was readmitted with hypercalcemia ten days later. Despite mild hypercalcemia, he was given calcitonin and pamidronate 0.25 mg/kg to lower FW requirement. Ca improved, and FW was decreased. DDAVP dose maintained. After Ca normalized, polyuria and feeding intolerance improved.

Two weeks later he developed hyponatremia (123 mg/dL), resolving after lowering both FW and DDAVP. Ca has remained stable for 1.5 months on reduced FW and DDAVP.

**Conclusions**

This case is unique in that mild hypercalcemia contributed to symptoms of polyuria and feeding intolerance leading to inappropriate increases in DDAVP dose and FW volume. Once hypercalcemia resolved, improved polyuria then resulted in hyponatremia.

Although hyperhydration is the standard treatment for mild hypercalcemia, this case demonstrates that bisphosphonates should be considered regardless of hypercalcemia severity. While bisphosphonates prevent hypercalcemia complications, they may have little impact on KD bone health as the low alkaline phosphatase levels in this case and others suggest KD is associated with decreased osteoblast bone formation. Additional studies are needed to develop a strategy for optimizing bone health in children on KD.

Disclosure none declared

### LB18

### Successful Management of a Toxic Zoledronic Acid Dose Erroneously Infused in 2 Siblings with Osteogenesis Imperfecta

Abdulmajeed AlSubaihin

King Saud University, Riyadh, Saudi Arabia

### Abstract

**Background:**

Antiresorptive therapy with bisphosphonates is the first line treatment in children with osteogenesis imperfecta. Among bisphosphonates, Zoledronic Acid (ZA) is advantageous as it can be infused over 30 minutes every 6 months minimizing the number of hospital days for children with OI. Being an intravenous drug, the steps of prescription, preparation, dilution and infusion of ZA are however amenable to error. Case reports of Zoledronic acid overdosing in children are lacking. In this report, the outcome and management of a toxic ZA dose erroneously infused in 2 siblings with osteogenesis imperfecta is discussed.

**Presenting problem:**

Two siblings, a 4-year-old girl (15kg) and a 16-month-old boy (11.6 kg), with OI where admitted for ZA infusion. The older sibling had received 2 cycles of ZA while the younger sibling was Bisphosphonate naïve. Four mg were infused erroneously in both siblings. That is 5 times the intended dose in the older sibling and 27 times the intended dose in the younger sibling.

**Clinical Management:**

The error was identified 2 hours following the end of the infusion. The error was immediately reported to the clinical pharmacist on duty. Both siblings where kept under monitoring in hospital. Oral calcium, magnesium and active vitamin D supplements where initiated. IV infusion of normal saline at maintenance rate was also commenced. Both siblings developed hypocalcemia and hypophosphatemia. Maintaining Calcium level in the younger sibling however required continuous IV calcium for 48 hours. No other side effects were observed.

**Discussion:**

This report provides insights on the effect of large ZA doses in children. The observed toxic effects of Zoledronic acid were limited to mineral metabolism and fortunately did not cause any cardiac or renal complications. The peak effect of ZA infusion on minerals was observed on day 3. A Lagging secondary hyperparathyroidism extended however to a month after infusion despite oral calcium supplements. The dilution of ZA (4mg in 100 ml of D5W) in our infusion protocol may have prevented the severe effects of ZA toxicity. Severe hypocalcemia may have been minimized by the avoidance of phsophorus supplement in our patients.

**Conclusion:**

Although appears to have a wide margin of safety, a model to prevent medication administration errors of ZA are highly recommended.

### Disclosure

None declared

### LB19

### Clinical and Biochemical Differences Between Hypocalcemic and Hypophosphatemic Rickets: A Multicenter Italian Pediatric Cohort

Alessia Angelelli[1], Danilo Fintini[2], Tommaso Aversa[3,4], Enrica Bertelli[5], Vincenza Carbone[6], Marta Calvo[7], Sara Cencioni[8], Domenico Corica[3], Lara Devilli[9], Maria Felicia Faienza[10], Adalgisa Festa[11], Roberto Franceschi[12], Anna Grandone[13], Mario Giordano[6], Anastasia Ibba[14], Millaann Kalapurackal[15], Federica Mela[8], Giulia Mirra[2], Marco Pitea[16], Elisa Sala[16], Andrea Secco[5], Grazia Maria Ubertini[2], Francesco Vierucci[17], Malgorzata Wasniewska[3], Daniele Tessaris[18,19], Luisa De Sanctis[7,19], Natascia Di Iorgi[1,8]

[1]Department of Pediatrics, IRCCS Istituto Giannina Gaslini, Genova, Italy. [2]Endocrinology and Diabetology Unit, Pediatric University Department, Bambino Gesù Children's Hospital, IRCCS, Rome, Italy. [3]Department of Human Pathology of Adulthood and Childhood «G. Barresi», University of Messina, Messina, Italy. [4]Unit of Pediatrics, University Hospital «G. Martino» of Messina, Messina, Italy. [5]Pediatrics and Pediatric Emergency Unit, AOU SS. Antonio e Biagio e C. Arrigo, Alessandria, Italy, Alessandria, Italy. [6]Pediatric Nephrology and Dialysis Unit, "Giovanni XXIII" Hospital, Bari, Italy. [7]Pediatric Endocrinology, Regina Margherita Children Hospital , Torino - Department of Public Health and Pediatric Sciences, University of Torino, Torino, Italy. [8]Department of Neurosciences, Rehabilitation, Ophthalmology, Genetics, Maternal and Child Health (DINOGMI), University of Genova, Genova, Italy, Genova, Italy. [9]UOM Pediatria, Pediatric Department, Ospedale S. Chiara di Trento, ASUIT, Trento, Italy, Trento, Italy. [10]Pediatric Unit, Department of Precision and Regenerative Medicine and Ionian Area, University of Bari "A.Moro", Bari, Italy. [11]UOC di Pediatria Generale e Specialistica, AOU Università della Campania Luigi Vanvitelli, Napoli, Italy. [12]UOM Pediatria, Pediatric Department, Ospedale S. Chiara di Trento; Centro Interdipartimentale di Scienze Mediche - CISMed, Univerity of Trento, Trento, Italy. [13]Department of Woman, Child and General and Specialized Surgery, University of Campania "Luigi Vanvitelli", Napoli, Italy. [14]Paediatric Endocrine Unit, Paediatric Hospital Microcitemico "A. Cao", AO Brotzu, Cagliari, Italy. [15]Department of Pediatrics, Hospital of Bolzano (SABES-ASDAA), Teaching Hospital of Paracelsus Medical University, Bolzano, Italy. [16]Pediatric Endocrinology Unit, Department of Pediatrics, IRCCS Ospedale San Raffaele, Milano, Italy. [17]Pediatric Unit, San Luca Hospital, Lucca, Italy. [18]Pediatric Endocrinology, Regina Margherita Children Hospital, Torino, Italy. [19]Department of Public Health and Pediatric Sciences, University of Torino, Torino, Italy

### Abstract

### Objective:

Differentiating hypocalcemic (HC) from hypophosphatemic (HP) rickets at presentation is clinically and prognostically relevant but often challenging due to overlapping manifestations. We aimed to characterize demographic, clinical, and biochemical features of pediatric rickets across Italy and identify distinguishing features between HC and HP forms at diagnosis.

### Methods:

We conducted a retrospective multicenter cohort study including 180 children and adolescents diagnosed with rickets in 14 Italian pediatric endocrinology centers. Patients were classified as HC or HP based on biochemical criteria. HC-rickets were further categorized as nutritional (n=85), genetic (n=5), or secondary (n=5). Clinical, anthropometric, and biochemical parameters were compared between groups and stratified by sex and age (<2, 2–10, >10years).

### Results:

A total of 95 HC (52.8%) and 85 HP (47.2%) patients were included (95 females, 85 males); 78/85 HP patients carried a *PHEX* mutation. A different sex distribution was observed (female-to-male ratio 0.82:1 in HC, p=NS vs 1.5:1 in HP, p=0.03). Median age at diagnosis was similar (2.2 vs 2.4 years), but distribution differed (p=0.002), with HC-rickets more frequently diagnosed before 2 years (49.5%) and HP-rickets between 2–10years (58.8%). Females with HC nutritional rickets were diagnosed later than males (median 3.9 vs 1.7years, p=0.004).

Intermediate and dark phototypes were prevalent in HC, whereas fair phototype in HP (p<0.0001). HP-rickets showed a higher prevalence of skeletal deformities (84.4% vs 61.7%, p=0.001), craniosynostosis (p=0.04), and short stature (35.1% vs 23.5%), with height impairment greater across all age groups (p=0.005). In HC-rickets varus deformities were more common (70.1% vs 33%, p<0.0001), while seizures and tetany occurred in 10.8% and 9.7%, respectively (p<0.01) (Figure1). HC-rickets were characterized by hypocalcemia and elevated PTH (median 295.2 pg/mL) and ALP (900 U/L) values, whereas HP-rickets showed hypophosphatemia with lower PTH (50.0 pg/mL, p<0.0001) and ALP (657 U/L, p=0.0004); 25(OH)D <50 nmol/L was observed in 28.2% of HP patients.

**Conclusions:**

HC and HP rickets exhibit distinct patterns in age at diagnosis, sex and phototype distribution, clinical and biochemical presentation. The later diagnosis and greater height deficit in HP-rickets highlight the need for improved early recognition and provide actionable clues for differential diagnosis and optimized management.

### Disclosure

None declared

### LB20

### Early and Sustained Vosoritide Treatment Is Associated with Favorable Body Composition Trajectories in Children with Achondroplasia

Klaus Mohnike[1], Julie Hoover-Fong[2], Andrew Dauber[3], Ravi Savarirayan[4], Melita Irving[5], Christine Rivat[6], Sue Lawrinson[6], Alice Huntsman Labed[6], Ian Sabir[6]

[1]University Hospital Magdeburg, Pediatrics, Hamburg, Germany. [2]Johns Hopkins University, Baltimore, USA. [3]Children's National Hospital, Washington, DC, USA. [4]Murdoch Children's Research Institute, Royal Children's Hospital, and University of Melbourne, Parkville, Victoria, Australia. [5]Guy's and St. Thomas' NHS Foundation Trust, Evelina Children's Hospital, London, UK. [6]BioMarin (UK) Ltd, London, UK

### Abstract

### Objectives:

Achondroplasia is a skeletal dysplasia characterized by impaired endochondral bone growth associated with multisystem alterations in physical development and body composition. Vosoritide, a CNP analog, inhibits overactive FGFR3, stimulating endochondral bone growth to provide sustained, durable improvements in growth and body proportionality for children with achondroplasia. We investigated effects of long-term vosoritide treatment on body composition.

### Methods:

Children with achondroplasia who initiated vosoritide aged 5 to <18 years (y) from the phase 3, double-blind, randomized, placebo-controlled trial CANOPY ACH-3 (111-301; NCT03197766) and its ongoing open-label extension CANOPY ACH-EXT (111-302; NCT03424018) were analyzed. Body composition was measured using dual X-ray absorptiometry of whole-body-less-head (WBLH), including regional fat and lean mass, to quantify fat-to-lean-mass ratio. Android-to-gynoid-fat-mass ratio, height, and weight were measured. ANCOVA models comparing vosoritide vs placebo were performed at week (W)52. Changes from baseline were assessed longitudinally over 7y.

### Results:

Among 119 participants randomized to vosoritide (n=58) or placebo (n=61) mean±standard deviation (SD) age was 9.2±2.6y and 52.9% were male. At data cutoff (25Aug2025), mean±SD treatment duration was 5.5±1.6y. WBLH lean mass increased in vosoritide (n=47) vs placebo (n=52) at W52 (least-squares mean [LSM] difference, 355.66g; 95% confidence interval [CI]: 38.23, 673.09; *P*=0.0285). In adolescents (≥11 to <15y), vosoritide-treated (n=8) vs placebo (n=11) had less fat mass increase (LSM difference, –778.04g; 95% CI: –2373.08, 817.00), increased lean mass (LSM difference, 1482.36g; 95% CI: –57.30, 3022.03), and the largest decrease in WBLH fat-to-lean-mass ratio (LSM difference, –0.076; 95% CI: –0.165, 0.012). Reduced fat-to-lean-mass ratio in this age group was most pronounced at 4y (n=17; mean±SD, –0.072±0.148) and continued in 5y (n=5; mean±SD, –0.153±0.0243). In all participants, android-to-gynoid-fat-mass ratio remained stable; at 6y, reduced fat-to-lean-mass ratio positively correlated with change in body mass index (**Table**).

### Conclusions:

Lean mass increased vs placebo in children initiating vosoritide aged ≥5y. Long-term, fat-to-lean-mass ratio stabilized or decreased for children aged ≥11 to <15y, a period when this ratio increases in untreated children. Given small sample sizes, sex-specific effects could not be evaluated. Sustained vosoritide treatment may positively impact body composition; however, more research is needed on body composition and impacts on achondroplasia-associated health outcomes.

**Table.** Impact of vosoritide on body composition

| | |
|---|---|
| **Overall** | |
| Treatment duration, mean ± SD (range), years | N = 119<br>5.5 ± 1.6<br>(1.7 to 7.8) |
| Change from baseline in WBLH lean mass (g) at week 52, LSM difference vs placebo (95% CI) | n = 47 vosoritide, 52 placebo<br>355.66 (38.23, 673.09)<br>*P* = 0.0285 |
| Correlation between change from baseline in WBLH fat-to-lean-mass ratio and BMI (kg/m$^2$) at year 6, Pearson r | |
| BMI | 0.489<br>*P* = 0.0009 |
| BMI Z-score | 0.429<br>*P* = 0.0041 |
| Android-to-gynoid-fat-mass ratio change from baseline, mean ± SD | |
| At year 7 | n = 17<br>0.054 (0.087) |
| **≥11 to <15 years** | |
| Change from baseline in WBLH fat mass (g) at week 52, LSM difference vs placebo (95%CI) | n = 8 vosoritide, 11 placebo<br>–778.04 (–2373.08, 817.00)<br>*P* = 0.3060 |
| Change from baseline in WBLH lean mass (g) at week 52, LSM difference vs placebo (95%CI) | n = 8 vosoritide, 11 placebo<br>1482.36 (–57.30, 3022.03)<br>*P* = 0.0577 |
| Change from baseline in WBLH fat-to-lean-mass ratio at week 52, LSM difference vs placebo (95%CI) | n = 8 vosoritide, 11 placebo<br>–0.076 (–0.165, 0.012)<br>*P* = 0.0852 |
| WBLH fat-to-lean-mass ratio change from baseline, mean ± SD | |
| At year 4 | n = 17<br>–0.072 ± 0.148 |
| At year 5 | n = 5<br>–0.153 ± 0.243 |

Statistical testing was descriptive with no control for type I error. Differences are calculated as vosoritide minus placebo.

BMI, body mass index; CI, confidence interval; LSM, least-squares mean; SD, standard deviation, WBLH, whole body less head.

### Disclosure

Funding was provided by BioMarin Pharmaceutical Inc. KM has served as a BioMarin Pharmaceutical Inc. advisory board member; has received personal fees from BioMarin Pharmaceutical Inc.; has received consulting fees and grants from Ascendis, BioMarin Pharmaceutical Inc., Novo Nordisk, and QED Therapeutics; and was an investigator in BioMarin Pharmaceutical Inc. clinical trials. JH-F has participated in advisory boards and served as a consultant for Ascendis, AstraZeneca, BioMarin Pharmaceutical Inc., Children's Mercy Research Institute, Medscape, Pfizer, QED Therapeutics, and Therachon and has received research support from AstraZeneca, BioMarin Pharmaceutical Inc., and QED Therapeutics. AD has served as a consultant for BioMarin Pharmaceutical Inc., but all compensation was paid to Children's National Hospital, and has received an investigator-initiated grant from BioMarin Pharmaceutical Inc. RS has received consulting fees and travel support from Ascendis, BioMarin Pharmaceutical Inc., and QED Therapeutics and has participated as a clinical trial investigator for Ascendis, BioMarin Pharmaceutical Inc., QED Therapeutics, and Sanofi. MI has received consulting fees from Ascendis, BioMarin Pharmaceutical Inc., QED Therapeutics/BridgeBio, Sanofi, and Tyra; speaker fees from Ascendis, BioMarin Pharmaceutical Inc., Ipsen, QED Therapeutics/BridgeBio, and Sandoz; travel support from Ascendis, BioMarin Pharmaceutical Inc., and QED Therapeutics/BridgeBio; and has participated as a clinical trial investigator for Ascendis, BioMarin Pharmaceutical Inc., and QED Therapeutics/BridgeBio. IS, AHL, and CR are employees and shareholders of BioMarin (UK) Ltd.

### LB21

### Lean Mass Deficit and Functional Impairment Despite Preserved Physical Activity in Children with X-Linked Hypophosphatemia: A Case-Control Study

Guido de Paula Colares Neto, Pedro Henrique Nunes Leite, Milena Santos Aguiar, Ana Catarina Lunz Macedo, Marina Mattiello Gabriele, Hamilton Roschel, Bruno Guaiano, Mariana Camargo de Sousa, Hamilton Cabral de Menezes Filho, Louise Cominato, Ruth Rocha Franco, Juliana Morgado, Durval Damiani

University of São Paulo, São Paulo, Brazil

### Abstract

### Objective:

To evaluate body composition, functional capacity, and habitual physical activity in children and adolescents with XLH compared with healthy age-, sex-, and pubertal stage-matched controls.

### Methods:

This cross-sectional case-control study included 14 XLH patients (10F/4M; mean age 11.15 +/- 3.21 years) and 14 age-, sex-, and pubertal status-matched controls. All participants were followed at the pediatric endocrinology outpatient clinic. Body composition was assessed by dual-energy X-ray absorptiometry (DXA; Hologic Horizon Wi). Functional capacity was evaluated using the Timed-Stands (TS) and Timed Up-and-Go (TUG) tests. Habitual physical activity was assessed by triaxial accelerometry (ActivPAL). Statistical analysis was performed in SPSS v27.0. A p-value $< 0.05$ was considered significant.

### Results:

Accelerometry demonstrated no significant between-group differences in step count (8,412 vs. 8,067 steps/day; $p = 0.980$), MVPA (1.91 vs. 1.75 min/day; $p = 0.184$), standing time (212.8 vs. 181.1 min/day; $p = 0.474$), or total sedentary time (546.2 vs. 611.1 min/day; $p = 0.069$). In contrast, functional performance was significantly impaired in XLH. TUG times were longer (median 8.59 s [4.91-27.06] vs. 6.50 s [4.51-10.35]; $p = 0.045$). TS repetitions were lower (13.38 +/- 2.90 vs. 15.92 +/- 3.62; $p = 0.060$; $d = -0.774$). DXA revealed a borderline significant reduction in TBLH lean mass in XLH patients (17,421 +/- 5,023 g vs. 22,561 +/- 7,912 g; $p = 0.060$; $d = -0.776$). The effect sizes were consistent with those recorded for the TS test. Fat mass variables did not differ significantly: total body fat (36.9 +/- 8.6% vs. 34.3 +/- 5.8%; $p = 0.383$), android/gynoid ratio (0.75 +/- 0.10 vs. 0.78 +/- 0.14; $p = 0.464$), and visceral adipose tissue mass (198.5 +/- 98.0 g vs. 189.0 +/- 90.9 g; $p = 0.802$). Lumbar spine L1-L4 Z-scores were significantly higher in XLH (median +1.80 [0.0-4.6] vs. 0.00 [-0.5 to 1.7]; $p = 0.001$; $r = 0.878$), consistent with disease-related bone remodeling.

### Conclusion:

Children with XLH demonstrate impaired functional capacity and reduced lean mass despite preserved physical activity levels, suggesting that intrinsic muscle impairment, rather than inactivity, is the primary driver of functional limitation.

### Disclosure

None declared

### LB22

### Appendicular Mechanical Loading Adaptations from Childhood to Peak Bone Mass: Menarche-Centered Musculoskeletal Growth Curves in Gymnasts vs. Non-Gymnasts

Jodi N Dowthwaite[1,2], Anna D Nicotra[1], Tim J Cole[3], Laure Spake[4], Tamara A Scerpella[1,2]

[1]SUNY Upstate Medical University, Syracuse, NY, USA. [2]University of Wisconsin-Madison, Madison, WI, USA. [3]University College London, London, UK. [4]Binghamton University, Binghamton, NY, USA

### Abstract

**OBJECTIVES:**

Gymnastics involves extreme upper extremity weight-bearing and impact-loading, unlike other sports. We evaluated functional muscle-bone unit development from childhood to peak bone mass (PBM), using menarche-centered growth curves for non-dominant arm and left leg appendicular muscle mass and bone mineral content (MM, BMC: g) in gymnasts (GYM) vs. non-gymnasts (NON). We hypothesized: 1) leg MM & BMC growth curves would be similar for GYM vs. NON; 2) arm MM & BMC growth curves would demonstrate higher size, earlier timing, and higher intensity of growth in GYM vs. NON.

**METHODS:**

Height (cm) was measured semi-annually for gymnasts (GYM: gymnastics ≥4.5h/wk, ≥1yr) and non-gymnasts (NON: others). Annual whole-body DXA scans yielded MM & BMC for arm and leg. SITAR (v1.5.0, R v 4.5.3) modeled menarche-centered growth curve size, timing, and intensity for height & MM, extracting individual random effects for use as parameter-matched covariates (size/timing/intensity). Menarche-centered growth curves for MM & BMC evaluated GYM/NON differences, after adjustment for height covariates (MM), or both height & MM covariates (BMC).

**RESULTS:**

Height growth curves indicated lower height size and intensity in GYM vs. NON ($p \leq 0.02$) but similar height timing ($p=0.6$). For leg and arm: height positively influenced MM & BMC growth patterns (size/timing/intensity: $p<0.001$), justifying adjustments. For leg: MM & BMC growth curves were similar for GYM vs. NON ($p>0.09$), except earlier GYM BMC timing (vs. NON, $p<0.002$). For arm: despite similar MM timing ($p>0.8$), GYM had earlier BMC timing, with higher GYM size and intensity for both MM & BMC (vs. NON, $p<0.007$).

**CONCLUSIONS:**

For the leg, GYM and NON had similar growth curves with “coupled” growth patterns for height, MM, and BMC. Gymnastic loading was linked to earlier BMC growth spurts for both leg and arm. Furthermore, for the non-dominant arm, steeper GYM peak growth velocity for MM & BMC yielded higher GYM arm MM & BMC at PBM, contrasting with lower NON arm BMC accrual that lagged after menarche, >1yr after peak velocity for height and MM. Thus, extreme weight-bearing impact-loading via youth gymnastics was linked to persistent, substantial arm BMC advantages, independent of disproportionately high gymnast muscle growth for height.

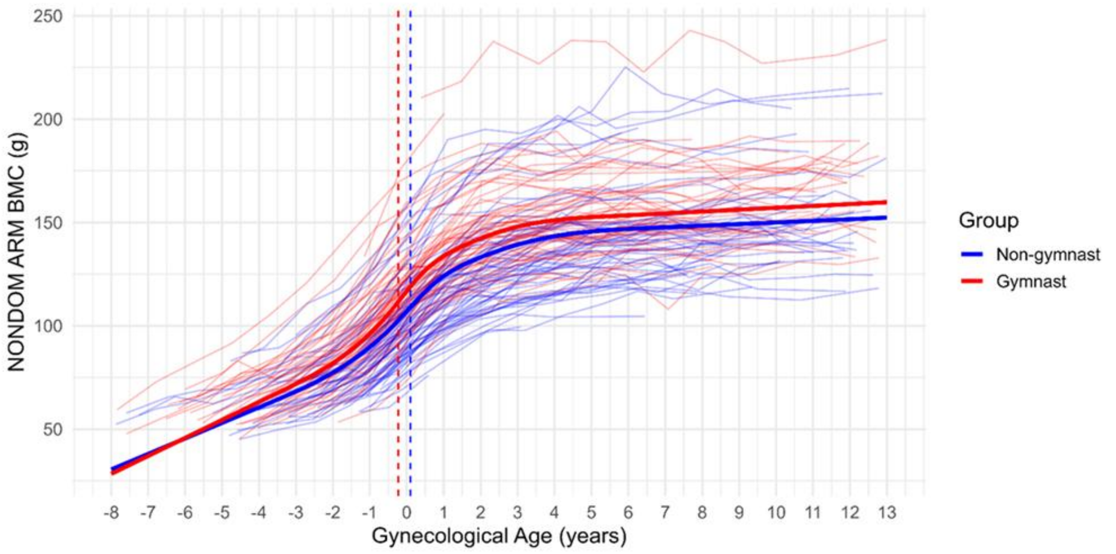

**Menarche-Centered Non-Dominant Arm BMC Growth Curves for Gymnasts vs. Non-Gymnasts, Adjusted for Size, Timing, and Intensity of Individual Growth in Height and Arm Muscle Mass**

GYM (n=67; 688 observations) vs. NON (n=69; 704 observations): Bold, solid lines depict group mean curves (df=5), while vertical lines depict group-specific timing of peak velocity for BMC accrual. SITAR curves for adjusted BMC differ in size, timing, and intensity [GYM vs. NON: $p<0.007$]. Notably, for GYM and NON, timing of peak growth velocities for height and muscle mass occurred roughly 1yr before menarche (gynecological age circa -1yr; data not shown).

### Disclosure

None declared.

### LB23

### What are the Physical and Psychosocial Needs of Individuals with Escobar Syndrome: A Scoping Review

Nithikaa Shashikanth[1,2], Naomi Zukerman[1,2], Andrea N Fabregas Roman[3], Neda Naghdi[2], Noemi Dahan-Oliel[2,1]

[1]McGill University, Montreal, Canada. [2]Shriner's Childrens Hospital, Montreal, Canada. [3]Universidad Central del Caribe, Bayamón, Puerto Rico

### Abstract

### Objectives:

Escobar syndrome, the nonlethal form of multiple pterygium syndrome (MPS), is a rare congenital genetic disorder first described in 1902. Its estimated global prevalence is fewer than 1 per 1,000,000 individuals. Key clinical features include growth restriction, short stature, joint contractures, and digital anomalies such as syndactyly and camptodactyly. Additional manifestations may include spinal deformities, cervical vertebral fusion, and foot abnormalities like congenital vertical talus. This study aimed to compile and synthesize existing evidence on the physical and psychosocial health of individuals with Escobar syndrome across the lifespan, using the International Classification of Functioning, Disability and Health (ICF) as a guiding framework.

### Methods:

A comprehensive search of five databases was conducted by an information specialist. Screening was performed independently by pairs of reviewers at the title/abstract and full-text levels. Studies were included if they examined physical or psychosocial health in children or adults with Escobar syndrome. Relevant data on assessments, interventions, and treatments were extracted, analyzed descriptively, and summarized narratively using Covidence, then mapped to the ICF framework. Findings were also discussed with interest holders, including a caregiver of two youth with Escobar syndrome and an adult living with the condition.

### Results:

A total of 46 studies met the inclusion criteria, with an inter-reviewer agreement rate of 81.3% during the pilot phase. Nearly half of these studies did not examine the psychosocial aspects of individuals with Escobar syndrome. Among those that did, the primary focus remained on physical characteristics, with only minimal—often single-sentence—mentions of the mental or psychosocial factors. The studies that addressed mental aspects focused primarily on intellectual and cognitive functioning, without examining the individuals’ quality of life. Interest holder consultations also highlighted key gaps, particularly in mental health (e.g., depression, anxiety, and body image), activity and participation (e.g., grocery shopping and employment), and environmental (e.g., societal attitudes) and personal factors (e.g., gender, motivation).

### Conclusion:

Escobar syndrome remains a rare condition with limited research, especially regarding psychosocial health. While current literature emphasizes physical outcomes, early diagnosis, and rehabilitation, there is a clear need for more comprehensive exploration of psychosocial dimensions to improve overall quality of life.

### Disclosure

None declared

### LB24

### Co-designing a Social Robot for Children in Orthopaedics

Renaud Lamonde[1], Juan Carlos Sabillon[1], Zachary Laramée[1], Amélie Achard[1], Félix Migué[1], Janin Roy[1], Argerie Tsimicalis[2,3], Hatice Kose[2,3]

[1]École de technologie supérieure (ÉTS), Montreal, Canada. [2]Shriners Hospitals for Children Canada, Montreal, Canada. [3]McGill University, Montreal, Canada

### Abstract

**Abstract**

**Objectives:**

This paper details the co-design and development of a social robot created to support children at Shriners Hospitals for Children Canada who are undergoing treatment for various orthopaedic, muscular, and neuromuscular conditions. Social robots are increasingly recognized for their ability to provide emotional support and distraction during distressing medical encounters (Triantafyllidis, et. al, 2023).

**Methods:**

To develop this tool, a multidisciplinary team of researchers from Electrical Engineering, Computer Science, and Nursing, supervised engineering students on a student project. The process involved bi-weekly meetings, site visits to the medical day center and casting room, and interviews with nursing and child life specialists to ensure the robot would be effective for distraction during procedures. The final design was based on ongoing discussions with robotics and nursing researchers, as well as feedback from clinicians and child consultants.

**Results:**

The resulting robot features an animated face, movable arms, static legs, a body-mounted screen, and a projector on its head (Figure 1). It is situated on a mobile, adjustable overbed table to accommodate children in hospital beds or wheelchairs while remaining easy to move within the hospital workflow. The arms and head are capable of simple gestures and dances coordinated with facial expressions and the projector to distract children during painful tasks like IV placement or cast removal. Additionally, the screen informs children about their medical procedures. For ease of use, two buttons beneath the screen control the system’s functions: a green button to start and a red button to stop actions such as singing, dancing and video playback. All songs and information are provided by hospital staff from established clinical sources. To create a superhero appearance and a sense of familiarity, the robot wears a cape and a "B" sign (representing "Bone") to resemble the mascot Captain Strongbone. The design remains simple to prioritize safety and usability.

**Conclusions:**

A social robot was co-designed and developed over a four-month period, and will be subject to testing and evaluation, as another non-pharmacological tool to children undergoing medical procedures. A demonstration will be available at the Shriners Hospitals for Children Canada booth.

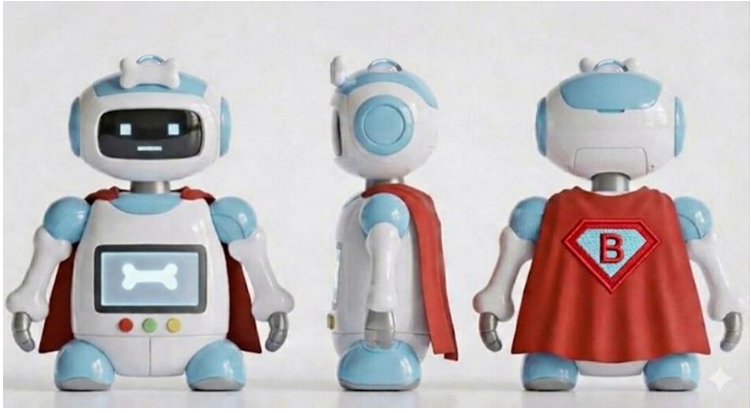

Figure 1. The social robot

References:

Andreas Triantafyllidis, Anastasios Alexiadis, Konstantinos Votis, Dimitrios Tzovaras, Social robot interventions for child healthcare: A systematic review of the literature, Computer Methods and Programs in Biomedicine Update, Volume 3, pp:100-108, 2023

### Disclosure

None declared

### LB25

### La Guérison par L’Art / Healing through Art

John Grow[1], Suzanne Charlebois[1], Hatice Kose[2,3], Helen Chrenowski[2], Marie-Annie Lagace[2], Chad Polito[2], Angie Gugliotti[2], Sylvain Rondeau[2], Kelly Thorstad[2], Julianna Marulanda[2], Eshwyn Makkar[3], Argerie Tsimicalis[2,3]

[1]Galerie d'Art Émergence, Montreal, Canada. [2]Shriners Hospitals for Children Canada, Montreal, Canada. [3]McGill University, Montreal, Canada

### Abstract

**Objectives:**

Historically, Shriners Hospitals for Children Canada (Shriners) has embraced art for healing and wellness. Aesthetics knowledge is displayed in multiple facets at the hospitals. Children use art to express their feelings, surgeons draw surgical procedures to facilitate assent, nurses sing during a medical procedure, artists render 3-D images of bones, researchers use puppets to facilitate data collection, and children offer moving dance performances for fundraising events. The objective is to publicly display the art of the Shriners community in collaboration with the Galerie d'Art Émergence; a non-for-profit, dedicated to showcasing Quebec's emerging artists.

**Methods**:

A Task Force has been assembled at the Shriners Hospitals for Children Canada to showcase the art of the Shriners community. Members include representation from clinical practice, research, fundraising, committee on arts & memorabilia, and the museum. Bi-weekly meetings are underway, planning for this arts-based knowledge mobilization event. Artwork will be analyzed, interpreted and synthesised to inform the theme, installation and layout of the exhibit.

**Results:**

The art exhibit will be on display at Galerie d'Art Émergence (primary location), from June 24 to July 2, 2026, with off-site viewings to be displayed at the Shriners Hospitals for Children Canada. Submissions to date include children’s artwork, primarily drawings and paintings, 3D printed models of bone, clay sculptures and animations.

**Conclusions:**

Art is crucial in improving patient outcomes through a holistic (wrap-around) and cost-effective approach to care that extends beyond simply the physical health of patients. It serves as a valuable complementary and evidence-based strategy supporting all aspects of a patient’s mental, emotional, social and physical well-being by helping to reduce stress, anxiety, pain and contribute to faster recovery times.

### Disclosure

None declared

### LB26

### Maturity-Specific Models of Youth Physical Activity Exposure: Height- and Weight-Adjusted Bone Accrual to Peak Bone Mass

Jodi N Dowthwaite[1,2], Anna D Nicotra[1], Tim J Cole[3], Stephanie Kliethermes[2], Tamara A Scerpella[1,2]

[1]SUNY Upstate Medical University, Syracuse, NY, USA. [2]University of Wisconsin-Madison, Madison, WI, USA. [3]University College London, London, UK

### Abstract

**OBJECTIVES:**

Youth physical activity (PA) influences peak bone mass (PBM), but maturity-specific loading responses across time are unclear. Bone mineral content (BMC) peak accrual velocity (${}_{PK}V_{BMC}$) is centered on menarche, plateauing to PBM 4-6yrs post-menarche. We evaluated cumulative PA ($PA_{CUM}$) patterns and gymnastic loading TYPE as factors in menarche-centered growth curves for whole-body ($BMC_{WB}$) and non-dominant arm ($BMC_{ARM}$).

**METHODS:**

From our longitudinal study, we included participants with menarche-centered data spanning pre-${}_{PK}V_{BMC}$ to PBM: $BMC_{WB}$ & $BMC_{ARM}$ (g), height (cm), weight (kg), and non-aquatic PA (month-to-month, hrs/wk) for $PA_{CUM}$ calculation (hrs). TYPE contrasted gymnastics exposure groups vs. NON (no gymnastics): $HI_{GYM}$ (≥14.5hrs/wk) & $MED_{GYM}$ (≥4.5 hrs/wk) continued training pre-menarche to post-menarche; $EX_{GYM}$ (≥4.5hrs/wk) quit by 3mos post-menarche; $LO_{GYM}$ (<4.5h/wk) trained at low levels, mainly pre-menarche. We used SITAR (v1.5.0, R v4.5.3) to model menarche-centered growth curves for $PA_{CUM}$, height, and weight to extract individual random effects for SITAR parameters (size/timing/intensity) for use as covariates. Menarche-centered BMC curves evaluated $PA_{CUM}$ profiles and TYPE as factors in size, timing, and intensity of $BMC_{WB}$ & $BMC_{ARM}$ accrual to PBM, after height and weight adjustment (SITAR covariates: TYPE and parameter-matched random effects).

**RESULTS:**

BMC curves were adjusted for positive height and weight influence ($p<0.001$: size/timing/intensity). For $BMC_{WB}$, $PA_{CUM}$ was positively associated with $BMC_{WB}$ size, timing, and intensity ($p<0.001$). Few $BMC_{WB}$ differences were observed by TYPE: $MED_{GYM}$ had earlier $BMC_{WB}$-timing, and $EX_{GYM}$ /$LO_{GYM}$ had lower $BMC_{WB}$-intensity (vs. NON, $p<0.03$). For $BMC_{ARM}$, loading TYPE was most important, as $PA_{CUM}$ only positively influenced $BMC_{ARM}$-timing ($p<0.01$): $HI_{GYM}$/$MED_{GYM}$ had higher $BMC_{ARM}$-size & $BMC_{ARM}$-intensity, while $EX_{GYM}$ and $MED_{GYM}$/$HI_{GYM}$ had earlier $BMC_{ARM}$-timing (vs. NON, $p<0.05$) [see Figure 1].

**CONCLUSIONS:**

After adjustment for menarche-centered height and weight growth profiles, timing of peak intensity of non-aquatic physical activity exposure was a positive factor in ${}_{PK}V_{BMC}$ timing. Averaged across the whole-body, $PA_{CUM}$ dose, timing, and intensity were more potent positive predictors of $BMC_{WB}$ accrual to PBM than gymnastic loading type. For the non-dominant arm, $PA_{CUM}$ timing remained influential, but advantages in $BMC_{ARM}$ PBM required MED-HI level gymnastic training, sustained over the menarche-centered ${}_{PK}V_{BMC}$ window, ensuring arm-specific, high-impact loading after menarche.

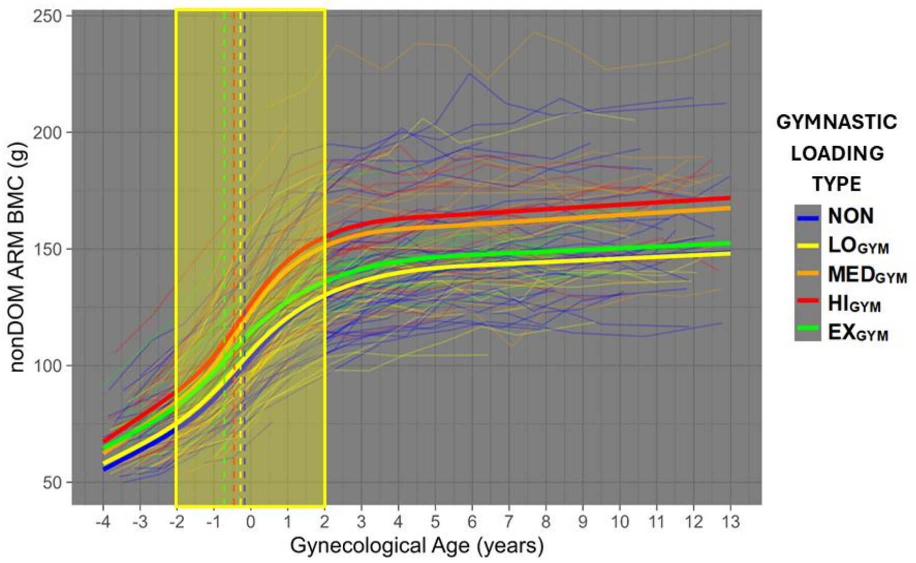

**FIGURE 1:** **Menarche-Centered Non-Dominant Arm Bone Mineral Content, Adjusted for Height, Weight, and Cumulative Non-Aquatic Physical Activity Exposure ($PA_{CUM}$)**

[n=126 (Non-gymnasts: NON=31; Gymnasts: $LO_{GYM}$=26; $MED_{GYM}$=24; $HI_{GYM}$=26; $EX_{GYM}$=19); scans=1251; df=4; AIC=8343]

Bold lines show group-specific mean curves. Vertical dotted lines show group mean timing of $_{PK}V_{BMC}$ within the high $V_{BMC}$ window (yellow box). $MED_{GYM}$ & $HI_{GYM}$ had higher growth size & intensity; $EX_{GYM}$ & $MED_{GYM}$/$HI_{GYM}$ (red) had earlier mean timing of $_{PK}V_{BMC}$ (vs. NON, $p<0.05$).

### Disclosure

None declared

### LB27

### Radiographic Evaluation of Ulnar Variance from Childhood to Adulthood in Female Gymnasts Versus Non-gymnasts

Irina D Sokolik[1], Alex Rodriguez[1], Angelique Dabel[1], Dante Whitmire[1], Austin Zeplowitz[1], Carson P Hobler[1], Jackson Bright[1], Tracy Eng[1], Anna D Nicotra[1], Tamara A Scerpella[1,2], Jodi N Dowthwaite[1,2]

[1]SUNY Upstate Medical University, Syracuse, NY, USA. [2]University of Wisconsin-Madison, Madison, WI, USA

### Abstract

**PURPOSE:**

“Normal” wrists have negative ulnar variance (radius distal to ulna); positive variance denotes ulna growth past the radius. In Acquired Madelung’s Deformity (AMD, or "gymnast’s wrist”), youths with open growth plates repeat high-intensity wrist-loading; premature radial physis closure and positive ulnar variance (>2mm) may have long-term complications. We assessed ulnar variance across childhood, adolescence, and early adulthood in females subjected to repetitive high-impact distal radius stresses during growth (gymnasts, GYM) vs. non-gymnasts (NON). We hypothesized that GYM would have higher ulnar variance than NON.

**METHODS**:

Our team collected annual DXA scans of the distal non-dominant forearm in female GYM (≥1-year, annual mean training ≥6hrs/wk) & NON (others). Multiple observers used Fiji to measure ulnar variance and assess skeletal maturation using the Distal Radius and Ulna rating system (Luk DRU). We used a general linear model (SPSS) to compare median ulnar variances (UV, mm) in GYM vs. NON, using a model random effect to link repeated UV measurements within-individual, accounting for timing relative to menarche (gynecological age, yrs: $GYN_{AGE}$) at each scan date.

**RESULTS:**

NON (n=37, 141 scans) and GYM (n=24, 194 scans) yielded 335 scans, representing pre-puberty to adulthood. In GYM, grand annual mean gymnastic training was 8.3 h/wk (sd 7.0). Radius DRU ratings ranged from early epiphyseal maturation to full fusion (DRU: R5 to R11). Accounting for individual effects and interaction of $GYN_{AGE}$*GYM status ($p<0.001$), group mean UV was higher for GYM than NON ($p<0.04$; estimated marginal means at 1.3yrs post-menarche: GYM= -0.05mm; NON= -1.41mm) [see Figure]. Nonetheless, few GYM had UV >2mm (GYM=4, 12 scans; NON=8, 23 scans).

**CONCLUSION:**

Although GYM means for UV were higher than NON, few GYM were positive for UV>2mm in this cohort comprised of GYM and NON with diverse physical activity histories. Future work should assess whether underlying soft tissue damage (e.g. triangular fibrocartilage complex tears) leads to chronic pain or dysfunction in GYM and NON without frank AMD. Our work advances the field with prospective longitudinal data for female GYM & NON recruited at a healthy baseline, exposed to varied loading across susceptible stages, and followed up to late post-menarche, circum-peak bone mass.

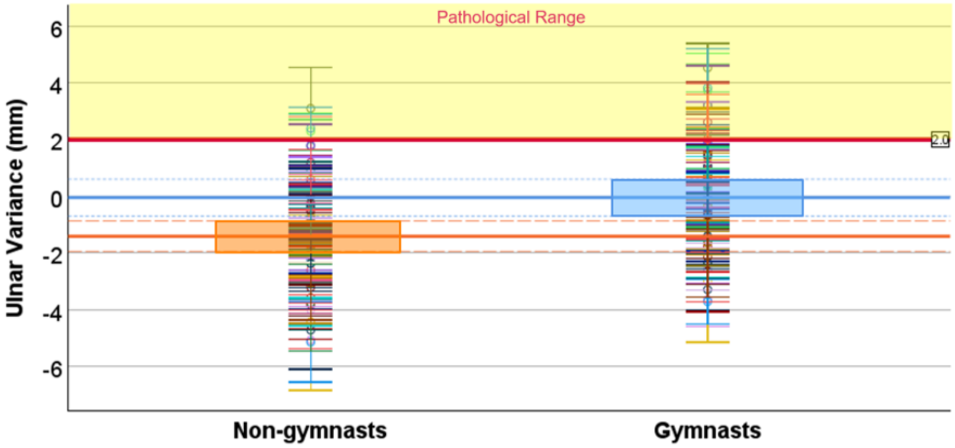

Covariates appearing in the model are evaluated at: Gynecological Age (yrs) = 1.34

Estimated Marginal Means with 95% CI

As, shown, gymnasts have a higher group estimated marginal mean (EMM) for repeated measurements of median ulnar variance than non-gymnasts. These EMMs account for individual changes in ulnar variance and gynecological age across skeletal maturation, from childhood into late post-menarche, circum-peak bone mass. Gymnasts' group EMM is shown by the bold blue line, with a blue box indicating the 95% confidence interval; non-gymnast values are depicted in orange. Within-group, individual data are plotted in diverse colors with error bars (95% confidence intervals for individual EMMs). "Normal" ulnar variance is negative, so the red line indicates the threshold for the pathological range of positive ulnar variance (+2 mm), beyond which individuals may be at greater risk of complications such as triangular fibrocartilage complex tears.

### Disclosure

None declared

### LB28

### Accelerometer-Measured Physical Activity in Relation to Bone Health Among Children and Adolescents in Rural India

Rubina Mandlik[1], Sharvani Patil[1], Aditi Vargante[1], Chidvilas More[1], Jasmin Bhawra[2], Tarun Katapally[3], Mark Tremblay[4], Richard Larouche[5], Mahdi Rostami Haji Abadi[5], Anuradha Khadilkar[1]

[1]Hirabai Cowasji Jehangir Medical Research Institute, Pune, India. [2]Toronto Metropolitan University, Toronto, Canada. [3]Western University, London, Canada. [4]CHEO Research Institute, Ottawa, Canada. [5]University of Lethbridge, Lethbridge, Canada

### Abstract

**Objective:**

Physical activity is essential for musculoskeletal health and development in children and adolescents. This study explored associations between accelerometer-measured physical activity and bone mass, area, and strength measured by Dual Energy X-ray Absorptiometry (DXA) and peripheral quantitative computed tomography (pQCT).

**Methods:**

This cross-sectional, observational study included 161 children and adolescents aged 8-13 years residing in villages located around Pune, Western India. Hip-worn accelerometers (ActiGraph wGT3X-BT) were used for 8 consecutive days (100 Hz sample rate, 5 sec epoch) to measure physical activity. The Freedson 1998 cut points were used to classify accelerometer data. Bone mass, area and strength were measured using DXA and pQCT. Total body less head, and radius and tibia were the preferred scan sites for DXA and pQCT, respectively.

**Results:**

Average time spent in light activity was 214.9 ± 39.0 and 200.7 ± 44.5 min/day and in moderate-to-vigorous physical activity (MVPA) was 54.8 ± 28.0 and 44.6 ± 23.9 min/day for boys and girls, respectively. Only 46% of boys and 18% of girls met WHO recommendations for sufficient physical activity (MVPA ≥60 min/day). Linear regression analysis showed that vigorous physical activity was associated with higher total body bone mineral density (TBBMD) in boys ($\beta = 2.2$, $p = 0.003$), after controlling for height and weight (Fig 1). In girls, after adjusting for radius length and weight, light physical activity was negatively associated with total area ($\beta = -0.2$, $p = 0.027$) and positively associated with total density ($\beta = 0.3$, $p = 0.015$) at radius shaft. Light activity was also negatively associated with trabecular area ($\beta = -0.1$, $p = 0.027$) at the distal radius. No significant associations were observed for tibial parameters.

**Conclusion:**

Rural Indian children, particularly girls, demonstrated low engagement in MVPA. Physical activity was significantly associated with bone parameters; notably, vigorous physical activity was positively associated with TBBMD in boys, while in girls, light physical activity exhibited mixed associations with bone parameters. These findings underscore the need for targeted interventions to promote higher intensity physical activity among children during growth to support optimal bone development and reduce future risk of compromised bone health.

**Figure 1: Association of physical activity parameters with bone outcomes**

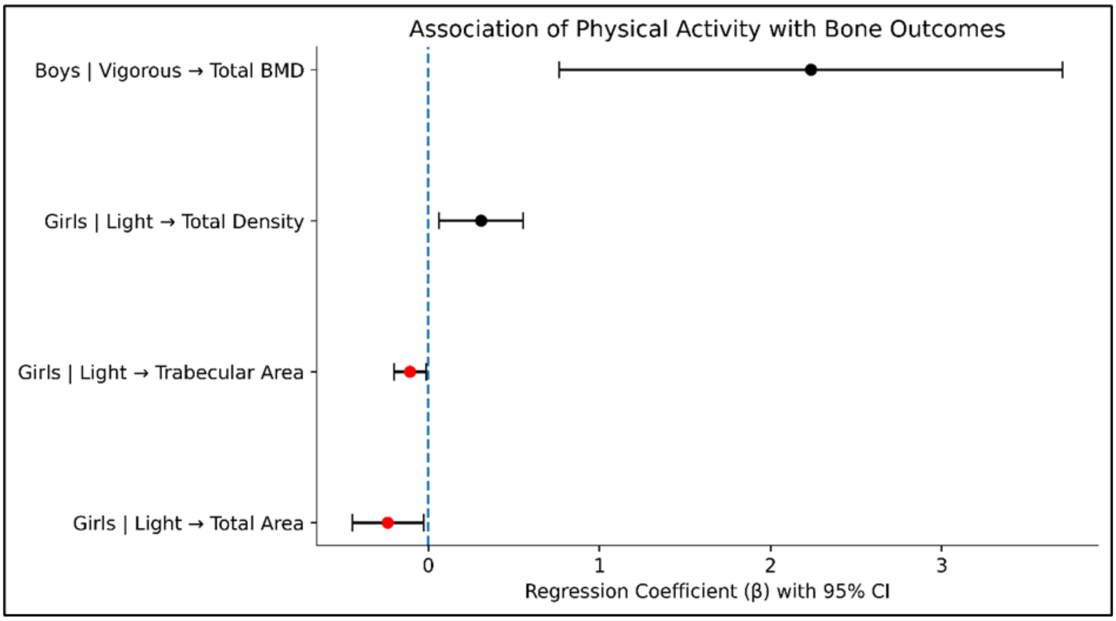

### Disclosure

None declared.

### LB29

### Interrelations of Adiposity, Vitamin D Status, and Bone Metabolism in Pediatric Low-Energy Trauma: A Case–Control Study

Andrea Cosentino[1,2], Daniel Weghuber[2], Wolfgang Högler[3]

[1]Dolomiti Sportclinic, St. Ulrich, Italy. [2]Paracelsus Medical University, Salzburg, Austria. [3]Johannes Kepler University, Linz, Austria

### Abstract

### Objectives:

Pediatric fractures are common, and both adiposity and vitamin D status have been implicated as potential contributors to fracture risk. This prospective study aimed to investigate the relative contributions to fracture risk of biomechanical factors associated with increased body mass and vitamin D-calcium metabolism in healthy children.

### Methods:

We conducted a case–control study including 100 children presenting with low-energy trauma (50 with radiographically confirmed fractures and 50 controls). Anthropometric measurements and biochemical markers of bone mineralisation—including serum 25OH vitamin D (25OHD), parathyroid hormone (PTH), alkaline phosphatase (ALP), calcium, and phosphate—were assessed within seven days of injury. Vitamin D status and dietary calcium intake were analysed using clinically relevant categories. Associations between adiposity and biochemical parameters were explored using correlation analyses and multivariable logistic regression models, including both continuous and tertile-based exposures.

### Results:

Among children with low-energy fractures, 37 fractures involved the upper extremity and 13 involved the lower extremity; in the fracture group, children had significantly higher BMI and BMI z-scores compared with controls. Vitamin D deficiency (25OHD $<30$ nmol/L) was uncommon, affecting only 3% of participants, and biochemical markers of mineralisation were largely within age-appropriate reference ranges. BMI was inversely correlated with 25OHD ($\rho = -0.254$, $p = 0.011$) and positively correlated with PTH ($\rho = 0.268$, $p = 0.007$). In multivariable logistic regression, BMI remained the strongest independent predictor of fractures. Children in the lowest 25OHD tertile had higher odds of fracture compared with those in the highest tertile, whereas calcium intake tertiles were not independently associated with fracture risk.

### Conclusions:

In this pediatric cohort, fracture risk was primarily associated with adiposity rather than vitamin D–calcium metabolism. The absence of biochemical evidence of impaired mineralisation supports a predominantly biomechanical explanation for low-energy fractures in children with increased body mass. These findings align with current consensus guidelines that do not recommend routine vitamin D screening in otherwise healthy children presenting with fractures.

### Disclosure

None declared

### LB30

### Maturity-Specific Influence of Polygenic Risk Score, Physical Activity Exposure, and Loading Type in Height- & Weight-Adjusted Bone Accrual Curves to Peak Bone Mass

Jodi N Dowthwaite[1,2], Anna D Nicotra[1], Tim J Cole[3], Stephanie A Kliethermes[2], Tamara A Scerpella[1,2]

[1]SUNY Upstate Medical University, Syracuse, NY, USA. [2]University of Wisconsin-Madison, Madison, WI, USA. [3]University College London, London, UK

### Abstract

**OBJECTIVES:**

In females, bone mineral content (BMC) peak growth velocity ($_{PK}V_{BMC}$) is centered on menarche, and BMC plateaus 4-6yrs post-menarche to yield peak bone mass (PBM). Limited evidence suggests that a polygenic risk score (gSOS) and youth physical activity (PA) independently predict pediatric bone properties, but it is unclear how gSOS and PA influence bone accrual to PBM. We evaluated gSOS, gymnastic loading TYPE, and cumulative PA exposure ($PA_{CUM}$) as maturity-specific factors in menarche-centered BMC growth curves for whole-body ($BMC_{WB}$) and non-dominant arm ($BMC_{ARM}$), accounting for height and weight growth profiles.

**METHODS:**

For inclusion in the current analyses, gSOS and gynecological age ($GYN_{AGE,}$ menarche-centered yrs) were required, with focal prospective, longitudinal data spanning roughly 2yrs pre-menarche to $\geq$4yrs post-menarche: PA (h/wk), height (cm), weight (kg), and annual DXA for $BMC_{WB}$ and $BMC_{ARM}$ (g). Cumulative PA dose ($PA_{CUM}$) was calculated for each participant. TYPE groups were: GYM (≥12mos gymnastics, mean ≥4.5h/wk, continued post-menarche), EX (≥12mos gymnastics, mean ≥4.5h/wk, quit by 3mos post-menarche), and NON (all others). We used SITAR (v1.5.0, R v4.5.3) to model menarche-centered growth curves for $PA_{CUM}$, height, and weight, extracting individual random effects for SITAR parameters (size/timing/intensity). After adjusting for height and weight, we used menarche-centered growth curves to evaluate influence of gSOS, $PA_{CUM}$ patterns, and loading TYPE on $BMC_{WB}$ and $BMC_{ARM}$ accrual to PBM.

**RESULTS:**

Height and weight profiles positively influenced BMC size, timing, and intensity ($BMC_{WB}$ & $BMC_{ARM}$: $p<0.002$), justifying adjustment. For $BMC_{WB}$ and $BMC_{ARM}$, gSOS positively influenced size and intensity ($p<0.02$), but not timing ($p>0.7$). For $BMC_{WB}$ and $BMC_{ARM}$, $PA_{CUM}$ positively influenced size and timing, but only positively influenced intensity for $BMC_{WB}$ ($p<0.05$). GYM had higher $BMC_{ARM}$ intensity, contrasting with lower EX intensity for $BMC_{WB}$ and $BMC_{ARM}$ (vs. NON, $p<0.03$).

**CONCLUSIONS:**

Adjusting for influences of height, weight, and gSOS, $PA_{CUM}$ was a positive factor in size, timing, and intensity of menarche-centered $BMC_{WB}$ accrual. Independent of height, weight, and gSOS, sustained circum-menarcheal gymnastic loading was linked to $BMC_{ARM}$ benefits, exceeding $PA_{CUM}$ influence for all but timing. Regardless of polygenic risk, mechanical loading across 4 menarche-centered years encompassing $_{PK}V_{BMC}$ was associated with higher PBM.

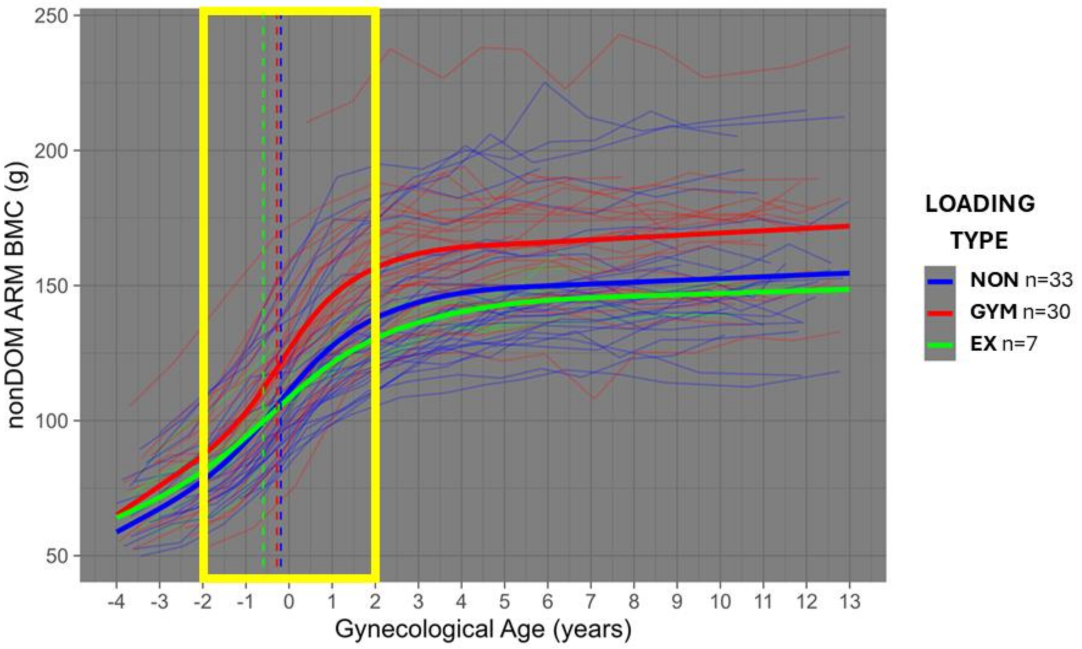

**FIGURE 1: Menarche-Centered Growth Curves for Non-Dominant Arm Bone Mineral Content**

Bold, solid lines depict group mean growth curves, while vertical dotted lines depict group-specific means for timing of $_{PK}V_{BMC}$ relative to menarche (gynecological age = 0). After adjusting for positive influence of height, weight, and gSOS, gymnasts (GYM) have higher intensity of arm BMC accrual velocity and size of BMC accrual to peak bone mass than non-gymnasts (vs. NON, $p<0.001$). In contrast, due to cessation of gymnastics by 3 months post-menarche, ex-gymnasts (EX) have lower intensity of arm BMC accrual (vs. NON, $p<0.01$). These findings highlight the importance of osteogenic loading sustained across 4-years of peak BMC accrual velocity centered at menarche (yellow box) to promote peak bone mass, regardless of genetic risk.

### Disclosure

None declared

### LB31

### Developing a Pediatric Digital Companion: A Three-Phase Co-Design and Quality Improvement Study of Children's Journey

Natalia Ayodele[1,2,3], Tina Athanasoulias[4], Kelly Thorstad[4], Argerie Tsimicalis[4,2,3], Manoja Chandralingam[1], Elena Guadagno[1], Dan Poenaru[1,2,3]

[1]Harvey E. Beardmore Division of Pediatric Surgery, The Montreal Children's Hospital, McGill University Health Centre, Montreal, Canada. [2]Faculty of Medicine and Health Sciences, McGill University, Montreal, Canada. [3]McGill University, Montreal, Canada. [4]Shriners Hospital for Children – Canada, Montreal, Canada

### Abstract

**Background:** Communication gaps contribute to medical errors, reduced quality of care, and avoidable complications in pediatric care. Children with unmet information needs experience heightened anxiety and reduced sense of control during hospital visits. While mobile health (mHealth) applications show perioperative benefit in addressing these challenges, a critical gap remains - pediatric-specific interventions co-designed with children are scarce. We developed Children's Journey, a digital platform currently serving caregivers, and launched a co-design initiative to develop a child-centered digital tool grounded in children's preferences. We aimed to engage pediatric surgical patients to inform a digital companion by examining their interest in a health application, preferred features, and responses to Children's Journey mock-up screens.

**Methods:** In this three-phase cross-sectional quality improvement study, we recruited children (aged 8-17 years, n = 106) receiving surgical care at Shriners Hospitals for Children - Canada. Phase 1 (n = 26) assessed interest in mHealth applications. Phase 2 (n = 59) explored feature preferences and perceived care utility. Phase 3 (n = 21) evaluated responses to preliminary Children's Journey mock screens. Data were collected via surveys. Quantitative data were analyzed descriptively and open-ended responses categorized thematically.

**Results:** In Phase 1, most children had limited health app experience but were receptive; 69% of kids preferred video-based learning and 77% wanted peer stories. Adolescents (14-17 years) (76%) prioritized secure provider communication; younger children (8-13 years) (67%) sought peer connection. In Phase 2, 71% of respondents wanted clear perioperative information; 76% wanted pain management support; 66% wanted the ability to track health; 63% wanted secure provider messaging; peer stories (46%) and connection (12%) were less favoured. In Phase 3, 57% wanted health tracking; 48% wanted pain management; 57% valued peer stories; 24% wanted peer connection. Children consistently prioritised personalization, short engagement duration (5-10 minutes), with a visual media-based learning. Open-ended responses primarily offer additional healthcare context outside of app development and were not included.

**Conclusion:** Children undergoing surgery express consistent, cross-phase demand for a personalized, perioperative mHealth platform that supports information, engagement, and health management needs. These findings directly inform Children's Journey development and support its broader implementation across other pediatric clinical settings locally and across Canada.

### Disclosure

Dr. Dan Poenaru and Elena Guadagno are co-founders of SurgAI Inc. & PetitVR Inc.

### LB32

### Turtlebrace$^{TM}$ Immobilisation for Paediatric Lower Leg Fractures in Osteogenesis Imperfecta

Kerry Myatt[1], Liam Johnson[1,2], Diane Jensen[1,2]

[1]Queensland Children's Hospital, Brisbane, Australia. [2]University of Queensland, Brisbane, Australia

### Abstract

**BACKGROUND**

Osteogenesis imperfecta (OI) is a heterogeneous genetic disorder characterised by impaired bone quality, skeletal fragility, and a high lifetime fracture burden. Children with OI commonly sustain long bone fractures, which can significantly impact mobility and participation. Surgical management frequently involves intramedullary rodding, such as Fassier-Duval (FD) rods, and/or cast immobilisation, as well as deformity correction where required.

**PRESENTING PROBLEM**

Optimal management of fractures in OI favours short immobilisation periods (Nijhuis et al 2022), as clinically appropriate, to support healing. Prolonged immobilisation and non-weight bearing can contribute to exacerbation of muscle weakness, delayed functional recovery, and increase subsequent fracture risk, creating a clinical challenge in balancing fracture stability with early rehabilitation. There are many potential known complications of casting for long periods. Controlled Ankle Motion (CAM) walkers, or Moon Boots, are an alternative to casting, but can be cumbersome, particularly for paediatric patients.

**CLINICAL MANAGEMENT**

To reduce immobilisation-associated limitations, our centre utilised a hybrid orthosis (Turtlebrace$^{TM}$) for three male patients with OI aged between 8 and 10 years. Clinical indications included a tibial re-fracture with pre-existing FD rod in situ, a fibula fracture managed as an alternative to a traditional plaster backslab cast, with clearance for weight-bearing as tolerated, and pre- and post-operative (insertion of FD rod) immobilisation for a slow-healing tibial fracture as an alternative to a moon boot and traditional casting.

**DISCUSSION**

Use of the Turtlebrace provided effective immobilisation while allowing regular skin checks, scar management where applicable, improved hygiene, and early participation in water-based rehabilitation. The removable, re-mouldable design supported earlier functional rehabilitation, permitted weight-bearing where clinically appropriate, and facilitated gait retraining through compatibility with footwear. For children with OI, this approach may reduce the secondary complications of prolonged immobilisation and enhance patient tolerance. This case series suggests that hybrid orthotic immobilisation may be a valuable adjunct or alternative to casting in the management of selected lower leg fractures in the paediatric OI population.

*Reference:*

*Nijhuis, W., Verhoef, M., van Bergen, C., Weinans, H., & Sakkers, R. (2022). Fractures in Osteogenesis Imperfecta: Pathogenesis, Treatment, Rehabilitation*

### Disclosure

None declared

### LB33

### Endpoints Beyond Anthropometry in Therapeutic Studies for Children with Achondroplasia: A Scoping Review

Mei Wang[1], Nadia Guele[1], Damian Rauch[1], Roozbeh Pourziaei Manesh[1], Lynn MacLeay[1], Abrar Mohammed Abdul[1], Rana Halloun[1], Rodrigo Montero-Lopez[1], Nasrin Khan[1], Stefan Jackowski[1], Maya Sharke[1], Leanne Ward[2,1]

[1]Pediatric Bone Health Research Group, Children's Hospital of Eastern Ontario Research Institute, Ottawa, Canada. [2]Division of Endocrinology and Metabolism, Department of Pediatrics, Children's Hospital of Eastern Ontario, University of Ottawa, Ottawa, Canada

### Abstract

OBJECTIVES:

We conducted a scoping review of the literature on methods for quantifying functional endpoints beyond growth in studies of growth-promoting therapy in children with achondroplasia (ACH).

METHODS:

Medline, Embase, Web of Science Core Collection, and ClinicalTrials.gov databases and registries were systematically searched for English-language studies published up to Apr 8, 2026. Original studies and study protocols investigating therapeutic interventions in children with ACH were deemed eligible. Data were narratively synthesized using a descriptive–analytical framework, emphasizing efficacy endpoints beyond growth-related outcomes (i.e., apart from stature, segmental growth, and body proportionality).

RESULTS:

A total of 39 publications (31 study reports and 8 protocols) representing 33 distinct studies (17 single-arm and 16 comparative) were included in this review, encompassing 7,981 individuals with ACH aged <18 years. The interventions investigated included growth hormone (13/33), vosoritide (14/33), recifercept (3/33), navepegritide (2/33), meclizine (1/33), infigratinib (1/33), and growth hormone + vosoritide (1/33). Across 33 studies, clinical imaging endpoints were used in 27/33 studies (80.0%), including limb length and lower extremity alignment (13/33), spinal deformity and canal dimensions (11/33), and cranial outcomes (4/33). Imaging modalities included radiographs (25/33), magnetic resonance imaging (3/33), and computed tomography (2/33). Questionnaire-based outcomes were reported in 8/33 studies (24.2%), assessing health-related quality of life (HRQoL, 4/33), functional independence (2/33), and ACH-specific patient- and observer-reported outcomes (2/33). Functional assessments were collected in 3 of the 33 studies (9.1%), including the 6-minute walk test (1/33), jumping mechanography (1/33), and polysomnography (1/33). Recently, 3-dimensional facial surface imaging has emerged as an innovative method for quantifying changes in facial structure on growth-promoting therapy (1/33).

CONCLUSION:

While growth and body proportionality remain key endpoints in therapeutic trials for children with ACH, imaging endpoints and direct or questionnaire assessments of physical and respiratory function, independence, and HRQoL underscore the importance of other phenotypic features and their impact on a child's life. Standardizing endpoints that extend beyond growth and body proportionality to more fully capture the impact of key skeletal dysplasia features and their effects on daily living will be valuable for future research by clarifying their relationships with the growth response to innovative therapies.

### Disclosure

None declared.

### LB34

### Linear and Segmental Growth in Therapeutic Trials for Children with Achondroplasia: A Scoping Review

Nadia Guele[1], Mei Wang[1], Damian Rauch[1], Roozbeh Pourziaei Manesh[1], Lynn MacLeay[1], Abrar Mohammed Abdul[1], Rana Halloun[1], Rodrigo Montero Lopez[1], Nasrin Khan[1], Stefan Jackowski[1], Maya Sharke[1], Leanne Ward[2,1]

[1]Pediatric Bone Health Research Group, Children's Hospital of Eastern Ontario (CHEO) Research Institute, Ottawa, Canada. [2]Division of Endocrinology and Metabolism, Department of Pediatrics, Children's Hospital of Eastern Ontario, University of Ottawa, Ottawa, Canada

### Abstract

**OBJECTIVES**:

We conducted a scoping review of the literature on methods for quantifying growth and other anthropometric endpoints in therapeutic trials involving children with achondroplasia (ACH).

**METHODS**:

The Medline, Embase, Web of Science Core Collection, and ClinicalTrials.gov databases or registries were searched for studies published in English through March 16, 2026. Original trials or trial-related protocols were eligible if they targeted a growth-modulating therapy in children and adolescents ≤18 years of age with ACH. Data were synthesized narratively using a descriptive-analytical approach, focusing on linear and segmental growth plus body proportionality.

**RESULTS**:

A total of 36 peer-reviewed publications (28 study reports and 8 protocols) for 30 trials (17 single-arm and 13 comparative studies) were identified in this review, comprising 7,784 children with ACH. Therapeutic interventions included growth hormone (12/30 studies), vosoritide (10/30), recifercept (3/30), navepegritide (2/30), meclizine (1/30), infigratinib (1/30), and combined growth hormone + vosoritide (1/30). Growth outcomes were expressed as changes in height (8/30, 26.9%), height z-scores (23/30, 76.9%), growth velocity (GV) (22/30, 76.9%), GV z-scores (7/30, 26.9%), and proportion of patients with GV ≥ 6 cm/year (1/30, 3.3%). Across 30 studies, anthropometric measures were widely used, with emphasis on segmental growth and body proportions. The most frequently assessed measures included upper-to-lower segment ratio (9/30), sitting height-to-height ratio (8/30), and arm span (6/30). Additional measures included body mass index (6/30), weight (6/30), arm span-to-height ratio (5/30), head circumference (5/30), and sitting height (5/30). Less commonly, studies assessed specific segment lengths or ratios, including arm span–to-height differences (3/30) and lower limb-to-height ratios (3/30). The change in bone age relative to the change in chronological age was reported in 8/30 studies.

**CONCLUSIONS**:

Changes in height, growth velocity (also expressed as Z-scores), and assessments of segmental growth and body proportionality have been the focus of therapeutic trials in children with ACH to date, with most (but not all) reporting the pace of skeletal maturation. Identifying the minimal clinically important differences in growth parameters by evaluating functional endpoints and their relationships with the growth response is an important direction for future research in children receiving medical treatments for ACH.

### Disclosure

None declared

### LB35

### Non-Ambulatory Patients with Unexplained Post-Fracture Symptoms: Was it Fat Embolism Syndrome?

Virginia Kaperick

Children's Wisconsin, Milwaukee, USA. Medical College of Wisconsin, Milwaukee, USA

### Abstract

Although rare in pediatrics, non-ambulatory patients are at high risk for Fat Embolism Syndrome (FES) after injury, which is frequently underrecognized. Two non-ambulatory patients with several fractures were recently hospitalized with multiple symptoms unresponsive to standard therapies. In retrospect they likely had FES.

A 17-year-old male with Duchenne Muscular Dystrophy presented with one week history of altered mental status following multiple fractures sustained in a vehicle accident. Also, the night prior to admission he fell from his wheelchair in the setting of heightened family tensions. Follow-up head CT was without concerns and abnormal laboratory results noted dehydration, borderline low hemoglobin and mildly elevated bilirubin level. He had hypoxia, tachycardia, intermittent hypotension, urinary retention and anorexia. Neurology diagnosed probable post-concussive syndrome, and psychiatry noted concerns of depression. He remained hospitalized for eleven days with gradually improving symptoms although persistent blurry vision. Additional fractures in the leg not previously imaged were found as the cause of persistent difficulties with transfers during hospitalization. Follow up after admission notes significant changes to baseline visual acuity. MRI brain and orbits was normal, and urgent neuro-ophthalmology consult scheduled.

This 9-year-old non-ambulatory boy with severe congenital myopathy presented with hive-like rash, tachycardia, tachypnea and hypoxia, non-responsive to fluids and pain medications, although resolution of rash with antihistamine. He was incidentally noted to have fallen the prior evening and xrays showed several minor fractures in his lower leg. The chest CT showed interstitial fluid but negative for pulmonary embolism. Laboratory tests noted mildly elevated AST and ESR with significant elevation of C reactive protein level. He was hospitalized for five days for hypoxia and tachycardia with no immediate improvement to standard therapies, and no infectious etiology found. He had no history of respiratory issues prior to this event and has had none since.

Fat Embolism Syndrome is a rare potentially life-threatening complication that carries a mortality rate as high as 44% in DMD patients. There is no specific pharmacologic therapy for Fat Embolism Syndrome. Maintaining a high index of suspicion, with early recognition and aggressive supportive therapy, are paramount given the high mortality risk.

Learning points

1. Review of Fat Embolism Syndrome pathophysiology, presentation and why DMD and other non-ambulatory patients are at significantly higher risk.
2. Review specific exam findings, laboratory and imaging findings that can aid in appropriate diagnosis of Fat Embolism Syndrome.
3. Review supportive treatment recommendations for FES including specific precautions in the DMD population.

### Disclosure none declared

### LB36

### Baseline Characteristics in Part 1 of the Ongoing ANDECAL Study (ASH-FOP-201), a Two-Part Phase 2/3 Study of Andecaliximab (anti-matrix metalloproteinase-9 mAb) in FOP

Mona Al Mukaddam[1], Robert Pignolo[2], Edward Hsiao[3], Kelly Wentworth[4,5], Deborah Wenkert[6], Frederick Kaplan[7]

[1]Departments of Orthopedic Surgery and Medicine, The Center for Research in FOP and Related Disorders,Perelman School of Medicine, University of Pennsylvania, Philadelphia, USA. [2]Department of Medicine, Mayo Clinic, Rochester, USA. [3]Division of Endocrinology and Metabolism and the Institute for Human Genetics, Department of Medicine, University of California, San Francisco, San Francisco, USA. [4]Division of Endocrinology and Metabolism, Department of Medicine, University of California, San Francisco, San Francisco, USA. [5]Endocrine Research Unit, Department of Medicine, San Francisco Veterans Affairs Health, San Francisco, USA. [6]āshibio, Inc., Burlingame, USA. [7]Departments of Orthopedic Surgery and Medicine, The Center for Research in FOP and Related Disorders, Perelman School of Medicine, University of Pennsylvania, Philadelphia, USA

### Abstract

**OBJECTIVES:** Describe baseline characteristics in Part 1 of ANDECAL (ANDECAL-1), the dose finding and safety portion of ANDECAL.

**METHODS:** Baseline characteristics in the US-based ANDECAL-1 were summarized and compared to a global FOP Natural History Study (NHS; Pignolo, *Genetics in Medicine*, 2022).

**RESULTS:** ANDECAL-1 enrolled 12 participants: 16.7% male, mean age = 19.1 yr; 92% had the R206H *ACVR1* variant. All had new or ongoing flare-ups (FU) within 2.5 months prior to Baseline. All were ambulatory and needed help with ADLs. Mean Cumulative Analogue Joint Involvement Scale (CAJIS) was 10.7 (max=30 for complete ankylosis). 83% had elevated age-adjusted alkaline phosphatase (mean 145.6% ULN; n=7) and/or P1NP (mean=169.6% ULN; n=6). 41.7% had a history of fall(s). 58% had prior fractures.

Congenital manifestations included hallux valgus (75%), other digital shortening (67%), hip dysplasia or short broad femoral necks (50%), and fusion of cervical vertebrae (83%). The first postnatal symptoms: age of FU, HO, and diagnosis, reported in 10 to 11 participants, occurred on average at ages 7.2 (0.5 to 17), 7.1 (0.5-12), and 7.5 (2 to 14) years, respectively. 33% had undergone a biopsy. The longest delay between first FU or HO and diagnosis was 3 years.

The prevalence of additional FOP manifestations in participants age 15-22 years (mean age=18; n=11)] was evaluated separately to allow a comparison to the 15-<25 group in the NHS (see Table): Osteochondromas were present in 90%; hearing loss in 73%. Most had TL Spine ankylosis; 50% had neck ankylosis. Mean FOP Physical Function Questionnaire (FOP-PFQ) was 47.6% out of 100% (100% indicates complete inability to carry out described tasks).

**CONCLUSIONS**: As shown in the table, baseline mean age, and FOP-PFQ, were similar in ANDECAL-1 and NHS, but in ANDECAL-1 only 16.7% participants are male (vs. 54.4% in NHS).

Importantly, participants in ANDECAL-1 experienced a greater number of new flare-ups prior to baseline than seen in the NHS. In addition, mean baseline CAJIS in ANDECAL-1 was closer to mean baseline CAJIS of the 8-<15 yr old age group in NHS. These differences will be considered in the interpretation of the ANDECAL-1 results.

| | | | ANDECAL-1 | NHS (full cohort) |
|---|---|---|---|---|
| Demographics | N | | 12 | 114 |
| | % Male | | 16.7 | 54.4 |
| | Mean age (years) | | 19.1 | 17.6 |
| | Range (age in years) | | 15-31 | 4-56 |
| Flare-ups | Mean age of first flare-up (years) | | 7.2 | 4.9 |
| | Range (age of first flare-up in years) | | 0.5 - 17 | |
| | Years since last flare-up | Mean (SD) | 0.38 (0.8) | 1.5 (2.6) |
| | | Median (range) | 0.15 (0.02-2.8) | 0.5 (0-15) |
| | Flare-ups in the 12 months before Baseline | Percent with ≥1 flare-up | 92% | 66.7% |
| | | Mean # of Flare-ups/participant | 3.25 | 2.5 |
| | Years since first flare-up | | 12.2 (n=11) | 12.7 |
| | Range (years since 1st FU) | | 5-19 | 0-45 |
| Disease Burden | Mean CAJIS* | | 10.7 | 11.8 |
| | CAJIS* range | | 7-17 | 1-30 |
| | Fractures | | 58% had 1-6 fractures | 19.3% |

| Age restricted cohorts (years): | | ANDECAL-1, age 15 to 22 | NHS-age 15 to <25 subgroup | NHS-age 8 to <15 subgroup |
|---|---|---|---|---|
| CAJIS | N | 11 | 34 | 36 |
| | CAJIS | 10.6 | 13.9 | 9.2 |
| | CAJIS range | 7-17 | 1-26 | 1-20 |
| FOP-PFQ | N | 11 | 33 | 31 |
| | Mean FOP-PFQ | 47.6% | 43.1% | 44.4% |
| | FOP-PFQ range | 20% to 68% | 0 to 100% | 1.9 to 82.7% |

### Disclosure

MAM, ECH, RJP, and KW are site investigators for this study, which is supported by funding from āshibio to the Institution. Clinical trial research support is also received through their institutions from Clementia/Ipsen (MAM, ECH, RJP), Ascendis (ECH, MAM, KW), and Incyte (MAM, ECH, RJP); ECH has served as a consultant on an advisory panel for Regeneron. RJP is a co-inventor for the use of andecalixamab, and receives royalties from the University of Pennsylvania based on a licensing agreement with āshibio. DW is a full-time employee of āshibio receiving salary and options.

### LB37

### Immunomodulatory Therapy for Refractory Fibrodysplasia Ossificans Progressiva: Sustained Clinical Improvement in a Young Child

Arunabh Sinha, Susan Ballinger

Indiana University School of Medicine, South Bend, USA

### Abstract

BACKGROUND:

Fibrodysplasia ossificans progressiva (FOP) is an ultra-rare genetic disorder caused by ACVR1 mutations, characterized by progressive heterotopic ossification (HO) leading to severe disability. Emerging evidence implicates inflammatory pathways, particularly IL-1β, IL-6, TNFα, and mast cell/macrophage activation, as central drivers of HO formation. Immunomodulatory therapies including JAK inhibitors and IL-1 inhibitors represent novel off-label treatment options with preliminary evidence suggesting efficacy in refractory pediatric cases.

PRESENTING PROBLEM:

A 5-year-old boy presented with genetically confirmed FOP (pathogenic de novo ACVR1 variant at age 20 months) and progressive HO refractory to imatinib. Congenital features included the typical bilateral hallux valgus deformities, plus bilateral clasped thumbs (atypical for FOP), and metopic craniosynostosis requiring surgical correction at 8 months. Progressive soft-tissue swelling beginning at 12-18 months was accompanied by reduced neck range of motion. MRI demonstrated paraspinal edema and nuchal ligament thickening.

CLINICAL MANAGEMENT:

Initial therapy with imatinib 100 mg daily starting at age 2 years stabilized inflammatory markers but failed to prevent progressive mobility loss and new HO formation in neck, shoulders, and back over 18 months. At age 3.5 years, treatment was transitioned to tofacitinib (5 mg AM/2.5 mg PM). Over 20 months of follow-up, the patient demonstrated remarkable improvement with reported reduction in previously palpable ossified lesions, significantly improved range of motion in neck and limb joints, increased independent mobility, and improved independence in activities of daily living. Pain episodes decreased in severity, becoming self-resolving and manageable with NSAIDs. Laboratory monitoring showed persistently normal inflammatory markers, renal and hepatic function, with no adverse effects observed.

DISCUSSION:

This case represents one of the youngest patients treated with tofacitinib for FOP and demonstrates sustained clinical benefit over 20 months. The reported regression of palpable ossified lesions is unprecedented in FOP literature, though formal serial imaging confirmation was not obtained. Recent evidence demonstrates IL-1 inhibitors reduce flare activity and improve quality of life in FOP patients, though subcutaneous administration poses theoretical trauma risk. The broad immunomodulatory effects of JAK inhibition may target multiple inflammatory pathways simultaneously, potentially explaining the dramatic response observed. This case supports consideration of immunomodulatory therapies in pediatric FOP patients with progressive disease refractory to conventional management.

### Disclosure

None declared.

### LB38

### Relationships Between Treatment and Health-Related Quality of Life in Pediatric Patients with X-Linked Hypophosphatemia: Longitudinal Real-World Data from the International XLH Registry

Raja Padidela[1], Laura Guazzaroti[2], Francesco Emma[3], Maria Gema Ariceta Iraola[4], Carmen de Lucas Collantes[5], Ola Nilsson[6], Shannon Ferrante[7], Lak Nilusha Kotinkaduwa[7], Kerry Sandilands[8], Annabel Bowden[9], Jennifer E Dent[9], Agnès Linglart[10]

[1]Royal Manchester Children's Hospital and Faculty of Biology, Medicine and Health, The University of Manchester, Manchester, UK. [2]Pediatric Endocrinology Unit, Department of Woman and Child Health, University of Padua, Padua, Italy. [3]Division of Nephrology, Children's Hospital Bambino Gesù-IRCCS, Rome, Italy. [4]Department of Paediatric Nephrology, University Hospital Vall d'Hebron, Autonomous University of Barcelona, Barcelona, Spain. [5]Departamento de Nefrología Pediátrica, Hospital Infantil Universitario Niño Jesús and Departamento de Pediatría, Universidad Autónoma de Madrid, Madrid, Spain. [6]Pediatric Endocrinology Unit and Center for Molecular Medicine, Department of Women's and Children's Health, Karolinska Institutet and University Hospital, Stockholm, Sweden. [7]Kyowa Kirin, Princeton, USA. [8]Kyowa Kirin, Marlow, UK. [9]Spire Outcomes, London, UK. [10]Université Paris Saclay, AP-HP, INSERM, Service endocrinologie et diabète de l'enfant, Centre de référence des maladies rares du métabolisme du calcium et du phosphate, Hôpital Bicêtre Paris Saclay, Paris, France

### Abstract

**OBJECTIVE:** X-linked hypophosphatemia (XLH) is the most frequent form of genetic rickets in which profound hypophosphatemia and abnormal bone metabolism lead to skeletal deformities, muscle weakness, pain, and impaired health-related quality of life (HRQL). This study evaluated associations between current treatment and HRQL in pediatric patients with XLH.

**METHODS:** Data were from pediatric patients (aged 1–17 years) enrolled in the International XLH Registry, who completed the Pediatric Quality of Life Inventory (PedsQL) up to four times (every 6 months) over 18 months. Associations between PedsQL scores and demographic, medical history, and treatment variables was assessed using Wilcoxon/Kruskal–Wallis test or Spearman's rank correlation. Associations between XLH treatment and PedsQL scores were evaluated using linear mixed models, adjusted for confounding factors according to clinical relevance, with treatment-by-time interaction terms.

**RESULTS:** At first PedsQL completion (M0), patients (n=173) had a mean (SD) age of 10.0 (4.58) years; 62% were female; 83% were receiving burosumab and 11% phosphate supplements and/or active vitamin D (Pi/D). Mean (SD) Physical health summary (PHS) and Psychosocial health summary (PSS) scores at M0 were 77.8 (18.02) and 75.2 (16.75), respectively. In bivariate analyses, worse PHS scores at M0 were significantly ($p<0.05$) associated with older age at diagnosis, no family history of XLH, prior orthopedic surgery, and being burosumab naïve; scores also differed by study country. Worse PSS scores at M0 were associated with no family history of XLH, prior orthopedic surgery, and being burosumab naïve; scores also differed by current treatment. In the multivariable linear mixed model (Table 1), adjusted PHS scores over 18 months were significantly worse in patients with prior orthopedic surgery and differed significantly by nationality and family XLH history; adjusted PHS scores differed significantly by current treatment and time (better scores with burosumab and variation across timepoints); the interaction effects were not significant. Adjusted PSS scores over 18 months were significantly worse in patients with prior orthopedic surgery and did not differ significantly by current treatment or time.

**CONCLUSION:** Over 18 months, physical HRQL was better in pediatric patients treated with burosumab than with Pi/D, with a consistent difference over time.

Table 1 Linear mixed effects model for PedsQL PHS and PSS scores over 18 months (n=156)

| Parameter | | PHS | | PSS | |
|---|---|---|---|---|---|
| | | Estimate (95% CIs) | P value | Estimate (95% CIs) | P value |
| Intercept | | 80.68 (72.12, 89.24) | **<0.001** | 77.48 (69.24, 85.73) | **<0.001** |
| Age (years) | | - 0.10 (- 0.74, 0.55) | 0.762 | - 0.13 (- 0.75, 0.49) | 0.683 |
| Sex | Female | Reference | 0.186 | Reference | 0.387 |
| | Male | - 3.90 (- 9.67, 1.86) | | - 2.46 (- 8.01, 3.10) | |
| Country | France | Reference | **0.012** | Reference | 0.472 |
| | Italy | - 3.58 (- 13.4, 6.25) | | - 1.89 (- 11.4, 7.57) | |
| | Spain | - 1.21 (- 10.8, 8.37) | | - 0.40 (- 9.63, 8.82) | |
| | UK | - 11.8 (- 18.8, - 4.77) | | - 5.33 (- 12.1, 1.41) | |
| Age at diagnosis (years) | | 0.28 (- 0.86, 1.42) | 0.629 | 0.33 (- 0.77, 1.42) | 0.562 |
| Surgery | No | Reference | **0.033** | Reference | **0.047** |
| | Yes | - 7.97 (- 15.2, - 0.71) | | - 7.13 (- 14.1, - 0.13) | |
| Family history | No | Reference | **0.014** | Reference | 0.2578 |
| | Yes | 8.35 (2.05, 14.66) | | 4.71 (- 1.36, 10.79) | |
| | Unknown | - 5.72 (- 18.2, 6.72) | | - 2.14 (- 14.1, 9.81) | |
| | | | | | |
| HRQL assessment month[a] | Month 0 | Reference | **0.014** | Reference | 0.3189 |
| | Month 6 | -3.91 (-7.59, -0.23) | | -2.30 (-5.63, 1.03) | |
| | Month 12 | -4.39 (-8.24, -0.54) | | -1.72 (-5.07, 1.62) | |
| | Month 18 | -7.14 (-12.0, -2.27) | | -3.99 (-8.59, 0.62) | |
| Current treatment[a] | Burosumab | Reference | **0.001** | Reference | 0.1052 |
| | Pi/D | -11.3 (-18.0, -4.69) | | -5.06 (-11.1, 1.03) | |
| Treatment by HRQL assessment month | | Interaction effect | 0.123 | Interaction effect | 0.6365 |

[a]Fixed effect, estimates from least square means
Significant values (p<0.05) shown in **bold.**
CIs, confidence intervals; HRQL, health-related quality of life; PedsQL, Pediatric Quality of Life Inventory; PHS, physical health summary; Pi/D, phosphate and/or active Vitamin D; PSS, Psychosocial health summary.

### Disclosure

RP, LG, FE, MGAI, CDLC, ON and AL are study investigators; their institutions received financial support from Kyowa Kirin International. SF, LNK and KS are employees of Kyowa Kirin. AB and JED are employed by Spire Outcomes, which received payment from Kyowa Kirin International for analysis and medical writing.

### LB39

### Effects of Navepegritide and Lonapegsomatropin on Manifestations of Skeletal Dysplasia in Children with Achondroplasia: 52-Week Results from the Phase 2 COACH Trial

Leanne Ward[1,2], Melita Irving[3], Hanne Hove[4], Line Aagaard Nolting[4], Stefan Jackowski[2], Kevin Smit[2], Andrew Tice[2], Sasha Carsen[2], Meng Mao[5], Simon Mejlhede Jensen[6], Michael Makara[5], Sohair Abdelrahman[6], Kim Shimy[6], Allison Komirenko[6], Michael Ominsky[6], Aimee Shu[5], Ciara McDonnell[7,8]

[1]University of Ottawa, Ottawa, Canada. [2]Children's Hospital of Eastern Ontario, Ottawa, Canada. [3]Guy's and St. Thomas' NHS Foundation Trust, Evelina Children's Hospital, London, UK. [4]Copenhagen University Hospital, Rigshospitalet,, Copenhagen, Denmark. [5]Ascendis Pharma, LLC, Palo Alto, CA, USA. [6]Ascendis Pharma A/S,, Copenhagen, Denmark. [7]Children's Health Ireland at Temple Street, Dublin, Ireland. [8]Discipline of Paediatrics, University of Dublin, Trinity College, Dublin, Ireland

### Abstract

### Objectives:

Children with achondroplasia experience manifestations of skeletal dysplasia beyond short stature, including lower extremity malalignment and reduced arm span and spinal canal dimensions, which can potentially lead to pain, altered mobility and functionality, and need for surgeries. Navepegritide is a prodrug of C-type natriuretic peptide (CNP), administered subcutaneously once-weekly and designed to provide continuous exposure to active CNP to counteract the constitutively active fibroblast growth factor receptor 3 in achondroplasia. Adding once-weekly lonapegsomatropin, a prodrug of somatropin, to navepegritide is hypothesized to augment the effects observed with navepegritide monotherapy on endochondral bone formation. Here we report 52-week growth and orthopedic findings from the COACH Trial, which evaluated navepegritide combined with lonapegsomatropin in children with achondroplasia.

### Methods:

COACH enrolled participants aged 2–11 years with achondroplasia into 2 cohorts: treatment-naive (TN) children (n=12) initiated navepegritide (100 µg/kg/week) and lonapegsomatropin (starting dose: 0.30 mg hGH/kg/week) on Day 1; navepegritide-experienced (NE) children (n=9; average prior exposure to 100 µg/kg/week navepegritide: 2.56 years) added lonapegsomatropin to their existing navepegritide monotherapy. Anthropometric measures (arm span) and bone morphometry (spinal canal width and lower extremity alignment, assessed radiographically by central readers) were evaluated through Week 52.

### Results:

Improvements in arm span and arm span Z-score were observed at Week 52 (Figure 1A) beyond those previously reported in trials of monotherapy treatments in children with achondroplasia. Interpedicular distance (IPD) averaged across L1-L5 improved from baseline to Week 52 in both cohorts (TN: +1.7 mm; NE: +1.1 mm). Tibial-femoral angle (TFA) and absolute average TFA Z-score improved in the TN cohort (mean change from baseline to Week 52 of -3.0 degrees and -0.86, respectively), indicating a reduction in lower limb malalignment, while children in the NE cohort maintained an absolute average TFA Z-score within the range for children of average stature (-2 to +2) (Figure 1B).

### Conclusions:

Once-weekly navepegritide combined with once-weekly lonapegsomatropin was associated with improvements in arm span, spinal canal dimensions, and lower limb alignment in children with achondroplasia. These findings suggest that combined treatment with navepegritide and lonapegsomatropin confers enhanced benefits beyond linear growth, addressing structural and functional aspects of skeletal dysplasia in achondroplasia.

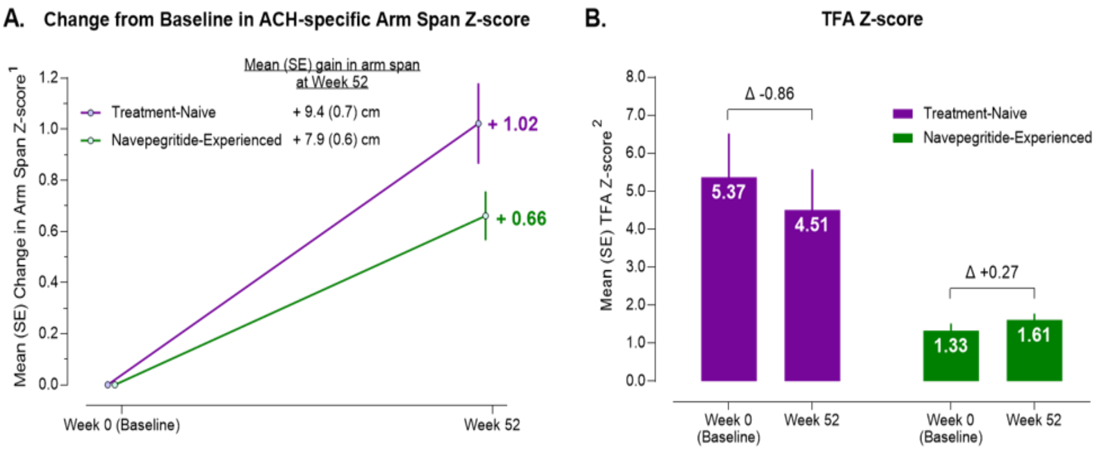

ACH = achondroplasia; SE = standard error; TFA, tibial-femoral angle
Note: TFA Z-scores reported are absolute average values

[1] Children with ACH: Merker A, et al. *Am J Med Genet A* 2018;176(9):1819–1829.
[2] Children of average stature: Sabharwal S, Zhao C. *J Bone Joint Surg Am*. 2009 Oct;91(10):2461-8.

### Disclosure

L Ward: Ascendis Pharma – Clinical Trial Investigator and Grant/Research Support; QED – Clinical Trial Investigator and Grant/Research Support; BioMarin – Consultancy. M Irving: Ascendis Pharma – Consultant, Grant/Research Support, Speakers' Bureau HB Hove: Ascendis Pharma – Consultant and Grant/Research Support.L Nolting: Ascendis Pharma – Grant/Research Support.K Smit: Medtronic – Grant/Research Support; ZimVie – Grant/Research Support; SpinoModulation – Grant/Research Support; Ascendis Pharma – Grant/Research Support; Ultragenix – Grant/Research Support.A Tice: Ascendis Pharma – Grant/Research Support. M Mao, SM Jensen, MA Makara, SG Abdelrahman, KJ Shimy, AS Komirenko, MS Ominsky, AD Shu: Ascendis Pharma – Employees C McDonnell: Ascendis Pharma – Consultant, Grant/Research Support, Speakers' Bureau; BioMarin – Consultant and Speakers' Bureau

### LB40

### Cosegregation of Disease with a Deep Intronic PHEX Variant in a Three-Generation Family: Evidence from Linkage Analysis and Minigene Assay

Chelsey Grimbly[1,2], Oana Caluseriu[1,2], Wanling Pan[1], Laurie Mereu[1], Elizabeth Rosolowsky[1,2], Chaitanya Gandhi[1], Frank Rauch[3,4], Leanne Ward[5,6], R Todd Alexander[1,2]

[1]University of Alberta, Edmonton, Canada. [2]Women and Children's Health Research Institute, Edmonton, Canada. [3]Shriner's Hospital for Children, Montreal, Canada. [4]McGill University, Montreal, Canada. [5]University of Ottawa, Ottawa, Canada. [6]Children's Hospital of Eastern Ontario, Ottawa, Canada

### Abstract

### Introduction

XLH is the most common genetic form of rickets, caused by pathogenic variants in the *PHEX* gene. However, exon sequencing fails to identify a pathogenic *PHEX* variant in 10-15% of clinically affected individuals. We describe a three-generation family with XLH features but negative *PHEX* analysis on exon gene panels. RNA sequencing of urine cells identified a deep intronic *PHEX* variant in the proband (Grimbly et al, 2023). We pursued genotyping/phenotyping of family members and *in vitro* splicing analysis of the variant to understand its functional significance.

### Methods

Family members of the proband were eligible for clinical, biochemical, and radiological phenotyping. DNA was isolated from buccal swabs and the known PHEX deep intronic variant (c.2147+1197 A>G) was sequenced. A minigene assay was performed to assess the splicing impact of the PHEX variant *in vitro*. A genomic fragment containing the variant of interest and flanking intronic sequences was cloned into a reporter plasmid, transfected into HEK293T cells and splicing patterns analyzed by RT-PCR and Sanger sequencing.

### Results

Sixteen family members were enrolled. Eight (8/16) members had a clinical diagnosis of XLH and all eight had the deep intronic *PHEX* variant (c.2147+1197 A>G). The clinical phenotype was highly variable including different degrees of short stature, bone pain, limb bowing, enthesopathies, dental abscesses, and/or craniosynostosis; overall, all individuals had at least 3 features consistent with XLH. Three affected yet untreated individuals had intermittently normal serum phosphate (0.97-1.16 mmol/L) and two individuals had elevated serum 1,25 dihyroxyvitamin D concentrations (180-208 pmol/L, N 43-168 pmol/L). The minigene assay demonstrated that the intronic variant resulted in the insertion of an 84 bp pseudoexon between exons 21-22.

### Conclusion

We demonstrate that a deep intronic *PHEX* variant segregates with clinical phenotype in a three-generation family, further supporting this variant as disease-causing. Interestingly, serum phosphate and 1,25 dihydroxyvitamin D concentrations were normal in some individuals who nevertheless had phenotypic features of XLH. Urine RNA sequencing is a diagnostic tool that can aid in cases where traditional gene panels do not identify pathogenic variants. Further studies are needed to elucidate how intronic variants may affect clinical phenotypes.

### Disclosure

None declared

### LB41

### Dentoalveolar defects in the Hyp mouse model of X-linked hypophosphatemia

Parastoo Asadi[1], Aonjittra Phanrungsuwan[2], Brian L Foster[2], Natalie Reznikov[1,3,4], Marc D McKee[1,3]

[1]Dental Medicine and Oral Health Sciences, McGill University, Montreal, Canada. [2]College of Dentistry, The Ohio State University, Coumbus, OH, USA. [3]Anatomy & Cell Biology, McGill University, Montreal, Canada. [4]Bioengineering, McGill University, Montreal, Canada

### Abstract

**OBJECTIVES:**

X-linked hypophosphatemia (XLH) is a hereditary disorder caused by inactivating mutations in the PHEX gene leading to renal phosphate wasting and accumulation of mineralization inhibitors in bones and teeth that cause mineralization defects (osteomalacia and odontomalacia). The *Hyp* mouse model of XLH carries a truncating *Phex* mutation whose phenotype closely reproduces the skeletal and dentition mineralization defects observed in XLH patients. Traditional microscopies have helped in identification of mineralization abnormalities in XLH/*Hyp*, but they are generally destructive and limited in their ability to image and quantify dentoalveolar structures in 3D. Here we have developed a nondestructive, high-resolution 3D imaging approach for visualizing and quantitatively characterizing dentoalveolar defects in the mandible of the *Hyp* mouse model of XLH.

**METHODS:**

Hemimandibles from *Hyp* and wild type (WT) mice were iodine-stained, critical point-dried and imaged using submicron X-ray microcomputed tomography. Deep learning-assisted segmentation and quantitative analysis were performed using Dragonfly software to assess tissue volumes, thickness, mandibular morphology, alveolar bone architecture mineralization defects in dentoalveolar tissues.

**RESULTS:**

*Teeth*: Molars of *Hyp* mice exhibited increased unmineralized tissue (predentin, cementoid) and reduced mineralized dentin and cementum volume and thickness, particularly in roots. Interglobular dentin defects were evident, and the dentin mineralization front showed enlarged calcospherites and increased mineralized surface irregularity. In the furcation region, dentin had layered mineralization defects with an altered dentino-cemental junction. *Alveolar bone architecture: Hyp* mice exhibited increased osteoid accumulation, reduced mineralized bone volume, and elevated bone surface area and surface-to-volume ratio. Osteocyte mineral lacunae were enlarged but reduced in number. Fractal dimension analysis indicated a modest increase in mineralized bone surface irregularity. *Mandibular morphology*: Compared to WT controls, *Hyp* mice exhibited reduced mandibular base length, shorter crowns, and decreased cusp height. These changes indicate that mineralization defects extend beyond tissue composition to affect dentoalveolar architecture across multiple scales.

**CONCLUSIONS:**

μCT imaging combined with deep learning-assisted segmentation enables nondestructive 3D assessment of dentoalveolar abnormalities in XLH/*Hyp*. Such findings help to explain the oral health manifestations of XLH. We provide a robust workflow/analysis pipeline for dentoalveolar characterization, enabling assessment of dental and alveolar bone mineralization defects, disease progression, and therapeutic outcomes.

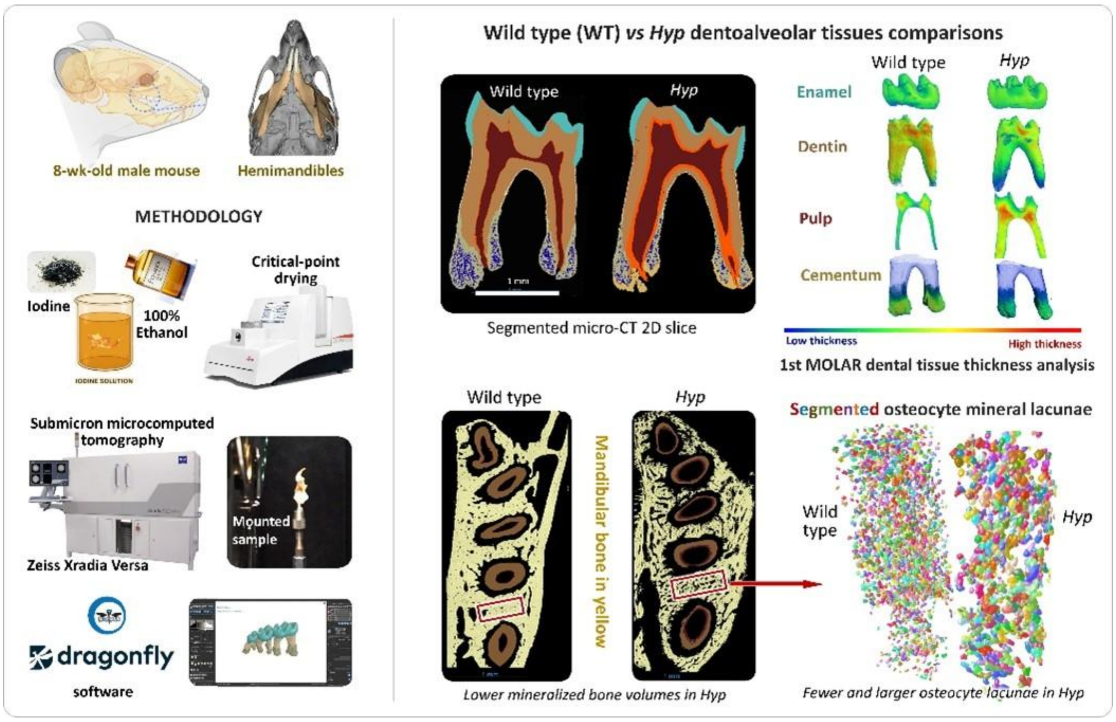

### Disclosure

None declared

### LB42

### Autosomal Dominant Hypocalcemia Type 1 and Type 2: Baseline Burden of Disease and Quality of Life in Pediatric Participants in the CLARIFY Disease Monitoring Study

Outi Makitie[1], Matthew Benson[2], Peter Tebben[3], Agnes Linglart[4], Christina Jacobsen[5], Tariq Ahmad[6], Justine Bacchetta[7], Halley Wasserman[8], Stefano Mora[9], Nina Ma[10], Takuo Kubota[11], Jessica Chen[12], Dingfeng Li[12], Scott Adler[12], Raja Padidela[13]

[1]Children's Hospital, University of Helsinki and Helsinki University Hospital, Helsinki, Finland. [2]Nemours Children's Health, Division of Endocrinology, Diabetes & Metabolism, Jacksonville, USA. [3]Yale University School of Medicine, New Haven, USA. [4]Paris Saclay University, AP-HP, Hopital Bicetre Paris Saclay, Le Kremlin-Bicêtre, France. [5]Boston Children's Hospital, Boston, USA. [6]University of California San Francisco - Benioff Children's Hospital, Oakland, USA. [7]Hospices Civils de Lyon, INSERM1033, Lyon, France. [8]Cincinnati Children's Hospital Medical Center and the University of Cincinnati, Cincinnati, USA. [9]Laboratory of Pediatric Endocrinology, Department of Pediatrics, IRCCS Ospedale San Raffaele, Milano, Italy. [10]Children's Hospital Colorado and University of Colorado Anschutz Medical Center, Aurora, USA. [11]Department of Pediatric Nephrology and Metabolism, Osaka Women's and Children's Hospital, Osaka Prefecture Hospital Organization, Izumi, Japan. [12]Calcilytix Therapeutics, a BridgeBio Company, San Francisco, USA. [13]Royal Manchester Children's Hospital & Faculty of Biology Medicine and Health, University of Manchester, Manchester, UK

### Abstract

OBJECTIVES:

Autosomal dominant hypocalcemia types 1 and 2 (ADH1/2) are rare disorders caused by activating variants in CASR and GNA11, respectively, and are characterized by hypocalcemia and hypercalciuria. This study aims to characterize disease burden and quality of life (QoL) in children with ADH1/2.

METHODS:

The CLARIFY disease monitoring study (NCT05227287) is an ongoing, global, multicenter, longitudinal, observational study to characterize disease burden, management patterns, and progression in patients with ADH1/2 over a 5-year period. Among 95 enrolled participants, 25 are children (<18 years; 22 with ADH1 and 3 with ADH2). Their mean age at enrollment was 9.0 years, and 64% (n=16) were female. Baseline self-reported QoL and disease burden were assessed using the 10-Item Short Form Health Survey (SF-10) and a sponsor-developed ADH1/2 questionnaire to collect information on clinical manifestations, symptoms, and treatment history.

RESULTS:

Baseline mineral parameters are demonstrated in the Table. Neuromuscular symptoms were the most frequently reported symptoms impacting QoL: muscle cramps/spasms (60% [15]), paresthesia (44% [11]), followed by impaired concentration (36% [9]) and sleep difficulty (28% [7]). Symptoms reported as having the greatest overall impact on daily functioning were muscle cramps/spasms (36% [9]), paresthesia (20% [5]), sleep difficulty (20% [5]), and impaired concentration (20% [5]). The most affected activities of daily living included peer interactions (24% [6]), extracurricular activities (20% [5]), and regular exercise (20% [5]). Reported as norm-based summary scores relative to the US general population, SF-10 results indicated a greater impairment on physical health (mean 44.1 ± 11.5) than on psychosocial health (mean 50.3 ± 13.6). Disease burden was further highlighted by hospitalization history in 56% (14) of participants (28% [7] with 1-2 hospitalizations; 28% [7] with ≥3 hospitalizations) and prior intravenous calcium treatment for acute hypocalcemia in 50% (12) (25% [6] with 1-2 infusions; 25% [6] with ≥3 infusions).

CONCLUSIONS:

As the largest pediatric ADH1/2 cohort to date, this study demonstrated substantial baseline disease burden marked by chronic neuromuscular symptoms, functional impairment, and recurrent severe hypocalcemia requiring hospitalization and intravenous calcium. Long-term prospective data are needed to better define risk factors and guide monitoring and management strategies in children with ADH1/2.

Table: Bone and Mineral Parameters at Baseline

| Characteristic / Parameter | n/N | % | Definition (Reference Range/Threshold) |
|---|---|---|---|
| Hypocalcemia | 23/25 | 92% | 0–<1 year: <2.1 mmol/L;<br>1–17 years: <2.15 mmol/L |
| Low parathyroid hormone | 20/25 | 83% | < 15 ng/L |
| Hyperphosphatemia | 21/25 | 84% | 0–<1 year: >2.5 mmol/L;<br>1–12 years: >1.9 mmol/L;<br>13–17 years: >1.5 mmol/L |
| Hypomagnesemia | 2/25 | 8% | <0.62 mmol/L |
| Normal total 25-OH Vitamin D | 21/25 | 84% | 50–125 nmol/L |

### Disclosure

Outi Makitie: speaker’s fees and compensation for consultancy and participation in advisory board from Kyowa Kirin, Novo Nordisk, Merck, Sandoz, Alexion, BridgeBio, Inozyme Pharma and Ascendis Pharma; Matthew Benson: Novo Nordisk-research support, BridgeBio-research support in ADH related to this abstract, Sanofi-consultant fees, Beta Bionics-consultant fees, Neurocrine Biosciences research support, Enable Biosciences-research support, KiHealth-salary and stock options, Alexion Pharmaceuticals-consultant fees; Peter Tebben: Research funding from and consulting for Calcilytix; Agnès Linglart: Received fees from BridgeBio, Biomarin, Inozyme, Alexion, Kyowa Kirin, NovoNordisk, Merck, Pfizer; Christina Jacobsen: None declared; Tariq Ahmad: Institutional research funding for clinical trials from Calcilytix; Justine Bacchetta: None declared; Halley Wasserman: Consultant for Kyowa Kirin, Alexion; Stefano Mora: Consultant for Ascendis Pharma, Alexion, Biomarin, Calcilytix, Kyowa Kirin; Nina Ma: Peer-reviewer royalties from UpToDate, Site investigator for industry-sponsored clinical trials (Calcilytix, Inozyme, Ultragenyx); Takuo Kubota: Speaker’s fees and compensation for consultancy from Teijin Pharma; Jessica Chen: Employee of BridgeBio and own company stock; Dingfeng Li: Employee of BridgeBio and own company stock; Scott Adler: Employee of BridgeBio and own company stock; Raja Padidela: Consultant for Alexion, Calcilytix and Kyowa Kirin.

### LB43

### Rapid Improvement with Burosumab in Severe Fibrous Dysplasia and Phosphate Wasting

Anne Dittrich[1], Natasha Appelman-Dijkstra[2]

[1]Amalia Children's Hospital, Radboudumc, Nijmegen, Netherlands. [2]Leiden University Medical Center, Leiden, Netherlands

### Abstract

BACKGROUND

We present a 6 year old boy with an unremarkable medical history.

PRESENTING PROBLEM

Since 3 months he experienced a waddling gait with frequent falls. He did not report pain, there were no associated symptoms or fractures. On physical examination, the left leg was 0.9 cm shorter than the right and external rotation of the left hip was restricted. His gait was waddling and wide-based. The remainder of the physical examination was unremarkable. Considering the broad differential diagnosis, laboratory testing revealed elevated alkaline phosphatase (94% bone-derived), low phosphate with renal wasting and increased P1NP. PTH, calcium and vitamin D levels were normal but FGF-23 levels were inappropriately elevated. Imaging revealed multiple skeletal lesions (figure 1a) and the diagnosis polyostotic fibrous dysplasia was made. Hormonal screening to exclude McCune-Albright syndrome did not reveal any endocrinopathies.

CLINICAL MANAGEMENT

Supplementation with calcium, active vitamin D, and phosphate was initiated. Pamidronate infusions were started to improve bone strength. Unfortunately, his condition worsened, with increased waddling, fatigue and a femur fracture needing surgery. In time repeated radiographs demonstrated progressive varus deformity of both femora (Figure 1b) needing corrective osteotomy and fixation (figure 1c). Despite supplementation, phosphate levels stayed low and the deformities were progressive with recurrent stress fractures needing repeated surgery. Unfortunately, in the Netherlands burosumab is not reimbursed in conditions other then XLH and TIO. However, the manufacturer was willing to provide burosumab on the condition that, if the treatment was successful, the health care insurance would provide reimbursement. After a single dose, his serum phosphate levels normalized. His condition has since improved: he is more active, and follow-up radiographs show no further progression of skeletal deformities or fractures.

DISCUSSION

This case illustrates the clinical course of severe polyostotic fibrous dysplasia. Despite conventional management, the patient experienced progressive skeletal deformities and worsening gait. Initiation of burosumab led to rapid normalization of serum phosphate, clinical improvement, and stabilization of skeletal lesions. This case highlights that in severe manifestations of fibrous dysplasia with persistent phosphate wasting, burosumab should be considered a therapeutic option and should be available for children with disease progression on maximal therapy.

*Figure 1a, extensive fibrous dysplasia lesions, b; accompanied by progressive coxa vara, c; after corrective osteotomy and fixation*

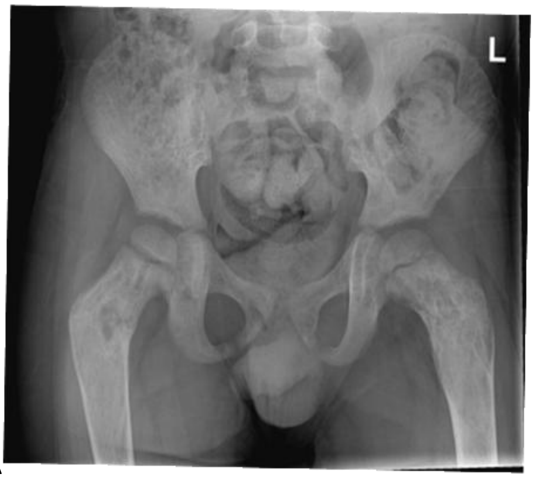

A

B

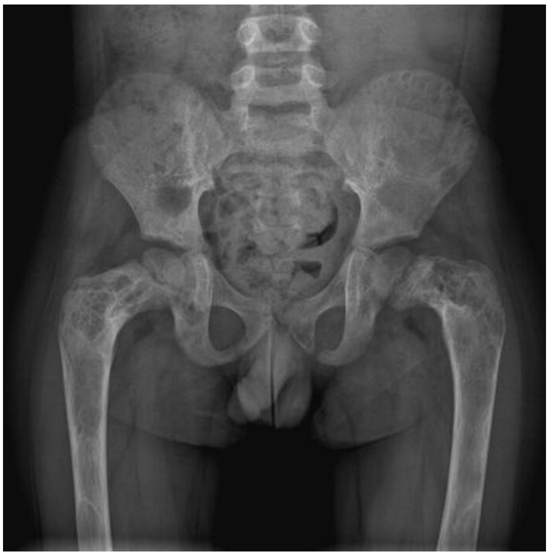

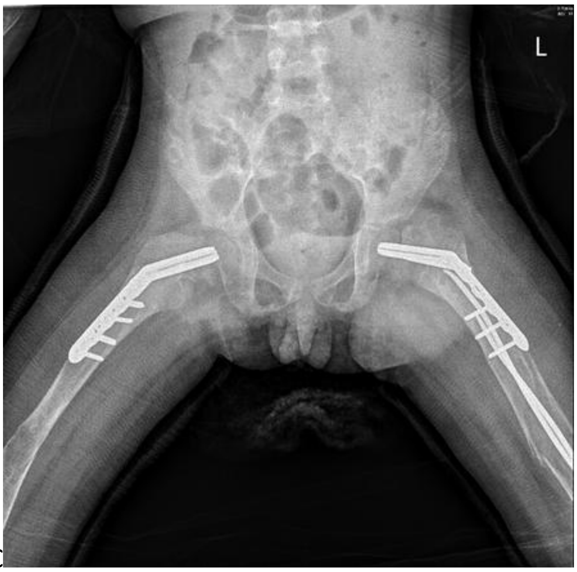

C

### Disclosure

None declared

**LB44**

Abstract withdrawn

### LB45

### Same Mutation, Different Outcomes: Impact of Early Intervention on Osteogenesis Imperfecta Type VIII in Four Karen Patients with P3H1 c.2055+86A>G Founder Mutation

Tawnie Graff[1], Lauren McClure Yauch[2]

[1]Blank Children's Hospital, Des Moines, USA. [2]Gillette Children's Hospital, Saint Paul, USA

### Abstract

Background

Osteogenesis imperfecta (OI) type VIII is a rare autosomal recessive form caused by mutations in the P3H1 gene, which impairs collagen post-translational modification. Null mutations typically produce a perinatally lethal or severe phenotype. The P3H1 c.2055+86A>G (IVS14+86A>G) variant is a founder mutation exclusive to the Karen people, an ethnic group native to Myanmar and Thailand, many of whom live in refugee camps due to ongoing conflict. All 11 previously reported patients with this variant had severe phenotypes with progressive limb deformities and non-ambulatory status. Little is known about the impact of treatment on clinical outcomes in this population.

Case Series

Three Karen siblings (P1–P3; ages 13, 9, and 6 years) presented to our clinic after immigrating to the US following six years in a Thailand refugee camp. None had received prior OI-directed care. All three demonstrated triangular facies, white sclerae, severe short stature, non-weight-bearing status, numerous fragility fractures, popcorn epiphyses, and long-bone deformities. Genetic testing confirmed homozygosity for the P3H1 c.2055+86A>G variant.

P4 presented at age 5 months, born full-term via cesarean section to a mother with undiagnosed bone fragility who carried the same variant. P4 had multiple rib fractures, vertebral compression fractures, and healing femur fractures at birth.

P1–P3 initiated multidisciplinary care including bisphosphonate therapy, orthopedic intervention, and physical medicine. Over the treatment course, all three demonstrated decreased annual fracture rates, improved DXA scores, and enhanced mobility, independence, and quality of life. P4 began multidisciplinary care and bisphosphonate therapy at 5 months. She was walking at 18 months old and she remains ambulatory, using her wheelchair sparingly.

Despite sharing the same genotype, P1–P3 exhibited severe phenotypes with marked growth deficiency, rhizomelic limb shortening, and non-weight-bearing status, whereas P4 demonstrated a moderate phenotype with mild growth deficiency, proportional limb lengths, and ambulatory status.

Discussion

To our knowledge, P4 is the first reported ambulatory individual with this variant, likely attributable to early multidisciplinary intervention. This case series provides critical evidence that early diagnosis and treatment can meaningfully modify outcomes in severe recessive OI and underscores the need for international standards to improve access to care in resource-limited settings.

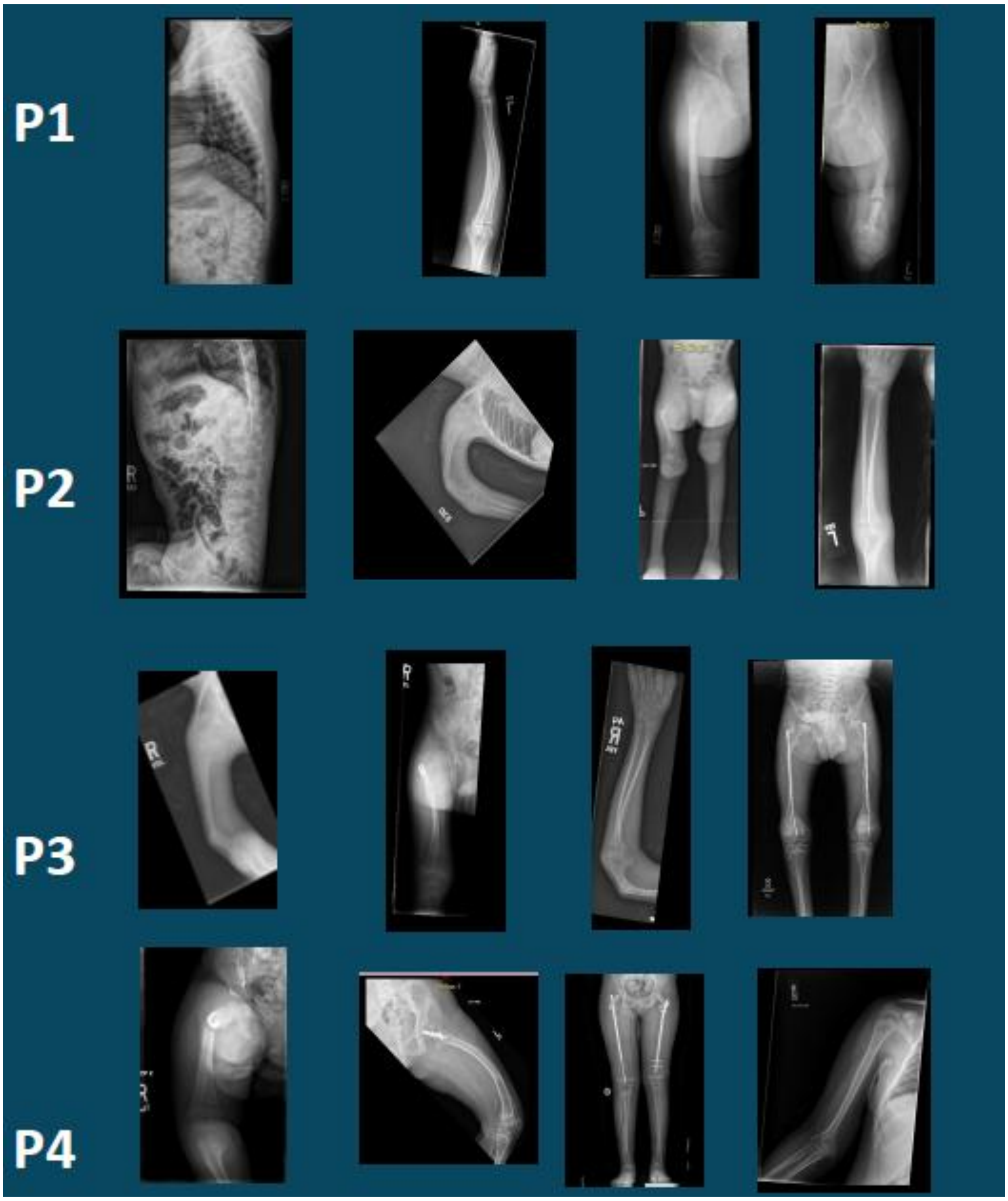

Figure 1: Radiographic images of P1-P4 at time of presentation and post-surgical intervention.

### Disclosure none declared

**LB46**

Abstract Withdrawn

**LB47**

Abstract Withdrawn

**LB48**

Abstract withdrawn

### LB49

### Physiotherapy Rehabilitation after Bilateral Femoral Lengthening in an Adolescent with Achondroplasia: A Clinical Case

Letícia Álava Rubín[1], Inês Alves[2]

[1]APCE, Évora, Portugal. [2]ANDO Portugal, Évora, Portugal

### Abstract

**BACKGROUND**

Achondroplasia is a skeletal dysplasia with an estimated prevalence of approximately 1:25.000 births. Bilateral femoral lengthening is an invasive surgical approach that allows increase of height that may also improve functional outcomes. For this purpose, post-operative rehabilitation is advised to reduce complications by enhancing soft tissues adaptation and recovery.

**PRESENTING PROBLEM**

A 13-year-old girl with achondroplasia underwent bilateral intramedullary femoral lengthening in October 2025 with magnetic nails with strict non-weight bearing throughout all process and use of wheelchair. The patient was referred for outpatient rehabilitation during the full process, still ongoing. Rehabilitation process started one week after surgery. During the first 6 post-surgery months, comprising full distraction phase and beginning of bone consolidation, goals focused on improving joint mobility, muscle strength, positioning, orthosis management, and functional independence in daily activities.

**CLINICAL MANAGEMENT**

During these 6 post-surgery months, rehabilitation was delivered in an outpatient setting following a structured protocol from a Physical Medicine and Rehabilitation team, experienced in managing cases of children with achondroplasia. Physiotherapy was provided three times weekly and, from month 4 (week 12), adjusted to two land-based sessions plus one aquatic therapy session. A weekly psychology session was also offered as a home exercise program (HEP) for the patient and family. Outcomes were monitored through clinical assessment of joint range of motion, muscle strength, and functional independence.

**DISCUSSION**

After 53 physiotherapy sessions, favorable progress was observed in muscle strength, joint mobility, and functional independence, including self-positioning, and orthosis management. This case supports early, intensive, protocol-driven, multidisciplinary rehabilitation after femoral lengthening in adolescents with achondroplasia and suggests that aquatic physiotherapy may be a useful adjunct. The absence of standardized pre-operative measurements limits comparison across time points and highlights the need for prospective use of standardized outcome measures in future cases.

### Disclosure

None declared

### LB50

### Vanishing Masses: The Unexpected Resolution of Idiopathic Tumoral Calcinosis

Anne Dittrich, Petra van Setten, Ellen Schatorjé

Amalia Children's Hospital, Radboudumc, Nijmegen, Netherlands

### Abstract

BACKGROUND

We present a12 year old girl, of Antillean descent, with an unremarkable medical history.

PRESENTING PROBLEM

She presented with a dull pain in both hips for a year. The pain started spontaneously (no trauma or illness) in both hips at the same time. The pain was equal on both the left and right side and has stayed roughly the same over time. The pain increased with movement, and she sometimes had to stop activities because of it. There was no pain at other sites. Family history was negative. On physical examination, firm masses were palpated just above the greater trochanters at both sides, the remainder of the examination was unremarkable. X-ray of the pelvis showed bilateral extra-articular tumoral calcinosis, proximal to the greater trochanter (figure 1).

CLINICAL MANAGEMENT

Extensive additional investigations were initiated; there was a mildly elevated parathyroid hormone level (21 pmol/l), with normal values of calcium and phosphate (in both blood and urine), alkaline phosphatase, vitamin D, FGF-23, osteoblast- and osteoclast activity and kidney function. There was no evidence of autoimmune disease. DXA showed normal bone density. Exome sequencing was performed, followed by analysis of sequence and copy number variants in the STX16 (NM_001001433.3), SAMD9 (NM_017654.4), XPR1 (NM_004736.4), FAM20C (NM_020223.4), FGF23 (NM_020638.3), GALNT3 (NM_004482.4), and KL (NM_004795.4) genes. No abnormalities were found. She was diagnosed with idiopathic tumoral calcinosis. After 12 months, a repeat pelvic X-ray showed that the tumoral calcinosis had resolved (figure 1). After two years, the parathyroid hormone level had normalized. No other abnormalities occurred during follow up.

DISCUSSION

Tumoral calcinosis is characterized by a deposit of calcium phosphate crystals in the extra-articular soft tissues, which are rare benign conditions, especially in children (1). The treatment consists of a precocious and complete surgical excision (2). In our case, however, we demonstrate that the calcifications can also resolve spontaneously. This, of course, does not guarantee a similar course in the future, as recurrence might occur (3). Nevertheless, it does support a watchful waiting approach, as long as the calcifications do not cause significant pain or functional limitation.

References

1. Rajaonarison Ny Ony Narindra LH, Nirinaharimanitra VS, Hunald FA, Ahmad A. Two new cases of pediatric tumoral calcinosis. J Surg Case Rep. 2020 Dec 17;2020(12):rjaa481. doi: 10.1093/jscr/rjaa481. PMID: 33365118; PMCID: PMC7745145.

2. Chebbi W, Belhaj G, Belhaj Messaoud A. Tumoral calcinosis: a rare disease. Tunis Med. 2021 Feb;99(2):306-310. English. PMID: 33899204; PMCID: PMC8715796.

3. Janssen MCH, de Sévaux RGL. Tumoral calcinosis. J In-herit Metab Dis 2010; 33: 91-92

Figure 1 Above pelvic X-ray at preentation showing bilateral tumoral calcinosis. Lower pelvic X-ray showing that the tumoral calcinoses have resolved.

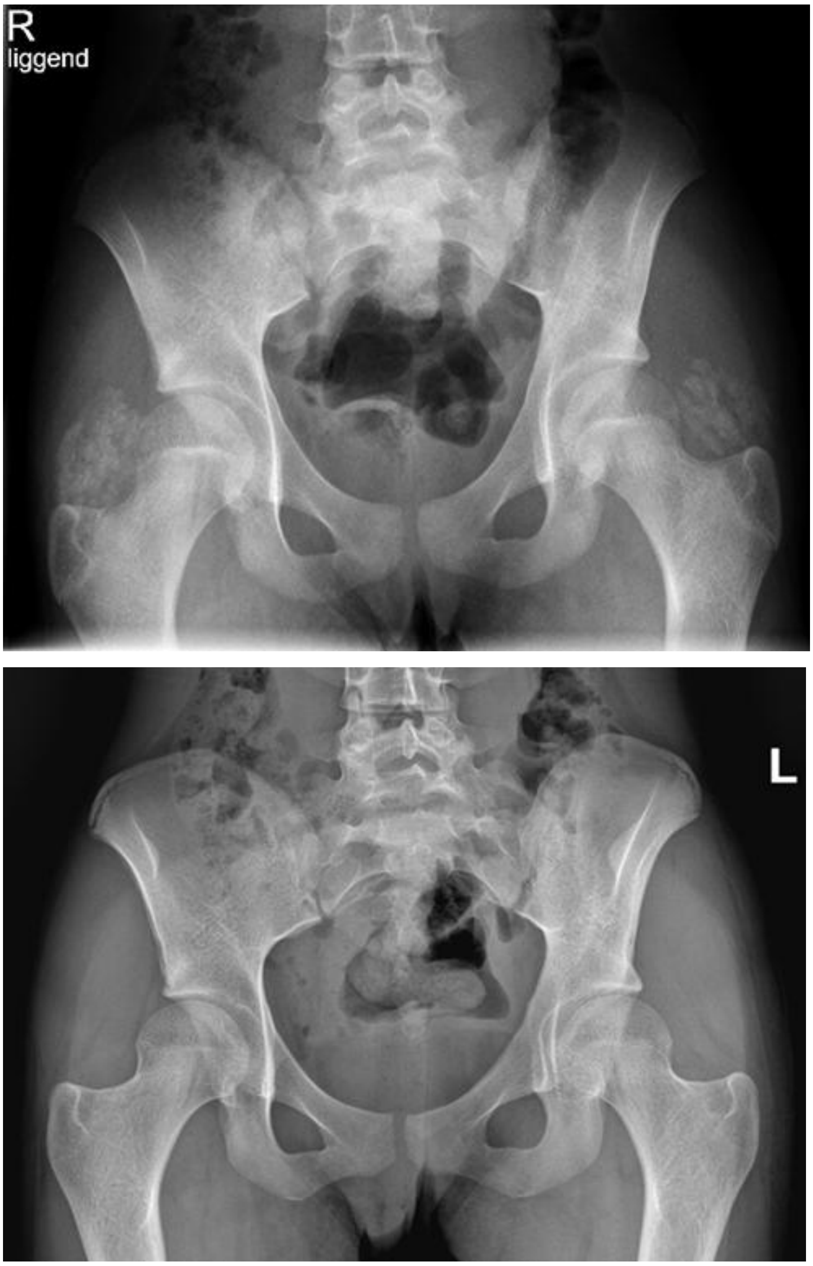

### Disclosure none declared
